# Comparative Evaluation of Large Language Models in Responding to Common Misconceptions Regarding Periodontal Disease and Oral Hygiene

**DOI:** 10.3390/diagnostics16193158

**Published:** 2026-09-28

**Authors:** İsmail Gül, Resül Çolak, Merve Küçükoğlu Çolak, Orhan Cicek

**Affiliations:** 1 Department of Periodontics, Faculty of Dentistry, Zonguldak Bülent Ecevit University, 67600 Zonguldak, Türkiye; r.colak@beun.edu.tr (R.Ç.); mervekucukoglu@beun.edu.tr (M.K.Ç.); 2 Department of Orthodontics, Faculty of Dentistry, Zonguldak Bülent Ecevit University, 67600 Zonguldak, Türkiye; orhancicek@beun.edu.tr

**Keywords:** periodontal disease, misconceptions, artificial intelligence, large language models, health literacy

## Abstract

**Background/Objective:** As large language models (LLMs) are increasingly used to obtain oral health information, their ability to provide accurate and comprehensive responses to patient-focused periodontal questions derived from commonly reported misconceptions is increasingly relevant to patient education. This study aimed to evaluate the performance of contemporary LLMs in answering such questions with respect to accuracy, comprehensiveness, readability, and temporal consistency. **Methods**: Following a comprehensive review of the existing literature, structured patient-focused periodontal questions derived from commonly reported misconceptions were presented to seven different LLMs using a standardized prompt, and their performance was compared. Responses were evaluated for accuracy and comprehensiveness using a Likert scale, while readability was assessed using the Simple Measure of Gobbledygook (SMOG), the Flesch Reading Ease (FRE) score, and the Flesch–Kincaid Grade Level (FKGL). **Results**: The evaluated LLMs demonstrated uniformly high performance across the benchmark, with no individual response scoring below the scale midpoint (score < 3) for accuracy or comprehensiveness, confirming the absence of clinically unsafe or misleading information. Although statistically significant inter-model differences were observed for accuracy (*p* < 0.001, Kendall’s W = 0.197) and comprehensiveness (*p* < 0.001, Kendall’s W = 0.257), the rank-based effect sizes were small, indicating that relative rankings reflect minor numerical variations rather than clinically substantial performance gaps. Regarding readability, absolute metrics across all models revealed a high level of structural and linguistic difficulty that exceeds layperson health literacy requirements. Mean FRE scores across models ranged from 39.5 ± 9.1 to 52.1 ± 12.0 (indicating “difficult” to “fairly difficult” reading ease), mean FKGL scores ranged from 9.1 ± 2.2 to 10.8 ± 1.3 (requiring a 9th to 11th-grade education level), and mean SMOG scores ranged from 11.8 ± 1.6 to 14.0 ± 2.2. Statistically significant differences with large effect sizes were observed among models for FRE (*p* < 0.001, partial η_p_^2^ = 0.308, η_G_^2^ = 0.178), FKGL (*p* < 0.001, partial η_p_^2^ = 0.162, η_G_^2^ = 0.104) and SMOG (*p* < 0.001, partial η_p_^2^ = 0.217, η_G_^2^ = 0.140), with DeepSeek V4 and Gemini 3.1 exhibiting relatively more favorable readability profiles. In the temporal consistency analysis, significant differences in change scores were observed only for SMOG (*p* = 0.004), whereas no significant temporal changes occurred for accuracy, comprehensiveness, FRE, or FKGL. **Conclusions**: Contemporary LLMs provide uniformly accurate and clinically safe responses to patient-focused periodontal misconceptions, with scores consistently concentrated at the ceiling. However, absolute readability metrics demonstrate that AI-generated outputs consistently exceed standard public health literacy comprehension levels, presenting a critical barrier for general patient communication that necessitates targeted optimization.

## 1. Introduction

Individuals make important health-related decisions based on their beliefs regarding the severity of diseases, their perceived susceptibility to health risks, and the effectiveness and safety of recommended healthcare interventions [1]. Consequently, the informational environment to which individuals are exposed plays a pivotal role in shaping health beliefs and behaviors [2]. In recent years, the widespread dissemination of health-related misinformation and misconceptions has emerged as a major public health concern because of its potential to mislead individuals and adversely affect population health [3,4]. Exposure to health misinformation has been shown to reinforce erroneous health beliefs and undermine the adoption of evidence-based preventive health behaviors [2].

Periodontal disease is one of the most prevalent oral diseases worldwide [5,6]. Given the high global burden of periodontal disease, misconceptions regarding periodontal health may affect a substantial proportion of the population. These misconceptions are often influenced by limited health literacy, religious beliefs, and sociocultural misconceptions [7]. Because such beliefs are deeply embedded within communities, they can be difficult to modify through conventional public health interventions. Studies conducted across diverse populations have consistently demonstrated that misconceptions regarding oral health are widespread [8,9,10]. In a study by Gowdar et al., approximately half of the participants were found to hold at least one misconception regarding dental treatment. The same study further reported that 46.52% of participants believed that visiting a dentist was unnecessary in the absence of dental pain [11]. Such misconceptions may negatively influence oral hygiene practices, utilization of preventive dental services, and treatment-seeking behaviors. Indeed, these false beliefs can shape people’s perceptions of periodontal diseases and influence their decisions regarding seeking dental care and accepting treatment [7].

Beyond their impact on preventive behaviors and treatment-seeking, periodontal misconceptions may also complicate the diagnostic process. Patients who rely on inaccurate beliefs may misinterpret or underreport symptoms, delay seeking professional care, or provide incomplete or biased medical histories, thereby reducing the reliability of information obtained during the initial clinical assessment [12,13]. In this context, LLMs capable of identifying and correcting common misconceptions may serve not only as effective patient education tools but also as valuable resources that support more informed pre-diagnostic information seeking [13]. Furthermore, by improving the quality of patient-reported histories and complementing clinical decision support during the early stages of patient evaluation, these models have the potential to enhance the overall diagnostic process. Therefore, identifying misconceptions related to oral health and understanding the factors underlying their persistence are essential for developing effective oral health education and promotion strategies [14,15,16].

Rapid advances in digital health technologies have substantially transformed the way individuals access and seek health information. As part of this transformation, large language models (LLMs) have become increasingly integrated into healthcare, serving as novel tools for health information retrieval, symptom assessment, patient education, and clinical decision support [17,18,19]. Particularly in specialized disciplines such as dentistry, the accuracy of the information generated by these systems, their contribution to clinical decision-making, and their reliability in patient education have become major areas of research interest in recent years [8,20,21].

Given the rapid evolution and frequent updates of LLMs, evaluating their performance in terms of accuracy and comprehensiveness has become increasingly important [22]. Studies focusing on patient education have demonstrated that educational materials generated by LLMs can improve patient knowledge, reduce treatment-related anxiety, and facilitate patient–clinician communication compared with conventional educational approaches [23]. Furthermore, ChatGPT-4 and Gemini have been reported to provide largely accurate, comprehensible, and up-to-date responses to the oral health frequently asked questions published by the World Dental Federation (FDI), suggesting that these systems may serve as reliable sources of information for patient education [24]. Building upon our previous methodological framework evaluating LLMs in dental patient communication [25], the present study addresses a critical gap, as no prior investigation has systematically evaluated the accuracy, temporal consistency, and comprehensiveness of LLM-generated responses to structured, patient-focused periodontal questions derived from commonly reported misconceptions. Based on evaluations conducted by expert periodontists, this study further sought to investigate the potential of these systems as patient education tools for addressing and correcting misconceptions related to periodontal health.

The null hypothesis (H_0_) was that there would be no significant differences among the evaluated LLMs in terms of the accuracy, temporal consistency, and comprehensiveness of their responses to the patient-focused periodontal questions.

## 2. Materials and Methods

### 2.1. Study Design

This comparative content analysis was conducted at the Department of Periodontology, Zonguldak Bülent Ecevit University, between May and June 2026. As the study did not involve human participants or animals, ethical approval was not required.

### 2.2. Sample Size Calculation

The sample size was determined a priori using G*Power software (Version 3.1.9.7; Heinrich Heine University, Düsseldorf, Germany) for a repeated-measures analysis of variance (ANOVA). Consistent with the methodology adopted in previous comparative studies evaluating multiple LLMs, each question was considered an independent observational unit because all LLMs were assessed using the same standardized set of questions. The anticipated effect size was determined based on previously published studies employing comparable repeated-measures designs, and a medium effect size (f = 0.25) was assumed. With a significance level of α = 0.05, a statistical power of 0.90, one group, seven repeated measurements, an assumed average correlation of 0.50 among repeated measurements, and a nonsphericity correction factor (ε) of 1.0, the minimum required sample size was calculated to be 21 observational units (questions) (noncentrality parameter λ = 18.37; critical F = 2.17). To enhance statistical precision and ensure balanced representation across the predefined thematic categories, a total of 30 questions were ultimately included in the study.

### 2.3. Question Development

An empirical literature search was conducted to identify commonly reported misconceptions related to periodontal disease and oral hygiene practices and to develop patient-focused periodontal questions based on these misconceptions. As the literature search was conducted empirically for the purpose of question development rather than as a systematic review, study identification and selection were not managed within a PRISMA-based review framework. The search included PubMed (https://pubmed.ncbi.nlm.nih.gov/, accessed on 1 May 2026), medRxiv (https://www.medrxiv.org/), arXiv (https://arxiv.org/) and Google Scholar (https://scholar.google.com/, accessed on 1 May 2026) and covered studies published from the inception of each database through 7 May 2026. Search terms related to “myths,” “misconceptions,” and “misbeliefs” in the fields of dentistry and oral health were used to identify potentially relevant publications. Relevant studies were reviewed, and commonly reported misconceptions relevant to periodontal health and oral hygiene were extracted and subsequently used as the basis for developing patient-focused periodontal questions.

The inclusion criteria were as follows:

- Studies addressing periodontal disease and/or oral hygiene practices;
- Studies including the terms “myths,” “misconceptions-corrections,” “evidence-confirmation,” or “misbeliefs” in the title, abstract, or keywords;
- Studies evaluating patient perspectives, patient feedback, or patient-derived misconceptions;
- Full-text articles published in English.

The literature search identified studies reporting commonly encountered periodontal misconceptions, patient-derived myths, and frequently raised questions related to periodontal disease and oral hygiene practices [5,7,8,11,16,20,26,27,28,29,30,31,32,33]. These findings informed the development of patient-focused periodontal questions reflecting commonly reported patient concerns. An initial pool of 573 questions and statements was compiled through the empirical literature search. An initial relevance assessment was then performed to identify items directly related to the scope of the study. At this stage, 419 items that were not directly related to periodontal health, misconceptions, or myths were excluded. The remaining 154 items were retained for further detailed evaluation (Supplementary File S2).

To ensure the benchmark specifically targeted layperson misconceptions rather than specialized clinical workflows, a two-stage filtering and question-development process was applied. In the first stage, 78 items were excluded based on four predefined criteria: (A) Outside the predefined scope (including general clinical dentistry, financial costs, and non-periodontal procedures; *n* = 8), (B) Redundant or duplicate items (*n* = 26), (C) Specialty-specific topics addressing highly specialized clinical management and advanced surgical techniques of periodontics, including sub-specialty nuances of gingival recession (*n* = 42), and (D) Pediatric dentistry-related topics (*n* = 2). The remaining 76 items constituted the source pool for question development and were subsequently evaluated by two periodontists for thematic overlap, public relevance, and semantic clarity. Based on this evaluation, conceptually related and overlapping content was considered collectively and used to develop a standardized set of 30 patient-focused questions. A complete itemized record detailing the retention, exclusion stage, and specific reason for the 154 items, where applicable, is provided in Supplementary File S2.

To ensure consistency in content and format, items originally presented as declarative statements were reformulated into interrogative form, resulting in a standardized set of 30 patient-focused questions. The final question set was reviewed for linguistic clarity and consistency. Subsequently, the questions were categorized into three thematic domains representing common misconceptions related to periodontal disease and oral hygiene practices:

- Periodontal symptoms and risk factors (*n* = 10);
- Oral hygiene and daily habits (*n* = 10);
- Treatment process (*n* = 10).

The complete list of questions organized according to the thematic categories is presented in Table 1.

### 2.4. Development of the Reference Standard Answers

To establish an independent evaluation baseline, a reference answer set (Supplementary File S1) was authored by one investigator (İG) between 2 June and 7 June 2026, prior to LLM response generation. The answers were synthesized directly from the peer-reviewed literature and evidence-based clinical guidelines, with references provided for all factual and quantitative claims. No large language models or AI tools were used at any stage of drafting or editing this reference set, ensuring complete methodological independence from the evaluated systems.

### 2.5. Response Generation

Seven LLM-based AI systems commonly used for health information purposes were selected for evaluation: DeepSeek V4 (non-thinking default mode), Claude 4.6 Sonnet, GPT-5.5 (Instant configuration), Gemini 3.1 Flash, Grok 4.3, Microsoft Copilot Microsoft Copilot (web-based version, build/engine updated as of May 2026, Microsoft Corp., Redmond, WA, USA), and Monica AI. All systems were accessed through their official web interfaces using default standard configurations (default temperature and system prompts). Free-access (unpaid) subscription tiers were used consistently across all seven systems during both evaluation sessions. Microsoft Copilot was evaluated as a user-facing AI interface that routes queries to different underlying models rather than a single foundation model, whereas Monica AI was evaluated as an AI aggregation platform providing access to multiple underlying models. Question delivery was standardized to closely simulate how patients typically seek health information in real-world settings. All models were evaluated using the same predefined English-language questions presented in an identical order. No additional instructions, prompt refinement, pilot testing, or other interventions were applied.

All LLMs were accessed through their official web-based interfaces. Initial responses were generated between 9 June and 10 June 2026, and the complete response-generation procedure was repeated between 16 June and 17 June 2026, under identical study conditions. All questions were submitted in English by a single investigator, and the original responses were recorded without modification. Before each question–response session, the browser history and cookies were cleared, and a new chat session was initiated to minimize contextual carryover. No application programming interfaces (APIs), third-party software, conversation memory, custom instructions, web browsing, external tools, or optional reasoning modes were used during response generation. All models were evaluated under their default user settings. All prompts were submitted using the same laptop (Nitro AN16-41, 16 GB RAM; Acer Inc., New Taipei City, Taiwan) under identical technical conditions, including a 4.5G internet connection and a virtual private network (VPN; version 3.9, Astrill Systems Corp., Santa Clara, CA, USA). Because each model–question combination was evaluated using a single generated response per session, within-model run-to-run variability was not independently estimated in the present study.

All responses were exported to a Microsoft Word document (Microsoft Corporation, Redmond, WA, USA) (Supplementary File S4). Prior to calculating the readability indices, the raw responses were preprocessed by one investigator (İG) to remove presentation-related Markdown formatting cues, including bold and italic formatting, bullet symbols, and dashes, and to standardize sentence segmentation (Supplementary File S5). Substantive textual content, including headings and subheadings appearing within the responses, was retained. To minimize assessment bias, the responses were anonymized and blinded before evaluation by removing identifiers that could reveal the identity of the LLMs, including model-specific stylistic cues. The collected responses served as the primary dataset for all subsequent analyses. List items and headers lacking terminal punctuation were systematically appended with a period to ensure consistent sentence segmentation and to prevent artificial sentence-length variation during automated parsing.

### 2.6. Evaluation and Scoring Procedure

The responses generated by the LLMs were independently evaluated by two board-certified periodontists, each with more than five years of clinical experience, using a 5-point Likert scale [21,34] (Table 2). All evaluations were performed using anonymized and blinded responses to minimize assessment bias. Before the formal evaluation, the evaluators jointly reviewed representative question–response examples to calibrate the predefined scoring criteria. The responses provided by each LLM to all questions were then assessed independently by both evaluators based on their clinical expertise and current scientific evidence.

After completion of the independent evaluations, inter-evaluator agreement was assessed using exact agreement and agreement within one point. Exact agreement was defined as the proportion of responses receiving identical scores from both evaluators, whereas agreement within one point was defined as the proportion of responses with a score difference in no more than one point on the 5-point Likert scale. For all subsequent statistical analyses, the mean score of the two evaluators was used for each response. All responses generated by the LLMs were obtained by a single investigator (İG) and subsequently anonymized before evaluation. The anonymized responses were independently evaluated by two periodontists with expertise in the field (MKÇ and RÇ), neither of whom was involved in submitting the questions to the LLMs or generating/collecting the responses (Supplementary File S3).

### 2.7. Evaluation Criteria and Scoring Method

Each response was evaluated according to two predefined criteria:

***Accuracy***: Accuracy was defined strictly as the factual correctness and clinical validity of the provided information. This criterion evaluated whether the response was scientifically sound, free from medical inaccuracies or misleading claims, and aligned with evidence-based periodontal guidelines [21], independent of response length or depth of coverage.

***Comprehensiveness***: Comprehensiveness was defined strictly as the completeness and breadth of clinical coverage. This criterion evaluated whether the response addressed all necessary clinical dimensions, nuances, and underlying factors required for a holistic understanding of the topic [21], without penalizing concise statements that were otherwise factually accurate.

**Readability**: Readability refers to the ease with which a written text can be read and understood by its intended audience [35]. In the present study, readability was assessed using the Simple Measure of Gobbledygook (SMOG) [36], the Flesch Reading Ease (FRE) score [37] and the Flesch–Kincaid Grade Level (FKGL) [38]. Before readability analysis, model-specific stylistic and formatting cues were removed from the responses to minimize potential identification of the LLM source. Readability indices were then calculated using the modified text. For each response, readability was assessed by copying the modified text into The Readability Test Tool, a free online readability calculator (Simpson, D. The Readability Test Tool. http://www.readable.com (2013)). The formulas for SMOG, FRE, and FKGL are presented below; all three indices estimate readability based on the relationships among the numbers of words, sentences, and syllables within a text.

SMOG=3+√[no.of words with 3 or more syllables]×[30/no.of sentences]

FRE=206.835−(1.015×average no.of words per sentence)−

(84.6×average no.of syllables per word)

$$FKGL=0.39\left(\frac{words}{sentences}\right)+11.8\left(\frac{syllables}{words}\right)-15.59$$

The evaluation results were recorded in a Microsoft Excel spreadsheet (Microsoft Corporation, Redmond, WA, USA) for statistical analysis. Data for each question, including the accuracy and comprehensiveness ratings assigned by the evaluators, were systematically documented.

### 2.8. Statistical Analysis

Statistical analyses were performed using SPSS Statistics version 26.0 (IBM Corp., Armonk, NY, USA). Descriptive statistics are presented as mean ± standard deviation, median, and interquartile range (Q1–Q3). Data normality was assessed using the Shapiro–Wilk test. Statistical significance was set at *p* < 0.05. Agreement between the evaluators’ accuracy and comprehensiveness ratings was assessed descriptively using exact agreement and agreement within one point. Each response served as the unit of analysis, and the mean of the two evaluators’ scores was used for all subsequent statistical analyses [25].

Accuracy and comprehensiveness scores were compared using the Friedman test, followed by Bonferroni-adjusted Wilcoxon signed-rank tests for pairwise comparisons (adjusted significance threshold: *p* < 0.00238). Effect sizes were expressed as Kendall’s W. To examine the potential relationship and conceptual distinction between accuracy and comprehensiveness as separate evaluation dimensions, Spearman’s rank correlation analysis was performed using the mean scores of the two evaluators.

Readability outcomes, including the Flesch Reading Ease (FRE), Flesch–Kincaid Grade Level (FKGL), and Simple Measure of Gobbledygook (SMOG) scores, were analyzed using one-way repeated-measures analysis of variance (RM-ANOVA). The assumption of sphericity was evaluated using Mauchly’s test. When the assumption was violated, Greenhouse–Geisser-corrected results were reported. Significant overall effects were followed by Bonferroni-adjusted pairwise comparisons. Effect sizes were reported as partial eta squared (ηp^2^) and generalized eta squared (ηG^2^).

The relationships between response length (word count) and readability indices (FRE, FKGL, and SMOG) as well as evaluation outcomes (comprehensiveness) were assessed across each LLM using Pearson correlation analysis.

Temporal consistency was evaluated for all outcome measures by calculating change scores (Δ) as the difference between the follow-up and initial assessments (follow-up − initial). The resulting Δ values for accuracy, comprehensiveness, FRE, FKGL, and SMOG were compared among the evaluated LLMs using one-way repeated-measures ANOVA. Effect sizes were expressed as partial eta squared (ηp^2^) and generalized eta squared (ηG^2^).

To further assess temporal consistency at the individual-model level, within-model changes in accuracy and comprehensiveness were evaluated using paired-samples t-tests or Wilcoxon signed-rank tests, as appropriate, and Holm-adjusted *p* values were reported. Between-session agreement was additionally examined using Bland–Altman analysis, reporting mean differences and 95% limits of agreement (LoA).

## 3. Results

### 3.1. Agreement Between Evaluators

Across the 420 responses generated in the two evaluation sessions (210 responses per session), exact agreement between the two evaluators was 71.4% for accuracy and 70.2% for comprehensiveness, while agreement within one point was 96.9% and 95.7%, respectively. To account for chance agreement and the ordinal nature of the scoring scale, inter-evaluator agreement was additionally assessed using quadratically weighted Cohen’s kappa. The weighted kappa was 0.184 (95% bootstrap CI: 0.065–0.300) for accuracy and 0.169 (95% bootstrap CI: 0.068–0.271) for comprehensiveness. Thus, although the evaluators showed high observed agreement and scoring discrepancies were rarely greater than one point, chance-corrected agreement was limited. This discrepancy should be interpreted in the context of the marked concentration of scores at the upper end of the five-point scale, which restricted score variability.

### 3.2. Accuracy

A statistically significant difference in accuracy scores was observed among the evaluated LLMs (χ^2^ = 35.506, df = 6, *p* < 0.001), with a small effect size (Kendall’s W = 0.197). Mean accuracy scores were 4.46 ± 0.52 for Monica AI, 4.85 ± 0.29 for Copilot, 4.88 ± 0.25 for Grok 4.3, 4.83 ± 0.23 for Gemini 3.1, 4.55 ± 0.49 for GPT-5.5, 4.83 ± 0.35 for Claude 4.6 Sonnet, and 4.83 ± 0.30 for DeepSeek V4. Despite the statistically significant overall difference, the relatively small Kendall’s W value and the concentration of mean scores near the upper limit of the scale indicate that the observed differences represented relatively modest between-model variation rather than marked differences in overall accuracy.

Pairwise comparisons were interpreted using the Bonferroni-adjusted significance threshold (*p* < 0.00238). Monica AI demonstrated significantly lower accuracy scores than Copilot (*p* = 0.002), Grok 4.3 (*p* = 0.001), and Claude 4.6 Sonnet (*p* = 0.002). Furthermore, Grok 4.3 achieved significantly higher accuracy scores than GPT-5.5 (*p* = 0.002), and DeepSeek V4 achieved significantly higher accuracy scores than GPT-5.5 (*p* = 0.002). No statistically significant differences were observed between Monica AI and Gemini 3.1, Monica AI and GPT-5.5, Monica AI and DeepSeek V4, Copilot and Grok 4.3, Copilot and Gemini 3.1, Copilot and GPT-5.5, Copilot and Claude 4.6 Sonnet, Copilot and DeepSeek V4, Grok 4.3 and Gemini 3.1, Grok 4.3 and Claude 4.6 Sonnet, Grok 4.3 and DeepSeek V4, Gemini 3.1 and GPT-5.5, Gemini 3.1 and Claude 4.6 Sonnet, Gemini 3.1 and DeepSeek V4, or Claude 4.6 Sonnet and DeepSeek V4 following Bonferroni correction.

### 3.3. Comprehensiveness

A statistically significant difference in comprehensiveness scores was observed among the evaluated LLMs (χ^2^ = 46.241, df = 6, *p* < 0.001), with a small effect size (Kendall’s W = 0.257). Mean comprehensiveness scores were 4.41 ± 0.49 for Monica AI, 4.88 ± 0.25 for Copilot, 4.90 ± 0.24 for Grok 4.3, 4.70 ± 0.33 for Gemini 3.1, 4.43 ± 0.48 for GPT-5.5, 4.78 ± 0.38 for Claude 4.6 Sonnet, and 4.85 ± 0.29 for DeepSeek V4.

Pairwise comparisons were interpreted using the Bonferroni-adjusted significance threshold (*p* < 0.00238). Monica AI demonstrated significantly lower comprehensiveness scores than Copilot (*p* < 0.001), Grok 4.3 (*p* < 0.001), and DeepSeek V4 (*p* = 0.002). Furthermore, GPT-5.5 demonstrated significantly lower comprehensiveness scores than Copilot (*p* < 0.001), Grok 4.3 (*p* < 0.001), and DeepSeek V4 (*p* = 0.001). No statistically significant differences were observed among the remaining pairwise comparisons following Bonferroni correction.

As an additional descriptive and exploratory analysis, responses with a mean evaluator score of 3.5 or below (≤ 3.5) were examined across the 420 responses generated in the two evaluation sessions. This threshold was selected descriptively to identify and characterize responses representing the lower end of the observed score distribution, rather than as a predefined criterion for inadequate performance. For accuracy, 13 of the 420 responses (3.1%) met this criterion. These responses were limited to Monica AI (*n* = 8), GPT-5.5 (*n* = 4), and Claude 4.6 Sonnet (*n* = 1), with no lower-scoring responses identified for the other four LLMs. Lower-scoring responses most frequently concerned Question 5 (*n* = 4) and Question 7 (*n* = 3), followed by Questions 6 and 13 (*n* = 2 each) and Questions 22 and 25 (*n* = 1 each). For comprehensiveness, 12 responses (2.9%) met the criterion, occurring in Monica AI (*n* = 7), GPT-5.5 (*n* = 4), and Claude 4.6 Sonnet (*n* = 1). In terms of question distribution, Question 5 was the most frequent (*n* = 4), followed by Question 6 (*n* = 2); Questions 4, 7, 10, 22, 24, and 25 were each represented once. No responses from the remaining four LLMs met this criterion for either dimension.

Based on the mean scores of the two evaluators in the first evaluation session, accuracy and comprehensiveness showed a moderate positive correlation (Spearman’s ρ = 0.438, *p* < 0.001). This finding indicates that although the two evaluation dimensions were related, they were not strongly overlapping and captured distinguishable aspects of LLM response quality.

### 3.4. Category-Based Analysis

As each thematic category consisted of 10 questions, category-based analyses were performed in an exploratory nature, taking into account the limited subgroup sample sizes. Within this scope, non-parametric Friedman tests were applied to evaluate the distribution of LLMs’ accuracy and comprehensiveness performance across the three predefined thematic categories. For Symptoms and Risk Factors, no statistically significant differences among LLMs were observed for accuracy (x^2^ = 9.313, df = 6, *p* = 0.157). In contrast, a statistically significant difference among LLMs was observed for comprehensiveness (x^2^ = 13.047, df = 6, *p* = 0.042); however, following Bonferroni correction for pairwise post hoc comparisons (α_adj_ = 0.00238), no specific model pairs demonstrated statistically significant differences. Similarly, for Oral Care and Daily Habits, no statistically significant differences among LLMs were found for accuracy (x^2^ = 9.945, df = 6, *p* = 0.127), whereas a statistically significant difference was identified for comprehensiveness (x^2^ = 15.467, df = 6, *p* = 0.017). Nevertheless, no pairwise comparisons remained statistically significant after Bonferroni correction. In contrast, for Treatment Process, statistically significant differences among LLMs were identified for both accuracy (x^2^ = 30.490, df = 6, *p* < 0.001) and comprehensiveness (x^2^ = 27.074, df = 6, *p* < 0.001). However, following Bonferroni correction for pairwise post hoc comparisons (α_adj_ = 0.00238), no specific model pairs retained statistical significance for either parameter.

### 3.5. Flesch Reading Ease (FRE)

A statistically significant difference in FRE scores was observed among the evaluated LLMs (F(6,174) = 12.920, *p* < 0.001), with a large effect size (partial η_p_^2^ = 0.308, η_G_^2^ = 0.178). Because the assumption of sphericity was met (Mauchly’s test, *p* = 0.155), the sphericity-assumed results were reported. Mean FRE scores were 44.8 ± 10.7 for Monica AI, 40.0 ± 8.1 for Copilot, 39.5 ± 9.1 for Grok 4.3, 51.4 ± 11.5 for Gemini 3.1, 48.2 ± 7.7 for GPT-5.5, 44.4 ± 11.3 for Claude 4.6 Sonnet, and 52.1 ± 12.0 for DeepSeek V4. Bonferroni-adjusted pairwise comparisons showed that Monica AI had significantly lower FRE scores than DeepSeek V4 (*p* = 0.045). Copilot demonstrated significantly lower FRE scores than Gemini 3.1, GPT-5.5, and DeepSeek V4 (all *p* < 0.001). Similarly, Grok 4.3 showed significantly lower FRE scores than Gemini 3.1 and GPT-5.5 (both *p* < 0.001) and DeepSeek V4 (*p* = 0.001). In contrast, no significant differences were observed between Monica AI and Copilot, Monica AI and Grok 4.3, Monica AI and Gemini 3.1, Monica AI and GPT-5.5, Monica AI and Claude 4.6 Sonnet, Copilot and Grok 4.3, Copilot and Claude 4.6 Sonnet, Grok 4.3 and Claude 4.6 Sonnet, Gemini 3.1 and GPT-5.5, Gemini 3.1 and DeepSeek V4, GPT-5.5 and Claude 4.6 Sonnet, GPT-5.5 and DeepSeek V4, or Claude 4.6 Sonnet and DeepSeek V4. Furthermore, Gemini 3.1 demonstrated significantly higher FRE scores than Claude 4.6 Sonnet (*p* = 0.027), while DeepSeek V4 demonstrated significantly higher FRE scores than Claude 4.6 Sonnet (*p* = 0.013).

### 3.6. Flesch–Kincaid Grade Level (FKGL)

A statistically significant difference in FKGL scores was observed among the evaluated LLMs (F(3.764, 109.142) = 5.598, *p* < 0.001), with a large effect size (partial η_p_^2^ = 0.162, η_G_^2^ = 0.104). Because the assumption of sphericity was violated (Mauchly’s test, *p* = 0.011), the Greenhouse–Geisser correction was applied. Mean FKGL scores were 9.6 ± 1.6 for Monica AI, 10.2 ± 1.2 for Copilot, 10.8 ± 1.3 for Grok 4.3, 9.8 ± 1.7 for Gemini 3.1, 9.2 ± 1.4 for GPT-5.5, 10.2 ± 1.7 for Claude 4.6 Sonnet, and 9.1 ± 2.2 for DeepSeek V4. Bonferroni-adjusted pairwise comparisons showed that Monica AI demonstrated significantly lower FKGL scores than Grok 4.3 (*p* = 0.020). In addition, GPT-5.5 demonstrated significantly lower FKGL scores than Copilot (*p* = 0.027), Grok 4.3 (*p* < 0.001), and Claude 4.6 Sonnet (*p* = 0.017). No statistically significant differences were observed among the remaining pairwise comparisons.

### 3.7. Simple Measure of Gobbledygook (SMOG)

A statistically significant difference in SMOG scores was observed among the evaluated LLMs (F(4.205, 121.946) = 8.057, *p* < 0.001), with a large effect size (partial η_p_^2^ = 0.217, η_G_^2^ = 0.140). Because the assumption of sphericity was violated (Mauchly’s test, *p* = 0.003), the Greenhouse–Geisser correction was applied. Mean SMOG scores were 13.4 ± 1.3 for Monica AI, 12.4 ± 1.2 for Copilot, 13.4 ± 1.2 for Grok 4.3, 13.1 ± 1.5 for Gemini 3.1, 13.2 ± 2.1 for GPT-5.5, 14.0 ± 2.2 for Claude 4.6 Sonnet, and 11.8 ± 1.6 for DeepSeek V4. Bonferroni-adjusted pairwise comparisons showed that DeepSeek V4 achieved significantly lower SMOG scores than Monica AI (*p* = 0.001), Grok 4.3 (*p* = 0.001), Gemini 3.1 (*p* = 0.011), GPT-5.5 (*p* = 0.011), and Claude 4.6 Sonnet (*p* < 0.001). In addition, Copilot demonstrated significantly lower SMOG scores than Monica AI (*p* = 0.025) and Claude 4.6 Sonnet (*p* = 0.007). Furthermore, Grok 4.3 demonstrated significantly higher SMOG scores than Copilot (*p* < 0.001). No statistically significant differences were observed among the remaining pairwise comparisons after Bonferroni correction. Complete descriptive statistics, including means, standard deviations, medians, interquartile ranges, and significant post hoc comparisons, are presented in Table 3.

Figure 1 presents the comparative performance of the evaluated LLMs with respect to accuracy (AC), comprehensiveness (CO), and readability metrics.

### 3.8. Response Length and Correlation Analyses

The structural metrics (sentence count and word count) along with the Pearson correlation coefficients analyzing the relationship between word count, readability indices, and comprehensiveness scores across the evaluated LLMs are presented in Table 4.

Response length varied across the evaluated LLMs. Mean sentence counts ranged from 18.70 ± 5.01 for Monica AI to 39.30 ± 26.48 for DeepSeek V4, while mean word counts ranged from 186.43 ± 21.60 to 431.30 ± 253.02, respectively. The widest ranges in both sentence count (5–89) and word count (71–810) were observed for DeepSeek V4.

Correlation analyses showed that word count was significantly correlated with comprehensiveness for GPT-5.5 (r = 0.584, *p* < 0.001), Claude 4.6 Sonnet (r = 0.502, *p* = 0.005), and DeepSeek V4 (r = 0.502, *p* = 0.005). Regarding readability, word count was significantly correlated with SMOG for Grok 4.3 (r = 0.568, *p* = 0.001), with FRE for Grok 4.3 (r = −0.470, *p* = 0.009) and DeepSeek V4 (r = 0.501, *p* = 0.005), and with FKGL for Grok 4.3 (r = 0.469, *p* = 0.009) and DeepSeek V4 (r = −0.638, *p* < 0.001). No significant correlations between word count and comprehensiveness or readability measures were observed for the remaining LLMs.

For temporal consistency, no statistically significant differences in change scores were observed among the evaluated LLMs for any of the assessed outcomes. Accuracy change scores did not differ significantly across the LLMs (*p* = 0.173, partial η_p_^2^ = 0.053, ηG2 = 0.048), nor did comprehensiveness change scores (*p* = 0.107, partial η_p_^2^ = 0.062, ηG2 = 0.056). Likewise, no significant differences were observed for FRE (*p* = 0.924, partial η_p_^2^ = 0.008, ηG^2^ = 0.005) or FKGL (*p* = 0.862, partial η_p_^2^ = 0.010, ηG^2^ = 0.008). In contrast, a statistically significant difference was observed for SMOG (*p* = 0.004, partial η_p_^2^ = 0.126, ηG^2^ = 0.108). These findings indicate that the magnitude of change between the initial and follow-up assessments was comparable across all evaluated LLMs for accuracy, comprehensiveness, FRE, and FKGL, whereas a significant difference in change scores was observed for SMOG. Effect sizes were small for accuracy, comprehensiveness, FRE, and FKGL, reflecting minimal temporal variation among models, whereas a medium effect size was observed for SMOG.

The comparative results of the temporal consistency analysis are presented in Table 5, whereas the overall magnitude and direction of changes between the initial and follow-up assessments are illustrated in Figure 2.

Within-model analyses showed no significant between-session changes in accuracy after Holm adjustment across the seven LLMs (all adjusted *p* > 0.05). For comprehensiveness, a significant increase was observed only for Gemini 3.1 (mean difference: 0.23; 95% CI: 0.11–0.36; Holm-adjusted *p* = 0.013), whereas no significant changes were detected for the remaining models. Bland–Altman analysis showed generally small mean between-session differences, with the 95% limits of agreement reflecting varying degrees of question-level variability across models (Table 6).

## 4. Discussion

The increasing role of digital technologies in health communication has enabled individuals to obtain health information not only from healthcare professionals and traditional printed sources but also from diverse resources, including online platforms and artificial intelligence-based digital tools. A recent study demonstrated that LLM-based chatbots are emerging as a new source of health information [39]. Despite this expanding information ecosystem, health-related misconceptions continue to persist within communities [40]. A study conducted in the United States reported that more than 94% of participants held at least one medical misconception, and approximately half of the evaluated misconceptions were perceived as being true by the respondents [41]. Similarly, misconceptions related to oral health have also been reported to be widespread [42,43].

In this context, considering the fundamental role of individual oral health behaviors and treatment adherence in the prevention and management of periodontal disease, misconceptions related to periodontal health warrant particular attention. These misconceptions are not merely passive reflections of insufficient knowledge; rather, they may influence oral health behaviors, contribute to delays in seeking professional care, lead to the neglect of symptoms, and result in avoidance of necessary treatments [5,44,45,46,47,48,49]. The increasing use of LLM-based chatbots as sources of health information further underscores the clinical importance of evaluating the accuracy, temporal consistency, and comprehensiveness of their responses to common patient-focused questions related to periodontal disease and oral hygiene practices. However, to the best of our knowledge, LLM responses to patient-focused periodontal questions informed by commonly reported misconceptions and patient concerns have not previously been systematically investigated.

In the present study, the responses generated by LLMs to common patient-focused questions related to periodontal disease and oral hygiene practices were evaluated in terms of accuracy, comprehensiveness, readability, and temporal consistency. Overall, the evaluated models demonstrated good performance in addressing these questions, although differences were observed across the evaluated dimensions. Although statistically significant differences were identified among the models regarding accuracy, comprehensiveness, and readability, all models achieved an overall high level of accuracy. Grok 4.3, Copilot, and DeepSeek V4 achieved higher scores in terms of accuracy and comprehensiveness, whereas DeepSeek V4 and Gemini 3.1 showed relatively superior performance in readability metrics. No statistically significant between-model differences in change scores were observed for accuracy, comprehensiveness, FRE, or FKGL, whereas a significant between-model difference was observed for SMOG.

Previous studies in dentistry and medicine have reported that LLMs can generally achieve high levels of accuracy and comprehensiveness, although variations in performance exist among different models [48,50,51]. Previous studies have demonstrated that differences in performance exist among LLMs in terms of accuracy and comprehensiveness when evaluating knowledge in dentistry and medicine [52,53,54,55]. In a study by Lin et al. assessing the accuracy and comprehensiveness of LLM-generated responses to examination questions, ChatGPT-3.5, Gemini, and Claude-2 were compared, with Claude-2 achieving the highest performance (54.8%) among the evaluated models [52]. Similarly, another study identified ChatGPT-4o as the best-performing model, achieving an accuracy of 86.1%, whereas Bard, ChatGPT-3.5, and Copilot demonstrated lower levels of accuracy [53]. Furthermore, international evaluations involving the United States, Hong Kong, and China medical licensing examinations also demonstrated that GPT-4o outperformed other LLMs [54]. In addition, Chatzopoulos et al. evaluated LLM-generated responses to clinical questions regarding periodontal furcation defects and reported that Google Gemini Advanced achieved the highest comprehensiveness, whereas Microsoft Copilot demonstrated comparatively lower comprehensiveness scores [55].

In the present study, Grok 4.3, Copilot, and DeepSeek V4 were among the highest-performing models regarding accuracy and comprehensiveness. The relative differences in performance between the current findings and the previous literature may be attributed to methodological variations, including the question sets used, evaluation approaches, prompt designs, and the continuously evolving nature of LLMs. Indeed, while most previous studies have focused on examination-based questions or assessments of clinical knowledge, the present study employed common periodontal misconceptions encountered within the general population. This approach required the models not only to provide accurate information but also to identify incorrect statements, correct them based on scientific evidence, and explain the corrections in an understandable manner for users. Therefore, the question set used in this study may have evaluated not only factual knowledge retrieval but also the ability to critically assess misinformation and correct it according to evidence-based principles. Although statistically significant differences were observed among the models in terms of accuracy and comprehensiveness, the effect sizes were small. This finding suggests that all evaluated models performed generally well in addressing periodontal misconceptions and that the observed differences primarily represent relative variations in model performance rather than substantial clinical inadequacies. Importantly, the exploratory analysis of lower-scoring responses showed that, despite the generally high overall scores, a small proportion of responses received mean scores below 3.5 (3.1% for accuracy and 2.9% for comprehensiveness). These responses were concentrated in a limited number of LLMs and included clinically relevant questions concerning periodontal treatment, tooth loss, bleeding, and dental scaling. Although this analysis was descriptive, it highlights that occasional potentially misleading or incomplete responses may occur despite high overall performance and underscores the importance of clinical verification of AI-generated periodontal information. A qualitative re-examination of these lower-scoring responses suggests that certain sub-threshold scores were driven by inter-rater scoring variance rather than fundamental model error. For instance, in response to Q5 (Can gum disease get better without treatment?), GPT-5.5 and Monica AI provided answers that were substantively concordant with the reference standard, yet received scores of 3.5 or below. This indicates that a portion of the lower scores reflects instrument noise and evaluator variability rather than intrinsic clinical inaccuracies within the outputs, suggesting that claims of model failure in these specific instances should be interpreted cautiously. Based on these findings, the null hypothesis (H_0_) of the study was rejected.

Readability is a critical component of patient education in periodontal disease, as it may influence treatment adherence and clinical decision-making processes [20]. Previous studies have reported variations in readability performance among different LLMs, with Gemini Advanced, Copilot, Bing Chat, and GPT-4 demonstrating superior readability in different investigations [55,56,57,58,59]. In the present study, DeepSeek V4, Gemini 3.1, and, to a lesser extent, GPT-5.5 generated responses with higher readability scores. Crucially, however, despite these relative differences among systems, absolute metrics across all evaluated models revealed a high level of structural and linguistic complexity that exceeds layperson health literacy requirements. Mean FKGL scores across models indicate that the generated content consistently required a high-school reading comprehension level, while mean FRE scores fell into the “difficult” to “fairly difficult” reading categories. Readability performance may depend not only on the model itself but also on factors such as the subject matter, prompt design, and evaluation methodology. However, because more comprehensive responses may often be associated with increased linguistic complexity, achieving an appropriate balance between accuracy, comprehensiveness, and readability is essential. Improving readability should not compromise clinical accuracy or comprehensiveness, particularly when communicating complex periodontal concepts. Excessive simplification may omit clinically relevant details or reduce scientific nuance. This finding highlights the importance of maintaining an optimal balance between scientific accuracy and comprehensibility, particularly for individuals with limited health literacy. Furthermore, no significant between-model differences in change scores were observed for accuracy, comprehensiveness, FRE, or FKGL, whereas a significant difference was observed for SMOG. These findings suggest broadly consistent short-term performance across the evaluated models for accuracy, comprehensiveness, FRE, and FKGL, whereas SMOG scores showed differential changes between the two assessment time points.

Extending beyond patient education, the high accuracy and comprehensiveness demonstrated by the evaluated LLMs may have potential implications for patient communication and health literacy. As patients increasingly consult AI-based systems before seeking professional care, the quality of the information provided may influence how they perceive symptoms and communicate their medical history during clinical encounters [60,61]. Alanezi et al. reported that ChatGPT supported health literacy among patients with cancer through emotional, informational, and motivational assistance [62]. By providing accessible educational materials and facilitating communication between patients and healthcare providers, LLMs may enhance patient education and engagement [63]. However, the present study did not evaluate their effects on clinical decision-making, patient history-taking, or diagnostic efficiency. These potential implications should therefore be considered hypotheses for future research rather than outcomes demonstrated by the present study.

An important limitation that may affect the accuracy of LLM-generated responses is the tendency toward sycophancy, which has been increasingly recognized in recent years [64]. This behavior may cause models to align with user expectations, particularly when confronted with incorrect assumptions or leading statements, resulting in responses that inadequately challenge claims inconsistent with scientific evidence. Indeed, Fanous et al. reported that this behavior was observed in 58.19% of evaluated cases [65]. Therefore, the potential emergence of such tendencies across different usage scenarios should be considered, and AI-generated responses should be interpreted with caution. Furthermore, responses generated by LLMs may not always fully reflect individual clinical characteristics or diverse clinical scenarios. In addition, limitations in their direct access to up-to-date scientific resources may influence the accuracy and reliability of their outputs [21,66]. The present study provides preliminary evidence regarding the potential application of artificial intelligence in correcting misconceptions related to periodontal disease and oral hygiene practices. However, these findings should not be interpreted as indicating that AI-based systems will demonstrate equally reliable performance across all clinical contexts. Therefore, verification of AI-generated responses against reliable scientific sources and professional healthcare expertise remains essential. Accordingly, LLMs should not be considered substitutes for clinical decision-making processes but rather as supportive information resources to be used under the supervision of healthcare professionals.

The major strengths of this study include the comparative evaluation of seven contemporary LLMs within a standardized methodological framework. Furthermore, the assessment of LLM performance based on validated readability indices, together with the evaluation of accuracy and comprehensiveness, represents a distinctive aspect of the present study. Although observed agreement between the evaluators was high and discrepancies greater than one point were uncommon, chance-corrected agreement was limited. The use of the mean of the two independent ratings was therefore adopted to reduce the influence of individual evaluator scoring differences. Importantly, from a clinical safety perspective, across all individual ratings, neither examiner assigned a score below 3 for accuracy or comprehensiveness. This demonstrates that no LLM response was judged as predominantly incorrect or clinically hazardous—even when addressing high-risk queries such as gingival bleeding, antibiotic indications, and surgical necessity. The included questions were developed through a comprehensive literature review and consisted of focused items specifically addressing periodontal misconceptions. To ensure a controlled comparison among the models, a single standardized prompt was used for each question, and follow-up interactions were avoided. This approach may have minimized potential sources of bias during the evaluation process and contributed to a more objective assessment of model performance.

Several limitations should be acknowledged. Presenting questions in English enabled standardized comparison but may not fully capture cultural and linguistic variation in periodontal misconceptions. The evaluated LLMs were not specifically developed for oral and dental healthcare applications, and model performance was not separately examined for confirmation versus refutation tasks. The question set was derived from an empirical and pragmatic literature search rather than a systematic or PRISMA-based review; therefore, the identified studies and resulting questions may not represent the full range of the relevant literature or periodontal misconceptions. Moreover, the question set was developed specifically for this comparative evaluation and was not intended or validated as a comprehensive measurement instrument.

The study period coincided with platform-level changes, including the beta development of Grok 4.3, the ChatGPT personalization rollout on 9 June 2026, and retirement of the intermediate GPT-5.2 model family before Session 2 (16 June 2026). Because only one response was generated for each model–question combination per session, run-to-run variability could not be separated from temporal effects. Given the inherent stochasticity of LLM outputs, small between-model and between-session differences should therefore be interpreted cautiously.

Evaluations were conducted by only two periodontists from a single institution, limiting generalizability. The low weighted kappa values, together with the marked ceiling effect, also indicate limited discrimination among predominantly high-scoring responses. Future studies should consider broader evaluator panels, repeated model runs, and more sensitive assessment instruments.

Finally, although FRE, FKGL, and SMOG were used to assess different aspects of readability, five of the seven LLMs had mean sentence counts below the recommended 30-sentence minimum for SMOG. SMOG findings should therefore be interpreted with caution.

Future research should consider evaluating patient-generated questions in different languages, incorporating larger and more diverse question sets, exploring alternative prompt strategies, and developing LLMs specifically tailored to domain-specific expertise. Such investigations may provide stronger evidence regarding the effectiveness of AI-assisted systems in supporting patient education and improving oral health literacy. Furthermore, prospective evaluation of continuously evolving AI technologies following their integration into real clinical settings with patients will enable a more comprehensive understanding of their contributions to patient comprehension, clinical decision-making processes, and treatment management under real-world conditions.

## 5. Conclusions

This study demonstrates that LLMs have considerable potential to provide accurate and comprehensive information in response to common patient-focused questions related to periodontal disease and oral hygiene practices. Among the evaluated models, Grok 4.3, Copilot, and DeepSeek V4 demonstrated superior performance in terms of accuracy and comprehensiveness, while DeepSeek V4 and Gemini 3.1 produced more readable content. However, none of the evaluated systems consistently achieved a reading level appropriate for general patient-oriented health communication, and no single model consistently outperformed the others across all evaluation criteria. Furthermore, improvements are needed to enhance the readability of LLM-generated responses for healthcare users without compromising scientific accuracy and comprehensiveness. Overall, although LLMs hold significant promise for supporting patient education and engagement, clinical examination and professional evaluation remain fundamental for the accurate diagnosis and appropriate treatment planning of periodontal diseases.

## Figures and Tables

**Figure 1:**
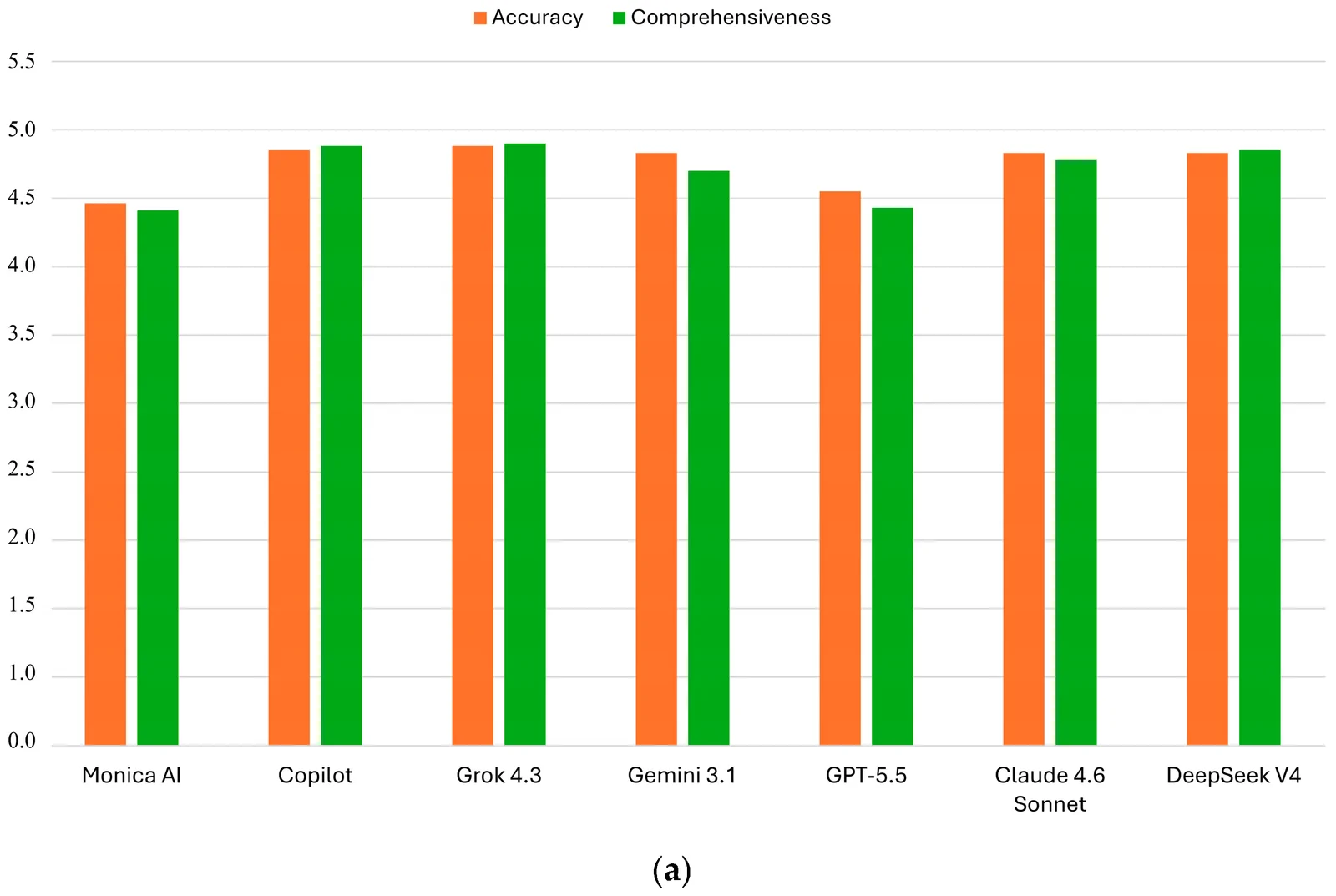

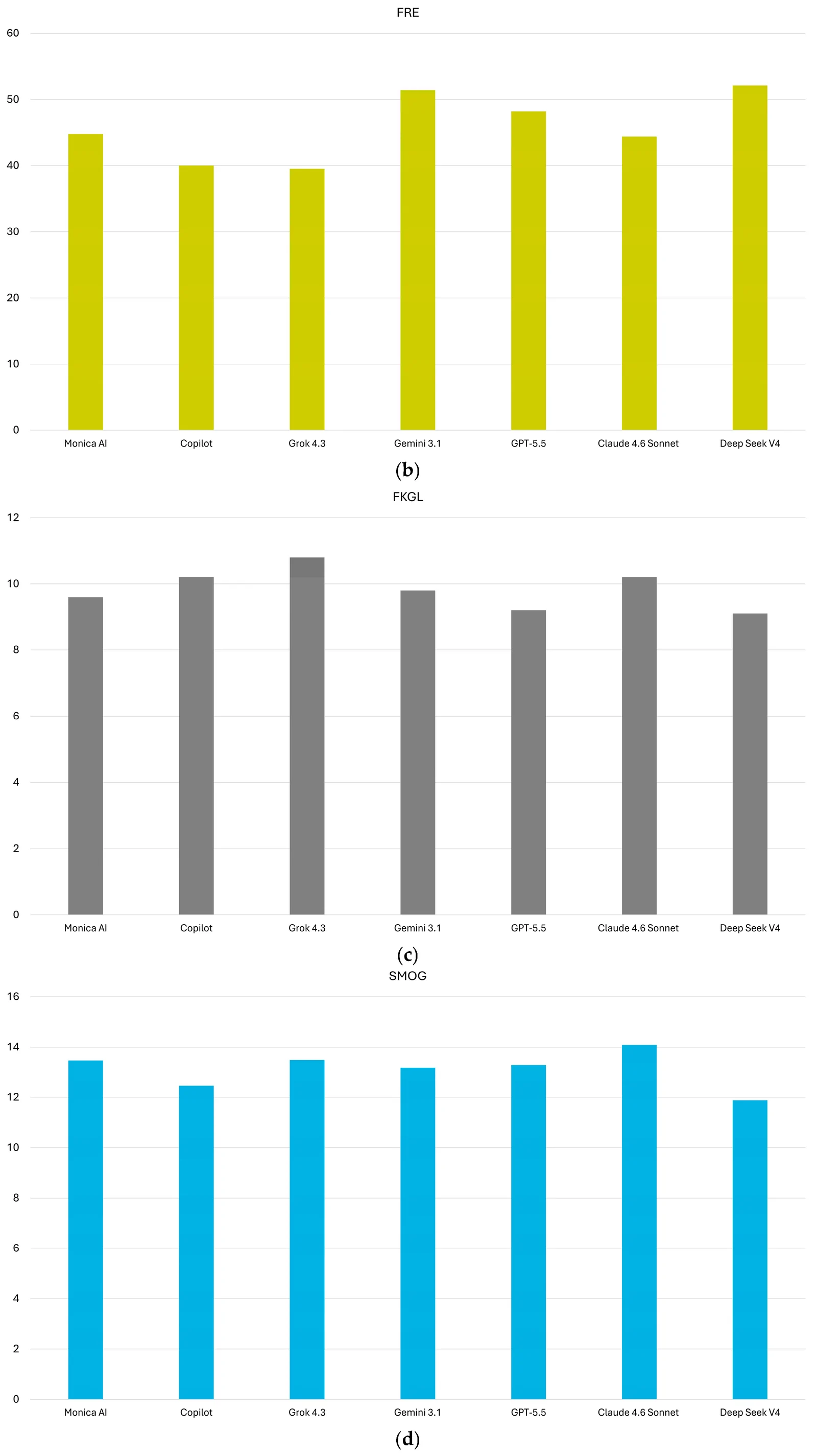
Comparative performance of the evaluated LLMs AC, CO and readability metrics. Orange and green bars in the upper panel represent the mean accuracy and comprehensiveness scores (**a**), respectively. The lower panels present the mean FRE (**b**), FKGL (**c**), and SMOG (**d**) scores, shown by yellow, gray, and blue bars, respectively. Higher FRE scores indicate greater readability, whereas higher FKGL and SMOG scores indicate greater reading difficulty.

**Figure 2:**
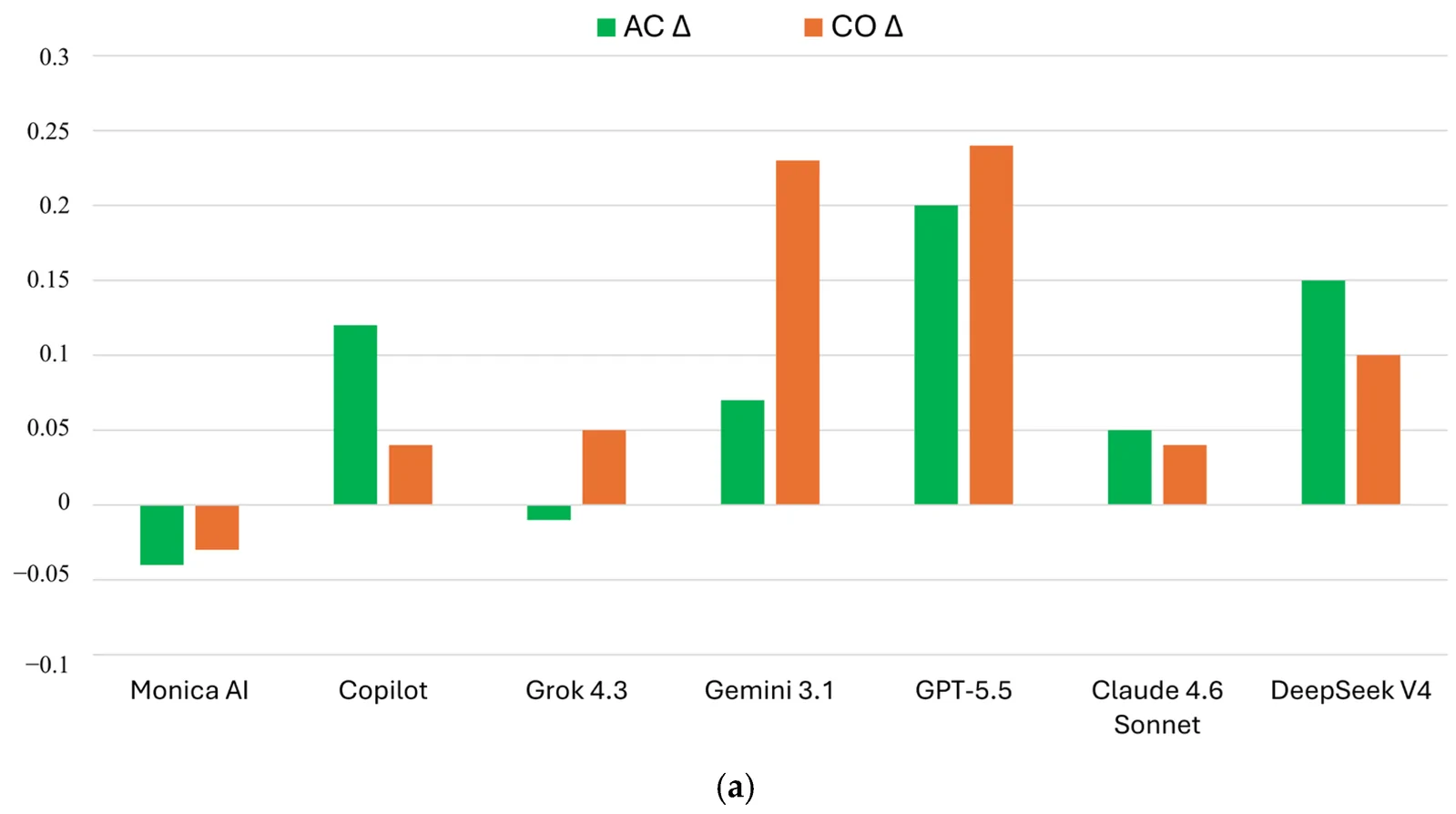

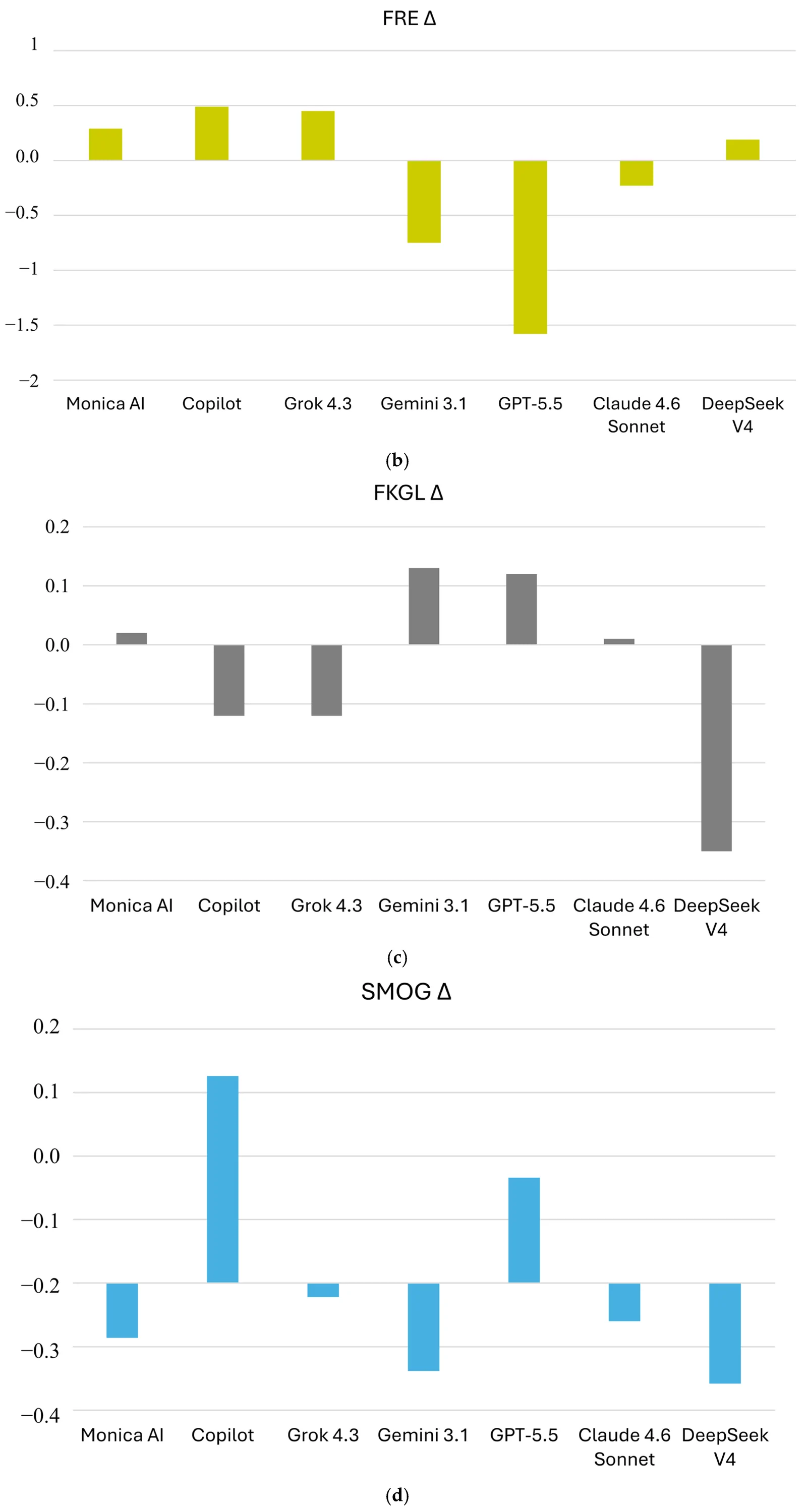
Comparison of differences (Δ) in accuracy, comprehensiveness, and readability metrics among the evaluated large language models. Comparison of differences (Δ) in accuracy, comprehensiveness, and readability metrics among the evaluated large language models. (**a**) Mean changes in Accuracy (Δ AC, green) and Comprehensiveness (Δ CO, orange). (**b**) Mean changes in Flesch Reading Ease (Δ FRE, yellow). (**c**) Mean changes in Flesch–Kincaid Grade Level (Δ FKGL, gray). (**d**) Mean changes in Simple Measure of Gobbledygook (Δ SMOG, blue). Note: Bars represent the mean changes (Δ) for each metric across the evaluated models.

**Table 1:** Literature-Based Thematic Question Set Derived from Misconceptions Regarding Periodontal Diseases.

Category	No	Question
**Symptoms and Risk Factors**	Q1	Do gum diseases only affect elderly people?
	Q2	Is it normal if my gums bleed when I brush my teeth?
	Q3	Can I get gum disease even if I brush my teeth regularly?
	Q4	Can I have gum disease even if my gums don’t hurt?
	Q5	Can gum disease get better without treatment?
	Q6	Are gum diseases rare?
	Q7	Is it normal to lose teeth because of gum disease as you get older?
	Q8	If gum disease runs in my family, will I definitely get it too?
	Q9	Can stress make me more likely to get gum disease?
	Q10	Do gum diseases only affect the mouth and teeth?
**Oral Care and Daily Habits**	Q11	Will a hard-bristled toothbrush clean my teeth better?
	Q12	Should I stop brushing my teeth for a few days if my gums are bleeding?
	Q13	Do I need to brush my teeth after every meal to keep my gums healthy?
	Q14	Even if I brush my teeth regularly, do I still need to get my gums checked?
	Q15	Is brushing my teeth once a day enough to keep my mouth healthy?
	Q16	Can flossing cause gum recession?
	Q17	Do electric toothbrushes damage the gums more than regular toothbrushes?
	Q18	Would using mouthwash alone be enough without brushing my teeth?
	Q19	After I quit smoking, my gums started bleeding. Is that normal?
	Q20	Can natural products effectively keep my gums healthy?
**Treatment Process**	Q21	Can getting a dental scaling damage my teeth?
	Q22	Can dental scaling cause gaps between my teeth?
	Q23	Do all gum diseases require the same treatment?
	Q24	Is gum treatment painful?
	Q25	Can gum surgery cause tooth loss?
	Q26	Can gum treatment make my teeth feel loose?
	Q27	Does tartar removal cause more tartar buildup?
	Q28	Are antibiotics enough to treat gum disease?
	Q29	After gum disease treatment, will I ever need treatment again?
	Q30	Is surgery always necessary for gum treatment?

**Table 2:** Scoring rubric for the independent assessment of accuracy and comprehensiveness based on a 5-point Likert scale.

	Accuracy		Comprehensiveness	
Score	Category	Definition	Category	Definition
1	Completely incorrect	Inconsistent with current scientific evidence or clinically misleading	Not adequate	Addresses only a minor part of the question; highly inadequate explanation
2	More incorrect than correct	Includes some correct elements but contains major factual inaccuracies	Somewhat adequate	Covers the question partially; omits key points
3	Partially correct/partially incorrect	Generally consistent with evidence but contains a minor factual inaccuracy	Adequate	Provides a general answer to the question; lacks depth in some areas
4	More correct than incorrect	Factually accurate and consistent with evidence, but contains minor terminological or phrasing imprecisions	Very adequate	Addresses most aspects of the question with sufficient and detailed explanation
5	Completely correct	Fully consistent with current evidence and clinical guidelines without any errors	Extremely adequate	Thoroughly and clearly covers all relevant aspects of the question in a balanced and informative manner

**Table 3:** Comparison of Accuracy, Comprehensiveness, and Readability Metrics Across LLMs.

Criteria		LLMs							*p*	c
		Monica AI ^a^	Copilot ^b^	Grok 4.3 ^c^	Gemini 3.1 ^d^	GPT-5.5 ^e^	Claude 4.6 Sonnet ^f^	DeepSeek V4 ^g^		
**AC**	Mean ± SD	4.46 ± 0.52 ^b,c,f^	4.85 ± 0.29	4.88 ± 0.25 ^e^	4.83 ± 0.23	4.55 ± 0.49 ^g^	4.83 ± 0.35	4.83 ± 0.30	**<0.001**	0.197 ^w^
	Median	4.5	5	5	5	4.5	5	5		
	(Q1–Q3)	[4.00–5.00]	[5.00–5.00]	[5.00–5.00]	[4.50–5.00]	[4.50–5.00]	[5.00–5.00]	[4.50–5.00]		
**CO**	Mean ± SD	4.41 ± 0.49 ^b,c,g^	4.88 ± 0.25 ^e^	4.90 ± 0.24 ^e^	4.70 ± 0.33	4.43 ± 0.48 ^g^	4.78 ± 0.38	4.85 ± 0.29	**<0.001**	0.257 ^w^
	Median	4.5	5	5	4.75	4.5	5	5		
	(Q1–Q3)	[4.00–5.00]	[5.00–5.00]	[5.00–5.00]	[4.50–5.00]	[4.00–5.00]	[4.50–5.00]	[5.00–5.00]		
**FRE**	Mean ± SD	44.8 ± 10.7 ^g^	40.0 ± 8.1 ^d,e,g^	39.5 ± 9.1 ^d,e,g^	51.4 ± 11.5 ^f^	48.2 ± 7.7	44.4 ± 11.3 ^g^	52.1 ± 12.0	**<0.001**	η_p_^2^ = 0.308/η_G_^2^ = 0.178
	Median	44.9	43.1	40.8	55.2	48.6	48.3	52.6		
	(Q1–Q3)	[38.0–52.1]	[35.4–44.6]	[33.5–49.4]	[46.9–57.3]	[42.8–53.4]	[40.0–50.4]	[45.9–60.2]		
**FKGL**	Mean ± SD	9.6 ± 1.6 ^c^	10.2 ± 1.2 ^e^	10.8 ± 1.3 ^e^	9.8 ± 1.7	9.2 ± 1.4 ^f^	10.2 ± 1.7	9.1 ± 2.2	**<0.001**	η_p_^2^ = 0.162/η_G_^2^ = 0.104
	Median	9.6	10.1	10.9	9.5	8.9	9.9	8.7		
	(Q1–Q3)	[8.3–10.8]	[9.5–11.0]	[9.7–11.8]	[8.8–10.7]	[8.2–10.2]	[9.3–11.2]	[8.0–9.8]		
**SMOG**	Mean ± SD	13.4 ± 1.3 ^b,g^	12.4 ± 1.2 ^c,f^	13.4 ± 1.2 ^g^	13.1 ± 1.5 ^g^	13.2 ± 2.1 ^g^	14 ± 2.2 ^g^	11.8 ± 1.6	**<0.001**	η_p_^2^ = 0.217/η_G_^2^ = 0.140
	Median	13.3	12.3	13.5	13	13.1	13.4	11.5		
	(Q1–Q3)	[12.6–14.4]	[11.5–13.3]	[12.7–14.5]	[12.1–14.2]	[12–13.7]	[12.6–15.5]	[10.7–13]		

LLM, Large Language Model; SD, standard deviation; FRE, Flesch Reading Ease; FKGL, Flesch–Kincaid Grade Level; SMOG, Simple Measure of Gobbledygook; *p*, significance value. Effect sizes are presented as Kendall’s W for Friedman test analyses and as partial eta squared (ηp^2^) and generalized eta squared (ηG^2^) for repeated-measures ANOVA. Bold values indicate statistically significant differences (*p* < 0.05). Superscript letters indicate statistically significant pairwise differences between LLMs following post hoc comparisons, ^a^, significantly different from Monica AI; ^b^, significantly different from Copilot; ^c^, significantly different from Grok 4.3; ^d^, significantly different from Gemini 3.1; ^e^, significantly different from GPT-5.5; ^f^, significantly different from Claude 4.6 Sonnet; ^g^, significantly different from DeepSeek V4.

**Table 4:** Response length, sentence count, and correlations of word count with comprehensiveness and readability measures across the evaluated LLMs.

Variables		LLMs													
		Monica AI		Copilot		Grok 4.3		Gemini 3.1		GPT-5.5		Sonnet	Claude 4.6	DeepSeek V4	
**(Min.–Max.)**	**Sentence count, mean ± SD**	(12–32)	18.70 ± 5.01	(9–55)	36 ± 9	(16–39)	28.53 ± 5.09	(17–37)	26.10 ± 5.08	(13–35)	20.56 ± 5.34	(15–31)	21.90 ± 4.15	(5–89)	39.30 ± 26.48
**(Min.–Max.)**	**Word count, mean ± SD**	(140–233)	186.43 ± 21.60	(168–406)	299.30 ± 74.14	(215–484)	354.60 ± 53.82	(292–558)	380.60 ± 59.05	(159–278)	215.80 ± 36.16	(195–358)	270.90 ± 37.84	(71–810)	431.30 ± 253.02
**count r (*p*)**	**CO–word**	−0.018 (0.927)		−0.280 (0.135)		−0.146 (0.441)		−0.325 (0.080)		0.584 ** (<0.001)		0.502 ** (0.005)		0.502 ** (0.005)	
**count r (*p*)**	**SMOG–word**	0.112 (0.555)		0.156 (0.409)		0.568 ** (0.001)		0.033 (0.864)		0.130 (0.493)		0.050 (0.793)		−0.326 (0.079)	
**count r (*p*)**	**FRE–word**	−0.219 (0.246)		−0.071 (0.708)		−0.470 ** (0.009)		0.064 (0.737)		−0.053 (0.781)		−0.233 (0.215)		0.501 ** (0.005)	
**count r (*p*)**	**FKGL–word**	0.140 (0.461)		−0.026 (0.890)		0.469 ** (0.009)		−0.035 (0.853)		0.159 (0.402)		0.313 (0.092)		−0.638 ** (<0.001)	

Values are presented as mean ± standard deviation (SD) or range, as appropriate. Pearson correlation coefficients (r) are reported for the associations between word count and comprehensiveness (CO), SMOG, FRE, and FKGL scores. CO, comprehensiveness; SMOG, Simple Measure of Gobbledygook; FRE, Flesch Reading Ease; FKGL, Flesch–Kincaid Grade Level. * *p* < 0.05; ** *p* < 0.01. Bold values indicate statistically significant correlation coefficients (*p* < 0.05). Note: Sentence counts, word counts, and structural metrics are based exclusively on data collected from the first evaluation session.

**Table 5:** Changes in Accuracy, Comprehensiveness, and Readability Metrics Between the First and Second Assessments Across LLMs.

Criteria		LLMs							*p*	Effect Size
		Monica AI	Copilot	Grok 4.3	Gemini 3.1	GPT-5.5	Claude 4.6 Sonnet	DeepSeek V4		
**AC Δ**	Mean ± SD	−0.04 ± 0.59	0.12 ± 0.34	−0.01 ± 0.33	0.07 ± 0.25	0.20 ± 0.39	0.05 ± 0.27	0.15 ± 0.33	0.173	η_p_^2^ = 0.053/η_G_^2^ = 0.048
	Median	0	0	0	0	0	0	0		
	(Q1–Q3)	[−0.50–0.50]	[0.00 –0.00]	[0.00–0.00]	[0.00–0.00]	[−0.50–0.00]	[0.00–0.00]	[−0.50–0.00]		
**CO Δ**	Mean ± SD	−0.03 ± 0.56	0.04 ± 0.29	0.05 ± 0.27	0.23 ± 0.34	0.24 ± 0.47	0.04 ± 0.45	0.10 ± 0.33	0.107	η_p_^2^ = 0.062/η_G_^2^ = 0.056
	Median	0	0	0	0	0	0	0		
	(Q1–Q3)	[−0,50–0,50]	[0.00–0.00]	[0.00–0.00]	[−0.50–0.00]	[−0.50–0.00]	[−0.50–0.00]	[0.00–0.00]		
**FRE Δ**	Mean ± SD	0.29 ± 8.51	0.49 ± 8.20	0.45 ± 6.61	−0.75 ± 10.79	−1.58 ± 7.68	−0.23 ± 8.05	0.19 ± 14.98	0.924	0.008 η_p_^2^/η_G_^2^ = 0.005
	Median	0.5	0	0.15	1.05	(−1.00)	0.3	1.65		
	(Q1–Q3)	[−7.80–7.10]	[−4.10−2.20]	[−2.40−3.80]	[−8.90−7.80]	[−8.30−4.10]	[−7.20−6.70]	[−8.60–12.80]		
**FKGL Δ**	Mean ± SD	0.02 ± 1.41	−0.12 ± 1.29	−0.12 ± 1.06	0.13 ± 1.79	0.12 ± 1.50	0.01 ± 1.50	−0.35 ± 2.96	0.862	0.010 η_p_^2^/η_G_^2^ = 0.008
	Median	(−0.1)	0	(−0.05)	(−0.05)	0.25	(−0.05)	(−0.05)		
	(Q1–Q3)	[−0.6–0.9]	[−0.7–0.7]	[−0.7–0.4]	[−1.2–1.5]	[−0.5–1.3]	[−1.0–1.2]	[−2.9–1.6]		
**SMOG Δ**	Mean ± SD	−0.43 ± 1.12	1.63 ± 2.55	−0.11 ± 0.92	−0.69 ± 1.51	0.83 ± 3.23	−0.30 ± 2.83	−0.79 ± 2.06	0.004	0.126 η_p_^2^/η_G_^2^ = 0.108
	Median	(−0.10)	1.45	(−0.25)	(−0.75)	0.2	(−0.45)	0.75		
	(Q1–Q3)	[−0.80–0.80]	[−0.40–1.90]	[−0.80–0.70]	[−2.00–0.40]	[−0.60–2.80]	[−1.40–1.10]	[−1.10–2.40]		

Δ indicates the change between the second and first assessments. Comparisons were performed using repeated-measures ANOVA. Effect sizes are presented as partial eta squared (ηp^2^) and generalized eta squared (ηG^2^) from the repeated-measures ANOVA.

**Table 6:** Within-Model Between-Session Comparisons and Bland–Altman Agreement Analysis for Accuracy and Comprehensiveness.

LLM	Initial Mean ± SD	Follow Up Mean ± SD	Mean Difference ± SD	95% CI of Difference	*p*	Holm-Adjusted *p*	Bland–Altman 95% LoA
Accuracy							
**Monica AI**	4.46 ± 0.52	4.42 ± 0.53	−0.04 ± 0.59	−0.27 to 0.17	0.647 ^t^	1.000	−1.21 to 1.11
**Copilot**	4.85 ± 0.29	4.97 ± 0.13	0.12 ± 0.34	−0.01 to 0.24	0.070 ^w^	0.351	−0.55 to 0.78
**Grok 4.3**	4.88 ± 0.25	4.87 ± 0.22	−0.01 ± 0.33	−0.14 to 0.11	0.782 ^w^	1.000	−0.67 to 0.64
**Gemini 3.1**	4.83 ± 0.23	4.90 ± 0.20	0.07 ± 0.25	−0.03 to 0.16	0.157 ^w^	0.629	−0.43 to 0.56
**GPT-5.5**	4.55 ± 0.49	4.75 ± 0.39	0.20 ± 0.39	0.06 to 0.34	0.011 ^w^	0.074	−0.55 to 0.95
**Claude 4.6 Sonnet**	4.83 ± 0.35	4.88 ± 0.25	0.05 ± 0.27	−0.05 to 0.15	0.317 ^w^	0.952	−0.49 to 0.59
**DeepSeek V4**	4.83 ± 0.30	4.98 ± 0.09	0.15 ± 0.33	0.03 to 0.27	0.021 ^w^	0.125	−0.49 to 0.79
**Comprehensiveness**							
**Monica AI**	4.41 ± 0.49	4.38 ± 0.52	−0.03 ± 0.56	−0.24 to 0.17	0.727 ^w^	1.000	−1.12 to 1.06
**Copilot**	4.88 ± 0.25	4.92 ± 0.23	0.04 ± 0.29	−0.08 to 0.14	0.527 ^w^	1.000	−0.54 to 0.60
**Grok 4.3**	4.90 ± 0.24	4.95 ± 0.15	0.05 ± 0.27	−0.05 to 0.15	0.317 ^w^	1.000	−0.49 to 0.59
**Gemini 3.1**	4.70 ± 0.33	4.93 ± 0.17	0.23 ± 0.34	0.11 to 0.36	0.002 ^w^	0.013	−0.43 to 0.90
**GPT-5.5**	4.43 ± 0.48	4.67 ± 0.36	0.24 ± 0.47	0.06 to 0.41	0.016 ^w^	0.098	−0.69 to 1.15
**Claude 4.6 Sonnet**	4.78 ± 0.38	4.82 ± 0.31	0.04 ± 0.45	−0.14 to 0.20	0.672 ^w^	1.000	−0.86 to 0.92
**DeepSeek V4**	4.85 ± 0.29	4.95 ± 0.20	0.10 ± 0.33	−0.02 to 0.22	0.107 ^w^	0.534	−0.55 to 0.75

Differences were calculated as Follow Up − Initial. Within-model comparisons were performed using paired-samples *t*-tests or Wilcoxon signed-rank tests, as appropriate. Holm-adjusted *p* values account for multiple comparisons across the seven LLMs within each evaluation dimension. Bland–Altman 95% limits of agreement (LoA) were calculated as the mean difference ± 1.96 SD of the paired differences. ^t^ Analyzed using Paired *t*-test. ^w^ Analyzed using Wilcoxon signed-rank test.

## Data Availability

The original contributions presented in this study are included in the article/Supplementary Materials. Further inquiries can be directed to the corresponding author.

## Supplementary Materials

The following supporting information can be downloaded at https://www.mdpi.com/article/10.3390/diagnostics16193158/s1, File S1: Question Response; File S2: All Question; File S3: Examiners’ Scoring Matrix; File S4: LLMs response unprocessed; File S5: LLMs response stylistic elements removed.

## Abbreviations

The following abbreviations are used in this manuscript:

LLM	Large Language Model
FRE	Flesch Reading Ease
FKGL	Flesch–Kincaid Grade Level
SMOG	Simple Measure of Gobbledygook
AC	Accuracy
CO	Comprehensiveness
APIs	Application Programming Interfaces
x^2^	Chi-square statistic
df	Degrees of freedom
α_adj_	Adjusted alpha level for multiple comparisons
