# Supplementary material for: Comparative Evaluation of Large Language Models in Responding to Common Misconceptions Regarding Periodontal Disease and Oral Hygiene
Source: Diagnostics (Basel). 2026 Sep 28;16(19):3158. doi: 10.3390/diagnostics16193158 (PMC13664004; doi:10.3390/diagnostics16193158)
Supplement: Supplementary file 1 [file diagnostics-16-03158-s001.zip › Supplementary File S1 Question Response.pdf]

Category	No	Question
**Symptoms and Risk Factors**	Q1	Do gum diseases only affect elderly people?<br>**Response: No, gum diseases do not only affect elderly people. They can occur at any age. Gingivitis, which is inflammation of the gums, is particularly common among children and teenagers. Bacterial plaque that builds up on the teeth can irritate the gums and cause redness, swelling, and bleeding during brushing. A recent consensus report published in 2026 also states that gingivitis is common among children and adolescents and that its prevalence tends to increase with age. Hormonal changes during adolescence can also make the gums more sensitive to dental plaque and increase inflammation.**<br>**A more serious form of gum disease, called periodontitis, is more common in adults, and the risk increases with age. However, age is not the only factor responsible for gum disease. Poor oral hygiene, smoking, diabetes, genetic predisposition, and certain hormonal or systemic conditions can also increase the risk. If gingivitis is not properly managed, it may progress to periodontitis, which can damage the tissues and bone supporting the teeth.**<br>**Therefore, the statement "gum diseases only affect elderly people" is incorrect. Gingivitis is more commonly seen in children and young people, while severe periodontitis is more common among adults, particularly older adults. However, periodontitis can also occur in children and adolescents, especially in individuals with certain systemic diseases or genetic predispositions.**<br>**REFERENCES:**<br>**1-**Chapple I, Gizani S, Sanz M, Declerck D, Madianos PN, Clerehugh V, et al. Gingival and periodontal diseases and conditions in children and adolescents: Consensus report. *J Clin Periodontol.* 2026;53(7):1068-1099. doi:10.1111/jcpe.70033.<br>**2-**Trindade D, Carvalho R, Machado V, Chambrone L, Mendes JJ, Botelho J. Prevalence of periodontitis in dentate people between 2011 and 2020: A systematic review and meta-analysis of epidemiological studies. *J Clin Periodontol.* 2023;50(5):604-626. doi:10.1111/jcpe.13769.
	Q2	Is it normal if my gums bleed when I brush my teeth?<br>**Response: No, bleeding gums when you brush your teeth should not be considered completely normal, although it is quite common. In many cases, bleeding is a sign of gingivitis, the early stage of gum disease. Gingivitis usually occurs when dental plaque builds up along the gumline and irritates the gums, causing them to become red, swollen, and more likely to bleed.**

		**However, bleeding does not necessarily mean that you have a serious gum disease. It can often improve when you maintain good oral hygiene by brushing your teeth gently twice a day with fluoride toothpaste and cleaning between your teeth regularly with floss or interdental brushes. It is important not to stop brushing because your gums bleed; instead, gentle and consistent cleaning helps remove the plaque that is causing the irritation.**<br>**If your gums continue to bleed regularly, or if you also have swollen or painful gums, persistent bad breath, receding gums, or loose teeth, you should see a dentist. These symptoms can indicate a more advanced form of gum disease called periodontitis, which can damage the tissues and bone supporting the teeth if it is left untreated.**<br><br>**REFERENCES:**<br>**1-**Chapple ILC, Mealey BL, Van Dyke TE, et al. Periodontal health and gingival diseases and conditions on an intact and a reduced periodontium: Consensus report of Workgroup 1 of the 2017 World Workshop on the Classification of Periodontal and Peri-Implant Diseases and Conditions. J Clin Periodontol. 2018;45(Suppl 20):S68-S77. doi:10.1111/jcpe.12940.<br>**2-**Chapple ILC, Van der Weijden F, Doerfer C, et al. Primary prevention of periodontitis: managing gingivitis. J Clin Periodontol. 2015;42(Suppl 16):S71-S76. doi:10.1111/jcpe.12366.
	Q3	Can I get gum disease even if I brush my teeth regularly?<br>**Response: Yes, you can still develop gum disease even if you brush your teeth regularly. Brushing your teeth twice a day is one of the most important ways to prevent gum disease, but it does not completely eliminate the risk. Gum disease is mainly caused by dental plaque, a sticky layer of bacteria that can build up around and between the teeth. If plaque is not removed effectively, it can irritate the gums and cause gingivitis.**<br>**One reason is that brushing alone may not clean all areas of the mouth, especially between the teeth and just below the gumline. Regular cleaning between the teeth with dental floss or interdental brushes can help remove plaque from these difficult-to-reach areas. In addition, factors such as smoking, diabetes, hormonal changes, certain medications, genetics, and some medical conditions can increase a person's risk of developing gum disease even when they brush regularly.**<br>**Therefore, brushing regularly significantly reduces the risk of gum disease, but it does not guarantee that you will never develop it. Good oral hygiene should include brushing twice a day with fluoride toothpaste, cleaning between the teeth regularly,**

		**and having regular dental check-ups. If your gums frequently bleed, swell, or become painful despite good oral hygiene, it is a good idea to visit a dentist because these can be signs of gum disease.**<br>**REFERENCES**:<br>**1-** Chapple ILC, Mealey BL, Van Dyke TE, et al. Periodontal health and gingival diseases and conditions on an intact and a reduced periodontium: Consensus report of Workgroup 1 of the 2017 World Workshop on the Classification of Periodontal and Peri-Implant Diseases and Conditions. *J Clin Periodontol.* 2018;45(Suppl 20):S68-S77. doi:10.1111/jcpe.12940.<br>**2-**Chapple ILC, Van der Weijden F, Doerfer C, et al. Primary prevention of periodontitis: managing gingivitis. *J Clin Periodontol.* 2015;42(Suppl 16):S71-S76. doi:10.1111/jcpe.12366.
	Q4	Can I have gum disease even if my gums don't hurt?<br>**Response: Yes, you can have gum disease even if your gums do not hurt. In fact, gum disease can develop without causing noticeable pain, especially in its early stages. Gingivitis, the earliest stage of gum disease, may cause symptoms such as redness, swelling, tenderness, or bleeding when brushing, but some people may have very few or no obvious symptoms.**<br>**This is particularly important with periodontitis, a more advanced form of gum disease. Periodontitis can gradually damage the tissues and bone that support the teeth, and it may progress without significant pain. Some possible warning signs include bleeding gums, persistent bad breath, receding gums, loose teeth, or changes in the way the teeth fit together when biting.**<br>**Therefore, the absence of pain does not necessarily mean that your gums are healthy. Regular brushing, cleaning between your teeth, and dental check-ups are important because a dentist can detect gum problems even when you do not notice any symptoms yourself.**<br>**REFERENCES**:<br>**1-**Trombelli L, Farina R, Silva CO, Tatakis DN. Plaque-induced gingivitis: Case definition and diagnostic considerations. *J Periodontol.* 2018;89(Suppl 1):S46-S73. doi:10.1002/JPER.17-0576.<br>**2-** Salvi GE, Roccuzzo A, Imber J-C, Stähli A, Klinge B, Lang NP. Clinical periodontal diagnosis. *Periodontol 2000.* 2023;00:1-19. doi:10.1111/prd.12487.
	Q5	Can gum disease get better without treatment?<br>**Response: Yes, mild gum disease, especially gingivitis, can often improve with good oral hygiene without professional treatment. Gingivitis is usually caused by plaque buildup around the teeth and gums. If the plaque is removed regularly through**

		**proper brushing and cleaning between the teeth, the inflammation can gradually decrease and the gums can return to a healthier condition. However, it is important not to ignore symptoms such as bleeding, redness, or swelling, because untreated gingivitis can sometimes progress to a more serious condition.**<br>**More advanced gum disease, known as periodontitis, usually does not go away on its own. It can cause permanent damage to the tissues and bone supporting the teeth and generally requires professional dental treatment to control the infection and prevent further damage. Good oral hygiene is still essential, but it may not be enough by itself once periodontitis has developed.**<br>**Therefore, if your gums are occasionally irritated or bleed slightly, improving your brushing and cleaning between your teeth may help. However, if bleeding continues, your gums remain swollen or painful, or you notice loose or sensitive teeth, you should see a dentist for an evaluation. Early treatment can prevent gum disease from becoming more serious.**<br>**REFERENCES**:<br>**1-**Chapple ILC, Van der Weijden F, Doerfer C, et al. Primary prevention of periodontitis: managing gingivitis. *J Clin Periodontol.* 2015;42(Suppl 16):S71-S76. doi:10.1111/jcpe.12366.<br>**2-**Trombelli L, Farina R, Silva CO, Tatakis DN. Plaque-induced gingivitis: Case definition and diagnostic considerations. *J Clin Periodontol.* 2018;45(Suppl 20):S44-S67. doi:10.1111/jcpe.12939.<br>**3-**Sanz M, Herrera D, Kebschull M, Chapple I, Jepsen S, Berglundh T, et al. Treatment of stage I–III periodontitis—The EFP S3 level clinical practice guideline. *J Clin Periodontol.* 2020;47(Suppl 22):4-60. doi:10.1111/jcpe.13290
	Q6	Are gum diseases rare?<br>**Response: No, gum diseases are not rare. In fact, they are among the most common oral health problems worldwide. The World Health Organization (WHO) estimates that severe periodontal disease affects about 1 billion people worldwide, and its estimated prevalence among people aged 15 and older was about 19%, meaning roughly 1 in 5 people were affected in 2019.**<br>**Gum disease includes conditions such as gingivitis and periodontitis. Gingivitis is the milder form and can cause red, swollen, or bleeding gums. Periodontitis is more serious because it can damage the tissues and bone that support the teeth. In the United States, for example, the CDC reports that about 42% of adults aged 30 and older have periodontitis, while severe periodontitis affects about 8% of adults.**

		**Therefore, gum disease is very common rather than rare. However, the good news is that many cases can be prevented or controlled through good oral hygiene, regular dental check-ups, and avoiding risk factors such as smoking.**<br>**REFERENCES**:<br>**1-**World Health Organization. *Global oral health status report: towards universal health coverage for oral health by 2030.* Geneva: World Health Organization; 2022.<br>**2-**Chapple ILC, Van der Weijden F, Doerfer C, et al. Primary prevention of periodontitis: managing gingivitis. *J Clin Periodontol.* 2015;42(Suppl 16):S71-S76. doi:10.1111/jcpe.12366.
	Q7	Is it normal to lose teeth because of gum disease as you get older?<br>**Response: No, losing teeth because of gum disease as you get older is not considered a normal or inevitable part of aging. It is true that gum disease becomes more common with age, and the damage caused by periodontitis can accumulate over many years. However, with appropriate oral hygiene, regular dental care, and treatment when necessary, many older adults can keep their natural teeth throughout their lives. The National Institute of Dental and Craniofacial Research (NIDCR) reports that the prevalence of periodontitis increases with age, but tooth loss among older adults has actually decreased over recent decades, showing that tooth loss is not simply an unavoidable consequence of getting older.**<br>**When gum disease becomes advanced, it can destroy the gums, periodontal tissues, and bone that support the teeth. As this support is lost, teeth can become loose and may eventually need to be removed. Periodontitis is considered one of the major causes of tooth loss in adults. Therefore, if an older person loses teeth because of gum disease, it is a sign of untreated or advanced periodontal disease rather than something that should simply be accepted as a normal part of aging. Early diagnosis and treatment can help prevent further damage and tooth loss.**<br>**REFERENCES**:<br>**1-**National Institute of Dental and Craniofacial Research. *Periodontal Disease in Adults (Age 30 or Older).* National Institutes of Health; 2026.<br>**2-**National Institute of Dental and Craniofacial Research. *Tooth Loss in Seniors.* National Institutes of Health; 2026.<br>**3-**Winning L, Bertl K, Polyzois I, van der Waal Y, Kahatab R, Harrison P, McKenna G. The impact of periodontitis on oral health outcomes in older adults: a systematic review. *Gerodontology.* 2026;43(3):318-338.
	Q8	If gum disease runs in my family, will I definitely get it too?<br>**Response:** **No, if gum disease runs in your family, it does not mean that you will definitely develop it. Genetics can influence how susceptible a person is to gum**

		**disease, but having a family history is only one of many risk factors. Factors such as dental plaque, oral hygiene, smoking, diabetes, certain medications, hormonal changes, and access to regular dental care can also affect the likelihood of developing gum disease.**<br>**Some people may be genetically more susceptible to periodontitis, meaning their immune system or inflammatory response may react differently to the bacteria associated with gum disease. However, genetic susceptibility does not guarantee that the disease will develop. Maintaining good oral hygiene, including brushing twice a day with fluoride toothpaste and cleaning between the teeth, can help reduce the amount of plaque and lower the risk.**<br>**Therefore, having a family history of gum disease means you may have a higher risk, not that you will definitely get it. If gum disease is common in your family, regular dental check-ups are especially useful because early gum problems may not cause pain or obvious symptoms.**<br>**REFERENCE**:<br>**1-**Shaddox LM, Morford LA, Nibali L. Periodontal health and disease: The contribution of genetics. *Periodontol 2000.* 2021;85(1):161-181. doi:10.1111/prd.12357.
	Q9	Can stress make me more likely to get gum disease?<br>**Response: Yes, stress may make you more likely to develop gum disease or may make existing gum disease worse, but stress alone does not directly cause gum disease. The main cause of periodontal disease is the accumulation of bacteria in dental plaque, while stress can influence how the body responds to infection and inflammation. Research suggests that psychological stress may affect the immune and inflammatory responses involved in periodontal disease.**<br>**Stress can also affect oral health indirectly. When people are under prolonged stress, they may find it harder to maintain good oral-hygiene habits, and some may smoke more, clench or grind their teeth, or neglect regular dental care. These behaviors can increase the risk of periodontal problems. Recent research also suggests that stress may be associated with poorer periodontal treatment outcomes, although the exact relationship is complex and more long-term research is still needed.**<br>**Therefore, being stressed does not mean you will definitely develop gum disease, but chronic or severe stress may increase your susceptibility, particularly when**

		**combined with other risk factors. Maintaining good oral hygiene, cleaning between your teeth, having regular dental check-ups, and managing long-term stress can all help protect your gums.**<br>**REFERENCES**:<br>**1-**Aggarwal K, Gupta J, Kaur RK, Bansal D, Jain A. Effect of anxiety and psychologic stress on periodontal health: a systematic review and meta-analysis. *Quintessence Int.* 2022;53(2):144-154. doi:10.3290/j.qi.b2091191.<br>**2-**Mauland EK, Neupane SP. The influence of psychological stress and stress-related disorders in the treatment outcome of periodontitis: a systematic review and meta-analysis. *J Clin Periodontol.* 2025;52(12):1760-1778. doi:10.1111/jcpe.70022.
	Q10	Do gum diseases only affect the mouth and teeth?<br>**Response: No, gum diseases do not only affect the mouth and teeth. Although gum disease begins in the tissues surrounding the teeth, particularly in the gums, research shows that there is an important connection between oral health and overall health. Periodontitis is a chronic inflammatory disease, and the inflammation and bacteria associated with it may interact with other systems in the body.**<br>**There is a particularly strong and well-established relationship between gum disease and diabetes. People with diabetes are more likely to develop periodontal disease, while severe gum disease may also make blood sugar control more difficult. This relationship appears to work in both directions, although researchers are still studying the exact mechanisms involved.**<br>**Gum disease has also been associated with cardiovascular disease, stroke, pregnancy complications, respiratory conditions, and some other chronic diseases. However, it is important to understand that an association does not always mean that gum disease directly causes these conditions. Many of these diseases share common risk factors, such as smoking, poor diet, obesity, and diabetes, so researchers are still investigating exactly how periodontal disease contributes to overall health.**<br>**Therefore, gum disease should not be viewed as a problem that affects only the teeth and gums. Maintaining healthy gums is also an important part of maintaining general health. Good oral hygiene, regular dental check-ups, and treatment of gum disease when necessary can help protect both oral and overall health. The World Health Organization also emphasizes that oral health is an integral part of general health.**

		**REFERENCES**:<br><br>**1-**Genco RJ, Sanz M. Clinical and public health implications of periodontal and systemic diseases: an overview. *Periodontol 2000.* 2020;83(1):7-13. doi:10.1111/prd.12344.<br>**2-**Herrera D, Sanz M, Shapira L, et al. Association between periodontal diseases and cardiovascular diseases, diabetes and respiratory diseases: Consensus report of the Joint Workshop by the European Federation of Periodontology (EFP) and the European arm of the World Organization of Family Doctors (WONCA Europe). *J Clin Periodontol.* 2023;50(6):819-841. doi:10.1111/jcpe.13807.
**Oral Care and Daily Habits**	Q11	Will a hard-bristled toothbrush clean my teeth better?<br><br>**Response: No. A hard-bristled toothbrush does not necessarily clean your teeth better, and using one can actually damage your gums and the protective surface of your teeth if you brush too aggressively. What matters more is brushing thoroughly with the correct technique rather than using a hard-bristled brush. Dental professionals generally recommend a soft-bristled toothbrush, because it can effectively remove plaque while being gentler on the gums and tooth enamel.**<br>**Using a hard-bristled toothbrush or applying too much pressure may contribute to gum irritation, gum recession, and abrasion of the tooth surface over time. This is particularly important if your gums are already sensitive or tend to bleed. A soft toothbrush, used with gentle movements along the gumline, is usually a safer choice for everyday brushing.**<br>**Therefore, harder does not mean cleaner. For most people, a soft-bristled toothbrush combined with proper brushing technique and regular cleaning between the teeth provides effective plaque removal without unnecessarily damaging the gums or teeth.**<br>**REFERENCES**:<br><br>**1-**Ranzan N, Muniz FWMG, Rösing CK. Are bristle stiffness and bristle end-shape related to adverse effects on soft tissues during toothbrushing? A systematic review. *Int Dent J.* 2019;69(3):171-182. doi:10.1111/idj.12421.<br>**2-**Zimmer S, Öztürk M, Barthel CR, Bizhang M, Jordan RA. Cleaning efficacy and soft tissue trauma after use of manual toothbrushes with different bristle stiffness. *J Periodontol.* 2011;82(2):267-271. doi:10.1902/jop.2010.100328.
	Q12	Should I stop brushing my teeth for a few days if my gums are bleeding? **Response: No, you should not stop brushing your teeth for a few days just because your gums are bleeding. In fact, stopping brushing can allow more dental plaque to build up, which can make gum inflammation and bleeding worse. Dental guidance recommends continuing to brush twice a day with fluoride toothpaste, using gentle movements rather than brushing aggressively.**

		**Bleeding while brushing is often a sign of gum inflammation (gingivitis) caused by plaque buildup. Continuing to clean your teeth helps remove the plaque responsible for the irritation. You can also gently clean between your teeth with floss or interdental brushes, because toothbrush bristles cannot effectively reach all the spaces between teeth.**<br>**It is also better to use a soft-bristled toothbrush and avoid pressing too hard. If the bleeding continues for several days, happens frequently, or is accompanied by swollen or painful gums, bad breath, receding gums, or loose teeth, you should see a dentist because these can be signs of gum disease that needs professional treatment.**<br>**REFERENCE**:<br>**1-**Chapple ILC, Van der Weijden F, Doerfer C, et al. Primary prevention of periodontitis: managing gingivitis. *J Clin Periodontol.* 2015;42(Suppl 16):S71-S76. doi:10.1111/jcpe.12366.
	Q13	Do I need to brush my teeth after every meal to keep my gums healthy? **Response: No, you do not need to brush your teeth after every meal to keep your gums healthy. For most people, brushing twice a day—once in the morning and once before going to bed—with fluoride toothpaste is sufficient to maintain good oral hygiene. The important thing is to brush thoroughly and gently, especially along the gumline, where plaque tends to accumulate.**<br>**Brushing after every meal is not necessarily harmful, but it is usually unnecessary and may be too frequent for some people, especially if they brush aggressively. After consuming acidic foods or drinks, such as citrus fruits or soft drinks, it is recommended to wait about an hour before brushing, because acids can temporarily soften tooth enamel and brushing immediately afterward may contribute to enamel wear. Rinsing your mouth with water after eating can be a useful alternative.**<br>**Therefore, a good routine is to brush twice a day with a soft-bristled toothbrush and fluoride toothpaste and clean between your teeth regularly. Regular dental check-ups are also important for detecting gum problems early. Brushing properly and consistently is more important for gum health than brushing after every meal.**<br>**REFERENCES**:<br>**1-**Chapple ILC, Van der Weijden F, Doerfer C, et al. Primary prevention of periodontitis: managing gingivitis. *J Clin Periodontol.* 2015;42(Suppl 16):S71-S76. doi:10.1111/jcpe.12366.

		**2-**Attin T, Knöfel S, Buchalla W, Tütüncü R. In situ evaluation of different remineralization periods to decrease brushing abrasion of demineralized enamel. *Caries Res.* 2001;35(3):216-222. doi:10.1159/000047459.
	Q14	Even if I brush my teeth regularly, do I still need to get my gums checked? **Response: Yes, even if you brush your teeth regularly, it is still important to have your gums checked by a dentist. Brushing twice a day helps remove plaque and greatly reduces the risk of gum disease, but brushing alone cannot always clean every area, especially between the teeth and below the gumline. Plaque that remains in these areas can harden into tartar, which cannot be removed effectively with a toothbrush and requires professional cleaning.**<br>**Regular dental check-ups are also important because gum disease does not always cause noticeable symptoms, particularly in its early stages. During a dental examination, a dentist or dental hygienist can look for signs of inflammation and measure the spaces around the teeth to identify possible periodontal disease. Detecting problems early makes them easier to manage and can help prevent more serious damage to the tissues and bone supporting the teeth.**<br>**Therefore, good brushing and regular dental check-ups work together. Brushing and cleaning between your teeth help prevent gum disease at home, while professional examinations and cleanings can detect problems that you may not notice yourself and remove tartar that home care cannot remove. How often you need a check-up can depend on your individual risk, so your dentist can recommend an appropriate schedule.**<br>**REFERENCE**:<br>**1-**Clarkson JE, Pitts NB, Goulao B, et al. Examining the effectiveness of different dental recall strategies on maintenance of optimum oral health: the INTERVAL dental recalls randomised controlled trial. *Br Dent J.* 2021;230:236-243. doi:10.1038/s41415-021-2612-0.
	Q15	Is brushing my teeth once a day enough to keep my mouth healthy? **Response: No, brushing your teeth only once a day is generally not enough to maintain optimal oral health. Dental professionals recommend brushing twice a day for about two minutes each time, using fluoride toothpaste. Brushing twice daily helps remove dental plaque, which is a major cause of tooth decay and gum disease.**<br>**Brushing only once a day may remove some plaque, but plaque can build up again throughout the day. If it remains on the teeth and around the gumline, it can contribute to gingivitis, which may cause red, swollen, or bleeding gums. Over time,**

		**untreated gum inflammation can develop into more serious periodontal disease. Cleaning between the teeth with floss or interdental brushes is also important because a toothbrush cannot reach all surfaces effectively.**<br>**Therefore, brushing twice a day with fluoride toothpaste, combined with daily cleaning between the teeth and regular dental check-ups, is a much better routine for maintaining healthy teeth and gums. If you can only brush once on a particular day, it is still better than not brushing at all, but you should aim for twice-daily brushing as your normal routine.**<br>**REFERENCES**:<br>**1-**Chapple ILC, Van der Weijden F, Doerfer C, et al. Primary prevention of periodontitis: managing gingivitis. *J Clin Periodontol.* 2015;42(Suppl 16):S71-S76. doi:10.1111/jcpe.12366.<br>**2-**Kumar S, Tadakamadla J, Johnson NW. Effect of toothbrushing frequency on incidence and increment of dental caries: a systematic review and meta-analysis. *J Dent Res.* 2016;95(11):1230-1236. doi:10.1177/0022034516655315.
	Q16	Can flossing cause gum recession?<br>**Response: No, proper flossing does not normally cause gum recession. In fact, cleaning between your teeth regularly with dental floss can help prevent gum disease by removing plaque from areas that a toothbrush cannot reach. Gum recession is more commonly associated with factors such as gum disease, aggressive brushing, trauma to the gums, smoking, and certain anatomical or genetic factors. However, flossing too aggressively or using the wrong technique can irritate or injure the gums. For example, snapping the floss forcefully into the gums or repeatedly pressing it too hard can cause temporary bleeding or soreness. This does not mean that you should stop flossing; instead, the floss should be guided gently between the teeth and curved around each tooth to clean along the gumline without forcing it into the tissue.**<br>**It is also worth noting that bleeding when you first start flossing is often a sign of existing gum inflammation rather than damage caused by flossing. With consistent and gentle cleaning, the inflammation and bleeding should generally improve. If your gums continue to bleed, recede, or become painful despite good oral hygiene, a dentist should examine them to determine the cause.**<br>**Therefore, flossing correctly is beneficial for your gums and does not normally cause gum recession. The key is to use gentle, controlled movements rather than forcing or snapping the floss.**

		**REFERENCES**:<br><br>**1-**Worthington HV, MacDonald L, Poklepovic Pericic T, et al. Home use of interdental cleaning devices, in addition to toothbrushing, for preventing and controlling periodontal diseases and dental caries. *Cochrane Database Syst Rev.* 2019;4(4):CD012018. doi:10.1002/14651858.CD012018.pub2.<br>**2-**Marschner F, Lechte C, Kanzow P, Hraský V, Pfister W. Systematic review and meta-analysis on prevalence and risk factors for gingival recession. *J Dent.* 2025;155:105645. doi:10.1016/j.jdent.2025.105645.
	Q17	Do electric toothbrushes damage the gums more than regular toothbrushes? **Response: No. Electric toothbrushes do not generally damage the gums more than regular manual toothbrushes. When used correctly, both types can clean the teeth effectively and help maintain healthy gums. In fact, research suggests that powered toothbrushes can provide slightly better plaque removal and may reduce gingivitis compared with manual toothbrushes.**<br>**The main issue is how you brush rather than whether the toothbrush is electric or manual. Applying too much pressure with either type can irritate the gums and, over time, contribute to gum recession or damage to the tooth surface. Many electric toothbrushes have pressure sensors that alert you when you are pressing too hard, which can actually make it easier to avoid excessive pressure.**<br>**Therefore, an electric toothbrush is generally safe for the gums when used properly. Using a soft-bristled brush head, applying gentle pressure, and following the manufacturer's instructions can help protect the gums. If your gums frequently bleed, become painful, or appear to be receding, it is a good idea to have them checked by a dentist.**<br>**REFERENCES**:<br><br>**1-**Yaacob M, Worthington HV, Deacon SA, Deery C, Walmsley AD, Robinson PG, Glenny AM. Powered versus manual toothbrushing for oral health. *Cochrane Database Syst Rev.* 2014;(6):CD002281. doi:10.1002/14651858.CD002281.pub3.<br>**2-**Molina C, Pimentel RP, Oballe HJR, Muniz FWMG, Rösing CK. Influence of manual and powered toothbrushes on gingival lesions: a systematic review and meta-analysis. *Int J Dent Hyg.* 2026:1-10. doi:10.1111/idh.70086.
	Q18	Would using mouthwash alone be enough without brushing my teeth? **Response: No, using mouthwash alone is not enough to keep your teeth and gums healthy. Mouthwash can be a useful addition to an oral-care routine, but it cannot replace brushing and cleaning between the teeth. Brushing physically removes dental plaque and food debris from the surfaces of the teeth, while mouthwash mainly**

		**helps reduce bacteria and can provide additional benefits depending on its ingredients.**<br>**This is particularly important for gum health. Plaque can accumulate along the gumline and between the teeth, where it can cause inflammation and lead to gingivitis. If plaque is not removed regularly, it can eventually harden into tartar, which cannot be removed effectively by mouthwash and usually requires professional cleaning. Therefore, simply rinsing with mouthwash does not provide the same cleaning effect as brushing and interdental cleaning.**<br>**For good oral health, it is generally recommended to brush your teeth twice a day with fluoride toothpaste and clean between your teeth daily. Mouthwash can be added to this routine if appropriate, but it should be considered an additional measure rather than a substitute for brushing.**<br>**REFERENCES**:<br>**1-**Chapple ILC, Van der Weijden F, Doerfer C, et al. Primary prevention of periodontitis: managing gingivitis. *J Clin Periodontol.* 2015;42(Suppl 16):S71-S76. doi:10.1111/jcpe.12366.<br>**2-**Figuero E, Roldán S, Serrano J, Escribano M, Martín C, Preshaw PM. Efficacy of adjunctive therapies in patients with gingival inflammation: a systematic review and meta-analysis. *J Clin Periodontol.* 2020;47(Suppl 22):125-143. doi:10.1111/jcpe.13244.
	Q19	After I quit smoking, my gums started bleeding. Is that normal?<br>**Response: Yes, it can happen after quitting smoking, and it may be related to changes in your gums after you stop smoking. Smoking can reduce blood flow to the gums and can mask some of the signs of gum inflammation. When you quit, blood flow and the gums' normal inflammatory response can improve, so bleeding may become more noticeable. An NHS oral-health resource specifically notes that some people may notice their gums bleeding after quitting smoking because the blood vessels in the gums become better oxygenated and respond to existing gum inflammation.**<br>**However, bleeding gums should not simply be assumed to be a normal consequence of quitting smoking. Bleeding is also a common sign of gingivitis or periodontal disease. Smoking is itself a major risk factor for gum disease, so if you previously smoked, you may have had underlying gum inflammation that was less obvious while smoking.**<br>**The best approach is to continue brushing your teeth twice a day with a soft-bristled toothbrush and clean between your teeth every day. Do not stop brushing**

		**because of the bleeding. If the bleeding continues, becomes heavy, or is accompanied by swollen gums, persistent bad breath, gum recession, or loose teeth, you should have your gums examined by a dentist.**<br>**Bleeding can occur after quitting smoking, but it can also be a sign of existing gum inflammation. Quitting smoking is nevertheless one of the best things you can do for your oral and general health.**<br>**REFERENCES**:<br>**1-**Nair P, Sutherland G, Palmer RM, Wilson RF, Scott DA. Gingival bleeding on probing increases after quitting smoking. *J Clin Periodontol.* 2003;30(5):435-437. doi:10.1034/j.1600-051X.2003.20039.x.<br>**2-**Buduneli N, Scott DA. Tobacco-induced suppression of the vascular response to dental plaque. *Mol Oral Microbiol.* 2018;33(4):271-282. doi:10.1111/omi.12228.
	Q20	Can natural products effectively keep my gums healthy?<br>**Response: Yes, some natural products may provide additional benefits for gum health, but they should not replace the basic methods of oral hygiene. Some herbal toothpastes and mouthwashes have been studied for their ability to reduce dental plaque and gingivitis, and research suggests that certain products may have some effectiveness. However, the evidence is generally not strong enough to conclude that natural products are better than conventional oral-care products. A systematic review found that herbal toothpaste could be effective, but it was not superior to fluoride toothpaste, while evidence for herbal mouthwashes was limited in quality. For keeping your gums healthy, the most important steps remain brushing twice a day with fluoride toothpaste and cleaning between your teeth regularly. These methods are recommended by the National Institute of Dental and Craniofacial Research (NIDCR) because they effectively remove plaque, which is a major cause of gum disease.**<br>**Natural products such as herbal mouthwashes can therefore be considered an additional part of oral care, but using something simply because it is "natural" does not automatically make it safer or more effective. For example, a mouthwash should not be used as a substitute for brushing and flossing. The American Dental Association also emphasizes that mouthwash, whether conventional or containing particular natural ingredients, is an addition to—not a replacement for—proper brushing and interdental cleaning.**

		**Therefore, natural products can potentially help support gum health, but good brushing, cleaning between the teeth, fluoride toothpaste, and regular dental check-ups are still the most reliable ways to protect your gums.**<br>**REFERENCES**:<br>**1-**Mehta V, Mathur A, Tripathy S, Rizwan SA, Sharma T. Effectiveness of herbal oral care products in reducing dental plaque and gingivitis: an overview of systematic reviews. *Can J Dent Hyg.* 2024;58(2):120-134.<br>**2-**Chapple ILC, Van der Weijden F, Doerfer C, et al. Primary prevention of periodontitis: managing gingivitis. *J Clin Periodontol.* 2015;42(Suppl 16):S71-S76. doi:10.1111/jcpe.12366.
**Treatment Process**	Q21	Can getting a dental scaling damage my teeth?<br>**Response: No, dental scaling (professional removal of plaque and tartar) does not normally damage healthy teeth when it is performed properly by a dental professional. Scaling is specifically used to remove hardened tartar that cannot be removed effectively with a toothbrush, and it can be an important part of preventing and treating gum disease. The American Dental Association describes scaling and root planing as a treatment used to remove plaque and tartar from above and below the gumline.**<br>**After scaling, some people may experience temporary tooth sensitivity, gum tenderness, slight bleeding, or the feeling that their teeth look more separated. These effects do not necessarily mean that the teeth have been damaged. In particular, when a significant amount of tartar has been removed, the teeth may feel different because the tartar that had been covering parts of the tooth is gone. Sensitivity can also occur when previously covered root surfaces become exposed during periodontal treatment.**<br>**There is a small but important distinction when it comes to dental restorations such as fillings, crowns, or other materials. Research shows that ultrasonic scaling can affect the surface roughness of some restorative materials, with the degree depending on the material and the instrument used. This is different from saying that scaling damages natural tooth enamel.**<br>**Therefore, you generally should not avoid scaling because you are worried that it will wear away your teeth. If you have tartar buildup or gum disease, professional cleaning can be beneficial. The dentist or dental hygienist can also choose an appropriate instrument and technique based on your teeth, gums, fillings, crowns, or other dental work.**

		**REFERENCES**:<br><br>**1-**Smiley CJ, Tracy SL, Abt E, et al. Evidence-based clinical practice guideline on the nonsurgical treatment of chronic periodontitis by means of scaling and root planing with or without adjuncts. *J Am Dent Assoc.* 2015;146(7):525-535. doi:10.1016/j.adaj.2015.01.026.<br>**2-**Graziani F, Tinto M, Orsolini C, Izzetti R, Tomasi C. Complications and treatment errors in nonsurgical periodontal therapy. *Periodontol 2000.* 2023;92(1):21-61. doi:10.1111/prd.12478.
	Q22	Can dental scaling cause gaps between my teeth?<br><br>**Response: Yes, your teeth can sometimes appear to have larger gaps after dental scaling, but scaling itself does not normally create new spaces between healthy teeth. This can happen because tartar (calculus) may have accumulated between the teeth or around the gumline. When the tartar is removed, the underlying spaces that were already present become visible, making the teeth look more separated. Another reason is gum disease. If you have had gingivitis or periodontitis, the gums may have been swollen before the cleaning. After the inflammation decreases following scaling, the gums can shrink back toward their healthier position. This can make spaces between the teeth, sometimes called "black triangles," more noticeable. In cases of periodontitis, there may also have been loss of bone and gum tissue before the scaling, which can contribute to these spaces.**<br>**Therefore, if you notice gaps after scaling, it usually does not mean that the dentist damaged or moved your teeth. More often, the cleaning has removed tartar and reduced swelling, revealing the actual shape and position of the teeth and gums. If a gap is new, continues to increase, or is accompanied by loose teeth or significant gum recession, you should have it evaluated by a dentist to check for periodontal disease.**<br>**REFERENCES**:<br><br>**1-**Kaufmann ME, Wiedemeier DB, Zellweger U, Solderer A, Attin T, Schmidlin PR. Gingival recession after scaling and root planing with or without systemic metronidazole and amoxicillin: a re-review. *Clin Oral Investig.* 2020;24(3):1091-1100. doi:10.1007/s00784-020-03198-4.<br>**2-**Barakat SO. Interdental papilla recession and reconstruction of the lost triangle: a review of the current literature. *Front Dent Med.* 2025;5:1537452. doi:10.3389/fdmed.2024.1537452.
	Q23	Do all gum diseases require the same treatment?<br><br>**Response: No, all gum diseases do not require the same treatment. The appropriate treatment depends on the type and severity of the gum disease, as well as the condition of the teeth and the supporting tissues.**

		**For example, gingivitis, which is the early and usually reversible form of gum disease, is generally managed by improving oral hygiene and removing plaque and tartar through professional cleaning when necessary. Regular brushing with fluoride toothpaste and cleaning between the teeth can often help the gums return to a healthy condition.**<br>**Periodontitis, on the other hand, is a more serious condition in which the tissues and bone supporting the teeth can be damaged. It usually requires professional periodontal treatment, which may include scaling and root planing, improved daily oral hygiene, and regular periodontal monitoring. In more advanced cases, additional treatments such as periodontal surgery may be necessary.**<br>**There are also other types of gum conditions that may have different causes and therefore require different approaches. For example, gum problems related to certain medications, hormonal changes, systemic diseases, or infections may require treatment of the underlying condition in addition to maintaining good oral hygiene. Therefore, there is no single treatment that works for every gum disease. A dentist or periodontist needs to determine the type and severity of the condition before choosing the appropriate treatment. The earlier gum disease is detected, the easier it is generally to manage and prevent further damage.**<br>**REFERENCES**:<br>**1-**Murakami S, Mealey BL, Mariotti A, Chapple ILC. Dental plaque-induced gingival conditions. *J Clin Periodontol.* 2018;45(Suppl 20):S17-S27. doi:10.1111/jcpe.12937.<br>**2-**Sanz M, Herrera D, Kebschull M, et al. Treatment of stage I–III periodontitis—The EFP S3 level clinical practice guideline. *J Clin Periodontol.* 2020;47(Suppl 22):4-60. doi:10.1111/jcpe.13290.
	Q24	Is gum treatment painful?<br>**Response: Gum treatment does not necessarily have to be painful. The amount of discomfort depends on the type of treatment and how severe the gum disease is. For mild gingivitis, treatment may involve professional cleaning and improving oral hygiene, which usually causes little discomfort. If deeper cleaning such as scaling and root planing is needed for periodontitis, the dentist can use a local anesthetic to numb the gums and make the procedure more comfortable.**<br>**After deeper periodontal treatment, it is possible to experience temporary sensitivity, mild soreness, or tenderness, particularly around areas where there was significant inflammation or where the tooth roots have become exposed. These**

		**symptoms generally improve as the gums heal. Following the dentist's instructions for oral hygiene and eating can help with recovery.**<br>**The important point is that avoiding treatment because of fear of pain can allow gum disease to become more serious. Untreated periodontitis can gradually damage the bone and tissues supporting the teeth. Modern dental techniques and local anesthesia can make most periodontal procedures much more manageable.**<br>**So, while some gum treatments may cause temporary discomfort, they should not normally be severely painful. If you are worried about pain, you can tell your dentist beforehand; they can discuss anesthesia and ways to make the procedure more comfortable.**<br>**REFERENCES**:<br>**1-**Santos EC, Huller D, Brigola S, Ferreira MD, Pochapski MT, Dos Santos FA. Pain management in periodontal therapy using local anesthetics and other drugs: an integrative review. *J Dent Anesth Pain Med.* 2023;23(5):245-256. doi:10.17245/jdapm.2023.23.5.245.<br>**2-**Lin YH, Gillam DG. The prevalence of root sensitivity following periodontal therapy: a systematic review. *Int J Dent.* 2012;2012:407023. doi:10.1155/2012/407023.
	Q25	Can gum surgery cause tooth loss?<br>**Response: No, gum surgery does not normally cause tooth loss. In fact, periodontal surgery is usually performed for the opposite reason: to control gum disease, protect the tissues supporting the teeth, and help prevent further tooth loss. During periodontal surgery, the dentist or periodontist can access areas that are difficult to clean, remove bacteria and tartar from deep gum pockets, and sometimes regenerate lost bone or tissue.**<br>**After surgery, it is possible for teeth to feel slightly looser temporarily. This can happen because the gums and surrounding tissues are healing, and it does not necessarily mean that the tooth will fall out. Some patients may also notice increased sensitivity, gum shrinkage, or spaces between their teeth becoming more visible after the inflammation decreases. These effects can be part of the healing process.**<br>**However, in cases where gum disease is already very advanced, a tooth may have lost so much supporting bone that it cannot be saved. In such situations, extraction may sometimes be necessary, but this is due to the severity of the existing periodontal disease rather than the surgery itself. Periodontitis can destroy the bone and tissues supporting teeth and is a major cause of tooth loss in adults.**

		**Therefore, gum surgery itself is generally intended to save teeth, not cause them to be lost. If a tooth becomes significantly looser after surgery, or if you experience increasing pain, swelling, or persistent bleeding, you should contact your dentist or periodontist for an examination.**<br>**REFERENCES**:<br>**1-**Sanz M, Herrera D, Kebschull M, et al. Treatment of stage I–III periodontitis—The EFP S3 level clinical practice guideline. *J Clin Periodontol.* 2020;47(Suppl 22):4-60. doi:10.1111/jcpe.13290.<br>**2-**Stavropoulos A, Bertl K, Spineli LM, et al. Medium- and long-term clinical benefits of periodontal regenerative/reconstructive procedures in intrabony defects: systematic review and network meta-analysis of randomized controlled clinical studies. *J Clin Periodontol.* 2021;48(3):410-430. doi:10.1111/jcpe.13409.
	Q26	Can gum treatment make my teeth feel loose?<br>**Response: Yes, gum treatment can sometimes make your teeth feel slightly loose temporarily, particularly after deep cleaning or periodontal surgery. This does not necessarily mean that the treatment has damaged your teeth. When inflamed and swollen gums are cleaned and begin to heal, the gums can shrink back to a healthier state, and teeth may temporarily feel different or slightly more mobile. NHS guidance specifically notes that temporary increases in tooth mobility can occur after periodontal treatment.**<br>**It is also important to remember that gum disease itself can cause loose teeth. Periodontitis can gradually destroy the bone and tissues that support the teeth, so a tooth that already has significant bone loss may feel loose before treatment. In this situation, the treatment is intended to control the disease and protect the remaining support rather than cause the looseness.**<br>**Therefore, a small amount of temporary looseness after gum treatment can be normal, but significant or increasing looseness should be checked by a dentist or periodontist. If a tooth becomes very loose, painful, or seems to be changing position, you should contact your dental professional promptly.**<br>**REFERENCES**:<br>**1-**Kerry GJ, Morrison EC, Ramfjord SP, Hill RW, Caffesse RG, Nissle RR, Appleberry EA. Effect of periodontal treatment on tooth mobility. *J Periodontol.* 1982;53(10):635-638. doi:10.1902/jop.1982.53.10.635.<br>**2-**Chatzopoulos GS, Wolff LF. Change in tooth mobility following non-surgical periodontal therapy: a retrospective cohort study of clinical outcomes. *J Periodontol.* 2026;97(7):1388-1394. doi:10.1002/jper.70046.
	Q27	Does tartar removal cause more tartar buildup?

		**Response: No, tartar removal does not cause more tartar buildup. Tartar, also called calculus, is hardened dental plaque. When it is professionally removed, the procedure does not make the teeth produce tartar faster or create a condition that causes extra buildup. Instead, removing tartar helps eliminate a surface where plaque can accumulate and can help improve gum health.**<br>**However, tartar can build up again after a professional cleaning because plaque naturally forms on the teeth every day. If plaque is not removed through regular brushing and cleaning between the teeth, it can harden into new tartar. The amount and speed of buildup vary from person to person, partly because saliva contains minerals that contribute to calculus formation.**<br>**Therefore, if you notice tartar returning after a scaling, it does not mean that the scaling caused it. It means that new plaque has gradually hardened. Brushing twice a day with fluoride toothpaste and cleaning between your teeth regularly can reduce plaque and slow down tartar formation, although existing tartar cannot be removed effectively at home and requires professional cleaning.**<br>**REFERENCES**:<br>**1-**Akcalı A, Lang NP. Dental calculus: the calcified biofilm and its role in disease development. *Periodontol 2000.* 2018;76(1):109-115. doi:10.1111/prd.12151.<br>**2-**Chapple ILC, Van der Weijden F, Doerfer C, et al. Primary prevention of periodontitis: managing gingivitis. *J Clin Periodontol.* 2015;42(Suppl 16):S71-S76. doi:10.1111/jcpe.12366.
	Q28	Are antibiotics enough to treat gum disease?<br>**Response: No, antibiotics alone are generally not enough to treat gum disease, especially periodontitis. Gum disease is primarily associated with bacterial plaque and the body's inflammatory response to it, so treatment needs to address the plaque and tartar that accumulate around and below the gumline. For periodontitis, professional mechanical cleaning such as scaling and root planing is considered the main treatment, together with good daily oral hygiene.**<br>**Antibiotics can sometimes be used as an additional treatment, but they are not routinely necessary for every patient with gum disease. They may be considered in particular cases, such as certain severe or rapidly progressing periodontal infections or when the disease does not respond adequately to mechanical treatment. The European Federation of Periodontology specifically states that systemic antibiotics should not be used routinely and, when appropriate, should be used as an adjunct rather than as the only treatment.**

		**For example, if someone has periodontitis and takes antibiotics without having the accumulated plaque and tartar professionally removed, the underlying cause of the disease is not adequately addressed. The infection and inflammation can therefore return or continue. Professional cleaning, effective brushing and interdental cleaning, and regular periodontal monitoring are much more important for long-term control.**<br>**Therefore, antibiotics are sometimes useful, but they should not be considered a standalone cure for gum disease. They should only be taken when prescribed by a dentist or doctor for an appropriate reason, because unnecessary antibiotic use can contribute to antibiotic resistance and other adverse effects.**<br>**REFERENCE**:<br>**1-**Teughels W, Feres M, Oud V, Martín C, Matesanz P, Herrera D. Adjunctive effect of systemic antimicrobials in periodontitis therapy: a systematic review and meta-analysis. J Clin Periodontol. 2020;47(Suppl 22):257-281. doi:10.1111/jcpe.13264.
	Q29	After gum disease treatment, will I ever need treatment again?<br>**Response: Yes, you may need further gum treatment after your initial treatment, but this does not mean that the first treatment has failed. Gum disease, particularly periodontitis, is a chronic condition that can be controlled but may return if plaque and inflammation build up again. After active treatment, dentists generally recommend supportive periodontal care, which includes regular check-ups, professional cleaning when necessary, and maintaining good oral hygiene at home. For some people, maintenance visits may be enough to keep the gums healthy and stable for many years. The European Federation of Periodontology recommends that these follow-up appointments be scheduled according to the person's individual risk, with intervals generally ranging from 3 to 12 months. People with a higher risk of recurrence may need more frequent appointments.**<br>**However, some patients may need additional active treatment later if signs of periodontitis return or continue. For example, persistent deep gum pockets, bleeding, or further loss of supporting bone may require another round of professional cleaning or, in more advanced cases, periodontal surgery.**<br>**The good news is that successful treatment can usually halt the progression of periodontitis, especially when you maintain good oral hygiene and attend regular follow-up appointments. So, rather than thinking of gum treatment as something**

		**you do once and never need again, it is better to think of it as initial treatment followed by long-term maintenance.**<br>**REFERENCE**:<br>**1-**Sanz M, Herrera D, Kebschull M, et al. Treatment of stage I–III periodontitis—The EFP S3 level clinical practice guideline. *J Clin Periodontol.* 2020;47(Suppl 22):4-60. doi:10.1111/jcpe.13290.<br>**2-**Rattu V, Raindi D, Antonoglou G, Nibali L. Prevalence of stable and successfully treated periodontitis subjects and incidence of subsequent tooth loss within supportive periodontal care: a systematic review with meta-analyses. *J Clin Periodontol.* 2023;50(10):1371-1389. doi:10.1111/jcpe.13835.
	Q30	Is surgery always necessary for gum treatment?<br>**Response: No, surgery is not always necessary for gum treatment. The treatment depends on the type and severity of the gum disease. In many cases, especially during the early stages, gum disease can be managed without surgery through improved oral hygiene and professional removal of plaque and tartar. For gingivitis, which is the early and reversible form of gum disease, improving daily plaque control and receiving professional cleaning when necessary is usually sufficient.**<br>**For periodontitis, treatment often begins with non-surgical methods such as professional cleaning below the gumline, commonly called scaling and root planing, combined with good oral hygiene at home. The American Dental Association recommends nonsurgical periodontal treatment as an important initial approach for patients with periodontitis.**<br>**However, some advanced cases may require surgery. If deep gum pockets remain after nonsurgical treatment, or if significant bone or tissue damage has occurred, a periodontist may recommend periodontal surgery to access difficult-to-clean areas or to regenerate lost supporting tissues. Surgery is therefore generally considered when less invasive treatment has not achieved adequate control of the disease.**<br>**So, having gum disease does not automatically mean that you need surgery. Many people can manage their condition with professional non-surgical treatment, good oral hygiene, and regular maintenance. The need for surgery depends on the severity of the disease and how well the gums respond to initial treatment.**<br>**REFERENCE**:<br>**1-**Sanz M, Herrera D, Kebschull M, et al. Treatment of stage I–III periodontitis—The EFP S3 level clinical practice guideline. *J Clin Periodontol.* 2020;47(Suppl 22):4-60. doi:10.1111/jcpe.13290.
