# Supplementary material for: Comparative Evaluation of Large Language Models in Responding to Common Misconceptions Regarding Periodontal Disease and Oral Hygiene
Source: Diagnostics (Basel). 2026 Sep 28;16(19):3158. doi: 10.3390/diagnostics16193158 (PMC13664004; doi:10.3390/diagnostics16193158)

Source	Item ID	Question/Statement	Initial Relevance Assessment	Secondary Screening / Exclusion Code	Question Development Pool
**Gholami, M., A. Pakdaman, and J.I. Virtanen, Common perceptions of periodontal health and illness among adults: a qualitative study. Int. Sch. Res. Notices, 2012. 2012(1): p. 671879.doi.org/10.5402/2012/671879**	001	When a tooth is decayed and becomes yellow and black, it can affect one's gums (a woman aged 35+).	**Excluded**	—	—
	002	My teeth became loose because of ageing, and I've got spaces between my teeth resulting in food impaction. I try to remove food debris by all accessible means such as with a strand of plastic or a thread, only causing injury and infection of my gums (a man aged 35+).	**Retained**	**Excluded – A**	—
	003	In my opinion, hereditary factors and tooth "tissue" are very important. My husband never pays attention to his oral hygiene, he smokes too and doesn't use dental floss, but he still has healthy teeth and gums. In comparison, even though I take care of my teeth and gums more than he does, most of my teeth have been restored with crowns (a young woman).	**Retained**	**Retained**	**Included**

	004	Regular and correct tooth brushing, three times a day or at least once at night, is necessary to prevent gum disease (a young man).	**Retained**	**Retained**	**Included**
	005	When food debris such as meat pieces sticks between the teeth, we use dental floss to remove it (a young woman).	**Retained**	**Excluded – A**	—
	006	Usually we go to visit a dentist when we face oral problems such as pain. Dentists recommend a regular dental visit every 4 to 6 months, but we cannot follow that due to several daily problems (a young woman).	**Retained**	**Retained**	**Included**
	007	Regarding the individuals with bad oral odour, it's clear they consider it as of no value for themselves and other people who have to tolerate them (a young woman).	**Retained**	**Excluded – A**	—
	008	Children pay more attention to aesthetics when they grow up. My daughter has noticeably large teeth, and when I mentioned it to her, she decided to brush them regularly (a young woman).	**Excluded**	—	—
	009	We should make a delay between drinking of hot tea and cold water because this intermittent	**Retained**	**Retained**	**Included**

		consumption of cold and hot foods can cause gum and tooth problems (a man aged 35+).			
	010	When we forget brushing teeth, for example when travelling, we can eat an apple or carrot before bed, which can clean the teeth sufficiently (a woman aged 35+).	**Retained**	**Excluded – B**	—
	011	I use a herbaceous drug. I boil sumac and gargle its extract and rub it on my gums. It has a great effect in relieving bleeding and swelling of gums (a women aged 35+).	**Retained**	**Retained**	**Included**
**Dagar, J., et al., Dental Myths and Taboos: Hurdles to Oral Health. IHRJ, 2019. 25(3): p. 5.doi.org/10.26440/IHRJ/0305.08276**	012	Extraction of teeth, especially upper impairs and adversely affects the eye sight and vision. There is no such detailed fact in literature. Sometimes, extraction becomes mandatory but it has nothing to do with eyesight.	**Excluded**	—	—
	013	There is no need to visit dentist for treating milk teeth as they need not be cared for because they don't last long and these will ultimately be replaced by permanent teeth. Early loss of primary teeth will lead to difficulty chewing and will affect the child's nutritional intake. It might also result in drifting of the adjacent teeth causing the	**Excluded**	—	—

		permanent teeth to erupt in irregular position ultimately resulting in crowding.			
	014	Spacing between teeth is a sign of good luck. Spacing has nothing to do with destiny. It results in social stigma affecting the oral health related quality of life.	**Excluded**	—	—
	015	Swelling caused by painful tooth should be fomented with hot water. On the contrary, this procedure increases swelling.	**Excluded**	—	—
	016	Cleaning teeth with fingers is better than cleaning with a toothbrush. Toothbrush bristles can easily intervene the interdental areas and other regions of dentition and oral cavity which cannot be reached by fingers.	**Retained**	**Retained**	**Included**
	017	Abrasives like charcoal, salt and tobacco powder are better than toothpaste for cleaning teeth. These kind of materials abrade the tooth surface leading to sensitivity and even fractures.	**Retained**	**Retained**	**Included**

	018	White teeth are stronger and harder brushing can help achieving it. Every person has got a different shade of teeth mainly depending on the mineral and organic content.	**Excluded**	—	—
	019	It is not good to get dental treatment during pregnancy. Second trimester is safe for a few dental procedures.	**Excluded**	—	—
	020	Old and male dentists are more expert than younger ones and their female counterparts. Expertise does not depend on age and gender.	**Excluded**	—	—
	021	Oral cancer is related to past sins. Oral cancer is multifactorial with genetics, tobacco use, sharp tooth edge, spices and many more.	**Excluded**	—	—
**Abalkhail, A.A. and M.M. Ahmed, Multinationality mapping of public misconceptions about periodontal diseases and their public health implications. Front. Public Health, 2026. 14: p. 1695554.doi.org/10.3389/fpubh.2026.1695554**	022	For healthy gums, teeth should be brushed after every meal.	**Retained**	**Retained**	**Included**
	023	Gum disease will not occur if oral hygiene is good.	**Retained**	**Retained**	**Included**
	024	When oral hygiene is good, no need to get gums checked.	**Retained**	**Retained**	**Included**
	025	Ageing by itself causes teeth to fall out.	**Retained**	**Retained**	**Included**
	026	Gum disease does not cause serious oral health issues	**Retained**	**Excluded – B**	—
	027	No pain means no gum disease.	**Retained**	**Retained**	**Included**

	028	Only in elderly age can gum problems occur.	**Retained**	**Retained**	**Included**
	029	Just the mouth and teeth are affected by gum diseases	**Retained**	**Retained**	**Included**
	030	Children do not get gum problems.	**Retained**	**Retained**	**Included**
	031	Gum problems usually go away on their own.	**Retained**	**Retained**	**Included**
	032	Gum disease can be cured solely by taking medicines.	**Retained**	**Retained**	**Included**
	033	Gum diseases treatment hurts a lot.	**Retained**	**Excluded – B**	—
	034	Teeth become loose when they are cleaned by dentist (teeth scaling).	**Retained**	**Retained**	**Included**
	035	Scaling (teeth cleaning) the teeth makes spaces between the teeth.	**Retained**	**Retained**	**Included**
	036	Gum treatment affects my eye sight.	**Retained**	**Retained**	**Included**
	037	Gum treatment should not be done during pregnancy.	**Retained**	**Retained**	**Included**
**Gowdar, I.M., et al., Oral health myths among general population at Riyadh region, Saudi Arabia. J. Pharm. Bioallied Sci., 2021. 13(Suppl 1): p. S241-S245.doi.org/10.4103/jpbs.JPBS_700_20**	038	If there is no pain in the tooth no need of visiting a dentist.	**Excluded**	—	—
	039	Dental infection will not spread among siblings.	**Excluded**	—	—
	040	Not to worry about milk teeth as they will eventually fall out with time.	**Excluded**	—	—
	041	Exfoliated tooth should be buried.	**Excluded**	—	—

	042	A child born with a tooth is a sign of bad luck in the family.	**Excluded**	—	—
	043	Scaling will weaken the tooth structure.	**Retained**	**Excluded – B**	—
	044	Extraction of teeth will affect eye vision.	**Excluded**	—	—
	045	Pregnant ladies were not supposed to take dental treatment until delivery.	**Excluded**	—	—
	046	Extraction of upper teeth will affect the brain.	**Excluded**	—	—
	047	Placing tobacco over painful tooth will reduce pain.	**Excluded**	—	—
	048	Extracted teeth need no replacement with an artificial teeth.	**Excluded**	—	—
	049	We should not eat anything when we are going for tooth extraction.	**Excluded**	—	—
	050	Brushing your teeth using salt, whitens your teeth.	**Retained**	**Excluded – B**	—
	051	More you brush using hard bristles, more whiter your teeth becomes.	**Retained**	**Retained**	**Included**
	052	Eruption of the third molar will increase wisdom.	**Excluded**	—	—
**Sharma, R., et al., Dental myth, fallacies and misconceptions and its association with socio-dental impact locus of control scale.**	053	Dental decay occurs because my teeth are soft	**Excluded**	—	—
	054	Decay in milk teeth requires no treatment as they are going to fall anyways	**Excluded**	—	—

**Int. j. preventive public health sci., 2015. 1(2): p. 14-20.doi.org/10.17354/ijpphs/2015/11**	055	Placing cloves in a decayed tooth always relieves pain	**Excluded**	—	—
	056	Any tooth pain due to decay, it is better to extract rather than saving	**Excluded**	—	—
	057	Swelling caused by painful tooth should be fomented with hot water	**Excluded**	—	—
	058	Chewing of pan is good for oral health	**Retained**	**Retained**	**Included**
	059	Only aged people get cancer in the mouth	**Excluded**	—	—
	060	Chewing of pan improves bowel movement after heavy meal	**Excluded**	—	—
	061	Betel nut quid chewing with slaked lime can keep gum problems away	**Retained**	**Retained**	**Included**
	062	All dental treatments are painful	**Retained**	**Excluded – B**	—
	063	If I am not in pain I do not need to visit the dentist	**Retained**	**Retained**	**Included**
	064	Extraction of teeth of upper jaw causes loss of vision	**Excluded**	—	—
	065	A tooth once treated does not require any more treatment	**Retained**	**Retained**	**Included**
	066	Cleaning of teeth by a dentist causes loosening of teeth	**Retained**	**Excluded – B**	—
	067	Bleeding of gums is normal during brushing	**Retained**	**Retained**	**Included**

	068	More you brush using hard bristles, whiter your teeth becomes	**Retained**	**Excluded – B**	—
	069	Who is responsible for keeping my teeth healthy?	**Excluded**	—	—
	070	If I have good oral health, who should get the credit?	**Excluded**	—	—
	071	If I get tooth decay or ache, who is to blame?	**Excluded**	—	—
	072	If I have missing teeth, or sensitive teeth or bleeding/swollen gums, who is to blame?	**Retained**	**Excluded – A**	—
	073	If I have difficulty in eating, speaking, chewing and enjoying food because of problems with my teeth or mouth, who is to blame?	**Excluded**	—	—
	074	If I have difficulty in smiling, mixing with friends or indulging in social activities because of problems with my teeth, who is to blame?	**Excluded**	—	—
	075	If people make fun of me because of the condition of my teeth and mouth, who is to blame?	**Excluded**	—	—
**Karakış Akcan, S., et al., Evaluation of ChatGPT as a Source of Patient-Oriented Information on Gingival Recession.**	076	What is gingival recession?	**Excluded**	—	—
	077	Why does the gum move upward?	**Excluded**	—	—
	078	Does gingival recession mean tooth loss?	**Excluded**	—	—
	079	Is gingival recession a disease?	**Retained**	**Retained**	**Included**

**Healthcare (Basel), 2026. 14(10): p. 1339.doi.org/10.3390/healthcare14101339**	080	Is gingival recession related to aging?	**Retained**	**Excluded – B**	—
	081	Does gingival recession occur only in older people?	**Retained**	**Excluded – B**	—
	082	Can gingival recession be stopped?	**Retained**	**Excluded – C**	—
	083	Can gingival recession be reversed?	**Retained**	**Excluded – C**	—
	084	Is gingival recession the same as bone loss?	**Retained**	**Retained**	**Included**
	085	How can gingival recession be noticed?	**Excluded**	—	—
	086	Is gingival recession an aesthetic problem?	**Excluded**	—	—
	087	Does everyone with gingival recession always need treatment?	**Retained**	**Retained**	**Included**
	088	How long does it take for gingival recession to develop?	**Excluded**	—	—
	089	Can gingival recession occur in children?	**Retained**	**Excluded – D**	—
	090	Does everyone with gingival recession need surgery?	**Excluded**	—	—
	091	Can tooth brushing cause gingival recession?	**Retained**	**Excluded – C**	—
	092	Does using a hard toothbrush lead to gingival recession?	**Retained**	**Retained**	**Included**
	093	Is brushing teeth too frequently harmful?	**Retained**	**Excluded – B**	—

	094	Can incorrect brushing technique cause gingival recession?	**Retained**	**Excluded – C**	—
	095	Does clenching or grinding teeth affect gingival recession?	**Retained**	**Excluded – C**	—
	096	Can dental calculus cause gingival recession?	**Retained**	**Excluded – C**	—
	097	Can gingivitis lead to gingival recession over time?	**Retained**	**Excluded – C**	—
	098	Does periodontitis cause gingival recession?	**Retained**	**Excluded – C**	—
	099	Do genetic factors affect gingival recession?	**Retained**	**Excluded – C**	—
	100	Does smoking accelerate gingival recession?	**Retained**	**Excluded – C**	—
	101	Can gingival recession occur after orthodontic treatment?	**Retained**	**Excluded – C**	—
	102	Does gingival recession increase with age?	**Retained**	**Excluded – C**	—
	103	Can gingival recession result from poor oral hygiene?	**Retained**	**Excluded – C**	—
	104	Does diet influence gingival recession?	**Retained**	**Excluded – C**	—
	105	Do systemic diseases affect gingival recession?	**Retained**	**Excluded – C**	—
	106	Can removable dentures cause gingival recession?	**Retained**	**Excluded – C**	—

	107	Can faulty fillings or crowns cause gingival recession?	**Excluded**	—	—
	108	Can scaling cause gingival recession?	**Retained**	**Excluded – C**	—
	109	After scaling my teeth look longer—is this recession?	**Retained**	**Retained**	**Included**
	110	Does gingival recession increase after scaling?	**Retained**	**Excluded – C**	—
	111	Is it possible to prevent gingival recession?	**Retained**	**Excluded – C**	—
	112	How should I brush my teeth to prevent gingival recession?	**Retained**	**Excluded – C**	—
	113	Do electric toothbrushes help prevent gingival recession?	**Retained**	**Excluded – C**	—
	114	Which toothbrush is more suitable for preventing gingival recession?	**Retained**	**Excluded – C**	—
	115	Which toothpastes are better for gum health?	**Retained**	**Excluded – C**	—
	116	Does using an oral irrigator prevent gingival recession?	**Retained**	**Excluded – C**	—
	117	Does dental floss harm or benefit the gums?	**Retained**	**Retained**	**Included**
	118	Does regular scaling prevent gingival recession?	**Retained**	**Excluded – C**	—
	119	Do dietary habits affect gingival recession?	**Retained**	**Excluded – C**	—

	120	Does quitting smoking slow gingival recession?	**Retained**	**Excluded – C**	—
	121	Does using a night guard help prevent gingival recession?	**Retained**	**Excluded – C**	—
	122	What should I do when I notice gingival recession?	**Excluded**	—	—
	123	Does regular dental check-up prevent gingival recession?	**Retained**	**Excluded – C**	—
	124	If there is a family history of gingival recession, can early prevention help?	**Retained**	**Excluded – C**	—
	125	Is early detection of gingival recession advantageous?	**Retained**	**Excluded – C**	—
	126	Does gingival recession cause pain?	**Retained**	**Excluded – C**	—
	127	Does gingival recession cause tooth sensitivity?	**Retained**	**Excluded – C**	—
	128	Does gingival recession cause discomfort with hot or cold foods?	**Retained**	**Excluded – C**	—
	129	Can gingival recession cause bad breath?	**Retained**	**Excluded – C**	—
	130	Does gingival recession affect appearance?	**Excluded**	—	—
	131	Does gingival recession affect my smile?	**Excluded**	—	—
	132	Does gingival recession affect speech?	**Excluded**	—	—

	133	Does food accumulate in areas with gingival recession?	**Retained**	**Excluded – C**	—
	134	Does gingival recession cause gaps between teeth?	**Retained**	**Excluded – C**	—
	135	Does gingival recession affect chewing function?	**Retained**	**Excluded – C**	—
	136	Does gingival recession affect daily life?	**Excluded**	—	—
	137	Does gingival recession affect self-confidence?	**Excluded**	—	—
	138	Does gingival recession affect social life?	**Excluded**	—	—
	139	Should I worry about tooth loss due to gingival recession?	**Excluded**	—	—
	140	Can gingival recession heal on its own?	**Excluded**	—	—
	141	How can I tell if gingival recession is progressing?	**Excluded**	—	—
	142	What treatment options are available for gingival recession?	**Excluded**	—	—
	143	Can gingival recession be treated with laser therapy?	**Excluded**	—	—
	144	What does grafting mean in gingival recession treatment?	**Excluded**	—	—
	145	Is graft surgery necessary for every recession?	**Excluded**	—	—
	146	Can fillings be used to treat gingival recession?	**Excluded**	—	—

	147	Can pink esthetic procedures correct gingival recession?	**Excluded**	—	—
	148	Is crown treatment appropriate for gingival recession?	**Excluded**	—	—
	149	In which cases is non-surgical treatment sufficient?	**Excluded**	—	—
	150	Is flap surgery performed for gingival recession?	**Excluded**	—	—
	151	Is root surface debridement effective for gingival recession?	**Excluded**	—	—
	152	Is graft treatment painful?	**Excluded**	—	—
	153	Can composite restorations be placed in areas with gingival recession?	**Excluded**	—	—
	154	Which types of grafts are used for gingival recession?	**Excluded**	—	—
	155	What is the difference between free gingival grafts and connective tissue grafts?	**Excluded**	—	—
	156	Is additional treatment required after surgery?	**Excluded**	—	—
	157	What happens if graft surgery fails?	**Excluded**	—	—
	158	Are there treatment options other than surgery?	**Excluded**	—	—
	159	Can biomaterials such as PRF or CGF be used?	**Excluded**	—	—

	160	Should laser or graft treatment be preferred for gingival recession?	**Excluded**	—	—
	161	Are sutures placed after graft surgery?	**Excluded**	—	—
	162	When are sutures removed?	**Excluded**	—	—
	163	Can gingival recession recur after surgery?	**Excluded**	—	—
	164	Can graft surgery be repeated if necessary?	**Excluded**	—	—
	165	What should I pay attention to during the healing period?	**Excluded**	—	—
	166	Is preparation required before graft surgery?	**Excluded**	—	—
	167	How long does graft surgery take?	**Excluded**	—	—
	168	Is hospitalization required for graft surgery?	**Excluded**	—	—
	169	Is the procedure performed under local anesthesia?	**Excluded**	—	—
	170	Which dental specialist should I consult for gingival recession?	**Excluded**	—	—
	171	Will the appearance of my teeth improve after treatment?	**Excluded**	—	—
	172	Does smoking affect graft success?	**Excluded**	—	—
	173	Is gingival recession treatment expensive?	**Excluded**	—	—
	174	Is graft treatment covered by social security insurance?	**Excluded**	—	—
	175	Can grafts be performed on multiple areas at the same time?	**Excluded**	—	—

	176	When should I return for follow-up after graft surgery?	**Excluded**	—	—
	177	Does every patient with gingival recession require surgical treatment?	**Excluded**	—	—
	178	Is treatment necessary for mild gingival recession?	**Excluded**	—	—
	179	What happens if the recession level is not suitable for treatment?	**Excluded**	—	—
	180	Is tooth sensitivity required to perform graft surgery?	**Excluded**	—	—
	181	Can grafting be done for purely esthetic reasons?	**Excluded**	—	—
	182	Can graft surgery be performed in smokers?	**Excluded**	—	—
	183	I have diabetes—can I undergo graft surgery?	**Excluded**	—	—
	184	Can gingival recession be treated during pregnancy?	**Excluded**	—	—
	185	Can graft surgery be performed in patients under 18 years old?	**Excluded**	—	—
	186	Is graft surgery possible in elderly patients?	**Excluded**	—	—
	187	Can graft surgery be performed in patients using psychiatric medications?	**Excluded**	—	—
	188	Can patients taking anticoagulant drugs undergo graft surgery?	**Excluded**	—	—

	189	Can graft surgery be performed if there is a filling in the affected area?	**Excluded**	—	—
	190	Can graft surgery be repeated in the same area?	**Excluded**	—	—
	191	Is general health status important for graft surgery?	**Excluded**	—	—
	192	Can graft surgery be performed around implants?	**Excluded**	—	—
	193	Does graft success vary depending on personal factors?	**Excluded**	—	—
	194	Can patients with limited mouth opening undergo graft surgery?	**Excluded**	—	—
	195	Is allergy testing required before graft surgery?	**Excluded**	—	—
	196	Does graft selection change according to recession depth?	**Excluded**	—	—
	197	Can graft surgery be performed in patients with a history of periodontitis?	**Excluded**	—	—
	198	Is graft surgery possible if bone loss is present?	**Excluded**	—	—
	199	Can graft surgery be performed if periodontal disease is active?	**Excluded**	—	—
	200	Can graft surgery be performed in patients with bruxism?	**Excluded**	—	—

	201	Can patients with severe gag reflex undergo graft surgery?	**Excluded**	—	—
	202	Can graft surgery be performed in patients with poor oral hygiene?	**Excluded**	—	—
	203	Can graft surgery be performed during orthodontic treatment?	**Excluded**	—	—
	204	Can grafts be performed in areas with crowns or bridges?	**Excluded**	—	—
	205	Can removable denture users undergo graft surgery?	**Excluded**	—	—
	206	Is graft surgery recommended for anxious patients?	**Excluded**	—	—
	207	Are special precautions required for patients with systemic diseases?	**Excluded**	—	—
	208	Is time off work required after graft surgery?	**Excluded**	—	—
	209	Is scaling mandatory before graft surgery?	**Excluded**	—	—
	210	Should early-stage gingival recession be monitored before treatment?	**Excluded**	—	—
	211	If gingival recession has stopped progressing, is treatment unnecessary?	**Excluded**	—	—
	212	What is the most common complication after graft surgery?	**Excluded**	—	—
	213	What happens if the graft does not survive?	**Excluded**	—	—

	214	Is bleeding normal after graft surgery?	**Excluded**	—	—
	215	Is there a risk of infection after graft surgery?	**Excluded**	—	—
	216	How long does pain last after graft surgery?	**Excluded**	—	—
	217	Is bruising around the graft area dangerous?	**Excluded**	—	—
	218	What happens if graft sutures open?	**Excluded**	—	—
	219	Is it a problem if sutures are not placed after graft surgery?	**Excluded**	—	—
	220	Can graft surgery cause poor esthetic outcomes?	**Excluded**	—	—
	221	Can graft surgery be repeated if unsuccessful?	**Excluded**	—	—
	222	Do lip movements affect graft healing?	**Excluded**	—	—
	223	Do jaw movements affect graft healing?	**Excluded**	—	—
	224	Can numbness occur after graft surgery?	**Excluded**	—	—
	225	Is tissue difference felt after graft surgery?	**Excluded**	—	—
	226	Is tissue hardness after graft surgery temporary?	**Excluded**	—	—
	227	Is recurrence of recession normal after graft surgery?	**Excluded**	—	—
	228	Is permanent color difference possible after graft surgery?	**Excluded**	—	—
	229	Can gingival recession accelerate after graft surgery?	**Excluded**	—	—
	230	Can tooth mobility occur after graft surgery?	**Excluded**	—	—

	231	Can cold sensitivity develop after graft surgery?	**Excluded**	—	—
	232	Is facial swelling normal after graft surgery?	**Excluded**	—	—
	233	How long does gingival swelling last after surgery?	**Excluded**	—	—
	234	Can tissue breakdown occur after graft surgery?	**Excluded**	—	—
	235	Can brushing cause tissue loss after graft surgery?	**Excluded**	—	—
	236	When can graft success be evaluated?	**Excluded**	—	—
	237	Will esthetic irregularities resolve over time?	**Excluded**	—	—
	238	What should be done if recession recurs after graft surgery?	**Excluded**	—	—
	239	Can tissue sagging occur after graft surgery?	**Excluded**	—	—
	240	Can bad breath occur after graft surgery?	**Excluded**	—	—
	241	How should the graft area be cleaned postoperatively?	**Excluded**	—	—
	242	Does smoking cause graft failure?	**Excluded**	—	—
	243	Can numbness persist after graft surgery?	**Excluded**	—	—
	244	Can saliva damage sutures?	**Excluded**	—	—
	245	Is a white layer over the graft area normal?	**Excluded**	—	—
	246	Is food impaction in the graft area problematic?	**Excluded**	—	—

	247	When can repeat surgery be performed if graft fails?	**Excluded**	—	—
	248	Should gingival recession be treated before crown placement?	**Excluded**	—	—
	249	Does graft surgery damage crowned teeth?	**Excluded**	—	—
	250	When can crowns be placed after graft surgery?	**Excluded**	—	—
	251	Can graft surgery be performed for recession under a crown?	**Excluded**	—	—
	252	How long should one wait before taking impressions after graft surgery?	**Excluded**	—	—
	253	Does placing crowns without grafting affect long-term success?	**Excluded**	—	—
	254	Are existing crowns replaced after graft surgery?	**Excluded**	—	—
	255	What happens if the crown margin pressures the graft area?	**Excluded**	—	—
	256	Should I be careful while eating with my prosthesis after graft surgery?	**Excluded**	—	—
	257	Is prosthesis re-adjusted after graft surgery?	**Excluded**	—	—
	258	Is using a temporary prosthesis harmful after graft surgery?	**Excluded**	—	—

	259	What should be done if the crown does not fit the gingiva after surgery?	**Excluded**	—	—
	260	Does grafting over prosthetic areas improve esthetics?	**Excluded**	—	—
	261	How long does pain last after surgery?	**Excluded**	—	—
	262	Is swelling normal after graft surgery?	**Excluded**	—	—
	263	What should I do if bleeding occurs?	**Excluded**	—	—
	264	Is ice application necessary after surgery?	**Excluded**	—	—
	265	Should hot foods be avoided on the first day?	**Excluded**	—	—
	266	Does chewing on the graft area cause damage?	**Excluded**	—	—
	267	Is tooth brushing harmful in the early postoperative period?	**Excluded**	—	—
	268	When are sutures removed after surgery?	**Excluded**	—	—
	269	Should the graft area be brushed?	**Excluded**	—	—
	270	Does brushing sutures harm graft healing?	**Excluded**	—	—
	271	Should mouthwash be used after surgery?	**Excluded**	—	—
	272	Does spitting blood affect graft success?	**Excluded**	—	—
	273	Does smoking impair graft healing?	**Excluded**	—	—
	274	Are antibiotics necessary after graft surgery?	**Excluded**	—	—
	275	How long should painkillers be used?	**Excluded**	—	—
	276	What foods can I eat after surgery?	**Excluded**	—	—
	277	Is soft diet required during the first week?	**Excluded**	—	—
	278	What happens if the graft area is touched?	**Excluded**	—	—

	279	Can I exercise during the first week?	**Excluded**	—	—
	280	When can mouth rinsing begin?	**Excluded**	—	—
	281	What should I do if the graft moves?	**Excluded**	—	—
	282	Does a fallen graft require repeat surgery?	**Excluded**	—	—
	283	How should sutured areas be protected?	**Excluded**	—	—
	284	Is excessive talking harmful after surgery?	**Excluded**	—	—
	285	Is fever normal after graft surgery?	**Excluded**	—	—
	286	How long does complete healing take after graft surgery?	**Excluded**	—	—
	287	Is color change in the graft area normal?	**Excluded**	—	—
	288	Is graft shrinkage normal after one month?	**Excluded**	—	—
	289	Is white appearance of the graft area problematic?	**Excluded**	—	—
	290	What should be done if recession persists after two months?	**Excluded**	—	—
	291	Is persistent sensitivity a sign of graft failure?	**Excluded**	—	—
	292	Is color mismatch between graft and gingiva normal?	**Excluded**	—	—
	293	Does the graft adapt to surrounding tissue over time?	**Excluded**	—	—
	294	Is hardness of the graft area normal?	**Excluded**	—	—
	295	When does the final appearance stabilize?	**Excluded**	—	—
	296	How can I tell if the graft has failed?	**Excluded**	—	—

	297	What should be done if recession recurs after six months?	**Excluded**	—	—
	298	Is long-term recurrence possible after graft surgery?	**Excluded**	—	—
	299	Can esthetic corrections be made if the result is unsatisfactory?	**Excluded**	—	—
	300	Is smile asymmetry permanent after graft surgery?	**Excluded**	—	—
	301	How should brushing be performed after healing?	**Excluded**	—	—
	302	Which toothpastes are recommended after graft surgery?	**Excluded**	—	—
	303	What happens if the graft area is brushed too hard?	**Excluded**	—	—
	304	What is evaluated during follow-up visits?	**Excluded**	—	—
	305	Is feeling graft enlargement normal?	**Excluded**	—	—
	306	How often should follow-up visits be scheduled?	**Excluded**	—	—
	307	Can scaling damage the graft area?	**Excluded**	—	—
	308	Can crowns be placed six months after graft surgery?	**Excluded**	—	—
	309	Can orthodontic treatment be performed over grafted areas?	**Excluded**	—	—

	310	Is tissue thickening after graft surgery beneficial?	**Excluded**	—	—
	311	How does the clinician determine graft adequacy?	**Excluded**	—	—
	312	What happens if gingival recession is left untreated?	**Excluded**	—	—
	313	Can gingival recession stop spontaneously without treatment?	**Excluded**	—	—
	314	Can untreated gingival recession lead to tooth loss?	**Excluded**	—	—
	315	Does untreated gingival recession cause bone loss?	**Excluded**	—	—
	316	Does tooth sensitivity increase if untreated?	**Excluded**	—	—
	317	Does gingival recession become painful over time?	**Excluded**	—	—
	318	Does untreated recession worsen esthetics?	**Excluded**	—	—
	319	Do spaces between teeth increase?	**Excluded**	—	—
	320	Does gingival recession cause bad breath?	**Excluded**	—	—
	321	Can gingival recession affect speech?	**Excluded**	—	—
	322	Does gingival recession impair chewing function?	**Excluded**	—	—
	323	Can untreated recession harm neighboring teeth?	**Excluded**	—	—

	324	Can gingival recession progress to tooth loss?	**Excluded**	—	—
	325	Does untreated recession affect orthodontic treatment?	**Excluded**	—	—
	326	Does gingival recession complicate prosthetic planning?	**Excluded**	—	—
	327	Can gingival recession prevent future implant placement?	**Excluded**	—	—
	328	Does delaying treatment reduce success rates?	**Excluded**	—	—
	329	Does delaying treatment increase costs?	**Excluded**	—	—
	330	Does early intervention simplify treatment?	**Excluded**	—	—
	331	Can untreated gingival recession have systemic effects?	**Excluded**	—	—
	332	Does gingival recession cause psychological distress?	**Excluded**	—	—
	333	Does gingival recession affect facial esthetics?	**Excluded**	—	—
	334	Is it normal for gingival recession to affect self-confidence?	**Excluded**	—	—
	335	Do individuals with gingival recession avoid social settings?	**Excluded**	—	—
	336	Do esthetic concerns influence treatment decisions?	**Excluded**	—	—
	337	Do patients cover their mouth while smiling due to recession?	**Excluded**	—	—

	338	Does gingival recession cause anxiety during speaking?	**Excluded**	—	—
	339	Does gingival recession affect work performance?	**Excluded**	—	—
	340	Does gingival recession alter facial expression?	**Excluded**	—	—
	341	Can gingival recession affect mood? Are individuals with gingival recession more prone to depression?	**Excluded**	—	—
	342	Does gingival recession cause shyness in relationships?	**Excluded**	—	—
	343	Does gingival recession have greater psychological impact in younger individuals?	**Excluded**	—	—
	344	Is smile design reasonable for gingival recession?	**Excluded**	—	—
	345	Does social media increase awareness of gingival esthetics?	**Excluded**	—	—
	346	Does gingival recession increase interest in esthetic dentistry?	**Excluded**	—	—
	347	Does self-confidence improve after gingival treatment?	**Excluded**	—	—
**Periodontology, A.A.o. American Academy of Periodontology for-patients FAQs 2026**	348	What are common signs and symptoms of periodontal disease?	**Excluded**	—	—

**[cited 2026 May 25, 2026]; Available from: https://www.perio.org/for-patients/faqs/#1558537292986-94d377d6-47b0.**	349	I have heard there is a connection between gum disease and other diseases. Is this true? Where can I find more information?	**Retained**	**Retained**	**Included**
	350	My periodontist informed me that I need implants, but I can't afford them at this time. Is there any financial assistance for periodontal treatments?	**Excluded**	—	—
	351	I lost a tooth, and I'm interested in replacing it with an implant. How much does the average implant cost?	**Excluded**	—	—
	352	What is the difference between plaque and calculus?	**Excluded**	—	—
	353	Can children be at risk for developing periodontal disease?	**Retained**	**Excluded – D**	—
	354	Other than diagnose and treat gum disease, what else have periodontists been trained to do?	**Excluded**	—	—
	355	My periodontist says that he is "board certified." What does that mean?	**Excluded**	—	—
	356	Who should treat my periodontal disease: my general dentist or a periodontist?	**Excluded**	—	—
	357	What can I do at home to prevent periodontal disease?	**Excluded**	—	—

	358	Both of my parents have periodontal disease, and I'm worried that it may be genetic. Is there a way to determine my risk for developing gum disease?	**Retained**	**Excluded – B**	—
	359	My dentist told me that I have signs of periodontal disease and that I should see a periodontist for a consultation. How can I find a periodontist in my area?	**Excluded**	—	—
	360	Is periodontal disease contagious?	**Retained**	**Retained**	**Included**
	361	I was recently diagnosed with periodontal disease. How often should I see my periodontist for an examination?	**Excluded**	—	—
	362	I have big gums and short teeth, so when I smile you can almost only see my gums. I don't smile very often anymore because I am so self conscious of my gums. Is there a way to improve my smile?	**Retained**	**Excluded – A**	—
	363	What are the consequences of missing teeth?	**Excluded**	—	—
**Periodontology, E.F.o. European Federation of Periodontology gum diseases faqs. 2026; Available from: https://www.efp.org/for-patients/gum-diseases/faqs/.**	364	What is gum disease?	**Excluded**	—	—
	365	What is gingivitis?	**Excluded**	—	—
	366	What is periodontitis?	**Excluded**	—	—
	367	What are the symptoms of periodontitis?	**Excluded**	—	—

	368	What is the difference between gingivitis and periodontitis?	**Excluded**	—	—
	369	My gums are receding: does that mean I have periodontitis?	**Excluded**	—	—
	370	What are the causes of periodontitis?	**Excluded**	—	—
	371	What are the consequences of periodontal disease?	**Excluded**	—	—
	372	What are the risk factors for periodontitis?	**Excluded**	—	—
	373	What can I do to prevent periodontal disease?	**Excluded**	—	—
	374	How is periodontitis diagnosed?	**Excluded**	—	—
	375	How is periodontitis treated?	**Excluded**	—	—
	376	What is periodontology?	**Excluded**	—	—
	377	What is a periodontist?	**Excluded**	—	—
	378	What are the links between periodontitis and other diseases?	**Retained**	**Retained**	**Included**
**Babayiğit, O., et al., Potential use of ChatGPT for patient information in periodontology: a descriptive pilot study. Cureus, 2023. 15(11).doi.org/10.7759/cureus.48518**	379	What causes tooth sensitivity?	**Excluded**	—	—
	380	What can I do to prevent tooth sensitivity?	**Excluded**	—	—
	381	How is tooth sensitivity treated?	**Excluded**	—	—
	382	Can tooth sensitivity be a sign of a more serious dental problem?	**Excluded**	—	—
	383	Can tooth sensitivity be reversed?	**Excluded**	—	—
	384	Can tooth sensitivity be related to gum disease?	**Excluded**	—	—

	385	Are there any home remedies for tooth sensitivity?	**Excluded**	—	—
	386	How often should I see a dentist if I have tooth sensitivity?	**Excluded**	—	—
	387	Can teeth whitening cause tooth sensitivity?	**Excluded**	—	—
	388	Is tooth sensitivity common and how many people experience it?	**Excluded**	—	—
	389	What causes bad breath?	**Excluded**	—	—
	390	How can I prevent bad breath?	**Excluded**	—	—
	391	Is bad breath a sign of a serious medical condition?	**Excluded**	—	—
	392	Can certain foods or drinks cause bad breath?	**Excluded**	—	—
	393	What are some common treatments for bad breath?	**Excluded**	—	—
	394	Should I see a dentist or a doctor for bad breath?	**Excluded**	—	—
	395	Can smoking cause bad breath?	**Excluded**	—	—
	396	Is there a link between gum disease and bad breath?	**Retained**	**Retained**	**Included**
	397	How often should I brush and floss to prevent bad breath?	**Retained**	**Retained**	**Included**
	398	What medications cause bad breath?	**Retained**	**Excluded – C**	—

	399	What is gingival recession?	**Excluded**	—	—
	400	What causes gingival recession?	**Excluded**	—	—
	401	What are the symptoms of gingival recession?	**Excluded**	—	—
	402	Can gingival recession be prevented?	**Retained**	**Excluded – C**	—
	403	Can gingival recession be treated?	**Retained**	**Excluded – C**	—
	404	Is gingival recession reversible?	**Retained**	**Retained**	**Included**
	405	What are the complications of untreated gingival recession?	**Excluded**	—	—
	406	Can brushing too hard cause gingival recession?	**Retained**	**Retained**	**Included**
	407	Are there any home remedies for gingival recession?	**Excluded**	—	—
	408	How often should I visit the dentist to check for gingival recession?	**Excluded**	—	—
	409	What is periodontal disease?	**Excluded**	—	—
	410	What are the symptoms of periodontal disease?	**Excluded**	—	—
	411	How is periodontal disease diagnosed?	**Excluded**	—	—
	412	What causes periodontal disease?	**Excluded**	—	—
	413	Can periodontal disease be prevented?	**Retained**	**Excluded – B**	—
	414	How is periodontal disease treated?	**Excluded**	—	—

	415	Is periodontal disease contagious?	**Retained**	**Excluded – B**	—
	416	How long does it take to treat periodontal disease?	**Excluded**	—	—
	417	What is the difference between gingivitis and periodontitis?	**Excluded**	—	—
	418	What can happen if periodontal disease is left untreated?	**Excluded**	—	—
	419	What are peri-implant diseases?	**Excluded**	—	—
	420	What are the symptoms of peri-implant diseases?	**Excluded**	—	—
	421	How are peri-implant diseases diagnosed?	**Excluded**	—	—
	422	What causes peri-implant diseases?	**Excluded**	—	—
	423	Can peri-implant diseases be prevented?	**Excluded**	—	—
	424	How are peri-implant diseases treated?	**Excluded**	—	—
	425	Can I still have dental implants if I have a history of periodontal disease?	**Excluded**	—	—
	426	Is there a difference between peri-implant mucositis and peri-implantitis?	**Excluded**	—	—
	427	How often should I have my dental implants checked for peri-implant diseases?	**Excluded**	—	—
	428	How can I maintain good oral hygiene to prevent peri-implant diseases?	**Excluded**	—	—

	429	What are dental implants?	**Excluded**	—	—
	430	How long do dental implants last?	**Excluded**	—	—
	431	Are dental implants painful?	**Excluded**	—	—
	432	What is the success rate of dental implants?	**Excluded**	—	—
	433	Can anyone get dental implants?	**Excluded**	—	—
	434	How much do dental implants cost?	**Excluded**	—	—
	435	How long does the dental implant process take?	**Excluded**	—	—
	436	Are dental implants safe?	**Excluded**	—	—
	437	What are the benefits of dental implants?	**Excluded**	—	—
	438	How do I care for my dental implants?	**Excluded**	—	—
	439	What is periodontal surgery?	**Excluded**	—	—
	440	Why do I need periodontal surgery?	**Excluded**	—	—
	441	What types of periodontal surgeries are available?	**Excluded**	—	—
	442	Is periodontal surgery painful?	**Excluded**	—	—
	443	How long does periodontal surgery take?	**Excluded**	—	—
	444	What is the recovery time after periodontal surgery?	**Excluded**	—	—
	445	Will I need to take time off work or school after periodontal surgery?	**Excluded**	—	—
	446	How do I prepare for periodontal surgery?	**Excluded**	—	—
	447	What can I eat after periodontal surgery?	**Excluded**	—	—

	448	How do I take care of my mouth after periodontal surgery?	**Excluded**	—	—
**Zhang, Q., et al., Comprehensiveness of large language models in patient queries on gingival and endodontic health. Int. Dent. J., 2025. 75(1): p. 151-157.doi.org/10.1016/j.identj.2024.06.022**	449	Why do my gums bleed?	**Retained**	**Retained**	**Included**
	450	Why do my gums recede?	**Retained**	**Retained**	**Included**
	451	What causes bad breath?	**Excluded**	—	—
	452	Why are my teeth sensitive to cold?	**Excluded**	—	—
	453	Why do my tooth hurt?	**Excluded**	—	—
	454	What is plaque and why is it harmful?	**Excluded**	—	—
	455	Are sweets really bad for my teeth?	**Excluded**	—	—
	456	What are early signs of dental trouble?	**Excluded**	—	—
	457	What are the symptoms of gum disease?	**Excluded**	—	—
	458	How does my dentist diagnose periodontal disease?	**Excluded**	—	—
	459	What would happen if I have a dental problem while pregnant?	**Excluded**	—	—
	460	What type of surgery is used to treat gum recession?	**Excluded**	—	—
	461	How can I prevent gum recession?	**Retained**	**Retained**	**Included**
	462	What can I do to prevent bad breath?	**Retained**	**Retained**	**Included**
	463	What can I do if my tooth gets knocked out in an accident?	**Excluded**	—	—
	464	My tooth is cracked, how should I proceed?	**Excluded**	—	—
	465	How can gum disease be prevented?	**Excluded**	—	—

	466	How is gum disease treated?	**Excluded**	—	—
	467	How are cavities treated?	**Excluded**	—	—
	468	What tooth paste should I use?	**Excluded**	—	—
	469	How often should I brush my teeth and for how long each time?	**Retained**	**Retained**	**Included**
	470	Are electric toothbrushes better than manual brushes?	**Excluded**	—	—
	471	Is gum disease linked to other health problems?	**Excluded**	—	—
	472	How many times a year should I have my teeth cleaned?	**Excluded**	—	—
	473	How should I care for my teeth and gums?	**Excluded**	—	—
**BinHamdan, R.H., et al., Social media use and oral health–related misconceptions in Saudi Arabia: cross-sectional study. JMIR Form. Res., 2025. 9: p. e70071.doi.org/10.2196/70071**	474	During pregnancy, the baby absorbs calcium from the mother's teeth and bones	**Excluded**	—	—
	475	Pregnant women should only undergo dental treatment after childbirth	**Excluded**	—	—
	476	You should not eat anything when going for tooth extraction	**Excluded**	—	—
	477	Scaling weakens the structure of the teeth	**Retained**	**Excluded – B**	—
	478	Upper teeth extraction affects the brain	**Excluded**	—	—
	479	Brushing with salt helps whiten teeth	**Retained**	**Excluded – B**	—

	480	It is better not to brush your teeth when you have bleeding gums	**Retained**	**Retained**	**Included**
	481	Extracted teeth do not need to be replaced with artificial teeth	**Excluded**	—	—
	482	Taking antibiotics will relieve the tooth pain	**Retained**	**Retained**	**Included**
	483	If there is no tooth pain, there is no need to visit the dentist	**Retained**	**Retained**	**Included**
	484	Leaving a milk bottle in the baby's mouth during sleep does not harm teeth	**Excluded**	—	—
	485	The appearance of wisdom teeth increases one's wisdom	**Excluded**	—	—
	486	Using a hard-bristled toothbrush makes teeth whiter	**Retained**	**Excluded – B**	—
	487	There is no need to care for primary teeth, as they will be replaced by permanent teeth anyway	**Excluded**	—	—
	488	If there's no tooth pain, there's no need to visit the dentist	**Retained**	**Excluded – B**	—
	489	Scaling (cleaning the teeth in the dental clinic by using an electronic device to remove calculus) will weaken the tooth structure	**Retained**	**Excluded – B**	—
	490	The appearance of wisdom teeth increases one's wisdom	**Excluded**	—	—

	491	Cleaning your teeth using a hard bristled toothbrush makes your teeth whiter	**Retained**	**Excluded – B**	—
	492	Brushing your teeth with salt helps to whiten your teeth	**Retained**	**Excluded – B**	—
	493	You should not eat anything when you are going for tooth extraction	**Excluded**	—	—
	494	Extracted teeth do not need to be replaced with artificial teeth	**Excluded**	—	—
	495	Extraction of the upper teeth will affect the brain	**Excluded**	—	—
	496	Pregnant women should only undergo dental treatment after childbirth	**Excluded**	—	—
	497	It is better not to brush your teeth when you have bleeding gums	**Retained**	**Excluded – B**	—
	498	During pregnancy, the baby absorbs calcium from the mother's teeth and bones	**Excluded**	—	—
	499	Leaving a milk bottle in the baby's mouth during sleep does not harm teeth	**Excluded**	—	—
	500	If you have pain in a particular tooth, taking antibiotics will relieve the tooth pain	**Retained**	**Retained**	**Included**
	501	There is no need to care for primary teeth, as they will be replaced by permanent teeth anyway	**Excluded**	—	—

**Pierce, J., et al., Patient self-reported perception and knowledge of periodontal disease: An observational, university-representative, survey-based analysis in an academic setting. J Am Dent Assoc, 2025. 156(3): p. 239-250. e4.doi.org/10.1016/j.adaj.2024.12.011**	502	Periodontitis is a disease of the tooth supporting tissues (gums and bone) surrounding the teeth.	**Excluded**	—	—
	503	I understand what periodontitis does to teeth and gums/bone surrounding teeth.	**Excluded**	—	—
	504	Periodontitis can cause tooth loss.	**Excluded**	—	—
	505	Bacteria is the main cause of periodontitis.	**Excluded**	—	—
	506	Periodontitis only happens to elder individuals.	**Retained**	**Retained**	**Included**
	507	Periodontitis affects a large part of the population (greater than 40% of people).	**Excluded**	—	—
	508	Periodontitis is a chronic disease.	**Excluded**	—	—
	509	Yearly exams (radiographs and dental charting) are important to maintain good periodontal health.	**Excluded**	—	—
	510	I understand what a periodontal pocket (number recorded when taking “probing depths”) means.	**Excluded**	—	—
	511	Periodontitis can impact overall health and has been related to other diseases like heart disease and diabetes.	**Excluded**	—	—
	512	Dental implants can also suffer bone loss from a similar disease.	**Excluded**	—	—
	513	I understand why I need gum treatment.	**Excluded**	—	—

	514	I feel a positive difference in dental health since gum treatment.	**Excluded**	—	—
	515	If I had to decide again, I would get this treatment again.	**Excluded**	—	—
	516	I am comfortable asking my dentist/hygienist questions about my dental health.	**Excluded**	—	—
	517	I get clear and helpful instructions from my dentist/hygienist.	**Excluded**	—	—
	518	My dentist/hygienist is knowledgeable about periodontitis.	**Excluded**	—	—
	519	I would recommend my gum treatment to other people with periodontitis.	**Excluded**	—	—
	520	I feel like the gum treatment is going to improve my overall health.	**Excluded**	—	—
	521	I am satisfied with the current health of my teeth and gums.	**Excluded**	—	—
	522	My time and money put into gum treatment is worthwhile.	**Excluded**	—	—
	523	I am highly motivated to keep up with maintenance cleaning appointment.	**Excluded**	—	—
	524	I maintain higher levels of oral hygiene since receiving gum treatment.	**Excluded**	—	—

	525	I attend appointments recommended by my dentist/hygienist because they are important to my dental health.	**Excluded**	—	—
	526	If I had to continue living with periodontitis, that would be acceptable to me.	**Excluded**	—	—
**Gobika N, G.M., Lalitha Tanjore Arunachalam, Navina Ravindran, Nimisha Mithradas A Study On Myths About Periodontal Disease Among General Public IJCRT, 2025. 13(10)**	527	Bleeding gums while brushing is normal	**Retained**	**Retained**	**Included**
	528	Gum disease affects only older people	**Retained**	**Excluded – B**	—
	529	Bad breath is always due to stomach problems	**Retained**	**Retained**	**Included**
	530	Professional cleaning does not loosen teeth	**Retained**	**Excluded – A**	—
	531	Mouth rinse can permanently cure gum disease	**Retained**	**Retained**	**Included**
	532	Tooth loss is a natural part of ageing	**Retained**	**Retained**	**Included**
	533	Brushing harder cleans teeth better	**Retained**	**Retained**	**Included**
	534	Chewing hard food cleans/strengthens teeth	**Retained**	**Retained**	**Included**
	535	Gum disease is not serious if painless	**Retained**	**Retained**	**Included**
	536	Smoking does not affect gums if brushing regularly	**Retained**	**Retained**	**Included**
	537	Only people with cavities get gum disease	**Excluded**	—	—
	538	Receded gums can grow back naturally	**Retained**	**Excluded – C**	—
	539	Periodontal health doesn't affect general health	**Retained**	**Retained**	**Included**
	540	Visiting a dentist regularly is unnecessary	**Retained**	**Retained**	**Included**
	541	Scaling damages enamel	**Retained**	**Excluded – B**	—

	542	Periodontal disease is hereditary and not preventable	**Retained**	**Retained**	**Included**
	543	Herbal or saltwater remedies alone cure gum disease	**Retained**	**Retained**	**Included**
	544	Brushing twice a day is insufficient	**Retained**	**Retained**	**Included**
	545	Pregnant women are not at increased gum risk	**Retained**	**Retained**	**Included**
	546	Diabetes doesn't impact gum disease	**Retained**	**Retained**	**Included**
	547	Stress isn't related to gum disease	**Retained**	**Retained**	**Included**
	548	Antibiotics can replace scaling completely	**Retained**	**Retained**	**Included**
	549	Oil pulling can cure gum disease permanently	**Retained**	**Retained**	**Included**
	550	Using toothpicks frequently prevents gum disease	**Retained**	**Retained**	**Included**
**Kinra, M., Dental myth, fallacies and misconceptions in rural population of Bhopal city: a cross-sectional study. AJDS, 2018. 1(1): p. 1-7.doi.org/10.9734/AJDS/2018/44586**	551	Brushing since once a day is required only to maintain good oral hygiene	**Retained**	**Retained**	**Included**
	552	Using finger with powdered salt charcoal, brick powder, ash to clean the teeth is as effective as the use of toothbrush and toothpaste	**Retained**	**Retained**	**Included**
	553	Harder brushing for a longer time makes teeth cleaner	**Retained**	**Retained**	**Included**
	554	Eating sweets cause tooth decay	**Excluded**	—	—
	555	The worm is there inside the decayed tooth	**Excluded**	—	—
	556	Tooth decay is the result of past sins	**Excluded**	—	—

	557	Application of catechu prevents tooth decay	**Excluded**	—	—
	558	Hot water fermentation gives relief in swelling and pain caused by tooth decay	**Excluded**	—	—
	559	Keeping tobacco in a decayed tooth relives tooth pain	**Excluded**	—	—
	560	Baby tooth are not important as they are going to fall out anyway	**Excluded**	—	—
	561	Throwing the exfoliated milk tooth of the children on the roof of the house and keeping fallen teeth in rat holes of underneath a stone can lead to the eruption of health and strong permanent teeth	**Excluded**	—	—
	562	A baby with teeth at birth believed to be a threat	**Excluded**	—	—
	563	All dental treatments are painful	**Retained**	**Retained**	**Included**
	564	Dental treatment is always expensive	**Retained**	**Excluded – A**	—
	565	Home remedies are better for dental treatment that what the dental prescribes	**Retained**	**Excluded – A**	—
	566	If I am not pain I don’t need to visit the dentist	**Retained**	**Excluded – B**	—
	567	Extraction of teeth of upper jaw causes loss of vision	**Excluded**	—	—

	568	A decayed painful tooth can't be saved and better extract	**Excluded**	—	—
	569	Cleaning of teeth by dentist cause loosening of teeth	**Retained**	**Excluded – B**	—
	570	Extracted teeth need no replacement with artificial teeth	**Excluded**	—	—
	571	Chewing betel quid removes foul odour from the mouth	**Retained**	**Retained**	**Included**
	572	Betel quid chewing with slaked lime and tobacco keeps gum health	**Retained**	**Retained**	**Included**
	573	Chewing tobacco helps in maintaining good oral hygiene	**Retained**	**Retained**	**Included**

Note: "Retained" in the Initial Relevance Assessment indicates that the item proceeded to secondary screening. During secondary screening, excluded items were coded according to the following criteria: A, outside the study scope, including general clinical dentistry, financial costs, and non-periodontal procedures; B, redundant or duplicate items; C, specialty-specific topics addressing advanced clinical management and surgical techniques in periodontics, including specialized aspects of gingival recession; and D, pediatric dentistry-related topics. "Retained" in the Secondary Screening / Exclusion Code column indicates that the item passed secondary screening, whereas "Included" in the Question Development Pool column indicates inclusion in the 76-item source pool used for subsequent question development. An em dash (—) indicates that the item did not proceed to the corresponding subsequent stage because it had been excluded at an earlier stage.

METHODOLOGY & RESEARCH FLOW

Flow Diagram of Literature Search, Item Screening, and Question Set Development

14 Sources | 573 Raw Items | 76 Eligible Pool | 30 Final Questions

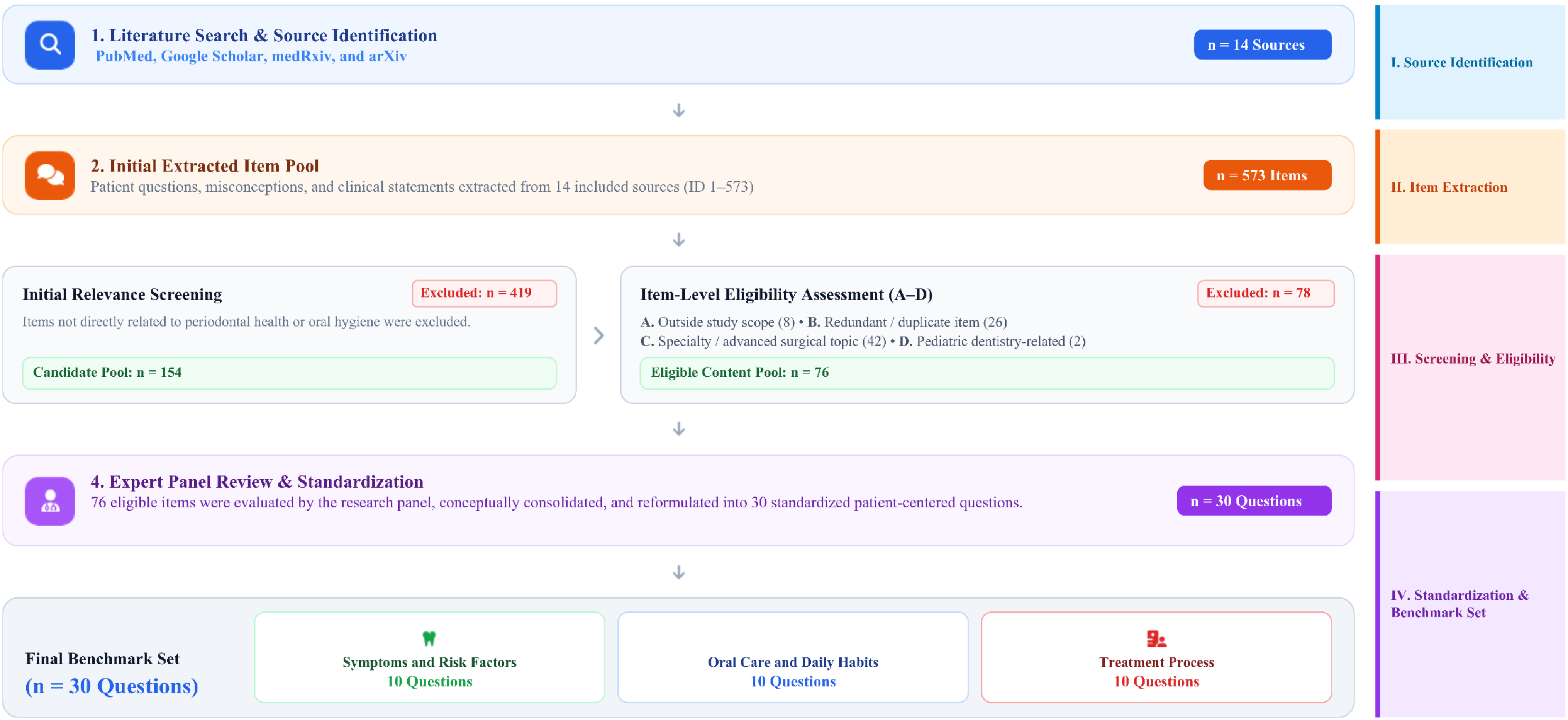

Supplement: Supplementary file 1 [file diagnostics-16-03158-s001.zip › Supplementary File S2 All Question.pdf]
