# Supplementary material for: Comparative Evaluation of Large Language Models in Responding to Common Misconceptions Regarding Periodontal Disease and Oral Hygiene
Source: Diagnostics (Basel). 2026 Sep 28;16(19):3158. doi: 10.3390/diagnostics16193158 (PMC13664004; doi:10.3390/diagnostics16193158)
Supplement: Supplementary file 1 [file diagnostics-16-03158-s001.zip › Supplementary File S4 LLMs response unprocessed.zip!/1. LLMs response/Copilot.pdf]

Q1 Do gum diseases only affect elderly people?

No — gum diseases do not only affect elderly people. They are common across all age groups, including children, teenagers, and adults, though severity and risk increase with age due to cumulative plaque build up and other health factors.

Who Can Get Gum Disease?

- **Children and teens** Gingivitis (early gum disease) is common in young people, especially during puberty when hormonal changes make gums more sensitive. Poor brushing habits also increase risk.
- **Adults** Many adults develop gum disease due to lifestyle factors like smoking, stress, or poor oral hygiene.
- **Elderly people** Older adults are more likely to have advanced periodontitis because plaque and tartar have accumulated over decades, and other health conditions (like diabetes or heart disease) can worsen gum health.

Key Risk Factors Beyond Age

- **Poor oral hygiene** – Not brushing and flossing regularly allows plaque to harden into tartar.
- **Smoking** – Tobacco use is one of the strongest risk factors.
- **Hormonal changes** – Puberty, pregnancy, and menopause can make gums more vulnerable.
- **Chronic diseases** – Diabetes, autoimmune disorders, and heart disease increase susceptibility.
- **Genetics** – Family history can predispose someone to gum problems.

Symptoms to Watch For

- Bleeding gums when brushing or flossing
- Swollen, red, or tender gums
- Persistent bad breath
- Gum recession (teeth look longer)
- Loose teeth or changes in bite alignment

Prevention Tips

- **Brush twice daily** with fluoride toothpaste.
- **Floss or use interdental brushes** every day.
- **Visit a dentist regularly** (every 6–12 months).
- **Avoid smoking** and maintain a balanced diet.
- **Manage chronic conditions** like diabetes to reduce risk.

Risks of Ignoring Gum Disease

- Tooth loss due to bone destruction.

- Increased risk of systemic health problems like **heart disease, stroke, and diabetes complications**.
- In pregnant women, gum disease has been linked to **preterm birth and low birth weight**.

So, gum disease is **not just an elderly issue** — it's a lifelong concern. Would you like me to focus more on **early gum disease in children**, **pregnancy-related gum issues**, or **advanced gum disease in older adults**?

Q2 Is it normal if my gums bleed when I brush my teeth?

Bleeding gums when brushing are *not normal* and usually signal gum disease (gingivitis or periodontitis), though they can also result from vitamin deficiencies, hormone changes, or certain medications. If bleeding persists for more than two weeks, you should see a dentist to rule out underlying conditions.

🚨 Why Gums Bleed

- **Gum disease**: The most common cause. Plaque buildup irritates gums, leading to inflammation and bleeding.
- **Poor oral hygiene**: Infrequent brushing/flossing allows bacteria to thrive.
- **Vitamin deficiencies**: Lack of vitamin C or K can weaken gum health.
- **Hormonal changes**: Pregnancy, puberty, or menopause can make gums more sensitive.
- **Medications**: Blood thinners increase bleeding risk.
- **Systemic conditions**: Diabetes, clotting disorders, or rarely leukemia can cause gum bleeding.

🦷 What You Should Do

- **Improve oral hygiene**: Brush twice daily with a soft-bristled toothbrush, floss once daily, and use antibacterial mouthwash.
- **Avoid smoking**: Smoking worsens gum disease.
- **Rinse with saltwater**: Helps reduce inflammation.
- **Monitor duration**: If bleeding continues beyond **two weeks**, consult a dentist.
- **Seek urgent care** if you also notice loose teeth, severe swelling, or mouth ulcers.

⚠️ Risks of Ignoring Bleeding Gums

- Progression from **gingivitis to periodontitis**, leading to gum recession, bone loss, and tooth loss.
- Possible indication of **systemic illness** (e.g., diabetes, blood disorders).
- Persistent bad breath and infections.

📊 Quick Comparison of Causes

Cause	Typical Signs	Next Step
**Gum disease**	Red, swollen gums, bad breath	Dentist cleaning & hygiene
**Vitamin deficiency**	Easy bruising, fatigue	Blood test, supplements
**Hormonal changes**	Pregnancy-related gum swelling	Dental monitoring
**Medications**	On anticoagulants	Doctor review
**Systemic illness**	Persistent bleeding, other symptoms	Medical evaluation

Q3 Can I get gum disease even if I brush my teeth regularly?

Yes — gum disease can still develop even if you brush regularly. Brushing alone doesn't always remove plaque between teeth or below the gumline, and factors like genetics, smoking, diabetes, or hormonal changes can increase your risk. Regular flossing and professional dental cleanings are essential to prevent gum disease.

🦷 Why Gum Disease Can Occur Despite Brushing

- Plaque buildup: Brushing removes surface plaque, but bacteria can hide between teeth and under gums. If not removed, plaque hardens into tartar, which brushing cannot eliminate.
- Flossing importance: Daily flossing or interdental cleaning is necessary to reach areas your toothbrush misses.
- Professional cleaning: Only dentists or hygienists can remove tartar. Without this, gum irritation can progress to gingivitis and periodontitis.
- Risk factors: Smoking, diabetes, hormonal changes (pregnancy, menopause), genetics, and certain medications can make gum disease more likely even with good brushing habits.

🚨 Symptoms to Watch For

- Bleeding gums when brushing or flossing
- Swollen, red, or tender gums
- Persistent bad breath
- Receding gums (teeth look longer)
- Loose teeth or changes in bite

🛡️ Prevention Tips

- Brush twice daily with fluoride toothpaste for at least 2 minutes.
- Floss or use interdental brushes once a day.
- Visit your dentist every 6–12 months (more often if you have risk factors).
- Avoid smoking and maintain a balanced diet rich in vitamin C.
- Manage health conditions like diabetes that increase gum disease risk.

⚖️ Key Takeaway

Brushing is necessary but not sufficient on its own. Gum disease prevention requires a combination of brushing, flossing, and professional care. Even people with excellent brushing habits can develop gum disease if other risk factors are present.

Q4 Can I have gum disease even if my gums don't hurt?

Yes — gum disease can develop even without pain. In fact, most people don't feel discomfort in the early stages, which is why it often goes unnoticed until more serious symptoms appear. The earliest warning signs are usually bleeding gums, swelling, or bad breath rather than pain. Regular dental checkups are essential to catch it early.

🦷 **Why Gum Disease Can Be Painless**

- **Early stage gingivitis**: Causes redness, swelling, and bleeding gums, but usually no pain. It is reversible with good oral hygiene.
- **Periodontitis**: A more advanced infection that damages gum tissue and bone. Pain may appear later, but many people only notice bleeding or loose teeth first.
- **Silent progression**: Gum disease often worsens gradually, with tissue and bone loss happening without obvious discomfort.

🚨 **Key Symptoms to Watch For**

Even without pain, gum disease may show signs such as:

- **Bleeding gums** when brushing or flossing
- **Swollen or red gums**
- **Persistent bad breath** or bad taste
- **Receding gums** making teeth look longer
- **Loose teeth** or changes in bite alignment

🛡️ **Risks of Ignoring Gum Disease**

- **Tooth loss**: Advanced gum disease is the leading cause of adult tooth loss.
- **Systemic health issues**: Linked to diabetes, heart disease, and pregnancy complications.
- **Chronic infection**: Bacteria can spread into the bloodstream, affecting overall health.

✅ **Prevention & Next Steps**

- Brush **twice daily** with fluoride toothpaste.
- Floss or use **interdental brushes** daily.
- Avoid smoking, which worsens gum disease.
- Schedule **regular dental checkups** (every 6–12 months, or more often if at risk).
- See a dentist promptly if you notice bleeding, swelling, or gum recession.

📊 Quick Comparison: Gingivitis vs. Periodontitis

Stage	Symptoms	Pain?	Reversible?
**Gingivitis**	Red, swollen gums, bleeding	Rare	Yes, with good care
**Periodontitis**	Gum recession, loose teeth, bone loss	Sometimes (later stages)	

Q5 Can gum disease get better without treatment?

No, gum disease does not get better on its own without treatment. Early-stage gum disease (gingivitis) can be reversed with professional dental care and improved oral hygiene, but once it progresses to periodontitis, it cannot heal naturally and will worsen without intervention, potentially leading to tooth and bone loss.

🦷 Stages of Gum Disease

Stage	Symptoms	Can It Heal Without Treatment?	Outcome if Untreated
**Gingivitis**	Red, swollen gums, bleeding when brushing/flossing	❌ No — but reversible with dental cleaning + good hygiene	Progresses to periodontitis
**Mild Periodontitis**	Gum pockets form, early bone involvement	❌ Cannot heal naturally	Infection spreads deeper
**Moderate Periodontitis**	Gum recession, pus, bad breath, loose teeth	❌ Needs professional treatment	Tissue and bone destruction
**Advanced Periodontitis**	Severe bone loss, teeth loosen/fall out	❌ Irreversible	Tooth loss, systemic health risks

🚨 Why Treatment Is Essential

- **Plaque and tartar** cannot be removed by brushing alone once hardened; only a dentist can clean them.
- **Untreated gum disease** leads to **tooth loss** and can increase risks for **heart disease, diabetes complications, and pregnancy issues**.
- **Early intervention** (scaling, root planing, improved hygiene) can stop progression and restore gum health if caught at the gingivitis stage.

✅ What You Can Do Right Now

- **Brush twice daily** with fluoride toothpaste.
- **Floss or use interdental brushes** daily.
- **Schedule a dental check-up** — especially if gums bleed or feel sore.
- **Quit smoking**, which worsens gum disease.
- **Maintain a healthy diet** rich in vitamin C and low in sugar.

⚠️ Risks of Ignoring Gum Disease

- **Permanent tooth loss** due to bone destruction.
- **Chronic bad breath** and gum pain.
- **Systemic health problems**: linked to cardiovascular disease, rheumatoid arthritis, and poor diabetes control.

In short: **gingivitis can be reversed with treatment, but periodontitis cannot heal naturally and will only worsen without professional care.** Would you like me to explain the **best treatments for gum disease** or focus on **home care tips** to prevent progression?

Q6 Are gum diseases rare?

No, gum diseases are not rare — they are actually very common. In fact, nearly half of all adults in the United States have some form of gum disease, and it is one of the leading causes of tooth loss worldwide.

🦷 How Common Are Gum Diseases?

- **Prevalence**: About **50% of adults** in the U.S. experience gum disease at some stage. Globally, it is considered one of the most widespread oral health problems.
- **Stages**:
  - *Gingivitis*: Early stage, reversible with good oral hygiene.
  - *Periodontitis*: Advanced stage, can cause bone loss and tooth loss.
- **Risk Factors**: Smoking, diabetes, poor oral hygiene, genetics, hormonal changes, and stress increase the likelihood of developing gum disease.

🚨 Symptoms to Watch For

- **Bleeding gums** when brushing or flossing
- **Swollen or red gums**
- **Bad breath** that doesn't go away
- **Loose teeth** or gum recession

These signs often appear gradually, and many people don't feel pain until the disease is advanced.

⚕️ Why It Matters

- Gum disease is **the leading cause of tooth loss** in adults.
- It has been linked to **systemic health problems** such as **heart disease, diabetes, and stroke**.
- Early detection and treatment can prevent irreversible damage.

🛡️ Prevention & Care

- **Daily brushing** with fluoride toothpaste
- **Flossing** or using interdental brushes

- **Regular dental checkups** (every 6–12 months)
- **Quit smoking** to reduce risk
- **Professional cleaning** to remove tartar

📊 **Quick Comparison: Gingivitis vs. Periodontitis**

**Stage**	**Gingivitis**	**Periodontitis**
**Severity**	Mild, reversible	Severe, irreversible damage
**Symptoms**	Red, swollen gums, bleeding	Loose teeth, gum recession, bone loss
**Pain**	Usually painless	Can cause pain when chewing
**Treatment**	Improved hygiene, cleaning	Deep cleaning, surgery, grafts

In short, gum diseases are **very common and preventable**. Would you like me to focus more on **prevention tips** or on **treatment options** for advanced cases?

Q7 Is it normal to lose teeth because of gum disease as you get older?

Yes, gum disease (periodontitis) is the leading cause of tooth loss in adults, but it is *not* an inevitable part of aging. With proper oral hygiene and regular dental care, most people can prevent tooth loss even later in life. Gum disease progresses when plaque and tartar damage the gums and supporting bone, eventually loosening teeth if untreated.

🦷 **Why Gum Disease Causes Tooth Loss**

- **Periodontitis**: A severe gum infection that damages soft tissue and bone, leading to loose teeth and eventual loss.
- **Plaque and tartar**: Bacterial buildup under the gumline triggers inflammation and infection.
- **Smoking**: Doubles the risk of gum disease and makes treatment less effective.
- **Systemic conditions**: Diabetes, rheumatoid arthritis, and immune disorders increase susceptibility.

📊 **Tooth Loss Facts**

**Cause**	**Impact**	**Notes**
**Gum disease**	cause of adult tooth loss	Preventable with hygiene & dental care
**Cavities**	Decay destroys tooth structure	Common but treatable early
**Trauma**	Accidents or injury	Mouthguards reduce risk
**Bruxism**	Grinding wears teeth down	Night guards help

- **178 million U.S. adults** have lost at least one tooth.
- **40 million adults** have lost all their teeth.

🚨 Warning Signs of Gum Disease

- Bleeding gums when brushing or flossing
- Swollen, tender, or receding gums
- Persistent bad breath
- Loose teeth or shifting bite

If you notice these, **see a dentist promptly** — early treatment can stop progression.

✅ Prevention & Care

- **Brush twice daily** with fluoride toothpaste
- **Floss once daily** to remove plaque between teeth
- **Dental checkups every 6–12 months** (more often if high risk)
- **Quit smoking** to improve gum healing
- **Balanced diet** with vitamin C for gum health

🧩 Treatment Options if Teeth Are Lost

- **Dental implants**: Long-lasting, closest to natural teeth
- **Bridges**: Fill gaps using adjacent teeth
- **Dentures**: Replace multiple missing teeth

Q8 If gum disease runs in my family, will I definitely get it too?

Having gum disease in your family does *not* mean you will definitely get it. Genetics can increase your susceptibility, but lifestyle factors like oral hygiene, smoking, and health conditions (such as diabetes) play a much larger role in whether gum disease develops.

🧬 Genetics vs. Environment in Gum Disease

- **Genetic predisposition:** Up to 30% of people may have genetic variations that make them more vulnerable to gum inflammation. These include differences in immune response proteins like interleukin-1 (IL-1).
- **Environmental factors:** Smoking, poor oral hygiene, uncontrolled diabetes, and stress are stronger predictors of gum disease than genetics.
- **Family history:** If gum disease runs in your family, you may be predisposed, but it is not inevitable. Preventive care can significantly reduce risk.

🚨 Risk Factors That Matter Most

- **Plaque buildup:** The primary cause of gum disease; hardened plaque (tartar) irritates gums.
- **Smoking:** Doubles the risk of gum disease and slows healing.
- **Diabetes:** High blood sugar weakens gum defenses.
- **Hormonal changes:** Pregnancy, menopause, and puberty can make gums more sensitive.

- **Stress:** Weakens immunity and may worsen gum inflammation.

🛡️ Prevention Strategies

- **Daily oral hygiene:** Brush twice daily with fluoride toothpaste and floss once daily.
- **Regular dental visits:** Professional cleanings every 6 months (or more often if at higher risk).
- **Lifestyle changes:** Quit smoking, manage diabetes, eat a balanced diet, and reduce stress.
- **Early detection:** Watch for bleeding gums, bad breath, or gum recession.

📊 Quick Comparison: Genetics vs Lifestyle

Factor	Impact on Gum Disease	Control Level
**Genetics**	30–50% contribution to susceptibility	Cannot change, but manageable
**Smoking**	Doubles risk, worsens severity	Fully controllable
**Oral hygiene**	Directly prevents plaque buildup	Fully controllable
**Diabetes**	Increases infection risk	Manageable with treatment
**Stress**	Weakens immune response	Partially controllable

✅ Key Takeaway

Even if gum disease runs in your family, **you can greatly reduce your risk** with consistent oral hygiene, regular dental checkups, and healthy lifestyle choices. Genetics may load the gun, but environment pulls the trigger.

Q9 Can stress make me more likely to get gum disease?

Yes — chronic stress can increase your risk of gum disease. Elevated stress hormones like cortisol weaken your immune system, promote inflammation in the gums, and often lead to habits (like teeth grinding or neglecting oral hygiene) that make gum problems worse. Managing stress and maintaining consistent oral care are key to protecting your gum health.

How Stress Affects Gum Health

- **Immune suppression**: Stress reduces your body's ability to fight bacteria, allowing harmful oral microbes to thrive.
- **Inflammation**: Cortisol triggers chronic inflammation, making gums more sensitive and prone to infection.
- **Dry mouth**: Stress lowers saliva production, reducing natural protection against bacteria.
- **Bruxism**: Teeth grinding or jaw clenching under stress puts pressure on gums, leading to irritation and recession.
- **Lifestyle habits**: Smoking, alcohol, poor diet, or skipped brushing/flossing during stressful times all worsen gum disease risk.

Signs Stress May Be Affecting Your Gums

- **Bleeding gums** when brushing or flossing
- **Red, swollen, or tender gums**
- **Persistent bad breath**
- **Loose teeth or gum recession**
- **Jaw soreness or tooth sensitivity**

Prevention & Management Tips

- **Stick to oral hygiene**: Brush twice daily, floss, and use antibacterial mouthwash.
- **Stress management**: Try meditation, yoga, deep breathing, or regular exercise.
- **Healthy diet**: Eat vitamin C-rich foods (oranges, peppers) and omega-3s (salmon, walnuts).
- **Stay hydrated**: Water helps prevent dry mouth and washes away bacteria.
- **Address bruxism**: Ask your dentist about a nightguard if you grind your teeth.
- **Regular dental visits**: Professional cleanings and early detection are crucial.

Risks & Trade-offs

- **Untreated stress-related gum issues** can progress from gingivitis (reversible) to periodontitis (irreversible), leading to tooth loss.
- **Even with good hygiene**, chronic stress may slow gum healing, making dental treatment less effective.

Visual Guide

Here’s how stress impacts gum health at different levels:

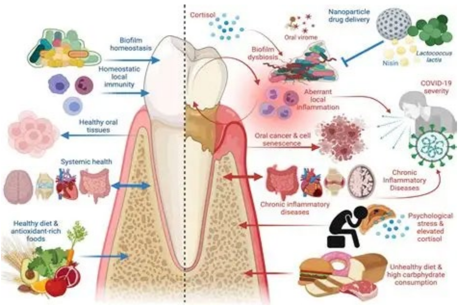

Q10 Do gum diseases only affect the mouth and teeth?

No — gum diseases primarily affect the mouth and teeth, but they can also have serious effects on overall health, including links to heart disease, diabetes, respiratory problems, and pregnancy complications. This happens because bacteria and inflammation from the gums can spread into the bloodstream and strain the immune system.

🦷 **Local Effects of Gum Disease**

- **Gum inflammation**: Red, swollen, bleeding gums are the earliest signs (gingivitis).
- **Tooth loss**: Advanced gum disease erodes bone and tissue, loosening teeth.

- **Bad breath**: Persistent halitosis due to bacterial infection.
- **Gum recession**: Gums pull away, exposing roots and increasing sensitivity.

🌍 **Systemic Health Connections**

- **Heart disease**: Bacteria from gums can enter the bloodstream, contributing to arterial inflammation and cardiovascular risk.
- **Diabetes**: Gum disease makes blood sugar harder to control, while diabetes increases gum infection risk.
- **Respiratory disease**: Inhaled oral bacteria may worsen lung conditions.
- **Pregnancy complications**: Associated with preterm birth and low birth weight.
- **Autoimmune disorders**: Conditions like rheumatoid arthritis and lupus are linked to gum inflammation.

📊 **Comparison Table: Local vs Systemic Effects**

Local Effects	Systemic Effects
**Gum swelling & bleeding**	Increased risk of heart disease
**Tooth loosening & loss**	Poor diabetes control
**Bad breath & abscesses**	Respiratory infections
**Gum recession**	Pregnancy complications

⚠️ **Risks & Prevention**

- **Risks**: Smoking, poor oral hygiene, stress, hormonal changes, and genetic predisposition.
- **Prevention**: Brush twice daily, floss, avoid tobacco, and get regular dental checkups.

✅ **Key Takeaway**

Gum disease is not just a dental issue — it's a **whole-body health concern**. Treating and preventing gum disease protects both your teeth and your long-term health. Would you like me to explain how **gum disease prevention** strategies differ for people with **diabetes** versus those with **heart disease**?

Q11 Will a hard-bristled toothbrush clean my teeth better?

Hard-bristled toothbrushes can remove plaque effectively, but they are more likely to cause gum damage, enamel wear, and sensitivity compared to soft or medium bristles. Dentists generally recommend soft-bristled brushes for safe, thorough cleaning.

🦷 **Cleaning Effectiveness**

- **Hard bristles**: They can scrape plaque and stains more aggressively, which may feel like a deeper clean.
- **Soft bristles**: Designed to flex and reach between teeth and along the gumline, removing plaque effectively without damaging tissues.

- **Medium bristles**: A compromise between cleaning power and gentleness, but still riskier than soft bristles for sensitive gums.

⚠️ Risks of Hard-Bristled Toothbrushes

- **Gum abrasion**: Hard bristles can cause micro-tears in gum tissue, leading to bleeding and discomfort.
- **Gingival recession**: Repeated trauma from stiff bristles may push gums back, exposing tooth roots.
- **Enamel wear**: Over time, hard brushing can erode enamel, increasing sensitivity.
- **Sensitivity**: Exposed dentin from gum recession or enamel loss can cause pain with hot, cold, or sweet foods.

✅ Dentist Recommendations

- **Use soft-bristled brushes**: They are effective at plaque removal when paired with proper technique and fluoride toothpaste.
- **Brush gently**: Pressure matters more than bristle stiffness; brushing too hard with any brush can cause damage.
- **Replace every 3–4 months**: Worn bristles lose effectiveness and can harbor bacteria.
- **Consider electric toothbrushes**: Many models have pressure sensors and oscillating heads that clean efficiently without excessive force.

📊 Comparison Table

Toothbrush Type	Cleaning Effectiveness	Gum Safety	Enamel Safety	Best For
**Hard bristle**	High plaque removal	Poor (abrasion risk)	Poor (wear risk)	Heavy stain removal (coffee, smoking)
**Medium bristle**	Moderate	Moderate	Moderate	General use (not sensitive gums)
**Soft bristle**	High (with proper technique)	Excellent	Excellent	Daily use, sensitive gums, children

🎯 Key Takeaway

If your goal is **long-term oral health**, a **soft-bristled toothbrush** is the safest and most effective choice. Hard bristles may feel stronger but can cause lasting damage to gums and enamel. If you struggle with stains, consider whitening toothpaste or professional cleaning instead of relying on hard bristles.

Q12 Should I stop brushing my teeth for a few days if my gums are bleeding?

No — you should not stop brushing your teeth if your gums are bleeding. In fact, stopping can make things worse, since plaque buildup is the most common cause of bleeding gums. Instead, use a soft-

bristled toothbrush, brush gently, floss daily, and see a dentist if bleeding persists for more than two weeks.

Why Gums Bleed

- **Gum disease**: Gingivitis (early stage) and periodontitis (advanced stage) are the leading causes.
- **Plaque buildup**: Not brushing allows bacteria to irritate gums.
- **Vitamin deficiencies**: Lack of vitamin C or K can contribute.
- **Other health issues**: Diabetes, blood-thinning medications, or rare conditions like leukemia.

What To Do Instead of Stopping Brushing

- **Brush gently**: Use a soft-bristled toothbrush 2–3 times daily.
- **Floss daily**: Removes plaque between teeth.
- **Rinse with saltwater**: Helps reduce inflammation.
- **Use antibacterial mouthwash**: Controls bacteria.
- **Avoid smoking**: Smoking worsens gum problems.

When to See a Dentist

- If bleeding **lasts longer than two weeks**.
- If gums are **swollen, painful, or receding**.
- If you notice **bad breath, loose teeth, or mouth ulcers**.

Risks of Ignoring Bleeding Gums

- **Progression to periodontitis** → bone loss and loose teeth.
- **Persistent infection** → bad breath and abscesses.
- **Underlying health conditions** may worsen if untreated.

Visual Guide

Here's what bleeding gums and gum disease progression look like:

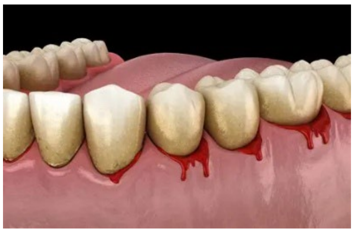

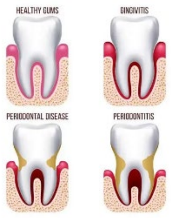

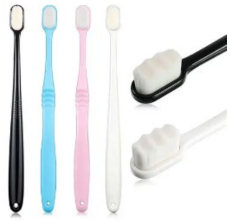

Q13 Do I need to brush my teeth after every meal to keep my gums healthy?

You don't need to brush after every meal to keep your gums healthy — dentists recommend brushing twice daily with fluoride toothpaste, plus flossing once a day. Brushing immediately after acidic foods can actually damage enamel, so waiting at least 30 minutes is safer.

🦷 Recommended Brushing Routine

- **Twice daily brushing**: Morning and before bed are the most important times.
- **Fluoride toothpaste**: Strengthens enamel and protects against cavities.
- **Flossing once daily**: Removes plaque and food particles between teeth where brushes can't reach.
- **Mouthwash use**: Helps reduce bacteria and freshen breath.

✅ Brushing After Meals: Pros & Cons

Benefit	Risk
**Removes food particles quickly**	Brushing too soon after acidic foods (citrus, soda) can erode enamel
**Reduces plaque buildup**	Over-brushing may irritate gums
**Helps prevent bad breath**	Requires waiting ~30 minutes after acidic meals

🌍 Practical Tips for Daily Gum Health

- **Wait 30–60 minutes after acidic meals** before brushing to avoid enamel wear.
- **Carry travel toothbrushes** if you want to brush after lunch at work or school.
- **Drink water after meals** to rinse away food particles and acids until you can brush.
- **Regular dental checkups** (every 6 months) are essential for gum health.

🚨 Risks of Over-Brushing

- **Enamel erosion** if brushing too soon after acidic foods.
- **Gum irritation** from brushing too hard or too often.
- **False sense of security** — brushing alone isn't enough; flossing and professional cleanings are critical.

📌 Key Takeaway

For healthy gums, **brush twice daily, floss once daily, and wait at least 30 minutes after acidic meals before brushing**. Brushing after every meal isn't necessary, but rinsing with water or chewing sugar-free gum can help between brushes.

Q14 Even if I brush my teeth regularly, do I still need to get my gums checked?

Yes — even if you brush your teeth regularly, it's still important to have your gums checked. Brushing is excellent for removing plaque from the surfaces of your teeth, but gum health involves more than just brushing:

- **Gum disease**: Conditions like gingivitis and periodontitis can develop silently, even with good brushing habits. Early signs include bleeding, swelling, or tenderness, which a dentist can spot before they worsen.

- **Plaque and tartar**: Brushing doesn't always remove plaque from below the gumline or hardened tartar, which requires professional cleaning.
- **Systemic health links**: Gum disease has been linked to heart disease, diabetes, and other systemic conditions, making regular checkups more important.
- **Preventive care**: Dentists can provide scaling, polishing, and advice tailored to your gum health, helping prevent long-term damage.

So, while brushing is a cornerstone of oral hygiene, gum checkups are about catching issues you can't see or feel yet. If you notice bleeding when brushing, persistent bad breath, or gum recession, those are clear signals to get checked sooner rather than later.

Q15 Is brushing my teeth once a day enough to keep my mouth healthy?

Brushing only once a day is not enough to keep your mouth healthy. Dentists and health organizations strongly recommend brushing twice daily with fluoride toothpaste for at least two minutes each time to effectively prevent cavities, gum disease, and plaque buildup.

🦷 Why Twice Daily Matters

- **Plaque buildup**: Plaque forms constantly, day and night. If not removed regularly, it produces acids that erode enamel and irritate gums.
- **Cavity prevention**: Brushing twice daily reduces the risk of tooth decay by removing food particles and bacteria before they harden into tartar.
- **Gum health**: Infrequent brushing increases the risk of gingivitis and periodontitis, which can lead to tooth loss.
- **Fresh breath**: Brushing twice helps control odor-causing bacteria more effectively than once.

✅ Recommended Routine

- **Brush twice daily**: Morning (ideally before breakfast) and before bed.
- **Use fluoride toothpaste**: Strengthens enamel and protects against decay.
- **Brush for 2 minutes**: Cover every surface of every tooth.
- **Floss once daily**: Removes plaque and food between teeth where brushes can't reach.
- **Don't rinse with water after brushing**: Spit out excess toothpaste to keep fluoride on teeth.
- **Wait 30–60 minutes after acidic foods/drinks**: Brushing too soon can damage softened enamel.

⚠️ Risks of Brushing Only Once

- **Higher cavity risk**: Plaque acids attack enamel throughout the day.
- **Increased tartar buildup**: Hardened plaque is harder to remove and requires professional cleaning.
- **Gum disease**: Inflammation from bacteria can progress to serious periodontal disease.
- **Bad breath**: Bacteria multiply overnight if teeth aren't cleaned before bed.

Comparison Table

Routine	Effectiveness	Risks
**Once daily**	Removes some plaque	High risk of cavities, gum disease, bad breath
**Twice daily**	Optimal plaque removal	Strong enamel protection, healthier gums
**After every meal**	Extra clean, but not necessary	Risk of enamel wear if brushing too soon after acidic foods

🪥 Visual Guide

Here's what proper brushing looks like:

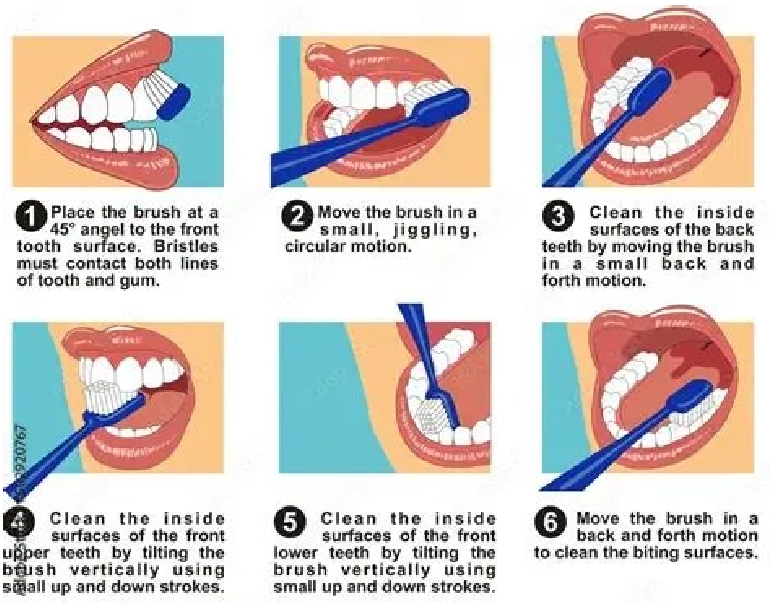

In short, **brushing once a day is insufficient**. To keep your teeth and gums healthy long-term, commit to brushing **twice daily for two minutes with fluoride toothpaste**. Would you like me to share some **best toothbrush options** or **tips for flossing correctly** to make your routine more effective?

Q16 Can flossing cause gum recession?

Flossing itself does not cause gum recession when done correctly — in fact, it helps prevent gum disease, which is the leading cause of recession. However, *aggressive or improper flossing techniques* (like snapping the floss into gums or sawing harshly) can irritate or damage gum tissue, contributing to recession over time.

✅ Proper Flossing vs. Gum Recession

- **Proper flossing**
  - Removes plaque and food particles between teeth.
  - Prevents gum disease (gingivitis, periodontitis), which is the main driver of gum recession.
  - Does not damage gum tissue when done gently.

- **Improper flossing**
  - Snapping floss forcefully into gums can cause trauma.
  - Sawing back and forth aggressively may wear away gum tissue.
  - Using too much pressure can irritate gums, leading to bleeding or sensitivity.

🚩 Main Causes of Gum Recession (Not Flossing)

Cause	Impact on Gums
**Aggressive brushing**	Wears away gum tissue and enamel.
**Periodontal disease**	Bacterial infection destroys gum tissue and bone.
**Genetics**	Thin gum tissue predisposes some people to recession.
**Bruxism**	Grinding/clenching puts excess force on gums.
**Misaligned teeth**	Uneven pressure points damage gums.
**Tobacco use**	Reduces blood flow, impairs healing, increases gum disease risk.

🦷 Correct Flossing Technique

- Use **18 cm of floss**, wrapping most around your middle fingers.
- Gently guide floss between teeth — *never snap*.
- Curve into a **"C" shape** around each tooth.
- Slide up and down gently below the gum line.
- Use a **fresh section** for each tooth.

⚠️ Risks & Prevention

- **Risk:** Improper flossing can irritate gums.
- **Prevention:** Learn correct technique or use alternatives like **water flossers** or **interdental brushes**.
- **Professional care:** If recession has already occurred, flossing cannot reverse it — treatments like **scaling and root planing** or **gum graft surgery** may be needed.

📌 Key Takeaway

Flossing is *essential* for gum health and does **not** cause recession when done properly. The real culprits are gum disease, aggressive brushing, genetics, and lifestyle factors. If you notice gum sensitivity or visible recession, consult a dentist promptly for evaluation and treatment.

Would you like me to show you a **step-by-step visual guide to proper flossing techniques** or a **comparison of flossing alternatives like water flossers vs interdental brushes**?

Q17 Do electric toothbrushes damage the gums more than regular toothbrushes?

Electric toothbrushes do not inherently damage gums more than manual toothbrushes — the risk comes from *improper use*, such as pressing too hard, using hard bristles, or failing to replace worn brush heads. When used correctly with soft bristles and gentle pressure, they are often safer and more effective than manual brushes.

⚖️ Electric vs. Manual Toothbrushes: Gum Health Comparison

Factor	Electric Toothbrush	Manual Toothbrush
**Pressure control**	Built-in pressure sensors prevent over-brushing	User must self-regulate pressure
**Brushing technique**	Brush does the work; requires guiding only	Scrubbing motion often leads to excessive force
**Plaque removal**	Removes ~21% more plaque, reduces gingivitis by ~11% in 3 months	Less consistent, depends on user skill
**Risk of gum recession**	Possible if pressing too hard or using hard bristles	Also possible with aggressive scrubbing
**Ease of use**	Helpful for people with limited dexterity	Requires more effort and technique awareness

🚨 Risks of Improper Use

- **Excessive pressure**: Combining motorized motion with manual force increases abrasion, leading to gum recession.
- **Hard bristles**: Too firm bristles scrape gum tissue, causing irritation.
- **Worn brush heads**: Frayed bristles become jagged and abrasive.
- **Scrubbing motion**: Electric brushes should be guided, not scrubbed back and forth.

✅ Safe Brushing Tips

- **Use soft bristles**: Gentle on gums, effective on plaque.
- **Let the brush do the work**: Apply minimal pressure, just guide along gumline.
- **Replace heads every 3 months**: Prevents damage from worn bristles.
- **Choose sensitive mode**: Many models have gum-care settings.
- **Follow the 2-minute rule**: Avoid over-brushing one area.

🩺 When to See a Dentist

Seek professional advice if you notice:

- **Bleeding gums** after brushing
- **Receding gumline** (teeth appear longer)

- **Persistent sensitivity** to hot/cold foods

📌 **Key Takeaway**

Electric toothbrushes are **not more damaging than manual ones** — in fact, they often improve gum health when used properly. The danger lies in *user error*: pressing too hard, using the wrong bristles, or poor technique. With gentle use and regular dental checkups, electric toothbrushes are a safe and effective choice.

Q18 Would using mouthwash alone be enough without brushing my teeth?

No — using mouthwash alone is not enough. Mouthwash can freshen breath and kill some bacteria, but it cannot physically remove plaque and food particles the way brushing and flossing do. Dentists emphasize that mouthwash should only *supplement* brushing and flossing, not replace them.

🦷 Why Brushing Is Essential

- Plaque removal: Brushing physically scrubs away plaque, a sticky film of bacteria that causes cavities and gum disease. Mouthwash cannot remove plaque.
- Cavity prevention: Fluoride toothpaste strengthens enamel and prevents decay, something mouthwash alone cannot achieve.
- Gum health: Brushing reduces gingival inflammation and bleeding, protecting against gum disease.

🌿 What Mouthwash Actually Does

- Freshens breath: Kills odor-causing bacteria temporarily.
- Reduces bacteria: Antibacterial rinses lower bacterial load but don't remove debris.
- Fluoride protection: Some rinses strengthen enamel, but only when combined with brushing.

🔑 Comparison Table

Method	Main Benefit	Limitations
Brushing teeth	Removes plaque, food particles, prevents cavities	Cannot reach between teeth fully (needs flossing)
Mouthwash	Freshens breath, kills bacteria, adds fluoride	Cannot remove plaque or food particles

🚨 Risks of Relying Only on Mouthwash

- Plaque buildup → leads to tartar, cavities, gum disease.
- Cavities → brushing is the only way to prevent daily plaque turning into decay.
- Gum disease → mouthwash cannot stop gingivitis without brushing and flossing.

✅ Best Oral Hygiene Routine

1. Brush twice daily with fluoride toothpaste for 2 minutes.
2. Floss once daily to clean between teeth.
3. Use mouthwash after brushing to kill bacteria and freshen breath.
4. Dental checkups every 6 months for professional cleaning.

In short, mouthwash is a helpful *addition* but never a substitute. If you skip brushing and rely only on mouthwash, you risk cavities, gum disease, and long-term oral health problems. Would you like me to break down the best mouthwash types (fluoride, antibacterial, alcohol-free) so you can choose the right one for your routine?

Q19 After I quit smoking, my gums started bleeding. Is that normal?

Yes, bleeding gums after quitting smoking can be normal. Smoking constricts blood vessels and masks gum disease symptoms; once you quit, improved blood flow reveals underlying inflammation, often causing temporary bleeding. However, persistent or heavy bleeding should be checked by a dentist.

🦷 Why Gums Bleed After Quitting Smoking

- **Nicotine effect**: Nicotine narrows blood vessels, reducing circulation and hiding gum disease symptoms.
- **Improved blood flow**: After quitting, circulation returns, exposing inflammation that was previously suppressed.
- **Underlying gum disease**: Bleeding is often a sign of gingivitis or periodontitis, conditions more common in smokers.
- **Healing response**: The bleeding indicates your immune system is finally addressing hidden gum problems.

⏳ Recovery Timeline

- **First week**: Blood flow improves, bleeding becomes noticeable.
- **Weeks 1–4**: Bleeding may peak as gums respond to hidden inflammation.
- **1–3 months**: With good oral hygiene, bleeding usually decreases significantly.
- **Up to 6 months**: Advanced gum disease may take longer to heal.

✅ What You Should Do

- **Visit a dentist**: Essential to rule out serious gum disease or other conditions.
- **Maintain oral hygiene**: Brush gently twice daily with fluoride toothpaste, floss correctly, and use antimicrobial mouthwash.
- **Stay hydrated**: Dry mouth worsens gum problems.
- **Eat a balanced diet**: Vitamins C and K are important for gum healing.
- **Regular check-ups**: Professional cleaning every 6 months helps prevent recurrence.

⚠️ When to Seek Immediate Help

- Persistent bleeding beyond 2 months
- Severe pain or swelling
- Pus at the gumline
- Loose teeth or gum recession
- Bad taste or breath that doesn't improve

Q20 Can natural products effectively keep my gums healthy?

Yes — certain natural products like aloe vera, tea tree oil, neem, and castor oil can help reduce gum inflammation, fight bacteria, and support healing, but they work best as *complements* to daily brushing, flossing, and professional dental care rather than replacements. If gum problems persist or worsen, you should consult a dentist to prevent progression to serious conditions like periodontitis.

🌿 Effective Natural Products for Gum Health

- **Castor Oil**
  - Contains ricinoleic acid with anti-inflammatory and antimicrobial properties.
  - Helps reduce swelling, soothe bleeding gums, and promote tissue healing.
  - Use: Massage a few drops into gums or try oil pulling for 5–10 minutes.
- **Aloe Vera Gel**
  - Rich in antioxidants and compounds like acemannan that stimulate healing.
  - Reduces redness, swelling, and bleeding in gingivitis.
  - Use: Apply food-grade gel to gums after brushing, leave for 10–15 minutes, then rinse.
- **Tea Tree Oil Toothpaste**
  - Antibacterial, antiviral, and antifungal properties target harmful bacteria.
  - Found in specialized natural toothpastes.
  - Helps prevent gum disease without harsh chemicals.
- **Neem**
  - Traditional antibacterial herb used in oral care.
  - Fights plaque and gingivitis.
  - Use: Neem oil, toothpaste, or chewing sticks.
- **Clove**
  - Analgesic and antimicrobial.
  - Provides relief from gum pain and controls bacteria.

    - Use: Clove oil or whole spice.

⚖️ Comparison Table

Natural Product	Key Benefit	Best Use
**Castor Oil**	Anti-inflammatory, healing	Massage/oil pulling
**Aloe Vera**	Soothes irritation, antimicrobial	Gel application
**Tea Tree Oil**	Antibacterial, antifungal	Toothpaste
**Neem**	Plaque & gingivitis control	Oil, toothpaste, twig
**Clove**	Pain relief, bacterial control	Oil, spice

🚨 Risks & Limitations

- **Not substitutes for dental care**: Natural remedies can ease symptoms but won't remove tartar or treat advanced gum disease.
- **Possible irritation**: Essential oils (like tea tree or clove) may cause sensitivity if not diluted properly.
- **Consistency required**: Benefits are gradual and require regular use.
- **Underlying conditions**: Gum disease can be linked to systemic health issues (e.g., diabetes, heart disease). Professional evaluation is essential.

✅ Practical Advice

- Use natural remedies as **adjuncts** to brushing twice daily, flossing, and regular dental checkups.
- If you notice **persistent bleeding, swelling, or gum recession**, seek professional care immediately.
- Combine natural care with a **balanced diet** (rich in vitamin C and antioxidants) to strengthen gum tissue.

Would you like me to focus more on **herbal remedies** like neem and clove, or on **plant gels and oils** such as aloe vera and castor oil?

Q21 Can getting a dental scaling damage my teeth?

Dental scaling does not damage healthy teeth when performed correctly by a professional; instead, it protects them by removing plaque and tartar. However, temporary side effects such as sensitivity, mild gum recession, or soreness can occur, but these usually resolve within days to weeks.

✅ What Dental Scaling Does

- **Plaque & tartar removal**: Scaling eliminates hardened deposits that brushing and flossing cannot reach.
- **Root planing**: Smooths tooth roots, making it harder for bacteria to reattach.

- **Gum disease prevention**: Reduces inflammation, bleeding, and risk of tooth loss.
- **Whole-body health**: Lower oral bacteria reduces risks linked to systemic conditions like heart disease.

⚠️ Possible Risks & Side Effects

- **Temporary sensitivity**: Teeth may feel sensitive to hot/cold for 1–2 months.
- **Mild gum recession**: Swollen gums shrink back after infection clears, exposing more tooth surface.
- **Tenderness or bleeding**: Short-term soreness or bleeding is common.
- **Loose feeling**: Teeth may feel slightly mobile until gums heal and tighten.
- **Infection risk**: Rare, but dentists may prescribe antibiotics if needed.

📊 Benefits vs Risks

Aspect	Benefits	Risks/Side Effects
**Plaque removal**	Prevents cavities & gum disease	None if done properly
**Root planing**	Stops bacteria reattachment	Temporary sensitivity
**Gum health**	Reduces bleeding & swelling	Mild gum recession
**Overall oral health**	Prevents tooth loss	Loose feeling in teeth (temporary)
**Aesthetics**	Cleaner, brighter smile	Minor soreness

🦷 Expert Guidance

- **Scaling is safe** when performed by trained dentists or hygienists.
- **Complications are rare** and usually mild compared to the risks of untreated gum disease.
- **Good oral hygiene afterward** (brushing, flossing, mouthwash) helps maintain results and reduces sensitivity.

📌 Key Takeaway

Dental scaling **does not damage teeth**; it protects them. Any discomfort is temporary and far less harmful than leaving tartar and bacteria untreated, which can cause irreversible gum disease and tooth loss.

Would you like me to explain how often you should get **scaling done** or how to reduce **sensitivity after scaling**?

Q22 Can dental scaling cause gaps between my teeth?

Dental scaling itself does not create gaps between teeth, but it can make existing spaces more noticeable. When tartar and plaque are removed, swollen gums shrink back to a healthier position, sometimes revealing spaces that were previously hidden. This is a normal part of recovery and usually indicates improved gum health rather than damage.

🦷 **Why Gaps May Appear After Scaling**

- **Gum recession**: If gums were inflamed or swollen due to gum disease, scaling reduces the swelling. As gums heal and tighten, teeth may look longer and small gaps may appear.
- **Tartar removal**: Hardened tartar can act like a "bridge" between teeth. Once removed, natural spacing is revealed.
- **Loose teeth**: In cases of advanced gum disease, teeth may feel temporarily loose after scaling. This usually improves as gums heal and reattach.
- **Root exposure**: Cleaning below the gumline can expose parts of the tooth root, making gaps more visible.

✅ **Benefits of Scaling**

- **Prevents tooth loss** by stopping gum disease progression.
- **Reduces bad breath** by removing bacteria.
- **Improves gum health** by eliminating infection and inflammation.
- **Protects overall health**, since oral bacteria can affect the heart and other organs.

⚠️ **Risks & Side Effects**

- **Temporary sensitivity** to hot/cold foods.
- **Mild bleeding or soreness** for a few days.
- **Visible gaps** due to gum shrinkage (not caused by scaling itself, but by healing).
- **Rare infection risk**, usually prevented with antibiotics if needed.

🔑 **Key Takeaway**

If you notice gaps after scaling, it usually means your gums are healthier and no longer swollen. The gaps are not caused by damage from the procedure but by the removal of tartar and the return of gums to their natural position. If gaps feel excessive or teeth feel loose, consult your dentist for follow-up care.

Q23 Do all gum diseases require the same treatment?

No, gum diseases do not all require the same treatment. Early-stage gum disease (gingivitis) can often be reversed with professional cleanings and improved oral hygiene, while advanced gum disease (periodontitis) may require deep cleaning, antibiotics, or even surgical procedures to restore gum and bone health.

🦷 Types of Gum Disease and Treatments

Stage	Gingivitis	Periodontitis
Cause	Plaque buildup causing gum inflammation	Plaque/tartar leading to infection, bone and tissue damage

Stage	Gingivitis	Periodontitis
Symptoms	Red, swollen gums, bleeding when brushing	Deep gum pockets, loose teeth, gum recession, bad breath
Treatment	- Professional dental cleaning (prophylaxis) - Improved daily brushing & flossing - Antibacterial rinses	- Scaling & root planing (deep cleaning) - Antibiotics (topical or oral) - Surgical options: flap surgery, bone grafts, gum grafts, guided tissue regeneration
Outcome	Reversible with proper care	Manageable but not fully curable; requires ongoing maintenance

🔑 Key Treatment Approaches

- Nonsurgical treatments
  - Scaling and root planing (deep cleaning under gums)
  - Antibiotic therapy (gels, rinses, or oral medication)
  - Laser therapy for tissue removal and bacteria control
- Surgical treatments
  - Flap surgery (pocket reduction)
  - Bone grafting to rebuild jaw support
  - Gum grafting to cover exposed roots
  - Guided tissue regeneration to encourage bone regrowth
- Lifestyle changes
  - Quit smoking (major risk factor)
  - Control diabetes and other systemic conditions
  - Maintain strict oral hygiene (brushing twice daily, flossing, regular dental visits)

⚠️ Risks of Untreated Gum Disease

- Progression from gingivitis to periodontitis
- Permanent bone loss and tooth loss
- Increased risk of systemic conditions (heart disease, stroke, pregnancy complications)

Q24 Is gum treatment painful?

Most gum treatments are not painful because dentists use local anesthesia to numb the area, but you may feel mild discomfort or sensitivity afterward depending on the procedure. Deep cleanings (scaling and root planing) can cause temporary soreness, while surgical treatments may involve more recovery discomfort.

🦷 **Types of Gum Treatments and Pain Levels**

Treatment	Pain During Procedure	After-Effects	Notes
**Professional cleaning**	Usually painless	Mild gum sensitivity	Routine cleaning removes plaque and tartar.
**Scaling & root planing**	Local anesthesia prevents pain	Gum soreness for 1–2 days	Deep cleaning under gums; common for gingivitis/early periodontitis.
**Antibiotic treatment**	No pain	Possible taste changes	Used to control bacterial infection.
**Flap surgery**	Done under anesthesia	Swelling, mild pain for a week	Gum tissue is lifted for deep cleaning.
**Gum grafts**	Anesthetized	Discomfort at donor site	Tissue taken from palate or donor source.
**Bone grafting**	Anesthetized	Recovery discomfort	Used in advanced periodontitis to restore bone.

🔑 Key Points About Pain Management

- **Dentists use local anesthesia** for most gum procedures, so you should not feel pain during treatment.
- **Post-treatment discomfort** is usually mild and manageable with over-the-counter painkillers.
- **Surgical treatments** (like gum grafts or flap surgery) may cause swelling and soreness, but dentists often prescribe pain relief and antibiotics.
- **Good oral hygiene** after treatment reduces discomfort and speeds healing.

⚠️ Risks and Recovery Considerations

- **Temporary sensitivity** to hot/cold foods is common after deep cleaning.
- **Swelling or bleeding** may occur after surgery but usually subsides within a week.
- **Smoking** delays healing and increases pain risk.
- **Untreated gum disease** can progress to periodontitis, which requires more invasive (and uncomfortable) treatments.

Q25 Can gum surgery cause tooth loss?

Gum surgery itself does not directly cause tooth loss, but untreated gum disease and severe gum recession — the conditions that often lead to surgery — can result in bone loss, tooth mobility, and eventual tooth loss if not managed properly. Gum grafting and periodontal procedures are designed to *prevent* tooth loss by stabilizing the gums and protecting exposed roots.

🔍 Key Points About Gum Surgery and Tooth Loss

- **Periodontal surgery**

    - Common risks: pain, bleeding, swelling, gum recession, sensitivity.
    - Goal: reduce gum pockets, regenerate bone, or stabilize gum tissue.
    - Tooth loss is *not* a direct risk of surgery, but failure of the procedure or poor healing can leave teeth vulnerable.
- **Gum recession**
    - Exposes tooth roots, increasing risk of cavities, sensitivity, and bone loss.
    - Left untreated, it can cause teeth to loosen and eventually fall out.
    - Surgery (gum grafting) is often recommended to prevent progression.
- **Gum graft surgery**
    - Covers exposed roots, reduces sensitivity, and lowers risk of decay.
    - Success rate: over 90%.
    - Risks: infection, graft failure, excessive bleeding — but tooth loss is rare unless underlying disease continues unchecked.

⚖️ Comparison Table: Gum Surgery vs. Gum Disease Progression

Factor	With Gum Surgery	Without Treatment
**Tooth stability**	Improved, gums reinforced	Teeth may loosen
**Risk of cavities**	Reduced (roots covered)	Increased (roots exposed)
**Bone health**	May regenerate or stabilize	Progressive bone loss
**Tooth loss risk**	Low (if surgery succeeds)	High (if disease advances)

🚨 Risks & Considerations

- **Surgery failure**: If grafts don't heal properly, gums may recede again.
- **Poor oral hygiene**: Even after surgery, inadequate cleaning can lead to gum disease relapse.
- **Smoking**: Strongly increases risk of graft failure and gum disease progression.
- **Underlying periodontal disease**: If untreated, it remains the main driver of tooth loss.

Q26 Can gum treatment make my teeth feel loose?

Yes — gum treatment can sometimes make teeth feel loose temporarily, especially if you have gum disease, because cleaning and surgery remove infected tissue and expose weakened bone support. However, this looseness often improves as gums heal and stabilize, though advanced cases may require further treatment to reinforce teeth.

Why Teeth May Feel Loose After Gum Treatment

- **Gum disease**: Periodontitis damages the bone and ligaments holding teeth. When infected tissue is cleaned away, teeth may feel less supported until healing occurs.

- **Scaling and root planing**: Deep cleaning removes plaque and tartar below the gumline. This can reduce inflammation but may initially make teeth feel mobile.
- **Gum surgery**: Procedures like flap surgery or grafting expose roots and bone. Teeth can feel loose during recovery but often stabilize afterward.
- **Bone loss**: If gum disease has already eroded bone, treatment may reveal the extent of looseness. Bone grafts or splinting may be needed.

Typical Recovery and Outcomes

- **Temporary looseness**: Many patients notice mobility for a few weeks after treatment. As gums heal and inflammation decreases, teeth often feel firmer.
- **Permanent looseness**: If gum disease is advanced, teeth may remain mobile. Dentists may recommend splinting (bonding teeth together), bone grafts, or in severe cases, extraction and replacement.
- **Improved stability**: With proper oral hygiene and follow-up care, gum treatment usually prevents further loosening and helps preserve teeth long-term.

Risk Factors That Increase Looseness

- **Advanced gum disease** with significant bone loss
- **Teeth grinding (bruxism)** adding extra pressure
- **Smoking** which slows healing
- **Poor oral hygiene** after treatment

What You Should Do

- **Follow your dentist's instructions** carefully after gum treatment.
- **Avoid hard or sticky foods** during recovery.
- **Use antibacterial rinses** to reduce infection risk.
- **Schedule regular checkups** to monitor healing and tooth stability.
- **Seek urgent care** if looseness worsens, pain develops, or pus/swelling appears.

Quick Comparison of Causes vs. Treatment

Cause	Effect on Teeth	Treatment Options
**Gingivitis**	Mild looseness from swollen gums	Professional cleaning, improved hygiene
**Periodontitis**	Bone loss, significant looseness	Scaling, surgery, bone grafts
**Dental trauma**	Sudden mobility	Splinting, bite adjustment
**Teeth grinding**	Gradual mobility	Night guard, stress management

⚠️ Important Note

If your teeth feel loose after gum treatment, **do not ignore it**. Sometimes this is part of healing, but it can also signal advanced gum disease. Please consult your dentist promptly to ensure proper stabilization and prevent tooth loss.

Would you like me to explain the **healing timeline after gum surgery** so you know what's normal versus concerning?

Q27 Does tartar removal cause more tartar buildup?

No, tartar removal does not cause more tartar buildup. In fact, professional removal prevents tartar from worsening and reduces your risk of gum disease. Tartar forms when plaque hardens, and if not removed, it continues to accumulate. Regular cleanings help keep teeth smooth, making it harder for new plaque to stick and calcify.

🦷 What Happens During Tartar Removal

- **Professional cleaning**: Dentists or hygienists use special tools to safely remove tartar above and below the gum line.
- **Polishing**: After removal, teeth are polished to smooth surfaces, reducing plaque adhesion.
- **Frequency**: Most people need cleanings every 6 months, but those prone to gum disease may need them more often.

🚫 Does Removal Cause More Buildup?

- **Myth**: Some believe scraping teeth makes them "rougher" and prone to tartar.
- **Reality**: Removal actually *reduces* buildup risk. Smooth enamel is less likely to attract plaque.
- **Cause of tartar**: Poor oral hygiene, sugary diet, smoking, or dry mouth—not the cleaning itself.

✅ How to Prevent Tartar After Cleaning

- **Daily brushing**: 2–3 times per day with fluoride toothpaste.
- **Flossing**: Once daily to remove plaque between teeth.
- **Mouthwash**: Alcohol-free antibacterial rinse twice daily.
- **Diet control**: Limit sugary foods and drinks.
- **Quit smoking**: Tobacco greatly increases tartar risk.

⚠️ Risks of Not Removing Tartar

- Gum disease (gingivitis, periodontitis)
- Cavities and enamel erosion
- Gum recession and tooth loss
- Persistent bad breath (halitosis)

📊 Quick Comparison

Factor	With Regular Removal	Without Removal
**Plaque adhesion**	Reduced (smooth enamel)	Increased (rough tartar surface)
**Gum health**	Healthy, less inflammation	Gingivitis, gum recession
**Risk of cavities**	Lower	Higher
**Breath**	Fresher	Bad breath likely

Q28 Are antibiotics enough to treat gum disease?

Antibiotics alone are not enough to treat gum disease. They can help control bacterial infection, but the cornerstone of treatment is professional dental cleaning (scaling and root planing), improved oral hygiene, and sometimes surgery for advanced cases. Without mechanical removal of plaque and tartar, antibiotics cannot stop disease progression.

🔑 Why Antibiotics Are Not Sufficient

- **Scaling and root planing**: Removes tartar and bacteria below the gumline — essential for healing.
- **Antibiotics**: Used as adjunct therapy (oral tablets, gels, or rinses) to reduce bacterial load, but cannot replace cleaning.
- **Surgical treatments**: Needed in advanced cases to reduce pocket depth, regenerate bone, or graft tissue.
- **Daily oral hygiene**: Brushing, flossing, and mouth rinses are critical to prevent recurrence.

🦷 Treatment Options by Severity

Stage	Main Treatment	Role of Antibiotics
**Gingivitis**	Professional cleaning + improved hygiene	Rarely needed
**Mild periodontitis**	Scaling & root planing	Sometimes adjunct
**Moderate periodontitis**	Deep cleaning, possible surgery	Often adjunct
**Advanced periodontitis**	Surgery (flap, grafts, regeneration)	Used to control infection

⚠️ Risks of Relying Only on Antibiotics

- **Incomplete treatment**: Plaque and tartar remain, disease progresses.
- **Resistance**: Overuse of antibiotics can lead to bacterial resistance.
- **Recurrence**: Without cleaning and hygiene, gum disease quickly returns.

🏥 What You Should Do

- **See a dentist or periodontist** for a full evaluation — gum disease severity determines treatment.
- **Commit to oral hygiene**: Brush twice daily, floss, and use interdental brushes.
- **Lifestyle changes**

Q29 After gum disease treatment, will I ever need treatment again?

You may need future treatment for gum disease, even after successful therapy, because gum disease is a chronic condition that can return if oral hygiene or risk factors aren't controlled. Regular dental check-ups and cleanings are essential to prevent relapse.

🔑 Why Gum Disease Can Return

- **Chronic nature**: Gum disease (periodontitis) isn't considered "curable" once bone or tissue loss has occurred. It can be managed but may flare up again.
- **Risk factors**: Smoking, diabetes, stress, and genetics increase the chance of recurrence.
- **Plaque buildup**: Even after treatment, plaque can accumulate quickly if brushing and flossing aren't consistent.

🦷 Types of Follow-up Care

Treatment type	Purpose	When Needed
**Scaling & root planing**	Deep cleaning to remove bacteria	Mild to moderate gum disease
**Antibiotics**	Control bacterial infection	Persistent or recurrent infection
**Flap surgery**	Access deep pockets for cleaning	Advanced gum disease
**Bone or tissue grafts**	Restore lost support	Severe cases with bone/gum loss
**Maintenance cleanings**	Prevent relapse	Every 3–6 months

🛡️ Prevention After Treatment

- **Brush twice daily** with fluoride toothpaste.
- **Floss or use interdental brushes** to clean between teeth.
- **Regular dental visits** (every 3–6 months for periodontal maintenance).
- **Quit smoking** — tobacco is a major risk factor.
- **Manage health conditions** like diabetes, which worsen gum disease.

⚠️ Risks of Not Following Up

- Gum disease can progress silently, leading to **tooth loss**.
- It's linked to **systemic health issues** such as heart disease, stroke, and diabetes complications.

- Once bone is lost, it cannot fully regenerate, making prevention critical.

Q30 Is surgery always necessary for gum treatment?

No, surgery is not always necessary for gum treatment. In early stages like gingivitis or mild periodontitis, non-surgical methods such as professional cleaning, scaling, root planing, and antibiotics are usually effective. Surgery is generally reserved for advanced cases where gum tissue or bone has been significantly damaged.

🦷 Non-Surgical Gum Treatments

- **Scaling and root planing**: Deep cleaning to remove plaque and tartar below the gumline and smooth root surfaces.
- **Antibiotics**: Topical gels or oral medication to control bacterial infection.
- **Improved oral hygiene**: Brushing twice daily, flossing, and using interdental brushes or water flossers.
- **Lifestyle changes**: Quitting smoking, managing diabetes, and regular dental check-ups.

🏥 Surgical Gum Treatments (For Advanced Periodontitis)

- **Flap surgery**: Gum tissue is lifted to allow deep cleaning and reshaping of bone.
- **Soft tissue grafts**: Reinforces receding gums using tissue from the palate or donor sources.
- **Bone grafting**: Restores bone lost due to infection, preventing tooth loss.
- **Guided tissue regeneration**: Special membranes encourage regrowth of bone and tissue.
- **Laser-assisted procedures**: Less invasive option for pocket reduction.

📊 Comparison Table

Treatment Type	When Used	Goal
**Scaling & Root Planing**	Early to moderate gum disease	Remove plaque/tartar, smooth roots
**Antibiotics**	Mild to moderate infection	Control bacterial growth
**Flap Surgery**	Advanced gum disease	Deep cleaning, reshape bone
**Bone Grafting**	Severe bone loss	Prevent tooth loss, regrow bone
**Soft Tissue Grafts**	Gum recession	Cover exposed roots, restore gumline

⚠️ Risks & Considerations

- **Non-surgical treatments** are lower risk but require consistent oral hygiene to prevent recurrence.
- **Surgical treatments** may involve longer recovery, higher cost, and risk of infection, but they are often necessary to save teeth in severe cases.
- **Early detection** is critical: gingivitis is reversible, but advanced periodontitis can only be managed, not fully cured.

Would you like me to focus more on **preventive steps** you can take now to avoid surgery, or on **advanced surgical options** if gum disease is already severe?
