# Supplementary material for: Comparative Evaluation of Large Language Models in Responding to Common Misconceptions Regarding Periodontal Disease and Oral Hygiene
Source: Diagnostics (Basel). 2026 Sep 28;16(19):3158. doi: 10.3390/diagnostics16193158 (PMC13664004; doi:10.3390/diagnostics16193158)
Supplement: Supplementary file 1 [file diagnostics-16-03158-s001.zip › Supplementary File S4 LLMs response unprocessed.zip!/1. LLMs response/Gemini 3.1.pdf]

Q1 Do gum diseases only affect elderly people?

No, that is a very common misconception. While the *risk* and severity of gum disease definitely increase as you get older, it can—and does—affect people of all ages, including children and teenagers.

In fact, according to the Centers for Disease Control and Prevention (CDC), nearly half (47.2%) of all American adults aged 30 and older have some form of periodontal (gum) disease.

Here is why it affects such a broad age range and how it develops.

What Actually Causes It?

Gum disease is fundamentally an infection caused by the bacteria in plaque—the sticky, colorless film that constantly forms on your teeth. If plaque isn't removed through daily brushing and flossing, it hardens into tartar (calculus), which can only be removed by a dental professional. The longer plaque and tartar stay on your teeth, the more damage they do.

While aging naturally causes wear and tear and immune system changes, several other factors heavily influence who gets gum disease:

- Hormonal Changes: Puberty, pregnancy, and menstruation can make gums more sensitive and vulnerable to gingivitis (the earliest, reversible stage of gum disease). This is why it often spikes in teenagers.
- Smoking and Tobacco Use: This is one of the most significant risk factors. The CDC reports that over 60% of current smokers have periodontal disease, as smoking weakens your immune system's ability to fight off the gum infection.
- Genetics: Some people are simply genetically predisposed to gum disease. Even with perfect oral hygiene, up to 30% of the population may be more susceptible.
- Stress: Chronic stress makes it harder for your body to fight off infections, including those in your mouth.
- Other Health Conditions: Diseases that interfere with the body's immune system, like diabetes, significantly increase the risk. Almost 60% of people with diabetes also have periodontal disease.

The Age Factor

While you don't have to be elderly to get it, the cumulative effect of plaque, tartar, and the gradual weakening of the immune system does take a toll over time. That is why the prevalence jumps significantly in older demographics—about 70.1% of adults 65 and older have periodontal disease.

Because gum disease is usually painless in its early stages, many younger adults don't even realize they have it until it progresses to a more severe stage.

Q2 Is it normal if my gums bleed when I brush my teeth?

This text is for informational purposes only. Consult a specialist for medical advice or diagnoses.

No, it is not normal for your gums to bleed when you brush your teeth — but it is incredibly common.

Healthy gums are firm and pale pink. If they are bleeding, it is your body's way of signaling that they are irritated or inflamed. Think of it like washing your hands: if your hands started bleeding every time you washed them, you would know something was wrong. The same rule applies to your mouth.

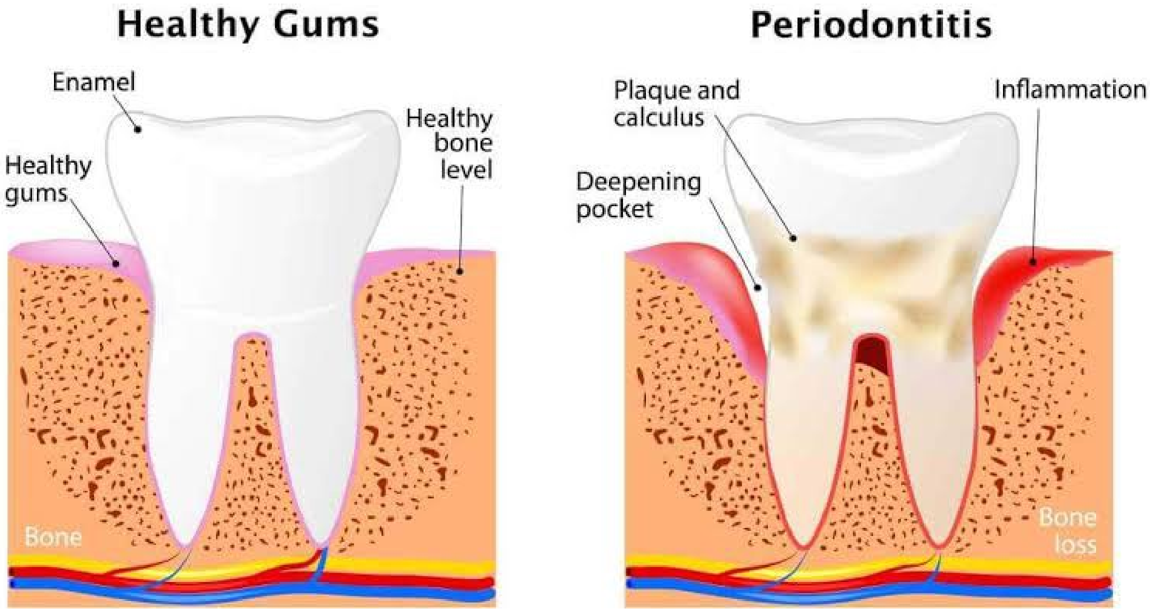

Here is a breakdown of why this happens and what you can do about it.

The Most Likely Cause: Early Gum Disease (Gingivitis)

The primary culprit behind bleeding gums is a buildup of **plaque** — a sticky, invisible film of bacteria that constantly forms on your teeth.

If plaque isn't thoroughly removed daily by brushing and flooring, the bacteria release toxins that irritate the gum tissue. This causes the gums to become red, swollen, tender, and prone to bleeding even with gentle brushing. This early stage of gum disease is called **gingivitis** .

Fortunately, gingivitis is entirely reversible with good oral hygiene and a professional dental cleaning. If left untreated, however, it can harden into tartar and progress into a much more serious condition called periodontitis, which can lead to tooth loss.

Other Common Causes

While plaque is the usual suspect, a few other factors can make your gums bleed:

- **Brushing too hard:** Using a toothbrush with hard bristles or scrubbing aggressively can physically tear delicate gum tissue. Always use a soft-bristled brush and gentle, circular movements.
- **A new flossing routine:** If you haven't flossed in a while and suddenly start, your gums might bleed for the first few days. This should stop within a week as the inflammation goes down.
- **Hormonal changes:** Fluctuations in hormones — particularly during pregnancy, puberty, menstruation, or menopause — increase blood flow to the gums and make them hyper-sensitive to plaque.
- **Medications:** Blood thinners and certain blood pressure or epilepsy medications can make your gums bleed more easily or cause gum swelling.

Healthline

- **Vitamin deficiencies:** Lacking Vitamin C or Vitamin K in your diet can weaken the blood vessels in your gums, though this is less common with modern diets.

When to See a Dentist

If your gums continue to bleed for more than a week or two despite careful, gentle brushing and daily flossing, you should schedule a checkup. You should also see a dentist immediately if you notice:

- Heavy or constant bleeding
- Gums that are visibly receding from your teeth
- Loose teeth or pain when chewing
- Persistent bad breath that won't go away

Q3 Can I get gum disease even if I brush my teeth regularly?

While regular brushing is your first line of defense, it is not a foolproof shield against gum disease (periodontal disease).

Here is why you can still develop gum disease despite having a solid brushing routine.

The Limitations of the Toothbrush

Even if you brush twice a day, mechanical limitations and technique can leave your gums vulnerable:

- Hard-to-reach areas: A toothbrush cleans the exposed surfaces of your teeth, but it cannot easily reach deep between the teeth or under the gumline. Plaque that is missed hardens into tartar (calculus) in just a couple of days. Once tartar forms, you cannot brush it away — it requires professional dental tools to remove.
- The flossing gap: If you brush but do not floss, you are leaving up to 35% of your tooth surfaces entirely uncleaned, providing a safe haven for bacteria to multiply.
- Brushing technique: Brushing too hard can actually cause your gums to recede, creating deeper pockets where bacteria can hide. Conversely, brushing too quickly (less than the recommended two minutes) leaves a sticky film of plaque behind.

Factors Beyond Oral Hygiene

Gum disease is an inflammatory response to bacteria.It isn't just about how clean your teeth are; it is also highly influenced by how your body reacts to that bacteria.

- Genetics: Gum disease can run in families.If you are genetically predisposed, your immune system may have an exaggerated, destructive inflammatory response to even a microscopic amount of plaque.
- Hormonal shifts: Fluctuations during puberty, pregnancy, menstruation, and menopause increase blood flow to the gums.This makes the gum tissue much more sensitive and vulnerable to irritation and swelling.

- Dry mouth: Saliva is your mouth's natural defense system, constantly washing away food particles and neutralizing bacterial acids. Many common medications (like antihistamines, antidepressants, and blood pressure pills) reduce saliva flow, creating a perfect breeding ground for bacteria.
- Systemic health conditions: Diseases that interfere with the body's immune system or inflammatory response — most notably diabetes , but also rheumatoid arthritis — significantly increase the risk and severity of gum infections.
- Smoking and vaping: Tobacco use is one of the most significant risk factors for gum disease.It restricts blood flow to the gums, which often masks early warning signs like bleeding, and heavily impairs the tissue's ability to fight off infection and heal.

Because of these hidden factors and the natural limitations of at-home tools, regular professional dental cleanings remain necessary to catch early inflammation and remove the tartar your toothbrush misses.

Q4 Can I have gum disease even if my gums don't hurt?

Yes, absolutely. In fact, most people with early to moderate gum disease do not experience any pain at all. It is often described as a "silent" condition—much like high blood pressure—because it can quietly cause significant damage long before you feel that anything is wrong.

Why It Doesn't Always Hurt

Gum disease begins when plaque—a sticky film of bacteria—builds up along the gumline. These bacteria release toxins that cause gingivitis (early inflammation of the gums).

Because this is a low-grade, chronic inflammation, it usually isn't intense enough to trigger your mouth's pain receptors.Your body simply adapts to the mild irritation. As the disease progresses into periodontitis, the bacteria travel below the gumline, slowly destroying the connective tissue and bone that hold your teeth in place.You might not actually feel pain until the very late stages, such as when an acute infection (like an abscess) forms or a tooth becomes visibly loose.

The "Silent" Warning Signs

Since pain isn't a reliable alarm bell, you have to look for visual and physical cues.Even if your mouth feels completely fine, you may have some stage of gum disease if you notice:

- Bleeding when brushing or flossing: Healthy gums are firm and shouldn't bleed from regular, gentle cleaning. *(Note: If you smoke, you might not experience this bleeding. Smoking restricts blood flow to the gums, which can completely mask this early warning sign.)*
- Color and texture changes: Healthy gums are generally light pink and firmly hug the teeth.Diseased gums tend to look dark red, puffy, or shiny.
- Persistent bad breath: Bacteria trapped in deep pockets below the gums release sulfur compounds that mints and regular brushing can't wash away.
- Receding gums: Your teeth might start looking "longer" or feeling more sensitive to cold as the gum tissue pulls away and exposes the roots.

Because the signs are so easily overlooked, the only definitive way to know the health of your gums is through a professional dental exam. A dentist or hygienist can use a tiny ruler to measure the depth of the pockets between your teeth and gums to spot disease long before it hurts.

Q5 Can gum disease get better without treatment?

If by "without treatment" you mean doing absolutely nothing and waiting for it to heal like a common cold, the answer is no . Gum disease is an active infection driven by a continuous buildup of bacteria (plaque). It will not resolve on its own.

However, if you mean "without professional dental procedures," the answer depends entirely on whether you are in the early or advanced stages of the disease.

Stage 1: Gingivitis (Reversible)

Gingivitis is the earliest stage of gum disease, where inflammation is strictly limited to the soft gum tissue.

- Symptoms: Red, puffy gums that bleed easily when you brush or floss.
- Can it get better at home? Yes. Because the infection hasn't reached the underlying bone, you can often reverse gingivitis by drastically improving your daily oral hygiene. This means brushing twice a day, flushing daily, and using an antibacterial mouthwash.
- The catch: While excellent home care stops new plaque, it cannot remove tartar (hardened plaque). If you have tartar buildup along the gumline, you will still need a routine professional dental cleaning to fully clear the inflammation.

Stage 2: Periodontitis (Irreversible)

If gingivitis is left unchecked, bacteria migrate below the gumline, triggering an advanced infection called periodontitis.

- Symptoms: Receding gums, deep "pockets" around the teeth, chronic bad breath, and eventually, loose teeth.
- Can it get better at home? No. At this stage, the bacteria have started destroying the connective ligaments and jawbone that hold your teeth in place. You cannot reach these deep bacterial colonies with a standard toothbrush or floss.
- Required treatment: Periodontitis requires professional dental intervention, such as scaling and root planning (a deep cleaning beneath the gums), localized antibiotics, or even surgery.The lost bone and tissue generally will not grow back, but clinical treatment can stop the disease and save your teeth.

Quick Comparison

Feature	Gingivitis	Periodontitis
Tissue Affected	Gums only	Gums, ligaments, and jawbone

Feature	Gingivitis	Periodontitis
Reversibility	Fully reversible	Irreversible (but manageable)
At-Home Healing	Possible with excellent hygiene	Impossible; requires a dentist

Q6 Are gum diseases rare?

No, gum diseases are not rare at all. In fact, they are among the most common chronic health conditions in the world.

The Statistics

Gum disease affects a massive portion of the population, though exact numbers depend on age and severity:

- Globally: The World Health Organization (WHO) estimates that severe periodontal (gum) disease affects around 19% of the global adult population — that is more than 1 billion people worldwide.
- In the US: According to the CDC, nearly half (42–47%) of all adults aged 30 and older have some form of periodontal disease.
- Aging: The prevalence increases significantly with age. Among adults aged 65 and older, over 60% have some form of gum disease.

The Two Main Stages

"Gum disease" is an umbrella term that primarily covers two main conditions:

1. Gingivitis: This is the earliest and most widespread stage, marked by red, swollen, and easily bleeding gums. Because it only affects the soft surface tissue, it is highly common but entirely reversible with good oral care.
2. Periodontitis: If gingivitis is left untreated, it can progress into periodontitis. This is a severe, irreversible infection that damages the soft tissue and destroys the bone supporting your teeth. It is a leading cause of tooth loss in adults.

Key Risk Factors

While poor oral hygiene (failing to brush and floss regularly) is the primary cause, several other factors heavily influence how common it is:

- Tobacco Use: Smoking is one of the most significant risk factors for developing severe gum disease, and it also makes treatment less effective.
- Systemic Diseases: Conditions that affect the body's immune system or inflammatory response — particularly diabetes — drastically increase the risk.
- Other Factors: Genetics, stress, hormonal changes (like pregnancy), and certain medications can also make gums more vulnerable to infection.

Because the early stages often cause no pain, many people don't realize they have it until it becomes severe. Daily brushing and flossing, paired with routine dental cleanings, are the most effective ways to prevent it.

Q7 Is it normal to lose teeth because of gum disease as you get older?

This text is for informational purposes only. Consult a specialist for medical advice or diagnoses.

While it is extremely common, losing teeth as you get older is not an inevitable or "normal" part of aging. You don't lose teeth simply because of your age; you lose them because of untreated oral health conditions, most notably advanced gum disease.

Gum disease, specifically in its advanced stage known as periodontitis, is actually the leading cause of tooth loss in adults.

How Gum Disease Causes Tooth Loss

Gum disease doesn't start by attacking the teeth directly; it attacks the foundation holding them in place.

1. Gingivitis (Early Stage): It starts with plaque buildup at the gumline. Bacteria in the plaque irritate the gums, making them red, swollen, and prone to bleeding. At this stage, the damage is completely reversible with good hygiene and a professional cleaning.
2. Periodontitis (Advanced Stage): If untreated, the plaque hardens into tartar. The inflammation spreads below the gumline, and your immune system's response to the bacteria starts breaking down the bone and connective tissue that hold your teeth in place.
3. Tooth Loss: As the bone and tissue are destroyed, the gums pull away from the teeth, forming deep "pockets" that harbor more bacteria. Eventually, the teeth become loose and either fall out on their own or need to be extracted by a dentist.

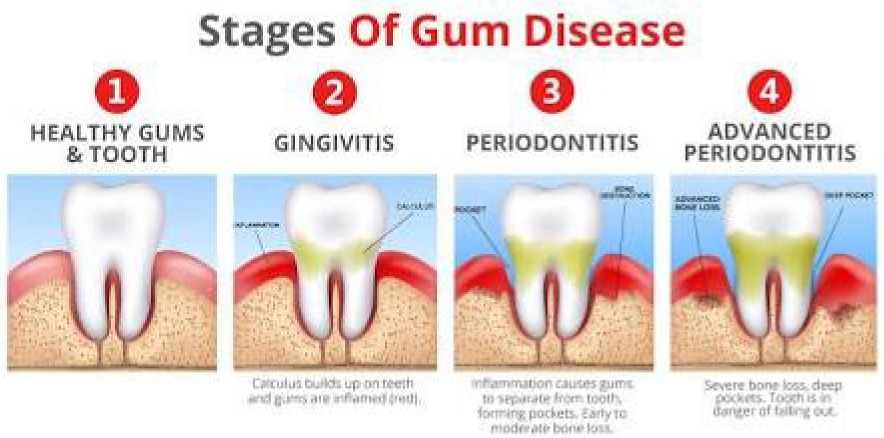

The Role of Aging

Age itself doesn't cause periodontitis, but the risk increases as you get older for a few reasons:

- Cumulative damage: It takes years for periodontitis to destroy enough bone to loosen a tooth. Older adults have simply had more time for the disease to progress if it has gone untreated.

- Dry mouth: Many medications commonly taken by older adults reduce saliva flow. Saliva is crucial for washing away bacteria and neutralizing acids.
- Other health conditions: Conditions like diabetes, which become more common with age, make it harder for the body to fight off infections, including those in the gums.

The good news is that with daily brushing and flossing, and regular dental checkups to catch issues early, you can keep your natural teeth for your entire life.

Q8 If gum disease runs in my family, will I definitely get it too?

No — having a family history of gum disease (periodontal disease) does not mean you are guaranteed to get it.

A great way to think about it in the dental world is: "Genetics load the gun, but lifestyle pulls the trigger." While your DNA might make your gums more vulnerable, the disease still needs bacteria from plaque to actually develop. If you control the plaque, you can control the disease.

Here is a breakdown of how your genetics actually play a role and what you can do to protect your smile.

How Genetics Affect Your Gums

Research shows that about 30% to 50% of your susceptibility to gum disease is influenced by your genes. If gum disease runs in your family, you might have inherited a few traits that make you more vulnerable:

- An exaggerated immune response: Gum disease isn't just an infection; it's an inflammation problem. Some people are genetically wired to have a stronger, more destructive inflammatory response to the bacteria in plaque, causing gum tissue to break down faster.
- Thinner gum tissue: Just like skin, gum thickness varies.People with genetically thinner gum tissue (a "thin biotype") are more prone to receding gums.
- Differences in collagen: Genetic variations can affect how well your body repairs collagen, making your gum tissue slightly weaker and slower to heal.

The bottom line: Your genetics might mean you have to work a little harder than someone without a family history, but your daily habits have a much bigger impact on your oral health than your DNA does.

How to Beat Your Genetics

If you know you are predisposed, you can't afford to be lazy with your oral hygiene. Here is how to keep your gums healthy:

- Step up your daily routine: Brushing twice a day and flossing daily aren't just suggestions for you — they are mandatory.Flossing physically breaks up the bacteria colonies between your teeth before they can harden into tartar.
- Increase your dental visits: Standard cleanings are usually every six months, but people with a family history of gum disease often benefit from going every 3 to 4 months. Early intervention is the best way to prevent permanent damage.

- Don't smoke: Smoking is one of the most significant risk factors for gum disease. Smokers are up to six times more likely to develop periodontal disease, and it drastically reduces your body's ability to heal gum tissue.
- Manage systemic health: Conditions like diabetes or chronic stress can weaken your immune system and make gum disease progress much faster.

Q9 Can stress make me more likely to get gum disease?

Yes, stress can significantly increase your risk of developing gum disease (periodontal disease) or make existing gum issues worse.

The connection between stress and your gums happens in two main ways: biologically (how your body physically reacts) and behaviorally (how your daily habits change).

The Biological Connection

When you are under chronic stress, your body stays in a prolonged "fight-or-flight" state. This directly impacts your oral tissues:

- Weakened Immune System: Stress triggers the release of cortisol. Persistently high cortisol levels suppress your immune system, making it much harder for your body to fight off the harmful bacteria that cause gum infections.
- Increased Inflammation: High stress hormone levels cause an exaggerated inflammatory response throughout your body. Even a small amount of plaque can trigger your gums to become swollen, tender, and prone to bleeding.

Use the interactive simulator below to see how rising stress levels directly alter the balance between your immune defenses and plaque bacteria.

Key insight: Because stress physically suppresses your immune response, some people develop gum problems during highly stressful periods even if they haven't changed their brushing or flossing routines.

The Behavioral Connection

Stress also shifts our daily habits, often in ways that quietly sabotage oral health:

Habit Change	How It Harms Your Gums
Teeth Grinding (Bruxism)	Clenching or grinding puts immense physical pressure on the tissues and bone supporting your teeth, accelerating gum recession and bone loss.
Skipping Oral Hygiene	When overwhelmed, people often rush or skip brushing and flossing entirely, allowing plaque to harden into tartar.

Habit Change	How It Harms Your Gums
Dry Mouth	Stress can reduce saliva production (as can many stress medications and extra caffeine). Saliva is crucial for washing away food particles and neutralizing bacterial acids.
Diet Changes	Seeking comfort in sugary snacks or simple carbohydrates feeds the exact bacteria responsible for gum disease.

Warning Signs to Watch For

Because stress compromises your body's natural defenses, it is important to be vigilant.Look out for these early indicators:

- Gums that bleed when you brush or floss.
- Red, swollen, or unusually tender gums.
- Persistent bad breath or a metallic taste in your mouth.
- Teeth that feel slightly loose or suddenly more sensitive.

If you are going through a demanding period, the best thing you can do for your oral health is to be uncompromising about your basics: keep brushing twice a day, floss daily, stay hydrated to prevent dry mouth, and don't skip your regular dental cleanings.

Q10 Do gum diseases only affect the mouth and teeth?

No, gum disease (periodontal disease) does not just affect your mouth. It has a profound impact on your overall, systemic health.

In fact, the mouth acts as a gateway to the rest of the body. When gum disease is left untreated, it stops being a localized dental issue and becomes a whole-body inflammatory burden.

How It Spreads

Gum disease impacts distant organs and systems primarily through two pathways:

- Bacterial Travel (Bacteremia): The mouth is full of bacteria. When gums are chronically infected and bleeding, routine activities like chewing, brushing, or flossing can force periodontal bacteria directly into the bloodstream, where they travel to other organs.
- Systemic Inflammation: Gum disease triggers a massive immune response. Your body produces inflammatory markers (like C-reactive protein and cytokines) to fight the oral infection. These molecules leak into your circulation, causing chronic inflammation throughout your entire cardiovascular system.

Major Health Connections

The continuous release of bacteria and inflammatory signals has been scientifically linked to several major health conditions:

Condition	The Connection
Cardiovascular Disease	Inflammatory markers and oral bacteria contribute to endothelial dysfunction and arterial plaque buildup (atherosclerosis), which increases the risk of heart attacks and strokes.
Type 2 Diabetes	This is a bidirectional relationship. Uncontrolled diabetes makes gum disease worse, while chronic gum inflammation increases insulin resistance, making it much harder to control blood sugar.
Respiratory Infections	Bacteria from infected periodontal pockets can be aspirated (breathed) directly into the lungs, increasing the risk for pneumonia and exacerbating conditions like COPD.
Pregnancy Complications	The systemic inflammatory burden of severe gum disease is associated with a significantly higher risk of preterm birth and low birth weight.
Autoimmune & Cognitive	Emerging research shows shared inflammatory pathways between gum disease and rheumatoid arthritis, as well as links to the neuroinflammation seen in Alzheimer's disease.

Because of these deep systemic links, treating gum disease isn't just about saving your teeth—it is an essential part of managing your total body health.

Q11 Will a hard-bristled toothbrush clean my teeth better?

No, a hard-bristled toothbrush will not clean your teeth better. In fact, most dentists and the American Dental Association (ADA) strongly advise against using them for daily brushing.

It is a common misconception to treat brushing your teeth like scrubbing a dirty pan—assuming the harder the bristles and the more pressure you apply, the cleaner your teeth will be. However, dental plaque is actually soft and sticky. You don't need to aggressively scrub it off; you simply need to gently sweep it away.

The Problem with Hard Bristles

Using a hard-bristled brush—especially if you brush with a heavy hand—acts a bit like sandpaper in your mouth. Over time, this leads to two major dental issues:

- Enamel Erosion: Enamel is the hard, protective outer layer of your teeth. Stiff bristles can physically wear this layer away, leaving your teeth permanently weakened, yellowed, and more prone to cavities.
- Gum Recession: Your gums are delicate soft tissue. Hard bristles irritate and traumatize the gums, causing them to pull back (recede) from the tooth.

Once enamel is worn away or your gums recede, the underlying layer of the tooth—called dentin—becomes exposed. Dentin contains microscopic tubes that lead directly to the tooth's nerve. This is why aggressive brushing with hard bristles is one of the leading causes of chronic tooth sensitivity to hot and cold temperatures.

What Actually Works

The ADA's consensus is clear: soft-bristled toothbrushes are the safest and most effective choice for almost everyone. Clinical studies consistently show that soft bristles remove plaque just as effectively as medium or hard bristles, but without the risk of damaging your mouth.

Because soft bristles are more flexible, they are actually *better* at bending and reaching into the microscopic crevices between your teeth and just under the gumline.

The secret to truly clean teeth isn't the firmness of the brush, but your technique:

1. Angle it right: Hold the brush at a 45-degree angle toward the gumline.
2. Be gentle: Use short, circular sweeping motions rather than aggressively sawing back and forth across your teeth.Let the tips of the bristles do the work.
3. Take your time: Brush for a full two minutes, twice a day.

If you feel like a soft manual brush isn't leaving your teeth feeling clean enough, look for a brush with multi-level or angled bristles. Alternatively, upgrading to an electric toothbrush (which still uses soft bristles) can provide that "extra clean" feeling without requiring you to scrub harder.

Q12 Should I stop brushing my teeth for a few days if my gums are bleeding?

This text is for informational purposes only. Consult a specialist for medical advice or diagnoses.

No, you absolutely should not stop brushing your teeth.

It feels counterintuitive—usually, when a part of your body bleeds, your first instinct is to leave it alone so it can heal. But your mouth operates under different rules.

Why Your Gums Are Bleeding

Bleeding is most commonly a sign of gingivitis (early gum disease). This happens when plaque—a sticky film of bacteria—builds up along your gumline. Your immune system reacts to the bacteria, causing your gums to become inflamed, swollen, and highly sensitive.

If you stop brushing, you are giving that bacteria a free pass to multiply. More plaque will accumulate, which will only make the inflammation and the bleeding worse. To stop the bleeding, you have to remove the plaque that's causing it.

What to Do Instead

You need to keep cleaning your teeth, but you need to adjust how you do it so you don't further irritate the tissue:

- Switch to a soft-bristled brush: Medium or hard bristles can tear at already inflamed tissue. Soft bristles are completely effective at removing plaque without causing damage.

- Ease up on the pressure: Don't scrub aggressively back and forth. Use gentle, circular motions angled at 45 degrees toward the gumline. Let the tips of the bristles do the work.
- Keep flossing: Your toothbrush can't reach the plaque hiding between your teeth. If flossing makes your gums bleed, don't panic—just be gentle. As you consistently clear that plaque away over a few days, the bleeding should stop.
- Rinse with saltwater: Swishing with warm salt water can help soothe the inflammation and promote healing.

When to see a professional If you keep up a gentle, thorough brushing and flossing routine and your gums are still bleeding after a week or two, it is time to see a dentist. Plaque that is left on the teeth eventually hardens into tartar (calculus), which cannot be brushed away at home and requires professional dental tools to remove.

Q13 Do I need to brush my teeth after every meal to keep my gums healthy?

No, you don't need to brush after every meal to maintain healthy gums. In fact, brushing that frequently—especially right after you eat—can actually do more harm than good to your teeth.

The American Dental Association (ADA) recommends brushing just twice a day for two minutes, paired with daily flossing, to keep your gums and teeth in top shape.

Why Brushing Immediately Can Be Harmful

When you eat, especially foods and drinks that are acidic (like citrus fruits, tomatoes, soda, or coffee), the pH level in your mouth drops and your tooth enamel temporarily softens.

If you brush while your enamel is in this weakened state, the abrasive action of your toothbrush bristles can literally scrub away microscopic layers of your enamel. Over time, this leads to enamel erosion, increased sensitivity, and a higher risk of decay.

The "30-Minute Rule"

If you strongly prefer to brush after a meal or snack, dental experts have a golden rule: wait at least 30 to 60 minutes.

This waiting period gives your saliva time to do its job. Saliva naturally neutralizes the acids, washes away lingering food particles, and remineralizes (hardens) your enamel so it is safe to brush again.

What to Do Right After Eating Instead

If you want to clear your mouth immediately after lunch without risking your enamel, try these steps instead:

- Swish with water: A vigorous rinse with plain water helps dislodge trapped food particles and dilutes mouth acids instantly.
- Chew sugar-free gum: Chewing stimulates your salivary glands. That extra saliva is your mouth's natural defense mechanism for neutralizing acid and washing away bacteria.
- Floss: If food is stuck between your teeth (which is a primary cause of localized gum inflammation), flossing is highly effective and doesn't involve scrubbing your enamel with abrasive toothpaste.

Ultimately, the true secret to keeping your gums healthy isn't brushing *more often*—it is brushing *effectively* twice a day and ensuring you clean between your teeth where the brush cannot reach.

Q14 Even if I brush my teeth regularly, do I still need to get my gums checked?

This text is for informational purposes only. Consult a specialist for medical advice or diagnoses.

Yes, absolutely. Even if your brushing routine is flawless, you still need regular professional gum check-ups.

Here is why brushing alone isn't a silver bullet for your gums:

The "Blind Spots" of Brushing

A toothbrush is excellent at cleaning the broad, flat, and exposed surfaces of your teeth. However, the bristles are physically unable to reach tightly between the teeth or deep underneath the gumline (a tiny pocket called the sulcus).

Here is a visual to show exactly what a toothbrush leaves behind:

Was this image helpful?

Key insight: The areas your brush misses are exactly where bacteria love to hide, multiply, and irritate your gums.

The Plaque to Tartar Pipeline

When plaque is left in those blind spots for just 24 to 72 hours, minerals in your saliva cause it to calcify and harden into a substance called tartar (or calculus).

- The Problem: You cannot brush or floss tartar away. It forms a rough, porous barrier that holds bacteria directly against your gum tissue, causing chronic inflammation.
- The Solution: Only a dentist or dental hygienist can safely remove tartar using specialized instruments (a process called scaling).

Gum Disease is "Silent"

Gum disease (periodontal disease) rarely causes pain in its early stages. By the time you notice symptoms at home—such as receding gums, chronic bad breath, or loose teeth—the disease has often progressed significantly and may have started destroying the bone that holds your teeth in place.

During a professional check-up, your dentist isn't just looking for cavities. They use a small ruler called a periodontal probe to gently measure the depth of the pockets between your teeth and gums.

- 1–3 millimeters: Generally healthy.
- 4+ millimeters: Indicates that your gums are inflamed and beginning to detach from the teeth, a primary sign of active gum disease.

In short: Brushing maintains your teeth day-to-day, but a professional check-up is the only way to measure your actual gum health, remove tartar, and stop gum disease before it causes permanent damage.

Q15 Is brushing my teeth once a day enough to keep my mouth healthy?

In short, no. Brushing just once a day falls short of what your mouth needs to stay healthy and avoid long-term dental issues.

The Official Recommendation

The American Dental Association (ADA) strongly recommends brushing twice a day for two minutes each time, using a fluoride toothpaste.

Why Once a Day Doesn't Cut It

When you only brush once every 24 hours, you give the bacteria in your mouth a massive head start.Here is exactly what happens:

- Plaque Hardens Quickly: Plaque is a sticky film of bacteria that constantly forms on your teeth. If left undisturbed for about 24 hours, it begins to harden into tartar (calculus). Once it becomes tartar, your toothbrush can no longer remove it—only a dental professional can scrape it away.
- Bacteria Multiply Rapidly: Every time you eat, especially carbohydrates and sugars, bacteria feast and produce acids that attack your tooth enamel. Brushing only once gives those acids a massive window to eat away at your teeth, leading to cavities.
- The Nighttime Danger Zone: If your single brushing session is in the morning, you leave an entire day's worth of food and bacteria on your teeth while you sleep. Your body's saliva production naturally drops at night. Because saliva is your mouth's natural defense mechanism for neutralizing acid, sleeping with unbrushed teeth leaves your enamel completely vulnerable.

If You Must Brush Only Once (Damage Control)

If you ever have a day where you only have the time or energy to brush once, do it right before bed. Cleaning away the day's buildup before your saliva production drops is critical. However, this is strictly a fallback for bad days—you should aim for twice daily to truly keep cavities, bad breath, and gum disease at bay.

You can experiment with the simulator below to see how adjusting your brushing habits affects plaque buildup over time:

Q16 Can flossing cause gum recession?

Yes, flossing can cause gum recession, but only if it is done incorrectly.

When done properly, flossing is actually one of the best ways to *prevent* gum recession because it removes the plaque and bacteria that lead to gum disease (the leading cause of receding gums). However, your gum tissue is delicate, and aggressive or improper technique can physically traumatize the tissue and cause it to pull away from the tooth.

Here are the flossing mistakes that lead to gum recession:

- Snapping the floss: Forcing the floss hard between tight teeth so that it violently snaps down into the gum tissue.This can cut the gums and cause permanent damage over time.
- The "Sawing" motion: Pulling the floss back and forth like a saw at the base of the tooth creates friction that wears down the gum line and even tooth enamel.
- Over-flossing: Flossing more than once or twice a day. Plaque takes 4 to 12 hours to develop, so excessive flossing provides no extra benefit and simply wears down your gums.
- Using too much force: Pushing down too hard into the gum pocket rather than gently sliding along the side of the tooth.

The Correct Way to Floss

To protect your gums and still get your teeth clean, dentists recommend the "C-shape" technique.

1.Measure and wrap:Use about 18 inches of floss.

Wind the majority of the floss lightly around the middle fingers of both hands, leaving about 1 to 2 inches of taut floss in the middle to work with.

2.Guide gently:Never snap.

Pinch the floss between your thumbs and index fingers. Gently slide it up and down between the teeth using a subtle zigzag motion. If the teeth are tight, work it through slowly—do not force or snap it.

3.Form a 'C' shape:The most critical step to prevent damage.

Once the floss reaches the gum line, curve it into a "C" shape against the side of one tooth. This wraps the floss around the tooth rather than pointing it down into the gums.

4.Slide under the gumline:

Gently slide the floss into the space between the gum and the tooth until you feel light resistance.

5.Rub up and down:

Move the floss up and down against the side of the tooth to scrape the plaque away. Do not saw back and forth. Repeat this on the adjacent tooth before removing the floss.

Note on bleeding: If your gums bleed when you floss gently, it's usually a sign of early gingivitis (inflammation from plaque buildup), not a sign that you are flossing too hard. With consistent, gentle flossing, the bleeding should stop within a week or two.

Q17 Do electric toothbrushes damage the gums more than regular toothbrushes?

The short answer is **no**—in fact, electric toothbrushes are generally *safer* for your gums than manual toothbrushes.

The primary cause of toothbrush-related gum damage (like gum recession and enamel wear) isn't the type of brush you use, but **how hard you brush**.

Here is why electric toothbrushes actually help protect your gums:

Why Electric Brushes are Gentler

- **Built-in Pressure Sensors:** Many modern electric toothbrushes have sensors that flash a light or slow down the motor if you press too hard against your teeth.This actively trains you to use a lighter touch.
- **They Do the Work:** Because the brush head is already oscillating or vibrating at thousands of strokes per minute, you don't need to use a vigorous, back-and-forth "sawing" motion.
- **Better Disease Prevention:** Clinical studies consistently show electric brushes remove significantly more plaque than manual ones. Less plaque means less gingivitis (gum inflammation), and healthy, inflammation-free gums are far less likely to recede.

The Risk With Manual Brushes

With a manual toothbrush, people often overcompensate to feel like they are getting their teeth clean. They grip the handle tightly and scrub vigorously. Over time, this aggressive physical friction can literally wear away the delicate gum tissue, exposing the roots of the teeth.

You can still damage your gums with an electric toothbrush, but usually only if you make the mistake of using it like a manual one.To protect your mouth:

- **Hold at a 45-degree angle:** Aim the bristles slightly toward the gumline where plaque builds up.
- **Don't scrub:** Let the brush rest on each tooth for a few seconds, then simply glide it to the next one.
- **Stick to soft bristles:** Always buy "soft" replacement heads. Medium or hard bristles will damage your gums regardless of the power source.

Q18 Would using mouthwash alone be enough without brushing my teeth?

This text is for informational purposes only. Consult a specialist for medical advice or diagnoses.

Absolutely not. Using mouthwash alone is not a substitute for brushing your teeth.

Think of plaque like the baked-on grease on a dirty frying pan. If you just pour soapy water over the pan and rinse it out, the sticky grime won't go anywhere. To actually get the pan clean, you need to scrub it with a sponge.

Your teeth work the exact same way.

The Problem with Plaque

The main enemy of healthy teeth and gums is dental plaque, which is a sticky, complex film of bacteria that constantly forms on your teeth.

- Brushing provides mechanical friction: The physical scrubbing action of toothbrush bristles is the only way to physically break up and sweep away that sticky biofilm.
- Mouthwash provides chemical action: Mouthwash can kill some bacteria and leave your breath minty, but it cannot physically dislodge plaque that has adhered to the tooth's surface.

If you don't mechanically remove that plaque, it hardens into tartar (calculus) within a few days. Once tartar forms, no amount of brushing or mouthwash will remove it—it has to be scraped off by a dental professional.

What is mouthwash actually good for?

While it can't replace your toothbrush, mouthwash is a great addition to a good hygiene routine. When used alongside brushing and flossing, a therapeutic mouthwash can:

- Reach hidden spots: Liquid can wash into microscopic crevices and the back of your throat where a brush can't reach.
- Strengthen enamel: Fluoride mouthwashes help remineralize teeth and prevent cavities.
- Reduce inflammation: Antibacterial rinses can help keep gingivitis and gum inflammation at bay.

The bottom line: Brushing and flossing are the foundation of your oral health. Mouthwash is just the sealant that supports that foundation.

Q19 After I quit smoking, my gums started bleeding. Is that normal?

This text is for informational purposes only. Consult a specialist for medical advice or diagnoses.

First off, congratulations on quitting smoking—that is a massive achievement. To answer your question directly:yes, this is completely normal.In fact, it is so incredibly common that dental professionals sometimes refer to it as the "cessation paradox."

While seeing blood when you brush is definitely alarming, it is actually a sign that your body is finally starting to heal.

The "Masking" Effect of Nicotine

To understand why this is happening now, it helps to look at what cigarettes were doing to your mouth.Nicotine is a powerful vasoconstrictor, meaning it tightens and shrinks your blood vessels.For however long you smoked, it severely reduced the blood flow to your gum tissues.

Because your gums were constantly starved of blood, they couldn't swell or bleed—even if bacterial plaque was building up beneath the gumline.The smoking essentially "masked" the normal warning signs of gum irritation, keeping your gums looking artificially pale and quiet while an infection was brewing underneath.

Your Immune System is Waking Up

Now that you have quit, those blood vessels are dilating back to their normal size. A healthy rush of blood, oxygen, and immune cells is flooding back into your gum tissues.

Your newly awakened immune system is suddenly detecting the backlog of bacterial plaque and tartar that the nicotine kept hidden, and it is finally fighting back. The bleeding, soreness, and swelling you are experiencing right now is the collateral damage of that fight. It's not a sign that your oral health is getting worse; it's a sign that your body's alarm system is working again.

What You Should Do Next

- Don't let it trick you. Many people experience this bleeding, assume quitting ruined their teeth, and start smoking again. Your gums are just finally able to ask for help—don't mute the alarm again.
- See a dentist. Because regular toothbrush bristles can't reach the hardened tartar deep beneath the gumline, you will likely need a professional cleaning to clear out the bacterial backlog.Once a hygienist removes that tartar, your newly oxygenated gums can actually reattach and heal.
- Keep brushing and flossing. Be gentle, but don't avoid the bleeding areas. You need to keep the daily surface plaque at bay while your gums recover.

Q20 Can natural products effectively keep my gums healthy?

This text is for informational purposes only. Consult a specialist for medical advice or diagnoses.

Yes, natural products can be highly effective at keeping your gums healthy and reducing inflammation — but only when used as a supporting cast. Clinical studies show that certain plant-based compounds can significantly reduce plaque bacteria and soothe irritated tissue, but they cannot replace the mechanical action of brushing and flossing.

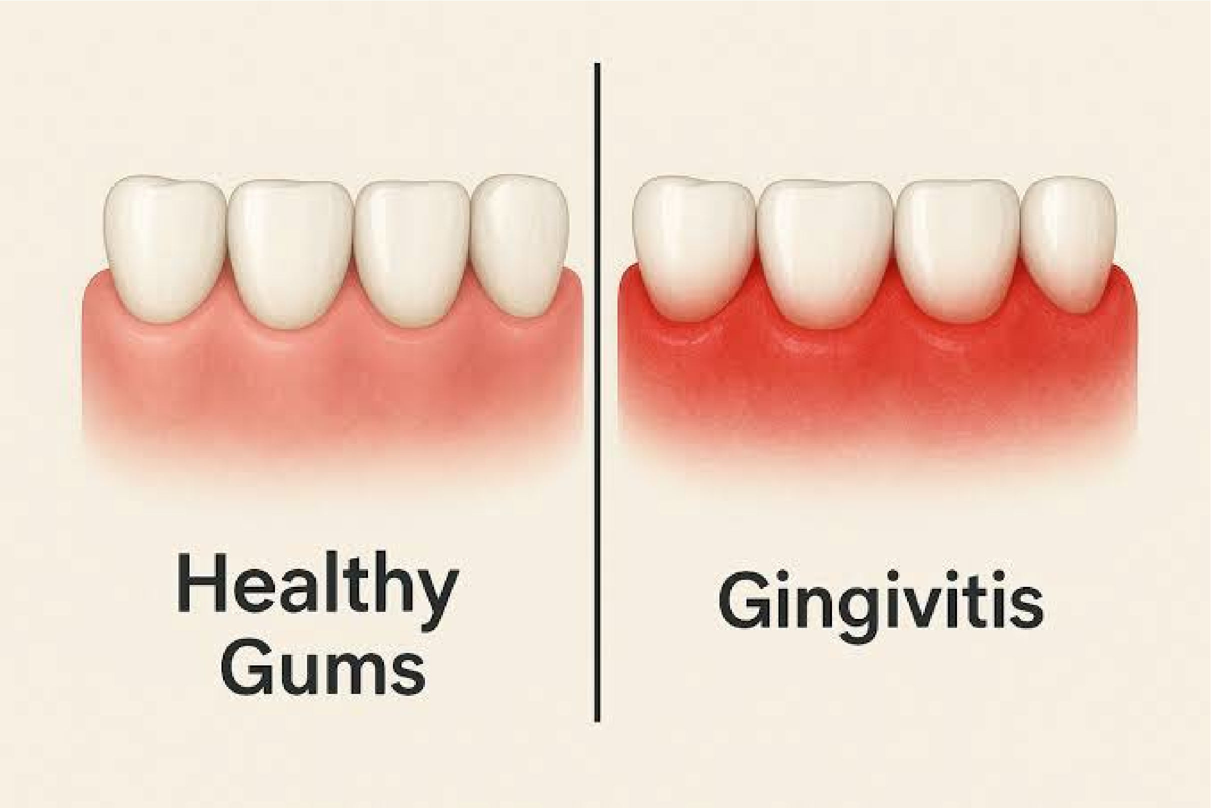

Science-Backed Natural Remedies

Here is a breakdown of the natural products with the strongest clinical evidence for gum health:

- **Green Tea:** Packed with antioxidants called **catechins**. Studies show that rinsing with a green tea extract mouthwash can reduce plaque and gum bleeding almost as effectively as traditional chlorhexidine (prescription) mouthwashes.

- **Turmeric (Curcumin):** Curcumin is a powerful anti-inflammatory and antimicrobial agent. Massaging a paste of turmeric and water onto the gums or using a curcumin mouthwash helps reduce swelling and prevents bacteria from multiplying.
- **Aloe Vera:** Known for healing skin, aloe vera gel is incredibly soothing for irritated gums. Clinical trials indicate that applying natural, alcohol-free aloe directly to the gums can significantly reduce bacteria and inflammation in people with gingivitis.
- **Oil Pulling (Sesame or Coconut Oil):** This ancient Ayurvedic practice involves swishing oil in the mouth for 15-20 minutes. The oil's high viscosity creates a barrier against plaque buildup, and the swishing mechanically traps toxins and bacteria so they can be spit out.
- **Sugar-Free Gum (with Xylitol):** Chewing xylitol-sweetened gum for 20 minutes after meals stimulates saliva flow by 10-12 times the normal rate. Saliva naturally washes away debris and neutralizes the acids that irritate gums. Xylitol specifically inhibits Streptococcus mutans, a primary plaque-causing bacterium.

The Non-Negotiable Foundation

Natural remedies are excellent for neutralizing bacteria and reducing inflammation, but they cannot physically dislodge the sticky biofilm (plaque) that forms on your teeth. If left alone, plaque hardens into tartar, which traps bacteria against the gum line and causes infection.

To prevent gum disease, your routine must still be anchored by:

1. **Mechanical Removal:** Brushing twice a day for two minutes to physically scrub the teeth.
2. **Interdental Cleaning:** Flossing daily to reach the 40% of the tooth surface your brush misses.
3. **Professional Cleanings:** Seeing a dentist to remove hardened tartar that neither brushes nor natural remedies can dissolve.

Q21 Can getting a dental scaling damage my teeth?

The short answer is no. When performed correctly by a trained dental professional, dental scaling will not damage your teeth, scratch your enamel, or harm your gums.In fact, it is one of the most important things you can do to *protect* them.

Because scaling involves metal tools and high-frequency vibrations, it's very common to worry that your teeth are being filed down. Here is a look at what is actually happening during the procedure.

Why People Think It Causes Damage (Myths vs. Facts)

The feeling of your mouth after a deep clean can be surprising, which leads to a few common misunderstandings:

- "It scrapes away my enamel."Fact: Tooth enamel is the hardest substance in the human body—harder than bone and harder than the steel tools used to clean them.Dental scalers (both the hand tools and the vibrating ultrasonic ones) are designed to break the chemical bonds of hard tartar (calculus), not to shave down your tooth.
- "It creates gaps between my teeth."Fact: Scaling doesn't create gaps; it reveals them. When tartar builds up over time, it fills in the natural spaces between your teeth. When the dentist removes that "cement," you are simply feeling the natural shape of your teeth again.

- "It makes my teeth loose."Fact: Heavy tartar buildup can sometimes act like a splint, artificially holding wobbly teeth together. If your teeth feel loose after a cleaning, it is because gum disease has eaten away at the underlying bone, not because the cleaning damaged the tooth. Removing the tartar is the only way to stop that bone loss from getting worse.

What You *Can* Expect

While scaling won't damage your teeth, it does temporarily change the environment in your mouth. It is completely normal to experience a few side effects for 1 to 3 days afterward:

- Temperature Sensitivity: Tartar acts like an insulating blanket over your teeth.When it's removed, newly exposed areas of the tooth might be sensitive to hot and cold drinks.
- Tender or Bleeding Gums: If your gums were inflamed and puffy from the bacteria trapped in the tartar, they might be a bit sore or bleed slightly when you brush. As the gums heal and the swelling goes down, they will tighten back up around the teeth.
- A "Rough" Feeling: Your teeth might feel slightly different or rough to your tongue immediately after, but the dentist usually finishes the appointment by polishing your teeth to leave them smooth and stain-free.

The real risk: The only minor risk involves patients with severe gum recession. The roots of your teeth are covered in a substance called *cementum*, which is softer than enamel. If you have exposed roots, an overly aggressive cleaning could make them sensitive, but your hygienist will adjust their technique to be gentler in those areas.

Q22 Can dental scaling cause gaps between my teeth?

It is completely understandable to be concerned if you notice new spaces in your smile after a dental cleaning. The short answer is no, dental scaling does not create gaps between your teeth — but it *can* reveal gaps that were already there.

Here is what is actually happening in your mouth when those spaces suddenly appear.

Why Gaps Appear After Scaling

There are a few main reasons you might suddenly feel or see spaces that weren't there before your appointment:

- Tartar was acting as "filler": Over time, plaque that isn't brushed away hardens into tartar (calculus). This hardened bacteria builds up along the gumline and physically fills the natural spaces between your teeth. When the dentist or hygienist scales that tartar away, the "filler" is gone, and you can finally feel the natural shape of your teeth.
- Swollen gums are shrinking: Tartar irritates your gums, causing them to become inflamed, puffy, and swollen. This swelling can temporarily mask the true gumline and make your teeth look snug.After a cleaning, your gums heal and the swelling goes down. As they return to their normal, healthy size, spaces that were hidden by puffy tissue become visible.
- Gum recession: If the tartar buildup caused gum disease (periodontitis), the bone and gum tissue supporting your teeth may have receded. The tartar and swollen gums hide this

damage.Once cleaned, you might notice small triangular spaces near the gumline (often called "black triangles").

Are these gaps permanent?

It depends on the cause. If the gaps are simply the natural anatomy of your teeth previously hidden by tartar, they will remain.

However, if the gaps look larger because your gums are still adjusting, they may actually shrink slightly.In the weeks following a deep cleaning, your gums will heal, firm up, and reattach more closely to the clean roots of your teeth.

A Sign of Health, Not Damage

While it can feel strange—your teeth might even feel slightly "looser" or sharper to your tongue because you are feeling the actual tooth enamel instead of a wall of tartar—these gaps are a sign that your mouth is healing.Leaving the tartar in place to hide the gaps would eventually lead to severe gum disease, bone loss, and even tooth loss.

Q23 Do all gum diseases require the same treatment?

No, they do not.The treatment for gum disease depends entirely on how far the condition has progressed.As the infection moves from the surface of the gums deeper into the tissue and bone, the required treatments shift from simple preventative care to advanced surgical procedures.

Here is how the treatment approach changes based on the stage of the disease:

1. Gingivitis (Early Stage)

Gingivitis is the mildest form of gum disease and the only stage that is completely reversible.At this point, the gums are inflamed and may bleed when you brush or floss, but the bone and deeper connective tissues are not yet affected.

- Professional Cleaning: A dentist or hygienist removes the plaque and tartar buildup on the surface of the teeth that is causing the irritation.
- Improved Home Care: Treatment relies heavily on the patient stepping up their daily oral hygiene—brushing twice a day and flossing daily to prevent plaque from returning.

2. Periodontitis (Moderate Stage)

If gingivitis is left untreated, it progresses into periodontitis.The gums begin to pull away from the teeth, forming "pockets" where bacteria collect and multiply. The bone holding the teeth in place starts to sustain irreversible damage. At this stage, the goal of treatment shifts from *curing* the disease to *managing* it and halting further damage.

- Scaling and Root Planing (SRP): Often referred to as a "deep cleaning." The dentist cleans deep beneath the gumline to clear out the pockets of bacteria (scaling) and smooths the root surfaces of the teeth (planing) so the gums can reattach.
- Medications: Prescription antimicrobial mouthwashes or topical antibiotics (often placed directly into the gum pockets) are used to help clear the infection.

- Laser Therapy: Sometimes used as a less invasive way to remove diseased tissue and kill bacteria inside the gum pockets.

3. Advanced Periodontitis (Severe Stage)

In the most severe stage, the infection has destroyed significant amounts of gum tissue and jawbone. Teeth may become loose, shift, or require extraction.Cases this advanced usually require the expertise of a periodontist (a gum specialist).

- Pocket Reduction Surgery (Flap Surgery): The gums are surgically folded back so the periodontist can access and clean the deep roots, then sutured snugly back in place to eliminate the deep pockets where bacteria hide.
- Bone Grafting: If the jawbone has been destroyed, bone grafts (using your own bone, donated bone, or synthetic materials) are placed to encourage new bone growth and stabilize the teeth.
- Soft Tissue Grafts:Tissue is taken from the roof of the mouth and stitched to the gumline to repair receding gums and cover exposed tooth roots.
- Tooth Extraction & Implants: If a tooth cannot be saved due to severe bone loss, it must be removed. Once the infection is cleared and the bone is rebuilt,dental implants can be placed to restore function.

Q24 Is gum treatment painful?

It is completely normal to worry about dental pain, but the short answer is:during the procedure, no. Afterward, you can expect some mild to moderate soreness.

Thanks to modern anesthetics and techniques, dentists can completely numb the area before they begin. What you feel afterward depends heavily on the specific type of treatment you receive.

During the Procedure

Whether you are getting a routine deep cleaning or minor gum surgery, your dentist will use a local anesthetic to numb your gums and teeth.

- What you will feel: Pressure, vibration, and you might hear scraping sounds as plaque and tartar are removed from below the gumline.
- What you shouldn't feel: Sharp pain. If you do feel a pinch or a "zing," signal your dentist immediately so they can apply more numbing agent.

After the Procedure (Recovery)

Once the numbness wears off, your discomfort level will depend on the exact treatment:

Treatment	Expected Pain Level	Common Symptoms	Typical Duration
Deep Cleaning (Scaling & Root Planing)	Mild	Tender gums, tooth sensitivity to hot/cold	Soreness fades in 3–5 days; sensitivity may last a week
Periodontal Surgery (Pocket reduction, grafting)	Mild to moderate	Swelling, throbbing, minor bleeding	Discomfort peaks at 48–72 hours; the acute phase lasts ~1 week

Managing the Discomfort

The soreness is easily managed at home and rarely disrupts daily life if you follow a few basic steps:

- Pain relievers: Over-the-counter medications like ibuprofen or acetaminophen are usually all you need. Take your first dose *before* the anesthetic fully wears off so you stay ahead of the pain.
- Saltwater rinses: After the first 24 hours, gently rinsing with warm saltwater (¼ teaspoon salt in 8 ounces of water) reduces swelling, flushes out bacteria, and soothes the tissue.
- Soft diet: Stick to foods that require minimal chewing — like yogurt, smoothies, mashed potatoes, and lukewarm soups — for the first few days.Avoid spicy, acidic, or crunchy foods (like chips or seeds) that can poke and irritate the healing gums.

If you are putting off treatment because of fear, let your dentist know. Many practices offer anti-anxiety options like nitrous oxide (laughing gas) or oral sedation to help you stay relaxed in the chair. Delaying treatment usually allows the gum disease to worsen,which can lead to more invasive (and uncomfortable) procedures down the road.

Q25 Can gum surgery cause tooth loss?

The short answer is no—gum surgery itself is rarely the direct cause of tooth loss. In fact, periodontal surgeries are usually performed as a last line of defense to prevent you from losing teeth to advanced gum disease.

However, it is a common concern because teeth are sometimes still lost around the time of a surgical procedure. Here is why that can happen:

1. The Disease Was Too Advanced

Gum surgery relies on having enough healthy bone and tissue left to work with.If periodontal disease has already destroyed too much of the underlying jawbone, the tooth may lack the structural support it needs to stay anchored. In these cases, the surgery didn't cause the tooth loss; the disease had simply progressed too far for the surgery to save it.

2. Post-Surgical Infections or Graft Failures

Like any procedure, gum surgery carries a risk of complications.If bacteria enter the surgical site and cause a severe infection, or if a gum graft fails to attach and loses its blood supply, it can cause further damage to the surrounding tissue and bone.If left untreated, this progressive damage could eventually put the tooth at risk.

3. Intentional or Unintentional Gum Recession

Some types of periodontal surgery (like "pocket reduction" surgery) involve cleaning out deep bacterial pockets and reshaping the gums.As the gums heal and the swelling goes down, the gumline often rests lower than it did before, exposing more of the tooth root.While this recession doesn't cause the tooth to fall out, exposed roots are highly vulnerable to decay.If that decay isn't managed, it can eventually lead to tooth loss down the road.

How to Protect Your Teeth

If you are getting gum surgery to save a tooth, the best way to ensure a successful outcome is to strictly follow your periodontist's aftercare instructions:

- Do not smoke or vape during recovery, as nicotine severely restricts blood flow to the gums and is a leading cause of surgical failure.
- Eat soft foods to avoid physically disturbing the surgical site or stitches.
- Watch for signs of infection, such as persistent swelling, pus, a bad taste in your mouth, or pain that worsens after the first few days.

Q26 Can gum treatment make my teeth feel loose?

Yes, it is completely normal and very common for teeth to feel a bit loose after gum treatments like a deep cleaning (scaling and root planing) or periodontal surgery. While it can be an unsettling feeling, it is usually a temporary and expected part of the healing process.

Why This Happens

There are three main reasons your teeth might feel mobile right now:

- The "Tartar Splint" is Gone: Over time, hardened plaque (tartar or calculus) builds up around your teeth and below the gumline. In cases of advanced gum disease, this heavy buildup can act like cement, artificially splinting compromised teeth together. When your dentist removes this hard buildup, your teeth lose that rigid outer shell, which can suddenly make them feel loose.
- Temporary Inflammation: Gum treatment involves cleaning deep below the gumline, which temporarily irritates and stretches the periodontal ligament (the fleshy, shock-absorbing tissue that anchors your tooth root to the jawbone). When this ligament is swollen from the procedure, the tooth has more room to wiggle in its socket.
- Revealing Underlying Damage: Gum disease (periodontitis) eats away at the underlying bone that holds your teeth in place. The tartar and severe gum swelling may have been masking this existing bone loss. Once the area is thoroughly cleaned, you are feeling the true, unmasked state of the tooth's support system.

What to Expect Next

The good news is that this looseness is usually temporary. As your gums heal over the next few weeks, the inflammation will subside. The gum tissue will shrink, become healthier, and reattach more tightly around the roots of your teeth, providing a much firmer grip.

When to call your dentist: While mild to moderate looseness is normal during the healing phase, you should reach out to your dental office if a tooth feels like it might actually fall out, if you are experiencing severe pain, or if the mobility doesn't start to improve after two to three weeks.

Q27 Does tartar removal cause more tartar buildup?

No, tartar removal does not cause your teeth to build up more tartar. In fact, leaving tartar on your teeth is exactly what accelerates new buildup.

This is one of the most common dental myths. Here is a breakdown of why people believe this misconception, the actual biology of tartar, and why getting it removed is essential.

Where the Myth Comes From

It is easy to see why people feel their teeth get "dirtier" faster after a cleaning. Several physical sensations trick us into believing the cleaning caused a problem:

- **The "New Spaces" Illusion:** Over time, tartar (calculus) acts like cement, filling in the natural gaps between your teeth and along the gumline. When a hygienist removes it, your tongue suddenly feels those empty spaces. Many people mistake this natural tooth anatomy for "damage" or worry that these newly exposed gaps will trap more food and plaque.
- **The "Scratched Enamel" Fear:** A common fear is that metal dental scrapers scratch the tooth enamel, leaving a rough surface that catches more plaque. In reality, tooth enamel is the hardest substance in the human body. Furthermore, the **polishing** step at the end of a cleaning (using a gritty paste) is specifically designed to smooth out the tooth surface, making it *harder* for bacteria to stick.
- **The 24-Hour Plaque Cycle:** Plaque—the sticky, colorless film of bacteria—begins forming on your teeth just hours after a professional cleaning. Because your teeth were perfectly smooth when you left the clinic, the contrast of that new "fuzzy" plaque layer feels exaggerated. People often blame the cleaning, but the rapid return of plaque is just natural oral biology.

Why Leaving Tartar Actually *Causes* More Tartar

Tartar is calcified plaque that has absorbed minerals (like calcium and phosphate) from your saliva.It has a highly porous, rough surface—much like a microscopic coral reef.

If you don't remove it:

1. **It acts as a magnet:** The rough surface of existing tartar makes it incredibly easy for new, sticky bacterial plaque to anchor itself, multiply, and harden.
2. **It destroys the foundation:** The bacteria living inside tartar release toxins that trigger your immune system. This causes persistent inflammation (gingivitis), which eventually destroys the bone holding your teeth in place (periodontitis).
3. **It causes gum recession:** As tartar builds up along and below the gumline, it physically pushes the gums away from the teeth, creating deep pockets where even more bacteria can hide.

How to Slow Tartar's Return

Because plaque begins mineralizing into tartar in as little as **24 to 72 hours**, what you do at home between cleanings dictates how much tartar builds up.

- **Target the "Tartar Zones":** Your salivary glands pump mineral-rich saliva directly onto the **inside of your lower front teeth** and the **outside of your upper back molars**. Because these areas are flooded with calcium, plaque hardens here first. Spend extra time brushing these specific spots.
- **Disrupt the biofilm daily:** Once plaque hardens into tartar, a toothbrush cannot remove it. You have a narrow 24-to-48-hour window to brush and floss away soft plaque before it crystallizes into tartar.
- **Address dry mouth:** Saliva naturally washes away food and neutralizes acid.If you take medications that cause dry mouth or sleep with your mouth open, plaque becomes thicker, stickier, and hardens much faster. Drinking plenty of water and using dry-mouth rinses can slow this down.

Q28 Are antibiotics enough to treat gum disease?

While they can be a helpful supporting tool in certain severe situations, they are never a substitute for physical, professional dental treatment.

Think of tartar on your teeth like barnacles on a boat.Pouring chemicals into the water won't remove the barnacles; they have to be physically scraped off.

Why Medication Isn't a Cure-All

Gum disease (periodontal disease) is caused by bacteria that build up on your teeth and under your gumline. Here is why popping a pill won't solve the core problem:

- The Biofilm Shield: The bacteria in your mouth live in a sticky substance called plaque (a biofilm). This film acts like a protective fortress, making it highly resistant to antibiotics and preventing the medication from actually reaching all the bacteria inside.
- Hardened Tartar: If plaque isn't brushed and flossed away, it hardens into calculus (tartar). Antibiotics cannot dissolve or break down these rock-hard deposits. As long as the physical tartar remains under your gums, the irritation and infection will keep returning the moment you finish your prescription.

The Gold Standard: Mechanical Cleaning

The foundation of all gum disease treatment is physically removing the source of the infection.

This process is called scaling and root planing (often referred to as a "deep cleaning").A dentist or hygienist uses specialized tools to scrape away the tartar above and below the gumline. They then smooth out the roots of your teeth so bacteria have a much harder time clinging to them in the future.

When Are Antibiotics Actually Used?

For most mild to moderate cases (like gingivitis), a professional cleaning and excellent at-home care are all that's needed. However, your dentist might prescribe antibiotics *in addition* to a deep cleaning if:

- You have an aggressive or rapidly progressing form of periodontitis.
- You develop an acute, painful gum abscess.
- Your gum pockets remain deep and inflamed even after a standard deep cleaning.

When they are used, dentists increasingly prefer topical antibiotics. Rather than giving you a pill that travels through your whole bloodstream, they place antibiotic gels or powders directly into the infected gum pockets immediately after a deep cleaning. This delivers a concentrated dose exactly where the bacteria thrive while the physical barrier (tartar) is out of the way.

Q29 After gum disease treatment, will I ever need treatment again?

The short answer is yes — but it is more helpful to think of it as ongoing management rather than starting from scratch.Gum disease (periodontitis) is a chronic inflammatory condition.Like managing high blood pressure or diabetes, your initial treatment gets the disease under control, but it requires lifelong maintenance to keep it from returning.

Why the Infection Tries to Return

Your initial treatment — usually a deep cleaning known as scaling and root planing, or sometimes surgery — clears out the harmful bacteria, plaque, and tartar trapped deep below your gum line.It halts the bone loss and allows your gums to heal.

However, the bacteria that cause gum disease naturally live in your mouth. Within hours of brushing, they begin forming plaque again.Once you have had gum disease, the spaces between your teeth and gums (called **periodontal pockets**) are often deeper than normal.

If a pocket is deeper than 4 millimeters, it is physically impossible for a toothbrush or dental floss to reach the bottom.Without professional help, bacteria will hide in those deep spaces, multiply, and trigger inflammation and bone loss all over again.

The 90-Day Rule: Periodontal Maintenance

To prevent the disease from coming back, your dentist will shift you from a standard six-month cleaning schedule to **Periodontal Maintenance**.This is a specialized, targeted cleaning that focuses on those vulnerable deep pockets.

- **The Timeline:** You will usually need these visits every **3 to 4 months**.
- **The Science:** Clinical research shows it takes about 80 to 90 days for the bacteria trapped in deep pockets to reproduce and organize into destructive, bone-eating colonies.
- **The Goal:** By professionally cleaning your pockets right at the three-month mark, your hygienist disrupts the bacteria's life cycle before they can cause new damage.

If you stick to this schedule, you can often avoid ever needing the more invasive, expensive initial treatments (like another deep cleaning or gum surgery) again.

How to Protect Your Results

While your dental team handles what you can't reach, your daily habits dictate whether the disease stays dormant:

- **Stringent Home Care:** Brushing twice a day (angling the bristles toward the gum line) and cleaning between your teeth daily with floss, interdental brushes, or a water flosser is non-negotiable.
- **Control Outside Factors:** Smoking and uncontrolled diabetes drastically increase the risk of gum disease recurring. Quitting tobacco and managing blood sugar are two of the best things you can do for your gums.
- **Watch for Warning Signs:** If your gums bleed when you brush, become swollen or tender, or if you develop persistent bad breath, do not wait for your next appointment. These are early signs the bacteria are overwhelming your system again.

Q30 Is surgery always necessary for gum treatment?

This text is for informational purposes only. Consult a specialist for medical advice or diagnoses.

No, surgery is not always necessary for gum treatment. In fact, non-surgical approaches are considered the "gold standard" for early-to-moderate gum disease.Surgery is typically only recommended when the disease has advanced to a point where bone and deep tissues are severely compromised.

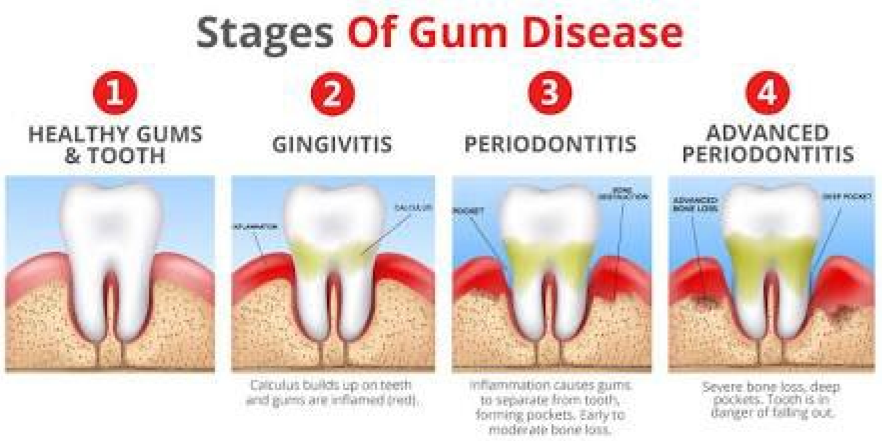

The treatment your dentist or periodontist recommends will depend entirely on how far the infection has progressed.

Non-Surgical Treatments

If caught in the early stages (gingivitis or mild periodontitis), gum disease can usually be managed and even reversed without a scalpel.

- **Scaling and Root Planing (SRP):** This is essentially a "deep cleaning." Scaling removes the hardened plaque (tartar) and bacteria from above and below your gumline. Root planing smooths out the rough spots on the roots of your teeth, removing bacterial toxins and giving your gums a clean surface to reattach to.
- **Antibiotics:** Often used alongside SRP, these can be oral medications or topical antibiotic gels and chips placed directly into the gum pockets to fight stubborn bacteria over time.

- **Laser Therapy:** A modern, minimally invasive alternative to traditional deep cleaning.Lasers use focused light energy to remove inflamed tissue and bacteria from the periodontal pocket, often with less discomfort and faster healing times than traditional methods.

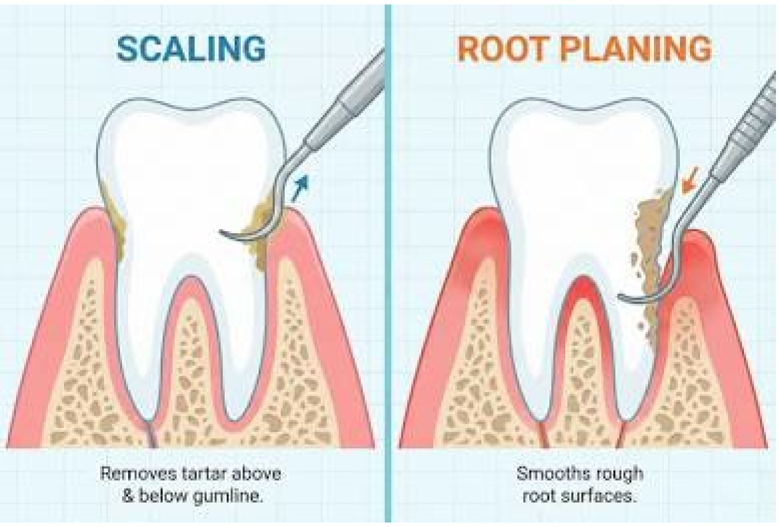

When Surgery Becomes Necessary

Surgical intervention is usually required when gum pockets become extremely deep (typically greater than 6 to 7 millimeters) or when there is significant bone and tissue loss. At this stage, non-surgical instruments simply cannot reach the base of these deep pockets effectively to clean out the infection.

Surgical options for advanced periodontitis include:

- **Flap Surgery (Pocket Reduction):**The gums are temporarily folded back to give the periodontist direct access to clean deep tartar deposits and smooth irregular bone before suturing the tissue back snugly around the tooth.
- **Bone Grafts:** If the disease has destroyed the bone supporting your teeth, natural or synthetic bone fragments are placed to stimulate new bone growth and save the tooth.
- **Soft Tissue Grafts:** To fix receding gums, tissue is taken from another part of your mouth (like the palate) and attached to the exposed roots to protect them and reduce sensitivity.

The takeaway: If your dentist suggests treating your gum disease, it will almost always start with a non-surgical deep cleaning. Catching it early and maintaining great oral hygiene at home is the best way to keep surgery off the table.
