# Supplementary material for: Comparative Evaluation of Large Language Models in Responding to Common Misconceptions Regarding Periodontal Disease and Oral Hygiene
Source: Diagnostics (Basel). 2026 Sep 28;16(19):3158. doi: 10.3390/diagnostics16193158 (PMC13664004; doi:10.3390/diagnostics16193158)
Supplement: Supplementary file 1 [file diagnostics-16-03158-s001.zip › Supplementary File S4 LLMs response unprocessed.zip!/2. LLMs response/Grok 4.3.pdf]

Q1 Do gum diseases only affect elderly people?

No, gum diseases do not only affect elderly people. They can occur at any age, though prevalence and severity generally increase with age.

Key Facts on Prevalence

- **Children and teens**: Gingivitis (the mild, reversible early stage) is very common. Studies show many children have some form of periodontal issues, often linked to poor oral hygiene, plaque buildup, puberty-related hormonal changes, or conditions like mouth breathing. More serious forms like aggressive periodontitis are rarer but can affect teens and young adults.
- **Young adults**: Gum disease is not uncommon in people in their 20s and 30s. Globally, it affects a significant portion of young adults, with rates varying by region and risk factors.
- **Adults 30+**: About 40-42% of U.S. adults aged 30 and older have some level of periodontitis (the more advanced form involving bone and tissue damage). It's more common in men than women.
- **Older adults (65+)**: Rates are highest here — around 60% or more have periodontitis — due to cumulative effects of plaque, medical conditions (e.g., diabetes), medications causing dry mouth, reduced dexterity for cleaning, and other factors. However, it's not an inevitable part of aging.

Why It Affects All Ages

Gum disease (primarily gingivitis and periodontitis) is caused mainly by bacterial plaque and tartar buildup from inadequate brushing/flossing. Risk factors that apply across ages include:

- Poor oral hygiene
- Smoking/tobacco use
- Diabetes and other systemic conditions
- Genetics
- Hormonal changes (e.g., puberty, pregnancy)
- Certain medications or dry mouth

It is preventable and often treatable with good hygiene, regular dental care, and addressing risk factors. Early signs like bleeding gums can appear even in young people.

If you're experiencing symptoms (red/swollen/bleeding gums, bad breath, loose teeth), see a dentist regardless of your age — early intervention is key. Maintaining consistent oral care benefits everyone.

Q2 **Is it normal if my gums bleed when I brush my teeth?**

No, it's not normal. Healthy gums are firm, pink, and shouldn't bleed during regular brushing or flossing.

Bleeding gums are one of the most common early signs of **gingivitis** (the mildest form of gum disease), which is usually caused by plaque buildup from inadequate brushing and flossing. Other frequent causes include:

- Brushing too hard or with a hard-bristled toothbrush.
- Not flossing regularly (which can irritate gums when you start).
- Hormonal changes (e.g., pregnancy, puberty).
- Certain medications (like blood thinners).
- Less commonly, vitamin deficiencies, smoking, or other health conditions.

What you can do

- **Improve your routine**: Brush gently twice a day with a soft-bristled toothbrush (using circular motions), floss daily, and consider an antiseptic mouthwash. Bleeding often improves within 1–2 weeks of consistent better hygiene as inflammation decreases.
- **Switch tools if needed**: Use a soft toothbrush and replace it every 3 months.
- **See a dentist**: This is important. Persistent bleeding should be checked professionally, as untreated gingivitis can progress to periodontitis (more serious gum disease that can lead to tooth loss). Your dentist can clean tartar, assess your gums, and rule out other issues.

Don't ignore it—even if it's mild or only occasional—because early action usually reverses the problem easily. If the bleeding is heavy, doesn't stop, or you have other symptoms (swollen/painful gums, bad breath, loose teeth), get it evaluated sooner. This isn't medical advice; a dental professional can give personalized guidance. Keep up the good work on oral care!

Q3 Can I get gum disease even if I brush my teeth regularly?

Yes, you can still get gum disease (gingivitis or periodontitis) even if you brush your teeth regularly. Brushing is one of the best defenses against it, but it's not always enough on its own.

Why This Happens

Gum disease is primarily driven by **plaque**—a sticky film of bacteria that forms on teeth. If plaque isn't fully removed, it can irritate gums (gingivitis) and eventually lead to deeper infection and bone loss (periodontitis). Regular brushing helps a lot, but several factors can still allow problems to develop:

- **Incomplete plaque removal**: Brushing might miss areas (especially between teeth or below the gumline). Even careful brushing doesn't always eliminate every trace of bacteria, and plaque can harden into tartar (calculus) that only a dentist or hygienist can remove.
- **Technique and habits**: Brushing too lightly, too hard, for less than two minutes, or without flossing limits effectiveness. Hard brushing can actually irritate gums and contribute to recession.
- **Other risk factors** (many unrelated to daily brushing):
    - **Genetics**: Some people have an immune system that overreacts to bacteria, making them more susceptible even with good hygiene.
    - **Smoking or tobacco use**: A major risk that impairs healing and increases severity.

- Medical conditions: Diabetes, autoimmune diseases, obesity, or conditions that weaken immunity.
   - **Hormonal changes**: Pregnancy, menopause, etc.
   - **Medications**: Those causing dry mouth or gum changes.
   - **Stress, poor diet, or age**.

How to Lower Your Risk Further

- **Brush effectively**: Twice a day for at least two minutes with a soft toothbrush (consider electric). Use fluoride toothpaste.
- **Floss or use interdental cleaners daily**: This reaches spots brushing misses.
- **See a dentist regularly**: Professional cleanings (every 6–12 months, or more often if at higher risk) remove tartar and catch early signs. Annual checkups help.
- **Lifestyle steps**: Quit smoking, manage conditions like diabetes, eat a balanced diet low in sugars, and address stress.

Early gum disease (gingivitis) is often reversible with good care, but advanced periodontitis can lead to tooth loss if untreated. If you notice bleeding gums, swelling, bad breath, or recession, see a dentist promptly—even with solid brushing habits.

In short, consistent brushing is excellent and protective for most people, but combining it with flossing, professional care, and managing other risks gives the best protection. Consult your dentist for personalized advice.

Q4 Can I have gum disease even if my gums don't hurt?

Yes, you can have gum disease (gingivitis or periodontitis) even if your gums don't hurt. In fact, it's very common for it to progress without pain, especially in the early stages.

Why Gum Disease Is Often "Silent"

Gum disease starts with plaque buildup that causes inflammation. In **gingivitis** (the mild, reversible stage), you might notice red, swollen, or bleeding gums when brushing/flossing, but many people feel little to no discomfort. As it advances to **periodontitis**, it can damage the bone and tissues supporting your teeth—again, often without significant pain until later stages (e.g., loose teeth or painful chewing).

Reliable sources like the Cleveland Clinic, Mayo Clinic, CDC, and NIDCR emphasize this:

- "Periodontal disease is usually painless, especially in the early stages."
- "Gum disease is often painless and without noticeable symptoms."
- It can become serious before you notice symptoms, which is why regular dental visits are key.

Common Signs (Even Without Pain)

Look for these instead of relying on discomfort:

- Bleeding gums (when brushing, flossing, or eating)

- Red, swollen, or puffy gums
- Persistent bad breath
- Receding gums (teeth looking longer)
- Tender gums when touched
- Changes in how your teeth fit together or loose teeth (later stages)

What to Do

- **See a dentist regularly** (at least twice a year) for professional cleanings and checkups. They can measure gum pockets and detect issues early with tools like probing, even if nothing hurts.
- Maintain good oral hygiene: Brush twice daily, floss daily, and use an antiseptic mouthwash if recommended.
- Risk factors that can worsen it silently include smoking, diabetes, genetics, certain medications, and poor nutrition.

I'm not a doctor, and this isn't medical advice. If you're concerned about your gums or have any risk factors, consult a dentist or periodontist for a proper evaluation—they're the best way to know for sure. Early treatment is straightforward and can prevent bigger problems like tooth loss.

Q5 Can gum disease get better without treatment?

No, gum disease generally does not get better without treatment, though early-stage cases (gingivitis) can sometimes reverse with improved oral hygiene. Advanced stages (periodontitis) cannot be reversed and require professional care to manage and prevent worsening.

Key Distinctions: Gingivitis vs. Periodontitis

Gum disease exists on a spectrum:

- **Gingivitis** (early, reversible stage): Caused by plaque buildup leading to inflamed, red, swollen gums that may bleed when brushing or flossing. No bone loss yet. This stage is often fully reversible with consistent brushing, flossing, and professional dental cleanings. Symptoms can improve within days to weeks of better hygiene.
- **Periodontitis** (advanced stage): Develops if gingivitis is untreated. It involves deeper infection, gum recession, pocket formation, and irreversible bone/tissue loss around teeth. This cannot be cured or reversed—only managed to slow or stop progression and prevent tooth loss.

Can It Improve on Its Own?

- **Rarely or not reliably.** While mild gingivitis symptoms might temporarily ease due to the body's immune response or minor hygiene changes, it usually persists or worsens without intervention. Untreated gingivitis frequently progresses to periodontitis.
- Periodontitis does **not** resolve spontaneously; it can have periods of stability but tends to advance over time, potentially leading to loose teeth or tooth loss.

Reliable sources like the Cleveland Clinic, Mayo Clinic, CDC, and ADA emphasize that professional evaluation and treatment (e.g., scaling, root planing) combined with daily hygiene are essential for reversal in early cases or control in later ones.

What Helps (and When to See a Dentist)

Improved home care—brushing twice daily, daily flossing, antimicrobial mouthwash—can support reversal of gingivitis and help manage periodontitis, but it's usually not enough alone for full resolution, especially if tartar (calculus) has built up below the gumline.

See a dentist promptly if you notice bleeding gums, persistent bad breath, receding gums, loose teeth, or pain. Early action greatly improves outcomes and can prevent systemic health links (e.g., to heart disease or diabetes).

In summary, don't rely on it improving without help—gum disease is progressive, and timely professional care is the most reliable path to better gum health.

Q6 Are gum diseases rare?

No, gum diseases are not rare—they are very common worldwide.

Gum (periodontal) diseases, which include milder forms like gingivitis and more advanced periodontitis, affect a large portion of the global population. They are among the most prevalent chronic inflammatory conditions.

Key Statistics

- Globally: Severe periodontal diseases affect over 1 billion people (around 11–19% of adults, depending on the study and severity). Oral diseases overall impact nearly 3.7 billion people.
- United States: About 42% (roughly 4 in 10) of adults aged 30+ have some form of periodontitis (mild, moderate, or severe). This rises to ~60% for those 65+. Men are more affected (~50%) than women (~35%).
- Broader estimates suggest periodontal diseases affect 20–50% or more of the global population in various forms, with prevalence increasing with age.

Milder gum inflammation (gingivitis) is even more widespread and often reversible with good oral hygiene, while untreated cases can progress to periodontitis, which can lead to tooth loss and has links to systemic health issues (e.g., cardiovascular disease, diabetes complications).

Why So Common?

Risk factors include poor oral hygiene, tobacco use, age, genetics, stress, certain medical conditions (like diabetes), and socioeconomic factors. Many cases go unnoticed in early stages because symptoms can be subtle.

Bottom line: Gum disease is a major public health issue, not a rare condition. Regular brushing, flossing, dental check-ups, and avoiding tobacco can prevent or manage it effectively for most people. If you have symptoms like bleeding gums, swelling, or bad breath, see a dentist promptly.

Q7 Is it normal to lose teeth because of gum disease as you get older?

No, it's not "normal" or inevitable to lose teeth from gum disease as you age, even though it becomes more common. Tooth loss in adults is usually a sign of underlying issues like untreated periodontal (gum) disease rather than aging itself.

Why It Happens More Often with Age

- **Gum disease (periodontitis)** is the leading cause of tooth loss in adults. It starts with plaque buildup causing inflammation (gingivitis), then progresses to destroy the gums, bone, and tissues supporting the teeth if untreated. This leads to loose teeth and eventual loss.
- Age-related factors increase risk: Plaque and tartar accumulate over decades, gums may naturally recede (exposing roots to more decay and infection), saliva production often decreases (making it easier for bacteria to thrive), and conditions like arthritis or medications can make daily brushing/flossing harder.
- **Statistics**: About 64% of U.S. adults 65+ have moderate or severe periodontal disease. Complete tooth loss (edentulism) affects around 13% of adults 65+ (higher in those 75+, around 17-18%), though this has declined over time thanks to better dental care. Many older adults keep most or all of their teeth.

Tooth loss is largely preventable with consistent oral hygiene and professional care. It's not a normal part of aging like gray hair—it's the result of cumulative damage that can often be stopped or managed.

Prevention and Management

- **Daily habits**: Brush twice a day with fluoride toothpaste, floss daily, and use an antimicrobial mouthwash. Regular dental cleanings (every 3-6 months, especially if you have gum issues) are key.
- **See a dentist promptly**: Early gum disease is often painless and silent, so routine checkups catch it before major damage. Treatments include deep cleanings (scaling/root planing), antibiotics, or surgery in advanced cases.
- **Lifestyle factors**: Quit smoking (a major risk), manage diabetes or other conditions, eat a balanced diet, and stay hydrated.
- If teeth are lost, options like implants, bridges, or dentures can restore function and prevent further issues (like shifting teeth or bone loss).

Losing teeth can affect nutrition, speech, appearance, confidence, and even link to broader health risks like heart disease, diabetes complications, or cognitive decline. Maintaining good oral health supports overall well-being. Consult a dentist or periodontist for personalized advice—they can assess your specific risk and create a plan. It's never too late to improve habits and protect your teeth.

Q8 If gum disease runs in my family, will I definitely get it too?

No, you will not definitely get gum disease even if it runs in your family. Genetics can increase your risk or susceptibility, but it is not a guarantee—periodontal (gum) disease is multifactorial, involving a combination of genetic predisposition, bacterial exposure, lifestyle, and other environmental factors.

Genetic Component

- Studies estimate that genetics play a role in roughly 30% to as much as 50% of cases, with heritability estimates around that range (higher for more severe forms).
- Up to about 30% of the population may have some genetic susceptibility. Certain gene variants (e.g., related to interleukin-1/IL-1, immune response, or inflammatory pathways) can make your gums react more aggressively to plaque bacteria, raising the odds of developing gingivitis progressing to periodontitis.
- Aggressive or early-onset forms of periodontitis often show stronger familial patterns and can run in families.

A family history is a known risk factor (per the American Dental Association), but it's one of many.

Why It's Not Inevitable

- **Lifestyle and modifiable factors dominate for most people**: Poor oral hygiene, smoking/tobacco use, diabetes, obesity, stress, certain medications, hormonal changes, poor nutrition, and dry mouth are major contributors. Good habits can often overcome genetic risk.
- Many people with a family history never develop significant problems if they maintain excellent care, while others without family history do due to environmental triggers.
- Genetic predisposition doesn't mean automatic disease—it's more like increased vulnerability that requires a "bacterial challenge" (plaque buildup) to manifest.

What You Can Do to Lower Your Risk

- **Daily habits**: Brush twice daily (with fluoride toothpaste), floss or use interdental cleaners daily, and consider an antimicrobial mouthwash.
- **Professional care**: See a dentist regularly (every 3–6 months if at higher risk) for cleanings, exams, and early intervention. Mention your family history so they can monitor closely.
- **Lifestyle**: Quit smoking/vaping, manage diabetes or other conditions, eat a balanced diet (limit sugars), and stay hydrated.
- In some cases, dentists may discuss genetic testing for specific markers (e.g., IL-1), but it's not routinely needed—standard prevention is the priority.

Early detection is key, as gum disease (especially in milder forms like gingivitis) is often reversible or manageable, while advanced periodontitis can lead to tooth loss. Consult a dentist or periodontist for personalized advice based on your health history—they can assess your specific risk.

Q9 Can stress make me more likely to get gum disease?

Yes, stress can increase your likelihood of developing gum disease (gingivitis and periodontitis). It's not a direct cause on its own—plaque buildup from bacteria is the primary trigger—but chronic stress acts as a significant risk factor by affecting your body and behaviors in ways that promote gum problems.

How Stress Contributes

Stress influences gum health through biological and behavioral pathways:

- **Weakened immune response**: Chronic stress raises cortisol levels, which can suppress immune function. This makes it harder for your body to fight off the bacteria that cause inflammation and infection in the gums.
- **Increased inflammation**: Elevated cortisol and other stress hormones promote systemic inflammation, making gums more reactive to even small amounts of plaque. This can lead to swollen, bleeding gums (gingivitis) that may progress to periodontitis if untreated.
- **Behavioral changes**: Stress often leads to neglecting oral hygiene (skipping brushing/flossing), poorer diet, smoking, or teeth grinding (bruxism), all of which worsen plaque accumulation and gum irritation.
- **Other effects**: It can reduce saliva flow (dry mouth increases bacterial growth) and impair healing after dental issues or treatments. Studies also link higher stress to worse outcomes in periodontal therapy.

Research, including systematic reviews and literature analyses, supports a positive association between psychological stress and the occurrence, severity, and progression of periodontal disease. For example, stress is linked to conditions like necrotizing ulcerative gingivitis ("trench mouth") and higher rates of gum recession or tooth loss in stressed individuals.

The connection is well-established in scientific literature, though individual risk varies based on genetics, overall health, oral hygiene habits, and other factors like smoking or diabetes.

What You Can Do

- **Manage stress**: Techniques like exercise, meditation, adequate sleep, or therapy can help lower cortisol and support better habits.
- **Maintain excellent oral hygiene**: Brush twice daily, floss, and use an antimicrobial mouthwash. Regular dental cleanings are crucial.
- **See a dentist**: If you notice bleeding gums, swelling, bad breath, or recession, get checked promptly—early intervention prevents progression.
- **Lifestyle support**: Eat a balanced diet, avoid tobacco, and stay hydrated.

If stress feels overwhelming, consider talking to a healthcare professional. Taking care of both your mental and oral health can break this cycle effectively.

Q10 Do gum diseases only affect the mouth and teeth?

No, gum diseases do not only affect the mouth and teeth. While they primarily involve the gums (gingiva), periodontal ligament, and supporting bone around the teeth, substantial evidence shows they can have broader systemic impacts on overall health through chronic inflammation and bacterial spread.

How Gum Disease Extends Beyond the Mouth

Gum diseases range from **gingivitis** (milder, reversible inflammation of the gums) to **periodontitis** (more severe, involving bone and tissue destruction). In periodontitis, pockets form between teeth and gums, allowing bacteria to thrive. These bacteria and the body's inflammatory response can enter the bloodstream (bacteremia), leading to widespread effects.

Key mechanisms include:

- **Chronic inflammation**: Persistent gum inflammation raises systemic inflammatory markers, contributing to other diseases.
- **Bacterial translocation**: Oral pathogens travel via blood to distant sites like the heart, brain, lungs, or placenta.

Associated Systemic Health Risks

Research links untreated gum disease (especially periodontitis) to increased risks of:

- **Cardiovascular disease** — Higher risk of heart attack, stroke, atherosclerosis (arterial plaque), and other heart issues. Inflammation and bacteria may damage blood vessels or contribute to clots. Studies show people with periodontal disease may have a ~30% higher chance of heart attack and up to 2.5x stroke risk.
- **Diabetes** — Bidirectional link: Gum disease can worsen blood sugar control, and diabetes increases gum disease severity.
- **Adverse pregnancy outcomes** — Linked to preterm birth, low birth weight, and complications affecting the placenta.
- **Respiratory issues** — Oral bacteria can be aspirated into the lungs, raising risks of pneumonia, COPD, or worsening existing conditions.
- **Other associations** — Rheumatoid arthritis, Alzheimer's/dementia, chronic kidney disease, osteoporosis, certain cancers (e.g., pancreatic, kidney), and more. These are often correlational, with shared inflammation as a common factor.

Note that while strong associations exist, direct causation isn't always proven for every condition—gum disease often acts as a risk factor or exacerbator alongside others like smoking, poor diet, or genetics. Treating gum disease can sometimes improve systemic markers (e.g., better glycemic control in diabetics).

Prevention and Takeaways

Good oral hygiene (brushing, flossing, regular dental visits), professional cleanings, and addressing risk factors can prevent or manage gum disease and potentially reduce related systemic risks. If you have symptoms like bleeding gums, bad breath, or loose teeth—or conditions like diabetes or heart issues—consult a dentist and physician for coordinated care.

In short, oral health is closely tied to whole-body health. What starts in the mouth doesn't always stay there.

Q11 Will a hard-bristled toothbrush clean my teeth better?

No, a hard-bristled toothbrush will not clean your teeth better overall—and it can actually cause more harm than good.

Why Soft Bristles Are Recommended

Dental professionals, including the **American Dental Association (ADA)**, recommend **soft-bristled toothbrushes** for most people. They effectively remove plaque and debris while being gentle on your teeth and gums.

- **Effective cleaning**: Soft bristles are flexible. They bend to reach between teeth, along the gumline, and into crevices better than stiff ones. Proper technique (gentle pressure, 2 minutes twice a day) matters far more than bristle hardness.
- **Medium or hard bristles** may feel like they're scrubbing more aggressively, but studies and clinical consensus show they don't provide meaningfully better plaque removal for daily use. Any slight advantage is outweighed by risks.

Risks of Hard Bristles

Hard (or extra-firm) bristles can:

- **Wear down tooth enamel** over time, leading to sensitivity, increased cavity risk, and discoloration.
- **Damage gums**, causing recession, irritation, bleeding, or abrasion. Gum tissue doesn't grow back easily, exposing roots and worsening sensitivity.
- Increase the chance of long-term issues like gum disease or the need for more dental interventions.

These effects are especially problematic if you brush vigorously or have sensitive teeth/gums already.

Practical Advice

- **Choose soft bristles** (ADA-approved if possible) with a small head and rounded ends.
- Replace your toothbrush every 3 months or sooner if bristles fray.
- Focus on technique: Use gentle circular or back-and-forth motions at a 45-degree angle to the gums. Let the bristles do the work—don't scrub hard.
- Consider an electric toothbrush with pressure sensors if you tend to brush too aggressively.
- If you have specific needs (e.g., heavy staining or orthodontic appliances), ask your dentist—they might suggest alternatives for occasional use, but not daily hard brushes.

In short, **soft is safer and just as (or more) effective** for long-term oral health. If you're unsure about your brushing habits or noticing sensitivity/recession, consult a dentist for personalized advice.

Q12 Should I stop brushing my teeth for a few days if my gums are bleeding?

No, you should not stop brushing your teeth. In fact, doing so would likely make the problem worse.

Bleeding gums when brushing is most often a sign of **gingivitis** (early gum disease) caused by plaque buildup along the gumline, which irritates and inflames the gums. Other common causes include brushing too hard, using a hard-bristled toothbrush, flossing incorrectly (especially if it's new), medications (like blood thinners), vitamin deficiencies, hormonal changes, or stress.

Why stopping brushing is a bad idea

- Plaque and bacteria will continue to accumulate, increasing inflammation, bleeding, and the risk of progressing to more serious **periodontitis** (which can lead to gum recession, bone loss, and tooth loss).
- Consistent, gentle cleaning is what helps resolve the issue by removing the irritants. Dentists emphasize that you need to brush and floss to make the bleeding stop.

What you should do instead

- **Continue brushing twice a day** (and flossing once daily) with a **soft-bristled toothbrush** using gentle circular motions. Avoid aggressive scrubbing.
- Consider an electric toothbrush with a pressure sensor if you tend to brush hard.
- Rinse with warm saltwater (½ teaspoon salt in 8 oz warm water) to soothe gums.
- Use an antimicrobial mouthwash if recommended.
- Replace your toothbrush every 3–4 months (or sooner if bristles fray).

When to see a dentist

- If bleeding persists beyond 1–2 weeks despite improved hygiene.
- If you have pain, swelling, persistent bad breath, or loose teeth.
- For a professional cleaning (scaling) to remove tartar that you can't reach at home.

Bleeding gums are common and often reversible with better oral care, but ignoring them or stopping hygiene isn't the solution. See your dentist for personalized advice, especially if it's recurring or accompanied by other symptoms. This isn't medical advice—professional evaluation is best for ongoing issues.

Q13 Do I need to brush my teeth after every meal to keep my gums healthy?

No, you don't need to brush your teeth after every meal to keep your gums healthy. The standard recommendation from the American Dental Association (ADA) and other experts is to brush **twice a day** (typically morning and before bed) for two minutes each time with fluoride toothpaste, combined with daily flossing or interdental cleaning.

Why Twice a Day Is Enough

- Plaque (the sticky bacterial film) builds up over time and can irritate gums, leading to **gingivitis** (early gum disease with redness, swelling, or bleeding). Regular twice-daily brushing and flossing effectively removes it and prevents progression to more serious periodontitis.
- More frequent brushing isn't proven to provide major additional benefits for most people and can introduce risks, especially if done immediately after meals.

Timing Matters: Avoid Brushing Right After Eating

Many sources (including Mayo Clinic) advise **waiting 30–60 minutes** after eating, particularly acidic foods/drinks (citrus, soda, tomatoes, etc.), before brushing. Acids temporarily soften enamel, and brushing too soon can wear it away.

Instead, after meals:

- Rinse with water to help neutralize acids and wash away loose particles.
- Chew sugar-free gum to stimulate saliva (which naturally cleanses the mouth).
- Floss if food is stuck between teeth.

Full Routine for Healthy Gums

For optimal gum health and to prevent or reverse early gingivitis:

- **Brush twice daily** (soft-bristled toothbrush, gentle 45-degree angle to the gums, short strokes).
- **Floss or use interdental cleaners once daily** — this is crucial for areas brushing misses.
- Consider an antimicrobial mouthwash.
- Eat a balanced diet, limit sugary/acidic snacks, and avoid tobacco.
- Get professional dental cleanings regularly (every 6–12 months).

Brushing after every meal isn't necessary or always advisable, and it can be impractical. Focus on consistency with the twice-daily routine, proper technique, and flossing — that's what keeps gums healthy long-term. If you have signs of gum issues (persistent bleeding, bad breath, recession), see a dentist for personalized advice.

Q14 Even if I brush my teeth regularly, do I still need to get my gums checked?

Yes, you should still get your gums checked regularly by a dentist or dental hygienist, even if you brush your teeth consistently. Brushing is essential but doesn't fully protect against gum issues on its own.

Why Brushing Alone Isn't Enough

- **Plaque and tartar buildup**: Brushing removes a lot of plaque (the sticky bacterial film on teeth), but it often misses areas between teeth and below the gumline. Over time, leftover plaque hardens into tartar (calculus), which a toothbrush or floss can't remove. This tartar irritates gums and leads to inflammation.
- **Gum disease risk**: Early gum disease (gingivitis) can develop from this buildup, causing swollen, bleeding, or receding gums. If untreated, it can progress to periodontitis, which damages the tissues and bone supporting teeth and can lead to tooth loss. Many people don't notice early signs.
- **Hidden problems**: Dentists use specialized tools and exams (sometimes with X-rays) to detect tartar, pockets between teeth and gums, early decay, oral cancer, or other issues you can't see or feel at home.

Recommended Routine

- **Daily habits**: Brush twice a day for two minutes with fluoride toothpaste, floss (or use interdental cleaners) once a day, and consider an antimicrobial mouthwash. These greatly reduce risk but don't replace professional care.

- **Professional checkups**: Most people benefit from dental visits every 6 months for exams and cleanings (scaling to remove tartar). Some may need more frequent visits (e.g., every 3–4 months) if they have risk factors like diabetes, smoking, a history of gum disease, dry mouth, or certain medications.
- **Personalized advice**: Your dentist can assess your specific risk and adjust the schedule.

Regular professional cleanings and checkups are one of the best ways to prevent gum disease and catch issues early, saving you from more extensive (and expensive) treatments later. If your gums bleed when brushing/flossing, feel tender, or you've noticed recession or bad breath, schedule an appointment sooner. This is general guidance—consult a dentist for advice tailored to your health.

Q15 Is brushing my teeth once a day enough to keep my mouth healthy?

No, brushing your teeth once a day is generally not enough for optimal oral health. The overwhelming consensus from dental authorities is to brush **twice daily**.

Why Twice a Day?

- **Plaque and bacteria buildup**: Every time you eat or drink (especially sugary or starchy items), bacteria in your mouth produce acids and form plaque. Brushing once leaves this buildup to accumulate over ~24 hours, increasing risks of cavities, gum disease (gingivitis/periodontitis), bad breath, and tooth decay.
- **Nighttime protection**: Brushing before bed is particularly important because saliva production drops while you sleep, giving bacteria more time to thrive. Brushing in the morning removes overnight buildup.

Official recommendations:

- **American Dental Association (ADA)**: Brush twice a day for at least two minutes each time with a soft-bristled toothbrush and fluoride toothpaste.
- **Mayo Clinic** and others echo this.

One older review suggested that meticulous brushing once per day could prevent caries and periodontal issues in some cases, but this is not the standard advice—twice daily provides better plaque control and is what most experts promote.

Better Results with a Full Routine

For the healthiest mouth:

- **Brush twice daily** (morning and night) for 2 minutes.
- **Floss once a day** (or use interdental cleaners) — brushing alone misses spaces between teeth.
- Use fluoride toothpaste.
- Consider an electric toothbrush for better plaque removal.
- Rinse with water after meals if you can't brush; limit sugary/acidic drinks and snacks.
- Visit a dentist regularly (e.g., every 6 months) for cleanings and checkups.

Brushing once a day is better than nothing and may work okay short-term for low-risk people with excellent technique, diet, and genetics—but it's not ideal long-term and raises your risk of problems. If you have specific concerns (e.g., dry mouth, braces, or gum issues), talk to your dentist for personalized advice. Consistent twice-daily brushing is a simple, effective habit for keeping your teeth and gums healthy.

Q16 Can flossing cause gum recession?

Yes, flossing *can* contribute to gum recession, but only if done incorrectly or too aggressively. Proper flossing is generally beneficial and helps *prevent* recession by removing plaque and food particles that cause gum disease.

Why Improper Flossing Can Cause Recession

Gum recession occurs when the gum tissue pulls away from the teeth, exposing the roots. Aggressive flossing is one of several contributing factors (others include aggressive brushing, genetics, smoking, periodontal disease, and poor overall hygiene).

Common mistakes that can irritate or damage gums include:

- **Snapping or yanking the floss** forcefully between teeth, which puts direct pressure on the gums.
- **Using too much force** or rubbing too hard against the gumline.
- **Flossing too frequently** (e.g., multiple times a day when not needed), leading to chronic irritation.
- Not using the proper "C-shape" technique around each tooth.

These actions can cause inflammation, bleeding, cuts, or trauma that, over time, leads to the gums receding.

The Benefits of Proper Flossing

- **Prevents gum disease**: Plaque buildup between teeth (where brushes can't reach) leads to gingivitis and periodontitis, major causes of recession. Daily flossing removes this effectively.
- Dentists and organizations like the American Dental Association (ADA) strongly recommend flossing once a day as part of good oral hygiene, alongside brushing and regular dental visits.
- Not flossing often worsens problems: It allows plaque to accumulate, inflaming gums and potentially causing more recession long-term.

How to Floss Correctly (to Avoid Harm)

Follow this technique recommended by dental professionals:

1. Use about 18 inches of floss; wrap most around your middle fingers.
2. Hold it taut with thumbs and index fingers.
3. Gently guide it between teeth with a rubbing motion—**don't snap it**.
4. Curve it into a "C" shape against one tooth and slide it gently under the gumline.

5. Rub up and down along the side of the tooth (not just sawing back and forth).
6. Repeat for each tooth, using a clean section of floss.

Tips: Be gentle—your gums are delicate. If your gums bleed at first, it's often normal (sign of inflammation) and should improve with consistent proper technique. Consider alternatives like water flossers if traditional floss is hard on your gums.

Other Advice

- Pair flossing with soft-bristled brushing (twice daily) and professional cleanings.
- If you notice ongoing recession, bleeding, sensitivity, or pain, see a dentist or periodontist. They can check for underlying issues and may recommend treatments like grafts for advanced cases.

In summary, **flossing itself is not the villain**—aggressive or sloppy technique is. Done right, it's one of the best things you can do for your gum health. If you're concerned about your habits, a quick demo from your dentist or hygienist can make a big difference.

Q17 Do electric toothbrushes damage the gums more than regular toothbrushes?

No, electric toothbrushes do not damage the gums more than regular (manual) toothbrushes. Evidence from systematic reviews, long-term studies, and dental organizations shows that electric brushes are generally at least as safe—and often superior—for gum health, primarily because they remove more plaque and reduce gingivitis more effectively.

Key Evidence from Studies

- **Cochrane Review (2014, with follow-ups)**: Powered toothbrushes reduce plaque by ~11-21% and gingivitis by ~6-11% more than manual brushing in short- and long-term use. Adverse effects like gingival abrasion were similar or slightly lower with powered brushes in the trials reviewed.
- **11-year cohort study**: Electric toothbrush users had **22% less gum recession** and **18% less tooth decay** progression compared to manual users.
- **3-year randomized study**: In people with pre-existing gingival recession, neither type worsened recession when used properly; some evidence showed slight benefits or stability with powered brushes.
- Other research (including meta-analyses) supports oscillating-rotating electric brushes for better plaque/gingivitis control, with features like pressure sensors helping prevent over-brushing.

Damage to gums (abrasion or recession) is mainly caused by **improper technique**—brushing too hard, using hard bristles, or scrubbing aggressively—**not the brush type itself**. Both manual and electric brushes can cause issues if misused, but many electric models include built-in safeguards like pressure sensors, timers, and gentle modes that reduce this risk.

Recommendations from Dental Authorities

- The **American Dental Association (ADA)** endorses both types when they carry the ADA Seal. They recommend **soft bristles** and gentle pressure for either, noting that powered brushes can be easier for thorough cleaning, especially for people with dexterity issues.
- Electric brushes are often highlighted as helpful for gum health because superior plaque removal reduces inflammation and supports healthier gums over time.

Practical Tips to Protect Your Gums

- Use **soft bristles** (avoid medium/hard).
- Brush gently at a 45-degree angle to the gumline for 2 minutes twice daily.
- Let the electric brush do the work—don't scrub or apply heavy pressure (many models alert you if you do).
- Replace brush heads every 3 months.
- Combine with flossing or interdental cleaning.
- If you have receding gums or sensitivity, consult a dentist; they may recommend specific modes or models.

In summary, when used correctly, electric toothbrushes are typically **better or equal** for gum health due to improved cleaning and user-friendly features. The choice often comes down to personal preference and consistency. If you have specific concerns (e.g., existing gum issues), see a dental professional for personalized advice.

Q18 Would using mouthwash alone be enough without brushing my teeth?

No, using mouthwash alone is not enough to maintain good oral health. Brushing your teeth is the foundation of effective dental hygiene, and mouthwash is best used as a supplement, not a replacement.

Why brushing is essential

- **Mechanical cleaning**: Brushing physically removes plaque (a sticky bacterial film), food particles, and debris from tooth surfaces, especially in hard-to-reach areas. Mouthwash mostly swishes around and doesn't scrub effectively.
- **Gum health and prevention**: Regular brushing helps prevent gum disease (gingivitis/periodontitis), cavities, and tartar buildup. Mouthwash can't do this on its own.
- **Fluoride delivery**: Most toothpastes contain fluoride, which strengthens enamel and protects against decay. Many mouthwashes have fluoride too, but the brushing action ensures better contact and longer retention on teeth.

What mouthwash does well

- Kills bacteria and reduces plaque-causing microbes (especially antiseptic ones like those with chlorhexidine or essential oils).
- Freshens breath.

- Can help with minor gum inflammation or sensitivity (depending on the type: fluoride, whitening, antibacterial, etc.).
- Reaches some areas brushing might miss.

However, even the best mouthwash leaves behind much of the plaque and debris that brushing targets.

Recommended routine (per dental guidelines)

1. **Brush twice daily** for 2 minutes with fluoride toothpaste.
2. **Floss or use interdental cleaners** once daily.
3. **Use mouthwash** as an optional rinse afterward (or at a separate time) — don't rinse with water right after brushing if using fluoride products, so the fluoride stays on your teeth longer.
4. Regular dental check-ups and cleanings.

Studies and organizations like the American Dental Association (ADA) consistently emphasize that **mechanical plaque removal via brushing and flossing is irreplaceable** for long-term oral health. Relying solely on mouthwash increases risks of cavities, gum disease, bad breath (from trapped debris), and staining.

Exceptions / special cases

- If you physically cannot brush (e.g., post-surgery or certain disabilities), your dentist might recommend specific antimicrobial rinses as a temporary measure — but even then, other tools like sponges or water flossers are often suggested.
- Some therapeutic mouthwashes (prescription-strength) are used for short-term treatment of infections, but still alongside brushing when possible.

Bottom line: Mouthwash is helpful, but skipping brushing is like washing your car with just air freshener — it might smell better temporarily, but the dirt stays. For best results, combine both (and flossing). If you have specific concerns (sensitivity, braces, etc.), consult a dentist for personalized advice.

Q19 After I quit smoking, my gums started bleeding. Is that normal?

Yes, bleeding gums after quitting smoking is quite common and often a normal (even positive) sign of your body starting to heal.

Why This Happens

Smoking constricts blood vessels and reduces blood flow to your gums. This can "mask" underlying issues like inflammation or early gum disease (gingivitis/periodontitis) by limiting redness, swelling, and bleeding. When you quit:

- Blood flow and circulation improve quickly (often within days to weeks).
- More oxygen and nutrients reach the gum tissues.

- Any hidden inflammation becomes noticeable, leading to temporary bleeding, tenderness, or swelling.

This "unmasking" effect is widely reported by dental professionals and people who quit. It's your gums "waking up" and responding more normally to plaque or minor irritation.

How Long Does It Last?

- It often peaks in the first 1–4 weeks and tends to improve as healing progresses.
- Many see noticeable reduction in bleeding within a few weeks, with broader gum health improvements over 1–3 months (and even more over longer periods).

When to See a Dentist

While temporary bleeding is expected, don't ignore it entirely—quitting gives your gums a much better chance to recover, but underlying gum disease may still need professional care (like a cleaning). Contact a dentist if you notice:

- Heavy or persistent bleeding beyond a few weeks.
- Severe pain, swelling, pus, bad taste, or loose teeth.
- Symptoms that worsen instead of improving.

Good oral hygiene helps a lot: Brush gently twice a day (soft toothbrush), floss daily, and consider an antimicrobial mouthwash. Your dentist can give personalized advice.

Congratulations on quitting smoking—that's one of the best things you can do for your overall and oral health! Gums, breath, taste, and more all tend to improve over time. If you're concerned, a dental check-up is the best next step for peace of mind.

Q20 Can natural products effectively keep my gums healthy?

Yes, some natural products can help support gum health effectively as adjuncts to standard oral hygiene, especially for preventing or managing mild gingivitis (gum inflammation), but they are not a substitute for brushing, flossing, professional dental cleanings, or treating advanced periodontal disease.

Core Principles for Gum Health

Healthy gums rely primarily on mechanical plaque removal (brushing twice daily, flossing daily) and regular dental visits. Plaque buildup causes gingivitis, which can progress to periodontitis (deeper infection and bone loss) if untreated. Natural options may reduce bacteria, inflammation, or plaque modestly but rarely reverse significant damage on their own.

Promising Natural Options with Some Evidence

Several natural approaches show benefits in studies, often comparable to certain conventional rinses for mild cases:

- **Aloe Vera**: Gel or mouthwash forms can reduce plaque and gingivitis symptoms similarly to chlorhexidine (a standard antibacterial rinse) in some trials, with anti-inflammatory and healing effects. Useful for soothing irritated gums or post-surgery recovery.

- **Green Tea**: Rich in catechins (antioxidants) with anti-inflammatory properties. Regular consumption links to lower gum bleeding and better gum health in observational studies.
- **Oil Pulling (e.g., Coconut Oil)**: Swishing oil for 10–20 minutes daily can reduce plaque and gingivitis signs in some small studies, likely due to antimicrobial lauric acid and mechanical action. Effects are often modest and best as an add-on; it doesn't reliably cure or reverse advanced disease.
- **Saltwater Rinses**: Simple, effective for short-term relief—reduces bacteria and inflammation similarly to some medicated rinses in studies.
- **Herbal Extracts and Essential Oils**:
    - Lemongrass oil mouthwash may outperform or match chlorhexidine for plaque reduction.
    - Tea tree oil, turmeric (curcumin for anti-inflammatory effects), clove oil, and others show antimicrobial or soothing benefits in trials.
    - Pomegranate, miswak, and certain herbal blends also have supportive evidence for gingivitis.
- **Supplements**: Vitamin C (for connective tissue), Vitamin D, lycopene, or proanthocyanidins may help reduce inflammation or support healing when addressing deficiencies, per some studies.

Limitations: Many studies are small, short-term, or focus on gingivitis rather than severe periodontitis. Results vary by individual, product quality, and consistency. Natural products don't remove tartar (hardened plaque) and may not penetrate deep pockets.

Recommendations and Cautions

- **Use as complements**: Combine with proven habits—fluoride toothpaste, interdental cleaning, and dentist visits every 6 months (or more if needed).
- **Safety**: Dilute essential oils properly (they can irritate or be toxic if swallowed undiluted). Test for allergies. Avoid if you have open wounds or specific conditions.
- **When to see a dentist**: For bleeding, swelling, pain, recession, bad breath, or loose teeth—these signal issues beyond what home remedies can handle.
- Consult a dental professional or doctor before relying heavily on naturals, especially if pregnant, on medications, or with existing health conditions.

In summary, natural products like aloe vera, green tea, oil pulling, and certain herbs can meaningfully contribute to healthier gums for many people when used consistently alongside good hygiene. They're not miracle cures but valuable supportive tools backed by varying levels of evidence. Prioritize professional advice for personalized care.

Q21 Can getting a dental scaling damage my teeth?

No, getting a dental scaling (also called professional cleaning or scaling and root planing) does not damage your teeth when performed by a qualified dentist or hygienist.

Why Scaling Is Safe and Beneficial

- **It targets tartar (calculus), not enamel**: Professional instruments (manual scalers or ultrasonic tools) are designed to remove hardened plaque and bacteria that stick to the tooth surface without significantly affecting the much harder enamel layer. Enamel is the hardest substance in the human body, and scaling tools are calibrated accordingly.
- **It prevents bigger problems**: Leaving tartar in place promotes gum disease (gingivitis progressing to periodontitis), which can cause gum recession, bone loss, loose teeth, and eventual tooth loss. Scaling helps maintain gum health and protects your teeth long-term.
- **Common myths debunked**: People sometimes worry that scaling "scrapes away enamel" or creates gaps/loosens teeth. In reality, any perceived changes (like teeth feeling different or spaces appearing) usually result from removing buildup that was masking natural tooth positions or from existing gum issues—not from damage caused by the procedure.

Potential Temporary Side Effects (Not Damage)

Most people experience only mild, short-term effects:

- **Tooth sensitivity** to hot/cold (often lasts a few days to a week) — this happens because cleaning removes the insulating layer of tartar, exposing more sensitive areas temporarily. Dentists often recommend desensitizing toothpaste.
- **Gum tenderness, swelling, or minor bleeding** — especially if you had significant buildup or early gum disease.
- These resolve quickly with proper aftercare.

Rare Risks

- **Aggressive or improper technique**: In rare cases, overly forceful scaling could cause minor enamel roughness, gum recession, or irritation—particularly on teeth with pre-existing cracks, early decay, or restorations. This is why choosing an experienced professional matters.
- **Exposed roots**: If you already have gum recession, the softer root surfaces (cementum) can be more sensitive or slightly affected, but this isn't typical "tooth damage."
- Studies (e.g., from PubMed) note ultrasonic scaling may increase surface roughness on compromised teeth, but overall clinical benefits outweigh risks for most patients.

DIY scaling or at-home tools are **not recommended** and can actually scratch enamel or injure gums.

Recommendations

- Scaling is a standard, evidence-based procedure endorsed by organizations like the American Dental Association (ADA). Benefits generally outweigh minor risks.
- Discuss any concerns (e.g., sensitivity history, existing restorations) with your dentist beforehand.
- Maintain good oral hygiene between visits to reduce how often deep cleanings are needed.

If you have specific symptoms or conditions (like severe gum disease), your dentist can tailor the approach. Overall, routine scaling supports healthier teeth and gums rather than harming them. Consult a dental professional for personalized advice.

Q22 Can dental scaling cause gaps between my teeth?

No, dental scaling (also called teeth cleaning or scaling and root planing) does not cause gaps between your teeth.

It can make existing spaces more noticeable, but the procedure itself doesn't create new gaps or push teeth apart. Here's why this misconception is common and what's actually happening:

Why gaps seem to appear after scaling

- **Tartar/plaque removal reveals hidden spaces**: Buildup of hardened tartar (calculus) can fill or mask small natural spaces between teeth. When it's removed during cleaning, those areas become visible, often surprising people.
- **Gum healing after inflammation**: Inflamed or swollen gums (from gingivitis or early periodontitis) can puff up and hide spaces. Deep cleaning reduces inflammation and bacteria, allowing gums to shrink back to a healthier state. This can expose or emphasize pre-existing gaps as the gums "tighten" around the teeth.
- **Underlying issues become apparent**: In cases of gum disease, there may already be some bone loss or recession. Cleaning doesn't cause this—it treats it and makes the true condition clearer. Teeth might feel temporarily looser for similar reasons (tartar no longer "gluing" them), but gums usually reattach with healing.

These changes are generally normal and part of the healing process. Many "gaps" improve or stabilize as gums recover over weeks.

When to be concerned

- If gaps are large, persistent, or accompanied by pain, increased sensitivity, bleeding, or loose teeth, consult your dentist. This could indicate more advanced periodontal disease needing further treatment.
- Scaling is safe and beneficial when done properly. It helps prevent worse issues like tooth loss from untreated gum disease.

Prevention and next steps

- Maintain good oral hygiene (brushing, flossing, regular cleanings) to minimize buildup and gum problems.
- Follow your dentist's post-cleaning advice, like using any prescribed rinses or avoiding certain foods temporarily.
- For cosmetic gaps (diastema), options like bonding, veneers, or orthodontics exist, but these are unrelated to the cleaning itself.

Always discuss concerns with your dentist or hygienist—they can explain your specific situation based on X-rays or exams. Professional cleanings are one of the best things you can do for long-term dental health.

Q23 Do all gum diseases require the same treatment?

No, not all gum diseases require the same treatment. Gum (periodontal) diseases vary in severity, and treatment is tailored to the specific type, stage, and individual factors like overall health, risk factors (smoking, diabetes, genetics), and how far the disease has progressed.

Main Types of Gum Disease

- **Gingivitis**: The mildest and most common form. It's inflammation of the gums caused primarily by plaque buildup. Symptoms include red, swollen, or bleeding gums. It is **reversible** with good care.
- **Periodontitis**: Develops if gingivitis is untreated. It involves deeper infection that damages the soft tissue and bone supporting the teeth. It can lead to gum recession, pockets, loose teeth, and tooth loss. This is more serious and **not fully reversible**, though it can be managed.

There are also aggressive forms, necrotizing periodontal diseases (more common in severely immunocompromised people), and other less common variants.

Why Treatments Differ

Treatments scale with severity:

For Gingivitis (early/mild):

- Improved daily oral hygiene: Brushing twice daily, flossing, using antimicrobial mouthwash.
- Professional dental cleaning (prophylaxis) to remove plaque and tartar.
- Lifestyle changes (better diet, quitting smoking).
- Often no need for advanced procedures — it can resolve in days to weeks with consistent care.

For Periodontitis (moderate to advanced):

- **Scaling and root planing** (deep cleaning) to remove tartar below the gumline.
- Antibiotics (topical gels, oral, or locally delivered) to control infection.
- Surgical options in advanced cases: flap surgery, bone grafts, tissue regeneration, or pocket reduction.
- Ongoing maintenance: More frequent professional cleanings (every 3–4 months).
- Addressing underlying conditions (e.g., diabetes control).

In severe cases with significant bone loss, treatment may involve tooth extraction and options like implants or dentures.

Common Factors Across Treatments

All gum disease management emphasizes:

- Removing bacterial plaque and tartar.
- Patient compliance with home care.
- Regular dental visits for monitoring.
- Risk factor modification (smoking cessation is especially important).

Early intervention is key — gingivitis can progress to periodontitis, but catching it early simplifies treatment dramatically.

Important note: This is general information. Diagnosis and personalized treatment plans must come from a dentist or periodontist after a clinical exam, X-rays, and probing measurements. Self-diagnosis or self-treatment isn't reliable. If you have symptoms like persistent bleeding gums, bad breath, pain, or loose teeth, see a dental professional promptly.

Q24 Is gum treatment painful?

No, gum treatment (periodontal treatment) is generally not very painful, especially with modern techniques and anesthesia.

During the Procedure

Most common gum treatments, like **scaling and root planing** (deep cleaning), are performed under **local anesthesia**, so patients typically feel little to no sharp pain—only pressure, vibration, or mild scraping sensations.

- **Laser therapy** or minimally invasive options often cause even less discomfort and may require minimal or no anesthesia in some cases.
- Surgical procedures (e.g., pocket reduction or gum grafts) are more invasive and can involve more discomfort, but anesthesia and sedation keep patients comfortable during the work.

Dentists prioritize patient comfort, and many people report the experience as far less daunting than expected.

After the Procedure

Mild soreness, tenderness, sensitivity, or swelling in the gums is common for a few days (sometimes up to a week), especially after deep cleaning. This is usually manageable with over-the-counter pain relievers (like ibuprofen), soft foods, and good oral hygiene.

Pain levels vary based on:

- Severity of gum disease (inflamed gums are more sensitive).
- Individual pain tolerance.
- Specific treatment type.

Why It's Worth It

Untreated gum disease can lead to more pain, bleeding, tooth loss, and other issues over time. Early treatment is often simpler and less uncomfortable.

Talk to your dentist or periodontist — they can explain your specific case, use numbing agents, offer sedation if needed, and provide aftercare tips. Modern dentistry has made these treatments much more comfortable than in the past. If you're anxious, discuss your concerns upfront for the best experience.

Q25 Can gum surgery cause tooth loss?

Yes, but it's uncommon and usually not a direct result of the surgery itself. Gum surgery (such as gum grafts, flap surgery, or osseous surgery for periodontal disease) is generally performed to *prevent* further gum recession, bone loss, and tooth loss. However, like any surgical procedure, it carries some risks that could indirectly contribute to complications affecting tooth stability.

Why Surgery Is Usually Protective

- Gum disease (periodontitis) and recession are leading causes of tooth loss in adults. Surgery aims to:
    - Reduce infection and inflammation.
    - Cover exposed roots (e.g., via gum grafts).
    - Reshape or regenerate bone and tissue to stabilize teeth.
- Successful procedures often improve long-term tooth retention by halting disease progression.

Potential Risks and Complications

Complications from gum surgery are relatively uncommon when performed by an experienced periodontist, but they can include:

- **Graft failure or rejection**: The transplanted tissue may not integrate well, leading to continued recession or exposure. This is more likely with poor healing, smoking, or advanced pre-existing bone loss.
- **Infection, excessive bleeding, or swelling**: These can delay healing and, if severe/untreated, potentially worsen periodontal issues.
- **Increased tooth mobility temporarily**: Surgery involves manipulating gums and sometimes bone; teeth may feel looser during healing, but this usually stabilizes.
- **Rare worsening of bone loss**: In advanced cases with significant pre-existing damage, surgery might not fully restore support, and teeth could eventually loosen or be lost if the underlying disease isn't controlled.

Tooth loss is far more commonly linked to **untreated gum disease** than to the surgery. Delaying treatment increases risks of decay, mobility, and loss.

Factors That Influence Risk

- **Severity of condition**: Surgery in late-stage disease (with major bone loss) has higher failure rates and may not save all teeth.
- **Patient factors**: Smoking, poor oral hygiene, uncontrolled diabetes, or not following post-op care raise complication risks.
- **Procedure type**: Gum grafts or regenerative procedures generally have good success rates, but outcomes vary.

What to Expect and How to Minimize Risks

- Recovery typically involves 1–2 weeks of discomfort, with full healing over weeks to months. Follow instructions on diet, oral care, and medications closely.

- Success depends on choosing a qualified specialist and maintaining good long-term hygiene.
- Consult your dentist/periodontist for a personalized assessment—they can evaluate your specific situation with X-rays or probing.

In summary, gum surgery is a proven way to help *avoid* tooth loss in most cases, but it's not risk-free. Discuss concerns, alternatives (like non-surgical scaling/root planing for milder cases), and realistic expectations with your oral health provider. Early intervention generally yields the best outcomes.

Q26 Can gum treatment make my teeth feel loose?

Yes, gum treatment (such as scaling and root planing, also called deep cleaning) can sometimes make teeth feel temporarily loose, but this is usually a normal, short-term effect rather than a sign of worsening problems.

Why This Happens

Gum disease (periodontitis) causes inflammation, swelling, and tartar buildup that can artificially "support" or stabilize teeth even as the underlying bone and ligaments weaken. During treatment like **scaling and root planing**:

- Dentists remove plaque, tartar (calculus), and infected tissue from below the gumline and smooth the tooth roots.
- This eliminates the bacterial infection and reduces swelling, which can reveal the true state of the supporting structures.
- As a result, teeth may feel wigglier right after the procedure because the "pseudo-support" from tartar and inflamed tissue is gone.

This sensation is common and typically temporary. As the gums heal and reattach (a process that can take weeks to months), teeth often feel more stable.

Important Context

- **The goal of treatment is to stabilize or improve things long-term**: Gum treatments address the root cause (infection and bone loss from periodontitis), which is the leading reason for loose teeth in adults. Without treatment, loose teeth would likely worsen, potentially leading to tooth loss.
- Studies and clinical observations show that scaling and root planing often reduces tooth mobility over time (e.g., many teeth with mild-to-moderate mobility become stable within a year).
- More advanced procedures (e.g., gum grafts, bone grafting, or splinting) may be used if needed to provide additional support.

When to Be Concerned and What to Do

- **Normal recovery**: Mild looseness, sensitivity, discomfort, or minor bleeding for a few days to weeks is expected. Good oral hygiene (gentle brushing, flossing, and any prescribed rinses) helps healing.

- **See your dentist promptly** if looseness worsens, persists beyond a few weeks, or comes with severe pain, swelling, pus, or difficulty chewing. They can assess mobility, consider splinting if needed, or adjust the treatment plan.
- Always follow post-treatment instructions and attend follow-up visits.

This is general information based on common dental practices—**I'm not a dentist, and this isn't personalized medical advice**. Individual outcomes vary based on the severity of gum disease, overall health, and other factors. Consult your dentist or periodontist for an evaluation tailored to your situation. Early intervention is key to the best results.

Q27 Does tartar removal cause more tartar buildup?

No, tartar (dental calculus) removal does not cause more tartar buildup.

Tartar forms when plaque—a sticky bacterial film—hardens due to minerals in saliva. It builds up in areas that are hard to clean, creating a rough, porous surface that attracts even more plaque and bacteria, which can lead to gum disease, inflammation, and other issues. Professional removal (scaling) breaks this cycle.

Why the myth might exist

Visible or quick reformation: Plaque starts reforming almost immediately after cleaning (within hours), and it can begin mineralizing into tartar within 24–72 hours if oral hygiene isn't excellent. People with heavy prior buildup, certain saliva compositions, diets, smoking, mouth breathing, or genetics may notice it seeming to return "faster" after a cleaning—simply because the teeth are now clean and any new deposits stand out more.

Smoother teeth after polishing: Dentists often polish teeth after scaling, which creates a smoother surface that actually slows future plaque adhesion and tartar formation.

No "rebound" effect: Reliable dental sources (e.g., Cleveland Clinic, ADA) and studies on scaling/root planing show no evidence that professional removal increases the rate of tartar formation. It reduces bacterial load, helps gums heal, and prevents progression of problems. Leaving tartar on makes buildup worse over time.

What actually happens with removal

A hygienist or dentist uses specialized tools (hand scalers, ultrasonic devices) to remove tartar above and below the gumline safely.

For deeper issues (periodontitis), scaling and root planing smooths roots to discourage reattachment.

Follow-up care (brushing twice daily, flossing, possibly antimicrobial rinses) and regular cleanings (typically every 6 months, or more often for high-risk individuals) keep it under control.

Prevention tips

Brush and floss effectively daily—plaque removal is key, as it never hardens into tartar if cleared promptly.

Use tartar-control toothpaste if recommended.

Maintain regular dental visits; some people form tartar faster due to individual factors (saliva pH, diet, etc.), but professional care manages it well.

Avoid home "picking" at tartar, which can damage enamel/gums and worsen issues.

In short, getting tartar removed is one of the best things you can do for your oral health—it doesn't create a vicious cycle, it interrupts one. If you're concerned about rapid recurrence, talk to your dentist about personalized factors and hygiene improvements.

Q28 Are antibiotics enough to treat gum disease?

No, antibiotics are generally not enough on their own to treat gum disease (gingivitis or periodontitis). They can help control bacterial infection as part of a broader treatment plan, but mechanical removal of plaque and tartar, along with good oral hygiene, is essential for effective, long-term results.

Why Antibiotics Alone Aren't Sufficient

Gum disease is driven by bacterial plaque and tartar (calculus) buildup, which irritates gums, causes inflammation, and can lead to deeper pockets, bone loss, and tooth mobility in advanced cases (periodontitis).

Antibiotics reduce bacteria but cannot physically remove the hardened tartar or plaque biofilm that harbors bacteria below the gumline. Without cleaning (scaling and root planing), bacteria quickly return once antibiotics stop.

Evidence shows limited or no benefit from systemic antibiotics as monotherapy for chronic periodontitis. Guidelines generally recommend against routine use of systemic antibiotics for standard cases due to antibiotic resistance concerns and side effects.

Standard Treatment Approach

Professional mechanical cleaning: Scaling (removing tartar) and root planing (smoothing roots) form the foundation. This is often enough for mild to moderate cases (gingivitis or early periodontitis), combined with improved daily brushing/flossing.

Antibiotics as adjuncts (when needed):

Topical/local: Gels, chips, or rinses (e.g., containing minocycline or chlorhexidine) placed in gum pockets for targeted effect with fewer systemic side effects.

Systemic (oral): Amoxicillin, metronidazole, doxycycline, or combinations for more severe, aggressive, or refractory cases, acute abscesses, or when infection spreads. Timing (e.g., starting before or with cleaning) can influence microbiome recovery.

Maintenance: Ongoing hygiene, regular dental visits, and addressing risk factors (smoking, diabetes, etc.).

In advanced cases, surgery (e.g., flap surgery or regeneration) may be required.

When Antibiotics Might Be Used

Severe or aggressive periodontitis.

Periodontal abscesses.

Non-responding sites or specific high-risk bacteria.

Certain patient groups (e.g., younger patients with deeper pockets).

Always consult a dentist or periodontist for personalized advice—self-medicating with antibiotics is ineffective and risky. Early intervention with cleanings and hygiene can often reverse gingivitis without drugs. Long-term success depends on consistent oral care to prevent recurrence.

Q29 After gum disease treatment, will I ever need treatment again?

No, gum disease (particularly periodontitis) is generally not "cured" in a one-and-done way—it's a chronic condition that often requires lifelong management to prevent recurrence or progression.

Key Points on Recurrence

Gingivitis (early, reversible stage) can often be fully reversed with improved oral hygiene and professional cleanings.

Periodontitis (advanced stage involving bone and tissue loss) is manageable but not fully curable. Once bone/supporting structures are lost, they don't fully regenerate in most cases, though treatment can halt progression and stabilize the condition.

Bacteria can recolonize gum pockets relatively quickly (as soon as 2–4 months after treatment) if plaque and tartar build up again. Recurrence is common without consistent care, but it doesn't mean the initial treatment "failed"—it reflects the ongoing nature of the disease.

What Successful Long-Term Management Looks Like

After active treatment (e.g., scaling/root planing, surgery, or other therapies), most patients enter a periodontal maintenance program:

Professional cleanings every 3–4 months (more frequent than the standard 6 months) to remove plaque below the gumline and monitor pockets/bone levels.

Excellent daily home care: brushing twice daily (soft/electric toothbrush), flossing or using interdental cleaners daily, and possibly antimicrobial rinses or other tools recommended by your dentist.

Regular check-ups with your dentist or periodontist to catch early signs of recurrence (e.g., bleeding, pocket deepening, bone loss on X-rays).

Addressing risk factors: quitting smoking, managing diabetes or other systemic conditions, and maintaining a healthy diet.

Studies and dental guidelines (e.g., from the ADA and periodontal associations) show that consistent maintenance significantly reduces the risk of tooth loss and disease progression.

Factors That Increase Recurrence Risk

Poor oral hygiene.

Skipping maintenance visits.

Smoking or tobacco use.

Uncontrolled health conditions (e.g., diabetes).

Genetic predisposition or certain medications.

If you notice signs like bleeding gums, bad breath, swelling, or loose teeth, see your dentist promptly—early intervention can prevent major issues.

This is general information based on dental health resources. Individual outcomes vary based on the severity of your case, treatment type, and personal factors. Consult your dentist or periodontist for personalized advice, as they know your specific history and can tailor a maintenance plan. With good compliance, many people maintain stable gum health for years or decades after treatment.

Q30 Is surgery always necessary for gum treatment?

No, surgery is not always necessary for gum treatment. Most cases of gum disease (periodontal disease) can be managed effectively with non-surgical approaches, especially when caught early. Surgery is typically reserved for more advanced or severe cases where non-surgical methods aren't enough.

Stages and Treatment Options

Gum disease progresses in stages, and treatment is tailored to the severity:

Gingivitis (early stage): Gums are inflamed, red, or bleed easily, but no major damage has occurred. This is often reversible with improved oral hygiene (brushing, flossing, regular cleanings) and professional cleanings. No surgery needed.

Mild to moderate periodontitis: Deeper pockets form around teeth due to plaque and tartar buildup. The first-line treatment is usually scaling and root planing (a deep cleaning to remove bacteria and smooth tooth roots), often combined with antibiotics or antimicrobial rinses. Many patients respond well without surgery. Laser therapy or other non-surgical methods may also help.

Advanced periodontitis: Significant bone loss, deep pockets, gum recession, or loose teeth may require surgical intervention to clean areas that can't be reached otherwise, reduce pocket depth, regenerate bone/tissue, or graft gums.

Common Non-Surgical Treatments

These are less invasive, have shorter recovery times, and are often sufficient:

Professional deep cleanings (scaling and root planing).

Improved daily oral hygiene and maintenance visits.

Antibiotics (topical or oral) to control infection.

Laser-assisted therapies in some cases.

When Surgery Might Be Needed

Surgery (e.g., flap surgery, bone grafts, gum grafts) is considered when:

Non-surgical treatments don't reduce pocket depths or control infection.

There's significant gum recession exposing roots, bone loss, or risk of tooth loss.

The goal is to regenerate lost tissue or stabilize advanced disease.

Even then, it's not "always" required—outcomes depend on individual factors like overall health, smoking status, genetics, and how well the patient maintains oral hygiene afterward.

Important Advice

See a professional: A dentist or periodontist will assess your specific situation with exams, X-rays, and pocket measurements. Early intervention often prevents the need for surgery.

Outcomes vary: Good oral care and follow-up maintenance are key to success in both non-surgical and surgical cases.

This is general information, not personalized medical advice. Consult a qualified dental professional for diagnosis and treatment recommendations.

Catching gum issues early makes a big difference—regular check-ups are your best tool.
