# Supplementary material for: Comparative Evaluation of Large Language Models in Responding to Common Misconceptions Regarding Periodontal Disease and Oral Hygiene
Source: Diagnostics (Basel). 2026 Sep 28;16(19):3158. doi: 10.3390/diagnostics16193158 (PMC13664004; doi:10.3390/diagnostics16193158)
Supplement: Supplementary file 1 [file diagnostics-16-03158-s001.zip › Supplementary File S5 LLMs response stylistic elements removed.zip!/1. LLMs response/Claude 4.6 Sonnet.pdf]

Q1 Do gum diseases only affect elderly people?

No, gum disease affects people of all ages including children, teenagers, and young adults.

Who gets gum disease?

Children and teenagers can develop gingivitis (the milder, early form), especially during puberty when hormonal changes make gums more sensitive to plaque buildup. It's actually quite common in adolescents.

Young and middle-aged adults are frequently diagnosed with periodontitis (the more serious form), often because early warning signs like bleeding gums get ignored.

Older adults do have higher rates of severe gum disease, which is where the misconception comes from but that's because it's cumulative. Years of mild, untreated disease progresses into something more serious, not because aging itself causes it.

The real risk factors (age is just one of them).

Poor oral hygiene the primary driver at any age.

Smoking one of the strongest risk factors overall.

Diabetes significantly raises susceptibility.

Genetics some people are more prone regardless of habits.

Hormonal changes pregnancy, puberty, menopause all increase gum sensitivity.

Medications some drugs cause gum overgrowth or dry mouth.

Stress weakens immune response, making it harder to fight infection.

The key takeaway.

Gum disease is largely a preventable, progressive condition. Starting good habits early brushing twice daily, flossing, regular dental cleanings dramatically reduces lifetime risk, regardless of age. The earlier it's caught, the easier it is to treat.

Q2 Is it normal if my gums bleed when I brush my teeth?

Occasional, mild bleeding isn't unusual if you've just started flossing or switched to a harder brush but regular bleeding when you brush is not something to ignore or consider "normal."

What's likely going on

The most common cause is gingivitis early-stage gum inflammation from plaque buildup along the gumline. The good news: gingivitis is reversible with better oral hygiene and a professional cleaning.

Other possible causes include:

Brushing too hard or using a medium firm-bristled brush

Flossing infrequently gums toughen up with consistent flossing; bleeding usually stops within a week or two.

Hormonal changes (pregnancy, menstrual cycle).

Certain medications blood thinners, aspirin, some antihistamines.

Vitamin C or K deficiency.

Periodontitis more advanced gum disease, if gingivitis goes untreated.

What you can do

Switch to a soft-bristled toothbrush and use gentle, circular motions not back-and-forth scrubbing.

Floss daily, consistently bleeding from flossing typically resolves within 1 2 weeks.

Rinse with an antiseptic mouthwash (chlorhexidine-based ones are most effective short-term).

See a dentist if bleeding persists beyond 2 weeks of good hygiene, is heavy, or is accompanied by swollen/receding gums or bad breath.

Persistent bleeding is a signal your gums need attention catching it at the gingivitis stage means a simple professional cleaning can usually sort it out completely. Left untreated, it can progress to periodontitis, which is harder to reverse.

Q3 Can I get gum disease even if I brush my teeth regularly?

Yes, absolutely brushing alone isn't enough to prevent gum disease, and it's actually quite common among people who consider themselves good brushers.

Why brushing isn't the whole story

The key culprit is plaque between your teeth. A toothbrush cleans the surfaces it can reach, but it can't get into the tight spaces between teeth or deep under the gumline. Plaque that hides there hardens into tartar (calculus) within 24 72 hours and once it hardens, only a dental professional can remove it.

Risk factors beyond brushing

Even with consistent brushing, gum disease can develop due to:

Skipping flossing this is the big one. Floss (or an interdental brush) is the only way to clean the contact points between teeth where gum disease often starts.

Brushing technique brushing too hard, too fast, or at the wrong angle can miss the gumline entirely.

Genetics some people are simply more prone to gum disease regardless of hygiene habits.

Smoking significantly raises risk and masks symptoms by reducing gum bleeding.

Hormonal changes pregnancy, puberty, and menopause can make gums more susceptible.

Certain medications some drugs reduce saliva flow or cause gum overgrowth.

Diabetes or immune conditions impair the body's ability to fight gum infections.

Skipping dental cleanings professional cleanings remove tartar that no home routine can touch.

Early signs to watch for.

Gums that bleed when you brush or floss.

Redness, swelling, or tenderness.

Persistent bad breath.

Gums pulling away from teeth.

The good news: gingivitis (the early stage) is fully reversible with improved hygiene and a professional cleaning. Left untreated, it can progress to periodontitis, which causes bone loss and is much harder to manage.

If you haven't had a cleaning in a while or notice any of those signs, a visit to a dentist or periodontist is worth prioritizing.

Q4 Can I have gum disease even if my gums don't hurt?

Yes, absolutely and this is one of the most important things to understand about gum disease. Pain is a poor indicator. Gum disease (periodontitis and its earlier stage, gingivitis) is often called a "silent" condition precisely because it typically progresses without any discomfort.

Why it doesn't hurt.

Gum disease develops gradually, and the early stages involve inflammation of soft tissue not the nerves that signal pain. By the time it becomes painful, the disease may already be quite advanced, with significant bone loss around the teeth.

Signs to watch for instead of pain.

Bleeding when brushing or flossing this is one of the earliest and most reliable warning signs.

Gums that look red, swollen, or puffy rather than firm and pale pink.

Gums pulling away from teeth (receding), making teeth look longer.

Persistent bad breath that doesn't go away.

Teeth that feel loose or shifting.

Pus between teeth and gums.

Changes in how your bite fits together.

Who's at higher risk.

Even with no symptoms, certain factors raise your risk significantly: smoking, diabetes, a family history of gum disease, dry mouth from medications, pregnancy, and poor oral hygiene.

What to do.

A dentist or periodontist can detect gum disease through a simple probing measurement of the pockets around each tooth deep pockets indicate bone loss even when you feel nothing. X-rays can also reveal bone loss invisible to the eye.

Regular dental check-ups (typically every 6 months) exist largely for this reason to catch problems that you genuinely cannot feel on your own. If it's been a while since your last visit, that's the most useful next step.

Q5 Can gum disease get better without treatment?

The answer depends on the stage of the disease.

Gingivitis (early stage) yes, it can reverse.

Gingivitis is inflammation confined to the gums, with no bone or tissue loss yet. At this stage the damage is fully reversible. Consistent brushing, flossing, and removing plaque buildup can clear it up completely, sometimes within a couple of weeks. Your gums go back to normal without any clinical intervention.

Periodontitis (advanced stage) no, it cannot.

Once gum disease progresses to periodontitis, the bone and connective tissue supporting your teeth have been destroyed. That loss is permanent the body does not regenerate it on its own. Without treatment, it steadily gets worse, not better. This stage needs professional intervention: deep cleaning (scaling and root planing), and in more advanced cases, surgery or bone grafting.

What makes the difference.

Plaque and tartar are the root cause. Plaque you can remove at home; tartar (calcified plaque) you cannot it requires a dentist's instruments.

Time matters a lot. Gingivitis that goes unaddressed for months can quietly cross into periodontitis, often without much pain, which is why it's frequently caught late.

Risk factors like smoking, diabetes, and dry mouth accelerate progression and make self-care less effective.

The practical takeaway: if your gums bleed when you brush but aren't painful, that's likely gingivitis and better oral hygiene now can genuinely reverse it. But if there's gum recession, teeth that feel loose, or persistent bad breath, those are signs the disease has advanced and a dentist visit isn't optional.

Q6 Are gum diseases rare?

Quite the opposite gum disease is remarkably common. It's one of the most widespread chronic conditions in the world.

A few key facts:

Nearly half of adults over 30 in the US have some form of periodontal (gum) disease, according to CDC data. That rises to about 70% of adults over 65.

Globally, severe gum disease affects roughly 19% of adults about 1 billion people making it the 11th most prevalent disease worldwide.

Mild to moderate forms (like gingivitis) are even more common; many dentists consider gingivitis nearly universal at some point in a person's life if oral hygiene lapses.

There are two main stages:

Gingivitis early stage, gums are inflamed and may bleed when brushing. Reversible with good hygiene and professional cleaning.

Periodontitis more advanced, where infection damages the bone and tissue holding teeth in place. This can lead to tooth loss and is *not* fully reversible, though it can be managed.

What makes gum disease particularly sneaky is that it's often painless in early stages, so people don't realize they have it. Risk factors include poor oral hygiene, smoking, diabetes, certain medications, and genetics.

The silver lining: when caught early, it's very treatable.

Q7 Is it normal to lose teeth because of gum disease as you get older?

Gum disease (periodontitis) is genuinely one of the leading causes of tooth loss in adults, and it does become more common with age but "normal" is a bit complicated here.

What's actually happening.

Gum disease is caused by bacterial plaque building up along and below the gumline. Over time, if untreated, it destroys the bone and connective tissue that hold teeth in place. Once enough support is

gone, teeth loosen and eventually fall out or need extraction. It's a slow, largely painless process, which is why people often don't notice until significant damage has occurred.

Why it seems age-related.

Cumulative damage Years of mild, undertreated inflammation quietly erode bone. The damage is incremental, so it shows up most dramatically later in life.

Medications Many common medications (for blood pressure, allergies, depression) cause dry mouth, which reduces saliva's natural antibacterial effect and accelerates gum disease.

Systemic conditions Diabetes, in particular, has a well-established bidirectional link with periodontitis. Both become more prevalent with age.

Reduced dexterity or access to care Can make consistent oral hygiene harder over time.

The key distinction: common vs. inevitable.

Losing teeth to gum disease is common in older adults, but it is not inevitable. It's a preventable and, in early stages, treatable disease. People who have consistent dental care, good oral hygiene, and manage risk factors (smoking is a major one) often keep their teeth well into old age.

Worth knowing.

If you or someone you're concerned about has noticed bleeding gums, receding gumline, loose teeth, or persistent bad breath, those are warning signs worth getting evaluated by a dentist or periodontist sooner rather than later earlier intervention makes a significant difference in outcomes. The bone that's lost doesn't grow back on its own.

Q8 If gum disease runs in my family, will I definitely get it too?

Not necessarily genetics loads the gun, but lifestyle pulls the trigger.

What the genetics actually mean.

Having a family history of gum disease (periodontitis) does raise your risk meaningfully. Studies suggest hereditary factors account for roughly 30–50% of susceptibility to periodontitis. Specific gene variants can affect how your immune system responds to the bacteria that cause gum disease, how much inflammation you produce, and how well your gum tissue holds up over time.

But genes set a *baseline risk*, not a destiny.

What matters just as much (or more).

The other 50–70% of your risk comes from modifiable factors:

Oral hygiene Plaque buildup is the primary trigger. Brushing twice daily and flossing consistently disrupts the bacterial environment that drives gum disease.

Smoking One of the strongest risk multipliers. Smokers are 2 7× more likely to develop periodontitis, and it masks early symptoms like bleeding.

Blood sugar control Diabetes and gum disease have a bidirectional relationship. Poorly controlled blood sugar significantly worsens gum health.

Stress Elevates cortisol, which suppresses immune response and worsens inflammation.

Diet A diet high in sugar feeds the bacteria involved; adequate vitamin C supports gum tissue integrity.

Regular dental cleanings Professional scaling removes tartar that brushing can't reach.

What to do with a family history.

Think of it as a heads-up, not a sentence. Useful steps:

Tell your dentist they can monitor you more closely, perhaps scheduling cleanings every 3 4 months instead of 6.

Know the early signs bleeding when brushing, gums pulling away from teeth, persistent bad breath. Early-stage gingivitis is fully reversible.

Be especially strict about quitting smoking if that's relevant to you.

Get blood sugar checked if you haven't recently.

People with high genetic susceptibility who maintain great habits often never develop significant disease while people with low genetic risk who neglect their oral health can develop severe periodontitis. The family history is worth knowing, but your daily choices carry more weight than it might feel like.

Q9 Can stress make me more likely to get gum disease?

Yes, stress genuinely does increase your risk of gum disease, and the connection is pretty well established. Here's how it works:

The stress-gum disease link.

Cortisol suppresses your immune system. When you're chronically stressed, elevated cortisol levels reduce your body's ability to fight off bacterial infections including the bacteria that cause gum disease (periodontitis). Your gums become more vulnerable to the same bugs that are always present in your mouth.

Inflammation goes haywire. Stress promotes systemic inflammation, and your gums are a prime target. This can accelerate the breakdown of gum tissue and the bone that supports your teeth.

Dry mouth. Stress (and many medications taken for anxiety or depression) reduces saliva flow. Saliva is your mouth's natural defense it neutralizes acids and washes away bacteria. Less saliva means more bacterial buildup.

The behavior side

Stress also changes what you *do*, which compounds the biological effects:

Neglecting oral hygiene brushing and flossing fall off the radar when you're overwhelmed.

Teeth grinding (bruxism) very common under stress, damages gum tissue and bone.

Smoking or drinking more both are independent risk factors for gum disease.

Worse diet more sugar, less nutrition, which feeds harmful bacteria.

What the research says.

Studies have found that people experiencing financial stress, work stress, or significant life events have measurably higher rates of periodontal disease. The relationship appears dose-dependent the more severe and prolonged the stress, the greater the risk.

The practical takeaway: if you're going through a stressful period, your gums deserve extra attention, not less. Keeping up with brushing, flossing, and regular dental cleanings becomes *more* important, not easier to skip. If you notice bleeding, swelling, or receding gums, it's worth mentioning to a dentist sooner rather than later.

Q10 Do gum diseases only affect the mouth and teeth?

No gum disease can have surprisingly far-reaching effects beyond the mouth. Here's what the research shows:

Systemic Conditions Linked to Gum Disease.

Cardiovascular disease is one of the most studied connections. The bacteria responsible for periodontitis can enter the bloodstream, trigger inflammation, and contribute to the buildup of arterial plaque raising the risk of heart attack and stroke.

Diabetes has a two-way relationship with gum disease. Uncontrolled blood sugar makes gum infections worse, and chronic gum inflammation can make blood sugar harder to control in return.

Respiratory disease is another link. Inhaling oral bacteria over time can contribute to lung infections, pneumonia, and may worsen chronic obstructive pulmonary disease (COPD).

Adverse pregnancy outcomes including preterm birth and low birth weight have been associated with severe periodontitis, likely because oral bacteria can trigger systemic inflammatory responses.

Cognitive decline and dementia are under active investigation. Some studies have found *Porphyromonas gingivalis* (a key pathogen in gum disease) in the brains of Alzheimer's patients, though causality is still debated.

Rheumatoid arthritis shares notable overlap both are chronic inflammatory conditions, and people with periodontitis have higher rates of RA, and vice versa.

Why the Connection Exists.

The main mechanisms are:

Bacteremia oral bacteria entering the bloodstream through inflamed gum tissue.

Systemic inflammation chronic low-grade inflammation from gum disease that affects the whole body.

Shared immune pathways periodontitis and many systemic diseases involve the same inflammatory molecules (like IL-6 and TNF-α).

The key takeaway: gum disease is a sign of a wider inflammatory burden on the body, not just a local dental problem. Treating it well has benefits that go well beyond a healthier smile.

Q11 Will a hard-bristled toothbrush clean my teeth better?

Not really and it can actually cause harm. Here's the breakdown:

Hard bristles don't clean better.

Plaque is a soft, sticky film. It doesn't require force to remove the *motion* and *coverage* of brushing matter far more than bristle stiffness. A soft-bristled brush used correctly removes plaque just as effectively.

What hard bristles actually do.

Abrade enamel enamel doesn't regenerate. Aggressive scrubbing with stiff bristles gradually wears it down, making teeth more sensitive and prone to decay.

Damage gums hard bristles can cause gum recession over time. Once gum tissue recedes, it doesn't grow back.

Cause cervical abrasion that's the notching or groove you sometimes see at the gumline, caused by repeated mechanical abrasion.

What dentists actually recommend.

A soft-bristled brush with proper technique: gentle, small circular or back-and-forth strokes, angling the bristles about 45° toward the gumline. Two minutes, twice a day. That's it.

The main culprit in poor cleaning isn't bristle hardness it's missed spots (especially the back molars and the gumline) and not brushing long enough.

If your teeth feel like they need more "scrubbing," that sensation is often about texture and habit rather than actual cleaning effectiveness. Switching to an electric toothbrush is a better upgrade than going harder the oscillating motion is clinically proven to outperform manual brushing for plaque removal.

Q12 Should I stop brushing my teeth for a few days if my gums are bleeding?

No actually the opposite is true. Stopping brushing when your gums bleed is one of the most common instincts people have, and it tends to make things worse.

Why gums bleed in the first place.

Bleeding gums are almost always a sign of inflammation caused by plaque buildup along the gumline. When plaque isn't removed regularly, bacteria irritate the gum tissue, making it swollen and prone to bleeding at the slightest contact. This early stage is called gingivitis.

Why you should keep brushing.

If you stop brushing, plaque accumulates even faster, the inflammation worsens, and the bleeding will get worse over time not better. Consistent, gentle brushing is actually the fix, not the problem.

A few things that help:

Brush twice a day with a soft-bristled brush firm bristles can aggravate already-sensitive gums.

Be gentle you don't need pressure; the bristles do the work.

Floss daily the gumline between teeth is where plaque hides and where gingivitis usually starts. Flossing may cause brief bleeding too, but stick with it.

Use a fluoride toothpaste, and consider one marketed for sensitive gums.

With consistent brushing and flossing, gingivitis-related bleeding typically clears up within 1 2 weeks.

When to see a dentist.

If the bleeding is heavy, persists beyond a couple of weeks of good oral hygiene, or you're also noticing gum recession, pain, or loose teeth, that's worth a dental visit. It could indicate periodontitis (more advanced gum disease) or occasionally an unrelated issue like a vitamin deficiency or medication side effect.

Q13 Do I need to brush my teeth after every meal to keep my gums healthy?

Brushing after every meal isn't strictly necessary, and in some cases can actually cause harm. Here's what the evidence supports:

The key recommendations:

Brush twice a day morning and before bed which is what most dental organizations recommend as the baseline for gum health.

Wait 30 60 minutes after eating before brushing, especially after acidic foods or drinks (citrus, soda, coffee). Acid temporarily softens enamel, and brushing right away can erode it.

Floss once a day this matters *more* for gum health than brushing frequency, since gum disease (gingivitis) typically starts between teeth where a brush can't reach.

What actually drives gum disease:

Plaque builds up over roughly 24 hours and hardens into tartar after about 48 72 hours. Brushing twice daily disrupts that cycle effectively. A third brush after lunch is fine if you want it, but it's not the deciding factor for gum health.

What does matter:

Technique gentle circles or the Bass method along the gumline, not aggressive scrubbing.

Flossing or using interdental brushes.

Using fluoride toothpaste.

Regular professional cleanings (every 6 12 months).

So no, you don't need to brush after every meal consistent twice-daily brushing with good technique, combined with daily flossing, is what keeps gums healthy. If you already have gum disease or are prone to it, a dentist might tailor that advice for your specific situation.

Q14 Even if I brush my teeth regularly, do I still need to get my gums checked?

Yes, absolutely brushing well maintains your tooth surfaces and helps with the gum line, but it doesn't give you a full picture of what's happening *beneath* the gums.

Here's why regular gum checks still matter:

What brushing can't do.

Brushing removes plaque from surfaces you can reach, but bacteria can accumulate in the *pockets* between your teeth and gums spaces a toothbrush simply can't access.

Tartar (hardened plaque) can only be removed professionally, and once it forms, it irritates gum tissue even in people who brush diligently.

What a gum check catches.

Pocket depth measurements a dentist or hygienist uses a small probe to measure the gap between tooth and gum. Deeper pockets signal gum disease (periodontitis) even when there's no pain.

Early gingivitis gum inflammation can be present with no obvious symptoms. Caught early, it's fully reversible.

Bone loss periodontitis can silently erode the bone supporting your teeth long before you notice anything wrong.

The tricky part: gum disease is often painless.

This is what catches a lot of careful brushers off guard. You can have progressing gum disease with no pain, no obvious swelling, and no bad breath and only find out when a dentist probes or takes an X-ray.

General guidance.

Most adults benefit from a professional cleaning and gum assessment at least every 6 months. If you have a history of gum disease, smoke, have diabetes, or are pregnant, more frequent checks (every 3 4 months) are often recommended.

So think of brushing as excellent *maintenance* and professional checks as the *monitoring* layer that catches what maintenance alone misses. They work together.

Q15 Is brushing my teeth once a day enough to keep my mouth healthy?

Once a day is better than nothing, but it's generally not enough for good oral health. Here's why:

What dentists actually recommend.

Brush twice a day morning and before bed. Nighttime brushing is the more critical one: saliva production drops while you sleep, which means bacteria have less natural defense to work against, so plaque that forms overnight does more damage.

Floss once a day brushing only cleans about 60% of tooth surfaces. The spaces between teeth and just below the gumline need floss (or an interdental brush) to clear the biofilm that leads to cavities and gum disease.

What happens with once-a-day brushing.

Plaque the sticky bacterial film on your teeth starts mineralizing into tartar within 24 72 hours. Once it hardens, brushing won't remove it; only a professional cleaning can. So brushing only once a day leaves longer windows for that process to begin, especially overnight. Over time this raises your risk of:

Cavities, particularly between teeth.

Gingivitis (early gum inflammation), which can progress to periodontitis.

Bad breath from bacterial activity.

What actually matters most.

If you're only going to brush once, make it before bed after your last meal. But ideally:

Brush for two full minutes, twice daily, with a fluoride toothpaste.

Floss once Daily.

See a dentist for a cleaning every 6 12 months.

Some people get away with less rigorous habits due to genetics, diet, and saliva composition but they're the exception, not the rule.

Q16 Can flossing cause gum recession?

Yes, flossing can cause gum recession but almost always because of *how* it's done, not because flossing itself is harmful.

How flossing leads to recession.

Aggressive technique is the main culprit. Snapping the floss hard down into the gum line, or sawing it back and forth with too much force, creates repetitive micro-trauma to the gingival tissue. Over time, that trauma causes the gums to pull back.

Specific habits that cause problems:

Snapping the floss between teeth and letting it smack the gumline.

Flossing too forcefully against the gum margin.

Over-flossing multiple times a day with heavy pressure.

Using the wrong tool older, thicker flosses or poorly-handled floss picks can be harder to control.

Why proper flossing doesn't cause recession.

Done correctly, flossing is one of the best ways to *prevent* gum recession, because it removes the bacteria that cause periodontal disease which is itself a leading cause of recession.

The correct technique:

Curve the floss into a C-shape around the tooth.

Slide it gently below the gumline, not into it.

Move it up and down against the tooth surface, not the gum.

Use a clean section for each tooth.

When to mention it to a dentist.

If you're already noticing recession, sensitivity at the gumline, or your gums seem to be pulling back despite gentle technique, it's worth a dental visit. Recession from mechanical trauma doesn't reverse on its own, and a dentist can assess whether the cause is flossing, brushing (another common culprit), or something like bruxism or gum disease.

The short version: the problem is almost never *that* you're flossing it's *how*.

Q17 Do electric toothbrushes damage the gums more than regular toothbrushes?

The short answer: no, not if used correctly and in many ways electric toothbrushes are *gentler* on gums than manual ones.

Where the concern comes from.

People often press harder with a manual toothbrush without realizing it, which is a leading cause of gum recession and enamel wear. Electric toothbrushes especially oscillating-rotating models (like Oral-B) do the scrubbing work for you, so there's less temptation to scrub aggressively. Many models have a pressure sensor that warns you when you're pushing too hard, which manual brushes obviously can't do.

What the evidence says.

Multiple clinical studies and systematic reviews consistently find that electric toothbrushes reduce plaque and gingivitis more effectively than manual brushes over time. Healthier gums from better plaque removal means *less* gum disease, not more damage.

When electric toothbrushes *can* cause irritation.

Technique errors pressing down hard and dragging the brush defeats the purpose; you should guide it gently from tooth to tooth.

Wrong brush head a medium or hard bristle head is too aggressive; always use soft bristles.

Already-receded or sensitive gums the vibration can feel intense at first, but this usually settles within a week or two.

Overuse of whitening/abrasive modes some brush heads or modes are more abrasive; daily use of aggressive settings on sensitive gums can cause irritation.

Bottom line.

For most people, an electric toothbrush is at least as safe as manual, and often better for gum health provided you use a soft brush head, let the brush do the work, and don't press hard. If you already have significant gum recession or sensitivity, it's worth checking with a dentist before switching, since they may have a preference based on your specific situation.

Q18 Would using mouthwash alone be enough without brushing my teeth?

No, mouthwash alone isn't enough and it's a pretty significant gap, not just a minor shortcut.

What mouthwash actually does.

Mouthwash kills bacteria and freshens breath, and some formulas deliver fluoride or target gum inflammation. It's genuinely useful as an *addition* to brushing. The problem is it's a liquid that rinses over surfaces it can't physically remove what's stuck to your teeth.

What it can't replace.

Brushing does things mouthwash simply cannot:

Removes plaque mechanically Plaque is a sticky biofilm that clings to tooth surfaces. Rinsing doesn't dislodge it; you have to physically scrub it off.

Prevents tartar buildup Plaque left in place hardens into tartar (calculus) within 24 72 hours. Once that happens, only a dental professional can remove it.

Cleans the gumline The area where teeth meet gums traps debris and bacteria. Brushing disrupts that accumulation; mouthwash mostly washes over it.

The consequences of skipping brushing.

If you stopped brushing and relied only on mouthwash, over weeks to months you'd likely see:

Accelerated plaque and tartar buildup.

Increased risk of cavities, since undisturbed plaque produces acids that erode enamel.

Gum disease (gingivitis progressing to periodontitis).

Bad breath that the mouthwash masks temporarily but doesn't address at its source.

The analogy.

Think of it like rinsing a dirty dish with water instead of scrubbing it. The rinse might wash off loose bits and smell better, but the stuck-on residue remains.

Mouthwash is a great complement use it after brushing. But it's firmly in the supporting role, not the lead.

Q19 After I quit smoking, my gums started bleeding. Is that normal?

Yes, this is actually quite common and has a well-understood cause though it's still worth paying attention to.

Why quitting smoking causes bleeding gums.

Nicotine is a vasoconstrictor, meaning it narrows blood vessels. While you were smoking, reduced blood flow to your gum tissue was *masking* inflammation your gums were likely already affected by gum disease (gingivitis or early periodontitis), but the restricted circulation kept the bleeding suppressed.

Once you quit, blood flow normalizes and rebounds. Your gums become better perfused, and any underlying inflammation finally shows up as bleeding. It's almost paradoxical: the bleeding is partly a sign that your circulation is *recovering*.

What to expect.

Bleeding typically peaks in the first few weeks after quitting and then gradually improves.

As your body continues to heal, gum tissue health usually improves significantly ex-smokers' gum health can approach that of people who never smoked over time.

The bleeding should diminish, not worsen, over weeks to months.

When to see a dentist.

You should get it checked out if:

Bleeding is heavy or persistent beyond a few weeks.

You have pain, swelling, or receding gums.

Gums bleed spontaneously (not just when brushing).

You notice loose teeth or changes in bite.

These could point to periodontitis that needs professional treatment which is much more effective now that you've quit smoking, since smoking is one of the biggest risk factors for gum disease progression.

The bottom line: mild bleeding shortly after quitting is expected and a sign your body is healing. Keep brushing gently twice a day, floss regularly, and schedule a dental cleaning if you haven't had one recently your dentist will be able to assess your baseline gum health and clear out any built-up plaque or tartar that's contributing to the inflammation.

Q20 Can natural products effectively keep my gums healthy?

Used Web Search.

Yes, several natural products have genuine evidence behind them though they work best as *complements* to brushing and flossing, not replacements for professional care.

What the research supports.

Saltwater rinses are one of the most well-validated options. Salt creates a temporarily hostile environment for bacteria and reduces inflammation. Rinsing with warm salt water (about ½ tsp per cup of water) a few times a day can meaningfully ease gingivitis symptoms.

Oil pulling swishing coconut or sesame oil for 10–20 minutes has shown in multiple studies to reduce plaque and harmful bacteria (particularly *Streptococcus mutans*) comparably to chlorhexidine mouthwash in some trials. The mechanical action of swishing is likely a big part of why it works.

Turmeric (curcumin) has solid anti-inflammatory and antimicrobial properties. Used as a gel or paste applied to the gums, it's been shown in clinical studies to reduce gingival inflammation, sometimes as effectively as standard medicated gels.

Tea tree oil is a potent natural antiseptic. Diluted versions (never swallowed) can reduce bleeding and bacterial load, though it needs to be used carefully concentrated amounts are irritating and toxic if ingested.

Aloe vera gel applied directly to irritated gum tissue can soothe inflammation and has demonstrated some antibacterial activity in trials.

Green tea contains catechins that inhibit bacteria associated with periodontal disease. Drinking it regularly (unsweetened) is associated with better gum health in observational studies.

Diet also plays a real role.

Vitamin C (citrus, bell peppers, strawberries) is essential for collagen synthesis gum tissue literally depends on it. Deficiency directly causes gum problems.

Crunchy vegetables like celery and carrots mechanically clean teeth surfaces and stimulate saliva, which is your mouth's natural antibacterial defense.

Reducing sugar is arguably the single most impactful dietary change it starves the bacteria that cause plaque and gum disease.

The honest caveat.

Natural products are genuinely useful for *prevention* and mild gingivitis, but once gum disease progresses beyond the early stage causing bone loss or deep pockets no home remedy reverses that. At that point, professional intervention (scaling, root planing) is necessary. Regular dental cleanings remain the cornerstone, and natural approaches work best layered on top of that foundation.

Q21 Can getting a dental scaling damage my teeth?

Dental scaling is generally very safe, but it's a fair thing to wonder about the scraping sensation can feel intense.

What scaling actually does.

Scaling removes hardened tartar (calculus) and plaque from the tooth surface and below the gumline. The instruments either metal scalers or an ultrasonic device are designed to target deposits *on* the enamel, not the enamel itself.

Can it cause damage?

In the hands of a trained hygienist or dentist, permanent damage is unlikely, but there are some real, temporary effects to know about:

Sensitivity The most common side effect. Removing tartar exposes areas of the tooth that were covered, and gums may temporarily recede slightly after a deep clean. Sensitivity usually fades within a few days to a couple of weeks.

Gum soreness and bleeding Normal after scaling, especially if tartar buildup was significant or gums were already inflamed. This settles quickly.

Microscopic enamel effects Studies show that repeated, aggressive scaling *can* cause minor surface scratching, particularly with metal instruments. This is why a skilled hand and appropriate instrument selection matter. In practice, the damage from *not* removing tartar (gum disease, bone loss, decay) far outweighs this risk.

Root surface In deep scaling (root planing), the root surface is intentionally smoothed to remove infected tissue. This is controlled and therapeutic, not destructive.

When to be more cautious.

If you have thin enamel or existing sensitivity, mention it beforehand the hygienist can adjust pressure and tool choice.

Ultrasonic scalers are generally gentler on enamel than hand instruments for routine scaling.

Going very long between cleanings means more aggressive work is needed each time, which increases the temporary discomfort.

Bottom line.

For the vast majority of people, the risk of *not* scaling progressive gum disease, bone loss, and eventually tooth loss is far greater than any risk from the procedure itself. If you've had a particularly rough experience or your teeth feel worse afterward for more than a week or two, it's worth flagging to your dentist.

Q22 Can dental scaling cause gaps between my teeth?

Dental scaling doesn't actually *create* gaps but it can make existing ones more visible, which is a very common source of confusion.

What's really happening.

Before scaling, tartar (calculus) builds up between and around your teeth, filling in the spaces near the gumline. It can act like a false "filler," making teeth look tight and closely packed. Once that buildup is removed, the space that was always there becomes apparent so it *feels* like gaps appeared, but they were hidden before.

Why gums also play a role.

Tartar buildup usually comes with gum inflammation. Inflamed gums are puffy and swollen, which also masks gaps. After scaling:

Inflammation resolves over 1 2 weeks.

Gums recede slightly back to their healthy position.

This can expose more of the tooth and reveal spaces between teeth (called "black triangles" near the gumline).

This is especially noticeable if you had moderate to advanced gum disease, because the underlying bone and gum tissue may have already been lost scaling just reveals what the swelling was covering up.

When to be reassured vs. concerned.

Normal: Slight visibility of gaps after cleaning that weren't obvious before this resolves or stabilizes within a few weeks.

Worth discussing with your dentist: If gaps feel significantly larger, teeth feel loose, or you have persistent sensitivity these could signal gum disease that needs further treatment.

The underlying message is actually positive: scaling is revealing your *true* gum health. The gaps aren't new damage; they're the baseline that was obscured by tartar and swelling.

Q23 Do all gum diseases require the same treatment?

No gum diseases vary quite a bit in severity and type, and treatment is matched accordingly.

The Main Categories.

Gingivitis is the mildest and most common form. It's inflammation limited to the gum tissue itself, with no bone loss. It's also fully reversible with:

A professional cleaning (scaling).

Improved brushing and flossing at home.

Periodontitis is more serious. Infection has spread below the gumline, destroying the bone and connective tissue that hold teeth in place. Treatment depends on how advanced it is:

Stage.	Typical Treatment.
Mild moderate.	Deep cleaning: scaling and root planing (SRP) removing plaque and tartar from below the gumline.
Moderate severe.	SRP plus antibiotics (topical or oral), or laser therapy.
Advanced.	Periodontal surgery flap surgery to access deep pockets, bone grafting, or guided tissue regeneration.

Necrotizing periodontal disease (e.g., ANUG/ANUP) is a distinct, aggressive form linked to immune compromise, stress, or malnutrition. It requires urgent debridement, antiseptic rinses (chlorhexidine), and often systemic antibiotics (typically metronidazole).

Peri-implantitis is essentially periodontitis around a dental implant. Treatment follows similar principles but is complicated by the implant surface, and may involve specialized mechanical debridement, surface decontamination, or implant removal in severe cases.

Key Variables That Shape Treatment.

Pocket depth how deep the space between tooth and gum has become.

Bone loss visible on X-rays; determines surgical need.

Systemic factors diabetes, smoking, and immune disorders both worsen gum disease *and* reduce treatment response.

Cause some cases are driven by specific bacteria, requiring targeted antibiotics; others are primarily mechanical.

The bottom line: gingivitis can often be resolved with a single cleaning and better hygiene, while advanced periodontitis may involve months of treatment across multiple visits. Getting an accurate diagnosis including probing depths and X-rays is what determines the right path.

Q24 Is gum treatment painful?

It depends on the type and stage of treatment, but here's an honest breakdown:

Mild to Moderate Discomfort Most Common.

For the most common gum treatments, pain is generally manageable:

Professional cleaning (scaling) Can feel uncomfortable, especially if you have sensitive gums or significant buildup, but it's rarely described as truly painful.

Deep cleaning (scaling and root planing) Your dentist or periodontist will use local anesthesia, so you won't feel pain during the procedure. Afterward, expect 1 3 days of soreness, sensitivity, and mild aching ibuprofen or acetaminophen typically handles it well.

More Significant Discomfort Surgical Treatments.

If your gum disease is advanced, surgery may be needed:

Flap surgery Done under local anesthesia, so no pain during. Post-op soreness lasts a few days to a week, and you'll likely be prescribed pain relief.

Gum grafts The donor site (usually the roof of your mouth) tends to be the sorest part. Most patients describe it as similar to a pizza burn but more prolonged uncomfortable for about a week.

What Makes It Feel Worse.

Going in with already inflamed, sensitive gums they bleed and hurt more easily.

Delaying treatment the more advanced the disease, the more involved (and uncomfortable) the treatment.

The Good News.

Most people say the anticipation is worse than the reality, especially with modern anesthesia and techniques. Dentists can also offer sedation if anxiety is a concern.

The real pain tends to come from *avoiding* treatment advanced gum disease can cause bone loss, tooth loss, and chronic infection, all of which are far more uncomfortable to deal with.

Q25 Can gum surgery cause tooth loss?

Gum surgery itself, when performed correctly, is actually aimed at saving teeth not losing them. But there are some nuances worth knowing.

How gum surgery protects teeth.

Procedures like flap surgery, bone grafting, or gum grafts are typically done to treat advanced periodontal (gum) disease. Left untreated, that disease *will* cause tooth loss by destroying the bone and tissue holding teeth in place. Surgery halts that destruction.

Can tooth loss happen anyway?

Yes, in certain situations:

Pre-existing severe damage If a tooth's supporting bone is already too far gone before surgery, the dentist or periodontist may determine the tooth is unrestorable and extract it at the same time. This isn't caused by the surgery the tooth was already lost to disease.

Surgical complications Rare, but infection, poor healing, or accidental damage to surrounding structures can occasionally compromise a tooth. This risk is much higher when surgery is performed on a tooth with a poor prognosis to begin with.

Post-surgical infection If aftercare instructions aren't followed and infection sets in, surrounding teeth can be affected.

Healing response in compromised patients People with diabetes, autoimmune conditions, or who smoke heal more slowly and face higher complication risks.

The bottom line.

In a healthy patient with a skilled periodontist, gum surgery carries a very low risk of causing tooth loss on its own. The bigger risk is *not* treating gum disease that's what reliably leads to losing teeth.

If you're facing a specific procedure and have concerns, it's worth asking your periodontist directly about the prognosis for each individual tooth before going in. They can give you a realistic picture based on your X-rays and bone levels.

Q26 Can gum treatment make my teeth feel loose?

Yes, this is actually a well-known and somewhat counterintuitive side effect of gum (periodontal) treatment and it's usually temporary.

Why teeth can feel loose after gum treatment.

Before treatment, inflamed gum tissue is often swollen and puffy, which can actually *brace* the teeth and mask underlying bone loss. Once treatment reduces that inflammation, the puffiness goes away, and you may suddenly notice the looseness that was already there.

Scaling and root planing (deep cleaning) is the most common culprit. It removes tartar and bacteria from deep below the gumline, which:

Temporarily irritates the surrounding tissues.

Reduces the swollen "cushion" effect of inflamed gums.

Can make teeth feel more mobile right after the procedure.

Bone loss from periodontitis is the real underlying issue. Advanced gum disease erodes the bone that holds teeth in place. Treatment stops the damage from progressing, but it doesn't restore lost bone so if significant bone loss was already present, some mobility may be a permanent reality.

When is it concerning vs. normal?

Situation.	What it means.
Mild looseness after deep cleaning.	Usually normal, often resolves in a few weeks.
Looseness that improves over months.	Healing is progressing well.
Looseness that gets *worse* after treatment.	Worth a follow-up may indicate ongoing infection or bone loss.
Severe mobility that doesn't settle.	Tooth may be at risk; discuss options with your periodontist.

What helps.

Sticking to the prescribed follow-up visits and maintenance cleanings.

Good home hygiene (brushing, flossing, interdental brushes).

Avoiding hard or crunchy foods on particularly mobile teeth while healing.

In some cases, a bite splint if clenching/grinding is adding stress to loose teeth.

The key reassurance: treatment stopping the disease progression is what protects the teeth long-term, even if the short-term sensation is unsettling. If the looseness is severe or worsening, it's worth calling your dentist or periodontist they can assess whether it's within the expected healing range or needs intervention.

Q27 Does tartar removal cause more tartar buildup?

The short answer is no tartar removal doesn't cause more tartar to build up. That's a myth worth unpacking.

Where the idea comes from.

After a professional cleaning, teeth can *feel* rougher or more sensitive, and some people notice buildup returning seemingly faster than before. This leads to the intuition that scraping "damaged" the tooth surface or "opened it up" to more tartar. Neither is accurate.

What's actually happening.

Tartar (calculus) forms from plaque, which is the soft bacterial film that coats teeth daily. When plaque isn't removed by brushing and flossing, it mineralizes usually within 24 72 hours and hardens into tartar.

Cleaning removes tartar but doesn't change the underlying biology. Your saliva composition, diet, oral bacteria, and brushing habits are what drive plaque and tartar formation not whether you've had a recent cleaning.

Smooth enamel actually resists buildup better. After a professional cleaning, teeth are smoother, which makes it *harder* for plaque to adhere, not easier.

Why tartar might seem to return quickly.

You're paying more attention to your teeth after a visit.

The cleaned baseline makes new buildup more noticeable by contrast.

If your oral hygiene habits haven't changed, tartar will return at roughly the same rate it always did.

The actual drivers of tartar rate.

Some people genuinely accumulate tartar faster than others. The factors behind that are:

Saliva chemistry higher calcium/phosphate concentration accelerates mineralization.

Oral bacteria composition.

Diet (high sugar/starch more plaque fuel).

Dry mouth less saliva means less natural rinsing.

Brushing and flossing frequency.

Regular cleanings (typically every 6 months, or more often if you're a fast builder) are the right response to tartar not avoiding them out of fear they'll make things worse.

Q28 Are antibiotics enough to treat gum disease?

Antibiotics alone are generally not enough to treat gum disease they're a supporting tool, not a standalone cure.

Why Antibiotics Can't Do It Alone.

Gum disease (periodontitis) is driven by bacterial biofilm a structured community of bacteria embedded in plaque and tartar that physically adheres to your teeth and below the gumline. Antibiotics circulating in your bloodstream (or even applied topically) can't penetrate and disrupt that biofilm effectively. The bacteria essentially hide inside it.

The foundation of treatment is mechanical removal scaling and root planing (a deep cleaning that goes below the gumline). Without that, antibiotics just temporarily suppress some bacteria, which quickly rebound.

Where Antibiotics *Do* Help.

They're genuinely useful in specific situations:

Aggressive periodontitis particularly fast-progressing or early-onset cases.

Acute infections like a periodontal abscess.

As an adjunct to scaling and root planing in moderate-to-severe cases, where studies show they improve outcomes beyond mechanical treatment alone.

Immunocompromised patients who need extra support.

Common options include doxycycline (sometimes in low-dose form, which works more by reducing inflammation than killing bacteria), metronidazole, or combination therapy with amoxicillin.

The Bigger Picture.

Even after successful treatment, gum disease tends to recur without ongoing maintenance. Regular professional cleanings (every 3 4 months for periodontitis patients), good home hygiene, and addressing risk factors like smoking or blood sugar control matter more long-term than any antibiotic course.

If you're dealing with gum disease, a periodontist can assess the severity and determine whether antibiotics make sense as part of not a replacement for a proper treatment plan.

Q29 After gum disease treatment, will I ever need treatment again?

Gum disease (periodontitis) is a chronic condition, which is the key thing to understand: it can be controlled very effectively, but it doesn't have a one-time cure. Here's what that means in practice:

After Treatment, You'll Need Ongoing Maintenance.

Once active treatment is complete whether that's scaling and root planing, surgery, or both the disease goes into remission, not elimination. The bacteria that caused it are still in your mouth, and without continued care, they'll recolonize and the disease will return.

Periodontal maintenance visits (usually every 3 4 months, at least initially) replace your regular dental cleanings. These are more thorough than standard hygiene appointments and specifically target the areas where disease was active. Over time, if your condition stays stable, some patients can eventually move to 6-month intervals but many stay on 3 4 months long-term.

What Affects Whether It Returns.

Home care Consistent, thorough brushing and flossing is non-negotiable. Gum disease is largely driven by bacterial plaque, so daily removal is your first line of defense.

Smoking One of the biggest risk factors for recurrence and poorer treatment outcomes.

Diabetes and other systemic conditions These are closely linked to gum disease; controlling them helps control periodontal health.

Genetics Some people are simply more susceptible, regardless of how well they care for their teeth.

Stress Affects immune response and can contribute to flare-ups.

The Realistic Picture.

Studies show that patients who stick to regular periodontal maintenance have dramatically lower rates of tooth loss and disease progression compared to those who skip it. The maintenance phase *is* the treatment continuing it's not just a check-up.

Think of it like managing blood pressure: medication and lifestyle changes control it, but stopping care lets it creep back up. The goal shifts from "cure" to "control," and for most people, that control is very achievable.

Your periodontist or dentist should give you a personalized maintenance schedule based on how severe your disease was, how you responded to treatment, and your individual risk factors.

Q30 Is surgery always necessary for gum treatment?

No, surgery isn't always necessary for gum (periodontal) disease. Whether it's needed depends mainly on how advanced the condition is.

Non-Surgical Treatments (Usually the First Step).

Scaling and root planing (deep cleaning) is the cornerstone of gum treatment for most people. A dentist or periodontist removes plaque and tartar from below the gumline and smooths the root surfaces so bacteria have less to cling to. This alone resolves many cases of early-to-moderate gum disease.

Other non-surgical options include:

Improved home care consistent brushing, flossing, and interdental cleaning.

Antibiotics topical gels placed in gum pockets, or oral antibiotics, to control bacterial infection.

Antiseptic rinses prescription chlorhexidine mouthwash to reduce bacteria.

Laser therapy some practices use lasers to remove infected tissue without cutting (less invasive than traditional surgery).

When Surgery Becomes Necessary.

Surgery is typically considered when non-surgical treatment hasn't been enough, or when disease is already severe:

Pocket depth reduction (flap surgery) gum tissue is folded back to clean deep deposits that scaling can't reach.

Bone grafting to rebuild bone lost to disease.

Soft tissue (gum) grafts to cover exposed roots or restore receded gum tissue.

Guided tissue regeneration to encourage regrowth of bone and tissue.

The Key Principle.

Gum disease is a spectrum. Gingivitis (early stage red, bleeding gums, no bone loss) is fully reversible with professional cleaning and better home care no surgery needed. Periodontitis (bone and tissue loss) may or may not require surgery depending on severity and how the gums respond to initial treatment.

Most periodontists follow a "conservative first" approach: try non-surgical treatment, reassess in 6 8 weeks, and only proceed to surgery if needed. Catching it early is the surest way to avoid the operating table.
