# Supplementary material for: Comparative Evaluation of Large Language Models in Responding to Common Misconceptions Regarding Periodontal Disease and Oral Hygiene
Source: Diagnostics (Basel). 2026 Sep 28;16(19):3158. doi: 10.3390/diagnostics16193158 (PMC13664004; doi:10.3390/diagnostics16193158)
Supplement: Supplementary file 1 [file diagnostics-16-03158-s001.zip › Supplementary File S5 LLMs response stylistic elements removed.zip!/1. LLMs response/DeepSeek V4.pdf]

Q1 Do gum diseases only affect elderly people?

No, gum diseases do not only affect elderly people. While the risk and prevalence of gum disease increase with age due to factors like cumulative plaque buildup, medications that reduce saliva flow, or other health conditions, people of all ages including children and young adults can develop gum disease. Early-stage gum disease (gingivitis) is common even in teenagers and young adults, often due to poor oral hygiene, hormonal changes (e.g., during pregnancy or puberty), smoking, or certain illnesses like diabetes. If left untreated, it can progress to periodontitis, which is more severe but still not exclusive to the elderly. So, good oral hygiene and regular dental checkups are important at every age.

Q2 Is it normal if my gums bleed when I brush my teeth?

It's common, but not considered "normal" in the sense of healthy. Occasional, very light bleeding might happen if you've just started flossing or brushing more vigorously, but regular bleeding when brushing is usually a sign of gum inflammation (gingivitis). The most common cause is plaque buildup along the gumline, which irritates the gums. Other possible causes include brushing too hard, using a hard-bristled toothbrush, or starting a new oral hygiene routine. If bleeding persists for more than a week despite gentle, thorough brushing and flossing, you should see a dentist to rule out periodontitis or other issues. In the meantime, try a soft-bristled brush and gentle circular motions rather than scrubbing.

Q3 Can I get gum disease even if I brush my teeth regularly?

Yes, it is possible to develop gum disease even if you brush regularly. Brushing alone cannot remove plaque and food debris from between teeth or below the gumline. Common reasons include:

Inadequate technique Missing the gumline, brushing too hard, or not long enough.

No flossing or interdental cleaning Brushing misses about 40% of tooth surfaces.

Genetics Some people are more susceptible to gum disease regardless of oral hygiene.

Medical conditions Diabetes, hormonal changes (pregnancy, menopause), and immune disorders increase risk.

Medications Antidepressants, antihistamines, or blood pressure drugs can reduce saliva flow, promoting bacterial growth.

Smoking or tobacco use Impairs gum healing and immune response.

Crooked teeth or dental appliances Create hard-to-clean areas.

So while regular brushing is essential, it must be part of a complete routine: proper technique, daily flossing or water flossing, regular dental cleanings, and managing overall health risks. If you notice bleeding, redness, or bad breath despite good brushing, see a dentist.

Q4 Can I have gum disease even if my gums don't hurt?

Yes, it is absolutely possible to have gum disease even if your gums don't hurt. In fact, the early and even moderate stages of gum disease (gingivitis and periodontitis) are often painless. This is why many people are unaware they have it.

Here's what to look for instead of pain:

Bleeding when brushing or flossing This is usually the first sign, even without discomfort.

Swollen, red, or shiny gums Healthy gums are pink and firm.

Receding gums Teeth may appear longer.

Persistent bad breath or bad taste Caused by bacteria.

Loose teeth or changes in your bite A later sign, but still often not painful.

Pus between teeth and gums A sign of infection, though not always painful.

Why no pain?
The bacteria that cause gum disease primarily damage the bone and supporting structures around teeth, which have fewer nerve endings than the gums themselves. Pain typically appears only in advanced stages, when an abscess forms or teeth become loose and shift.

What to do:
If you notice any of the above signs, see a dentist or periodontist even without pain. Early gum disease (gingivitis) is reversible with professional cleaning and improved home care. Without treatment, it can progress to periodontitis, leading to bone loss and tooth loss – still often without significant pain.

So don't wait for pain to act. Regular dental checkups are essential for catching gum disease early.

Q5 Can gum disease get better without treatment?

This is a great question, and the answer depends entirely on what stage of gum disease you're referring to. Here's the straightforward breakdown:

Gingivitis (Early, Reversible Stage): Yes, it can get better without professional treatment, but only if you immediately start a perfect at-home oral hygiene routine (thorough brushing and flossing). Gingivitis is an inflammation of the gums caused by plaque. It is not an infection of the bone. If you remove the plaque, your body can heal the inflammation on its own.

Periodontitis (Advanced, Destructive Stage): No, this will not get better without treatment. In fact, it will almost certainly get worse. Periodontitis is when the inflammation has spread to the bone and ligaments holding your teeth in place. This creates "pockets" around the teeth that are impossible to clean with a toothbrush or floss. The bone loss is permanent, and without professional cleaning (scaling and root planing), the infection will continue, leading to loosening and eventual loss of teeth.

The Critical Danger: Periodontitis is often painless until it's very advanced. You cannot "feel" bone loss. Many people assume their gum disease is getting better because it doesn't hurt, but the infection is actively destroying the support for their teeth.

Bottom Line: If your gums bleed when you brush but aren't receded, you can try 1-2 weeks of meticulous home care. If the bleeding stops, you reversed it. If you have any gum recession, loose teeth, or persistent bad breath, you need a dentist. Waiting will only make treatment more expensive and invasive.

Q6 Are gum diseases rare?

Gum diseases are not rare; they are very common. In fact, according to the CDC and WHO, nearly half of adults aged 30 and older have some form of periodontal (gum) disease. The mildest form, gingivitis, is even more widespread. Severe periodontitis, while less common, still affects about 9% of adults globally. So, while severe cases are less frequent, gum disease overall is one of the most prevalent human health conditions.

Q7 Is it normal to lose teeth because of gum disease as you get older?

This is a great question, and it gets at a common misunderstanding about aging and oral health.

The short answer is: No, it is not normal to lose teeth simply because you are getting older. Tooth loss is primarily caused by disease (like gum disease and cavities), not by age itself.

However, it is very common for older adults to lose teeth due to the *cumulative effects* of untreated gum disease over many years.

Let's break that down.

The "Normal" Aging Mouth vs. Disease.

A healthy mouth, even at 90 years old, should be able to retain natural teeth. The normal, age-related changes that *do* happen are minor:

Gums may recede slightly (exposing a bit more of the tooth root).

The bite force may decrease a little.

Existing fillings may weaken or crack.

None of these normal changes cause tooth loss.

The Real Culprit: Gum Disease (Periodontitis).

Gum disease is an infection of the tissues that hold your teeth in place. It starts as gingivitis (swollen, bleeding gums) and, if not treated, progresses to periodontitis.

Here's how periodontitis causes tooth loss, which becomes more common with age because the disease has had decades to progress:

Inflammation & Bone Loss: The bacterial infection causes your body's immune system to attack not just the bacteria, but also the bone and ligament that anchor your teeth.

Deepening "Pockets" : The gums pull away from the teeth, forming infected pockets that are impossible to clean with normal brushing.

Losing the Anchor: As the supporting bone is destroyed, the tooth becomes loose. There's simply not enough structure left to hold it firmly in place.

Tooth Loss: Eventually, the tooth becomes so mobile that it falls out or requires extraction.

So Why Does it Seem Like Older People Lose Teeth?

Because the risk factors for gum disease accumulate over a lifetime:

Duration of Neglect: Minor daily plaque buildup that isn't perfectly cleaned turns into tartar (calculus), which leads to worsening gum disease year after year.

Access to Care: People may have gone decades without regular dental cleanings, which are needed to remove tartar and catch early disease.

Other Age-Related Factors:

Dry Mouth (Xerostomia): Very common in older adults due to medications for blood pressure, depression, or allergies. Saliva is your mouth's natural cleanser and neutralizes acid. Without it, bacteria thrive, accelerating gum disease and cavities.

Chronic Diseases: Conditions like diabetes (especially if poorly controlled) dramatically increase the severity of gum disease.

Physical Limitations: Arthritis or vision problems can make brushing and flossing effectively very difficult.

Smoking: A long-term smoking habit is the single biggest risk factor for severe gum disease.

The Good News: It's Preventable.

Losing teeth from gum disease is not inevitable. It is a preventable and manageable disease, even as you get older.

You can keep your teeth for life if you manage gum disease.

Even if you have advanced gum disease, treatments (deep cleanings called scaling and root planing, medications, or surgery) can stop the disease and save your teeth.

When to See a Dentist Immediately.

Don't wait for loose teeth. See a dentist if you notice these early signs of gum disease:

Gums that bleed when you brush or floss (even gently).

Red, swollen, or tender gums.

Gums that are pulling away from your teeth (making teeth look longer).

Persistent bad breath or a bad taste in your mouth.

Teeth that feel loose or seem to have shifted.

Summary Table: Normal vs. Not Normal.

	Normal Aging.	Not Normal (Likely Gum Disease).
Losing Teeth.	No, it is not a required part of aging.	Yes, this is the primary sign of advanced, untreated disease.
Gums.	May recede *slightly* but don't bleed.	Bleeding, red, swollen, or pulling far away from teeth.
Bone Support.	Remains stable and healthy.	Progressive destruction of the jawbone.
Prevention.	Regular hygiene and checkups maintain teeth for life.	Requires professional treatment to stop the disease.

Conclusion: It is common for older people to have lost teeth to long-term, untreated gum disease, but it is not normal for aging itself to cause it. You absolutely can and should expect to keep your natural teeth for your entire life with good daily hygiene and regular professional dental care.

Q8 If gum disease runs in my family, will I definitely get it too?

That's a very understandable concern. Here’s the straightforward answer: No, you will not definitely get gum disease just because it runs in your family.

Think of it this way: family history loads the gun, but your daily habits and environment pull the trigger.

Here's a breakdown of why genetics are not destiny when it comes to gum disease:

Genetics Play a Role, But Not the Only Role.

Research suggests that about 30% of the population may be genetically susceptible to gum disease. This means you might have inherited:

An over-aggressive immune response: Your body may overreact to even small amounts of plaque bacteria, causing excessive inflammation and tissue damage.

Specific bacterial strains: You might be more likely to host the more aggressive bacteria that cause periodontitis.

However, even with these genetic factors, you won't develop gum disease without the initial trigger: dental plaque.

The "Shared Environment" Factor is Huge.

What we often call "family history" isn't just DNA. It's also shared habits and environments:

Oral hygiene habits: Families often share similar brushing, flossing, and dental visit patterns.

Diet: High sugar intake or frequent snacking feeds plaque bacteria.

Other health factors: Smoking, uncontrolled diabetes, and stress which also have family patterns are major risk factors.

So, your family members might have gum disease because they all share learned habits, not just shared genes.

You Can Break the Chain.

While you can't change your genes, you have enormous control over the other factors. To dramatically lower your risk (even with a family history), focus on what *is* in your control:

Perfect your home care: Brush twice daily for two minutes with fluoride toothpaste, and floss daily (this is non-negotiable for preventing gum disease).

Get professional cleanings: See your dentist or hygienist every 6 months, or every 3-4 months if they recommend it for "maintenance." They can detect the earliest signs (gingivitis) before permanent damage occurs.

Don't smoke: Smoking is one of the strongest risk factors for severe gum disease.

Manage health conditions: Keep diabetes and other inflammatory conditions well-controlled.

Know the early signs: Watch for bleeding when brushing (not normal!), persistent bad breath, receding gums, or loose teeth. See a dentist immediately if you notice these.

The Bottom Line.

Think of your family history as a warning light, not a guaranteed diagnosis.

Without family history: You might have to try hard to get severe gum disease.

With family history: You have a lower "threshold." The same amount of plaque will cause more damage in you than in someone without the genetic risk.

So, will you definitely get it? No.
Are you at higher risk? Yes.
Can you prevent it? Absolutely, with excellent hygiene and regular professional care.

Tell your dentist clearly: *"Gum disease runs in my family. I want to be proactive about prevention."* They can set up a personalized prevention plan (like more frequent cleanings) to help you beat your genetic odds.

Q9 Can stress make me more likely to get gum disease?

That's an excellent question. The short answer is yes, stress is a significant risk factor for gum disease (periodontal disease). While stress doesn't directly cause gum disease on its own, it creates the perfect storm in your body and your habits that makes it much more likely to develop and get worse.

Here's the breakdown of how stress increases your risk:

Stress Weakens Your Immune System.

This is the most direct biological link. When you're chronically stressed, your body produces high levels of hormones like cortisol. Elevated cortisol suppresses the immune system, reducing its ability to fight off the bacteria that naturally live in your mouth. This allows harmful bacteria to grow out of control, leading to inflammation, bleeding gums (gingivitis), and eventually the destruction of bone and tissue (periodontitis).

Stress Leads to "Neglect" Behaviors.

When you're overwhelmed, self-care is often the first thing to go. You might:

Brush and floss less often or with less care.

Skip dental check-ups due to lack of time or energy.

Have a poor diet, choosing sugary or processed comfort foods that feed harmful oral bacteria.

Stress Encourages Bad Habits.

Many people unconsciously adopt habits under stress that directly harm their gums:

Clenching or grinding your teeth (bruxism): This puts excessive force on the ligaments that hold your teeth in place, accelerating the bone loss already caused by gum disease.

Smoking or vaping: People often smoke more when stressed. Nicotine and other chemicals restrict blood flow to the gums, masking the early signs of infection (bleeding) while allowing the disease to progress silently.

Nail-biting or chewing on objects: These can create minor injuries in the gum tissue, making it easier for bacteria to invade.

The "Dry Mouth" Connection.

Stress activates the sympathetic nervous system ("fight or flight"), which can reduce saliva production. Saliva is your mouth's natural defense it washes away food and bacteria, neutralizes acids, and contains antimicrobial proteins. A chronically dry mouth is a much friendlier environment for the bacteria that cause gum disease.

The Scientific Evidence.

Numerous studies have confirmed this link:

The "Health Professionals Follow-Up Study" found that men who reported high levels of stress and difficulty coping were at significantly greater risk for bone loss due to periodontal disease.

Long-term studies show that caregivers for spouses with dementia (a known chronic stress model) show significantly more gum disease progression than their non-caregiver peers.

What You Can Do About It.

Knowing the link is the first step. Here's how to break the cycle:

Prioritize "Small" Oral Care: When stressed, aim for "good enough" rather than perfect. Two minutes of brushing and a quick floss is far better than nothing.

Manage the Stress Physically: Exercise, adequate sleep, and deep breathing can lower cortisol levels, boosting your immune system's ability to fight oral bacteria.

Hydrate: Drink plenty of water to combat dry mouth. Chew xylitol gum to stimulate saliva flow without feeding bacteria.

Get a Nightguard if You Clench/Grind: This is a crucial intervention to protect your gums and teeth from the mechanical destruction of bruxism.

See Your Dentist and Be Honest: Tell your dentist you're under stress. They can check for early signs of gum disease (which is often painless at first) and may recommend more frequent cleanings (e.g., every 3-4 months instead of 6).

In short: Stress doesn't put the bacteria in your mouth, but it disarms your defenses, encourages neglect, and promotes harmful habits. Managing your stress is therefore an essential part of managing your gum health.

Disclaimer: I'm an AI, not a doctor or dentist. If you're seeing blood on your toothbrush, persistent bad breath, or receding gums, please schedule a check-up.

Q10 Do gum diseases only affect the mouth and teeth?

This is an excellent question, as it gets to a common misconception.

The short answer is no, gum diseases do not only affect the mouth and teeth. While the primary damage (tooth loss, bone destruction) happens in the mouth, the inflammation and bacteria associated with gum disease have been scientifically linked to serious health problems throughout the entire body.

Here's how the effects can spread beyond the mouth:

The Core Mechanism: Inflammation.

Gum disease (especially the advanced form, periodontitis) is a chronic inflammatory condition. Think of it as a persistent, open wound in your mouth totaling about the size of the palm of your hand. This inflammation doesn't stay confined. It releases inflammatory signals (like C-reactive protein) and bacteria into your bloodstream.

Systemic Health Conditions Linked to Gum Disease.

Research has established strong associations between untreated gum disease and:

Cardiovascular Disease: The bacteria and inflammatory markers from your gums can enter your bloodstream, travel to your arteries, and contribute to the formation of atherosclerotic plaques (hardening of the arteries). This increases the risk of heart attacks and strokes.

Diabetes: This is a two-way relationship.

People with diabetes are more prone to infections, including gum disease.

Gum disease makes it harder to control blood sugar. Treating gum disease often leads to improved glycemic control in diabetics.

Adverse Pregnancy Outcomes: Pregnant women with untreated gum disease have a higher risk of preterm birth (early delivery) and low birth weight babies. The body's inflammatory response to oral bacteria is thought to trigger early labor.

Respiratory Infections: Bacteria from the mouth can be inhaled into the lungs, especially in older or immunocompromised individuals. This can lead to pneumonia, COPD exacerbations, and other respiratory diseases.

Rheumatoid Arthritis (RA): Both gum disease and RA share a common underlying mechanism: destructive, chronic inflammation. People with RA have a higher incidence of gum disease, and treating gum disease has been shown to reduce RA symptoms and disease activity.

Alzheimer's Disease (Emerging Research): *Porphyromonas gingivalis*, a key bacterium in chronic periodontitis, has been found in the brains of people with Alzheimer's. The leading theory is that the bacteria or their toxic byproducts (gingipains) can travel to the brain via nerves or the bloodstream, potentially contributing to the neural degeneration seen in Alzheimer's.

What Does *Not* Happen (Putting it in Perspective).

While the links above are serious, gum disease does not directly "infect" your heart, lungs, or joints. You won't get "heart periodontitis." Instead, gum disease acts as a chronic stressor and fuel source for inflammation that accelerates other diseases.

The Bottom Line.

Direct damage: Teeth loosen and fall out due to destruction of the jawbone holding them.

Indirect systemic damage: Increases the risk and severity of heart disease, diabetes, pregnancy complications, pneumonia, arthritis, and potentially dementia.

The take-home message: Treating gum disease is not just about saving your teeth. It is an essential component of managing your overall long-term health. Good oral hygiene (brushing, flossing, regular dental cleanings) is a powerful and inexpensive way to lower systemic inflammation.

Q11 Will a hard-bristled toothbrush clean my teeth better?

That’s a common assumption, but the short answer is no a hard-bristled toothbrush is unlikely to clean your teeth better, and it can actually damage them.

Here’s why:

Plaque is soft: The sticky film (plaque) you’re trying to remove is not firmly attached. It lifts easily with gentle, thorough brushing using soft or medium bristles. Hard bristles don’t remove plaque more effectively; they just apply unnecessary force.

Risk of gum damage: Hard bristles can erode gum tissue, causing recession. Once gums recede, they don’t grow back, leaving tooth roots exposed and vulnerable to sensitivity and decay.

Risk of enamel wear: Over time, hard bristles especially with aggressive brushing can wear down tooth enamel, leading to increased sensitivity and a higher risk of cavities.

Dentist recommendations: The American Dental Association (ADA) consistently recommends soft-bristled toothbrushes for nearly all patients. Soft bristles are flexible enough to clean along the gumline and into grooves without causing injury.

What actually cleans teeth better?

Proper technique (45-degree angle toward the gumline, small circular motions)

Brushing for a full two minutes, twice a day

Using a fluoride toothpaste

Flossing to clean between teeth

If you feel your teeth aren't getting clean enough with a soft brush, check your brushing duration and technique first switching to hard bristles will likely cause harm, not help.

Q12 Should I stop brushing my teeth for a few days if my gums are bleeding?

No, you should not stop brushing your teeth if your gums are bleeding. In fact, continuing to brush gently but thoroughly can help resolve the bleeding. Bleeding gums are often a sign of inflammation caused by plaque buildup along the gumline. Stopping brushing allows more plaque to accumulate, which can worsen the bleeding and lead to gum disease (gingivitis). Instead, try using a soft-bristled toothbrush, brushing gently at a 45-degree angle to the gums, and flossing daily. If bleeding persists beyond a week or two of good oral hygiene, see a dentist to rule out other issues.

Q13 Do I need to brush my teeth after every meal to keep my gums healthy?

That's a great question, and the answer is nuanced.

Short answer: No, you don't need to brush *after every single meal* for healthy gums. For most people, brushing twice a day (morning and before bed) combined with daily flossing is sufficient and recommended by dental professionals.

Longer answer with important details:

While brushing after every meal *sounds* ideal, it can sometimes cause more harm than good, especially for your gums.

Why twice a day is usually enough:

Plaque takes time to form: The sticky film of bacteria (plaque) that irritates your gums takes about 24-48 hours to mature enough to cause significant inflammation (gingivitis). Removing it thoroughly twice a day breaks that cycle.

Prevents over-brushing: Brushing too often or too aggressively can lead to gum recession (where gums pull away from teeth, exposing sensitive roots) and enamel wear. Your gums are delicate tissue.

The risk of brushing immediately after meals:
If you eat or drink anything acidic (citrus fruits, soda, coffee, wine, tomatoes, vinegar-based dressings), the acid temporarily softens your tooth enamel. Brushing right away can literally scrub away microscopic bits of softened enamel, damaging your teeth and potentially irritating gums. Instead, wait at least 30-60 minutes after acidic foods/drinks to brush.

So, when *would* brushing after every meal be helpful?

If you have active gum disease (periodontitis), your dentist or hygienist might recommend more frequent, gentle brushing for a period of time.

If you wear braces or other orthodontic appliances, food gets trapped very easily, so brushing after meals is often advised.

If you are prone to very rapid plaque buildup.

The better "after meal" routine for gum health:
Instead of brushing immediately, do this after eating:

Rinse your mouth vigorously with water – This helps neutralize acids and wash away loose food particles.

Chew sugar-free gum (especially with xylitol) – This stimulates saliva, your mouth's natural cleanser and acid-neutralizer.

Wait 30+ minutes, then brush if you feel you need to (or just stick to your twice-daily routine).

Never skip flossing – Brushing misses about 40% of tooth surfaces (between teeth), where gum disease starts.

The most important thing for gum health is NOT frequency of brushing it's *quality* of cleaning. One thorough, gentle 2-minute brushing with a soft-bristled brush plus flossing once a day is far better than five rushed, aggressive brushings.

Final verdict: For most people, brushing twice daily + flossing once daily = healthy gums. Brushing after every meal is unnecessary and can backfire if done incorrectly. If you're concerned about your gums (bleeding, redness, puffiness), see a dentist they may have specific advice for your unique situation.

Q14 Even if I brush my teeth regularly, do I still need to get my gums checked?

Yes, absolutely. Brushing regularly is essential, but it primarily cleans the visible surfaces of your teeth. It cannot effectively remove tartar (hardened plaque) that builds up below your gumline, nor can it fully detect early signs of gum disease like periodontitis. A dental professional uses specific tools to measure gum pockets, check for hidden inflammation, and remove deposits you can't reach at home. So, even with excellent brushing habits, professional gum checks are still necessary to prevent and treat issues that brushing alone misses.

Q15 Is brushing my teeth once a day enough to keep my mouth healthy?

This is a common question, and the short answer is: For most people, brushing once a day is not enough to keep your mouth fully healthy in the long term.

Here's a breakdown of why dental professionals almost universally recommend brushing twice a day.

Why Twice a Day is the Gold Standard.

Plaque Regrows Quickly: The sticky film of bacteria (plaque) constantly forms on your teeth. Within 24 hours, it can start to harden into tartar (calculus), which you cannot remove with a toothbrush at home and requires a professional cleaning.

Nighttime is High-Risk: While you sleep, your mouth produces less saliva. Saliva is your mouth's natural defense it washes away food particles and neutralizes acids. Brushing right before bed removes the day's buildup of plaque and food, so bacteria don't have a "free feast" all night long.

Acid Attacks After Every Meal: When you eat or drink anything sugary or starchy, bacteria in your mouth produce acid that attacks tooth enamel for about 20-30 minutes. Brushing twice a day helps disrupt and remove bacteria before they cause significant damage.

What Happens if You Brush Once a Day?

It’s not that you’ll immediately get cavities, but your risk increases significantly.

Increased Risk of Cavities: The areas between teeth and near the gum line, where bacteria accumulate, are more likely to develop decay.

Gum Disease (Gingivitis): The most common early sign is bleeding gums when you brush. Once-a-day brushing often allows enough plaque to build up to cause gum inflammation. This is reversible with better hygiene, but if ignored, it can progress to periodontitis (bone loss).

Bad Breath: Persistent morning breath or bad breath during the day is often due to bacteria and food debris left overnight.

Is There *Any* Scenario Where Once a Day is Enough?

Only very rarely, and usually not for a typical adult. A dentist might accept once-daily brushing for a person with:

An exceptionally low-sugar diet (no snacks, no soda, no sweets).

Very high saliva flow (natural mouth cleaner).

Perfect brushing technique (using a soft brush for a full 2 minutes, reaching every surface).

No history of cavities or gum disease.

Even then, most dentists would still recommend twice daily as cheap, easy insurance.

The Non-Negotiable "Once a Day" Partner.

If you absolutely cannot brush twice a day, there is one thing you must do daily without fail: Flossing (or using interdental brushes). Flossing removes plaque and food from *between* teeth where your brush never reaches. Some studies suggest flossing once a day may be as important as brushing for preventing gum disease.

The Bottom Line for Real Health.

Bare minimum to avoid immediate disaster: Brush thoroughly once a day (at night) AND floss once a day.

Recommended for long-term health & fresh breath: Brush twice a day (morning and night), floss once a day.

The ideal routine: Brush twice a day with fluoride toothpaste, floss once a day, and see your dentist for a cleaning every 6-12 months.

Recommendation: Try it as an experiment. Brush only once a day for a week, and pay attention to how your teeth feel in the morning (fuzzy? coated?) and if your gums bleed when you floss. Then switch to twice a day for a week. The difference in how clean your mouth feels is usually dramatic. That cleanliness is what prevents disease.

Q16 Can flossing cause gum recession?

This is an excellent question, as it gets at a common point of confusion between correlation (flossing is often seen in people with receding gums) and causation (flossing actively making the gums recede).

The short answer is: Proper, gentle flossing does NOT cause gum recession. However, aggressive or incorrect flossing CAN cause gum recession.

Let's break down why.

How Incorrect Flossing Causes Recession (The "Bad" Way).

Think of your gums as a tight, high-neck sweater. Aggressively forcing a thick rope (the floss) down and snapping it against the neck will stretch and fray the fibers. Similarly, bad flossing technique physically damages the gum tissue.

Here are the specific harmful techniques:

"Snapping" the floss: Forcing the floss straight down between teeth with a snapping motion. This traumatizes the triangular piece of gum (the papilla) and the attachment point where the gum meets the tooth.

Sawing motion: Rubbing the floss back and forth against the gum line like a saw blade. Over time, this can wear down the gum tissue and the root surface below it.

Flossing too deep/aggressively: Shoving the floss well past the point of gentle resistance, tearing the ligament that attaches the gum to the tooth. This creates a "false pocket" that can deepen, leading to the gum pulling away permanently.

The result of these habits: Over months or years, the cumulative trauma causes the gum margin to pull back (recede), exposing the sensitive, yellowish root of the tooth.

Why People *Think* Flossing Causes Recession (Correlation).

This is the most common reason for the myth. Gum disease (periodontitis) is the 1 cause of gum recession, not flossing.

The Sequence: Plaque builds up Gums get inflamed (gingivitis) Inflammation spreads below the gum line The bone and ligament holding the tooth are destroyed The gum recedes.

The Role of Floss: When a person finally notices their receding gums and begins to floss properly for the first time, they mistakenly blame the new action (flossing) for the existing problem (recession).

The "Bad Flossing Disease" Link: People who floss aggressively often do a poor job of removing plaque, because they are focused on the snap/saw action. The remaining plaque triggers the gum disease that actually causes the recession.

How Proper Flossing Prevents Recession (The "Good" Way).

Proper flossing is one of the best things you can do to prevent gum recession.

It removes the sticky bacterial plaque that causes gingivitis and periodontitis, the primary drivers of recession.

Healthy, clean gums fit snugly around the teeth. Inflamed gums are puffy, weak, and more prone to pulling away.

The Crucial Difference: Technique.

Feature.	Aggressive / Incorrect Flossing (Causes Recession).	Gentle / Correct Flossing (Prevents Recession).
Action.	Snapping down, sawing side-to-side.	Sliding down gently, curving into a "C" shape.

Feature.	Aggressive / Incorrect Flossing (Causes Recession).	Gentle / Correct Flossing (Prevents Recession).
Depth.	Forces floss deep into the gum, causing pain/bleeding.	Goes just below the gum line (2-3mm) until resistance is felt.
Pressure.	High pressure against the gum triangle.	Gentle pressure, hugging the side of the tooth.
Result.	Tears gum fibers, wears down tissue.	Massages gums, removes plaque, cleans the pocket.

How to Floss Correctly (To Protect Your Gums).

Follow this "C-Shape" method to ensure you are helping, not harming, your gums:

Use about 18 inches of floss, winding most around your middle fingers.

Hold it taut with your thumbs and index fingers.

Gently slide the floss between your teeth using a zig-zag motion. Do not snap it down.

Curve the floss into a "C" shape against one tooth.

Slide it gently under the gum line until you feel resistance.

Hug the tooth and scrape the floss *up and down* against the side of the tooth (not sawing into the gum). Repeat for the adjacent tooth.

Unwind to a fresh section of floss for each new gap.

When to See Your Dentist.

If your gums bleed regularly when you floss: This is a sign of active gum inflammation (gingivitis), not that you are flossing "too hard." Keep flossing gently, and the bleeding should stop within 2 weeks. If not, see your dentist.

If you already have signs of recession (notched teeth, sensitivity): Switch to an ultra-soft, "satin" floss or tape, which is gentler than a round, waxed floss. Ask your dentist if you need a deep cleaning or gum graft.

If you find it impossible to floss without snapping: Try alternatives like interdental brushes or a water flosser (use on a low-medium setting aimed at the gaps, not directly into the gum pocket).

Bottom line: Floss daily, but be gentle. The goal is to hug the tooth, not to hack the gum. If in doubt, ask your dentist or hygienist to watch you floss at your next cleaning they can correct your technique in seconds.

Q17 Do electric toothbrushes damage the gums more than regular toothbrushes?

This is a common concern, but the short answer is: No, electric toothbrushes do not inherently damage gums more than manual toothbrushes. In fact, when used correctly, they often lead to *healthier* gums. However, improper use of either type can cause gum damage.

Here’s the detailed breakdown:

When Damage Happens (With Either Brush).

Gum damage (recession, bleeding, abrasion) is almost always caused by technique, not the tool itself. The primary culprit is brushing too hard.

With a manual brush: People often saw back-and-forth aggressively, pressing hard. This can cut the gums and wear away enamel.

With an electric brush: Some people press as hard as they did with a manual brush. This can be worse because the electric brush's rapid oscillations add extra friction to that excessive pressure.

Key Differences That Affect Gums.

Electric Toothbrushes (specifically sonic or rotating-oscillating).

Built-in pressure sensors: Many modern electric brushes (Oral-B, Sonicare) beep or stop pulsing when you push too hard, preventing damage.

Require less force: You only need to guide the brush, not scrub. The movement does the work.

Clinical evidence: Multiple studies (e.g., in the *Journal of Clinical Periodontology*) show that electric brushes reduce gum inflammation (gingivitis) and bleeding more effectively than manual brushes *without* increasing recession when used as directed.

Manual Toothbrushes.

No pressure feedback: It's easy to press too hard without realizing it.

Variable technique: People often use harsh scrubbing motions instead of gentle circles.

What Actually Damages Gums?

The following habits hurt gums regardless of brush type:

Hard or medium bristles Always use *soft* or *extra-soft* bristles.

Excessive pressure You should feel the bristles tickle the gumline, not press firmly.

Horizontal scrubbing This causes V-shaped notches in teeth and gum recession.

Using a worn brush Frayed bristles become abrasive.

The Verdict.

Scenario.	Gum Damage Risk.
Correct use of electric brush (soft bristles, light pressure, guided along gumline).	Very low and reduces bleeding/gum disease.
Correct use of manual brush (soft bristles, gentle circles, 2 minutes).	Low.
Incorrect use of electric brush (pressing hard, medium bristles).	High but the brush will often warn you.

Scenario.	Gum Damage Risk.
Incorrect use of manual brush (hard scrubbing, firm pressure).	High no warning system.

Practical Takeaway.

If you already have gum recession or sensitivity, an electric brush with a pressure sensor is actually safer than a manual brush because it stops you from pushing too hard. The key steps:

Choose a soft-bristled electric brush head.

Use no more pressure than the weight of the brush itself.

Hold it at a 45-degree angle to the gumline and glide slowly.

Let the brush do the oscillating don’t scrub.

If you already use a manual brush with perfect light pressure and soft bristles, you may not need to switch. But if you tend to scrub aggressively, an electric brush with a pressure sensor will likely *reduce* your risk of gum damage.

Q18 Would using mouthwash alone be enough without brushing my teeth?

This is a great question, and the short, direct answer is no. Relying on mouthwash alone is not enough for good oral health.

Here’s a simple breakdown of why brushing is essential and what mouthwash can and cannot do.

Why Brushing is Non-Negotiable.

Think of brushing as the physical cleaning of your teeth. Its main job is mechanical removal.

Removing Plaque: Plaque is a sticky, colorless film of bacteria that constantly forms on your teeth. It's the root cause of cavities and gum disease. The only way to effectively break up and remove this sticky biofilm is with the gentle scrubbing action of a toothbrush bristles. Mouthwash cannot dissolve or lift off plaque.

Clearing Food Debris: While mouthwash can rinse away loose food particles, it can't dislodge the bits of food that get stuck between teeth or in the grooves of your chewing surfaces. These trapped pieces feed the bacteria that cause plaque.

Fluoride Application: This is the most important part of brushing. Fluoride toothpaste doesn't just clean; it strengthens your tooth enamel, making it more resistant to acid attacks from bacteria. You get a concentrated, targeted application of fluoride while brushing. A quick rinse with a fluoride mouthwash is much less effective.

What Mouthwash *Is* Good For.

Mouthwash is a supportive tool, not a replacement. Its main jobs are:

Reducing Bacteria (Temporarily): Antiseptic mouthwashes (like Listerine) kill bacteria in your mouth. However, they can't penetrate the plaque biofilm. They only kill bacteria on the surface, which return within a few hours. Brushing physically removes the entire biofilm colony.

Freshening Breath: Mouthwash is excellent for temporarily masking bad breath (halitosis). However, if the bad breath is caused by the smelly byproducts of bacteria living in the plaque you haven't brushed off, the mouthwash is just a temporary cover-up.

Reaching Hard-to-Brush Areas: A good rinse can help deliver anti-cavity (fluoride) or anti-gingivitis ingredients to areas you might have missed, like the back of your molars or along the gumline.

Therapeutic Benefits: Some specialized mouthwashes can help with specific conditions (like dry mouth or high cavity risk), but they are prescribed *in addition to* a good brushing and flossing routine.

The Unpleasant Analogy (It Works, Though).

Imagine your kitchen floor after you've spilled a thick, sticky smoothie.

Brushing is using a mop and scrub brush. You physically lift and remove the sticky residue (plaque) from the floor (your teeth). This cleans the surface.

Mouthwash alone is like splashing a bucket of water across the floor. You might thin out the sticky smoothie and wash away some of the loose bits, but the main sticky residue will still be there, dried onto the tiles.

The Verdict: The "Gold Standard" Routine.

For a truly clean and healthy mouth, you need the complete trio:

Brush: Twice a day for two minutes with a soft-bristled toothbrush and fluoride toothpaste. This cleans the front, back, and top surfaces of your teeth.

Floss: Once a day. This cleans the 40% of your tooth surfaces that your brush can't reach (the tight spaces between teeth).

Mouthwash (optional but helpful): Use it at a different time than brushing (e.g., after lunch or at a non-brushing time). If you use it right after brushing, you'll rinse away the concentrated fluoride from your toothpaste, making it less effective. If you do use it after brushing, choose a fluoride mouthwash and wait 30 minutes before eating or drinking.

Conclusion: If you skip brushing and just use mouthwash, you are not removing plaque. Over time, that plaque will harden into tartar (calculus), which can only be removed by a dentist or hygienist. This will lead to cavities, gum inflammation (gingivitis), and eventually, gum disease.

So, please keep brushing! Mouthwash is a great teammate for your toothbrush, but a terrible substitute.

Q19 After I quit smoking, my gums started bleeding. Is that normal?

It is actually quite common for gums to bleed after quitting smoking, and here’s why: smoking constricts blood vessels in your gums, which reduces blood flow and masks signs of underlying gum disease (like redness, swelling, or bleeding). When you stop smoking, circulation improves, and your immune system begins working more actively to fight bacteria in your mouth. This can lead to temporary gum inflammation and bleeding a phenomenon sometimes called “quitter’s gums.”

That said, while the bleeding itself is a normal reaction to improved circulation, it is also a sign that you likely have existing gingivitis or periodontitis that was suppressed by smoking. The good news is that this usually resolves within a few weeks to months with good oral hygiene: gentle brushing with a soft toothbrush, daily flossing, and using an antiseptic mouthwash. However, it’s wise to see a dentist for a thorough exam and professional cleaning, as they can help manage the inflammation and rule out more serious gum problems. If bleeding is heavy, persistent beyond a few months, or accompanied by pain or loose teeth, seek dental advice promptly.

Q20 Can natural products effectively keep my gums healthy?

This is an excellent question, as gum health is the foundation of a healthy mouth.

The short answer is yes, certain natural products can be effective aids in keeping your gums healthy, but they work best as a supplement to, not a replacement for, traditional oral hygiene. For treating active gum disease (like periodontitis), you must see a dentist.

Here is a practical, evidence-informed breakdown of what works, what might help, and what to watch out for.

The Gold Standard (Non-Negotiable).

Before using any natural product, you must have a solid routine. No natural product can effectively remove the sticky biofilm called plaque like mechanical cleaning.

Brushing (twice daily) with a soft-bristled brush.

Flossing (once daily). This is the single most important step for gum health.

Natural Products with Real Evidence.

These have the strongest scientific backing for reducing *gingivitis* (the early, reversible stage of gum disease, marked by redness and bleeding).

Salt Water Rinses (The Most Effective Natural Aid).

Why it works: Creates a hypertonic solution that draws fluid out of swollen, inflamed gums, reducing puffiness. It also temporarily raises the pH in your mouth, making it harder for bacteria to thrive.

How to use: Dissolve 1/2 teaspoon of salt in a cup of warm water. Swish for 30 seconds, then spit. Use 2-3 times per day, especially after flossing.

Best for: Reducing inflammation and soothing irritated gums. Do not use long-term (months), as it can eventually erode enamel.

2. Oil Pulling (Coconut Oil).

Why it works: The lauric acid in coconut oil has mild anti-inflammatory and antimicrobial properties. Swishing mechanically helps dislodge some bacteria. Studies show it can reduce plaque and gingivitis scores comparable to some mouthwashes.

How to use: Take 1 tablespoon of coconut oil (solid or liquid) and swish it around your mouth for 10-15 minutes first thing in the morning. Spit it into a trash can (not the sink, as it can clog pipes), then brush your teeth.

Best for: A gentle, daily anti-inflammatory practice. It is not a replacement for flossing.

3. Green Tea.

Why it works: Rich in antioxidants called catechins. Drinking unsweetened green tea has been shown in multiple studies to reduce periodontal pocket depth and bleeding on probing.

How to use: Drink 1-2 cups of plain, hot-brewed green tea daily. Do not add sugar or honey.

Best for: Systemic support for gum health. The effect is from drinking it, not using the leaves as a paste.

Natural Products with Weak or Mixed Evidence (Use Caution).

Tea Tree Oil.

Effectiveness: Very antimicrobial.

The problem: Highly irritating to oral tissues if not diluted properly. Never use pure tea tree oil in your mouth. Some toothpaste and mouthwash contain very low, safe concentrations.

Verdict: Skip the DIY version. If you want to try it, buy a commercially prepared, diluted oral care product.

Aloe Vera.

Effectiveness: Good for soothing inflammation.

The problem: Most studies show it works no better than a standard fluoride toothpaste. The gel must be pure, internal-use grade (free of aloin, a laxative compound).

Verdict: It's safe, but don't expect miracles. It may help with post-dental procedure healing.

3. Baking Soda.

Effectiveness: Excellent at neutralizing acid and mildly abrasive for plaque removal.

The problem: Can be too abrasive for gums if used as a dry powder. It also has no antibacterial properties.

Verdict: A toothpaste with baking soda is fine. Scrubbing your gums with baking soda paste is not.

Natural Products to AVOID for Gums.

Lemon juice, apple cider vinegar (undiluted), or any pure acid: These will erode your enamel instantly. Your gums may feel "clean" because the acid has denatured tissue, but you are damaging your teeth.

Hydrogen peroxide (full-strength): Can damage gum tissue and the surface of your tongue. Even diluted, it's harsh for routine use.

Activated charcoal: Gritty and abrasive. It can wear down enamel and gums over time.

The Most Important Thing to Understand.

Healthy gums do not bleed. If your gums bleed when you brush or floss, that is the first sign of infection (gingivitis).

What natural products can do: Reduce mild inflammation and supplement your hygiene to keep healthy gums healthy.

What natural products cannot do: Remove hardened plaque (tartar/calculus). Once plaque calcifies onto your teeth, no natural product, herbal rinse, or oil can remove it. Only a professional dental cleaning (scaling) can.

A Practical Routine for Gum Health.

Morning: Oil pull (coconut oil) for 10 min while you shower. Spit, then brush with fluoride toothpaste.

Night: Floss correctly. Brush for 2 full minutes. Rinse with warm salt water.

Throughout the day: Drink plain water and unsweetened green tea.

Every 6 months: See your dentist for a professional cleaning and check-up.

Bottom line: Use salt water and coconut oil pulling as safe, effective supplements. But never let them delay professional care for bleeding or painful gums.

Q21 Can getting a dental scaling damage my teeth?

This is a great question, and a common concern. The short answer is: No, a professional dental scaling (cleaning) performed by a qualified hygienist or dentist does not damage your teeth. In fact, it's one of the best things you can do to protect them.

However, there are a few sensations *after* a cleaning that can *feel* like damage. Let's break down why that happens.

Why Scaling Feels Like It Might Be Damaging, But Isn't.

People often confuse the *effects of removing buildup* with *damage to the tooth itself.*

Sensitivity to Cold/Air: After a deep cleaning, your teeth may feel more sensitive to cold drinks or air. This isn't damage. Before the cleaning, tartar (hardened plaque) covered the tooth surface, acting like an insulating blanket. Once that blanket is removed, the natural pores in your tooth (dentinal tubules) are exposed to the world for the first time in a while. This sensitivity is temporary (days to a week).

"Rough" or "Grooved" Feelings: You might run your tongue along a tooth and feel a groove or a rough spot that wasn't there before. This is almost always a pre-existing defect that was previously filled with tartar. The scaling removed the tartar, revealing the natural (or damaged) surface underneath. The scaling didn't create the groove; it just uncovered it.

Gums Appear to Recede: After a thorough cleaning, especially if you had significant tartar buildup below the gumline, your gums may look like they've shrunk or receded. This is actually healing. The tartar was pushing your gums away (causing inflammation and "false pockets"). Removing the tartar allows the gums to re-attach firmly to the clean tooth root at a healthier level. The scaling didn't cut your gums; it allowed the inflammation to resolve.

Can it *Ever* Cause Harm? (Rare Avoidable Cases).

In the hands of an inexperienced or careless clinician, minor issues are theoretically possible, but very rare with a trained professional.

Tooth Enamel: Enamel is incredibly hard (harder than bone). The fine ultrasonic scalers and hand curettes used by professionals are designed to remove much softer tartar without damaging enamel. Under a microscope, you might see very fine scratches, but these are no deeper than natural tooth wear and are smoothed over by the polishing step that follows scaling.

Dental Restorations (Fillings, Crowns, Veneers): There is a small risk of chipping the edge of an older, weak filling or dislodging a poorly bonded crown. A skilled hygienist will adjust their technique around restorations. This is a risk of the *procedure*, not intentional damage.

The Much Bigger Danger: *Not* Getting Your Teeth Scaled.

The real damage comes from avoiding cleanings. The buildup of plaque and tartar leads to:

Gum Disease (Periodontitis): This destroys the bone that holds your teeth in place. Bone does not grow back. This is the 1 cause of tooth loss in adults.

Tooth Decay: Plaque under the gumline eats away at tooth roots, causing cavities that can lead to root canals or extractions.

Bad breath and bleeding gums.

A Note on "Deep Cleaning" (Scaling Root Planing).

If your dentist recommends a "deep cleaning" (for active gum disease), there can be a temporary side effect: some increased tooth mobility. This sounds scary, but it's a normal part of healing. Before the cleaning, the tooth was held in by tartar "cement" and inflamed gums. Removing the tartar loosens the

tooth *temporarily* (weeks to months) while the gums heal and re-tighten around it. Not removing the tartar guarantees the tooth will eventually loosen and fall out.

The Bottom Line.

Can dental scaling damage my teeth?.	No.
What you might feel:	Temporary sensitivity, minor gum soreness, discovering pre-existing chips/grooves.
The real risk:	Gum disease and tooth loss from *avoiding* scaling.
Recommendation:	Get regular professional cleanings (usually every 6 months). If you have concerns about sensitivity or technique, talk to your dentist. They can use topical anesthetic and gentle settings.

In short: The proven, overwhelming benefit of removing disease-causing bacteria and tartar far, far outweighs any negligible, temporary, or reversible sensation you might feel. Professional dental scaling saves teeth; it doesn't damage them.

Q22 Can dental scaling cause gaps between my teeth?

This is a great question, and it’s a very common concern. Here’s the direct answer, followed by a clear explanation of what’s actually happening.

The short answer is no, dental scaling does not *create* new gaps or spaces between your teeth.

However, it can *reveal* spaces that were already there but were previously hidden by tartar (calculus) and inflammation.

Let's break down why this happens.

The Real Cause: Tartar Masked the Existing Gaps.

Think of dental calculus (hardened plaque) as a cement-like substance that builds up on and between your teeth. As gum disease (periodontitis) progresses, your gums become inflamed and pull away from your teeth, creating small pockets. Tartar can fill these pockets and bridge across teeth.

Here’s what happens during and after a deep cleaning (scaling and root planing):

Before Scaling: You have a buildup of tartar between your teeth. This tartar is physically taking up space. Your gums are likely swollen, red, and puffy due to the bacteria in the tartar. This swelling can make the spaces between teeth feel tighter and look fuller.

During Scaling: The dentist or hygienist removes the tartar from between your teeth. They also remove it from below the gum line, where the disease process is active.

After Scaling (The "Revelation"):

The tartar is gone. The physical "filler" that was occupying the space between your teeth is no longer there. You will now feel the true, natural contour of your teeth. If you had minor natural spacing or bone loss, this will now be noticeable.

The gum swelling goes down. Over the next few days and weeks, the inflammation subsides. The puffy, swollen gums heal and shrink back down to a healthy, firm state around the cleaner tooth surface. As the gums shrink, they no longer fill the spaces between the teeth as they did when swollen.

The result is that spaces that were always present (due to bone loss from gum disease) are now visible and feelable with your tongue. The scaling didn't cut or create the space; it just removed the disease that was hiding it.

The Important Distinction: Healthy Spaces vs. Unhealthy Spaces.

	Normal, Healthy Teeth.	Teeth After Gum Disease Treatment.
What's between teeth?	Healthy, pink, firm gum tissue (papilla) that fills the space.	Shrunken, healed gums that no longer fill the space due to past bone loss.
Will scaling create a gap?	No. In a healthy mouth, removing a little plaque and tartar will not create a gap. The gum will stay in place.	No, but a gap may appear. The gap was already there, hidden by tartar and swollen gums.
What you feel after cleaning	Smooth, tight contact between teeth.	A "black triangle" or open space where the gum used to be (or where tartar was).

What You Should Do.

Do not avoid dental cleanings. The worst thing you can do is stop getting your teeth cleaned because you're afraid of the spaces. The tartar that you let build up will only worsen the bone loss, leading to larger gaps and eventual tooth loss.

Understand the goal. The goal of scaling, especially deep cleaning for gum disease, is to save your teeth and the bone that supports them. A little visible space is a small price to pay for keeping your natural teeth for life.

Consider treatment for the gaps (if desired). Once your gums are healthy (usually a few months after scaling), a cosmetic dentist can discuss options like:

Bioclear / Injection-molded composite bonding: A fantastic, minimally invasive way to fill "black triangles."

Orthodontics (braces or clear aligners): Can sometimes close gaps.

Porcelain veneers: For reshaping teeth to close spaces.

The bottom line: Scaling doesn't create gaps. Untreated gum disease causes bone loss, which creates gaps. Scaling simply removes the tartar that was hiding the damage from you. Seeing a small gap after a cleaning is actually a sign that the treatment is working and your gums are healing.

Q23 Do all gum diseases require the same treatment?

No, not all gum diseases require the same treatment. Gum disease ranges from mild gingivitis to severe periodontitis, and treatment depends on the stage, severity, and individual patient factors.

Gingivitis (reversible, gums red/swollen but no bone loss) is typically treated with professional dental cleaning, improved oral hygiene (brushing, flossing, antiseptic mouthwash), and addressing contributing factors like smoking or poor nutrition.

Periodontitis (chronic, with bone loss and pocket formation) may require scaling and root planing (deep cleaning), locally applied antibiotics, or even surgical interventions like flap surgery, bone grafting, or guided tissue regeneration.

Aggressive or necrotizing periodontitis may need systemic antibiotics, laser therapy, or more intensive surgical approaches.

Thus, while prevention and basic hygiene are common to all, treatment is tailored to disease type and severity. Always consult a dentist for an accurate diagnosis and personalized treatment plan.

Q24 Is gum treatment painful?

This is a common concern, and the answer depends on the type of gum treatment and your individual pain tolerance.

In short: Modern gum treatments are not typically painful during the procedure, but some mild discomfort afterward is common.

Here’s a breakdown by treatment type:

Routine Cleaning (Prophylaxis) For Healthy Gums.

Pain level: None to very mild.

What to expect: You might feel scraping or vibration, but it shouldn't be painful if your gums are healthy. If you have sensitive teeth, cold water rinses might cause brief twinges.

Scaling and Root Planing (Deep Cleaning) For Gum Disease.

This is the most common treatment people ask about.

During the procedure: Not painful. Dentists almost always use a local anesthetic (numbing shots) to block pain. You'll feel pressure, scraping, and vibration, but not sharp pain.

After the procedure (24 72 hours): Mild to moderate discomfort. Your gums will likely feel:

Sore, tender, or bruised.

Slightly swollen.

Sensitive to hot, cold, or pressure.

This is often described as similar to the soreness after a hard workout. Over-the-counter pain relievers (like ibuprofen) and a soft-food diet manage this very well.

Gum Surgery (e.g., Pocket Reduction, Grafts) For Advanced Disease.

During the procedure: Not painful. Performed under strong local anesthesia (often with sedation options).

After the procedure (several days to a week): Moderate discomfort. This is more significant than after deep cleaning. You can expect throbbing, swelling, and pain managed with prescription painkillers. Most patients say the worst pain is in the first 48 hours and then improves quickly.

Laser Gum Treatment.

During the procedure: Less uncomfortable than traditional methods. Lasers are precise, cauterize as they cut (less bleeding), and often require less anesthesia.

After the procedure: Milder and shorter discomfort than with scalpels and sutures. Healing tends to be faster.

What Actually Causes Pain?

Not the cleaning itself (if you're numb).

The inflammation: Your gums are already sore from bacteria. Poking them, even gently, aggravates that existing inflammation.

The post-procedure healing: Your body's natural inflammatory response causes the soreness after the numbing wears off.

How to Minimize Discomfort.

Before Treatment.	During Treatment.	After Treatment.
Tell your dentist if you have anxiety – they can use numbing gel before shots or offer sedation.	Ask for more anesthetic if you feel sharp pain (not just pressure).	Take ibuprofen or acetaminophen as directed before the numbing wears off.
Use a sensitive toothpaste for 2 weeks prior to deep cleaning.	Use headphones/music to distract yourself.	Rinse with warm salt water (starting 24 hours later).
		Eat soft, cool foods (yogurt, smoothies, scrambled eggs).
		Avoid hot, spicy, crunchy, or chewy foods for a few days.

Key Takeaway.

The fear of pain is almost always worse than the reality. Most patients are surprised at how manageable it is. The real pain to worry about is avoiding treatment untreated gum disease leads to bone loss, loose teeth, and abscesses, which are genuinely painful.

If you have a specific procedure scheduled, ask your dentist for their exact post-op pain plan. They want you to be comfortable.

Q25 Can gum surgery cause tooth loss?

This is a great question, and it gets at a common fear people have about dental procedures. Here's the direct answer, followed by a more detailed explanation.

The short answer is: No, gum surgery performed correctly by a qualified periodontist or dentist does not cause tooth loss. In fact, its primary goal is to *prevent* tooth loss caused by severe gum disease.

However, there is a very important nuance: If a tooth is already so severely damaged by advanced gum disease that it has little to no bone support left, that tooth may be deemed "hopeless" and be extracted *during* or *prior to* the surgery. In this case, the surgery itself isn't the cause; the underlying disease is. The surgery simply reveals the true extent of the damage.

Let's break this down.

The Main Goal of Gum Surgery: Save Teeth.

Gum (periodontal) disease is an infection that destroys the bone and ligaments holding your teeth in place. As the disease progresses, teeth become loose. Gum surgery (like flap surgery or osseous surgery) is performed to:

Remove deep tartar and bacteria from under the gums that cannot be reached by regular cleaning.

Reshape damaged bone to eliminate pockets where bacteria hide.

Allow the gums to reattach more firmly around the teeth.

By stopping the bone loss, the surgery *strengthens* the long-term foundation of your teeth, preventing the eventual loss that would happen without treatment.

So Why the Confusion? When Might a Tooth Be Lost *Near* the Time of Surgery?

There are three specific scenarios where a tooth might be lost in the context of gum surgery, but the surgery itself isn't the cause:

The Tooth Was Already "Hopeless" (Most Common Scenario).

What happens: Severe gum disease has destroyed more than 50-75% of the bone around a tooth. It's already loose and has a poor long-term prognosis.

Surgery's role: A responsible surgeon won't waste time and money trying to save a tooth with zero chance of success. They will recommend extracting it *during* the gum surgery. This is considered a good outcome, as it removes a source of chronic infection.

Key takeaway: The disease caused the loss; surgery just provided the occasion for a necessary extraction.

Pre-Existing Severe Risk Factors (Patient-Specific).

Heavy Smokers: Smoking severely impairs healing after any surgery. If a patient with advanced disease and heavy smoking habits undergoes surgery, the gums may not heal properly, leading to reinfection and potential loss of a marginally stable tooth.

Uncontrolled Diabetes: High blood sugar prevents proper healing and infection control, increasing the risk of failure.

Poor Oral Hygiene: If a patient doesn't brush or floss after surgery, the disease will return aggressively and could finish destroying weakened support.

Extremely Rare Surgical Complications (Mistakes).

In the hands of an unskilled or careless surgeon, it's theoretically possible to damage a healthy tooth root or remove too much bone. But this is very rare with a qualified periodontist. Standard surgery is precise and aims to preserve every millimeter of bone.

What the Science & Guidelines Say.

Major dental associations (like the American Academy of Periodontology) are clear: The natural history of untreated moderate-to-severe gum disease is eventual tooth loss. Periodontal surgery is one of the most effective evidence-based treatments to prevent that outcome.

To Put Your Mind at Ease: What to Ask Your Dentist.

If your dentist has recommended gum surgery, ask these two questions to understand your specific risk:

"Can you show me my bone loss on the X-ray? Do any of my teeth have a hopeless prognosis?" (This tells you if extraction is already on the table, regardless of surgery).

"What is my specific risk of treatment failure based on my health (smoking, diabetes, etc.)?"

Bottom Line.

Healthy or moderately diseased teeth? Gum surgery will *protect* them from future loss. No risk of surgery causing loss.

Severely diseased, already-loose teeth? The disease has already doomed them. Surgery may simply be the time when a planned extraction happens as part of the treatment plan.

Don't let fear of losing teeth stop you from getting necessary gum surgery. Avoiding surgery for advanced gum disease is virtually a guarantee of eventual tooth loss. Getting surgery is the best way to keep your natural teeth for years to come.

Q26 Can gum treatment make my teeth feel loose?

This is a great question, and it touches on a common concern. Here's the direct answer, followed by a clear explanation.

Short answer: Yes, gum treatment can *temporarily* make your teeth feel slightly loose, but this is usually a sign that the treatment is working to save your teeth, not harm them. In the long run, gum treatment prevents the much more serious loosening and loss of teeth caused by advanced gum disease.

Let's break down why this happens.

The Main Reason: Removing Tartar (Calculus).

When you have gum disease (periodontitis), a hard, cement-like deposit called tartar or calculus builds up on your tooth roots, below the gum line.

Think of this tartar as a "crust" that splints loose teeth together. It fills the spaces between the tooth and the bone, making the teeth feel artificially firm and stable, even though the bone underneath is being destroyed.

What happens during a deep cleaning (scaling and root planing):
Your dentist or hygienist carefully scrapes that tartar away.

Before treatment (with disease): Tartar is splinting teeth. Bone is *already* gone (lost). Teeth feel "fine."

Immediately after treatment: The tartar splint is removed. The teeth are now sitting in their true, bone-deficient state. This new, honest looseness is what you feel. It's not that the treatment loosened them; the treatment just *revealed* the looseness that was already there from the disease.

What Else Contributes to the Loose Feeling?

Inflammation & Healing: After a deep clean, your gums are swollen and tender as part of the healing process (the body sending in repair cells). This swelling can change the bite slightly or make the support feel less solid. This subsides in 1-2 weeks.

Polishing: Aggressive polishing is rare in deep cleanings, but if done, it could temporarily disturb the gum attachment.

Will the Looseness Go Away?

Yes, usually significantly. This is the most important part.

Over 2-4 weeks: As the gums heal, they reattach *tightly* to the clean root surface. The swelling goes down. The initial loose feeling often diminishes greatly.

Over 3-12 months: The ultimate goal is to get your gums healthy. Healthy gums are like a tight, elastic cuff around the tooth. While the lost bone won't grow back on its own, the improved gum attachment and reduced inflammation will stabilize the tooth. In many cases, the teeth become firmer than they were before treatment, once the disease process is stopped.

When Should You Be Concerned?

Extreme looseness: If a tooth feels like it's moving more than 1mm in any direction, or you can push it with your tongue, tell your dentist. This suggests very advanced bone loss.

Worsening looseness: The feeling should improve, not get worse, after the first week or two.

New looseness in a previously firm tooth that wasn't treated.

The Crucial Bottom Line.

Do not avoid gum treatment because you're afraid of loose teeth.

If you have gum disease and DON'T treat it: The tartar will continue to destroy the bone. Teeth will eventually become *permanently* and irreversibly loose, leading to tooth loss. The "false" stability from the tartar only lasts a few years before the teeth fall out.

If you DO treat it: You may feel a *temporary* increase in awareness of looseness, but you stop the bone loss and give your teeth the best chance to tighten up and last a lifetime.

What you should do right now:
Call your dentist. Tell them, *"I'm concerned about my teeth feeling loose after treatment. Can we discuss my specific bone levels and what to expect?"* They can show you your x-rays to explain exactly how much support your teeth have.

You are asking the right questions. Understanding this process is key to successfully treating gum disease without unnecessary fear.

Q27 Does tartar removal cause more tartar build up?

No, tartar removal does not cause more tartar buildup. In fact, professional dental cleaning (scaling) removes existing tartar, which helps prevent future buildup. Tartar forms when plaque a sticky bacterial film is not removed by brushing and flossing, and then hardens on the teeth. After a professional cleaning, the tooth surfaces are smoother, making it easier for you to keep them clean through regular oral hygiene. Without removal, tartar only continues to accumulate. Some people mistakenly believe tartar returns faster after cleaning, but this is usually due to inadequate home care or the natural progression of plaque mineralization, not the cleaning itself.

Q28 Are antibiotics enough to treat gum disease?

This is an excellent question, and the short answer is no, antibiotics alone are almost never enough to treat gum disease (periodontal disease).

While antibiotics are a valuable tool, they are ineffective as a standalone treatment. Here's a detailed breakdown of why.

The Root Cause: Plaque and Tartar.

Gum disease is primarily caused by a buildup of dental plaque – a sticky film of bacteria. When plaque is not removed, it hardens into tartar (calculus).

Plaque is a soft biofilm that can be disrupted by brushing and flossing.

Tartar is rock-hard and bonds firmly to your tooth roots. Antibiotics cannot penetrate tartar. The bacteria living within the tartar are protected and will continue to cause infection and bone destruction.

Why Antibiotics Fail Alone.

Imagine tartar as a walled fortress, and the bacteria are the enemy soldiers inside. Taking an antibiotic is like spraying a hose over the fortress walls. The soldiers inside remain safe and will just rebuild their army once you stop the water.

Specifically:

Physical Barrier: The dense structure of tartar prevents antibiotics from reaching the bacteria deep within the periodontal pockets (the infected space between the tooth and gum).

Biofilm Resilience: Bacteria in plaque organize into a complex "biofilm" that is up to 1,000 times more resistant to antibiotics than free-floating bacteria.

No Removal of Cause: Antibiotics kill bacteria but do nothing to remove the hard deposits of tartar that are irritating the gums and causing the body's destructive inflammatory response.

The Proper Role of Antibiotics.

Antibiotics are used as a secondary, adjunctive treatment in specific situations, *after* the physical cause has been removed. A periodontist (gum specialist) might prescribe them for:

Aggressive Periodontitis: Rapidly progressing disease that can destroy bone quickly.

Acute Infections: Such as a periodontal abscess (a pus-filled pocket).

Patients with Weakened Immune Systems: Including those undergoing chemotherapy or with uncontrolled diabetes.

As a short-term adjunct: Following a deep cleaning procedure called Scaling and Root Planing (SRP) to help kill any remaining bacteria and improve healing.

The Correct, Proven Treatment for Gum Disease.

Standard, effective treatment follows a step-by-step process:

Scaling and Root Planing (SRP): This is the gold standard. The dentist or hygienist uses hand instruments and ultrasonic scalers to physically scrape away all plaque and tartar from above and below the gumline. They then smooth the tooth root so gums can reattach.

Improved Home Care: You must become meticulous with brushing twice a day, flossing daily, and possibly using interdental brushes or a water flosser. This removes new plaque before it can harden.

Antibiotics (If needed): Your dentist might place local, sustained-release antibiotics (like Arestin or Atridox) directly *into* the cleaned gum pockets. Oral antibiotics (pills) are less common but may be used for aggressive cases.

Surgical Intervention (For advanced cases): If SRP fails, procedures like flap surgery (to clean deep pockets directly) or bone grafting may be necessary.

When Might Antibiotics Seem to "Work" Alone?

If you have gingivitis (the mild, reversible form of gum disease with no bone loss, just bleeding gums), a course of antibiotics might temporarily reduce the bacteria and stop the bleeding.

However, this is dangerous. The tartar has likely already started to form. As soon as you stop the antibiotics, the bacteria living *under* the tartar will quickly repopulate, and you'll be left with the same problem, now potentially with antibiotic-resistant bacteria.

The Bottom Line.

	Antibiotics Alone.	Scaling & Root Planing.
Removes Tartar?	No.	Yes.
Removes Biofilm?	No, barely penetrates.	Yes, physically disrupts it.
Cures Gum Disease?	No.	Yes (in early/moderate cases).
Risk of Resistance?	High.	None.

Do not take antibiotics for gum disease unless prescribed by a dentist alongside a definitive mechanical cleaning. Using old antibiotics or pressuring a doctor for a prescription will only waste time, allow the disease to destroy more bone, and create resistant bacteria.

See a dentist for a proper evaluation. The treatment is almost always a physical deep cleaning. Antibiotics are just occasional helpers, not the cure.

Q29 After gum disease treatment, will I ever need treatment again?

This is a very common concern, and the honest answer is: possibly, but not necessarily in the way you might think.

Here's the breakdown of what "treatment again" really means after gum disease (periodontitis) treatment.

The Short Answer.

You will likely need *maintenance* treatments for life. These are regular, professional cleanings every 3-4 months. Think of these as the dental version of a blood pressure check or an oil change for your car.

You may need *retreatment* if the disease recurs. This means a repeat of the deep cleaning (scaling and root planing) or even further procedures like flap surgery. This happens if maintenance is skipped or home care isn't effective.

Why Recurrence is Possible.

Gum disease is a chronic, inflammatory condition, much like high blood pressure or diabetes. The bacteria that cause it are always present in your mouth. Treatment doesn't "cure" it forever; it *controls* it and puts it into remission.

You become vulnerable again if:

You don't keep your maintenance appointments. The biggest risk factor by far.

Your home care slips. Inconsistent brushing, not flossing, or not cleaning under bridges or around implants.

You have risk factors you can't change, like genetics or certain medications.

You develop new risk factors, like starting smoking or developing diabetes.

Your immune system changes (due to aging, stress, or other illnesses).

The Two Types of "Treatment Again".

	Maintenance (Supportive Periodontal Therapy).	Retreatment (Active Therapy Again).
Purpose.	Prevent relapse and keep disease in remission.	Stop an active, recurring infection.
What it is.	A specialized cleaning of your root surfaces, checking pocket depths, and reviewing your home care.	A repeat of scaling & root planing (deep cleaning) or possibly surgery.
How often?	Every 3-4 months, for life.	Only when active disease is detected (e.g., bleeding, deeper pockets).
Is it normal?	Yes, this is the standard of care after gum disease.	It's a sign that maintenance or home care has broken down.

What Does the Evidence Say?

With excellent home care and strict 3-month maintenance, the chance of needing another round of deep cleaning or surgery is very low (less than 5-10% over 5 years).

With no maintenance (or only yearly cleanings), the chance of needing retreatment within 1-2 years is over 80% .

The disease can progress faster the second time, leading to more bone loss and possibly tooth loss.

So, Will *You* Need Treatment Again?

You can almost guarantee you will NOT need active retreatment if you:

Never miss a maintenance visit (every 3-4 months as prescribed by your periodontist).

Floss or use interdental brushes daily and brush twice a day with excellent technique.

Manage your risk factors (stop smoking, control diabetes, reduce stress).

Alert your dentist to any changes (bleeding, loose teeth, bad taste).

You are very likely to need retreatment (another deep cleaning or surgery) if you:

Skip maintenance for 6+ months.

Resume smoking or fail to control blood sugar if diabetic.

Notice bleeding or gum swelling again but wait months to see a dentist.

The Most Important Thing to Know.

The goal of gum disease treatment is not a "one and done" fix. It's to transition you from active treatment to a lifelong *maintenance* program.

Your next appointment isn't a sign of failure it's the key to success. Many people with well-controlled gum disease keep their teeth for a lifetime, but they do so with the understanding that their mouths need ongoing professional support, just like a heart patient needs regular cardiology checkups.

Takeaway: You will absolutely need regular maintenance care. You will likely *not* need aggressive retreatment if you stick to that maintenance schedule. Ask your periodontist what your specific risk level is and follow their recall schedule precisely.

Q30 Is surgery always necessary for gum treatment?

This is an excellent question, as many people fear that gum disease automatically means surgery. The short answer is no, surgery is not always necessary. In fact, the vast majority of early to moderate gum disease can be treated successfully with non-surgical methods.

However, the best treatment depends entirely on the stage and severity of the gum disease.

Let's break it down by the typical stages of gum disease:

Gingivitis (Early, Reversible Stage).

What it is: Inflammation of the gums caused by plaque buildup. Gums are red, swollen, and bleed easily. There is no permanent damage to the bone or tissue holding your teeth.

Is surgery needed? Absolutely not.

Treatment: Professional dental cleaning (prophylaxis) to remove plaque and tartar, combined with improved daily brushing and flossing. The condition is completely reversible at this stage.

Periodontitis (Moderate Stage).

What it is: The infection has spread below the gum line, and the body's inflammatory response has started to destroy the bone and connective tissue that hold teeth in place. "Pockets" (spaces between the tooth and gum) begin to form.

Is surgery needed? Usually not. The gold standard treatment here is non-surgical.

Treatment: Scaling and Root Planing (SRP) . This is a deep-cleaning procedure, often done with local anesthesia. The dentist or hygienist scrapes away plaque and tartar from deep below the gum line and then smooths the tooth's root surfaces. This helps the gums reattach to the tooth and the pockets to shrink. For most patients with moderate disease, SRP is highly effective, and no further surgery is needed.

Advanced Periodontitis (Severe Stage).

What it is: Significant bone loss (50% or more), deep pockets (typically 6mm or deeper), gum recession, teeth may become loose or shift, and pus may appear.

Is surgery needed? Often, yes. While scaling and root planing is still the critical first step, if deep pockets and bone loss remain after healing from SRP, surgery is frequently recommended to save the teeth.

Surgical Treatments (When they become necessary):

Flap Surgery (Pocket Reduction Surgery): The gums are lifted back to expose the tooth roots and underlying bone. This allows for a thorough deep cleaning of the roots and bone surface. The gum tissue is then stitched back in place, often closer to the tooth, to reduce the pocket depth.

Bone Grafting: Performed with flap surgery. Bone graft material is placed where bone has been lost to help regenerate new bone.

Guided Tissue Regeneration: A special membrane is placed to encourage your body to regrow lost bone and tissue.

Gum Graft Surgery: Used to cover exposed tooth roots (recession) caused by advanced disease or over-vigorous brushing. Tissue is taken from the roof of your mouth and stitched to the receded area.

Key Factors That Determine if Surgery is Needed:

A dentist or periodontist (gum specialist) will decide based on several measurable factors:

Probing Pocket Depths: Healthy pockets are 1-3mm deep. 4-5mm may be treatable without surgery. 6mm or deeper often require surgery to clean effectively and maintain long-term.

Amount of Bone Loss: Seen on X-rays. Minor to moderate bone loss can be managed non-surgically. Severe bone loss often requires regeneration procedures.

Tooth Mobility: Loose teeth may require advanced surgical or even extraction.

Response to Non-Surgical Treatment: Most patients will re-evaluate 4-8 weeks after scaling and root planing. If pockets have reduced and gums are healthy, no surgery is needed. If deep pockets persist, surgery is the next step.

The Most Important Takeaway.

The best way to avoid gum surgery is early detection and treatment.

Gingivitis can be cured with a simple cleaning.

Moderate periodontitis is usually controlled with deep cleaning (scaling and root planing).

Advanced periodontitis is the stage where surgery often becomes necessary to prevent tooth loss.

Bottom Line: If your dentist recommends gum surgery, don't panic. Ask these critical questions first:

"What are my current pocket depths and bone loss levels?"

"Is there a non-surgical option (like scaling and root planing) that we can try first?"

"If I have scaling and root planing, how will we measure its success before deciding on surgery?"

A good dentist will always start with the least invasive, most effective treatment. Surgery is a powerful tool, but it is usually a last resort, not a first line of defense.
