# Supplementary material for: Comparative Evaluation of Large Language Models in Responding to Common Misconceptions Regarding Periodontal Disease and Oral Hygiene
Source: Diagnostics (Basel). 2026 Sep 28;16(19):3158. doi: 10.3390/diagnostics16193158 (PMC13664004; doi:10.3390/diagnostics16193158)
Supplement: Supplementary file 1 [file diagnostics-16-03158-s001.zip › Supplementary File S5 LLMs response stylistic elements removed.zip!/1. LLMs response/GPT-5.5.pdf]

Q1 Do gum diseases only affect elderly people?

No. Gum disease can affect people of almost any age, including teenagers and young adults.

The two main forms are:

Gingivitis – the early stage, where gums become red, swollen, or bleed easily. It is common in children, teens, and adults.

Periodontitis – a more advanced form that can damage the bone and tissues supporting the teeth. It becomes more common with age because damage accumulates over time, but younger adults can develop it too.

Factors that increase the risk of gum disease include:

Poor oral hygiene and plaque buildup.

Smoking or tobacco use.

Diabetes.

Hormonal changes (such as during pregnancy).

Certain medications that reduce saliva or affect gum tissue.

Genetic susceptibility.

Stress and some medical conditions.

Common signs include:

Bleeding when brushing or flossing.

Red, swollen, or tender gums.

Persistent bad breath.

Receding gums.

Loose teeth (in more advanced cases).

While older adults tend to have higher rates of severe gum disease, age itself is not the cause. Gum disease is primarily related to bacterial plaque, oral hygiene, health conditions, and other risk factors.

If you're noticing bleeding gums or other symptoms, a dental examination can help determine whether it's gingivitis (often reversible) or a more advanced condition.

Q2 Is it normal if my gums bleed when I brush my teeth?

Occasional gum bleeding can happen, but it is not considered normal if your gums regularly bleed when you brush or floss.

The most common cause is gum inflammation (gingivitis), which develops when plaque builds up around the gumline. Early signs often include:

Bleeding during brushing or flossing.

Red, swollen, or tender gums.

Bad breath.

Other possible causes include:

Brushing too aggressively or using a hard-bristled toothbrush.

Recently starting a flossing routine (mild bleeding may occur temporarily).

Poorly fitting dental appliances.

Vitamin deficiencies (especially vitamin C or vitamin K, though these are less common).

Certain medications, such as blood thinners.

Hormonal changes (for example, during pregnancy).

Less commonly, medical conditions that affect blood clotting.

A few things you can do:

Continue brushing twice daily with a soft-bristled toothbrush.

Floss gently every day.

Avoid scrubbing your gums hard.

Consider scheduling a dental cleaning and examination if you haven't had one recently.

You should see a dentist if:

The bleeding persists for more than about 1 2 weeks despite good oral hygiene.

Your gums are swollen, painful, or receding.

Your teeth feel loose.

The bleeding is heavy or occurs without brushing.

If you tell me your age, how long this has been happening, and whether the bleeding is mild (a little blood in the sink) or more significant, I can help narrow down the likely cause.

Q3 Can I get gum disease even if I brush my teeth regularly?

Yes. Brushing regularly greatly reduces your risk of gum disease, but it does not eliminate it.

Gum disease (also called Periodontal Disease) is caused by plaque and bacteria accumulating around the gums. Several factors can contribute even when you brush consistently:

Not cleaning between teeth: A toothbrush often misses areas where plaque builds up. Flossing or using interdental brushes helps remove it.

Brushing technique: Brushing twice a day is beneficial, but brushing too quickly or missing areas can leave plaque behind.

Tartar buildup: Once plaque hardens into tartar, it cannot be removed by brushing alone and usually requires professional cleaning.

Smoking or vaping: These significantly increase the risk of gum disease.

Medical conditions: Conditions such as Type 2 Diabetes can make gum disease more likely and harder to control.

Genetics: Some people are more susceptible despite good oral hygiene.

Certain medications: Some drugs can affect gum health or reduce saliva production.

Early gum disease often causes:

Red, swollen, or tender gums.

Bleeding when brushing or flossing.

Persistent bad breath.

If untreated, it can progress to more serious forms that may damage the bone supporting the teeth.

A good preventive routine includes:

Brushing twice daily with fluoride toothpaste.

Cleaning between teeth daily (floss or interdental brushes).

Regular dental checkups and professional cleanings.

Avoiding tobacco products.

If you're brushing regularly but notice bleeding gums, gum recession, or persistent bad breath, it's a good idea to see a dentist for an evaluation. How often do you brush and floss currently?

Q4 Can I have gum disease even if my gums don't hurt?

Yes. Gum disease can be present even when your gums don't hurt.

In its early stages, gum disease especially Gingivitis often causes little or no pain. Many people don't realize they have it until they notice signs such as:

Gums that bleed when brushing or flossing.

Red, swollen, or puffy gums.

Persistent bad breath.

Receding gums (teeth looking longer).

Increased sensitivity.

Loose teeth or changes in how teeth fit together (more common in advanced disease).

As gum disease progresses to Periodontitis, damage can occur to the tissues and bone supporting the teeth, sometimes still without significant pain.

If your gums don't hurt but you have bleeding, swelling, bad breath, gum recession, or other changes, it's worth having a dental examination. A dentist or dental hygienist can measure gum pockets and look for signs that aren't obvious at home.

If you'd like, tell me what symptoms you're noticing (bleeding, redness, recession, bad breath, etc.), and I can help you understand how concerning they might be.

Q5 Can gum disease get better without treatment?

It depends on the stage of the gum disease.

Early-stage gum disease (gingivitis) can sometimes improve if you thoroughly remove plaque every day with effective brushing, flossing/interdental cleaning, and good oral hygiene habits. Redness, swelling, and bleeding may decrease, and the gums can return to a healthier state.

More advanced gum disease (periodontitis) generally does not get better on its own. Once the infection has damaged the tissues and bone supporting the teeth, professional dental treatment is usually needed

to stop further damage. The lost bone and attachment typically do not regrow without specific treatments.

Signs that suggest you should see a dentist include:

Gums that bleed regularly when brushing or flossing.

Persistent bad breath.

Swollen, red, or tender gums.

Receding gums.

Loose teeth.

Pus around the gums.

If you think you might have gum disease, it's a good idea to have it evaluated. Early treatment is usually much simpler and more effective than waiting until it progresses.

If you'd like, you can describe your symptoms (for example, bleeding, swelling, pain, bad breath, loose teeth, how long it's been happening), and I can help you understand what stage it might resemble.

Q6 Are gum diseases rare?

No, gum diseases are very common.

The two main forms are:

Gingivitis the early stage, causing red, swollen, or bleeding gums.

Periodontitis a more serious form that can damage the tissues and bone supporting the teeth.

Globally, mild gum inflammation affects a large portion of adults at some point in their lives, and severe periodontitis is one of the most common chronic diseases worldwide.

Common risk factors include:

Inadequate brushing and flossing.

Smoking or tobacco use.

Diabetes.

Dry mouth.

Certain medications.

Hormonal changes (such as during pregnancy).

Genetic susceptibility.

Signs that may suggest gum disease:

Bleeding when brushing or flossing.

Red, swollen, or tender gums.

Persistent bad breath.

Receding gums.

Loose teeth (in more advanced cases).

The good news is that early gum disease is often reversible with good oral hygiene and professional dental cleaning. More advanced disease can usually be managed and treated, especially when caught early.

If you're asking because you've noticed symptoms like bleeding gums, bad breath, or gum recession, I can help you understand what might be going on and whether it's worth seeing a dentist soon.

Q7 Is it normal to lose teeth because of gum disease as you get older?

No. While gum disease becomes more common with age, losing teeth is not a normal or inevitable part of aging.

The most common cause of tooth loss in adults is advanced gum disease, specifically Periodontitis. In periodontitis, bacteria and inflammation damage the gums, bone, and other tissues that support the teeth. Over time, teeth can become loose and eventually fall out or require extraction.

Common signs of gum disease include:

Bleeding when brushing or flossing.

Red, swollen, or tender gums.

Persistent bad breath.

Receding gums (teeth look longer).

Loose or shifting teeth.

Pain when chewing (sometimes).

The good news is that many cases can be prevented or slowed with:

Regular brushing and flossing.

Professional dental cleanings and checkups.

Avoiding tobacco products.

Managing conditions such as Type 2 Diabetes if present.

If you are already noticing loose teeth, gum recession, or bleeding gums, it's worth seeing a dentist promptly. Treatment is often more effective before significant bone loss occurs.

Are you asking generally, or are you concerned about your own gums or teeth?

Q8 If gum disease runs in my family, will I definitely get it too?

No. A family history of gum disease does not mean you will definitely get it.

What can run in families is a combination of factors:

Genetics: Some people inherit immune-system traits that make them more susceptible to inflammation and periodontal (gum) disease.

Shared habits and environment: Family members may have similar oral hygiene routines, diets, smoking exposure, or access to dental care.

Medical conditions: Certain health conditions that can have hereditary components, such as diabetes, can also increase gum disease risk.

Even if you have a genetic predisposition, gum disease is not purely determined by your genes. The main trigger is the buildup of bacterial plaque around the teeth and gums. Many people with a family history never develop significant gum disease, while some people without a family history do.

To lower your risk:

Brush thoroughly twice a day with fluoride toothpaste.

Clean between your teeth daily (floss or interdental brushes).

Get regular dental checkups and cleanings.

Avoid smoking and tobacco products.

Manage conditions such as diabetes if applicable.

If multiple close relatives have had severe gum disease or tooth loss from gum problems, it's worth mentioning that to your dentist. They may recommend closer monitoring of your gum health.

Are you asking because a parent or sibling has gum disease, or because you've noticed symptoms such as bleeding gums yourself?

Q9 Can stress make me more likely to get gum disease?

Yes. Stress can increase your risk of gum disease, although it usually isn't the sole cause.

Here's how stress can contribute:

Weakened immune response: Chronic stress can make it harder for your immune system to control the bacteria that naturally live in your mouth, allowing gum inflammation to develop more easily.

Increased inflammation: Stress hormones such as cortisol can promote inflammatory responses that may worsen gum irritation and tissue damage.

Changes in habits: When stressed, some people brush and floss less consistently, eat more sugary foods, smoke more, or drink more alcohol all of which can increase the risk of gum problems.

Teeth grinding or clenching: Stress-related grinding (bruxism) can put extra strain on the teeth and supporting tissues, potentially aggravating existing gum issues.

Dry mouth: Stress and some medications used for anxiety or depression can reduce saliva flow. Saliva helps protect the mouth from bacterial overgrowth.

Common signs of gum disease include:

Red, swollen, or tender gums.

Bleeding when brushing or flossing.

Persistent bad breath.

Receding gums.

Loose teeth (in more advanced cases).

To reduce your risk:

Brush twice daily with fluoride toothpaste.

Clean between your teeth daily (floss or interdental brushes).

Keep up with regular dental checkups and cleanings.

Manage stress through exercise, sleep, social support, mindfulness practices, or other techniques that work for you.

If you're noticing bleeding gums, swelling, pain, or gum recession, it's worth seeing a dentist, as early gum disease is often reversible with proper care. How long have you been under increased stress, and are you experiencing any gum symptoms right now?

Q10 Do gum diseases only affect the mouth and teeth?

No. Gum disease can start in the mouth, but its effects may extend beyond the gums and teeth.

The most common forms are Gingivitis and Periodontitis. In periodontitis, bacteria and inflammation can damage the tissues and bone that support the teeth, potentially leading to tooth loss.

Researchers have also found associations between gum disease and several systemic health conditions, including:

Cardiovascular Disease.

Type 2 Diabetes.

Rheumatoid Arthritis.

Alzheimer's Disease.

Pregnancy complications such as preterm birth and low birth weight.

It's important to understand that many of these links are associations rather than proof that gum disease directly causes those conditions. However, chronic inflammation and the spread of oral bacteria into the bloodstream are thought to be possible mechanisms connecting oral health with overall health.

Signs of gum disease can include:

Red, swollen, or tender gums.

Bleeding when brushing or flossing.

Persistent bad breath.

Receding gums.

Loose teeth (in more advanced disease).

Good oral hygiene, regular dental checkups, and treatment of gum disease can improve oral health and may also reduce inflammation throughout the body.

If you're concerned about your own symptoms, tell me what you've noticed (for example, bleeding gums, gum recession, pain, or loose teeth), and I can explain what those signs might indicate.

Q11 Will a hard-bristled toothbrush clean my teeth better?

Not necessarily. In fact, most dentists recommend a soft-bristled toothbrush rather than a hard-bristled one.

A hard-bristled brush can:

Wear down tooth enamel over time, especially if you brush aggressively.

Cause gum recession by irritating or damaging gum tissue.

Increase tooth sensitivity if enamel becomes thinner.

A soft-bristled brush can:

Remove plaque effectively when used with proper technique.

Reach along the gumline more gently.

Reduce the risk of damaging teeth and gums.

What matters most is:

Brushing for about 2 minutes, twice a day.

Using gentle, small circular motions rather than scrubbing hard.

Replacing the brush head (or toothbrush) every 3 4 months.

Cleaning between teeth with floss or interdental brushes.

If you feel your teeth aren't getting clean with a soft brush, the issue is usually brushing technique, brushing time, or plaque buildup not that the bristles are too soft. An electric toothbrush with a soft brush head can also be very effective.

Do you mean a manual toothbrush or an electric toothbrush?

Q12 Should I stop brushing my teeth for a few days if my gums are bleeding?

No in most cases, you should continue brushing your teeth even if your gums are bleeding, but do it gently.

Bleeding gums are commonly caused by inflammation from plaque buildup (early gum disease, also called gingivitis). If you stop brushing, more plaque accumulates, which can make the inflammation and bleeding worse.

A few suggestions:

Use a soft-bristled toothbrush.

Brush gently along the gumline twice a day.

Floss carefully once a day (gums may bleed at first if they're inflamed, but this often improves with consistent cleaning).

Avoid aggressive scrubbing.

Consider an antiseptic mouthwash if recommended by your dentist.

However, bleeding gums can sometimes have other causes, including:

Brushing too hard or using a hard-bristled brush.

Poorly fitting dental appliances .

Vitamin deficiencies (especially vitamin C or K) .

Certain medications (such as blood thinners) .

Hormonal changes .

More advanced gum disease.

You should consider seeing a dentist if:

The bleeding has lasted more than about 1 2 weeks despite good oral hygiene.

Your gums are swollen, painful, receding, or you have persistent bad breath.

Teeth feel loose.

The bleeding is heavy, spontaneous, or occurs elsewhere in your body as well.

If you tell me:

How long your gums have been bleeding,

Whether it's only while brushing/flossing or also at other times,

Your age and any medications you take,

I can help you think through the most likely causes.

Q13 Do I need to brush my teeth after every meal to keep my gums healthy?

No. For most people, brushing after every meal is not necessary to keep gums healthy.

The main habits that support gum health are:

Brush thoroughly twice a day with a soft-bristled toothbrush and fluoride toothpaste.

Clean between your teeth once a day (floss, interdental brushes, or a water flosser, depending on your needs).

Get regular dental checkups and professional cleanings.

Avoid smoking or tobacco use, which significantly increases gum disease risk.

A few additional points:

If you've eaten or drunk something acidic (such as citrus fruits, soda, sports drinks, or wine), it's best to wait about 30 60 minutes before brushing. Acid temporarily softens the tooth surface, and brushing immediately can increase wear.

If food tends to get stuck between your teeth, cleaning between the teeth is often more important for gum health than adding extra brushing sessions.

If you're at high risk for gum disease, have braces, or have been given specific instructions by your dentist, they may recommend cleaning after meals.

If your main concern is preventing gum disease, brushing twice daily plus daily flossing/interdental cleaning is generally more effective than brushing after every meal but neglecting the spaces between the teeth.

Q14 Even if I brush my teeth regularly, do I still need to get my gums checked?

Yes. Regular brushing is important, but it doesn't completely rule out gum problems.

Gum disease can develop gradually and often causes few or no symptoms in its early stages. Even people who brush twice a day can develop issues if plaque and tartar accumulate below the gumline, if they miss certain areas while cleaning, or because of factors such as genetics, smoking, diabetes, certain medications, or hormonal changes.

It's a good idea to have your gums checked during routine dental visits. A dentist or dental hygienist can look for signs such as:

Red, swollen, or tender gums.

Bleeding when brushing or flossing.

Receding gums.

Deep pockets between the teeth and gums.

Persistent bad breath.

Loose teeth or changes in how your teeth fit together.

Many dental professionals recommend a checkup every 6–12 months, though some people need more frequent visits based on their risk factors and oral health history.

If you've noticed bleeding gums, gum recession, persistent bad breath, or tooth looseness, it's worth scheduling a dental exam sooner rather than waiting for your next routine visit.

Q15 Is brushing my teeth once a day enough to keep my mouth healthy?

For most people, brushing once a day is better than not brushing, but it is generally not considered enough for optimal oral health.

Most dental organizations, including the American Dental Association, recommend:

Brushing twice a day with fluoride toothpaste.

Brushing for about 2 minutes each time.

Cleaning between teeth daily (floss, interdental brushes, or a water flosser).

Regular dental checkups.

Why once a day may not be enough:

Plaque begins to build up continuously after eating and drinking.

Overnight, bacteria can accumulate in the mouth, especially if you don't brush before bed.

Brushing only once leaves longer periods where plaque and acids can affect teeth and gums.

The risk of cavities, gum inflammation, and bad breath tends to be higher compared with brushing twice daily.

That said, the impact depends on factors such as:

Your cavity risk and genetics.

Diet (especially sugary or acidic foods and drinks).

Whether you floss.

Saliva production and medications.

Existing gum disease or dental work.

If you currently brush only once a day, adding a nighttime brushing before bed usually provides the biggest benefit, since it removes plaque and food debris before the long period when saliva flow decreases during sleep.

If you're wondering about your own situation (age, diet, whether you floss, and whether you've had cavities), I can give a more personalized assessment.

Q16 Can flossing cause gum recession?

Flossing itself does not usually cause gum recession when done correctly. In fact, regular flossing helps reduce plaque and inflammation, which can protect your gums and support long-term gum health.

However, aggressive or improper flossing can irritate or injure the gums, especially if you:

Snap the floss forcefully into the gum tissue.

Saw back and forth harshly against the gums.

Use excessive pressure every day.

Repeatedly traumatize the same area.

This irritation may cause soreness, bleeding, or small cuts. Over time, repeated trauma could potentially contribute to localized gum damage, though flossing is generally not considered a common cause of significant gum recession.

More common causes of gum recession include:

- Gum disease (periodontitis).
- Brushing too hard or using a hard-bristled toothbrush.
- Naturally thin gum tissue or genetic factors.
- Teeth grinding or clenching.
- Tobacco use.
- Misaligned teeth or bite issues.

To floss safely:

- Gently guide the floss between the teeth instead of snapping it down.
- Curve the floss into a "C" shape around one tooth.
- Slide it up and down against the tooth surface and slightly under the gumline.
- Repeat on the adjacent tooth.

If you've noticed a specific area where the gums seem to be receding, or if flossing causes persistent pain or bleeding, it's worth having a dentist or periodontist examine it. They can determine whether the recession is related to technique, gum disease, or another cause.

If you'd like, you can describe what you're seeing (for example, which tooth, how long it's been happening, and whether there's bleeding or sensitivity), and I can help you think through the likely causes.

Q17 Do electric toothbrushes damage the gums more than regular toothbrushes?

No when used properly, electric toothbrushes do not generally damage gums more than regular toothbrushes. In fact, many studies have found that powered toothbrushes can remove more plaque and reduce gingivitis slightly better than manual brushes, without increasing gum damage.

The bigger factor is how you brush, not whether the brush is electric or manual.

What can damage gums?

With either type of toothbrush, gum irritation or recession can occur if you:

Brush too hard.

Use a hard-bristled brush.

Scrub aggressively along the gumline.

Brush for excessively long periods with heavy pressure.

Why electric toothbrushes can sometimes be gentler.

Many modern electric toothbrushes:

Have soft bristles.

Use oscillating or sonic movements that do the cleaning for you.

Include pressure sensors that warn you if you're pressing too hard.

Encourage proper brushing time with built-in timers.

These features can actually reduce the risk of over-scrubbing compared with manual brushing.

If your gums bleed after switching to an electric toothbrush.

A little bleeding during the first few days can happen if:

Plaque is being removed more effectively from inflamed gums.

You're still learning the correct technique.

However, persistent bleeding, pain, or worsening gum recession should be evaluated by a dentist, as it may indicate gum disease or brushing too aggressively.

Best practices.

Use a soft-bristled brush head.

Hold the brush lightly; don't press hard.

Let the brush move tooth by tooth rather than scrubbing back and forth.

Replace brush heads every 3 months (or sooner if bristles fray).

If you tell me which electric toothbrush model you're considering or already using I can explain whether it has any features that help protect your gums.

Q18 Would using mouthwash alone be enough without brushing my teeth?

No. Using mouthwash alone is generally not enough to keep your teeth and gums healthy.

Here's why:

Brushing physically removes plaque, a sticky film of bacteria that builds up on teeth. Mouthwash can reduce some bacteria, but it cannot scrub plaque off tooth surfaces effectively.

Plaque that isn't removed can harden into tartar, which contributes to cavities and gum disease.

Many mouthwashes help with specific issues such as bad breath, cavity prevention (fluoride rinses), or reducing gum inflammation, but they're intended as a supplement not a replacement for brushing.

A good basic routine is:

- Brush twice a day with fluoride toothpaste.
- Clean between teeth (floss, interdental brushes, or a water flosser).
- Use mouthwash if it addresses a need you have (e.g., fluoride for cavity prevention or an antiseptic rinse recommended by a dentist).

If you're asking because brushing is difficult due to pain, braces, disability, illness, or another reason, I can suggest alternatives that may work better than mouthwash alone.

Q19 After I quit smoking, my gums started bleeding. Is that normal?

Yes, this can happen, and it's a fairly well-recognized phenomenon after quitting smoking.

Smoking tends to suppress signs of gum inflammation by constricting blood vessels in the gums. When you stop smoking, blood flow to the gums improves. As a result, underlying gum disease or gum inflammation that was previously "masked" can become more noticeable, including bleeding during brushing or flossing.

Studies have found that some people experience increased gum bleeding in the weeks after quitting, even though their overall gum health is beginning to recover.

That said, bleeding gums are not something to ignore. Common possibilities include:

- Gingivitis (gum inflammation) often caused by plaque buildup.
- Periodontitis (gum disease) a more serious infection affecting the tissues supporting the teeth.
- Brushing or flossing changes.
- Less commonly, nutritional deficiencies, medications, or certain medical conditions.
- A few questions that would help clarify things:
- How long ago did you quit smoking?
- How much did you smoke before quitting?
- Do your gums bleed only when brushing/flossing, or do they bleed spontaneously?
- Have you noticed swelling, redness, bad breath, loose teeth, or gum recession?

In general, if the bleeding persists for more than a couple of weeks after quitting, or if you haven't had a dental cleaning recently, it's a good idea to schedule a dental exam. Quitting smoking is one of the best things you can do for your gum health, but it can reveal dental problems that were already present and worth treating.

Q20 Can natural products effectively keep my gums healthy?

Yes, some natural products can help support gum health, but they work best as complements to good oral hygiene not as replacements for brushing, flossing, and regular dental care.

Here are some of the natural approaches with the strongest evidence:

- Green tea: Contains compounds called catechins that may help reduce inflammation and inhibit bacteria associated with gum disease.
- Aloe vera: Some studies suggest aloe vera gels or mouth rinses can help reduce plaque and gum inflammation.

Tea tree oil: Has antimicrobial properties, but it should be used only in products specifically formulated for oral use and should not be swallowed.

Saltwater rinses: A simple warm saltwater rinse can temporarily soothe irritated gums and support healing after minor irritation.

Cranberry compounds: Certain compounds in cranberries may make it harder for harmful oral bacteria to adhere to teeth and gums.

Coenzyme Q10 (CoQ10): Research has explored its role in gum health, though evidence is mixed and generally less robust than standard periodontal treatments.

A few important points:

"Natural" does not automatically mean safe or effective. Some essential oils and herbal products can irritate oral tissues or interact with medications.

If you have symptoms such as bleeding gums, persistent bad breath, gum recession, loose teeth, or pain, natural remedies alone are unlikely to address the underlying problem.

The most effective routine for healthy gums remains brushing twice daily with fluoride toothpaste, cleaning between teeth (floss or interdental brushes), limiting tobacco use, and getting regular dental checkups.

If you're interested in a specific natural product such as coconut oil pulling, turmeric, clove oil, or herbal mouthwashes I can explain what the current evidence says about it.

Q21 Can getting a dental scaling damage my teeth?

In general, professional dental scaling (removal of plaque and tartar by a dentist or dental hygienist) does not damage healthy tooth structure when performed properly.

However, there are a few reasons people sometimes *feel* that their teeth were damaged after a scaling:

Increased sensitivity: Removing tartar can expose areas of the tooth that were previously covered, especially near the gumline. Cold drinks or air may feel more intense for a few days or weeks.

Teeth feel rough or different: If you've had significant tartar buildup, your teeth may feel strange afterward because you're feeling the actual tooth surface rather than the deposits.

Spaces between teeth seem larger: Tartar can fill gaps between teeth. Once it's removed, the natural spacing becomes noticeable.

Temporary gum soreness or minor bleeding: This is common, particularly if the gums were inflamed before treatment.

Actual damage from scaling is uncommon but can occur in certain situations:

Excessively aggressive instrumentation.

Repeated improper use of dental tools over many visits.

Existing conditions such as severe enamel wear, exposed root surfaces, or weakened teeth.

If after a scaling you experience:

Severe or worsening pain.

A chipped tooth.

A tooth that feels loose.

Sensitivity that persists for many weeks.

it's worth contacting your dentist for an evaluation.

If you tell me whether you had a routine scaling, deep cleaning (scaling and root planing), or ultrasonic scaling, and what symptoms you're experiencing, I can explain whether they're expected or something that should be checked.

Q22 Can dental scaling cause gaps between my teeth?

Yes, dental scaling (professional cleaning to remove plaque and tartar) can sometimes make it *appear* that gaps have developed between teeth, but the scaling itself does not create new spaces by removing healthy tooth structure.

What often happens is:

Tartar buildup can fill existing spaces between teeth and around the gums.

When the tartar is removed during scaling, those spaces become visible again.

If you had gum disease, inflammation may have caused swollen gums that concealed gaps. After treatment, the gums shrink back toward a healthier size, revealing spaces that were already there or were caused by previous gum and bone loss.

In some cases, people notice:

Small "black triangles" near the gumline between teeth.

More visible spacing after deep cleaning for periodontal disease.

Increased awareness of tooth shape and alignment once deposits are removed.

You should contact your dentist if:

The gaps seem to be getting larger after several weeks.

Teeth feel loose.

You have persistent bleeding, pain, or gum recession.

Your bite feels different.

If your scaling was recent (within the last few days or weeks), what you're seeing is often a normal result of removing tartar and reducing gum swelling. If you'd like, you can tell me:

How long ago the scaling was done.

Whether it was a routine cleaning or a deep cleaning.

Your age.

And whether the gap is between the front teeth or elsewhere,

and I can give a more specific explanation.

Q23 Do all gum diseases require the same treatment?

No. Gum disease is a spectrum of conditions, and treatment depends on the type, severity, and underlying causes.

The two main categories are:

Gingivitis.

Early-stage gum disease.

Causes redness, swelling, and bleeding gums.

Usually does not involve permanent damage to the bone or tissues supporting the teeth.

Treatment often includes:

Professional dental cleaning.

Improved brushing and flossing.

Addressing factors such as plaque buildup, smoking, or poorly fitting dental appliances.

Periodontitis.

More advanced gum disease.

Involves destruction of the tissues and bone supporting the teeth. Treatment may require:

Deep cleaning procedures (scaling and root planing).

More frequent maintenance visits.

Antibiotics in some cases.

Surgical procedures for severe disease.

Management of contributing conditions such as diabetes.

Other gum problems can mimic or contribute to gum disease and may require different approaches, including:

Gum infections caused by specific bacteria, viruses, or fungi.

Drug-related gum enlargement.

Autoimmune or inflammatory conditions affecting the gums.

Gum recession without active infection.

A dentist or periodontist typically determines treatment by examining:

Gum bleeding and inflammation.

Pocket depth around the teeth.

Bone loss on dental X-rays.

Overall health and risk factors.

So while all gum diseases involve the gums, they do not all require the same treatment. The appropriate treatment depends on the diagnosis and severity of the condition. If you have symptoms such as bleeding gums, persistent bad breath, loose teeth, or gum recession, a dental evaluation can help identify the specific problem and guide treatment.

Q24 Is gum treatment painful?

Most gum treatments are not very painful, because dentists use local anesthesia to numb the area. What you feel depends on the type of treatment and how severe the gum disease is.

Here's a general idea:

Deep cleaning (scaling and root planing):
Usually causes mild discomfort or pressure during the procedure. Your gums may feel sore or sensitive for a few days afterward.

Laser gum treatment:
Often less painful than traditional surgery and may have faster healing.

Gum surgery or grafting:
More invasive, so you can expect soreness, swelling, and tenderness for several days, but pain medication helps manage it.

Common after-effects include:

mild bleeding.

swollen gums.

tooth sensitivity.

tenderness when eating.

Most people say the discomfort is manageable and much less severe than they expected.

If you're nervous, tell your dentist or periodontist they can offer:

stronger numbing.

sedation options.

anxiety-reducing techniques.

Untreated gum disease can become much more painful over time, so treatment is usually worth it.

Q25 Can gum surgery cause tooth loss?

Yes, gum surgery can very rarely lead to tooth loss, but in most cases the surgery is performed to help save teeth, not endanger them.

Whether there's a risk depends on the type of surgery and the condition of the tooth and surrounding bone beforehand.

Common reasons a tooth might be lost after gum surgery include:

Advanced periodontal disease already present: Sometimes a tooth has already lost so much supporting bone and gum tissue that it cannot be saved, even with surgery.

Reduced support after treatment: Surgery may reveal how much bone loss has already occurred, and a tooth that seemed stable before may become noticeably looser afterward.

Infection or poor healing: Uncommon, but postoperative infection or impaired healing can damage supporting tissues.

Trauma to the tooth or surrounding structures: This is uncommon when the procedure is performed correctly.

Smoking, uncontrolled diabetes, or poor oral hygiene: These can increase the risk of complications and treatment failure.

It's also important to know that temporary tooth mobility (looseness) after gum surgery can occur and does not necessarily mean the tooth will be lost. Teeth may feel different as swelling resolves and tissues heal.

If you've recently had gum surgery and are experiencing:

Increasing tooth looseness,

Severe pain,

Pus or drainage,

Persistent bleeding,

A feeling that the tooth is shifting significantly,

you should contact your periodontist or dentist promptly for an evaluation.

If you tell me:

What type of gum surgery you had (gum graft, flap surgery, crown lengthening, regenerative surgery, etc.),

How long ago it was,

Which symptoms you're noticing,

I can give more specific information about what is expected during healing and what might be concerning.

Q26 Can gum treatment make my teeth feel loose?

Yes. After gum (periodontal) treatment, it's fairly common for teeth to feel looser, even when the treatment is working as intended.

Some reasons this can happen:

Inflamed gums shrink as they heal. Before treatment, swollen gum tissue can act like a "cushion" that makes teeth feel more stable. Once the inflammation goes down, you may notice movement that was previously hidden.

Tartar removal exposes the true condition. If you had significant buildup around the teeth, removing it can make the teeth feel different or less supported at first.

Existing bone loss becomes more noticeable. If gum disease has already damaged the supporting bone, treatment stops or slows the disease but cannot instantly replace lost support.

Temporary sensitivity or altered bite. After deep cleaning (scaling and root planing), teeth and gums can feel different for days to weeks.

However, there are situations where you should contact your dentist or periodontist:

The looseness is getting worse, not better.

You have significant pain, swelling, pus, or bleeding.

Your bite suddenly feels off and you're hitting one tooth first.

A tooth feels loose enough that you're worried about it coming out.

If you tell me:

What type of gum treatment you had (routine cleaning, deep cleaning/scaling and root planing, gum surgery, etc.),

How long ago it was done, and Which teeth feel loose,

I can give a better idea of whether what you're experiencing is expected.

Q27 Does tartar removal cause more tartar buildup?

No. Removing tartar (also called dental calculus) does not cause more tartar to build up.

This is a common misconception because after a dental cleaning:

Your teeth may feel rough or different for a short time as your tongue notices areas that were previously covered by tartar.

If tartar had filled gaps between teeth or along the gumline, those areas can feel more noticeable once it's removed.

Tartar can return if plaque continues to accumulate, which may make it seem like the cleaning "caused" it.

In reality, tartar forms when plaque (a sticky film of bacteria) remains on the teeth and hardens due to minerals in saliva. Once plaque has hardened into tartar, it generally cannot be removed by brushing alone and requires professional scaling.

Some people do accumulate tartar faster than others because of factors such as:

Saliva composition and mineral content.

Crowded teeth that are harder to clean.

Inconsistent brushing or flossing.

Smoking or tobacco use.

Dry mouth.

Regular brushing with fluoride toothpaste, cleaning between the teeth (floss or interdental brushes), and professional cleanings help reduce tartar buildup over time.

If you're asking because you've recently had tartar removed and feel like buildup is occurring faster afterward, I can explain what changes in your mouth might be making it seem that way.

Q28 Are antibiotics enough to treat gum disease?

It depends on the type and severity of the gum disease.

For most cases of gum disease, antibiotics alone are not enough. The main problem is the buildup of bacterial plaque and tartar on the teeth and under the gums. Antibiotics may temporarily reduce bacteria, but they generally cannot remove the plaque and tartar that allow the infection to continue.

Typical treatment includes:

Improved oral hygiene (brushing, flossing, cleaning between teeth) .

Professional dental cleaning .

Scaling and root planing (deep cleaning below the gumline) for more advanced disease .

Sometimes antibiotics (oral or locally applied) as an additional treatment .

For:

Gingivitis (early gum disease): professional cleaning and good oral hygiene are usually sufficient; antibiotics are rarely needed.

Periodontitis (more advanced gum disease): deep cleaning is usually the cornerstone of treatment. Antibiotics may be used in selected cases, especially if the disease is severe, rapidly progressing, or not responding well to standard treatment.

If you have symptoms such as swollen or bleeding gums, bad breath, loose teeth, gum recession, or pus around the gums, it's important to see a dentist. Delaying treatment can lead to permanent damage to the bone and tissues supporting the teeth.

If you're asking about a specific antibiotic or a diagnosis you've received, I can explain how it fits into treatment.

Q29 After gum disease treatment, will I ever need treatment again?

It depends on the severity of the gum disease and how well it's controlled afterward.

In most cases, gum disease (periodontitis) is managed rather than permanently cured. After successful treatment, the infection and inflammation can be brought under control, but you'll usually need ongoing maintenance to prevent it from returning.

Typical long-term expectations:

Regular periodontal maintenance visits (often every 3–4 months initially) are commonly recommended.

Good daily oral hygiene brushing, cleaning between teeth, and following your dentist's instructions greatly reduces the risk of recurrence.

Risk factors such as smoking, diabetes, certain medications, genetics, and dry mouth can increase the likelihood of needing additional treatment.

If gum disease returns or progresses, further treatment (such as deep cleaning, localized procedures, or surgery in some cases) may be necessary.

If your condition was limited to gingivitis (early-stage gum disease), treatment and good oral hygiene can often restore gum health completely. If you had periodontitis, ongoing monitoring and maintenance are usually part of long-term care.

If you'd like, tell me:

What type of treatment you had (deep cleaning/scaling and root planing, gum surgery, laser treatment, etc.),

How long ago it was,

And what your dentist said about the severity,

and I can give a more specific idea of what to expect.

Q30 Is surgery always necessary for gum treatment?

No, surgery is not always necessary for gum treatment. The right treatment depends on the severity and cause of the gum problem.

Common approaches include:

- Professional cleaning (scaling and polishing): Often enough for mild gum inflammation (gingivitis).
- Scaling and root planing (deep cleaning): Used when gum disease has progressed and pockets have formed around the teeth.
- Improved oral hygiene: Brushing twice daily, cleaning between teeth, and regular dental visits can help control early disease.
- Medications: Antimicrobial mouth rinses, localized antibiotics, or other treatments may be used in some cases.
- Surgery: Typically considered when gum disease is advanced, deep pockets remain after non-surgical treatment, there is significant bone loss, or gum tissue needs to be reshaped or regenerated.

If you're asking about a specific recommendation from your dentist or periodontist, sharing details such as:

- Your diagnosis (gingivitis, periodontitis, gum recession, etc.) .
- Pocket depths (if you know them) .
- Whether you've already had deep cleaning .
- Any symptoms (bleeding, loose teeth, recession) .

would help me explain why surgery may or may not have been recommended.
