# Supplementary material for: Comparative Evaluation of Large Language Models in Responding to Common Misconceptions Regarding Periodontal Disease and Oral Hygiene
Source: Diagnostics (Basel). 2026 Sep 28;16(19):3158. doi: 10.3390/diagnostics16193158 (PMC13664004; doi:10.3390/diagnostics16193158)
Supplement: Supplementary file 1 [file diagnostics-16-03158-s001.zip › Supplementary File S5 LLMs response stylistic elements removed.zip!/1. LLMs response/Grok 4.3.pdf]

Q1 Do gum diseases only affect elderly people?

No, gum diseases (such as gingivitis and periodontitis) do not only affect elderly people. They can occur at any age, though the risk and severity generally increase as people get older.

Key Facts on Prevalence.

Gingivitis (the milder, reversible early stage) is very common and can affect children, teens, and adults. Nearly all children and adolescents show some signs of it.

Periodontitis (the more serious form that can damage gums, bone, and teeth) is common in adults. According to CDC data, about 4 in 10 U.S. adults aged 30+ have some level of periodontitis.

Prevalence rises with age: 30% in ages 30 44, 46% in 45–64, and 60% in those 65.

Severe forms can appear in younger people too. Aggressive (or juvenile) periodontitis is a rarer but rapid form that mainly affects teens and young adults, often targeting molars and incisors.

Why It's More Common in Older Adults

Older adults face higher rates due to cumulative factors like:

Longer exposure to plaque and poor oral hygiene.

Age-related changes in gums and bone.

Higher rates of conditions like diabetes, dry mouth (from medications), or reduced dexterity for brushing/flossing.

Smoking history or other lifestyle factors.

However, these are risk factors, not inevitabilities gum disease is largely preventable with good oral hygiene, regular dental visits, and managing health conditions.

Bottom Line.

Gum disease is driven primarily by bacteria in plaque, not just aging. Anyone with poor oral care, tobacco use, genetics, certain medical conditions, or hormonal changes (e.g., pregnancy) can develop it. Early detection through dental check-ups is key at any age to prevent progression to tooth loss or links to other health issues like heart disease. If you have symptoms like bleeding gums, bad breath, or loose teeth, see a dentist regardless of your age.

Q2 Is it normal if my gums bleed when I brush my teeth?

No, it's not normal for your gums to bleed when you brush your teeth. Healthy gums are firm, pink, and shouldn't bleed during regular brushing or flossing.

Why It Happens.

The most common cause is gingivitis (early gum disease), which results from plaque buildup along the gumline. Plaque is a sticky film of bacteria that irritates the gums, leading to inflammation, swelling, and bleeding. If left untreated, it can progress to periodontitis, which damages the bone and tissues supporting your teeth and can lead to tooth loss.

Other possible reasons include:

Brushing too hard or with a hard-bristled toothbrush.

Not flossing regularly (or starting flossing after a long break).

Hormonal changes (e.g., pregnancy), vitamin deficiencies (like vitamin C or K), certain medications, or other health conditions.

Occasional minor bleeding might happen if you're starting a new routine or brushing more vigorously, but persistent or regular bleeding is a warning sign that needs attention.

What You Can Do Right Away.

Improve your technique: Use a soft-bristled toothbrush and gentle circular motions (not back-and-forth scrubbing). Brush for two full minutes twice a day.

Floss daily: This removes plaque between teeth that brushing misses.

Use an antimicrobial mouthwash to help reduce bacteria.

Consider an electric toothbrush with a pressure sensor if you tend to brush too hard.

With consistent better hygiene, mild gingivitis often improves within 1 2 weeks.

When to See a Dentist.

See a dentist promptly if:

Bleeding continues for more than 1 2 weeks despite better care.

You have other symptoms like red/swollen/painful gums, bad breath, receding gums, or loose teeth.

It's frequent or getting worse.

Early professional cleaning (scaling) can reverse gingivitis quickly. Don't ignore it gum disease is very common but highly preventable and treatable when caught early.

Important: This is general information, not personalized medical advice. A dentist or doctor can evaluate your specific situation, rule out other causes, and recommend the best treatment. Schedule a check-up for peace of mind!

Q3 Can I get gum disease even if I brush my teeth regularly?

Yes, you can still develop gum disease (gingivitis or periodontitis) even if you brush your teeth regularly. Brushing is essential but often not enough on its own to fully prevent it.

Why This Happens.

Gum disease is primarily driven by plaque a sticky film of bacteria that builds up on and between teeth. If not removed effectively, it irritates the gums, leading to inflammation (gingivitis) and potentially progressing to periodontitis, which damages the tissues and bone supporting your teeth.

Brushing limitations: A toothbrush cleans the visible surfaces of teeth well but struggles to reach tight spaces between teeth and below the gumline, where plaque and tartar (hardened plaque) often accumulate.

Technique and thoroughness matter: Many people don't brush for the full recommended 2 minutes twice daily, use improper technique (e.g., not angling the brush at 45 degrees to the gums), or use a toothbrush that's too hard or worn out. Aggressive brushing can even irritate gums and contribute to recession.

Other Key Risk Factors.

Even with diligent brushing (and flossing), several factors increase susceptibility:

Lack of flossing or interdental cleaning: This is a major gap. Flossing (or using interdental brushes/water flossers) removes plaque from areas brushing misses and is strongly recommended daily for gum health.

Genetics: Some people have immune responses or bacterial profiles that make them more prone to gum issues, regardless of hygiene.

Medical conditions: Diabetes, autoimmune diseases, hormonal changes (e.g., pregnancy, menopause), and dry mouth (from medications or conditions) heighten risk.

Lifestyle factors: Smoking/tobacco use is one of the biggest risks it impairs healing and blood flow to gums. Stress, poor nutrition, obesity, and certain medications also play roles.

Professional care gaps: Plaque that hardens into tartar requires dental cleanings (every 6 months or more often if at risk). At-home care alone can't remove it.

Prevention Tips Beyond Brushing.

Floss daily (or use alternatives like water flossers).

Use proper technique: Soft-bristled brush, fluoride toothpaste, gentle circular or back-and-forth motions.

Consider antimicrobial mouthwash.

Eat a balanced diet low in sugars, stay hydrated, and avoid tobacco.

Visit your dentist regularly for checkups and cleanings they can spot early signs and provide tailored advice.

Early gum disease is often reversible with improved habits and professional care, but advanced stages can lead to tooth loss if ignored. If you notice bleeding, swelling, bad breath, or receding gums, see a dentist promptly even if your brushing routine feels solid.

Q4 Can I have gum disease even if my gums don't hurt?

Yes, you can have gum disease even if your gums don't hurt.

Gum disease (also called periodontal disease) includes milder forms like gingivitis and more serious ones like periodontitis. It often progresses silently without significant pain, especially in the early stages. This is one reason many people don't realize they have it until it has advanced.

Why It Can Be Painless.

The disease is driven by plaque and bacteria causing inflammation and gradual tissue/bone damage, but this process frequently doesn't trigger strong pain signals early on.

Pain is more common only in later stages or with complications like abscesses. Many people experience no discomfort at all while gums recede, pockets deepen, or bone loss occurs.

Reliable sources like the Cleveland Clinic, Mayo Clinic, American Dental Association (ADA), and CDC emphasize that gum disease is often "usually painless" or has subtle signs that people overlook.

Common Warning Signs (Even Without Pain).

Look for these instead of relying on discomfort:

Bleeding gums (when brushing or flossing this is a classic early sign).

Red, swollen, or puffy gums.

Persistent bad breath (halitosis).

Receding gums (teeth looking longer).

Loose teeth or changes in bite.

Pus between teeth and gums.

What to Do.

Regular dental check-ups are the best way to catch it early, as dentists measure pocket depths and look for signs you might miss. Early gum disease (gingivitis) is often reversible with better oral hygiene and professional cleanings, while advanced periodontitis requires more intensive treatment to prevent tooth loss.

This is general information, not personalized medical advice. If you're concerned about your gums, see a dentist or periodontist for a proper evaluation they can diagnose it accurately even without symptoms. Good daily brushing, flossing, and routine visits go a long way in prevention.

Q5 Can gum disease get better without treatment?

No, gum disease generally does not get better on its own without treatment, though the earliest stage (gingivitis) can sometimes be reversed with improved oral hygiene alone. More advanced stages (periodontitis) do not resolve spontaneously and typically worsen without professional intervention.

Key Distinctions by Stage.

Gingivitis (early, reversible stage): This involves inflamed, red, swollen gums that may bleed when brushing or flossing, but no bone loss has occurred yet. It is often reversible with diligent home care (brushing twice daily, flossing daily, antibacterial mouthwash) and/or a professional dental cleaning. Many sources note that symptoms can improve within days to weeks of consistent hygiene.

Periodontitis (advanced stage): This develops if gingivitis is left untreated. It involves deeper infection, gum pockets, and bone/tissue loss around the teeth. This damage is irreversible the disease cannot be fully cured or reversed, though it can be managed to slow or stop progression and prevent tooth loss.

Why It Rarely Improves Without Help.

Gum disease is driven by bacterial plaque and tartar buildup. Without removing these (via brushing/flossing or professional scaling), inflammation persists and usually progresses.

Rare case reports mention temporary stabilization or slight improvement in some untreated advanced cases over years, but this is exceptional and not reliable most evidence shows progression leading to tooth loss, pain, and links to systemic issues (e.g., worse diabetes control, heart disease risk).

What Helps (and What Doesn't).

Early action works best: Professional cleaning excellent home hygiene can reverse gingivitis or control mild periodontitis.

Home remedies (e.g., saltwater rinses, oil pulling) may provide temporary symptom relief but are not substitutes for proper care and won't address tartar or deeper infection.

Untreated, it can lead to loose teeth, abscesses, and higher health risks.

See a dentist promptly if you notice bleeding gums, bad breath, receding gums, or loose teeth. Early detection makes a big difference. This is general information based on medical consensus personal advice requires a professional evaluation.

Q6 Are gum diseases rare?

No, gum diseases are not rare they are very common worldwide.

Gum (periodontal) diseases, which include milder forms like gingivitis (inflammation of the gums) and more serious periodontitis (which can damage the supporting bone and tissues), affect a large portion of the global population. They are among the most prevalent chronic oral health conditions.

Key Prevalence Statistics.

United States: About 42% of adults aged 30 and older have some form of periodontitis (mild, moderate, or severe), with roughly 8% having the severe form. This rises to around 60% in adults 65 and older. Men are more affected (50%) than women (35%).

Globally: Periodontal diseases affect 20 50% of the population in many studies. Severe periodontitis alone impacts over 1 billion people worldwide (about 11 12.5% age-standardized prevalence in recent estimates), with hundreds of millions more having milder forms.

Prevalence increases with age, and it's higher in certain groups (e.g., smokers, people with diabetes, lower socioeconomic status).

These figures come from sources like the CDC, NIDCR, WHO, and Global Burden of Disease studies. Gingivitis is even more widespread (often affecting a majority of adults to some degree), while advanced periodontitis is less common but still far from rare.

Why It Feels "Rare" to Some People.

Early stages (like gingivitis) can be subtle symptoms such as bleeding gums during brushing/flossing may be ignored.

Many cases go undiagnosed until they progress.

With good oral hygiene (brushing twice daily, flossing, regular dental visits), it's highly preventable and reversible in early stages.

Bottom line: Gum disease is a major public health issue, not an uncommon condition. Maintaining strong oral hygiene and seeing a dentist regularly is the best way to prevent or manage it. If you have symptoms like persistent bad breath, swollen/bleeding gums, or loose teeth, consult a dental professional.

Q7 Is it normal to lose teeth because of gum disease as you get older?

Yes, losing teeth due to gum disease (periodontitis) becomes more common as people age, but it is not "normal" or inevitable. It is a preventable condition, and many older adults retain most or all of their natural teeth with proper care.

Why It Happens More Often with Age.

Gum disease is the primary culprit for tooth loss in adults. Periodontitis involves inflammation and infection that destroy the gums, bone, and tissues supporting the teeth, eventually causing them to loosen and fall out (or require extraction).

Plaque and tartar buildup over decades, combined with age-related factors like receding gums, reduced saliva, dexterity issues (e.g., arthritis making brushing/flossing harder), medications causing dry mouth, and cumulative wear, increase the risk. It often progresses slowly and painlessly, going unnoticed until advanced stages.

Statistics show it's prevalent: Around 64-70% of U.S. adults 65 have moderate or severe periodontal disease. Seniors 65 average about 21 remaining teeth, with ~13-18% experiencing complete tooth loss (higher in those 75). Rates have declined over decades due to better dental care but remain significant, especially in certain groups (e.g., lower income, smokers, specific ethnic backgrounds).

Tooth loss is not caused by aging itself it's driven by untreated oral health issues that worsen over time.

Prevention and Management.

Good news: Much of this is avoidable or manageable:

Daily habits: Brush twice daily (with fluoride toothpaste), floss, use antimicrobial mouthwash, and stay hydrated.

Professional care: Regular dental cleanings (every 3-6 months for those at risk), early treatment of gingivitis (reversible stage), and addressing issues like dry mouth.

Lifestyle: Quit smoking (a major risk factor), control diabetes or other conditions, and eat a balanced diet.

Modern treatments (scaling/root planing, antibiotics, surgery, or implants/dentures if needed) can halt progression and replace lost teeth.

If you're experiencing symptoms like bleeding gums, bad breath, receding gums, loose teeth, or pain, see a dentist promptly early intervention is key. Regular check-ups are especially important as you age.

In summary, while tooth loss from gum disease is unfortunately common in older age due to cumulative effects, it's largely preventable and not a normal or unavoidable part of getting older. Maintaining oral health supports overall well-being, nutrition, speech, and quality of life. Consult a dentist for personalized advice.

Q8 If gum disease runs in my family, will I definitely get it too?

No, you will not definitely get gum disease even if it runs in your family. A family history increases your risk due to genetic factors, but it is far from a guarantee gum disease (periodontal disease) is multifactorial, involving genetics, bacteria, lifestyle, and other environmental influences.

Genetics and Risk.

Heritability estimates: Studies suggest genetic factors account for roughly 30 50% of the variance in periodontitis risk, with higher heritability for more severe forms. Up to about 30% of people may have some genetic susceptibility.

Specific genes (e.g., those affecting the immune response like interleukin-1/IL-1 variants) can make some individuals more prone to exaggerated inflammation or poorer response to oral bacteria. Aggressive periodontitis, in particular, often has a stronger genetic link and can run in families.

Family history serves as a useful marker of shared genetic *and* environmental risks (e.g., similar habits or bacteria transmission).

However, no single gene or set of genes dooms you to the disease. Many people with a family history never develop significant problems, while others without it do (due to factors like smoking, diabetes, or poor hygiene).

Other Major Risk Factors.

Gum disease is primarily driven by plaque bacteria, but these amplify risk significantly:

Poor oral hygiene (inadequate brushing/flossing).

Smoking/tobacco use (a very strong risk).

Diabetes, stress, certain medications, pregnancy, or conditions like acid reflux.

Diet, alcohol, and overall health.

Genetics may influence *susceptibility* (how your body reacts), but controllable factors often determine whether the disease actually develops or progresses.

What You Can Do to Lower Your Risk.

Even with genetic predisposition, prevention is highly effective:

Brush twice daily, floss daily, and use antimicrobial mouthwash if recommended.

Get regular dental checkups and professional cleanings (more frequent if you have a family history e.g., every 3 4 months).

Quit smoking and manage other health conditions like diabetes.

Maintain a balanced diet low in sugars.

Tell your dentist about your family history so they can monitor you closely (early detection via probing, X-rays, etc., makes treatment much easier).

In short, family history is a signal to be proactive, not a prediction of inevitable disease. Good habits can often overcome genetic risk. Consult a dentist or periodontist for personalized advice they can assess your specific situation.

Q9 Can stress make me more likely to get gum disease?

Yes, stress can make you more likely to develop or worsen gum disease (gingivitis and periodontitis). This connection is supported by scientific literature, including reviews and studies linking psychological stress to increased risk, severity, and poorer treatment outcomes for periodontal disease.

How Stress Contributes to Gum Disease.

Stress affects gum health through both behavioral and biological mechanisms:

Behavioral changes: Stress often leads to neglected oral hygiene (skipping brushing/flossing), poorer diet (more sugary or soft foods that promote plaque), smoking, or teeth grinding/clenching (bruxism), all of which increase plaque buildup and inflammation.

Biological effects: Chronic stress activates the body's stress response, raising cortisol and other hormones. This can:

Weaken the immune system, making it harder to fight oral bacteria.

Promote systemic and local inflammation in the gums.

Alter saliva production and gum tissue response, allowing plaque bacteria to cause more damage.

Impair wound healing and increase susceptibility to infections.

The primary cause of gum disease is bacterial plaque, but stress acts as a risk factor or risk indicator that amplifies vulnerability when plaque is present. Evidence from animal studies, human cohorts, cross-sectional research, and literature reviews consistently shows positive associations.

Evidence Summary.

Reviews (e.g., 2018 and 2023) conclude that chronic stress negatively impacts the occurrence, progression, and treatment response of periodontal disease via neuroimmunoendocrine pathways.

Organizations like the American Academy of Periodontology note that stress makes it harder for the body to fight periodontal infections.

Elevated cortisol is repeatedly linked to higher inflammation, deeper gum pockets, and greater tissue destruction.

Not everyone under stress will get gum disease genetics, overall health, oral hygiene, and other factors (like smoking or diabetes) play big roles but chronic or high stress clearly raises the odds.

What You Can Do.

Maintain excellent oral hygiene: Brush twice daily, floss, and use an antimicrobial mouthwash even when stressed.

Manage stress: Techniques like exercise, meditation, sleep, or professional help (therapy, counseling) can lower cortisol and support better habits.

See a dentist regularly: Professional cleanings and check-ups catch issues early. Mention stress levels if relevant.

Lifestyle support: Eat a balanced diet, avoid tobacco, and stay hydrated.

If you notice signs like bleeding gums, swelling, bad breath, or recession, consult a dentist or periodontist promptly. Managing stress benefits both your oral and overall health.

Q10 Do gum diseases only affect the mouth and teeth?

No, gum diseases (primarily gingivitis and periodontitis) do not only affect the mouth and teeth. While they originate in the oral cavity causing inflammation, infection of the gums, and potential damage to the bone and tissues supporting the teeth they are linked to broader health issues throughout the body.

How Gum Disease Can Have Systemic Effects.

Gum disease involves chronic bacterial infection and inflammation. Bacteria and inflammatory substances can enter the bloodstream through damaged gum tissue, potentially contributing to problems elsewhere. Untreated periodontitis can also strain the immune system and exacerbate existing conditions.

Key Associated Health Risks.

Research shows associations (though not always proven direct causation) with several conditions:

Heart disease and cardiovascular issues: Periodontal disease is linked to higher risks of heart attack, stroke, atherosclerosis (plaque in arteries), atrial fibrillation, and other events. Inflammation and bacteria may damage blood vessels.

Diabetes: There's a bidirectional relationship diabetes increases gum disease risk, and gum disease can make blood sugar control harder, worsening diabetic complications.

Respiratory diseases: Bacteria from the mouth can be aspirated into the lungs, contributing to pneumonia, COPD, or other infections.

Pregnancy complications: Links to preterm birth, low birth weight, and other issues.

Other associations: Rheumatoid arthritis, Alzheimer's/dementia, certain cancers (e.g., pancreatic, kidney), chronic kidney disease, osteoporosis, and more. Inflammation plays a central role.

These links are supported by observational studies, meta-analyses, and expert statements (e.g., from the American Heart Association, CDC, and Mayo Clinic), though more research is needed on exact mechanisms and causality in some cases.

Bottom Line.

Maintaining good oral hygiene (brushing, flossing, regular dental visits) and treating gum disease promptly can help protect not just your smile but your overall health. If you have symptoms like bleeding gums, persistent bad breath, or loose teeth or risk factors like smoking or diabetes see a dentist or periodontist. They can assess and manage it effectively. Consult a healthcare professional for personalized advice.

Q11 Will a hard-bristled toothbrush clean my teeth better?

No, a hard-bristled toothbrush will not clean your teeth better overall and it can actually do more harm than good.

Why Soft Bristles Are Recommended.

Effective cleaning without damage: Soft bristles are flexible enough to bend and reach around teeth, gums, and crevices, removing plaque effectively when you use proper technique (gentle pressure, 2 minutes twice a day). They clean just as well as (or better than) harder bristles for most people because they adapt to tooth surfaces.

ADA consensus: The American Dental Association explicitly recommends soft-bristled toothbrushes. They minimize the risk of gum abrasion, enamel wear, and recession while still being effective at biofilm (plaque) removal. Medium or hard bristles increase risks without providing meaningful extra cleaning benefits for daily use.

Risks of Hard Bristles.

Hard (or extra-firm) bristles can:

Wear down tooth enamel over time, leading to sensitivity and higher cavity risk.

Cause gum recession and irritation (or bleeding), exposing tooth roots.

Lead to long-term issues like increased sensitivity, aesthetic problems, or the need for more dental work.

Many people assume "harder cleaner," but studies and dental experts show that brushing technique (angle, duration, and gentleness) matters far more than bristle stiffness. Aggressive brushing with hard bristles often backfires.

When Might Hard Bristles Be Okay?

Very rarely for occasional use like cleaning dentures or heavy stains (not natural teeth).

Even then, most dentists advise against them for regular brushing. Medium bristles are a middle ground but still riskier than soft for daily use.

Better Tips for Cleaner Teeth.

Choose an ADA-accepted soft-bristled toothbrush (manual or electric).

Replace it every 3 months or when bristles fray.

Use fluoride toothpaste and gentle circular or bass method brushing.

Floss or use interdental cleaners daily.

See your dentist regularly.

If you have specific concerns (e.g., heavy plaque, sensitive gums, or braces), ask your dentist they can recommend the best option for your situation. Soft bristles good habits win for long-term oral health.

Q12 Should I stop brushing my teeth for a few days if my gums are bleeding?

No, you should not stop brushing your teeth. Doing so would likely make the problem worse by allowing plaque to build up, which is a primary cause of bleeding gums.

Bleeding gums when brushing or flossing is a common symptom of gingivitis (early gum disease), caused by plaque accumulation irritating the gum tissue. Other possible causes include brushing too hard, a new flossing routine, certain medications, vitamin deficiencies, hormonal changes, or more serious conditions like periodontitis.

What to do instead:

Keep brushing gently at least twice a day with a soft-bristled toothbrush using light circular motions (not back-and-forth scrubbing). This helps remove plaque without further irritating the gums.

Floss daily (gently) to clean between teeth.

Rinse with warm saltwater or an antibacterial mouthwash to soothe inflammation.

Maintain consistent oral hygiene bleeding often improves within 1 2 weeks of better habits.

If bleeding persists beyond 1 2 weeks, worsens, or comes with pain, swelling, receding gums, bad breath, or loose teeth, see a dentist promptly. They can diagnose the issue (e.g., professional cleaning for plaque/tartar) and rule out other causes. Ignoring it can lead to more serious gum disease and tooth loss.

This is general information based on common dental advice it's not a substitute for personalized professional care from a dentist or doctor.

Q13 Do I need to brush my teeth after every meal to keep my gums healthy?

No, you don't need to brush your teeth after every meal to keep your gums healthy. The standard, evidence-based recommendation from the American Dental Association (ADA) and other experts is to brush twice a day with fluoride toothpaste for about two minutes each time.

Why Twice a Day Is Usually Enough.

Brushing frequency studies show that twice-daily brushing (especially with fluoride toothpaste) is optimal for reducing plaque, preventing cavities, and lowering the risk of gum disease (gingivitis or periodontitis) compared to brushing less often. More frequent brushing doesn't provide major additional benefits for most people and can sometimes cause issues.

Plaque (a sticky bacterial film) builds up over time and can inflame gums if not removed. Twice-daily brushing, combined with daily flossing or interdental cleaning, disrupts this effectively.

When Brushing After Meals Can Be Problematic.

Brushing immediately after eating or drinking, especially acidic foods/drinks (e.g., citrus, soda, wine, tomatoes), can harm your enamel. Acids temporarily soften the enamel, and brushing right away can wear it away. Wait 30 60 minutes afterward so saliva can neutralize the acids and reharden the enamel.

Some sources note that brushing after meals can help remove food particles and plaque before it hardens into tartar, but this isn't required if you're already following a solid twice-daily routine.

For people with braces, dry mouth, or active gum disease, more frequent brushing (after meals) may be advised by a dentist.

Better Routine for Gum Health.

Brush twice daily once in the morning and once before bed (the nighttime one is especially important as saliva flow decreases while you sleep).

Floss or use interdental cleaners once a day this is crucial for removing plaque between teeth and along the gumline, where brushes can't reach.

Use proper technique Soft-bristled brush at a 45-degree angle to the gums, gentle short strokes. Don't scrub hard.

Additional tips:

Rinse with water or chew sugarless gum after meals if you can't brush.

Use antimicrobial mouthwash if recommended.

See a dentist regularly (typically every 6 months) for cleanings and checkups.

If you have signs of gum issues (bleeding, swelling, bad breath, receding gums), consult a dentist or periodontist they might recommend a more personalized routine. Overall, consistency with the basics (twice-daily brushing + daily flossing) is far more important than brushing after every single meal.

Q14 Even if I brush my teeth regularly, do I still need to get my gums checked?

Yes, you should still get your gums checked regularly by a dentist or dental hygienist, even if you brush your teeth diligently. Brushing is essential but not enough on its own to fully prevent or detect gum issues.

Why Brushing Isn't Sufficient.

Plaque and tartar buildup: Brushing removes a lot of plaque (the sticky bacterial film on teeth), but it often misses areas between teeth, along the gumline, and under the gums. Leftover plaque hardens into tartar (calculus), which brushing can't remove. Tartar irritates gums and promotes bacterial growth leading to inflammation.

Early detection: Gum disease (gingivitis or periodontitis) can start subtly with little to no pain or obvious symptoms. A professional can spot early signs like gum inflammation, recession, pockets, or bleeding that you might overlook.

Professional cleaning: Dentists/hygienists use specialized tools for deep cleaning (scaling) below the gumline, which home care can't replicate. This helps prevent progression to more serious problems like tooth loss.

Recommended Frequency.

Most people benefit from dental check-ups and cleanings every 6 months. Some may need more frequent visits if they have risk factors (e.g., smoking, diabetes, history of gum disease, or higher plaque buildup).

Additional Benefits.

Overall health links: Gum disease is associated with conditions like heart disease, diabetes complications, and more, so maintaining gum health supports your body broadly.

Catches other issues: Exams also screen for cavities, oral cancer, and other problems early, when they're easier (and cheaper) to treat.

Best Practices at Home.

Combine brushing (twice daily for 2 minutes) with daily flossing or interdental cleaning, and a healthy diet. This reduces risk significantly, but professional care completes the picture.

Consult your dentist for personalized advice they can assess your specific situation and recommend the right schedule. Regular visits are a key part of preventive care that helps keep your teeth and gums healthy long-term.

Q15 Is brushing my teeth once a day enough to keep my mouth healthy?

No, brushing your teeth once a day is generally not enough for optimal oral health. The overwhelming recommendation from major dental organizations, including the American Dental Association (ADA), is to brush twice a day (typically morning and night) for at least two minutes each time using fluoride toothpaste and a soft-bristled toothbrush.

Why Twice a Day?

Plaque and bacteria buildup: Every time you eat or drink (especially anything with sugars or carbs), bacteria in your mouth form a sticky film called plaque. This produces acids that attack tooth enamel and irritate gums. Brushing once a day allows plaque to accumulate for ~24 hours, increasing risks over time.

Nighttime is critical: Saliva production drops while you sleep, giving bacteria more opportunity to thrive. Brushing before bed removes food particles and plaque from the day, while morning brushing clears overnight buildup.

Evidence-based benefits: Twice-daily brushing (with fluoride) is linked to lower risks of cavities (caries), gum disease (gingivitis/periodontitis), and other issues compared to less frequent brushing.

What Happens If You Only Brush Once a Day?

Habitually doing so raises the likelihood of:

Cavities and tooth decay.

Gum inflammation or disease (which can lead to recession or tooth loss).

Tartar buildup (hardened plaque that requires professional cleaning).

Bad breath (halitosis).

In more severe cases, need for fillings, root canals, or other treatments.

One thorough brushing per day is better than none, and some research (e.g., older reviews) suggests meticulous once-daily brushing can prevent major issues in healthy mouths by disrupting bacterial colonies before they mature (they take ~24 48 hours). However, this is not the standard advice twice daily provides a stronger safety margin for most people.

Full Routine for a Healthy Mouth.

Brushing alone isn't sufficient. The ADA and others recommend:

Floss or clean between teeth once a day (toothbrush bristles can't reach everywhere).

Use fluoride toothpaste.

Consider an electric toothbrush for better plaque removal.

Replace your toothbrush every 3 4 months.

Visit a dentist regularly (e.g., every 6 months) for cleanings and checkups.

Limit sugary/acidic foods/drinks and rinse with water after them.

If you have specific conditions (e.g., braces, dry mouth, or gum issues), your dentist may give personalized advice. Brushing once is a step in the right direction if that's all you can manage consistently, but aiming for twice daily is the best way to keep your teeth and gums healthy long-term. If you're concerned about your current habits, talk to a dental professional.

Q16 Can flossing cause gum recession?

Yes, flossing *can* contribute to gum recession, but only if done incorrectly. Proper flossing is actually one of the best ways to *prevent* gum recession by removing plaque and food particles that lead to gum disease (gingivitis and periodontitis), a major cause of receding gums.

Why Improper Flossing Causes Problems.

Gum recession happens when gum tissue pulls away from the teeth, exposing roots and increasing risks like sensitivity, decay, and tooth loss. Aggressive or wrong techniques irritate or traumatize delicate gum tissue:

Snapping or forcing the floss hard into the gums instead of gently sliding it.

Using too much pressure or sawing motion that rubs against the gums.

Hitting the gums directly rather than hugging the sides of the teeth in a C-shape.

Flossing too aggressively or (rarely) too frequently, which can cause repeated irritation.

Dentists consistently note that these mistakes can lead to bleeding, inflammation, and eventual recession over time.

Proper Flossing Helps Prevent Recession.

Floss once a day (daily is ideal; more isn't necessarily better).

Use a gentle technique: Break off 18 24 inches of floss, wrap it around your fingers, slide it gently between teeth with a sawing motion, then curve it into a C-shape around each tooth and move it up and down along the side (not jamming into the gum).

Avoid snapping it down forcefully.

Other common causes of gum recession include aggressive brushing (especially with hard bristles), plaque/tartar buildup from poor hygiene, genetics, smoking, grinding teeth, and certain medical conditions or medications.

Bottom Line and Tips.

Don't stop flossing skipping it is far more likely to cause gum disease and recession than flossing properly.

If your gums bleed when you floss, it’s often a sign of inflammation (not necessarily damage from flossing) and usually improves with consistent gentle technique. Persistent issues warrant seeing a dentist.

Consider alternatives like water flossers if traditional floss irritates you they can be gentler for some people.

See your dentist regularly for cleanings and checkups; they can assess your technique and catch early signs of recession.

If you're experiencing recession or pain, consult a dental professional for personalized advice rather than self-diagnosing. Good oral hygiene overall is the key protector here.

Q17 Do electric toothbrushes damage the gums more than regular toothbrushes?

No, electric toothbrushes do not damage the gums more than regular (manual) toothbrushes. In fact, high-quality evidence from systematic reviews, long-term studies, and dental organizations indicates that electric toothbrushes are generally at least as safe for gums and often superior in promoting gum health when used correctly.

Key Evidence from Studies.

Cochrane Review (powered vs. manual toothbrushing): Powered (electric) toothbrushes reduce plaque more effectively (11 21% reduction) and gingivitis (6 11% reduction) compared to manual brushing, both short- and long-term. There was no evidence of greater harm; some trials even showed slightly fewer cases of gingival abrasion with powered brushes.

11-year cohort study: Electric toothbrush users had 22% less gum recession and 18% less tooth decay progression than manual users.

3-year randomized study on pre-existing recession: Neither type worsened recession when used properly; both helped stabilize or reduce it in some cases, with electric brushes sometimes showing advantages.

Other reviews and trials (e.g., oscillating-rotating models) confirm better plaque/gingivitis reductions without increased gum damage. Features like pressure sensors and timers help prevent over-brushing.

Why the Myth Persists.

Damage to gums (recession, abrasion, or sensitivity) usually comes from improper technique, not the brush type:

Applying too much pressure.

Using hard/medium bristles instead of soft.

Aggressive scrubbing or brushing too frequently/long.

Old/worn brush heads.

Electric brushes can sometimes highlight existing issues (e.g., more noticeable bleeding as they remove plaque better near the gumline), but this is generally a sign of improved cleaning rather than harm. Many modern electrics have pressure alerts that stop or alert if you're pressing too hard, which can actually reduce risk compared to manual brushing.

Recommendations from Dental Experts (e.g., ADA).

Both manual and electric toothbrushes are effective if you brush for 2 minutes twice daily with proper technique and soft bristles.

Electric versions often make it easier to achieve better results, especially for people with limited dexterity, children, or those prone to inconsistent brushing.

Look for the ADA Seal of Acceptance for safety and efficacy.

Bottom line: Technique and consistency matter far more than manual vs. electric. If you have concerns like receding gums or sensitivity, consult a dentist they may recommend a specific electric model with gentle modes. Replace brush heads (or manual brushes) every 3 4 months.

Q18 Would using mouthwash alone be enough without brushing my teeth?

No, using mouthwash alone is not enough to maintain good oral hygiene. Brushing your teeth is the essential foundation for cleaning your teeth effectively.

Why Brushing Matters More.

Mechanical removal of plaque and debris: Brushing physically scrubs away plaque (a sticky film of bacteria), food particles, and buildup on tooth surfaces and along the gumline. Mouthwash cannot do this effectively it mostly rinses and kills some bacteria but leaves plaque behind. Over time, this can lead to cavities, gum disease (gingivitis or periodontitis), tartar buildup, bad breath that returns quickly, and even tooth loss.

Expert consensus: Organizations like the American Dental Association (ADA) and sources like the NHS emphasize that brushing twice a day for two minutes with fluoride toothpaste, plus flossing, forms the core of oral care. Mouthwash is only a helpful *addition*, not a substitute.

What Mouthwash Does Well (and Its Limits).

Mouthwash can:

Reach areas a toothbrush might miss (e.g., between teeth, tongue, cheeks) and reduce bacteria.

Provide fluoride to help strengthen enamel and prevent decay (in fluoride rinses).

Freshen breath temporarily and reduce gingivitis risk in some cases.

However, it doesn't replace the scrubbing action of brushing. Using it alone might mask odors or give a temporary clean feeling, but it won't prevent long-term problems.

Recommended Routine.

Brush twice daily (morning and night) for at least 2 minutes with fluoride toothpaste.

Floss once a day to clean between teeth.

Mouthwash (optional, ideally fluoride or antimicrobial) as an extra step many experts suggest using it after brushing and flossing (check product labels and avoid right after brushing if it might wash away toothpaste fluoride).

Visit a dentist regularly for checkups and cleanings.

If you're short on time, prioritize brushing over mouthwash. For personalized advice (e.g., if you have specific conditions like dry mouth or high cavity risk), consult a dentist. Consistent brushing flossing will give you far better results than relying on rinses alone.

Q19 After I quit smoking, my gums started bleeding. Is that normal?

Yes, bleeding gums after quitting smoking is quite common and often a normal part of the healing process.

Smoking constricts blood vessels and reduces blood flow to the gums, which can mask underlying issues like inflammation or early gum disease (gingivitis or periodontitis). When you quit, circulation improves rapidly often within days to weeks as nicotine leaves your system. This brings more oxygen, nutrients, and immune cells to the tissues, which can temporarily cause or reveal bleeding, swelling, soreness, or sensitivity as the gums "wake up" and start repairing.

Why it happens.

Unmasking effect: Smokers often don't notice bleeding because reduced blood flow hides symptoms. Once you stop, the gums react more normally to plaque or minor irritation.

Healing response: Improved blood supply helps fight bacteria but can make tissues more reactive short-term.

This is frequently described as a positive sign that your body is recovering.

How long does it last?

It often peaks in the first 1 4 weeks and then improves as gums stabilize. Many people see noticeable benefits in gum health within a few weeks to a couple of months.

When to see a dentist or doctor.

While mild, occasional bleeding is usually normal, consult a dental professional promptly (ideally soon after quitting) for these reasons:

Heavy or persistent bleeding beyond a few weeks.

Severe pain, swelling that worsens, pus, bad taste, loose teeth, or receding gums.

Any other concerning symptoms.

A dentist can check for gum disease, provide a professional cleaning, and guide you on better oral hygiene (brushing, flossing, etc.). Quitting smoking greatly improves treatment outcomes for gum issues and lowers long-term risks of periodontitis, tooth loss, and oral cancer.

Tips while healing.

Brush gently with a soft toothbrush and floss daily (bleeding may happen at first but should decrease).

Use an antimicrobial mouthwash if recommended.

Stay hydrated and maintain good overall nutrition.

Avoid other irritants if possible.

Congratulations on quitting smoking that's one of the best things you can do for your oral and overall health! Improvements continue over time. This isn't medical advice, so professional evaluation is the safest next step for personalized reassurance.

Q20 Can natural products effectively keep my gums healthy?

Yes, certain natural products can help support gum health as adjuncts to proper oral hygiene, but they are not a substitute for brushing, flossing, professional dental care, or proven treatments for conditions like gingivitis or periodontitis.

Gum disease (gingivitis and periodontitis) is primarily driven by plaque buildup from bacteria. The most effective prevention and management comes from mechanical removal of plaque through brushing twice daily, daily flossing, and regular dental cleanings. Natural options may offer additional anti-inflammatory, antimicrobial, or soothing benefits, backed by varying levels of evidence from studies (often small or short-term).

Promising Natural Approaches with Some Evidence.

Saltwater rinses: Simple, effective for reducing inflammation and bacteria. Mix 1/2 tsp salt in warm water and rinse 2 3 times daily. Comparable to some mouthwashes for plaque reduction in studies.

Oil pulling (e.g., coconut or sesame oil): Swish 1 tbsp of oil in your mouth for 10 20 minutes daily, then spit it out. It can reduce plaque, bacteria counts, and gingival inflammation in some trials, with effects sometimes comparable to chlorhexidine mouthwash for mild cases. Evidence is promising but mixed/limited; it doesn't reverse advanced disease or receding gums.

Aloe vera: As a gel or mouth rinse, it shows anti-plaque and anti-gingivitis effects similar to chlorhexidine in some research, and may aid healing.

Herbal options:

Lemongrass oil mouthwash: Antimicrobial properties; studies suggest it reduces plaque and gingivitis effectively.

Tea tree oil, clove oil, or turmeric (curcumin): Antibacterial and anti-inflammatory. Turmeric paste or rinses may soothe gums; clove oil for temporary pain relief. Evidence is supportive but often from smaller studies.

Green tea: Antioxidants (catechins) linked to lower gum bleeding and better gum health with regular consumption.

Other supports: Vitamin C-rich foods (for connective tissue), probiotics (some evidence as adjuncts), and baking soda (mild abrasive/antibacterial).

Herbal toothpastes or mouthwashes sometimes perform comparably to conventional ones in short-term plaque/gingivitis reduction, per reviews.

Important Limitations and Cautions.

Evidence quality: Many studies are small, short-term, or lack strong controls. Results vary, and natural products are generally better for mild issues or maintenance than treating moderate/severe gum disease.

Not a cure-all: They won't remove tartar (calculus) or reverse significant bone loss. Delaying professional care can worsen problems.

Safety: Dilute essential oils properly (they can irritate or be toxic if swallowed undiluted). Test for allergies. Consult a dentist before using, especially if you have existing conditions, are pregnant, or on medications.

ADA/dental guidelines: Focus on evidence-based basics like fluoride toothpaste, interdental cleaning, and professional visits. Sugar-free gum (especially with xylitol) can help, but "natural" isn't automatically better.

Bottom line: Incorporate supportive natural products into a solid routine (brush, floss, diet low in sugars, regular checkups) for potential benefits. See a dentist or periodontist for personalized advice, especially if you notice bleeding, swelling, bad breath, or receding gums these need professional evaluation. Early intervention is key to keeping gums healthy long-term.

Q21 Can getting a dental scaling damage my teeth?

No, professional dental scaling does not damage healthy teeth when performed correctly by a trained dentist or hygienist. It is a safe, standard procedure designed to protect your oral health by removing plaque, tartar (calculus), and bacteria that can lead to cavities, gum disease, and tooth loss.

Why Scaling Is Safe for Teeth.

Enamel protection: Tooth enamel is the hardest substance in the human body. Scaling instruments (hand scalers or ultrasonic devices) are specifically designed to dislodge tartar from the tooth surface without significantly affecting the enamel. Any minor surface effects are negligible and repaired naturally through saliva and remineralization.

Benefits outweigh risks: Scaling prevents more serious damage from untreated tartar buildup, which harbors bacteria that erode enamel and damage gums/bone over time. Regular cleanings (often combined with polishing) actually help preserve teeth.

Supported by dental authorities: Organizations like the American Dental Association (ADA) endorse scaling and root planing as an effective treatment for gum disease (periodontitis), with clear benefits for patients.

Potential Temporary Side Effects (Not Damage).

Tooth sensitivity: Common after scaling, especially if there's significant tartar removal or gum recession exposing roots (which are covered by softer cementum). This usually lasts a few days to a week and can be managed with desensitizing toothpaste.

Gum tenderness or bleeding: Gums may feel sore or bleed slightly right after, as inflammation subsides.

Appearance changes: Teeth may look or feel "different" (smoother or with small gaps visible) because tartar that was filling spaces is göne this is not damage, just a return to a cleaner state.

Rare Cases Where Caution Applies.

Pre-existing conditions: Ultrasonic scaling might worsen damage on teeth with existing enamel cracks, early caries, or certain restorations (fillings). A good dentist will assess this first.

Aggressive or improper technique: Overly forceful scaling by an inexperienced provider could irritate gums or (very rarely) affect root surfaces in cases of recession. Choose a qualified professional to minimize this.

Medical factors: People with certain heart conditions or bleeding risks may need precautions (e.g., antibiotics), but the procedure itself remains safe overall.

Myths vs. Facts.

A common fear is that scaling "scrapes away enamel" or loosens teeth. This is a misconception tartar removal doesn't weaken teeth; the underlying gum disease (if present) is what threatens them. Avoiding scaling can actually lead to worse problems like bone loss and tooth mobility.

Bottom line: Get regular dental cleanings as recommended (typically every 6 months, or more often if you have gum issues). Discuss any concerns like sensitivity or medical history with your dentist beforehand they can tailor the procedure and provide aftercare tips. If you've had bad experiences or specific conditions, a second opinion from a periodontist can help. Maintaining good home hygiene (brushing, flossing, fluoride) complements professional care best.

Q22 Can dental scaling cause gaps between my teeth?

No, dental scaling (also called teeth cleaning or deep cleaning/scaling and root planing) does not cause gaps between your teeth.

It often *reveals* gaps that were already there but hidden by plaque, tartar (calculus), or swollen gums from inflammation or early gum disease. Here's why this happens and what to expect:

Why Gaps Appear After Scaling.

Tartar buildup hides spaces: Plaque hardens into tartar, which can fill or mask natural spaces between teeth and hold them in place (acting almost like a "splint"). Removing it during scaling makes those pre-existing spaces visible.

Gums heal and shrink: Inflamed or swollen gums (from gingivitis or periodontitis) can cover underlying gaps. Deep cleaning removes bacteria and tartar, allowing the gums to reduce in size and reattach properly. This can make spaces more noticeable temporarily as healing occurs.

Underlying issues are exposed: In cases of gum disease, there may already be some bone loss or recession. The cleaning doesn't create this it treats the cause and stops further damage, but the existing effects (like recession) become apparent.

This is a common and normal experience reported by many patients. The gaps are usually not "new" but were masked before.

What to Expect After Scaling.

Teeth may feel temporarily looser or more sensitive as the tartar "support" is gone and gums heal.

Any visible gaps often improve or stabilize over a few weeks as gums reattach and inflammation subsides.

Good oral hygiene afterward (brushing, flossing, regular check-ups) helps prevent worsening and supports healing.

When to See Your Dentist.

While usually harmless, consult your dentist or hygienist if:

Gaps are large, persistent, or accompanied by pain, significant looseness, or worsening symptoms.

You have advanced periodontal disease (they may recommend further treatment).

You're concerned about appearance or function options like bonding, veneers, or orthodontics can address cosmetic gaps if needed.

Scaling is a beneficial procedure that prevents more serious issues like tooth loss from untreated gum disease. If you're worried about your specific situation, your dental professional can examine your teeth and explain what's happening in your mouth. Regular cleanings are one of the best ways to maintain healthy teeth and gums long-term.

Q23 Do all gum diseases require the same treatment?

No, not all gum diseases require the same treatment. Treatments vary significantly depending on the type, stage, and severity of the condition, as well as individual factors like overall health, smoking status, and prior treatments.

Main Types of Gum Disease.

Gum disease (periodontal disease) primarily includes two main forms on a spectrum:

Gingivitis: The mildest, earliest stage. It involves inflammation of the gums (redness, swelling, bleeding) caused by plaque buildup, but no bone or tissue loss has occurred yet. This stage is reversible with proper care.

Periodontitis: Develops if gingivitis is untreated. Bacteria affect deeper tissues, leading to gum recession, pocket formation, bone loss, and potential tooth loosening or loss. This is not fully reversible (damage to bone/supporting structures can't be undone), but it can be managed to prevent worsening.

There are further stages of periodontitis (mild, moderate, advanced/severe), with increasing damage.

How Treatments Differ.

Treatment is tailored to the stage and progression:

For gingivitis (early/reversible):

Professional dental cleaning (prophylaxis) to remove plaque and tartar.

Improved daily oral hygiene: brushing twice daily, flossing, and possibly antibacterial mouth rinses.

Often fully resolves without further intervention if addressed promptly.

For periodontitis (deeper involvement):

Non-surgical options (often first-line for mild-moderate cases): Scaling and root planing ("deep cleaning") to remove plaque/tartar below the gumline, plus antibiotics (oral, gels, or rinses) to control infection.

Surgical options (for moderate to advanced cases): Flap surgery, pocket reduction, bone grafts, gum grafts, or regenerative procedures to address bone/tissue loss. Laser therapies (e.g., LANAP) may be used in some cases.

Ongoing periodontal maintenance (more frequent cleanings, e.g., every 3-4 months).

Home care and lifestyle changes (quitting smoking, managing conditions like diabetes) are essential across all stages but not sufficient alone for advanced disease.

Why Uniform Treatment Doesn't Work.

Early intervention can reverse or halt progression cheaply and non-invasively.

Advanced cases require more aggressive approaches to control infection and prevent tooth loss or systemic health links (e.g., to heart disease).

Factors like pocket depth, bone loss (assessed via exams and X-rays), and patient response determine the plan.

See a dentist or periodontist promptly if you notice symptoms like bleeding gums, bad breath, receding gums, or loose teeth. Early diagnosis leads to simpler, more effective treatment. Regular checkups and good hygiene are the best prevention. This is general information professional evaluation is needed for personalized advice.

Q24 Is gum treatment painful?

No, gum treatment is generally not very painful, especially with modern techniques, though some discomfort is common.

During the Procedure.

Most common gum treatments (like scaling and root planing for gum disease) are done with local anesthesia, so patients typically feel little to no sharp pain often just pressure, vibration, or mild scraping sensations.

More advanced options like laser therapy are often even more comfortable, with minimal or no anesthesia needed in some cases and reduced bleeding.

Surgical procedures (e.g., pocket reduction or gum grafts) involve anesthesia too, so the surgery itself is painless, though they are more involved.

Pain levels vary based on:

Your personal tolerance.

The severity of gum disease (inflamed gums are more sensitive).

The specific treatment.

Studies and patient reports show most people experience low pain during non-surgical treatments, with only a minority reporting moderate discomfort.

After the Procedure.

Mild soreness, tenderness, or sensitivity (to hot/cold or pressure) is common for a few days to a week as gums heal.

Over-the-counter pain relievers, soft foods, and good oral care usually manage this well. Some swelling or minor bleeding can occur but typically resolves quickly.

Laser or minimally invasive methods often lead to faster recovery and less post-op discomfort.

Why It Matters.

Untreated gum disease can become more painful over time (e.g., causing pain while chewing or abscesses) and lead to tooth loss. Early treatment prevents worse issues and is usually much more comfortable.

Talk to your dentist or periodontist they can tailor the approach (e.g., sedation for anxiety) and explain exactly what to expect for your situation. Many patients say the treatment is far less scary than they anticipated once they go through it. If you're concerned about pain, ask about numbing options or gentler techniques upfront.

Q25 Can gum surgery cause tooth loss?

Yes, but it's uncommon and usually not a direct result of the surgery itself. Gum surgery (such as gum grafts, flap surgery, or osseous surgery for periodontal disease) is typically done to *treat* or *prevent* tooth loss by addressing gum recession, bone loss, and infection. However, like any surgery, it carries risks that could indirectly contribute to complications, including potential tooth issues in rare cases.

Why Surgery Is Usually Protective.

Gum disease (periodontitis) is a leading cause of tooth loss in adults. It leads to gum recession, bone loss around teeth, loosening, and eventual loss if untreated.

Procedures like gum grafting cover exposed roots, reduce sensitivity, prevent decay, and stabilize teeth. Bone grafts or regenerative techniques can rebuild support structures.

Success rates are generally high when performed by a qualified periodontist, and proper aftercare helps preserve teeth long-term.

Potential Risks and Complications.

Complications from gum surgery are uncommon but can include:

Graft failure or rejection: The grafted tissue may not take properly (e.g., due to poor blood supply, infection, smoking, or disturbance like aggressive brushing). This can lead to continued or worsened recession.

Infection, excessive bleeding, swelling, or pain: These can delay healing and, if severe/untreated, potentially affect nearby teeth or bone.

Increased recession or sensitivity in some cases.

Rare indirect tooth loss: If surgery fails and underlying issues (like ongoing gum disease or significant pre-existing bone loss) aren't resolved, teeth may become loose or be lost. Pre-existing advanced disease makes outcomes less predictable.

Surgery doesn't typically "cause" healthy teeth to fall out. Tooth loss is far more likely from *not* treating progressive gum disease.

Factors That Increase Risks.

Smoking or tobacco use (impairs healing).

Poor oral hygiene or failure to follow post-op instructions.

Advanced pre-existing bone loss or loose teeth.

Certain health conditions (e.g., diabetes) or medications.

Surgeon experience and technique.

What to Do.

Consult a periodontist for a personalized assessment (X-rays, exam). They can evaluate if surgery is appropriate and discuss success odds for your situation.

Follow aftercare strictly: soft diet, gentle cleaning, medications as prescribed, and avoid disturbing the site.

Maintain excellent long-term hygiene, regular dental visits, and address risk factors (e.g., quit smoking) to maximize benefits.

If you're experiencing symptoms like loose teeth, pain, or recession post-surgery (or considering it), see your dentist or specialist promptly. Early intervention usually yields the best outcomes. This is general information not personalized medical advice.

Q26 Can gum treatment make my teeth feel loose?

Yes, gum treatment can make your teeth feel loose, but this is usually temporary and a normal part of the healing process.

Why This Happens.

Gum treatments, especially scaling and root planing (a common deep cleaning for gum disease/periodontitis), remove plaque, tartar (calculus), and bacteria from below the gumline. Before treatment:

Buildup and inflammation can create a false sense of stability by "propping up" the tooth.

After removal, teeth may feel wigglier because the true condition of the supporting bone and ligaments is revealed.

This sensation is common and does not mean the treatment caused permanent damage or that teeth will fall out. Instead, it's often a sign that the infection is being addressed so gums can reattach and heal.

What to Expect.

Temporary mobility: Teeth may feel loose for days to weeks (sometimes longer in advanced cases) as inflammation decreases and gums tighten.

Healing phase: With good oral hygiene and follow-up care, many people notice improved stability as the periodontal ligaments and gums recover. In some cases, teeth can tighten up noticeably.

Advanced gum disease: If there's significant bone loss already, full tightening may be limited, but treatment still halts progression and prevents further loosening or tooth loss.

When to Seek Professional Advice.

Contact your dentist or periodontist promptly if:

Looseness worsens significantly, or you have pain, swelling, or pus.

It persists beyond a few weeks without improvement.

You have other symptoms like bleeding gums or bad breath (common with untreated gum disease).

Your provider may recommend splinting (temporarily bonding teeth for support), antibiotics, or further procedures like pocket reduction surgery if needed.

Key Takeaway.

Gum treatment prevents worse loosening and tooth loss by tackling the root cause (periodontal disease, the leading reason for loose adult teeth). The initial "loose" feeling is typically part of getting healthier foundations, not a setback. Always follow post-treatment instructions (soft diet, gentle brushing, rinses) and attend follow-ups for the best outcome. This isn't medical advice consult a dentist for your specific situation.

Q27 Does tartar removal cause more tartar buildup?

No, tartar removal (professional dental scaling or cleaning) does not cause more tartar buildup.

Tartar (dental calculus) is hardened plaque that forms when minerals in saliva combine with bacterial plaque on your teeth. Only a dentist or hygienist can safely remove it brushing and flossing aren't enough once it hardens.

Why the Myth Persists.

Some people notice tartar returning after a cleaning and assume the procedure "caused" faster buildup. In reality:

Tartar always forms from plaque if plaque isn't removed daily. Professional cleaning resets the baseline by eliminating existing deposits, but it doesn't stop the natural process of plaque accumulation.

Teeth may feel "different" afterward (smoother or temporarily sensitive), which can make any new buildup more noticeable.

Without good oral hygiene, new plaque can mineralize into tartar within days to weeks typically starting in 24 72 hours and becoming noticeable in 1 2 weeks or more with poor habits.

What Actually Happens After Removal.

Smoother surfaces help: Scaling and polishing create smoother tooth surfaces, making it harder for new plaque to adhere and slowing future tartar formation compared to leaving rough tartar in place (which acts like a trap for more bacteria).

It breaks the cycle: Untreated tartar provides a rough, porous surface that accelerates more buildup and harbors bacteria, increasing risks of gum disease, cavities, and bad breath. Removal reduces this.

Tartar does not "grow back" in the same spots immediately; new formation depends on your habits, saliva composition, diet, smoking, medications, and genetics.

Evidence and Recommendations.

Reliable dental sources (e.g., Cleveland Clinic, ADA-aligned info) emphasize that regular professional cleanings prevent worse problems rather than causing them. Common myths like scaling weakening teeth, loosening them, or damaging enamel are not supported; the procedure targets deposits without harming healthy structure when done properly.

To minimize future buildup:

Brush twice daily with fluoride toothpaste (consider anti-tartar formulas).

Floss daily.

Use an antiseptic mouthwash.

Get professional cleanings every 6 months (or every 3 4 months if you have gum disease history, heavy tartar, smoking, diabetes, or dry mouth).

Avoid tobacco and limit sugary/acidic foods.

If you have heavy or rapid tartar formation, see a dentist they can check for underlying issues and tailor advice. Self-removal attempts (e.g., scraping) can damage enamel or gums and worsen problems. Professional care is the safe, effective approach.

Q28 Are antibiotics enough to treat gum disease?

No, antibiotics alone are not enough to treat gum disease (gingivitis or periodontitis). They can help control bacterial infection in some cases but do not address the root causes like plaque, tartar (calculus), and deep pockets, which require professional mechanical cleaning and ongoing hygiene.

Why Antibiotics Aren't Sufficient by Themselves.

Gum disease is driven by bacterial biofilm (plaque) that hardens into tartar below the gumline. Antibiotics may kill some bacteria temporarily, but they can't remove the physical buildup or smooth root surfaces for gums to reattach.

Without removing tartar and plaque through scaling and root planing (deep cleaning), the infection often returns or persists. Long-term success relies on professional treatment plus daily oral care.

Over-reliance on antibiotics can contribute to resistance and side effects, and they're not always needed for mild cases.

Standard Treatment for Gum Disease.

According to sources like the Mayo Clinic and dental guidelines:

Professional cleanings Scaling (removing tartar/bacteria) and root planing (smoothing roots) are the foundation for most cases.

Antibiotics as adjuncts (when used):

Topical: Antibiotic gels, chips, or mouth rinses placed in gum pockets (e.g., minocycline, doxycycline) for localized effect.

Oral (systemic): Pills like amoxicillin, metronidazole, or doxycycline for more aggressive or widespread infections, often combined with deep cleaning.

Advanced options if needed: Surgery (e.g., flap surgery, bone grafts) for severe periodontitis.

Maintenance: Excellent home care (brushing twice daily, flossing, antimicrobial rinses) and regular dental visits (every 3 6 months) to prevent recurrence.

Early gingivitis can often be reversed with cleanings and better hygiene alone. Periodontitis (deeper infection affecting bone) usually needs more intensive care.

When to See a Dentist.

See a dentist or periodontist promptly if you have symptoms like bleeding gums, swelling, bad breath, loose teeth, or receding gums. They'll assess severity (often with X-rays and pocket measurements) and create a personalized plan. Self-treating with antibiotics (e.g., leftover pills) is ineffective and risky.

Important note: This is general information based on medical sources. I'm not a doctor consult a dental professional for advice tailored to your situation, as untreated gum disease can lead to tooth loss and has links to other health issues. Good oral hygiene remains the best prevention and long-term solution.

Q29 After gum disease treatment, will I ever need treatment again?

Yes, you will likely need ongoing care and could require additional treatment in the future gum disease (especially periodontitis) is typically a chronic condition that needs lifelong management rather than a one-time cure.

Why Recurrence Is Common.

It's not usually "curable" in the permanent sense: Gingivitis (early, reversible stage) can often be fully reversed with treatment and good hygiene. However, once it progresses to periodontitis (involving bone and tissue loss), the damage is irreversible, and you remain susceptible to flare-ups. Bacteria can recolonize quickly, often within months if maintenance lapses.

Risk factors persist: Plaque and tartar buildup, genetics, smoking, diabetes, poor oral hygiene, certain medications, or other health issues can cause the disease to return even after successful initial treatment. Studies show recurrence risk increases over time without consistent care.

How to Minimize the Need for Repeat Treatment.

The good news is that with proper periodontal maintenance, many people keep the disease stable and avoid major progression or tooth loss. Key strategies include:

Regular professional cleanings: Most patients need periodontal maintenance (deeper cleanings targeting pockets) every 3–4 months initially, rather than standard 6-month checkups. This frequency has been shown to significantly reduce recurrence (e.g., up to 40% lower risk in some studies compared to less frequent visits). Your dentist or periodontist can adjust based on your response.

Excellent daily home care: Brush twice daily, floss or use interdental cleaners daily, and follow any specific instructions (e.g., antimicrobial rinses or special tools).

Lifestyle and risk control: Quit smoking, manage diabetes or other conditions, and maintain a healthy diet.

Monitoring: Regular exams, probing depths, and X-rays help catch issues early before they require extensive retreatment.

What to Expect Long-Term.

Many patients stay stable for years with diligent maintenance.

If recurrence happens, it’s often milder and more manageable if addressed promptly (e.g., another round of scaling/root planing or localized treatment).

Without maintenance, the disease can progress faster than before, leading to more bone loss or tooth issues.

This is general information based on dental guidelines and studies your specific outlook depends on the severity of your original condition, your overall health, and how well you follow the maintenance plan. Consult your dentist or periodontist for personalized advice, as they know your case best. Early and consistent action makes a big difference in keeping your gums healthy long-term.

Q30 Is surgery always necessary for gum treatment?

No, surgery is not always necessary for gum treatment.

Gum treatment (primarily for periodontal or gum disease) ranges from mild gingivitis to advanced periodontitis, and options vary accordingly. Many cases, especially early or moderate ones, can be managed effectively with non-surgical approaches.

Non-Surgical Treatments (Often Sufficient for Mild to Moderate Cases)

Improved oral hygiene: Brushing, flossing, and professional cleanings can reverse early gingivitis.

Scaling and root planing (deep cleaning): Removes plaque and tartar from below the gumline and smooths tooth roots to help gums reattach. This is a common first-line treatment.

Antibiotics or antimicrobials: Topical or oral medications to control infection.

Laser therapy: Sometimes used as a less invasive option alongside scaling.

These methods are often effective, have shorter recovery times, and are less costly than surgery. Many patients achieve good results with ongoing maintenance and good home care, avoiding surgery altogether.

When Surgery Might Be Needed.

Surgery becomes a consideration for advanced periodontitis where non-surgical methods aren't enough, such as:

Deep periodontal pockets that harbor bacteria.

Significant gum recession exposing tooth roots.

Bone loss around teeth.

Persistent infection or loose teeth.

Common surgical options include flap/pocket reduction surgery, gum grafts, bone grafts, or guided tissue regeneration. These aim to reduce pockets, regenerate lost tissue/bone, and prevent further damage or tooth loss.

Key Factors Determining Treatment.

Stage of disease: Gingivitis and early/moderate periodontitis are usually non-surgical; advanced cases with bone involvement often require surgery.

Response to initial treatment: If scaling/root planing doesn't improve pocket depths or inflammation, surgery may follow.

Individual factors: Overall health, smoking, genetics, and compliance with hygiene.

Early detection and prompt treatment (regular dental visits) greatly reduce the chance of needing surgery. Untreated gum disease can progress, leading to tooth loss and potential links to systemic health issues.

Always consult a dentist or periodontist for personalized advice they'll assess your gums (e.g., via pocket measurements and X-rays) and recommend the least invasive effective option. Self-diagnosis or delaying care isn't advisable. This is general information, not medical advice.
