# Supplementary material for: Comparative Evaluation of Large Language Models in Responding to Common Misconceptions Regarding Periodontal Disease and Oral Hygiene
Source: Diagnostics (Basel). 2026 Sep 28;16(19):3158. doi: 10.3390/diagnostics16193158 (PMC13664004; doi:10.3390/diagnostics16193158)
Supplement: Supplementary file 1 [file diagnostics-16-03158-s001.zip › Supplementary File S5 LLMs response stylistic elements removed.zip!/1. LLMs response/Monica AI.pdf]

Q1 Do gum diseases only affect elderly people?

No, gum diseases do not only affect elderly people. While the risk of developing gum disease, such as gingivitis or periodontitis, increases with age, they can affect individuals of all ages, including children and young adults.

Factors Contributing to Gum Disease:

Poor Oral Hygiene: Inadequate brushing and flossing can lead to plaque buildup.

Smoking and Tobacco Use: These habits significantly increase the risk of gum disease.

Genetics: Some individuals may be genetically predisposed to gum disease.

Health Conditions: Conditions like diabetes can increase susceptibility.

Hormonal Changes: Changes during puberty, pregnancy, or menopause can affect gum health.

Medications: Certain medications can reduce saliva flow, increasing the risk of gum disease.

Prevention Tips:

Maintain good oral hygiene by brushing twice a day and flossing daily.

Visit the dentist regularly for check-ups and cleanings.

Avoid tobacco products.

Eat a balanced diet to support oral health.

In summary, gum diseases can affect anyone, regardless of age, and it's important to practice good oral hygiene to prevent them.

Q2 Is it normal if my gums bleed when I brush my teeth?

Bleeding gums when brushing your teeth can be a sign of several issues, and while it might be common, it is not considered normal. Here are some potential causes:

Possible Causes of Bleeding Gums:

Gingivitis: This is an early stage of gum disease caused by plaque buildup. It often leads to inflammation and bleeding.

Periodontitis: If gingivitis is not treated, it can progress to periodontitis, which is more severe and can lead to tooth loss.

Improper Brushing Technique: Brushing too hard or using a hard-bristled toothbrush can irritate the gums.

Hormonal Changes: Factors like pregnancy, menstruation, or menopause can affect gum health.

Nutritional Deficiencies: Lack of vitamin C (scurvy) or vitamin K can lead to bleeding gums.

Medical Conditions: Conditions like diabetes or blood disorders can also contribute to gum bleeding.

Recommendations:

Visit a Dentist: It's important to consult with a dental professional to determine the exact cause and receive appropriate treatment.

Improve Oral Hygiene: Brush gently with a soft-bristled toothbrush and floss daily.

Regular Check-ups: Regular dental visits can help catch and prevent gum disease.

If you experience persistent bleeding, it's best to seek professional advice.

Q3 Can I get gum disease even if I brush my teeth regularly?

Yes, it is possible to develop gum disease even if you brush your teeth regularly. Here are some factors that can contribute to gum disease despite good oral hygiene practices:

Brushing Technique.

If you're not using the correct technique or brushing for the recommended two minutes, plaque may not be effectively removed.

Flossing.

Brushing alone may not remove plaque and food particles from between the teeth. Flossing is essential to clean those areas.

Diet.

A diet high in sugars and carbohydrates can contribute to plaque buildup, increasing the risk of gum disease.

Tobacco Use.

Smoking or using other tobacco products can impair gum health and reduce blood flow to the gums.

Genetics.

Some individuals may be genetically predisposed to gum disease, regardless of their oral hygiene habits.

Hormonal Changes.

Changes in hormone levels (e.g., during pregnancy, menstruation, or menopause) can make gums more sensitive and susceptible to disease.

Medical Conditions.

Conditions such as diabetes, autoimmune diseases, or certain medications can impact gum health.

Regular Dental Visits.

Skipping regular dental check-ups can allow gum disease to progress without detection.

Conclusion.

Maintaining good oral hygiene is crucial, but it's also important to consider these other factors. If you have concerns about gum disease, consult with a dental professional for personalized advice and care.

Q4 Can I have gum disease even if my gums don't hurt?

Yes, it is possible to have gum disease even if your gums don't hurt. Gum disease, or periodontal disease, can progress without noticeable pain or symptoms in its early stages. Here are some key points to consider:

Signs of Gum Disease.

Swollen or Red Gums: Gums may appear inflamed.

Bleeding Gums: Gums may bleed during brushing or flossing.

Receding Gums: Gums may pull away from the teeth.

Bad Breath: Persistent bad breath can be a sign.

Loose Teeth: Advanced gum disease can cause teeth to loosen.

Importance of Regular Check-ups.

Professional Cleanings: Regular dental visits can help identify gum disease early, even if you don't experience pain.

Preventive Care: Good oral hygiene practices can help prevent gum disease.

If you suspect you might have gum disease, it's important to consult with a dentist for an accurate diagnosis and appropriate treatment.

Q5 Can gum disease get better without treatment?

Gum disease, also known as periodontal disease, can sometimes improve on its own, especially in its early stages (gingivitis). Here are some key points to consider:

Early Stage (Gingivitis).

Reversible: Gingivitis is characterized by inflammation of the gums and is often reversible with improved oral hygiene practices, such as regular brushing, flossing, and professional cleanings.

Symptoms: Signs include redness, swelling, and bleeding of the gums during brushing or flossing.

Advanced Stage (Periodontitis).

Not Reversible: If gingivitis progresses to periodontitis, the damage to the gum tissue and bone may not fully heal without professional treatment.

Treatment Needed: Periodontitis requires more intensive treatment, such as scaling and root planing, and sometimes surgical interventions.

Self-Care.

Improvement Possible: Good oral hygiene can help manage symptoms and prevent further progression, but it is crucial to consult a dentist for proper diagnosis and treatment recommendations.

Conclusion.

While early gum disease may improve with better oral care, advanced gum disease typically requires professional treatment to prevent further complications. Regular dental check-ups are essential for maintaining gum health.

Q6 Are gum diseases rare?

Gum diseases, also known as periodontal diseases, are not rare. In fact, they are quite common and affect a significant portion of the population. Here are some key points:

Prevalence.

Mild to Moderate Cases: Many adults experience some form of gum disease, ranging from gingivitis (mild inflammation) to more severe forms like periodontitis.

Statistics: Studies indicate that nearly 50% of adults over the age of 30 have some form of gum disease.

Risk Factors.

Poor Oral Hygiene: Inadequate brushing and flossing can lead to plaque buildup, which is a primary cause of gum disease.

Smoking: Tobacco use significantly increases the risk of gum disease.

Genetics: Family history can play a role in susceptibility.

Health Conditions: Diabetes, certain medications, and hormonal changes can also increase risk.

Prevention.

Regular Dental Check-ups: Routine visits to the dentist for cleanings and check-ups can help catch gum disease early.

Good Oral Hygiene: Brushing twice a day and flossing daily are essential for prevention.

In summary, gum diseases are common and can affect anyone, but they are preventable with proper oral care.

Q7Is it normal to lose teeth because of gum disease as you get older?

Yes, it is relatively common for individuals to experience tooth loss due to gum disease as they age. Here are some key points to consider:

Understanding Gum Disease.

Gingivitis: This is the early stage of gum disease, characterized by inflammation and bleeding of the gums. It can often be reversed with good oral hygiene.

Periodontitis: If gingivitis is not treated, it can progress to periodontitis, which involves deeper gum infection and can lead to the destruction of the supporting bone structure around the teeth.

Factors Contributing to Tooth Loss.

Age: The risk of gum disease increases with age, partly due to cumulative exposure to bacteria and possible decline in overall health.

Oral Hygiene: Poor dental hygiene practices can exacerbate gum disease, leading to more severe consequences.

Health Conditions: Certain health issues, like diabetes, can increase the risk of gum disease and tooth loss.

Smoking: Tobacco use is a significant risk factor for the development of gum disease.

Prevention and Management.

Regular Dental Check-ups: Routine visits to the dentist can help catch gum disease early and prevent tooth loss.

Good Oral Hygiene: Brushing twice a day, flossing, and using mouthwash can help maintain gum health.

Healthy Lifestyle: A balanced diet and avoiding tobacco can reduce the risk of gum disease.

If you have concerns about gum disease or tooth loss, it's important to consult with a dental professional for personalized advice and treatment options.

Q8If gum disease runs in my family, will I definitely get it too?

While having a family history of gum disease can increase your risk, it does not guarantee that you will develop it. Here are some factors to consider:

Genetic Factors.

Hereditary Influence: Genetics can play a role in your susceptibility to gum disease. If your family members have experienced it, you may have a higher risk.

Lifestyle and Environmental Factors.

Oral Hygiene: Poor oral hygiene practices can lead to gum disease. Regular brushing and flossing can help mitigate risk.

Diet: A diet high in sugar and low in nutrients can contribute to gum disease.

Smoking: Tobacco use is a significant risk factor for gum disease.

Medical Conditions: Conditions like diabetes can increase the risk.

Preventive Measures.

Regular Dental Check-ups: Routine visits to the dentist can help detect early signs of gum disease.

Healthy Lifestyle: Maintaining a balanced diet, avoiding tobacco, and practicing good oral hygiene can reduce your risk.

Conclusion.

While family history is a factor, it’s essential to focus on preventive measures to maintain your oral health. Regular dental care and healthy habits can significantly lower your risk of developing gum disease.

Q9Can stress make me more likely to get gum disease?

Yes, stress can indeed make you more susceptible to gum disease. Here’s how:

How Stress Affects Gum Health.

Immune System Suppression: Stress can weaken your immune system, making it harder for your body to fight off infections, including those that affect your gums.

Hormonal Changes: Stress triggers the release of hormones like cortisol, which can lead to inflammation and affect your body's ability to maintain healthy gums.

Neglecting Oral Hygiene: During stressful times, people may neglect their oral hygiene routines, leading to plaque buildup and an increased risk of gum disease.

Unhealthy Habits: Stress may lead to unhealthy behaviors such as smoking, poor diet, or increased alcohol consumption, all of which can contribute to gum disease.

Bruxism: Stress can cause teeth grinding (bruxism), which can lead to gum recession and other dental issues.

Conclusion.

To maintain good gum health, it's important to manage stress effectively, practice good oral hygiene, and visit your dentist regularly. If you're experiencing significant stress, consider speaking with a healthcare professional for support.

Q10 Do gum diseases only affect the mouth and teeth?

Gum diseases, primarily known as periodontal diseases, primarily affect the gums and the supporting structures of the teeth. However, their impact can extend beyond the mouth and teeth in several ways:

Local Effects.

Gum Inflammation: Gingivitis is the early stage of gum disease, causing redness and swelling.

Periodontitis: This advanced stage can lead to tooth loss and bone damage.

Systemic Effects.

Gum diseases can also have implications for overall health, including:

Cardiovascular Disease: There is a link between periodontal disease and heart disease. Inflammation from gum disease may contribute to heart problems.

Diabetes: Gum disease can make it more difficult to control blood sugar levels, and diabetes can increase the risk of gum disease.

Respiratory Issues: Bacteria from gum disease can be inhaled into the lungs, potentially causing respiratory infections.

Pregnancy Complications: Pregnant women with gum disease may be at higher risk for preterm birth and low birth weight.

Conclusion.

While gum diseases primarily affect oral health, they can have significant effects on overall health, highlighting the importance of maintaining good oral hygiene and regular dental check-ups.

Q11 Will a hard-bristled toothbrush clean my teeth better?

Using a hard-bristled toothbrush can effectively remove plaque and debris from your teeth, but it comes with some important considerations:

Pros of Hard-Bristled Toothbrushes.

Effective Cleaning: They can be more effective at removing stubborn plaque and food particles.

Stain Removal: May help in removing surface stains on teeth.

Cons of Hard-Bristled Toothbrushes.

Gum Damage: They can cause gum irritation, recession, or damage due to their stiffness.

Enamel Wear: Over time, they may contribute to enamel erosion, increasing the risk of tooth sensitivity and cavities.

Discomfort: They can be uncomfortable for some people, especially those with sensitive gums.

Recommendations.

Consult a Dentist: It's best to consult with your dentist about the right toothbrush for your specific dental needs.

Consider Soft-Bristled Brushes: Most dental professionals recommend using a soft-bristled toothbrush, as they are gentle on gums and effective at cleaning teeth.

In summary, while a hard-bristled toothbrush may clean your teeth effectively, the potential risks to your gums and enamel should be carefully considered.

Q12 Should I stop brushing my teeth for a few days if my gums are bleeding?

If your gums are bleeding, it's important to take care of your oral health, but stopping brushing your teeth is not the right approach. Here are some steps you should consider:

Continue Brushing Gently.

Use a soft-bristled toothbrush and brush gently to avoid further irritation.

Floss Carefully.

Flossing can help remove plaque and food particles that may be contributing to gum irritation. Be gentle to avoid exacerbating the bleeding.

Rinse with Salt Water.

A saltwater rinse can help soothe your gums and reduce inflammation. Mix about half a teaspoon of salt in a glass of warm water and rinse your mouth.

Avoid Irritants.

Stay away from tobacco, alcohol, and spicy foods that can irritate your gums.

Consult a Dentist.

If the bleeding persists for more than a few days or is severe, it's crucial to see a dentist. They can assess the situation and provide appropriate treatment.

Taking care of your oral hygiene is essential, even when experiencing issues like bleeding gums.

Q13 Do I need to brush my teeth after every meal to keep my gums healthy?

Brushing your teeth after every meal can be beneficial for maintaining oral health, including healthy gums. Here are some key points to consider:

Benefits of Brushing After Meals.

Removes Food Particles: Brushing helps eliminate food debris that can contribute to plaque buildup.

Reduces Plaque Formation: Regular brushing can prevent plaque from hardening into tartar, which can lead to gum disease.

Freshens Breath: It helps keep your breath fresh by removing odor-causing bacteria.

Recommendations.

Frequency: Ideally, brushing twice a day (morning and night) is recommended, but brushing after meals can be beneficial, especially if you consume sugary or acidic foods.

Wait Before Brushing: If you've eaten acidic foods (like citrus), it's best to wait about 30 minutes before brushing to avoid damaging your enamel.

Flossing: Incorporating flossing into your routine is also crucial for gum health, as it removes plaque and food particles from between teeth.

Conclusion.

While brushing after every meal can enhance gum health, ensure you're following a consistent oral hygiene routine that includes brushing twice a day and flossing daily. Regular dental check-ups are also important for maintaining overall oral health.

Q14 Even if I brush my teeth regularly, do I still need to get my gums checked?

Yes, even if you brush your teeth regularly, it's important to get your gums checked. Here are a few reasons why:

Importance of Gum Checks.

Early Detection of Gum Disease:

Regular check-ups can help detect early signs of gum disease (gingivitis or periodontitis) that might not be noticeable during daily brushing.

Professional Cleaning:

Dentists can remove plaque and tartar that regular brushing may miss, which can help prevent gum disease.

Assessment of Gum Health:

A dental professional can assess the health of your gums and provide advice tailored to your oral hygiene routine.

Overall Health Connection:

Gum health is linked to overall health. Conditions such as heart disease and diabetes can be influenced by gum health.

Personalized Care:

A dentist can offer personalized recommendations based on your specific oral health needs.

Recommendations.

Frequency of Visits: It's generally recommended to visit the dentist every six months for a check-up and cleaning.

Signs to Watch For: If you notice bleeding gums, persistent bad breath, or gum recession, schedule an appointment sooner.

Maintaining good oral hygiene at home is essential, but professional evaluations are crucial for long-term gum health.

Q15 Is brushing my teeth once a day enough to keep my mouth healthy?

Brushing your teeth once a day is better than not brushing at all, but it's generally recommended to brush at least twice a day for optimal oral health. Here are some key points to consider:

Benefits of Twice-Daily Brushing.

Plaque Removal: Brushing twice helps remove plaque that can accumulate on teeth throughout the day.

Prevention of Cavities: Regular brushing reduces the risk of tooth decay and cavities.

Gum Health: It helps prevent gum disease by keeping your gums clean and healthy.

Fresh Breath: Brushing more frequently can help maintain fresher breath.

Additional Oral Care Tips.

Floss Daily: Flossing helps remove food particles and plaque from between teeth where a toothbrush can't reach.

Regular Dental Check-ups: Visit your dentist regularly for cleanings and check-ups.

Limit Sugary Foods: Reducing sugar intake can help prevent cavities and improve overall oral health.

Use Mouthwash: An antibacterial mouthwash can provide additional protection against plaque and bad breath.

Conclusion.

While brushing once a day is a start, aiming for twice a day along with other oral hygiene practices is the best way to maintain a healthy mouth.

Q16 Can flossing cause gum recession?

Flossing is an essential part of oral hygiene, but improper technique can potentially lead to gum recession. Here are some key points to consider:

How Flossing Can Affect Gum Health.

Technique Matters:

Aggressive Flossing: Using excessive force or snapping the floss can damage the gum tissue and lead to recession.

Incorrect Angle: Flossing at the wrong angle can irritate the gums.

Frequency and Consistency:

Regular flossing is beneficial for removing plaque and preventing gum disease, which is a significant cause of gum recession.

Underlying Conditions:

Existing periodontal disease or other dental issues can exacerbate gum recession, and while flossing is important, it should be done correctly.

Recommendations.

Gentle Technique: Use a gentle sawing motion to guide the floss between teeth without forcing it.

Consult a Dentist: If you're experiencing gum issues, it's best to consult with a dental professional for personalized advice and proper flossing techniques.

Complementary Care: Combine flossing with regular brushing and dental check-ups for optimal gum health.

In summary, while flossing itself does not cause gum recession, improper technique can contribute to it. Proper flossing is crucial for maintaining healthy gums.

Q17 Do electric toothbrushes damage the gums more than regular toothbrushes?

Electric toothbrushes can offer several benefits over regular manual toothbrushes, but their impact on gums largely depends on usage technique rather than the type of toothbrush itself. Here are some key points to consider:

Potential Risks of Electric Toothbrushes.

Over-Pressure: If used with excessive force, electric toothbrushes can potentially cause gum damage, just like manual brushes. It's essential to apply gentle pressure.

Technique: Users unfamiliar with electric toothbrushes may not know how to use them properly, which can lead to gum irritation.

Benefits of Electric Toothbrushes.

Consistent Brushing: They often provide more consistent brushing motion, which can lead to better plaque removal if used correctly.

Timers and Sensors: Many electric toothbrushes have built-in timers and pressure sensors that help ensure effective and safe brushing.

Conclusion.

While electric toothbrushes can damage gums if misused, they can also promote better oral health when used correctly. It's crucial to follow the manufacturer's instructions and consult with a dentist for personalized advice. Regular dental check-ups can help monitor gum health regardless of the toothbrush type.

Q18 Would using mouthwash alone be enough without brushing my teeth?

Using mouthwash alone is not sufficient for maintaining optimal oral hygiene. Here are some key points to consider:

Why Mouthwash Alone Isn't Enough.

Plaque Removal:

Brushing your teeth helps remove plaque and food particles that mouthwash cannot eliminate. Plaque can harden into tartar if not removed, leading to gum disease and cavities.

Fluoride Benefits:

Many toothpastes contain fluoride, which helps strengthen tooth enamel and prevent decay. Mouthwash may contain fluoride as well, but it typically does not provide the same level of protection as brushing with toothpaste.

Mechanics of Cleaning:

Brushing physically scrubs the surfaces of your teeth, while mouthwash primarily freshens breath and may kill some bacteria, but it doesn't provide the mechanical action needed to clean effectively.

Gum Health:

Regular brushing helps maintain gum health, while relying solely on mouthwash may not adequately address issues like gingivitis.

Recommended Oral Hygiene Routine.

Brush Twice a Day: Use fluoride toothpaste and brush for at least two minutes.

Floss Daily: Flossing helps remove debris and plaque from between teeth where a toothbrush can't reach.

Use Mouthwash: Incorporate mouthwash as a supplementary step after brushing and flossing for added freshness and antibacterial benefits.

In summary, while mouthwash can be a helpful addition to your oral care routine, it should not replace brushing and flossing.

Q19 After I quit smoking, my gums started bleeding. Is that normal?

It's not uncommon to experience changes in your oral health after quitting smoking, including bleeding gums. Here are a few points to consider:

Possible Reasons for Bleeding Gums After Quitting Smoking:

Improved Blood Circulation: Smoking restricts blood vessels, and after quitting, blood flow improves, which can lead to changes in the gums.

Gum Health Improvement: Smoking can mask gum disease symptoms. Once you quit, your body may begin to heal, revealing underlying issues that were previously unnoticed.

Changes in Oral Hygiene Habits: Sometimes, people change their oral hygiene routines after quitting smoking, which can affect gum health.

Withdrawal Symptoms: Some individuals experience various withdrawal symptoms, which can include changes in oral health.

Recommendations:

Maintain Good Oral Hygiene: Brush and floss regularly to keep your gums healthy.

Visit a Dentist: Schedule an appointment with your dentist to assess your gum health and rule out any serious issues.

Stay Hydrated: Drink plenty of water to help maintain oral health.

Consider a Mouthwash: An antibacterial mouthwash may help reduce gum inflammation.

If the bleeding persists or worsens, it's essential to consult a healthcare professional for further evaluation.

Q20 Can natural products effectively keep my gums healthy?

Yes, natural products can be effective in maintaining gum health. Here are some key natural products and practices that may help:

Essential Oils.

Tea Tree Oil: Known for its antibacterial properties, it can help reduce plaque and gingivitis.

Peppermint Oil: Provides a refreshing taste and has antimicrobial properties.

Herbal Remedies.

Aloe Vera: Its soothing properties can help reduce inflammation and promote healing of the gums.

Green Tea: Rich in antioxidants, it may help reduce inflammation and improve gum health.

Nutritional Supplements.

Vitamin C: Essential for collagen production and gum health; consider citrus fruits or supplements.

Coenzyme Q10: May help improve gum health and reduce inflammation.

Natural Mouthwashes.

Salt Water Rinse: A simple saline solution can help reduce bacteria and soothe inflamed gums.

Apple Cider Vinegar: Diluted with water, it may help balance oral pH and reduce bacteria.

Dietary Choices.

Crunchy Fruits and Vegetables: Foods like apples and carrots can help clean teeth and stimulate gums.

Probiotics: Foods like yogurt may promote healthy bacteria in the mouth.

Good Oral Hygiene Practices.

Regular Brushing and Flossing: Essential for removing plaque and preventing gum disease.

Regular Dental Check-ups: Professional cleanings and check-ups are important for maintaining gum health.

Conclusion.

While natural products can support gum health, they should complement, not replace, traditional dental care practices. Always consult with a dental professional before starting any new treatment or supplement.

Q21 Can getting a dental scaling damage my teeth?

Dental scaling is a common procedure performed by dental professionals to remove plaque and tartar buildup from the teeth and gums. Here are some key points regarding its safety and potential effects:

Benefits of Dental Scaling.

Prevents Gum Disease: Regular scaling helps prevent gum disease by removing harmful bacteria.

Improves Oral Health: It can improve overall oral health and reduce the risk of cavities.

Freshens Breath: By removing bacteria and debris, scaling can help eliminate bad breath.

Potential Risks.

While dental scaling is generally safe, there are some considerations:

Enamel Damage: If done improperly or too aggressively, scaling can potentially damage the enamel. However, trained professionals are skilled in performing this procedure safely.

Sensitivity: Some patients may experience temporary tooth sensitivity following scaling.

Gum Irritation: Minor irritation or bleeding may occur, but this typically resolves quickly.

Conclusion.

When performed by a qualified dental professional, dental scaling is safe and beneficial for maintaining oral health. It's important to communicate any concerns with your dentist and follow their recommendations for care before and after the procedure. Regular dental check-ups can help ensure that scaling is done appropriately and safely.

Q22 Can dental scaling cause gaps between my teeth?

Dental scaling is a procedure that removes plaque and tartar buildup from the teeth and can improve gum health. However, there are a few factors to consider regarding gaps between teeth:

Possible Reasons for Gaps After Scaling.

Reduction of Gum Inflammation:

Scaling can reduce gum swelling, which may make it seem like there are gaps between the teeth that weren't noticeable before.

Tooth Movement:

If teeth were previously crowded, scaling might allow them to shift slightly, creating gaps.

Bone Loss:

In cases of severe periodontal disease, scaling can reveal changes in the bone that supports the teeth, leading to a perception of increased spacing.

Improper Technique:

If not done carefully, scaling could potentially affect the alignment of teeth.

Recommendations.

Consult Your Dentist: If you notice significant changes in spacing after scaling, it's important to discuss this with your dentist. They can assess your situation and recommend any necessary treatments.

Regular Check-ups: Regular dental visits can help monitor your oral health and prevent issues that may lead to gaps.

If you have further questions or concerns, feel free to ask!

Q23 Do all gum diseases require the same treatment?

No, not all gum diseases require the same treatment. Gum diseases, primarily categorized into gingivitis and periodontitis, have different causes, symptoms, and severities, which influence their treatment approaches. Here's a brief overview:

Gingivitis.

Description: Mild inflammation of the gums, often caused by plaque buildup.

Symptoms: Red, swollen gums that may bleed during brushing.

Treatment:

Improved oral hygiene (brushing and flossing).

Professional dental cleanings.

Antiseptic mouthwashes.

Periodontitis.

Description: A more severe form of gum disease that can lead to the destruction of the supporting bone and tissues.

Symptoms: Persistent bad breath, gum recession, pockets between teeth and gums, and tooth mobility.

Treatment:

Deep cleaning (scaling and root planing).

Antibiotics to control infection.

Surgical treatments (if necessary) to restore supportive tissues or bone.

Factors Influencing Treatment.

Severity: The stage of the gum disease will dictate the intensity of the treatment.

Underlying Health Conditions: Conditions like diabetes can affect treatment.

Patient Compliance: Adherence to oral hygiene practices is crucial for effective treatment.

Conclusion.

It's essential for individuals to consult with a dental professional to determine the specific type of gum disease they have and the appropriate treatment plan tailored to their condition. Regular dental check-ups are also vital for prevention and early detection.

Q24 Is gum treatment painful?

Gum treatment can vary in terms of pain and discomfort depending on the type of treatment being performed. Here are some common types of gum treatments and their associated pain levels:

Scaling and Root Planing.

Description: A deep cleaning procedure to remove plaque and tartar from below the gum line.

Pain Level: Mild to moderate discomfort is common, but local anesthesia is often used to minimize pain.

Gum Surgery.

Description: Surgical procedures to treat advanced gum disease or reshape the gums.

Pain Level: Can be painful post-surgery; however, pain management techniques are typically provided.

Gum Grafting.

Description: A procedure to cover exposed roots by taking tissue from another part of the mouth.

Pain Level: Moderate pain is expected after the procedure, but it can usually be managed with medication.

Laser Gum Treatment.

Description: A less invasive method for treating gum disease using lasers.

Pain Level: Generally less painful than traditional methods, with minimal discomfort reported.

Pain Management.

Dentists often use local anesthesia, sedation, and pain relievers to help manage discomfort during and after treatment.

If you're concerned about pain during a specific gum treatment, it's best to discuss this with your dentist, who can provide personalized advice and options for pain management.

Q25 Can gum surgery cause tooth loss?

Yes, gum surgery can potentially lead to tooth loss, although this is not a common outcome. Here are some factors to consider:

Reasons Why Gum Surgery Might Lead to Tooth Loss.

Underlying Conditions: If there are severe periodontal diseases or other underlying dental issues, the surgery may not be able to save the affected teeth.

Infection: Post-surgical infections can occur, which might compromise the health of the surrounding teeth and lead to tooth loss.

Bone Loss: In some cases, the surgery may expose or remove bone that supports the teeth, leading to instability.

Healing Complications: If the healing process is compromised due to poor oral hygiene, smoking, or other factors, it can affect the stability of the teeth.

Patient-Specific Factors: Individual factors such as age, overall health, and the presence of other dental issues can influence the outcome of the surgery.

Conclusion.

While gum surgery is generally aimed at preserving teeth and improving oral health, there are risks involved. It's crucial to discuss these risks with your dentist or periodontist before undergoing the procedure. Regular follow-ups and proper oral hygiene are essential for a successful outcome.

Q26 Can gum treatment make my teeth feel loose?

Yes, gum treatment can sometimes make your teeth feel loose, especially if the treatment involves scaling, root planing, or other procedures that target gum disease. Here are a few reasons why this might happen:

Reasons for Loose Teeth After Gum Treatment.

Inflammation Reduction: After gum treatment, the inflammation in the gums may decrease, which can temporarily change how your teeth feel.

Support Changes: If the treatment involved cleaning below the gum line, it may have affected the support structures of your teeth, leading to a feeling of looseness.

Bone Loss: In cases of advanced gum disease, there may be some bone loss that was previously masked by swollen gums. Once inflammation is reduced, this loss can become more apparent.

Healing Process: As your gums heal, they may shrink slightly, which can create a sensation of looseness in the teeth.

When to Seek Help.

If you notice persistent looseness or any other concerning symptoms, it's important to consult with your dentist or dental specialist. They can assess your condition and provide guidance on what to expect during the healing process.

Q27 Does tartar removal cause more tartar buildup?

Tartar removal does not directly cause more tartar buildup. However, there are a few considerations to keep in mind:

Understanding Tartar Buildup.

What is Tartar?: Tartar, or calculus, is hardened plaque that forms on teeth when plaque is not removed through regular brushing and flossing.

Formation Process: Tartar forms over time and can only be removed by a dental professional.

Effects of Tartar Removal.

Initial Removal: When tartar is removed, it cleans the surface of the teeth, which can help reduce the risk of gum disease and cavities.

Aftercare: If proper oral hygiene practices (like brushing twice a day and flossing) are not maintained after tartar removal, plaque can quickly accumulate again, leading to new tartar formation.

Regular Cleanings: Regular dental cleanings help prevent tartar buildup by removing plaque before it hardens.

Conclusion.

While tartar removal itself does not cause more tartar buildup, neglecting post-removal oral hygiene can lead to a faster return of tartar. Maintaining good oral hygiene is essential for preventing future buildup.

Q28 Are antibiotics enough to treat gum disease?

Antibiotics can play a role in the treatment of gum disease, particularly in cases of periodontitis, but they are not always sufficient on their own. Here's a breakdown of their use:

Role of Antibiotics in Gum Disease Treatment.

Types of Gum Disease:

Gingivitis: Mild form, often reversible with good oral hygiene.

Periodontitis: More severe, involves loss of bone and tissue; may require antibiotics.

When Antibiotics are Used:

Antibiotics may be prescribed when:

There is significant bacterial infection.

Traditional treatments (like scaling and root planing) are not enough.

The patient has specific health conditions that complicate treatment.

Limitations of Antibiotics:

Antibiotics alone cannot address the underlying causes of gum disease (like plaque and tartar).

They may only reduce bacterial load temporarily.

Resistance can develop if antibiotics are overused.

Comprehensive Treatment Approach

Professional Cleaning: Scaling and root planing are crucial to remove plaque and tartar.

Improved Oral Hygiene: Regular brushing, flossing, and possibly using antimicrobial mouth rinses.

Regular Dental Visits: Ongoing care and monitoring by a dental professional are essential.

Lifestyle Changes: Quitting smoking and improving diet can enhance gum health.

Conclusion.

While antibiotics can be a helpful adjunct in treating gum disease, they should be part of a comprehensive treatment plan that includes professional dental care and improved oral hygiene practices. Always consult with a dentist for a tailored treatment approach.

Q29 After gum disease treatment, will I ever need treatment again?

After gum disease treatment, the need for further treatment can depend on several factors:

Severity of Gum Disease.

Mild Cases: If the gum disease was caught early and treated effectively, you may not need further treatment.

Severe Cases: More advanced gum disease may require ongoing maintenance and monitoring.

Oral Hygiene Practices.

Maintaining good oral hygiene at home (brushing, flossing, and regular dental check-ups) is crucial. Poor hygiene can lead to a recurrence of gum disease.

Regular Dental Visits.

Regular check-ups (typically every 3 to 6 months) can help monitor your gum health and catch any issues early.

Lifestyle Factors.

Factors such as smoking, diet, and overall health can influence your gum health. Addressing these can reduce the risk of needing further treatment.

Conclusion.

While many patients do not need additional treatment after successful gum disease treatment, it’s essential to stay vigilant with oral care and follow your dentist's recommendations. If you notice any signs of gum disease returning (like bleeding gums), consult your dentist promptly.

Q30 Is surgery always necessary for gum treatment?

Surgery is not always necessary for gum treatment. The need for surgical intervention depends on the severity of the gum disease and the specific condition being treated. Here are some common approaches to gum treatment:

Non-Surgical Treatments.

Scaling and Root Planing: A deep cleaning procedure that removes plaque and tartar from below the gum line.

Antibiotics: Topical or oral antibiotics may be prescribed to help control bacterial infection.

Oral Hygiene Education: Improving brushing and flossing techniques can help manage gum disease.

Regular Dental Cleanings: Professional cleanings every six months can help maintain gum health.

Surgical Treatments.

Gum Grafting: Used to restore lost gum tissue.

Flap Surgery: Involves lifting the gums to clean the roots and reduce pocket depth.

Bone Grafting: May be necessary if there is significant bone loss due to advanced gum disease.

Conclusion.

The treatment plan should be tailored to the individual based on their specific condition. It's essential to consult with a dental professional to determine the best course of action.
