# Supplementary material for: Comparative Evaluation of Large Language Models in Responding to Common Misconceptions Regarding Periodontal Disease and Oral Hygiene
Source: Diagnostics (Basel). 2026 Sep 28;16(19):3158. doi: 10.3390/diagnostics16193158 (PMC13664004; doi:10.3390/diagnostics16193158)
Supplement: Supplementary file 1 [file diagnostics-16-03158-s001.zip › Supplementary File S5 LLMs response stylistic elements removed.zip!/2. LLMs response/Claude 4.6 Sonnet.pdf]

Q1 Do gum diseases only affect elderly people?

No, gum disease can affect people of any age including children, teenagers, and young adults.

Who Gets Gum Disease?

Children and teenagers can develop gingivitis (early-stage gum disease), often due to poor brushing habits, hormonal changes during puberty, or certain medications. It's actually quite common in adolescents.

Young adults are frequently affected too gum disease can progress silently without pain, so many people in their 20s and 30s have it without realizing it.

Middle-aged adults are at significant risk, especially if early warning signs were ignored. Studies consistently show that periodontitis (advanced gum disease) becomes more prevalent starting in the 30s and 40s.

Older adults do have *higher rates* overall, which is probably where the myth comes from but that's largely because gum disease is cumulative and gets worse with time if untreated, not because age itself is the primary cause.

The Real Risk Factors.

Age is a contributing factor, but these matter just as much often more:

Poor oral hygiene the main driver at any age.

Smoking one of the strongest risk factors.

Diabetes bidirectionally linked to gum disease.

Genetics some people are simply more susceptible.

Hormonal changes pregnancy, puberty, menopause all affect gum health.

Dry mouth (from medications or conditions).

Stress weakens immune response, allowing bacteria to thrive.

Nutritional deficiencies especially vitamin C.

The Takeaway.

Gum disease is largely preventable at any age with consistent brushing, flossing, and regular dental cleanings. The earlier it's caught, the easier it is to reverse gingivitis is fully reversible, while advanced periodontitis is managed but not fully cured. Good habits in youth are the best long-term protection.

Q2 Is it normal if my gums bleed when I brush my teeth?

Bleeding gums when brushing are common, but not normal it's your body flagging that something's off. Here's what's likely going on:

The most common causes.

Gingivitis the 1 culprit. Plaque buildup along the gumline causes inflammation, and inflamed gums bleed easily. This is reversible with good hygiene.

Brushing too hard or with a hard-bristle brush aggressive scrubbing damages gum tissue.

Infrequent flossing if you rarely floss and then do it, bleeding is almost expected at first. It usually stops within a week of consistent daily flossing.

New medication blood thinners, some antidepressants, and even antihistamines can make gums more prone to bleeding.

Hormonal changes pregnancy, menstruation, or puberty can temporarily make gums more sensitive.

When to take it more seriously.

Occasional, minor bleeding that resolves quickly is less urgent. But see a dentist if:

Bleeding happens every time you brush, or bleeds heavily.

Your gums are swollen, red, or receding.

You have bad breath that doesn't go away.

It's been more than 6 months since a dental checkup.

You have diabetes, which significantly raises gum disease risk

What you can do now.

Switch to a soft-bristle brush if you're not already using one.

Brush gently in small circular motions force isn't the goal, coverage is.

Floss daily, consistently, even if it bleeds at first.

Rinse with a non-alcoholic mouthwash to reduce bacteria.

Book a dental cleaning professional scaling removes tartar that brushing can't touch.

Gingivitis caught early is entirely reversible, so it's worth acting on rather than waiting. If you've had consistent bleeding for more than two weeks despite better hygiene, that's your cue to get it checked.

Q3 Can I get gum disease even if I brush my teeth regularly?

Yes, you can absolutely get gum disease even with regular brushing and it's more common than most people expect.

Why brushing alone isn't enough.

Gum disease (periodontal disease) is caused by bacterial plaque a sticky film that forms constantly on and between your teeth. The catch is that a toothbrush only cleans the *surfaces* of your teeth. The spaces between teeth and under the gumline are largely unreachable by bristles alone.

If plaque isn't removed from those areas, it hardens into tartar (calculus) within 24 72 hours. Tartar can't be brushed off only a dental professional can remove it. Once tartar builds up along the gumline, it irritates the gum tissue and creates an environment where bacteria thrive.

Other factors that raise your risk even with good brushing.

Skipping floss This is the biggest gap. Flossing disrupts plaque between teeth where gum disease often starts.

Genetics Some people are simply more susceptible to periodontal bacteria.

Smoking Significantly impairs gum healing and masks early symptoms.

Hormonal changes Pregnancy, menopause, and puberty can make gums more reactive.

Diabetes Raises infection risk and slows healing throughout the body.

Certain medications Some drugs (antihistamines, antidepressants, blood pressure meds) reduce saliva, which normally helps protect gums.

Stress Weakens immune response, making it harder to fight off the bacteria.

Early warning signs to watch for.

Gums that bleed when you brush or floss.

Redness, swelling, or tenderness.

Gums pulling away from teeth.

Persistent bad breath.

The earliest stage gingivitis is fully reversible with professional cleaning and better home care. If it progresses to periodontitis, the damage to bone and tissue becomes permanent, though it can be managed.

The practical takeaway: brushing twice daily is necessary but not sufficient. Flossing (or using an interdental brush), and getting a professional cleaning every 6 12 months, are what close the gap.

Q4Can I have gum disease even if my gums don't hurt?

Yes, absolutely and this is one of the most important things to understand about gum disease. Pain is a late symptom, not an early warning sign.

Gum disease is often silent.

The early stage, gingivitis, typically causes:

Redness or swelling you might not notice.

Gums that bleed when you brush or floss (many people dismiss this as normal it isn't).

Slight puffiness along the gumline.

None of this necessarily hurts. Many people have gingivitis for years without any discomfort.

The danger zone: periodontitis.

If gingivitis is left untreated, it can progress to periodontitis, where the infection spreads below the gumline and starts destroying the bone that holds your teeth in place. This is still often painless until significant damage has occurred. By the time a tooth feels loose or an area becomes sore, you may have already lost substantial bone.

Signs to watch for (even without pain).

Bleeding gums when brushing or flossing.

Gums pulling away from your teeth (teeth looking "longer").

Persistent bad breath that doesn't go away.

Gaps forming between teeth.

Teeth feeling slightly loose.

Why this matters.

Gum disease has also been linked to broader health issues cardiovascular disease, diabetes complications, and others so catching it early is worth taking seriously.

The most reliable way to know is a dental exam, where a dentist or hygienist probes the depth of the gum pockets around each tooth. That measurement tells the real story, regardless of how your gums feel.

Q5 Can gum disease get better without treatment?

The answer depends on the stage of the disease.

Gingivitis (early stage) yes, it can reverse.

Gingivitis is inflammation limited to the gums, and it's the one stage that *can* resolve on its own but only with a significant change in oral hygiene habits. If you:

Brush thoroughly twice a day (proper technique matters).

Floss daily to remove plaque between teeth.

Use an antibacterial mouthwash.

inflamed gums can return to healthy within 2 4 weeks. The bone and tissue underneath haven't been damaged yet, so there's nothing permanent to repair.

Periodontitis (advanced stage) no, it cannot.

Once gum disease progresses to periodontitis, the bone and connective tissue that hold your teeth in place start to break down. That damage is irreversible without professional treatment. Left alone, it:

Continues to worsen it doesn't plateau on its own.

Leads to deeper "pockets" between teeth and gums where bacteria thrive.

Eventually causes tooth loss and can affect jawbone density.

No amount of better brushing undoes bone loss that's already occurred.

The tricky part: knowing which stage you're in.

Most people can't tell. Periodontitis is often painless until it's quite advanced which is why dentists call it a "silent" disease. Signs worth taking seriously:

Gums that bleed when brushing or flossing.

Persistent bad breath.

Gums pulling away from teeth (teeth looking "longer").

Loose teeth or a change in bite.

If you've noticed any of these, a dental exam with X-rays is the only reliable way to assess whether bone loss has occurred. Caught early as gingivitis, good home care may be all you need. Caught later, scaling and root planing (a deep clean) or other interventions become necessary to stop progression.

Q6 Are gum diseases rare?

Gum disease is actually very common the opposite of rare.

Periodontitis (advanced gum disease) affects roughly 19% of adults globally, which translates to about 1 billion people. Milder gum inflammation (gingivitis) is even more widespread; some estimates suggest it affects the majority of adults at some point in their lives.

A few key facts:

It's the leading cause of tooth loss in adults worldwide.

Risk goes up with age it's particularly prevalent in people over 45.

It's strongly linked to other conditions like diabetes, cardiovascular disease, and smoking, which makes it both a cause and a consequence of broader health issues.

It's largely preventable and treatable, but many people don't notice symptoms early on because the early stages are painless.

The reason it might seem rare is that people often don't talk about it, and mild gingivitis can go unnoticed. But dentists and periodontists consider it one of the most common chronic diseases in humans.

Q7Is it normal to lose teeth because of gum disease as you get older?

Gum disease (periodontal disease) is actually one of the leading causes of tooth loss in adults, so unfortunately, yes it's common, though not inevitable.

How it happens.

Gum disease progresses in stages:

Gingivitis the early stage. Gums become inflamed and bleed easily. Fully reversible with good hygiene.

Periodontitis if untreated, infection spreads below the gumline. The body's immune response starts breaking down the bone and connective tissue holding teeth in place.

Advanced periodontitis significant bone loss means teeth loosen and eventually fall out or need extraction.

The connection to age is real but indirect. It's not that aging itself destroys your gums it's that decades of accumulated plaque, inconsistent dental care, and compounding risk factors take their toll over time.

What accelerates it.

Smoking (one of the biggest risk factors).

Poorly controlled diabetes.

Dry mouth (common with many medications older adults take).

Genetic predisposition.

Chronic stress.

Hormonal changes.

The important nuance.

Tooth loss from gum disease is largely preventable. People who brush and floss consistently, get regular professional cleanings, and catch problems early can keep their teeth well into old age. The idea that losing teeth is just a normal part of aging is outdated it's more a sign of disease that wasn't caught or treated in time.

If you're noticing symptoms like bleeding gums, recession, bad breath that won't go away, or any looseness in teeth, a periodontist (gum specialist) can assess the bone levels and catch things earlier than a general check-up might. Early intervention makes a significant difference in outcomes.

Q8If gum disease runs in my family, will I definitely get it too?

Not necessarily genetics loads the gun, but lifestyle and oral hygiene largely determine whether it fires.

What genetics actually does.

Having a family history of gum disease (periodontitis) does raise your risk, but it's not a simple one-gene destiny. What you may inherit is:

A more reactive immune response your immune system may trigger more inflammation in response to oral bacteria than someone else's would.

Differences in how your body repairs connective tissue and bone.

Certain gene variants (like variants in the IL-1 gene cluster) linked to higher inflammatory responses.

Studies suggest genetics accounts for roughly 50% of periodontitis susceptibility in the general population, which sounds high but it also means half the equation is environmental and behavioral.

What you can actually control.

These factors often matter more than your genes:

Factor.	Impact.
Brushing flossing.	Removes the bacterial plaque that triggers the whole disease cascade.
Smoking.	One of the strongest known risk factors it can mask early symptoms too.
Diet.	High sugar intake feeds harmful bacteria; vitamin C and D deficiencies impair gum tissue.
Blood sugar control.	Diabetes and gum disease have a well-documented two-way relationship.
Stress.	Elevates cortisol, which suppresses immune response and worsens inflammation.
Regular dental cleanings.	Professional cleaning removes calculus that home care can't reach.

The practical upshot.

Family history is genuinely useful information it means you should be more vigilant, not fatalistic. Tell your dentist, because they may recommend more frequent cleanings (every 3 4 months instead of 6), and they can monitor early signs like pocket depth and bleeding before anything serious develops.

Early-stage gum disease (gingivitis) is completely reversible with good care. Even moderate periodontitis, caught early, is very manageable. The people who develop serious bone loss typically had silent, unchecked disease for years which consistent dental visits prevent.

Q9Can stress make me more likely to get gum disease?

Yes, and it's a well-established connection. Here's how it works:

Stress Gum Disease: The Key Pathways.

Immune suppression is the biggest factor. Chronic stress elevates cortisol, which damps down your immune response meaning your body is less equipped to fight the bacteria that cause gum disease (periodontitis).

Inflammation is another route. Stress drives systemic inflammation, and your gums are particularly vulnerable to inflammatory responses. This can accelerate the breakdown of gum tissue and bone around teeth.

Behavior changes matter too. Stressed people tend to:

Skip or rush brushing and flossing.

Smoke or drink more.

Eat more sugar and processed food.

Clench or grind their teeth (bruxism), which stresses the gum-tooth junction.

Dry mouth is a side effect of many anxiety medications, and saliva is one of your mouth's natural defenses against harmful bacteria.

What the Research Shows.

Studies have consistently found that people with high psychological stress, depression, or financial hardship have higher rates of periodontitis even after controlling for oral hygiene habits. The link is strong enough that some periodontists now ask about stress levels as part of a standard intake.

The practical upshot: if you're going through a stressful period, your gums genuinely need extra attention more consistent brushing, flossing, and not skipping dental check-ups. Stress-related gum disease can progress quickly and quietly, since it often doesn't hurt until it's already fairly advanced.

Q10 Do gum diseases only affect the mouth and teeth?

No gum disease has effects well beyond the mouth, and the research on this has grown substantially over the past few decades.

Local effects (mouth and teeth).

The obvious damage happens locally: inflamed, bleeding gums, bone loss around teeth, receding gum tissue, loose teeth, and eventually tooth loss. This is periodontitis in its advanced form.

Systemic effects (the rest of the body).

The mouth isn't sealed off from the rest of the body. Bacteria from infected gum tissue and the chronic inflammation they trigger can enter the bloodstream and travel throughout the body.

Heart and cardiovascular system.

The most well-studied link. People with periodontitis have a significantly higher risk of heart disease, atherosclerosis (arterial plaque buildup), and stroke. The leading theory is that oral bacteria (especially *Porphyromonas gingivalis*) contribute to arterial inflammation and plaque formation.

Diabetes.

This one is a two-way street. High blood sugar makes gum disease worse (inflamed tissue heals poorly), *and* gum disease makes blood sugar harder to control by worsening systemic inflammation. Treating periodontitis has been shown to modestly improve HbA1c levels in diabetic patients.

Pregnancy complications.

Severe gum disease during pregnancy is associated with preterm birth and low birth weight, likely through inflammatory mediators that can influence uterine activity.

Respiratory disease.

Inhaling oral bacteria over time can contribute to pneumonia and worsen COPD, particularly in older adults or those with compromised immune systems.

Cognitive health.

Emerging research links chronic periodontal infection especially *P. gingivalis* to Alzheimer's disease. The bacterium and its toxic enzymes (gingipains) have been found in the brains of Alzheimer's patients at autopsy, suggesting a possible causal pathway, though this is still an active area of investigation.

Rheumatoid arthritis.

There's a bidirectional relationship here too. Both conditions involve chronic inflammatory processes, and *P. gingivalis* can trigger the same type of protein modification (citrullination) that drives autoimmune attacks in RA joints.

Why this matters practically.

Gum disease is extremely common and often silent in its early stages many people have it without knowing. The implication of this systemic research is that treating and preventing gum disease isn't just about keeping your teeth; it's genuinely relevant to overall health. Regular dental cleanings and early treatment of gingivitis (the reversible early stage) matter more than most people realize.

Q11 Will a hard-bristled toothbrush clean my teeth better?

No harder bristles don't mean cleaner teeth. In fact, the opposite tends to be true.

What hard bristles actually do:

They can abrade tooth enamel over time, especially with aggressive brushing.

They're more likely to cause gum recession which is irreversible.

They can wear down the cervical area of teeth (the neck, near the gumline), leading to sensitivity.

Why soft bristles work better: Plaque is a soft, loosely attached film. It doesn't require force to remove it just needs bristles reaching the right spots, including slightly under the gumline and between teeth. Soft bristles flex to get there; stiff ones don't.

Most dentists and the ADA recommend soft bristles for almost everyone. Extra-soft is recommended for people with gum disease or sensitivity.

The real variables that matter more than bristle hardness:

Technique gentle circular or Bass method strokes, not scrubbing.

Duration two minutes, hitting all surfaces.

Consistency twice a day, every day.

Flossing no toothbrush reaches between teeth effectively.

If your current brush is hard and you've been using it a while, it's worth switching you may notice less sensitivity fairly quickly.

Q12 Should I stop brushing my teeth for a few days if my gums are bleeding?

No stopping is actually the opposite of what you should do. Here's why:

Bleeding gums are usually a sign of gum inflammation (gingivitis), most commonly *caused* by plaque buildup. If you stop brushing, more plaque accumulates, which worsens the inflammation and makes the bleeding worse over time, not better.

What you should do instead.

Keep brushing gently, twice a day, with a soft-bristled brush. Soft bristles clean effectively without traumatizing the gum tissue.

Add flossing this is often the missing piece. Plaque between teeth is a primary driver of gum bleeding, and brushing alone can't reach it. Yes, it may bleed more at first; that's normal and typically resolves within a week or two of consistent flossing.

Use gentle technique angle the brush at 45° toward the gumline and use small circular strokes rather than hard back-and-forth scrubbing.

Give it 1–2 weeks with consistent brushing and flossing, bleeding from gingivitis usually improves noticeably.

When to see a dentist.

If the bleeding is heavy, persists beyond 2 weeks of good oral hygiene, is accompanied by pain, swelling, or receding gums, or if you haven't had a professional cleaning in a while it's worth getting checked. More advanced gum disease (periodontitis) won't resolve with brushing alone and needs professional treatment.

Some medications (like blood thinners) and conditions (like diabetes or vitamin C deficiency) can also contribute to gum bleeding, so persistent cases are worth discussing with a dentist or doctor.

Q13 Do I need to brush my teeth after every meal to keep my gums healthy?

Brushing after every meal isn't strictly necessary, and for some people it can actually cause harm here's the practical breakdown:

What actually matters for gum health.

Twice a day is the evidence-based standard. Brushing thoroughly in the morning and before bed removes the plaque buildup that causes gum disease. The *quality* of those two sessions matters far more than the frequency.

Flossing (or interdental cleaning) is arguably more important for gums. Gum disease starts between teeth and at the gumline places your toothbrush can't reach well. Once daily is the recommendation.

The case *against* brushing immediately after every meal.

Wait at least 30 60 minutes after acidic foods or drinks (citrus, coffee, soda, wine). Acid temporarily softens enamel, and brushing right away can abrade it. Rinse with water instead.

Overbrushing with hard pressure can cause gum recession which is itself a gum health problem.

What does help between meals.

Rinsing with water after eating removes loose food debris and dilutes acids.

Chewing sugar-free gum (with xylitol) stimulates saliva, which neutralizes acid and remineralizes enamel.

Avoiding constant snacking, which keeps your mouth in a state of acid exposure all day.

The bottom line.

Two good brushing sessions daily flossing regular dental cleanings is the core routine that protects gums. If you *want* to brush after lunch, that's fine just wait a bit and use a soft-bristled brush with gentle pressure.

If you're already noticing gum issues (bleeding, swelling, recession), a dentist or periodontist can assess whether something more targeted is needed, since those can signal early gum disease that routine brushing alone won't reverse.

Q14 Even if I brush my teeth regularly, do I still need to get my gums checked?

Yes, absolutely. Brushing takes care of the surfaces of your teeth, but it doesn't fully address what's happening below the gumline and that's where serious problems develop quietly.

Here's why gum checks matter even with good brushing habits:

Gum disease starts invisibly. Gingivitis and periodontitis develop in pockets between the teeth and gums that you can't see or feel until they're fairly advanced. A dentist or hygienist measures these pockets with a probe to catch problems early.

Plaque hardens into tartar. Within 24 72 hours, plaque that brushing misses calcifies into tartar (calculus) a hard deposit that no toothbrush can remove. It can only be scaled off professionally, and it's a major driver of gum inflammation.

Gum disease is largely painless early on. Bleeding gums might be the only sign, and many people ignore it or don't notice it at all. By the time there's pain or visible recession, significant bone loss may have already occurred.

Brushing technique isn't perfect. Even conscientious brushers tend to miss the same spots consistently typically the backs of molars and tight interdental areas. A professional check catches these patterns.

Systemic links. Gum disease is associated with cardiovascular disease, diabetes, and other systemic conditions, so it's more than just a dental issue.

The general recommendation is a professional cleaning and gum assessment every 6 months for most people, or more frequently (every 3 4 months) if you've had gum disease before.

Think of brushing as daily maintenance and dental visits as the inspection both are doing different jobs.

Q15 Is brushing my teeth once a day enough to keep my mouth healthy?

Once a day is better than nothing, but it's generally not enough. Here's why dental professionals recommend more:

What once-a-day misses.

Bacterial buildup Plaque starts reforming within 4 12 hours after brushing. Once a day leaves it undisturbed for too long, especially overnight when saliva flow drops and the mouth becomes more acidic.

Overnight risk Most dentists recommend brushing *before bed* as the single most important brush. If you're only doing it once, skipping the night brush is the worst choice.

Gum disease Consistent plaque accumulation inflames the gums (gingivitis), which can progress to periodontitis over time.

The actual standard.

The consensus recommendation from dental health organizations is:

Brush twice a day morning and before bed, for 2 minutes each time.

Floss once a day brushing only cleans about 60% of tooth surfaces; the spaces between teeth are where cavities most commonly develop.

Use fluoride toothpaste critical for enamel protection.

What "just once" looks like long-term.

People who brush once daily do have higher rates of cavities and gum disease than twice-daily brushers, though individual factors like diet, genetics, saliva composition, and fluoride exposure also play a big role. Some people with very low-sugar diets and naturally protective saliva get away with less; most don't.

If twice a day feels like a hard habit to build, anchoring one brush to bedtime and one to another routine (coffee, shower) makes it much easier to stick to.

Q16 Can flossing cause gum recession?

Yes, flossing *can* cause gum recession but only when done incorrectly. Done right, it actually helps prevent the gum disease that's a leading cause of recession.

How flossing causes damage.

The main culprit is aggressive technique:

Snapping the floss down hard into the gumline instead of sliding it gently creates repeated trauma to the gum tissue.

Sawing back and forth at the gumline rather than wrapping the floss in a C-shape around each tooth and moving it up and down.

Flossing too forcefully every day compounds the micro-trauma over time, and gum tissue doesn't regenerate well once lost.

Over months or years, this repeated mechanical irritation can cause the gums to recede particularly between teeth where the papilla (the triangular bit of gum between teeth) is delicate.

How to floss without causing recession.

Ease the floss gently between teeth don't snap it.

Curve it into a C-shape around each tooth, hugging the tooth surface.

Slide it up and down against the tooth, going just slightly below the gumline (about 1mm) not jabbing into it.

Use a fresh section of floss for each gap.

Light pressure is all you need; the goal is disrupting plaque, not scrubbing.

The bigger picture.

Gum recession has several causes aggressive toothbrushing, gum disease (periodontitis), thin gum tissue genetically, teeth grinding, and yes, improper flossing. If you're seeing recession, it's worth mentioning to a dentist to identify which factor is driving it, since the fix depends on the cause.

Q17 Do electric toothbrushes damage the gums more than regular toothbrushes?

The short answer: no, not inherently and for most people, electric toothbrushes are actually gentler on gums than manual brushing.

Why electric toothbrushes tend to be better for gums.

Consistent, appropriate pressure. Most modern electric toothbrushes have a pressure sensor that alerts you when you're pressing too hard something manual brushing has no feedback for. Over-aggressive manual scrubbing is a very common cause of gum recession.

Better plaque removal near the gumline. The oscillating or sonic action cleans more effectively along the gumline, which reduces the inflammation (gingivitis) that is the primary driver of gum damage over time.

Less reliance on technique. People tend to unconsciously "scrub" with a manual brush, especially on dominant-hand sides. Electric brushes do the work, so technique errors matter less.

When electric toothbrushes can cause problems.

Still pressing too hard without heeding the sensor the brush doing the oscillating doesn't mean force becomes harmless.

Using a worn-out brush head. Frayed bristles on any brush can be abrasive. Replace heads every 3 months.

Wrong bristle type. Hard bristles electric or manual damage gums and enamel. Stick to soft bristles.

The bottom line.

The evidence consistently points in one direction: electric toothbrushes reduce gingivitis and gum bleeding more than manual brushes for the average user. Gum damage is mostly caused by brushing technique and pressure, not the type of brush.

If you're seeing gum recession or sensitivity, the more likely culprits are brushing too hard, brushing too soon after acidic food/drink, grinding your teeth, or an underlying gum condition worth raising with a dentist either way.

Q18 Would using mouthwash alone be enough without brushing my teeth?

No, mouthwash alone isn't sufficient as a replacement for brushing.

Here's why brushing is irreplaceable:

Physical removal is the key mechanism. Plaque is a sticky biofilm that clings to tooth surfaces. Mouthwash can kill bacteria, but it can't dislodge or scrub away that film only the mechanical action of a toothbrush does that.

Fluoride delivery. Most toothpastes contain fluoride, which remineralizes enamel and protects against cavities. While some mouthwashes also contain fluoride, the contact time and concentration in toothpaste is generally more effective.

Gumline and between teeth. Brushing cleans the gumline where plaque accumulates most aggressively. Mouthwash rinses around but doesn't get into the tight contact points the way mechanical cleaning does.

What mouthwash actually does well:

Reduces bacteria counts temporarily.

Freshens breath.

Can reach areas between teeth that brushing misses.

Antiseptic types (e.g. chlorhexidine) help with gum inflammation as a *supplement.*

Think of mouthwash the way you'd think of hand sanitizer vs. washing your hands with soap useful in a pinch or as a complement, but not a substitute for the real thing.

If you're in a situation where brushing isn't possible (traveling, at work), rinsing with water or mouthwash is better than nothing, but it shouldn't become a regular substitute. Dentists consistently recommend brushing at least twice a day as the non-negotiable baseline.

Q19 After I quit smoking, my gums started bleeding. Is that normal?

Yes, this is actually quite common and well-documented it's sometimes called "quitter's gum" or described as part of the rebound effect after stopping smoking.

Here's what's happening:

Why smoking masks gum disease Nicotine and tobacco smoke constrict blood vessels in your gums, which *suppresses* the normal inflammatory response. Smokers often have significant gum disease without the usual warning signs (bleeding, swelling) precisely because smoking masks them.

What happens when you quit Once nicotine is out of your system, blood flow to your gums normalizes. Your immune system "wakes up" and starts responding to any existing inflammation or infection and that means bleeding, tenderness, and sometimes even swelling that wasn't visible before. It's not that quitting *caused* gum disease; it's that quitting *revealed* it.

Is it serious? It can be. The underlying gum disease (gingivitis or periodontitis) was likely there before smoking was just hiding it. Now that it's visible, it needs attention.

What to do.

See a dentist soon, ideally within a few weeks. Let them know you just quit smoking. A professional cleaning and gum assessment is the right move here.

Keep brushing gently twice a day and floss daily bleeding gums that aren't stimulated don't heal as quickly.

The bleeding often improves significantly within 2 8 weeks as your gums recover, *if* you stay on top of oral hygiene.

The good news: quitting smoking is one of the single best things you can do for your long-term gum health. Within a year, your risk of gum disease progression drops substantially. The bleeding now is uncomfortable, but it's your body doing exactly what it should.

Q20 Can natural products effectively keep my gums healthy?

Yes several natural products have genuine, research-backed benefits for gum health, though they work best as complements to good hygiene, not replacements for it. Here's what the evidence actually supports:

Well-Supported Options.

Saltwater rinses One of the simplest and most effective options. Salt creates an alkaline environment that disrupts bacterial growth, reduces inflammation, and helps heal irritated gum tissue. Rinse with ½ tsp of salt in warm water for 30 seconds, once or twice a day.

Oil pulling (coconut oil) Swishing a tablespoon of coconut oil for 10 15 minutes has decent clinical evidence behind it it reduces plaque and gingivitis markers, likely due to the antimicrobial properties of lauric acid. It's not a magic fix, but it's a legitimate adjunct to brushing and flossing.

Green tea Rich in catechins (antioxidants), green tea has been shown in studies to reduce gum inflammation and bacterial load when consumed regularly or used as a rinse.

Aloe vera gel Applied topically to the gums, aloe vera has antimicrobial and anti-inflammatory properties. Some trials show it performs comparably to conventional chlorhexidine mouthwash for reducing gingivitis, with fewer side effects like staining.

Neem Used for centuries in Ayurvedic medicine, neem (as a toothpaste, powder, or chew stick) has solid antibacterial evidence and is one of the more robustly studied herbal options for gum disease.

Weaker or Mixed Evidence.

Tea tree oil antimicrobial in the lab, but clinical results in humans are inconsistent. Can also be irritating or toxic if swallowed, so it requires care.

Turmeric (curcumin) gel promising anti-inflammatory data but mostly small trials; more research needed.

Hydrogen peroxide rinses technically natural-ish; does reduce bacteria, but overuse can damage gum tissue.

Natural products can meaningfully support gum health reducing inflammation, cutting bacterial load, and soothing irritated tissue. But they can't substitute for mechanical plaque removal (brushing flossing) or professional cleaning, which physically dislodges tartar that no rinse can touch.

If you're dealing with actual gum disease (persistent bleeding, receding gums, pain), it's worth seeing a dentist natural remedies alone won't reverse established periodontitis, and delays can lead to irreversible bone loss.

Q21 Can getting a dental scaling damage my teeth?

Dental scaling is generally very safe, but there are some legitimate concerns worth knowing about.

What scaling actually does.

Scaling removes calculus (tartar) and plaque from tooth surfaces and below the gumline. The instruments whether metal scalers or ultrasonic devices work on the deposits, not the tooth structure itself. Done correctly, it doesn't damage enamel.

Real but minor risks.

Temporary sensitivity The most common side effect. Removing buildup exposes tooth surfaces that were covered, making them feel sensitive to temperature for a few days to a couple of weeks. This resolves on its own.

Gum soreness or bleeding Normal, especially if there's existing inflammation. It settles within a few days.

Microscopic enamel roughness Aggressive or repeated ultrasonic scaling *can* create tiny surface irregularities, but research suggests this is clinically insignificant and the tooth remineralizes over time.

Root surface sensitivity Deep scaling (root planing, done for gum disease) goes below the gumline and can leave root surfaces temporarily sensitive, since roots lack the enamel protection crowns have.

When concerns are more valid.

Overly frequent scaling on already-healthy teeth with no buildup unnecessary and potentially abrasive over many years.

Improper technique An unskilled or rushed procedure can nick the root surface or traumatize gum tissue, though this is uncommon with trained dental professionals.

Pre-existing conditions If you have very thin enamel, dentinal hypersensitivity, or exposed roots already, you may feel effects more acutely.

Bottom line.

For most people, a routine scaling every 6 12 months is safe and *protective* tartar left in place causes far more damage (gum recession, bone loss, cavities) than the cleaning does. The temporary sensitivity afterward is normal, not a sign of damage.

If you're experiencing persistent sensitivity or pain weeks after a scaling, that's worth flagging to your dentist it could point to an exposed cavity, a cracked tooth, or unusually deep root exposure that needs follow-up.

Q22 Can dental scaling cause gaps between my teeth?

Dental scaling doesn't *create* gaps but it can make existing ones more visible, which often surprises people.

Here's what's actually happening:

Before scaling, tartar (calculus) builds up between and around your teeth, sometimes filling the spaces near the gumline. It can also cause gum inflammation, which makes the gums puff up and appear to close those spaces.

After scaling, two things change:

The tartar is removed, so any space it was occupying is now open.

Inflamed gums shrink back to their healthy size (this takes a few days to weeks).

The result is that gaps which were always there hidden under buildup or masked by swollen gums are suddenly exposed. It feels like something changed, but the gaps were pre-existing.

When should you be concerned?

If the gaps are large or your teeth feel noticeably looser after scaling, that's worth flagging to your dentist. Significant bone loss from gum disease (periodontitis) can leave real spacing once the inflammation resolves and that's not caused by the cleaning itself, but revealed by it.

What's normal:

Slight sensitivity for a few days.

Gums looking "lower" than before.

Small spaces near the gumline becoming visible.

All of that typically settles within 1 2 weeks as gums heal and tighten. If you're still bothered after that, a follow-up with your dentist to check gum attachment levels is the right move.

Q23 Do all gum diseases require the same treatment?

No gum diseases vary quite a bit in severity and type, and treatment is matched accordingly.

The main categories.

Gingivitis is the mildest and most common form. It's limited to the gum tissue itself (no bone loss yet) and is almost always reversible with:

A professional cleaning (scaling).

Improved daily brushing and flossing.

Sometimes an antibacterial rinse (e.g., chlorhexidine).

Periodontitis is more serious the infection has spread below the gumline, damaging the bone and connective tissue that hold teeth in place. Treatment scales with how far it's progressed:

Stage.	Typical Treatment.
Mild Moderate.	Scaling and root planing ("deep cleaning") removes tartar and bacteria from below the gumline.
Moderate Severe.	Deep cleaning local or systemic antibiotics.
Severe Advanced.	Surgical options flap surgery, bone grafts, guided tissue regeneration.

Necrotizing periodontal disease (NUG/NUP) is a distinct, aggressive form linked to immune compromise, severe stress, or malnutrition. It involves tissue death and requires urgent debridement, antibiotics (usually metronidazole), and pain management not just a standard cleaning.

Gum abscesses need immediate drainage, often combined with antibiotics and follow-up deep cleaning.

What drives the treatment choice.

Depth of gum pockets (measured by probing).

Amount of bone loss (seen on X-rays).

Presence of active infection.

Underlying health conditions (diabetes, immunosuppression, smoking all slow healing).

How you respond to initial treatment response to deep cleaning determines whether surgery is needed.

The upshot: a dentist or periodontist needs to actually assess the extent of disease before settling on a plan. What looks like "just bad gums" can range from a two-week fix with better brushing to months of specialist care.

Q24 Is gum treatment painful?

It depends on the type of treatment and how advanced the gum disease is, but here's an honest breakdown:

Mild treatments generally not painful.

Professional cleaning (scaling) for early gum disease is usually just uncomfortable, not painful. You might feel some sensitivity, especially if your gums are inflamed.

Routine check-ups and cleanings are well-tolerated by most people.

Deeper treatments can be uncomfortable.

Deep cleaning (scaling and root planing) goes below the gumline to remove tartar buildup. Dentists typically use local anesthesia, so you won't feel much during the procedure but expect soreness and sensitivity for a few days afterward.

The more inflamed or infected the gums are beforehand, the more tender the area tends to be post-treatment.

Surgical treatments more involved.

Procedures like flap surgery or bone grafts are done under local anesthesia (sometimes sedation), so the procedure itself is usually painless. Recovery involves swelling and discomfort for several days, managed with over-the-counter or prescribed pain relievers.

The bottom line.

Modern anesthesia and technique make gum treatment far more comfortable than most people expect. The fear of pain is usually worse than the reality and untreated gum disease causes far more pain and damage long-term (tooth loss, jawbone erosion, infections).

If pain anxiety is a concern, it's worth telling your dentist upfront. They can adjust the approach, offer sedation options, or walk you through what to expect step by step.

Q25 Can gum surgery cause tooth loss?

Yes, gum surgery can potentially lead to tooth loss, though it's not a common outcome and is usually the result of specific circumstances:

When gum surgery might contribute to tooth loss.

Pre-existing severe disease If periodontal disease has already caused significant bone loss around a tooth before surgery, that tooth may be too compromised to save. Surgery can sometimes reveal the full extent of the damage, leading to an extraction decision during or after the procedure.

Surgical complications Rare but possible: infection following surgery, damage to surrounding structures, or impaired blood supply can destabilize teeth that were previously stable.

Healing problems Patients with uncontrolled diabetes, immune disorders, or those who smoke heavily are at higher risk of poor healing, which can sometimes result in tooth loss.

Bone graft or implant failure Procedures involving grafts can occasionally fail, which may affect adjacent teeth.

The bigger picture.

Gum surgery is almost always performed *to prevent* tooth loss it's the treatment for advanced periodontal disease, which is one of the leading causes of tooth loss in adults. When done on appropriate candidates by a skilled periodontist, the goal and typical outcome is to preserve teeth that would otherwise be lost.

The risk calculus usually looks like this: untreated advanced gum disease near-certain eventual tooth loss; gum surgery small procedural risk with a much better long-term outcome.

If you're weighing whether to go ahead with a recommended procedure, the conversation to have with your periodontist is specifically about the current health of each tooth involved particularly how much bone support remains. That's the most reliable predictor of whether a tooth can be saved with surgery or whether extraction is the more realistic outcome regardless.

Q26 Can gum treatment make my teeth feel loose?

Yes, this is actually quite common and usually a good sign here's why:

Why Teeth Can Feel Loose After Gum Treatment.

The bone and tissue were already compromised. Gum disease (periodontitis) causes the bone and ligaments supporting your teeth to break down. The inflamed, swollen gum tissue was essentially "gripping" your teeth and masking how much support had already been lost. Once the infection and swelling go down after treatment, that artificial grip disappears and the actual state of the underlying support becomes apparent.

Scaling and root planing (deep cleaning) removes the tartar and bacteria from below the gumline. As the gums shrink back to a healthy, tighter fit, teeth can temporarily feel less anchored.

Is It Temporary?

Often, yes. As the gums heal and reattach to clean root surfaces over several weeks, the feeling typically stabilizes. Whether teeth remain loose long-term depends on:

How much bone was lost before treatment began.

How well you maintain oral hygiene going forward.

Whether the disease is stopped from progressing further.

When to Be Concerned.

If teeth feel *significantly* looser, are shifting visibly, or the sensation worsens rather than improves after a few weeks, that warrants a follow-up with your periodontist. It could mean the treatment needs reassessment, or that bone loss was more extensive than initially apparent.

The key takeaway: treatment didn't make the situation worse it revealed what was already there. Healthy gum tissue healing around the roots is the path back to stability, not further loosening.

Q27 Does tartar removal cause more tartar buildup?

This is a common concern people have after a dental cleaning, and the short answer is no tartar removal does not cause more tartar buildup. Here's what's actually going on:

Why the myth exists.

After a cleaning, teeth surfaces that were previously covered by tartar (calculus) are now exposed. Saliva and oral bacteria interact with those newly smooth surfaces differently than before, which can sometimes *feel* different. Some people interpret that as the teeth being "weaker" or more prone to buildup, but that's not what's happening biologically.

What actually drives tartar formation.

Tartar is mineralized dental plaque a biofilm of bacteria that hardens when minerals from saliva (mainly calcium and phosphate) deposit into it. The factors that determine how fast tartar builds up are:

Plaque control how consistently and effectively you brush and floss.

Saliva chemistry some people naturally have more mineral-rich saliva, making them faster tartar producers.

Diet sugary and starchy foods fuel the bacteria in plaque.

Genetics some individuals are simply more prone to rapid calculus formation.

None of these are affected by having tartar removed.

What the evidence says.

Scaling and root planing (professional tartar removal) actually makes the tooth surface *smoother*, which makes it harder for plaque to adhere and calculus to form not easier. Rough calculus deposits actually give plaque more surface area to cling to, so leaving tartar in place would accelerate buildup, not prevent it.

The practical upshot.

If you feel like tartar comes back faster after each cleaning, the likeliest explanations are that you're a naturally fast calculus producer (saliva chemistry), your home care has gaps, or you're more *aware* of the feeling now. The solution is better daily plaque removal brushing thoroughly twice a day and flossing not avoiding professional cleanings.

Q28 Are antibiotics enough to treat gum disease?

Antibiotics alone are generally not enough to treat gum disease they're a supporting tool, not a standalone cure.

Why antibiotics aren't the full answer.

Gum disease (periodontitis) is driven by bacterial biofilm specifically dental plaque and tartar that physically attach to teeth and below the gumline. Antibiotics circulate through your bloodstream or sit in your saliva, but they can't penetrate and break up that biofilm on their own. Without physically removing it, the infection persists and returns.

What actually treats gum disease.

The foundation of treatment is mechanical debridement cleaning performed by a dentist or hygienist:

Scaling and root planing (deep cleaning) scraping plaque and calculus from tooth surfaces above and below the gumline, then smoothing root surfaces so bacteria have less to cling to.

Surgical intervention for advanced cases, procedures like flap surgery or bone grafting to access deeper pockets and regenerate lost tissue.

Ongoing maintenance regular professional cleanings, usually every 3–4 months for people with periodontitis history.

Where antibiotics *do* help.

They're used as an adjunct, not a replacement, in specific situations:

Localized antibiotic delivery gels or microspheres (like doxycycline or minocycline) placed directly into gum pockets after deep cleaning to knock down residual bacteria.

Systemic antibiotics (oral pills) reserved for aggressive or rapidly progressing periodontitis, or when infection has spread beyond the gums. Common choices include metronidazole, amoxicillin, or a combination.

Acute gum abscesses antibiotics help control active, spreading infection alongside drainage.

The core principle.

Think of it like a wound that has gravel in it antibiotics can fight the infection, but until you physically remove the gravel, the wound won't heal. Gum disease is the same: the biofilm has to be mechanically removed first, and antibiotics help mop up what remains.

A periodontist or dentist can assess the severity of your situation and recommend the right combination of approaches.

Q29 After gum disease treatment, will I ever need treatment again?

Gum disease (periodontitis) is a chronic condition, which means it can be managed but not truly "cured." Here's what that means practically:

The honest answer: yes, ongoing care is needed.

Once you've had periodontitis, the bacteria and the susceptibility don't disappear they're kept in check. Without continued attention, the disease will return. Studies consistently show that patients who skip maintenance appointments see their disease recur within a few years.

What ongoing care looks like.

After active treatment (scaling and root planing, or surgery), you typically move into a supportive periodontal therapy (SPT) phase:

More frequent cleanings usually every 3 4 months rather than the standard 6, at least initially. This interval may relax over time if you stay stable.

Regular re-evaluation your periodontist or dentist checks pocket depths, bone levels, and gum health to catch any progression early.

Good home care brushing technique, flossing, and possibly adjuncts like interdental brushes or a water flosser matter more for you than for someone who's never had gum disease.

Factors that affect your long-term outlook.

Smoking significantly worsens prognosis quitting is the single biggest controllable factor.

Diabetes poorly controlled blood sugar makes gum disease harder to manage; the relationship runs both ways.

Genetics some people are simply more susceptible regardless of hygiene.

Your original severity more advanced disease at diagnosis generally means closer monitoring long-term.

The good news

With consistent maintenance, most people can keep their teeth for life. The research is pretty clear that patients who stick to their SPT schedule do dramatically better than those who don't so the investment in those 3-month appointments pays off.

Your periodontist will give you a personalized maintenance plan based on how your gums responded to treatment, which is the most reliable guide for your specific situation.

Q30 Is surgery always necessary for gum treatment?

No, surgery isn't always necessary for gum (periodontal) disease. Whether you need it depends largely on how advanced the condition is.

Non-Surgical Options (First-Line Treatment).

For mild to moderate gum disease, dentists typically start with non-surgical approaches:

Scaling and root planing (deep cleaning) The most common treatment. A hygienist removes plaque and tartar from below the gumline and smooths the root surfaces so gum tissue can reattach. Often done under local anesthesia.

Improved oral hygiene Better brushing, flossing, and regular cleanings can reverse *gingivitis* (early-stage gum disease) entirely.

Antibiotics Topical or oral antibiotics (like doxycycline or chlorhexidine gel) can be used alongside deep cleaning to control bacterial infection.

Laser therapy Some clinics use lasers to remove infected tissue with less discomfort and faster healing than traditional surgery.

When Surgery Becomes Necessary.

Surgery is typically recommended when:

Pockets are too deep (usually >5 6mm) for instruments to clean effectively.

Bone loss has occurred and needs to be reshaped or regenerated.

Gum recession is significant and needs grafting.

Non-surgical treatment hasn't resolved the infection after a few months.

Common surgical procedures include flap surgery (lifting the gum to clean underneath), bone grafts, and gum grafts.

The key principle is that periodontal treatment is staged you exhaust non-surgical options first and reassess. Many people with moderate gum disease respond well to deep cleaning alone, especially when caught early.

A periodontist (gum specialist) can probe pocket depths and take X-rays to give you a clearer picture of which route makes sense for your specific situation.
