# Supplementary material for: Comparative Evaluation of Large Language Models in Responding to Common Misconceptions Regarding Periodontal Disease and Oral Hygiene
Source: Diagnostics (Basel). 2026 Sep 28;16(19):3158. doi: 10.3390/diagnostics16193158 (PMC13664004; doi:10.3390/diagnostics16193158)
Supplement: Supplementary file 1 [file diagnostics-16-03158-s001.zip › Supplementary File S5 LLMs response stylistic elements removed.zip!/2. LLMs response/Copilot.pdf]

Q1 Do gum diseases only affect elderly people?

No gum diseases do not only affect elderly people. They can occur at any age, including in children and young adults, though the risk increases with age due to cumulative plaque buildup and other health factors.

Who Can Get Gum Disease?

Children and teens: Gingivitis, the earliest stage of gum disease, is common in younger people, especially during puberty when hormonal changes make gums more sensitive.

Adults: Nearly half of adults in the U.S. have some form of gum disease, making it one of the most widespread oral health issues.

Elderly: Older adults are more likely to develop advanced periodontitis due to long-term plaque buildup, reduced immunity, and other chronic health conditions.

Key Risk Factors Beyond Age.

Poor oral hygiene: Not brushing and flossing regularly allows plaque to harden into tartar, leading to gum inflammation.

Smoking: Tobacco use is one of the strongest risk factors for gum disease.

Hormonal changes: Puberty, pregnancy, and menopause can increase gum sensitivity.

Chronic diseases: Conditions like diabetes, heart disease, and autoimmune disorders raise susceptibility.

Genetics: Family history can predispose individuals to gum disease.

Symptoms to Watch For.

Bleeding gums when brushing or flossing.

Swollen, red, or tender gums.

Persistent bad breath.

Gum recession (teeth appearing longer).

Loose teeth or changes in bite alignment .

Prevention Tips.

Brush twice daily with fluoride toothpaste.

Floss or use interdental brushes every day.

Visit a dentist regularly (every 6–12 months, or more often if at risk).

Avoid smoking and maintain a balanced diet rich in vitamin C.

Why This Matters.

Gum disease is not just about oral health it’s linked to serious systemic conditions like heart disease, diabetes complications, and even adverse pregnancy outcomes. Early detection and consistent oral care can prevent progression from mild gingivitis to severe periodontitis.

Q2 Is it normal if my gums bleed when I brush my teeth?

Bleeding gums when brushing are *not normal* and usually signal gum disease (gingivitis or periodontitis), though they can also be linked to vitamin deficiencies, hormone changes, or certain medical conditions. If bleeding persists for more than two weeks, you should see a dentist to rule out serious causes and prevent complications.

Common Causes of Bleeding Gums.

Gingivitis: Early-stage gum disease caused by plaque buildup. Symptoms include redness, swelling, and bleeding when brushing or flossing.

Periodontitis: Advanced gum disease that can lead to tooth loss if untreated.

Poor oral hygiene: Not brushing/flossing properly allows bacteria to irritate gums.

Vitamin deficiencies: Lack of vitamin C or K can cause gum bleeding.

Hormonal changes: Pregnancy, puberty, or menopause can make gums more sensitive.

Medical conditions: Diabetes, blood-clotting disorders, or even leukemia may present with gum bleeding.

Medications: Blood thinners increase bleeding risk.

What You Can Do at Home.

Brush gently with a soft-bristled toothbrush 2 3 times daily.

Floss once a day to remove plaque between teeth.

Use antibacterial mouthwash or warm saltwater rinses.

Avoid smoking, which worsens gum disease.

Maintain a balanced diet rich in vitamins C and K.

When to See a Dentist.

If bleeding lasts more than 2 weeks.

If you notice swollen, painful gums, bad breath, or loose teeth.

If bleeding occurs without brushing or eating.

If you have other symptoms (fatigue, unexplained bruising, or frequent infections), which may point to systemic health issues.

Risks of Ignoring Bleeding Gums.

Progression from gingivitis to periodontitis, leading to tooth loss.

Increased risk of systemic health issues (diabetes complications, cardiovascular disease).

Persistent discomfort and bad breath.

Q3 Can I get gum disease even if I brush my teeth regularly?

Yes gum disease can still develop even if you brush regularly. Brushing is essential, but gum disease (gingivitis and periodontitis) is often caused by plaque and tartar buildup in areas brushing alone cannot reach, such as between teeth and under the gumline. Flossing, professional cleanings, and managing risk factors like smoking, diabetes, or genetics are equally important.

Why Gum Disease Can Occur Despite Brushing.

Plaque buildup: Plaque forms quickly after eating and can harden into tartar, which brushing cannot remove. Only a dentist can clean tartar.

Flossing importance: Brushing misses the tight spaces between teeth where bacteria thrive. Daily flossing or interdental brushes are crucial.

Risk factors: Smoking, hormonal changes, diabetes, poor nutrition, and genetics can increase susceptibility even with good brushing habits.

Professional cleanings: Dentists recommend checkups every 6 12 months. If you have risk factors, more frequent visits may be needed.

Symptoms to Watch For.

Bleeding gums when brushing or flossing.

Swollen, red, or tender gums.

Persistent bad breath.

Receding gums (teeth look longer).

Loose teeth or changes in bite.

If you notice these, see a dentist promptly early gum disease (gingivitis) is reversible, but advanced periodontitis can cause permanent bone and tooth loss.

Prevention Tips.

Brush twice daily for 2 minutes with fluoride toothpaste.

Floss or use interdental brushes once daily.

Avoid smoking and limit sugary foods.

Maintain a balanced diet with vitamin C.

Schedule regular dental checkups.

Quick Comparison: Brushing vs. Full Oral Care.

Practice.	Effectiveness Against Gum Disease.
Brushing only.	Removes surface plaque but misses between teeth and gumline.
Brushing Flossing.	Cleans between teeth, reduces risk significantly.
Professional cleaning.	Removes tartar, essential for long-term prevention.
Lifestyle management.	Reduces systemic risks (smoking, diabetes, poor diet).

Q4 Can I have gum disease even if my gums don't hurt?

Yes gum disease can develop even without pain. In fact, most people don’t feel pain in the early stages, so it often goes unnoticed until more serious symptoms like bleeding gums, bad breath, or loose teeth appear.

Why Gum Disease Can Be Painless.

Early stage gingivitis: Causes redness, swelling, and bleeding, but usually no pain.

Periodontitis: More advanced gum disease that damages bone and tissue. Pain may occur later, but many people only notice subtle changes like receding gums or shifting teeth.

Silent progression: Gum disease often advances slowly, with inflammation and tissue damage happening without obvious discomfort.

Warning Signs to Watch For.

Even without pain, gum disease may show:

Bleeding gums when brushing or flossing.

Persistent bad breath or bad taste.

Swollen or discolored gums (red, purple, or dark).

Receding gums making teeth look longer.

Loose teeth or changes in bite alignment.

Risks of Ignoring Gum Disease.

Tooth loss: Advanced gum disease can destroy bone support.

Systemic health issues: Linked to heart disease, diabetes, and even pregnancy complications.

Immune strain: Chronic inflammation can weaken your body's defenses.

Prevention Care.

Brush twice daily with fluoride toothpaste.

Floss or use interdental brushes once daily.

Get regular dental check-ups (every 6–12 months).

Avoid smoking or vaping, which increases risk.

Maintain a balanced diet with vitamin C for gum health.

Key Takeaway.

You can have gum disease without pain, so don't rely on discomfort as a warning sign. The safest approach is regular dental visits and paying attention to subtle symptoms like bleeding or gum changes.

Q5 Can gum disease get better without treatment?

No, gum disease does not get better on its own without treatment. Early-stage gum disease (gingivitis) can be reversed with professional cleaning and improved oral hygiene, but once it progresses to periodontitis, it cannot heal naturally and will worsen without dental care. Untreated gum disease can lead to tooth loss, bone damage, and even systemic health problems.

Stages of Gum Disease.

Stage.   Symptoms.   Can It Improve Without Treatment?

Gingivitis. Red, swollen gums, bleeding when brushing/flossing.   Yes, reversible with professional cleaning and good oral hygiene.

Mild Periodontitis. Gum pockets form, early bone loss. No, requires treatment (scaling/root planing).

Moderate Periodontitis. Gum recession, pus, bad breath, loose teeth. No, irreversible damage without dental intervention.

Advanced Periodontitis. Severe bone loss, teeth loosening/falling out No, only manageable with. surgery or grafting.

Risks of Leaving Gum Disease Untreated.

Tooth loss due to bone destruction.

Chronic bad breath and painful chewing.

Systemic health issues: linked to heart disease, diabetes, and pregnancy complications.

Permanent gum recession that cannot regrow naturally.

What You Can Do.

Brush twice daily with fluoride toothpaste.

Floss or use interdental brushes every day.

Get professional cleanings every 6 12 months (more often if at risk).

Quit smoking and maintain a healthy diet rich in vitamin C.

See a dentist immediately if gums bleed, swell, or teeth feel loose.

Q6 Are gum diseases rare?

Gum diseases are not rare at all in fact, they are among the most common oral health problems worldwide. Nearly half of adults in the United States have some form of gum disease, and it is also widespread in Türkiye and other regions.

How Common Are Gum Diseases?

Prevalence: About 50% of adults in the U.S. have gum disease. Similar rates are seen globally, making it one of the leading causes of tooth loss.

Stages:

Gingivitis: Early stage, reversible with good oral hygiene.

Periodontitis: Advanced stage, can cause bone loss and tooth loss.

Risk factors: Smoking, diabetes, poor oral hygiene, genetic predisposition, hormonal changes (pregnancy, menopause), and stress.

Symptoms to Watch For.

Bleeding gums when brushing or flossing.

Swollen or red gums.

Bad breath.

Loose teeth or gum recession.

Painful chewing.

Why It Matters.

Gum disease is linked not only to tooth loss but also to systemic health problems such as heart disease, diabetes complications, and pregnancy risks.

Untreated gum disease can progress silently, often without pain in early stages, which makes regular dental checkups essential.

Prevention Care.

Daily brushing (2 3 times) and flossing.

Regular dental checkups (every 6 12 months, or more often if at risk).

Quit smoking tobacco use is a major risk factor.

Healthy diet with sufficient vitamin C and balanced nutrition.

Professional cleaning to remove tartar that brushing cannot.

Key Takeaway.

Gum diseases are very common, not rare, and they can be prevented or managed with good oral hygiene and regular dental visits. Early detection (gingivitis) is reversible, but advanced stages (periodontitis) require professional treatment.

Q7 Is it normal to lose teeth because of gum disease as you get older?

Yes, losing teeth due to gum disease (periodontitis) is common in older adults, but it is *not inevitable*. With proper oral hygiene and regular dental care, most people can prevent gum disease from progressing to the point of tooth loss.

Why Gum Disease Causes Tooth Loss.

Periodontitis is a severe gum infection that damages the soft tissue and bone supporting teeth.

If untreated, gums recede, bone deteriorates, and teeth loosen until they fall out or require extraction.

Gum disease is the leading cause of tooth loss in adults, more than cavities or trauma.

How Common Is Tooth Loss?

178 million adults in the U.S. have lost at least one tooth.

40 million adults have lost all their teeth.

Gum disease is the primary cause of this, especially in older populations.

Risk Factors That Increase Tooth Loss.

Smoking or tobacco use (doubles risk).

Poor oral hygiene (infrequent brushing/flossing).

Chronic conditions: diabetes, rheumatoid arthritis, Crohn’s disease.

Hormonal changes (menopause, pregnancy).

Genetics (family history of gum disease).

Dry mouth from medications or autoimmune diseases.

Prevention Management.

Brush twice daily with fluoride toothpaste.

Floss once daily to remove plaque between teeth.

Regular dental checkups every 6 12 months (more often if at risk).

Quit smoking to improve gum healing and reduce risk.

Professional cleaning to remove tartar that brushing cannot.

Treat early signs (bleeding gums, bad breath, loose teeth) promptly.

Treatment Options if Teeth Are Lost.

Dental Implants. Closest to natural teeth, long-lasting.

Bridges. Supported by adjacent teeth, lasts 5–15 years.

Dentures. Replace multiple teeth, lasts 7–10 years.

Replacing lost teeth prevents jaw bone loss, shifting teeth, and bite problems.

Key Takeaway.

Tooth loss from gum disease is preventable, not a normal part of aging. If you notice bleeding gums, bad breath, or loose teeth, see a dentist immediately. Early treatment can stop progression and save your teeth.

Q8 If gum disease runs in my family, will I definitely get it too?

No, having gum disease in your family does not mean you will definitely get it genetics increase your risk but lifestyle and oral care habits play an equally important role. With consistent brushing, flossing, and regular dental visits, you can significantly reduce the chance of developing gum disease even if it runs in your family.

Genetics vs. Lifestyle in Gum Disease.

Genetic predisposition: Research shows that genetics account for 30–50% of gum disease susceptibility. Variations in immune-related genes (like IL-1 and IL-6) can make gums more reactive to plaque bacteria.

Family history: If close relatives had severe gum disease or lost teeth early, your dentist should consider you higher risk.

Environmental factors: Oral hygiene, smoking, diabetes, diet, and stress account for the other 50% or more of risk. Even without genetic predisposition, poor habits can cause gum disease.

Signs You May Be Genetically Prone.

Bleeding gums despite good brushing habits.

Rapid inflammation with small amounts of plaque.

Early tooth loss in family history.

Frequent deep cleanings recommended by your dentist.

Prevention Strategies.

Daily hygiene routine: Brush twice daily with fluoride toothpaste, floss once daily, and consider antimicrobial rinses.

Regular dental visits: Standard is every 6 months, but with family risk, every 3–4 months may be advised.

Lifestyle management: Quit smoking, control blood sugar if diabetic, eat a balanced diet, and manage stress.

Specialist care: A periodontist can assess your genetic risk and tailor a prevention plan.

Risks Trade-offs.

Genetics load the gun, environment pulls the trigger: Even with high genetic risk, gum disease is not inevitable. Conversely, poor hygiene can cause gum disease even without genetic predisposition.

Genetic testing: Some clinics offer saliva-based tests for gum disease risk, but results are not definitive. They are best used alongside clinical exams.

Key Takeaway.

If gum disease runs in your family, you should treat your gums as “high-risk” and be proactive. Genetics raise your baseline risk, but consistent oral hygiene, regular professional care, and healthy lifestyle choices can prevent gum disease from developing or progressing.

Q9 Can stress make me more likely to get gum disease?

Yes chronic stress can increase your risk of gum disease. Elevated stress hormones like cortisol weaken your immune system, promote inflammation in the gums, and encourage harmful bacteria to thrive, making gum tissues more vulnerable to infection and damage.

How Stress Affects Gum Health.

Cortisol elevation: Chronic stress keeps cortisol levels high, which directly promotes gum inflammation and slows tissue repair.

Immune suppression: Stress reduces the activity of immune cells (like neutrophils), allowing oral bacteria to cause more damage.

Reduced saliva: Stress decreases saliva flow, removing a natural defense against bacteria and acid.

Blood flow changes: Circulation is redirected away from gums during stress, slowing healing and increasing sensitivity.

Behavioral factors: Stress often leads to teeth grinding (bruxism), skipped brushing, poor diet, smoking, or alcohol use all of which worsen gum health.

Gum Problems Linked to Stress.

Gingivitis: Early gum inflammation can progress faster under stress.

Periodontitis: Severe gum disease with bone loss is more likely when stress is chronic.

Bleeding gums: Stress-driven inflammation makes gums bleed more easily when brushing.

Bruxism-related damage: Grinding teeth under stress can cause gum recession and loosen teeth.

Dry mouth: Reduced saliva increases infection risk and bad breath.

Protecting Your Gums During Stress.

Consistent oral hygiene: Brush twice daily, floss, and use antibacterial mouthwash.

Stress management: Practices like yoga, meditation, deep breathing, and regular exercise lower cortisol.

Healthy diet: Eat foods rich in vitamin C and calcium; avoid excess sugar and processed foods.

Hydration: Drink plenty of water to combat dry mouth.

Dental visits: Regular check-ups help catch gum problems early, especially during stressful periods.

Nightguards: If you grind your teeth, a dentist can provide a protective device.

Risks Considerations.

Stress is an independent risk factor for gum disease, not just a result of poor hygiene.

Even if you improve brushing and flossing, healing may be slower if cortisol remains high.

Severe stress can trigger acute gum conditions like necrotizing ulcerative gingivitis (painful, bleeding gums with tissue breakdown).

Q10 Do gum diseases only affect the mouth and teeth?

No gum diseases primarily affect the mouth and teeth, but they can also have serious consequences for overall health, including links to heart disease, diabetes, respiratory illness, and pregnancy complications. This means untreated gum disease is not just a dental issue but a systemic health risk.

Local Effects of Gum Disease.

Gingivitis: Early stage with red, swollen gums that bleed easily. Reversible with good oral hygiene.

Periodontitis: Advanced infection damaging gum tissue and bone, leading to loose teeth and tooth loss.

Symptoms: Bad breath, gum recession, pus around gums, painful chewing, and shifting teeth.

Systemic Health Connections.

Research shows gum disease bacteria and inflammation can spread beyond the mouth:

Heart disease: Oral bacteria can enter the bloodstream, contributing to arterial plaque and cardiovascular problems.

Diabetes: Gum disease makes blood sugar harder to control; diabetes also increases gum disease risk.

Respiratory disease: Inhaled oral bacteria may worsen lung conditions.

Pregnancy complications: Associated with preterm birth and low birth weight.

Autoimmune conditions: Links with rheumatoid arthritis and Crohn’s disease.

Comparison Table: Local vs Systemic Effects.

Local Effects.	Systemic Effects.
Gum swelling, bleeding.	Increased risk of heart disease.
Tooth loosening, loss.	Poor diabetes control.
Bad breath, abscesses.	Respiratory infections.

Gum recession.   Pregnancy complications.

Risks Prevention.

Risks: Smoking, poor oral hygiene, stress, hormonal changes, genetics, and chronic illnesses.

Prevention: Brush twice daily, floss, avoid tobacco, eat a balanced diet, and get regular dental checkups.

Treatment: Scaling, root planing, gum surgery, or bone grafting depending on severity.

Q11 Will a hard-bristled toothbrush clean my teeth better?

Hard-bristled toothbrushes can remove plaque effectively, but they are *not recommended* for daily use because they increase the risk of gum damage, enamel wear, and gingival recession. Dentists generally advise using a soft or medium-bristled toothbrush for safe and effective cleaning.

Cleaning Effectiveness.

Hard bristles: They can scrape away plaque and stains more aggressively, which may feel like a deeper clean.

Soft bristles: Designed to flex and reach between teeth and along the gumline, removing plaque without damaging tissues.

Medium bristles: A balance between cleaning power and safety, often recommended for most adults.

Risks of Hard-Bristled Toothbrushes.

Gum damage: Studies show hard bristles cause more gingival lesions compared to soft brushes. This can lead to gum recession and exposed roots.

Enamel wear: Excessive scrubbing with hard bristles can erode enamel over time, increasing sensitivity.

Oral discomfort: Hard bristles may feel harsh, especially for people with sensitive gums or dental work.

Dentist Recommendations.

Soft bristles are safest: Most dental associations recommend soft-bristled brushes for everyday use.

Technique matters more than stiffness: Gentle, circular brushing for 2 minutes twice daily is more effective than scrubbing hard.

Replace regularly: Change your toothbrush every 3 4 months, or sooner if bristles are frayed.

Comparison Table.

Toothbrush Type.	Cleaning Power	Gum Safety.	Enamel Safety.	Best For.
Hard bristles.	High.	Low.	Low.	Heavy stain removal (occasional use).
Medium bristles.	Moderate.	Moderate.	Moderate	General use if gums are healthy.
Soft bristles.	Good.	High.	High.	Daily brushing, sensitive gums.

Q12 Should I stop brushing my teeth for a few days if my gums are bleeding?

Bleeding gums can feel alarming, but stopping brushing altogether is usually not the right move. In fact, plaque buildup from not brushing can make gum bleeding worse. That said, there are some important points to keep in mind:

Gum disease: Bleeding gums are often an early sign of gingivitis, which develops when plaque irritates the gum tissue. Regular brushing and flossing help prevent this.

Brushing technique: Brushing too aggressively or with a hard-bristled toothbrush can cause or worsen bleeding. Dentists recommend using a soft-bristled brush and gentle circular motions.

Oral hygiene: Maintaining daily brushing and flossing is key, but if gums are sore, rinsing with an antibacterial mouthwash or warm salt water can help soothe irritation.

Professional checkup: Persistent or heavy bleeding should be evaluated by a dentist, as it could signal gum disease, vitamin deficiencies, or other health issues.

The safest step is to keep brushing gently rather than stopping, and to schedule a dental visit if the bleeding continues. If you notice swelling, pain, or bleeding that doesn't improve, please consult a qualified healthcare professional promptly.

Would you like me to walk you through home remedies that can ease gum irritation, or focus more on dentist treatments?

Q13 Do I need to brush my teeth after every meal to keep my gums healthy?

You don't need to brush after every meal brushing twice a day with fluoride toothpaste (especially before bed) and flossing once daily is enough to keep gums healthy. Brushing more often isn't harmful if done gently, but the key is *quality of brushing* and consistent plaque removal, not frequency.

Recommended Routine for Gum Health.

Brush twice Daily.

Once before bed (most important, as saliva decreases during sleep).

Once more during the day, ideally morning.

Use fluoride toothpaste and a soft-bristled brush angled at 45° to the gumline.

Floss once Daily.

Removes plaque and food particles between teeth where brushes can't reach.

Prevents tartar buildup and gingivitis.

Rinse and scrape.

Rinse with water or fluoride mouthwash.

Tongue scraping reduces bacteria and bad breath.

Dental check-ups.

Twice yearly is ideal, but at least once annually.

Professional cleaning removes hardened tartar.

Brushing Frequency: Pros Cons.

Brushing twice Daily.	Brushing after every meal.
Recommended by dentists worldwide.	Not necessary for most people.
Prevents plaque buildup effectively.	May help if you eat sticky/sugary foods.
Less risk of gum abrasion.	Overbrushing can irritate gums.
Sustainable long-term habit.	Harder to maintain consistently.

Risks Trade-offs.

Overbrushing: Brushing too often or too hard can wear down enamel and irritate gums.

Skipping bedtime brushing: This is the worst habit, as bacteria multiply overnight.

Diet impact: Frequent sugary snacks increase risk of gum disease; brushing after them may help.

Q14 Even if I brush my teeth regularly, do I still need to get my gums checked?

Yes even if you brush your teeth regularly, it’s still important to have your gums checked. Brushing is excellent for removing plaque from the surfaces of your teeth, but gum health involves more than just brushing.

Here’s why gum checks matter:

Gum disease: Conditions like gingivitis or periodontitis can develop silently, sometimes without pain, even if your teeth look clean.

Plaque and tartar: Brushing doesn’t always remove buildup under the gumline. Professional cleanings target these areas.

Systemic health: Gum disease has been linked to broader health issues, including heart disease and diabetes.

Early detection: Dentists can spot subtle changes in gum color, texture, or bleeding that you might overlook.

Preventive care: Regular checkups help prevent small gum issues from becoming major problems.

So, while brushing and flossing are your daily defense, professional gum checks are like a safety net they catch what you can’t see or reach.

Q15 Is brushing my teeth once a day enough to keep my mouth healthy?

Brushing your teeth only once a day is *not enough* to keep your mouth healthy long-term. Dentists and the American Dental Association recommend brushing *twice daily* with fluoride toothpaste to prevent cavities, gum disease, and tartar buildup. If you must choose one time, brushing before bed is more critical than in the morning.

Why Twice Daily Matters.

Plaque removal: Plaque forms constantly; brushing twice interrupts its growth before it hardens into tartar.

Cavity prevention: Bacteria feed on food particles and sugars, producing acids that erode enamel.

Gum health: Infrequent brushing allows bacteria to irritate gums, leading to gingivitis and periodontitis.

Fresh breath: Twice-daily brushing reduces odor-causing bacteria.

Comparison: Once vs Twice Daily Brushing.

Brushing Frequency.	Benefits.	Risks.
Once Daily.	Removes some plaque and food debris.	Higher risk of cavities, gum disease, bad breath, tartar buildup.
Twice Daily.	Optimal plaque control, cavity prevention, gum health.	Minimal risks if done gently with proper technique.
More than 3x Daily.	May help in special cases (braces, smoking).	Can damage enamel/gums if too aggressive.

Morning vs Night Brushing.

Morning brushing: Removes bacteria accumulated overnight, freshens breath.

Night brushing: *Most important* saliva production decreases while you sleep, so bacteria thrive unchecked if you skip brushing before bed.

Complete Oral Care Routine.

Flossing daily: Removes plaque between teeth.

Mouthwash use: Reaches areas brushing misses.

Healthy diet: Limit sugary foods/drinks.

Dental checkups: Every 6 months for cleaning and early detection.

Risks of Brushing Only Once.

Plaque hardens into tartar requires professional removal.

Gum inflammation (gingivitis) can progress to periodontitis.

Cavities and tooth decay may lead to fillings, crowns, or extractions.

Bad breath bacteria linger overnight.

Key Takeaway.

Brushing once a day occasionally won't ruin your teeth, but making it a habit will. For lifelong oral health, brush twice daily, floss once daily, and see your dentist regularly.

Q16 Can flossing cause gum recession?

Flossing itself does not cause gum recession when done correctly, it actually helps prevent it. Gum recession is usually caused by periodontal disease, aggressive brushing, genetics, or habits like teeth grinding, but improper flossing technique (snapping or sawing the floss) can irritate gums and make recession worse.

Proper Role of Flossing.

Correct flossing technique: Gentle, controlled movements clean plaque between teeth and under the gumline without harming tissue.

Prevents gum disease: Daily flossing reduces plaque buildup, lowering risk of gingivitis and periodontitis the main drivers of gum recession.

Cannot reverse recession: Once gum tissue is lost, flossing cannot regrow it, but it prevents further damage.

Causes of Gum Recession (Not Flossing).

Aggressive brushing: Hard bristles or too much force wear away gum tissue.

Periodontal disease: Bacterial infection destroys gum and bone support.

Genetics: Some people naturally have thinner gums.

Bruxism: Grinding/clenching teeth puts stress on gums.

Tobacco use: Smoking or chewing tobacco damages gum tissue and slows healing.

Misaligned teeth: Uneven pressure points can lead to localized recession.

Safe Flossing Technique.

Use 18 cm of floss, wrapping around middle fingers.

Guide floss gently between teeth never snap.

Curve into a “C” shape around each tooth.

Slide up and down against the tooth surface, not directly into gums.

Use a fresh section for each tooth.

Alternatives: Water flossers or interdental brushes can help if string floss feels difficult.

Risks Misconceptions.

Improper flossing (snapping, sawing) can traumatize gums and mimic recession symptoms.

Bleeding gums after flossing usually indicate inflammation from plaque, not damage from flossing.

Stopping flossing due to bleeding worsens gum health gentle, consistent flossing reduces inflammation over time.

When to Seek Professional Help.

Persistent gum recession despite proper flossing.

Bleeding gums that don’t improve after 2 weeks.

Tooth sensitivity or exposed roots.

Loose teeth or deep gum pockets.

Dentists may recommend scaling and root planing or gum graft surgery for advanced cases.

Q17 Do electric toothbrushes damage the gums more than regular toothbrushes?

Electric toothbrushes do *not* damage gums more than manual toothbrushes in fact, studies show they generally improve gum health by reducing plaque and gingivitis more effectively. The only risk is if you brush too hard, but many modern electric models include pressure sensors to prevent this.

Evidence from Clinical Studies.

Cochrane Review (2014): Electric toothbrushes reduced plaque by 21% and gingivitis by 11% compared to manual brushes over 3 months.

Meta-analyses (2020–2024): Oscillating-rotating brushes consistently showed fewer bleeding sites and better gum health outcomes.

Clinical trials: Users of electric brushes had significantly greater reductions in gum inflammation and plaque within 5 8 weeks.

Sonic toothbrushes: Removed more plaque in hard-to-reach areas, with fewer gum abrasions reported.

Comparison Table.

Electric toothbrush.	Manual toothbrush.
Removes more plaque (up to 20%).	Effective if technique is correct.
Reduces gingivitis and bleeding.	Can maintain gum health with proper use.
Pressure sensors prevent over-brushing.	Risk of brushing too hard without feedback.
Easier for people with braces or limited mobility.	Requires more manual dexterity.
Higher cost, needs charging.	Cheaper, no batteries.

Risks Considerations.

Over-brushing pressure: Both manual and electric brushes can cause gum recession if pressed too hard. Electric models with pressure sensors reduce this risk.

Technique matters: Holding the brush at a 45° angle to the gum line is key for both types.

Brush head replacement: Every 2 3 months, or sooner if bristles fray.

Practical Advice.

If you have sensitive gums or gum disease risk, an oscillating-rotating electric toothbrush is recommended.

If you prefer simplicity and lower cost, a soft-bristled manual toothbrush used correctly is still effective.

Always pair brushing with flossing or interdental cleaning for complete gum protection.

Key Takeaway.

Electric toothbrushes are generally *better for gum health* than manual ones, provided you use them correctly and don't apply excessive pressure. They are especially useful for people with braces, arthritis, or difficulty maintaining consistent brushing technique.

Q18 Would using mouthwash alone be enough without brushing my teeth?

No using mouthwash alone is not enough to maintain healthy teeth and gums. Mouthwash can freshen breath and kill some bacteria, but it does not physically remove plaque and food debris the way brushing and flossing do. Regular brushing twice a day is essential to prevent cavities, gum disease, and enamel damage.

Why Mouthwash Alone Isn't Sufficient.

Plaque removal: Mouthwash cannot scrape away plaque; brushing with fluoride toothpaste is the only way to physically remove it.

Cavity prevention: Without brushing, food particles and sugars remain, increasing the risk of tooth decay.

Gum health: Mouthwash may reduce gingivitis temporarily, but brushing is needed to clean near the gumline.

Bad breath: Mouthwash masks odor but doesn’t treat the root cause (like bacteria buildup or gum disease).

When Mouthwash Helps.

Quick freshening: Useful if you can’t brush after meals or while traveling.

Supplement to brushing: Adds antimicrobial protection when used alongside brushing and flossing.

Special conditions: Antiseptic mouthwash can help reduce gum inflammation between brushing sessions.

Comparison Table

Aspect.	Brushing.	Mouthwash Alone.
Plaque removal.	Physically scrapes plaque.	Does not remove plaque.
Cavity prevention.	Proven to prevent cavities.	Limited effect.
Gum disease.	Cleans gumline.	May reduce inflammation but not cure.
Breath freshness.	Removes odor-causing bacteria.	Masks odor temporarily.
Long-term oral health.	Essential.	Insufficient.

Risks of Relying Only on Mouthwash.

Plaque tartar buildup leads to cavities and gum disease.

Dry mouth from alcohol-based rinses worsens bad breath.

Enamel erosion with frequent use without brushing.

Hidden problems masks symptoms of cavities or gum disease without treating them.

Best Practice for Oral Hygiene.

Brush twice daily for 2 minutes with fluoride toothpaste.

Floss once daily to remove debris between teeth.

Use alcohol-free mouthwash as a supplement, not a replacement.

Replace toothbrush every 3 4 months.

Q19 After I quit smoking, my gums started bleeding. Is that normal?

Yes, bleeding gums after quitting smoking can be normal. Smoking often masks gum disease by restricting blood flow, so when you quit, improved circulation reveals underlying inflammation. This bleeding is usually temporary, but it can also signal gum disease that requires dental care. If bleeding is persistent or severe, you should see a dentist promptly.

Why Gums Bleed After Quitting Smoking.

Nicotine effect: Nicotine constricts blood vessels, reducing blood flow and hiding gum inflammation.

Improved circulation: Once you quit, blood vessels widen, restoring normal blood flow. This unmasks gum disease symptoms like redness, swelling, and bleeding.

Underlying gum disease: Many smokers already have gingivitis or periodontitis, but symptoms were suppressed. Quitting allows the immune system to fight infection, which can cause temporary bleeding.

Recovery Timeline.

First 1 4 weeks: Bleeding often increases as circulation improves.

1 3 months: With good oral hygiene, bleeding usually decreases significantly.

Up to 6 months: Advanced gum disease may take longer to heal.

What You Can Do.

Gentle brushing: Use a soft-bristled toothbrush and avoid scrubbing too hard.

Flossing routine: Daily flossing removes plaque between teeth.

Salt water rinse: Helps soothe gums and reduce bacteria.

Dental check-up: Professional cleaning may be needed to remove tartar and treat gum disease.

Healthy diet: Fruits, vegetables, and hydration support healing.

When to Seek Help.

See a dentist immediately if you notice:

Persistent bleeding beyond 2 months.

Severe pain or swelling.

Pus at the gumline.

Loose teeth or gum recession.

Key Takeaway.

Bleeding gums after quitting smoking are usually a sign of healing, not new damage. However, they can reveal pre-existing gum disease. Maintaining good oral hygiene and visiting a dentist will help ensure your gums recover fully.

Q20 Can natural products effectively keep my gums healthy?

Yes certain natural products like aloe vera, neem, clove, and tea tree oil can help reduce gum inflammation, fight bacteria, and support healing, but they should complement (not replace) professional dental care and daily brushing/flossing. If you have persistent gum problems, it’s important to consult a dentist to rule out serious conditions like periodontitis.

Natural Products for Gum Health.

Aloe Vera.

Properties: Anti-inflammatory, antimicrobial, soothing.

Use: Apply food-grade gel directly to gums for 10 15 minutes after brushing.

Benefit: Reduces redness, swelling, and bleeding from gingivitis.

Neem.

Properties: Antibacterial, anti-inflammatory.

Use: Found in natural toothpastes or used as chew sticks.

Benefit: Helps fight plaque and gingivitis.

Tea Tree Oil.

Properties: Antibacterial, antifungal.

Use: In specialized toothpastes or diluted rinses.

Benefit: Targets harmful bacteria linked to gum disease.

Clove.

Properties: Analgesic, antimicrobial.

Use: Clove oil applied sparingly or used in rinses.

Benefit: Relieves gum pain and controls bacteria.

Sage.

Properties: Anti-inflammatory, antimicrobial.

Use: Tea rinse or mouthwash.

Benefit: Tightens gums and combats bacteria.

Comparison Table.

Natural Product.	Key Properties.	Best Use.	Main Benefit.
Aloe Vera.	Anti-inflammatory, antimicrobial.	Gel on gums.	Soothes gingivitis.
Neem.	Antibacterial, anti-inflammatory.	Toothpaste, chew stick.	Fights plaque.
Tea Tree Oil.	Antibacterial, antifungal.	Toothpaste, rinse.	Reduces bacteria.
Clove.	Analgesic, antimicrobial.	Oil, rinse.	Pain relief.
Sage.	Anti-inflammatory, antimicrobial.	Tea rinse.	Tightens gums.

Risks Considerations.

Essential oils (like tea tree or clove) must be diluted; concentrated use can irritate gums.

Natural remedies are supportive, not curative they won’t reverse advanced gum disease.

Consistency matters occasional use won’t provide lasting benefits.

Professional care is essential dentists can detect early gum disease before it worsens.

Q21 Can getting a dental scaling damage my teeth?

Dental scaling does not damage healthy teeth when performed correctly; it mainly removes tartar and protects gums. However, minor side effects like temporary sensitivity, gum soreness, or slight bleeding can occur, especially after deep scaling. The long-term benefits for gum and overall health far outweigh these small risks.

What Dental Scaling Does.

Tartar removal: Scaling eliminates hardened plaque that brushing and flossing cannot remove.

Gum disease prevention: By cleaning below the gumline, scaling reduces inflammation and prevents bone loss.

Systemic health benefits: Treating gum disease lowers risks linked to heart disease and diabetes.

Possible Risks and Side Effects.

Tooth sensitivity: Temporary sensitivity to hot, cold, or sweet foods is common.

Gum tenderness: Mild soreness or swelling may last 1 2 days.

Minor bleeding: Especially if gums were inflamed before treatment.

Surface roughness: Ultrasonic tools can cause microscopic roughness on fillings or crowns, but enamel is generally unaffected.

Rare complications: Aggressive technique may irritate exposed roots or restoration margins, leading to sensitivity or discoloration.

Benefits vs Risks.

Aspect.	Benefits of Scaling.	Potential Risks.
Enamel.	Removes tartar, prevents decay.	Minimal microscopic roughness (rare).
Gums.	Reduces inflammation, reverses gingivitis.	Temporary soreness, bleeding.
Overall health.	Lowers risk of cardiovascular disease.	Rare infection if hygiene protocols fail.
Restorations.	Keeps margins clean.	Possible roughness on fillings/crowns.

Aftercare Tips.

Use desensitizing toothpaste if sensitivity persists.

Rinse with warm saltwater to soothe gums.

Avoid very hot, cold, or spicy foods for 24 48 hours.

Schedule regular cleanings every 6 months (or 3 4 months if prone to tartar buildup).

Q22 Can dental scaling cause gaps between my teeth?

Yes, dental scaling can sometimes make gaps between your teeth more noticeable, but it doesn't actually create new gaps. What happens is that scaling removes tartar and plaque that may have been filling spaces between teeth; once cleaned, the natural spacing or gum recession becomes visible. This is usually a sign of improved gum health, not damage.

Why Gaps May Appear After Scaling.

Tartar removal: Hardened deposits can act like “fillers” between teeth. Once removed, the underlying natural spacing is revealed.

Gum recession: If gums were swollen from infection, they shrink back after healing, exposing more of the tooth and root.

Loose teeth: In cases of advanced gum disease, teeth may feel temporarily wiggly after scaling, which can make gaps seem larger.

Improved gum attachment: Root planing helps gums reattach to teeth, which stabilizes them over time.

Benefits of Scaling.

Prevents tooth loss by removing bacteria that cause gum disease.

Reduces bad breath by eliminating odor-causing plaque.

Improves gum health by stopping bleeding and swelling.

Protects overall health, since oral bacteria can spread through the bloodstream.

Risks Side Effects.

Effect.	Why It Happens.	Duration.
Sensitivity.	Roots exposed after tartar removal.	Usually 1 2 weeks.
Tender gums.	Healing. process	Few days.
Bleeding.	Normal post-procedure response.	Short-term.
Visible gaps.	Tartar removal gum shrinkage.	Permanent, but natural.

Key Takeaway.

Scaling does not damage teeth or create gaps; it simply reveals the actual condition of your gums and teeth.

If gaps appear, it usually means tartar was masking them or gum disease had already caused recession.

Maintaining good oral hygiene (brushing, flossing, regular check-ups) helps prevent further gum loss and keeps gaps from worsening.

Q23 Do all gum diseases require the same treatment?

No, gum diseases do not require the same treatment mild cases like gingivitis can often be reversed with improved oral hygiene and professional cleaning, while advanced forms such as periodontitis may need deep cleaning, antibiotics, or even surgical procedures. The treatment depends on the stage and severity of the disease.

Types of Gum Disease Treatments.

Stage.	Gingivitis.	Periodontitis.
Severity.	Early, mild inflammation of gums.	Advanced infection affecting gums, ligaments, and bone.
Symptoms.	Red, swollen gums, bleeding when brushing.	Gum recession, deep pockets, loose teeth, bad breath.

Treatment   Professional cleaning.

Improved brushing flossing.

Quit smoking Scaling root planing.

Antibiotics (topical or oral).

Surgical options (flap surgery, bone grafts, gum grafts) Reversibility Fully reversible with proper care Not reversible, but manageable with treatment.

Key Treatment Approaches.

Early stage care.

Regular dental cleaning.

Daily brushing with fluoride toothpaste.

Flossing or interdental brushes.

Lifestyle changes (quit smoking, manage diabetes).

Moderate stage.

Scaling and root planing (deep cleaning under gums).

Antibiotic gels or rinses to control infection.

Advanced stage.

Flap surgery to clean deep pockets.

Bone grafts to restore lost bone.

Gum grafts to cover exposed roots.

Guided tissue regeneration to regrow bone.

Risks Considerations.

Untreated gum disease can lead to tooth loss and is linked to systemic conditions like heart disease, diabetes, and stroke.

Genetics, smoking, and hormonal changes (pregnancy, menopause) increase risk.

Gum disease is not curable once bone loss occurs, but it can be controlled with consistent treatment and oral hygiene.

Q24 Is gum treatment painful?

Most gum treatments are not painful because dentists use local anesthesia or sedation to keep you comfortable. Mild procedures like routine cleaning or scaling may cause only slight sensitivity, while advanced surgical treatments can involve temporary soreness during recovery.

Types of Gum Treatments and Pain Levels.

Treatment. Pain Level.   Notes.

Routine cleaning.   Minimal.   Feels like pressure or scraping; usually no anesthesia needed.

Scaling root planing.	Mild–moderate.	Local anesthesia is used; gums may feel sore for 1–2 days.
Antibiotic therapy.	None.	Medication placed in gum pockets; no pain.
Laser therapy.	Mild.	Less invasive than surgery; minimal bleeding and discomfort.
Flap surgery.	Moderate.	Performed under anesthesia; recovery soreness for 1–2 weeks.
Bone or gum grafting.	Moderate–high	Requires stitches; healing discomfort managed with painkillers.

Key Points About Pain Management.

Local anesthesia is almost always used for deep cleaning or surgery, so you won't feel pain during the procedure.

Sedation dentistry is available for anxious patients, making the experience more comfortable.

Post-treatment soreness is common, especially after surgical procedures, but usually controlled with prescribed painkillers.

Recovery time varies: simple cleanings need no downtime, while surgeries may require 1–4 weeks of healing.

Risks and Side Effects.

Temporary gum sensitivity to hot/cold foods.

Bleeding or swelling for a few days after surgery.

Rare complications: infection or gum recession.

Takeaway.

Gum treatment is generally not painful during the procedure thanks to anesthesia, though temporary soreness afterward is normal. If you're worried, ask your dentist about sedation options and pain management strategies.

Q25 Can gum surgery cause tooth loss?

No, gum surgery itself does not cause tooth loss in fact, it is usually performed to *prevent* teeth from becoming loose or falling out. However, if gum disease or bone loss is already severe before surgery, the affected teeth may still be at risk even after treatment.

Purpose of Gum Surgery.

Prevent tooth loss: Gum surgery removes infection, rebuilds bone, and restores gum tissue to stabilize teeth.

Treat gum recession: Procedures like gum grafting cover exposed roots, reducing sensitivity and protecting teeth.

Control advanced gum disease: Flap surgery cleans deep pockets of bacteria and repositions gums to fit snugly around teeth.

Rebuild bone support: Bone grafting strengthens the foundation of teeth when gum disease has damaged bone.

Risks vs. Benefits.

Procedure.	Goal.	Risk of Tooth Loss.
Gum grafting.	Covers exposed roots, prevents decay.	Very low — protects teeth.
Flap surgery.	Cleans deep pockets, reattaches gums.	Low stabilizes teeth.
Bone grafting.	Rebuilds bone support.	Low — strengthens tooth foundation.
Crown lengthening.	Exposes more tooth for restoration.	Minimal cosmetic/functional.
Laser surgery.	Removes diseased tissue with less trauma.	Very low promotes healing.

When Tooth Loss Can Still Happen.

Severe pre-existing damage: If gum disease has already destroyed too much bone, teeth may be unsalvageable.

Poor aftercare: Ignoring post-surgery instructions (e.g., smoking, poor hygiene) increases risk of complications.

Untreated gum disease: Surgery helps, but without ongoing care, infection can return and threaten teeth.

Key Takeaways.

Gum surgery is a protective treatment, not a cause of tooth loss.

The main goal is to restore gum and bone health so teeth remain stable.

Tooth loss risk depends more on the severity of gum disease before surgery than on the surgery itself.

Following dentist aftercare instructions (gentle brushing, antiseptic rinses, avoiding smoking) is critical for long-term success.

Q26 Can gum treatment make my teeth feel loose?

Yes gum treatments can sometimes make teeth feel loose temporarily, but this is usually part of the healing process. When inflamed gums shrink back after treatment, teeth may feel more mobile because the swollen tissue is no longer holding them tightly. However, the goal of gum therapy is to stop disease progression and stabilize teeth long-term, not make them fall out.

Why Teeth May Feel Loose After Gum Treatment.

Gum disease: Periodontitis damages the bone and ligaments supporting teeth. Treatments like scaling and root planing remove infected tissue, which can expose the true mobility of teeth.

Inflammation reduction: Swollen gums can “mask” looseness. Once swelling subsides, teeth may feel looser but are actually healthier.

Bone loss: If gum disease has already caused bone loss, treatment won’t immediately rebuild bone, so looseness may persist until healing or grafting occurs.

Healing phase: As gums reattach to teeth, stability often improves over weeks to months.

What Dentists Typically Do.

Scaling and root planing: Deep cleaning to remove plaque and tartar under the gums.

Splinting: Bonding loose teeth together for extra support.

Bone or gum grafts: Surgical options to rebuild lost support.

Night guards: Protect against grinding that worsens looseness.

When to Seek Help.

If looseness worsens after treatment instead of improving.

If you notice pain, pus, or swelling signs of infection.

If a tooth feels like it might fall out.

Key Takeaway.

Feeling loose teeth after gum treatment is common and often temporary.

The treatment is designed to stop gum disease and stabilize teeth.

Long-term stability depends on consistent oral hygiene and regular dental visits.

Q27 Does tartar removal cause more tartar buildup?

No, tartar removal does not cause more tartar buildup. In fact, professional removal of tartar (dental calculus) helps prevent gum disease and cavities. What sometimes happens is that people notice tartar forming again after a cleaning, but this is due to ongoing plaque accumulation from daily habitsnot because of the cleaning itself.

Why Tartar Returns After Removal.

Plaque formation: Plaque constantly develops on teeth from bacteria feeding on food particles. If not brushed away, it hardens into tartar.

Oral hygiene habits: Inconsistent brushing, flossing, or skipping mouthwash accelerates tartar buildup.

Dietary choices: Sugary and starchy foods promote plaque formation.

Lifestyle factors: Smoking, dry mouth, or braces increase tartar risk.

Benefits of Professional Tartar Removal.

Prevents gum disease: Scaling removes hardened deposits that irritate gums.

Protects enamel: Tartar can erode enamel if left untreated.

Improves breath aesthetics: Removal reduces odor and staining.

Allows better cleaning at home: Smooth tooth surfaces make brushing more effective.

Risks Misconceptions.

Myth: “Cleaning makes tartar grow faster.” Truth: Cleanings don’t accelerate tartar; they simply reset your teeth to a clean state. If plaque control is poor afterward, tartar reappears quickly.

Over-cleaning concern: Frequent professional cleanings are safe, but aggressive scraping at home with sharp tools can damage enamel and gums.

Natural break-off: Sometimes tartar chips off during eating or brushing, but this leaves rough surfaces that attract more plaque professional removal is safer.

How to Prevent Tartar After Removal.

Brush twice daily with fluoride toothpaste.

Floss once daily to remove plaque between teeth.

Use antibacterial mouthwash to reduce bacteria.

Limit sugary foods and drink water after meals.

Quit smoking to lower tartar risk.

Visit dentist regularly (every 6 months, or more often if prone to buildup).

Quick Comparison.

Factor.	With Tartar Removal.	Without Tartar Removal.
Gum health.	Healthier gums.	Risk of gum disease.
Enamel.	Protected.	Erosion cavities.
Breath.	Fresher	Bad. breath likely.
Appearance.	Whiter, smoother teeth.	Stains & rough surfaces.

Q28 Are antibiotics enough to treat gum disease?

Antibiotics alone are not enough to treat gum disease. They can help control infection, but the cornerstone of treatment is professional dental cleaning (scaling and root planing), improved oral hygiene, and sometimes surgical procedures depending on severity. Without mechanical removal of plaque and tartar, antibiotics cannot stop disease progression.

Why Antibiotics Are Not Sufficient.

Plaque removal: Gum disease is primarily caused by plaque buildup. Antibiotics cannot remove plaque or tartar; only scaling and root planing can.

Limited effect: Antibiotics reduce bacterial load but do not address the structural damage to gums and bone.

Resistance risk: Overuse of antibiotics can lead to resistant bacteria, making future infections harder to treat.

Standard Treatments for Gum Disease.

Treatment.	Purpose.	When Used.
Scaling root planing.	Removes plaque/tartar below gumline, smooths roots.	Mild to moderate gum disease.
Antibiotics.	Controls bacterial infection.	As adjunct to cleaning or surgery.
Flap surgery.	Opens gums for deep cleaning, reshapes bone.	Advanced periodontitis.
Bone grafting.	Restores lost bone support.	Severe bone loss.
Gum grafting.	Replaces lost gum tissue.	Gum recession cases.

Home Lifestyle Measures.

Daily brushing with fluoride toothpaste (2 3 times/day).

Flossing/interdental cleaning to remove plaque between teeth.

Quit smoking tobacco use worsens gum disease.

Regular dental check-ups every 6 12 months, or more often if at risk.

Risks of Relying Only on Antibiotics.

Gum disease may progress silently, leading to tooth loss.

Infection can spread deeper, requiring surgery.

Gum disease is linked to systemic health issues like diabetes, heart disease, and stroke.

Bottom Line.

Antibiotics can be part of gum disease treatment, but they are never a standalone cure. The most effective approach is professional cleaning plus consistent oral hygiene, with antibiotics used only as a supportive measure.

Q29 After gum disease treatment, will I ever need treatment again?

You may need further treatment after gum disease therapy, depending on the stage of disease, your oral hygiene, and risk factors. Gingivitis (early gum disease) can be fully reversed, but advanced periodontitis is usually managed long-term rather than “cured,” requiring ongoing maintenance and sometimes repeat procedures.

Key Points.

Early gum disease (gingivitis): Completely reversible with professional cleaning and good home care. If treated early, you may not need further treatment beyond regular check-ups.

Advanced gum disease (periodontitis): Damage to bone and tissue cannot be fully reversed. Treatment controls infection and stabilizes the condition, but ongoing maintenance is essential.

Maintenance therapy: Even after successful treatment, most patients need regular scaling, root planing, or specialist follow-ups every 3 6 months to prevent relapse.

Risk factors: Smoking, diabetes, stress, genetics, and poor oral hygiene increase the likelihood of recurrence.

Treatment Outlook.

Stage.	Treatment Effect.	Future Need.
Gingivitis.	Reversible with cleaning hygiene.	Usually no repeat treatment if hygiene is maintained.
Mild Periodontitis.	Scaling root planing.	Maintenance cleanings required.
Moderate Periodontitis.	Deep cleaning, possible antibiotics.	Ongoing monitoring possible repeat procedures.
Advanced Periodontitis.	Surgery, grafts, regeneration.	Lifelong management, risk of tooth loss.

Risks Considerations.

Relapse is common if plaque control is poor. Gum disease is driven by bacterial buildup, so daily brushing, flossing, and interdental cleaning are critical.

Systemic health links: Gum disease is associated with diabetes, heart disease, and pregnancy complications. Managing overall health reduces recurrence risk.

Lifestyle factors: Smoking and chewing tobacco significantly worsen prognosis. Quitting improves long-term outcomes.

Practical Guidance.

Regular dental check-ups: At least every 6 12 months, or more often if you've had periodontitis.

Professional cleanings: Every 3 6 months for those with a history of gum disease.

Home care: Brush twice daily with fluoride toothpaste, floss or use interdental brushes, and avoid smoking.

Specialist care: If you had advanced disease, ongoing follow-up with a periodontist may be necessary.

Q30 Is surgery always necessary for gum treatment?

No, surgery is not always necessary for gum treatment. Most cases of gum disease, especially in the early stages like gingivitis, can be managed with improved oral hygiene, professional cleanings, and nonsurgical procedures. Surgery is typically reserved for advanced periodontitis when deep pockets, bone loss, or severe tissue damage are present.

Gum Disease Treatment Options.

Early Stage (Gingivitis).

Professional dental cleaning and scaling.

Improved daily oral hygiene (brushing, flossing, interdental brushes).

Lifestyle changes such as quitting smoking.

Gingivitis is reversible with proper care.

Moderate Stage (Periodontitis).

Scaling and root planing: deep cleaning below the gumline.

Antibiotics: topical gels or oral medication to control infection.

Regular dental check-ups to monitor pocket depth and bone health.

Advanced Stage (Severe Periodontitis).

Flap surgery (pocket reduction): gums are lifted to clean roots and reshape bone.

Soft tissue grafts: restore receded gums and cover exposed roots.

Bone grafting: rebuild bone lost to infection.

Guided tissue regeneration or tissue-stimulating proteins: encourage regrowth of bone and gum tissue.

Comparison of Treatments.

Treatment.	Stage Used.	Invasiveness.	Goal.
Dental cleaning.	Gingivitis.	Non-invasive.	Remove plaque/tartar.
Scaling root planing.	Mild–Moderate.	Minimally invasive.	Clean deep pockets.
Antibiotics.	Mild–Moderate.	Non-invasive.	Control infection.

Flap surgery.	Advanced.	Surgical.	Reduce pocket depth.
Bone grafting.	Advanced.	Surgical.	Restore bone support.
Gum grafting.	Advanced.	Surgical.	Cover exposed roots.

Risks Considerations.

Delaying treatment can lead to tooth loss and systemic health issues (heart disease, diabetes complications).

Surgery carries risks like infection, pain, and longer recovery, but it may be the only option for severe cases.

Non-surgical treatments are effective if gum disease is caught early, making regular dental visits critical.

Key Takeaway.

If gum disease is detected early, surgery is not required. With proper oral hygiene and professional care, most cases can be reversed or controlled. Surgery becomes necessary only in advanced periodontitis when bone and tissue loss are significant.
