# Supplementary material for: Comparative Evaluation of Large Language Models in Responding to Common Misconceptions Regarding Periodontal Disease and Oral Hygiene
Source: Diagnostics (Basel). 2026 Sep 28;16(19):3158. doi: 10.3390/diagnostics16193158 (PMC13664004; doi:10.3390/diagnostics16193158)
Supplement: Supplementary file 1 [file diagnostics-16-03158-s001.zip › Supplementary File S5 LLMs response stylistic elements removed.zip!/2. LLMs response/DeepSeek V4.pdf]

Q1 Do gum diseases only affect elderly people?

No, gum diseases do not affect only elderly people. While it's true that the risk and severity of gum disease increase with age, it can occur at almost any stage of life starting in childhood and adolescence.

Gum disease is an inflammatory condition that generally falls into two categories. Understanding the difference between them is key.

Gum Disease Across Age Groups.

Condition.	Description.	Typical Age Group.
Gingivitis.	The mild, early stage, where gums become red, swollen, and may bleed easily. It is reversible with good oral hygiene.	This is common in children and teenagers. It is considered the most common form of periodontal disease in kids. Gingivitis typically begins in early childhood and becomes more prevalent and severe around puberty.
Periodontitis.	A more severe, chronic infection that damages the soft tissue and bone supporting the teeth, potentially leading to tooth loss.	It is uncommon in young children, but the prevalence increases during the teenage years and into adulthood. The disease often progresses slowly and becomes more clinically significant from the fourth decade of life onward.

Key Risk Factors Beyond Age.

Several factors can increase the risk of gum disease or make it worse, regardless of your age:

Hormonal Changes: Significant life stages like puberty, pregnancy, and menopause can make gums more sensitive and vulnerable to inflammation.

Systemic Health Conditions: Certain conditions, such as diabetes, can increase your risk.

Lifestyle Factors: Smoking or using tobacco products significantly lowers the resistance of gum tissue and increases susceptibility to disease .

Oral Hygiene: The primary cause of gum disease is the accumulation of dental plaque due to inadequate brushing and flossing .

In summary, age is a significant factor, but it's far from the only one. Poor oral hygiene can start the disease process in childhood, while other factors like hormonal changes and smoking can add to the risk throughout life.

Q2 Is it normal if my gums bleed when I brush my teeth?

This is one of the most common questions dentists get, and the short answer is: No, it is not normal. Healthy gums should *never* bleed from regular brushing.

However, it is very common and in most cases, it is reversible.

Here is exactly what is happening, why, and what to do about it.

The Cause: Gingivitis (Gum Inflammation).

Bleeding gums are the classic hallmark of gingivitis, which is the earliest stage of gum disease.

Your mouth naturally has bacteria that form a sticky film called plaque on your teeth.

If you don't remove plaque thoroughly, your immune system attacks the bacteria, causing inflammation in your gums.

Inflamed gums become swollen, red, and engorged with extra blood vessels. When your toothbrush bristles touch them, those fragile vessels burst, causing bleeding.

The big misconception: Many people think "My gums bleed, so I should brush softer or stop brushing that area." This is the worst thing you can do. You aren't hurting your gums; you are failing to remove the bacteria causing the inflammation.

Less Common Causes.

If you brush aggressively and your gums bleed, it could be due to:

Using a hard-bristled toothbrush: Switch to a soft or extra-soft brush.

New flossing routine: If you just started flossing after a long break, bleeding for 3 5 days is normal as your gums get used to it.

Medications: Blood thinners (like Warfarin, Eliquis) or certain blood pressure medications can make gums bleed more easily.

Hormonal changes: Pregnancy or puberty can make gums temporarily hypersensitive.

Vitamin deficiencies: Severe lack of Vitamin C or Vitamin K can contribute, though this is rare in developed countries.

The One Rule You MUST Follow.

Do not brush less; brush *more* (and better).

The bleeding will stop once the plaque is removed and the inflammation subsides. If you avoid the area, the plaque builds up, hardens into tartar (which you cannot remove with a brush), and the bleeding gets worse.

What to do right now.

Switch to an extra-soft toothbrush and use it at a 45-degree angle toward your gumline.

Use gentle, circular motions not a back-and-forth sawing motion.

Floss daily (even if it bleeds). The bleeding from flossing should stop entirely within a week of consistent flossing.

Rinse with warm salt water (1/2 teaspoon of salt in a glass of warm water) for a few days to soothe the inflammation.

When to see a dentist.

Make an appointment with your dentist for a professional cleaning if:

The bleeding doesn't stop after 7 to 10 days of stepping up your brushing and flossing routine.

You have persistent bad breath or a bad taste in your mouth.

Your gums are painful, very swollen, or pulling away (receding) from your teeth.

You have loose teeth or spaces opening up between your teeth.

If the bleeding stops with better hygiene, you likely just had mild gingivitis, and you have successfully reversed it. If it doesn't stop, you may have periodontitis (advanced gum disease) or an underlying medical issue that needs a dentist's or doctor's care.

Disclaimer: I am an AI, not a dentist. This information is for educational purposes and does not replace professional medical advice. If you are on blood thinners or have a compromised immune system, please call your dentist today rather than waiting a week.

Q3 Can I get gum disease even if I brush my teeth regularly?

Yes, you absolutely can.

Brushing regularly is the single most important thing you can do for your oral health, but it does not guarantee you will never get gum disease. Think of brushing as your first line of defense not your only one.

Here is why you can still develop gum disease even with a diligent brushing routine:

You are missing 40% of your tooth surfaces.
A standard toothbrush cannot reach the tight spaces *between* your teeth. If you don't floss or use a water flosser, plaque builds up in these gaps. Within 48 hours, that plaque hardens into tartar (calculus), which cannot be removed by a toothbrush and requires a dentist to scrape it off. Tartar is a safe haven for the bacteria that cause gum disease.

You are brushing incorrectly.
It is not just about frequency; it is about technique.

If you brush too hard, you can actually push your gums back (recession), exposing the softer root surface where bacteria easily attach.

If you don't brush along the gumline at a 45-degree angle, you are leaving plaque right at the margin where gum disease starts.

If you don't brush for a full two minutes, you likely aren't giving yourself enough time to clean every surface thoroughly.

Your genetics and biology are at play.
About 30% of the population is genetically predisposed to gum disease. Their immune system overreacts to the bacteria in plaque, causing excessive inflammation that destroys bone and tissue much faster than the average person. You can have perfect hygiene and still be susceptible.

Lifestyle factors override your brushing.
Several factors lower your mouth's natural defenses, making your brushing less effective:

Smoking or vaping: This restricts blood flow to your gums, starving them of oxygen and nutrients, and masks the early warning signs (bleeding).

Hormonal changes: Pregnancy, puberty, and menopause make gums more sensitive and reactive to even tiny amounts of plaque.

Medications: Antihistamines, blood pressure drugs, and antidepressants reduce saliva flow. Saliva is your mouth's natural mouthwash; without it, bacteria multiply faster.

Uncontrolled diabetes: High blood sugar makes it easier for gum infections to take hold and harder for them to heal.

You are confusing "bleeding" with "brushing too hard".
Many people see blood when they brush and think, *"I need to brush gentler."* Actually, healthy gums never bleed. Bleeding is a sign of inflammation caused by bacteria. If you brush regularly but your gums bleed, it means the bacteria are still there, and you need to brush *more* thoroughly along the gumline, not less (and start flossing).

The good news: If you brush regularly, you are far more likely to catch gum disease in its earliest, reversible stage gingivitis. This only affects the gums and can be cured with a professional cleaning and improved home care.

The more advanced stage periodontitis damages the bone holding your teeth and is permanent.

What to do next:

Add flossing or interdental brushes to your daily routine tonight.

Book a dental cleaning if you haven't had one in over 6 months. Only a dentist can remove the tartar your brush misses.

Check your technique: Hold your brush at a 45-degree angle toward your gums, use short back-and-forth strokes, and brush for a full 2 minutes.

Do you notice any bleeding when you brush, or are your gums looking redder or puffier than usual? That will tell you what stage you might be dealing with.

Q4 Can I have gum disease even if my gums don't hurt?

Yes, absolutely. In fact, pain is one of the last symptoms to appear with gum disease.

Many people are shocked to learn they have a serious dental problem because their gums feel completely fine. Here is why that happens and what to look for instead.

The Two Main Stages of Gum Disease:

Gingivitis (Early Stage): This is an infection of the gum tissue only. It is almost always painless. You might notice bleeding when you brush or floss, but it rarely hurts.

Periodontitis (Advanced Stage): This is when the infection destroys the bone and ligaments holding your teeth in place. Interestingly, this stage is often painless, too, because the deep pockets that form around the teeth allow bacteria to thrive without irritating the nerve endings on the surface. Pain usually only strikes very late, when an abscess forms or a tooth becomes so loose that it shifts and pinches nerves when you bite down.

Warning Signs to Watch For (That Aren't Pain):

Since pain is not a reliable indicator, check your mouth for these silent red flags:

Bleeding when you brush, floss, or eat crunchy food (this is the 1 earliest sign).

Pink-tinged saliva after spitting out your toothpaste.

Swollen, puffy, or shiny gums.

Bright red or dark purple gums (healthy gums are firm and pink).

Receding gums – teeth that look longer than they used to.

Persistent bad breath or a bad taste in your mouth that won't go away.

Loose teeth or changes in the way your bite fits together.

Spaces opening up between your teeth that weren't there before.

What you should do next:

Do not wait for pain to show up. By the time gum disease hurts, it is often very advanced and may require surgery or tooth loss.

Schedule a dental exam as soon as possible. Your dentist will use a small probe to measure the "pockets" around your teeth. If they are deeper than 3 millimeters, you have gum disease, even if you feel great.

In the meantime, ramp up your home care. Brush twice a day for two full minutes, and floss daily. Bleeding gums are a sign to floss *more*, not less; the bleeding will stop within 1 2 weeks of consistent flossing as the inflammation decreases.

If you haven't had a cleaning in over six months, call your dentist today. Catching it now could save you from painful, expensive treatments down the road.

Q5 Can gum disease get better without treatment?

The short answer is no gum disease generally will not get better without treatment. While there are rare exceptions, relying on your body to heal it on its own is a gamble that can lead to permanent damage.

Whether gum disease can improve without treatment depends entirely on its stage.

The Early Stage: Gingivitis

This is the earliest and mildest form of gum disease. The good news is that gingivitis is reversible, but it will not resolve on its own.

Why it doesn't go away on its own: Gingivitis is caused by plaque, a sticky film of bacteria that builds up on your teeth. If not removed, it hardens into tartar (calculus), which can only be removed by a dental professional. The inflammation will persist as long as the plaque and tartar remain.

What needs to happen: To reverse gingivitis, you need a professional dental cleaning to remove the tartar, combined with a diligent daily routine of brushing and flossing to prevent new plaque from forming. With this proper care, gums typically improve within a week or two.

The Advanced Stage: Periodontitis.

If gingivitis is left untreated, it can progress to periodontitis. This is a more serious stage where the infection spreads below the gum line and begins to destroy the bone and tissues that support your teeth.

This damage is permanent: Once periodontitis develops, the loss of supporting bone is irreversible. It will not get better on its own.

What can happen without treatment: Without professional treatment, the disease will typically continue to progress, leading to increased gum recession, loose teeth, and eventually, tooth loss. Treatment at this stage is focused on stopping the disease from getting worse and managing the damage already done.

A Rare Exception: "Self-Arresting" Disease.

The answer isn't completely black and white. There is evidence that in some cases, the progression of periodontal disease may slow down or stop on its own for a period of time. This is sometimes called "self-arresting" disease.

This means the active damage may pause, but the existing damage is not repaired.

Because the disease has a pattern of periods of activity and inactivity, it can be misleading. You might think the problem has resolved, only for it to start progressing again later.

The key takeaway: Even in these rare cases, you cannot rely on this happening. It's never a good idea to wait and see, as you risk allowing irreversible damage to occur to your teeth and gums.

The bottom line is that while early gum disease (gingivitis) is curable with professional help and improved hygiene, it will not fix itself. Advanced disease (periodontitis) can only be managed, not reversed, and ignoring it leads to tooth loss.

I hope this information is helpful. If you have any concerns about your gum health, a dentist can provide a proper diagnosis and the best course of action for your specific situation. Would you like to know more about the symptoms of gum disease or the specifics of professional cleaning procedures?

Q6 Are gum diseases rare?

No, gum diseases are not rare. They are one of the most common health conditions globally, affecting a significant portion of the adult population.

Here is a breakdown of the numbers:

In the United States: Nearly half (42-47%) of adults aged 30 and older have some form of periodontal disease. The prevalence increases with age, affecting about 60% of adults aged 65 and older.

Globally: It is estimated that gum disease affects up to 50% of the global population. In 2021 alone, there were nearly 1.1 billion prevalent cases of gum disease worldwide.

Understanding the Types and Severity.

Gum disease is a broad term that ranges from mild to severe.

Mild to Moderate (Gingivitis): This is the early, reversible stage of gum disease, characterized by inflammation of the gums. It is very common.

Severe (Periodontitis): This more advanced stage can destroy the bone and tissue supporting the teeth and is a leading cause of tooth loss. Severe periodontitis affects about 8-10% of the global population.

While it is common, gum disease is largely preventable and treatable with good oral hygiene and regular professional dental care.

Q7 Is it normal to lose teeth because of gum disease as you get older?

No, losing teeth from gum disease is not a normal or inevitable part of aging. While it is more common in older adults, it is a preventable disease, not a natural consequence of getting older. Periodontal (gum) disease is, in fact, the primary cause of tooth loss in adults.

Here’s a breakdown of why gum disease causes tooth loss and why age is a factor, but not the cause.

Why Gum Disease Causes Tooth Loss.

Periodontitis is a chronic inflammatory condition caused by a long-term buildup of bacterial plaque. This infection damages the gums and, more critically, destroys the periodontal ligament and the jawbone that anchor your teeth in place. Over time, this leads to receding gums, loose teeth, and eventually, tooth loss.

The Role of Age: Not the Cause, But a Risk Factor.

Tooth loss is more common with age because older adults have had a longer time for the effects of poor oral hygiene or untreated disease to accumulate. Other age-related changes can increase the risk, but they don't make tooth loss inevitable:

Receding Gums: Gums can naturally recede with age, exposing the softer, more vulnerable root surfaces to plaque and decay. This rate of recession is accelerated by gum disease.

Medications and Dry Mouth: Many common medications for older adults cause dry mouth (xerostomia), reducing saliva's protective effects and increasing the risk of cavities and gum disease.

Systemic Health Conditions: Conditions more prevalent in older age, like diabetes, are linked to a higher risk of periodontitis.

Physical Limitations: Conditions like arthritis can make it difficult to maintain a thorough oral hygiene routine.

Prevention and Management.

The good news is that periodontal disease and the resulting tooth loss are highly preventable and treatable. The key is to break the cycle of infection before it causes irreversible damage:

Excellent Daily Hygiene: Brushing twice a day and flossing daily are the foundation of prevention.

Regular Professional Care: See your dentist regularly for check-ups and professional cleanings. For those with a history of gum disease, more frequent maintenance visits may be necessary.

Manage Risk Factors: If you smoke, quitting is one of the most impactful things you can do for your gum health. Managing conditions like diabetes is also crucial.

I hope this clarifies the relationship between age, gum disease, and tooth loss. It's encouraging to know that with proper care, you can work to keep your natural teeth for a lifetime.

Q8 If gum disease runs in my family, will I definitely get it too?

That is one of the most common and understandable fears people have. Here is the straightforward answer: No, you will not *definitely* get it.

Having a family history of gum disease (periodontitis) does not mean it is your destiny. It means you have inherited a "loaded gun," but you are in complete control of whether the trigger gets pulled.

Here is why, and exactly what you can do about it.

What you actually inherit.
You don't inherit the *disease* itself. You inherit physical traits that make you *more susceptible* to it. These include:

The shape and depth of your gum pockets (deeper pockets trap more bacteria).

The specific makeup of your oral microbiome (the types of bacteria that naturally live in your mouth).

How aggressively your immune system responds to plaque (an overreaction causes the inflammation that destroys bone).

The 1 game-changer: Plaque.
Gum disease is 100% bacteria-driven. If you have a genetic susceptibility, your gums will react to a tiny amount of plaque the way a non-susceptible person reacts to a large amount of plaque.

But here is the crucial truth: If you remove that plaque every single day, your gums have nothing to react to. The genetic switch never gets flipped.

Your "Anti-Genetics" Action Plan.

If gum disease runs in your family, standard dental advice is not enough for you. You need to be proactive:

Upgrade your home care: Regular brushing isn't enough. You must floss daily, and more importantly, add a water flosser (Waterpik) or interdental brushes to flush out the plaque below the gumline where your brush can't reach.

Use an electric toothbrush: A rotating-oscillating electric brush (like Oral-B) or a sonic brush (like Sonicare) removes significantly more plaque than a manual brush.

Don't skip the dentist: For someone with average risk, every 6 months is fine. For you, every 3 to 4 months for professional cleanings (prophylaxis) is often recommended. This disrupts the bacteria before your genetics can react to it.

Know your medications: Certain meds (like blood pressure drugs or antidepressants) cause dry mouth, which reduces your saliva's natural ability to wash away bacteria. If you take these, tell your dentist.

The one thing that will override your genetics.
Smoking. If you have a family history of gum disease and you smoke, your risk goes through the roof. Tobacco use is the single biggest environmental factor that makes gum disease progress rapidly. Quitting is the most powerful genetic "off switch" you have.

The good news.
Unlike genes for eye color, the genes for gum disease are not hardwired. They are "expression genes" they only turn on in a bad environment. By keeping your mouth exceptionally clean, you can have perfectly healthy gums for your entire life, regardless of what your parents or grandparents experienced.

If you are currently in your 20s or 30s with no bone loss, you have caught this early enough to completely outrun your genetics.

Would you like me to break down the exact daily routine (step-by-step) that dentists recommend for people with high genetic risk?

Q9 Can stress make me more likely to get gum disease?

Yes, stress can increase your risk of developing gum disease. It does this through a combination of biological changes and the adoption of habits that are harmful to your oral health.

The Biological Connection.

Stress triggers the release of hormones that directly impact your body's ability to fight off infection.

Hormonal Cascade: When you're stressed, your body releases hormones like cortisol and DHEA. Studies consistently show that people with periodontitis have higher levels of these stress hormones compared to those with healthy gums .

Weakened Immune Defenses: High cortisol levels can suppress the immune system, reducing the activity of key immune cells . This makes it harder for your body to fight the bacteria that cause gum disease, allowing the infection to take hold and progress.

Changes in Oral Bacteria: Stress can also alter the environment in your mouth. The changes in immune function can make it easier for harmful bacteria, like *P. gingivalis* (a major pathogen in gum disease), to grow and become more aggressive .

The Behavioral Connection.

Stress doesn't just affect your biology; it often leads to behaviors that are bad for your gums .

Poor Oral Hygiene: When stressed, you might be more likely to neglect your regular brushing and flossing routine, allowing plaque to build up .

Unhealthy Coping Habits: Stress can also lead to habits like smoking, drinking more alcohol, or eating an unhealthy diet, which are all established risk factors for gum disease .

It's also worth noting that the relationship might go both ways. Some research suggests that chronic stress, depression, and the inflammation from gum disease can create a cycle where they worsen each other, but this is still an area of active research .

While the evidence linking stress and gum disease is compelling, it's important to highlight that some studies are still exploring the nuances. For instance, one study found that anxiety, rather than general stress, was the more significant factor after other variables were accounted for, showing a dramatically increased risk of periodontitis . This indicates that not all psychological stressors affect everyone in the same way.

What You Can Do.

Since stress is a part of life, managing its impact on your oral health is key.

Maintain Excellent Oral Hygiene: This is your first and best line of defense. Brushing twice a day and flossing daily is crucial, especially when you're feeling overwhelmed .

Find Healthy Ways to Manage Stress: Incorporate stress-reducing activities into your routine, such as exercise, meditation, or talking to a friend or therapist. Taking care of your mental health is an important part of protecting your gums.

See Your Dentist Regularly: Don't skip your dental checkups, especially during stressful periods. Your dentist can spot early signs of gum disease (gingivitis) and help you manage it before it becomes a more serious problem.

I hope this information is helpful for you. If you're feeling particularly stressed and are concerned about your gum health, it might be a good idea to schedule a checkup with your dentist for a personalized assessment.

Q10 Do gum diseases only affect the mouth and teeth?

No, gum diseases like periodontitis are not confined to the mouth. They are a significant risk factor for several serious systemic health conditions . The primary link is chronic inflammation the infection in

your gums can spread inflammatory mediators and bacteria throughout your body, affecting other organs .

Here is how gum disease is connected to different parts of the body:

Systemic Condition.	How Periodontitis Plays a Role.
Cardiovascular Disease.	Oral bacteria can enter the bloodstream and contribute to the formation of atherosclerotic plaques in arteries, increasing the risk of heart attack and stroke .
Diabetes.	This is a bidirectional relationship . Gum disease makes it harder to control blood sugar, while high blood sugar from diabetes worsens gum infections . Treating periodontitis can significantly improve blood glucose control, reducing HbA1c levels by about 0.4% .
Respiratory Infections.	Bacteria from the mouth can be aspirated into the lungs, leading to infections like pneumonia or aggravating conditions like COPD .
Rheumatoid Arthritis (RA).	Both conditions share similar inflammatory pathways and immune responses. The specific oral bacteria *Porphyromonas gingivalis* has been linked to producing proteins that trigger the autoimmune response in RA .
Alzheimer's Disease.	Research has found oral bacteria and their toxic byproducts in the brains of Alzheimer's patients, suggesting a potential link through systemic inflammation and neuroinflammation .

The connection is also influenced by the "oral-gut axis," where swallowed oral bacteria can alter the gut microbiome, further contributing to systemic inflammation and metabolic issues .

It is important to note that while these strong associations exist, establishing a direct cause-and-effect relationship is still an area of ongoing research. However, current evidence clearly supports gum health as an essential part of overall health .

Q11 Will a hard-bristled toothbrush clean my teeth better?

No, a hard-bristled toothbrush will not clean your teeth better in the long run. While they can be more efficient at removing plaque in a single use, this small benefit comes with a major risk of permanently damaging your teeth and gums. Dental professionals universally recommend soft-bristled brushes for safe and effective daily cleaning .

Here is a breakdown of why hard bristles are not recommended, and how soft bristles are a better choice for your oral health.

The Short-Term Benefit vs. Long-Term Harm.

Hard bristles are more abrasive. One study found that cleaning efficiency does increase with bristle hardness, meaning a hard brush can remove more plaque with fewer strokes . However, this does not make it a better choice for daily use.

Risks of Hard Bristles:

Gum Recession: The abrasive action of hard bristles, especially when combined with scrubbing pressure, can cause your gums to pull away from your teeth .

Enamel Erosion: Hard bristles can wear down the protective enamel on your teeth. This not only increases the risk of cavities and sensitivity but can also make your teeth appear more yellow as the darker dentin underneath becomes exposed .

Dentist Recommendations: Because of these risks, major dental organizations like the American Dental Association recommend soft-bristled toothbrushes .

Why Soft Bristles Are the Preferred Choice.

Dentists recommend soft-bristled toothbrushes for most people because they effectively remove plaque without damaging oral tissues . A soft brush's flexible bristles can bend and reach into the grooves and crevices of your teeth for a thorough clean .

Key Points for an Effective Clean:

Technique Matters: The effectiveness of brushing depends more on your technique and duration than on the hardness of the bristles. Using proper method is crucial for plaque removal .

Evidence for Medium Brushes: It's worth noting that one study found medium brushes were more effective at removing plaque than soft ones, but neither caused gum damage . However, the consensus among dental experts remains that soft bristles are the safest and most recommended choice for daily use to prevent accidental damage from overzealous brushing.

Ultimately, your goal is to protect your gums and enamel for a lifetime. For daily cleaning, a soft-bristled brush, used with the correct technique, is the most effective and safest option.

Q12 Should I stop brushing my teeth for a few days if my gums are bleeding?

No, you should not stop brushing your teeth. In fact, doing the opposite brushing *more* gently but more thoroughly is exactly what you need to do.

Here is why, and exactly how to handle it:

Why you shouldn't stop:
Bleeding gums are usually a sign of gingivitis inflammation caused by plaque buildup along your gumline. Plaque is a sticky film of bacteria. If you stop brushing, that plaque hardens into tartar within 48 hours, which makes the inflammation and bleeding worse, not better. Think of the bleeding like a tiny infection; stopping brushing is like ignoring a splinter instead of removing it.

The "Rules of Engagement" for bleeding gums:

Switch to an extra-soft toothbrush (labeled "soft" or "ultra-soft"). Hard bristles irritate inflamed gums.

Use a "massage" technique, not a "scrub." Hold the brush at a 45-degree angle toward your gumline and use tiny, circular, vibrating motions. Do not saw back-and-forth.

Floss Daily even if it bleeds. Flossing removes the plaque between teeth that your brush can't reach. The bleeding from flossing will stop within 3 to 5 days of consistent, gentle flossing as the inflammation subsides.

Rinse with warm salt water (1/2 teaspoon of salt in a cup of warm water) twice a day. This soothes the gums and helps reduce bacteria without harsh chemicals.

When to see a dentist (within the next week):

If the bleeding does not noticeably improve after 5 to 7 days of gentle, consistent brushing and flossing.

If you have a *persistent* metallic taste in your mouth, or if a single specific area bleeds heavily when you press on it (this could indicate an abscess).

If you take blood thinners (like Warfarin or Eliquis) or have a bleeding disorder in which case, call your dentist's office for specific advice today.

The one exception: If your gums are bleeding because you *just* had a dental extraction or oral surgery, follow your dentist's post-op instructions they will usually tell you to avoid brushing the surgical site for 24 hours. But for everyday bleeding gums, keep brushing!

Q13 Do I need to brush my teeth after every meal to keep my gums healthy?

The short answer is no, you do not need to brush after *every* single meal to keep your gums healthy. In fact, brushing too often or at the wrong time can actually harm your gums.

Here is what you actually need to know for healthy gums:

The "Gold Standard" Rule.
For optimal gum health, dentists recommend brushing twice a day (morning and night) and flossing once a day. The most critical brushing session is at night before bed, as saliva production drops while you sleep, allowing plaque to attack your gums and teeth more aggressively.

When brushing after a meal IS a good idea:

If you ate something highly sugary or sticky (like caramel or dried fruit).

If you have braces, retainers, or dentures, where food gets trapped easily.

If you are prone to rapid plaque buildup or have a history of gum disease.

The Big "Wait" Rule (Crucial for Gums).
If you just ate or drank anything acidic (citrus fruits, tomatoes, soda, wine, coffee, or sports drinks), do NOT brush for at least 30 to 60 minutes. Acid temporarily softens your tooth enamel and irritates your gum tissue. If you brush during this window, you are literally scrubbing away softened enamel and abrading your gums, which can cause them to recede over time. Instead, rinse your mouth with water or chew sugar-free gum to neutralize the acid.

What to do instead of brushing after every meal:

Rinse with water: Swish vigorously to dislodge food particles.

Chew sugar-free gum: Look for one with Xylitol, which stimulates saliva to wash away food and bacteria.

Use a water flosser: Gently spray between teeth to remove debris without abrading gums.

The mistake that hurts gums.
Brushing *too hard* (regardless of frequency) is worse for your gums than skipping an afternoon brush. Always use a soft-bristled toothbrush and hold it at a 45-degree angle toward your gumline, using gentle, circular motions not a back-and-forth sawing motion.

The Bottom Line:
If you eat a meal and feel the need to clean your mouth, rinse with water and wait 30 minutes. If you

still want to brush after that, go ahead but doing it after every single meal is unnecessary. Twice a day with proper technique, plus daily flossing, is the proven recipe for healthy gums.

Q14 Even if I brush my teeth regularly, do I still need to get my gums checked?

Yes, absolutely. Brushing regularly is fantastic (and non-negotiable), but it is not a substitute for professional gum check-ups.

Here is why:

Brushing misses the "danger zone".

Your toothbrush can only clean the visible surfaces of your teeth and about 1–2 millimeters below the gumline. But the gum pockets around your teeth can be 3–6 millimeters deep (or more). Plaque and tartar that accumulate in these deep pockets cause gum disease, and your brush simply cannot reach them.

You can't feel the early stages.

The early stage of gum disease (gingivitis) is usually painless. You might have bleeding gums when you brush, but many people dismiss this as "brushing too hard." By the time gum disease becomes painful, it has often progressed to periodontitis, which can cause permanent bone loss and tooth mobility.

Tartar is rock-hard.

Even if you brush perfectly, minerals in your saliva harden plaque into calculus (tartar) within 24–48 hours. Once it forms, tartar is bonded to your teeth like concrete. No amount of brushing or flossing can remove it only a dentist's specialized scraping tools (scalers and curettes) can.

Your dentist checks for more than cavities.

At a gum check, your dentist or hygienist does a "periodontal probing" they use a tiny ruler to measure the depth of the pockets around each tooth. They also check for gum recession, bleeding points, and bone loss (often via X-rays). These are things you cannot check for yourself in the mirror.

Your overall health is connected.

Research links untreated gum disease to systemic issues like heart disease, diabetes complications, and stroke. Your gums are a window to your systemic inflammation levels.

The Bottom Line:

If you brush perfectly (2 minutes, twice a day, with proper technique) + floss daily, you should still get a professional gum check at least once every 6 months.

If you smoke, have diabetes, or have a family history of gum disease, your dentist may recommend checks every 3–4 months, regardless of how well you brush.

Think of brushing as your daily shower, and the gum check as your annual physical. One keeps you clean day-to-day; the other catches hidden problems before they become emergencies.

Q15 Is brushing my teeth once a day enough to keep my mouth healthy?

Based on the consensus from dental professionals, brushing your teeth once a day is generally not enough to keep your mouth optimally healthy.

The official recommendation from organizations like the American Dental Association (ADA) is to brush your teeth twice a day for two minutes with a fluoride toothpaste. This advice is based on substantial scientific evidence linking twice-daily brushing to a lower risk of tooth decay and gum disease.

Why Twice a Day is the Standard.

The difference comes down to effectively fighting plaque, a sticky film of bacteria that constantly forms on your teeth. When you eat or drink, these bacteria produce acids that can erode tooth enamel, leading to cavities, and the plaque itself can harden into tartar, which causes gum inflammation. Here is what the evidence shows:

More Effective Plaque Removal: Studies have consistently found that brushing twice daily is significantly more effective at removing plaque than brushing just once a day. A study on children showed that those who brushed twice a day had notably better dental health than those who brushed only once. Furthermore, research on young adults demonstrated that those who brushed three times a day had the least plaque formation and the healthiest gums, strongly supporting the superiority of more frequent brushing.

The Role of Fluoride: The benefits of brushing twice a day are not just about physically removing plaque. It also ensures your teeth are exposed to the protective effects of fluoride toothpaste at least twice daily. Fluoride helps strengthen enamel and can even reverse early stages of decay.

Some might argue that, in theory, one perfect brushing session could be enough. However, research indicates that in practice, very few people are capable of removing all plaque thoroughly in a single brush, making twice a day a more reliable standard for maintaining healthy gums.

A quick summary of what you can do for optimal oral health:

Brush twice daily for two minutes each time using fluoride toothpaste.

Brush at night, as this is critical for removing the day's buildup of plaque and food.

Don't brush immediately after eating acidic foods or drinks. Wait about an hour to protect your softened enamel.

Visit your dentist regularly for checkups and professional cleanings.

I hope this information helps you make the best choice for your oral health! If you're interested, I can share some tips on proper brushing technique.

Q16 Can flossing cause gum recession?

Yes, but only if done incorrectly. Proper flossing is essential for preventing gum recession, while aggressive or improper technique can cause it.

Here is how flossing and gum recession are connected:

Incorrect Flossing Causes Recession: Using a sawing motion or forcing the floss too deeply can traumatize and cut the gum tissue, which can lead to recession over time. Aggressive flossing can cut into the gums and cause them to pull away from the teeth.

Not Flossing Also Causes Recession: Skipping flossing allows plaque to build up between teeth and along the gumline. This plaque can harden into tartar, leading to gum disease (periodontitis), which is the most common cause of gum recession.

The key is to use the correct technique. To floss safely, gently slide the floss between teeth, curve it into a "C-shape" against the side of each tooth, and carefully guide it beneath the gumline without forcing it. If you are unsure about your technique, your dentist or hygienist can demonstrate the proper method for you.

Q17 Do electric toothbrushes damage the gums more than regular toothbrushes?

Based on the evidence, no, electric toothbrushes do not inherently damage gums more than manual ones. In fact, for most people, they are likely safer and less harmful. The key factor is not the type of brush, but the technique used, especially the amount of pressure applied .

What the Evidence Shows.

The relationship between toothbrush type and gum damage has been studied extensively. Here's a breakdown of the findings:

Brushing Force: The primary cause of gum damage is brushing too hard. Studies consistently show that people tend to use significantly less force with an electric toothbrush compared to a manual one . This is a major advantage, as lower force reduces the risk of gum recession and abrasion .

Built-in Safety Features: Many modern electric toothbrushes come with pressure sensors that alert you when you are brushing too hard, helping to prevent damage . This feature is not available on manual brushes.

Clinical Study Results: A 1988 clinical study found that manual toothbrushes abraded the gums more than electric toothbrushes . A 2025 review of research concluded that electric toothbrushes are safer and induce less tooth surface loss . Other studies have found no significant difference in gum abrasion between the two when used correctly , or concluded that concerns about electric brushes causing recession are "not warranted" .

One Important Caveat.

While the overall evidence points to electric brushes being safer, one study simulating 8.5 years of brushing with the same force found that electric brushes caused more tooth surface loss . This highlights the most important rule: if you apply the same heavy force to an electric toothbrush as you do to a manual one, you could still cause damage .

How to Protect Your Gums.

Regardless of which brush you use, the following practices are key to protecting your gums:

Use soft bristles: Always choose a brush with soft bristles. Hard bristles are a proven cause of soft tissue trauma and gum lesions .

Don't press hard: Let the brush do the work. Hold the brush lightly (like a pencil) and use gentle, circular motions . If you use a manual brush, aim for a brushing force of around 2-3N .

Angle your brush: Hold your brush at a 45-degree angle towards your gumline to effectively clean without scrubbing .

Replace the head regularly: Change your brush head (or manual brush) every three months, or sooner if the bristles become frayed .

The Bottom Line.

If you are prone to brushing too hard, an electric toothbrush with a pressure sensor is an excellent investment to protect your gums . If you prefer a manual brush, focus intensely on using a gentle technique and soft bristles.

I hope this detailed breakdown helps you make the best choice for your dental health. Since brushing technique is so important, would you like me to explain the proper brushing technique in more detail?

Q18 Would using mouthwash alone be enough without brushing my teeth?

The short answer is no. Using mouthwash alone is *not* enough to maintain good oral health.

Here is why mouthwash cannot replace brushing, broken down by what each one actually does:

Plaque is a physical film.
Plaque is a sticky, glue-like biofilm of bacteria that adheres firmly to your teeth. The only way to physically break up and remove this film is through the mechanical scrubbing action of a toothbrush bristle. Mouthwash is a liquid; it rinses over the plaque but does not have the friction needed to scrape it off.

Mouthwash misses the "sweet spot".
The area where your tooth meets your gum line (the gingival sulcus) is where plaque builds up fastest and causes the most damage (gingivitis). Toothbrush bristles are designed to sweep into this crevice. Mouthwash cannot penetrate this tight space effectively.

Fluoride needs contact time.
Both toothpaste and many mouthwashes contain fluoride to strengthen enamel. However, toothpaste contains a much higher concentration of fluoride, and it stays on your teeth longer because the abrasives in the paste cling to the surface. Mouthwash is rinsed away in seconds, giving the fluoride very little time to absorb.

The "dead" bacteria problem.
While antibacterial mouthwashes do kill bacteria, they only kill the ones on the surface. The bacteria hiding deep in the plaque biofilm are protected by a slimy shield. If you don't brush away that shield first, the mouthwash can't reach the bacteria underneath.

What happens if you only use mouthwash?

Within 24 hours: Your teeth will feel fuzzy (plaque build-up).

Within a few days: Your gums will likely become inflamed, red, and prone to bleeding (gingivitis).

Long-term: The plaque will harden into tartar (calculus), which cannot be removed by brushing or mouthwash it requires a dentist's scraping tools. Over time, this leads to cavities and gum disease.

The proper hierarchy of oral care:

Brush (twice daily for 2 minutes) – This does 90% of the work.

Floss (once daily) – This cleans the 40% of tooth surfaces that your brush can't reach.

Mouthwash (optional, 1–2 times daily) – This is a *supplement*. Its best uses are to reach areas you might have missed, freshen breath, or deliver extra fluoride *if* your dentist recommends it.

Crucial tip: If you do use mouthwash, do not use it immediately after brushing. Rinsing with mouthwash right after brushing washes away the concentrated fluoride from your toothpaste. For the

best results, brush, spit out the excess toothpaste, and wait 30 minutes before using mouthwash. Or, use mouthwash at a different time of day (like after lunch).

If you are in a situation where you forget your toothbrush, using mouthwash is *much better than doing nothing*. But as a daily routine, it is never a substitute for brushing.

Q19 After I quit smoking, my gums started bleeding. Is that normal?

Yes, it is very normal for your gums to bleed after you quit smoking. In fact, this increase in bleeding is a well-documented sign that your body is healing and your circulation is returning to normal.

Here is why it happens and what it means:

The "Mask" of Smoking is Lifted.

The primary reason is that smoking acts as a powerful vasoconstrictor, meaning it narrows your blood vessels and reduces blood flow to your gums.

While smoking: This reduced blood flow suppresses the normal signs of gum inflammation, like redness and bleeding. It essentially "masks" the underlying gum disease caused by plaque buildup.

After quitting: Your circulation improves as your blood vessels return to normal function. The gums can now show the true inflammatory response to plaque, which includes bleeding when brushed or probed.

The Science Behind the Bleeding.

This isn't a sign that your oral health is getting worse. Instead, it's a sign of a positive change in your body's inflammatory and immune response. One study showed that gingival bleeding on probing can double from 16% of sites to 32% within 4 to 6 weeks of quitting, even when participants maintained good oral hygiene. Another study confirmed a significant increase in gingival bleeding three months after successfully quitting.

Other factors that may contribute to this bleeding include:

Nicotine Replacement Therapy (NRT): If you are using nicotine gum or lozenges, bleeding gums can be a side effect of the product itself.

Changes in Oral Bacteria: Quitting smoking changes the ecosystem of bacteria in your mouth, which can also play a role in the inflammation response.

Important Considerations.

While this is a normal part of the healing process, it does not mean you should ignore it. The bleeding is a sign that you have gum inflammation that needs to be managed.

This is temporary. The bleeding is a short-term effect. As your gums continue to heal and your circulation improves, this symptom will subside, especially if you follow a good oral hygiene routine.

Continue thorough cleaning: Continue to brush gently but thoroughly and floss. The bleeding will reduce as plaque is removed and your gums become healthier.

See a dentist: This is an excellent time to see a dentist for a professional cleaning and check-up. They can assess the health of your gums, remove any built-up calculus (tartar), and provide personalized advice to help you through this healing phase. This will prevent any apprehension about the bleeding from causing you to restart smoking.

Quitting is the single most important thing you can do for your long-term oral and overall health, and this temporary bleeding is a positive sign that the healing has begun.

Q20 Can natural products effectively keep my gums healthy?

Yes, natural products can be an effective part of a routine to keep your gums healthy, particularly when used alongside standard professional care and daily brushing and flossing. Recent research shows that several natural compounds and herbal formulations have measurable benefits for gum health.

Here's a look at some of the natural products with promising scientific support:

Natural Product / Compound.	How It May Help Your Gums.	Key Research Finding.
Herbal Mouthwashes (e.g., with *Achyranthes aspera*, *Trachyspermum ammi*).	Reduce plaque and gingivitis.	Found to be non-inferior to the "gold standard" chlorhexidine mouthwash, but without common side effects like taste alteration or tooth staining.
Complex Herbal Tincture (e.g., with marigold, yarrow, sage).	Anti-inflammatory, reduces plaque and bleeding.	As an adjunct to non-surgical therapy, it significantly reduced bleeding on probing and levels of key periodontitis-causing bacteria (like *Tannerella forsythensis*).
Gum Arabic (from Acacia trees).	Disrupts bacterial communication ("quorum sensing") to prevent biofilm formation and reduce inflammation.	Clinical studies show it significantly reduces plaque accumulation, gingival inflammation, and bleeding.
Morin (found in guava leaves, apple peels, certain teas).	Antimicrobial, anti-inflammatory, and antioxidant properties.	Laboratory tests show it effectively acts against the bacteria responsible for periodontal disease. (Currently, it is a promising compound being researched for use in oral hygiene products).
Herbal Gel (e.g., with *Picrorhiza kurroa*, *Ficus bengalensis*).	Promotes antimicrobial and anti-inflammatory effects in deeper pockets.	As an adjunct to non-surgical therapy, it significantly improved bone fill in severe periodontitis defects after 6 months.

How to use natural products effectively.

It's important to keep a few key points in mind:

They are an "Adjunct," Not a Replacement: Think of natural products as powerful teammates for your toothbrush and dental floss, not replacements for them. The clinical studies that show positive results used these natural products in addition to good oral hygiene and professional treatments like scaling and root planing. They help reduce plaque, bacteria, and inflammation, but they cannot remove hardened tartar on their own.

"Natural" Doesn't Always Mean "Safe" or "Effective": The beneficial effects of a natural compound often depend on how it is processed and formulated. For example, simply eating guava leaves won't deliver the same concentrated, controlled-release effect as a scientifically formulated product containing morin. Also, while many herbal mouthwashes may have fewer side effects than chlorhexidine, some, like the tincture mentioned in the studies, can still cause minor issues like taste changes in some people.

The Future is Promising: Research on natural products for oral health is an active area of study. The potential of compounds like morin and gum arabic is exciting, and ongoing research is likely to lead to more refined, effective, and accessible natural dental care products in the future.

I hope this gives you a clearer picture of the potential benefits and limitations of natural products for gum health. If you're interested in a specific type of product, like mouthwashes or gels, I can provide more detailed information.

Q21 Can getting a dental scaling damage my teeth?

This is one of the most common questions dentists hear, and the short answer is no when performed by a qualified professional, a dental scaling (cleaning) will not damage your teeth.

However, I completely understand why people think it does. Here is exactly why your teeth might *feel* damaged afterward, what is actually happening, and when scaling *could* cause problems.

Why your teeth feel "damaged" (but aren't):

Rough surfaces: After scraping off hard tartar (calculus), the root surfaces that were covered for years may feel rough or jagged. This isn't damage; it is the natural texture of your root. Your dentist polishes these surfaces to smooth them out.

Sensitivity: Scaling removes the "blanket" of tartar that was insulating your teeth from hot and cold. Without that blanket, your exposed enamel and roots will temporarily feel temperature changes more intensely. This fades within a few days to weeks.

Gaps between teeth: Tartar buildup often wedges between teeth, pushing your gums down and creating pseudo-"fillers." When that tartar is removed, you feel the natural gap with your tongue and think the hygienist chipped your tooth. They didn't; the gap was always there, just packed with bacteria.

The one exception: Over-aggressive scraping.
In very rare cases, a hygienist who uses excessive pressure or incorrectly angles their metal scaler *can* scratch the enamel or cementum (root surface). However, modern hygienists are trained to use ultrasonic scalers and light strokes to prevent this. Even if a tiny scratch occurs, it usually remineralizes with your saliva's minerals within a few weeks.

The real damage comes from NOT scaling.
This is the most important point: The true danger to your teeth is avoiding scaling. When you skip cleanings:

Tartar builds up below the gumline, causing periodontitis.

This destroys the bone that holds your teeth in place.

Bone loss is permanent and irreversible unlike sensitivity, which is temporary.

Eventually, the teeth loosen and fall out.

The only legitimate risk to be aware of:
If you have severe gum disease, scaling can temporarily cause your gums to bleed and swell more, and in extremely rare cases, bacteria can enter your bloodstream (bacteremia). If you have a heart condition or artificial joint, your dentist will give you antibiotics beforehand to prevent this. But this is a risk to your *body*, not damage to your *teeth*.

What to do if you are worried:
Request a "fine" or "ultrasonic" scaler rather than heavy hand-scaling. Ask your hygienist to use a *light touch*. And if you have sensitivity afterward, use a desensitizing toothpaste (like Sensodyne) for two weeks and avoid ice-cold water.

In summary: Do not skip your cleanings. The temporary discomfort of a cleaning is protection; skipping it is the real cause of tooth loss. If your teeth feel sensitive or rough afterward, give it 10 days if it doesn't resolve, call your dentist for a polish, but know that your teeth are structurally sound.

Q22 Can dental scaling cause gaps between my teeth?

No, dental scaling does not actually create new gaps between your teeth. What you're noticing after the procedure is most likely a positive change the removal of tartar that was hiding existing spaces and the reduction of swollen, inflamed gum tissue.

Here’s a breakdown of what's really happening:

Tartar Was Filling the Gaps: Before scaling, hard calculus (tartar) may have built up between your teeth. This deposit acted as a "filler," creating the illusion that your teeth were close together. Once the dentist removes it, the natural, original spaces between your teeth become visible .

Gum Inflammation Was Masking the Issue: Gum disease causes gums to become swollen, puffy, and inflamed, which can also hide spaces created by underlying bone loss . Scaling removes the cause of this inflammation.

Healing Gums Shrink to a Healthier Size: After the deep cleaning, the gum tissue heals and reduces to its healthy, firm state. Scientific studies have confirmed this tissue shrinkage. One study found an average reduction in gum width of about 0.34 mm and height of about 0.2 mm after treatment . This reduction in swollen tissue makes pre-existing gaps more noticeable .

These changes are signs that your gums are healing from gum disease, not that the treatment has harmed your teeth . The gaps were likely already there, caused by the gum disease process itself, and were simply hidden until the cleaning .

Q23 Do all gum diseases require the same treatment?

No, not all gum diseases require the same treatment. The approach depends entirely on the severity of the condition, which falls into two main stages: gingivitis (reversible gum inflammation) and periodontitis (advanced, irreversible disease that damages supporting bone).

The table below summarizes the key differences in treatment approaches:

Condition.	Stage Characteristics.	Primary Treatment Goal.	Typical Treatments.
Gingivitis.	Mild, early-stage inflammation of the gums. It is reversible and does not involve bone loss.	Remove the cause (plaque) and reverse the inflammation.	Improved daily oral hygiene (brushing, flossing), professional dental cleanings (scaling), and possibly antimicrobial mouthwash.
Periodontitis.	Advanced infection that damages the soft tissue and bone supporting the teeth. It is manageable but not curable.	Control the infection, halt disease progression, and regenerate lost support where possible.	Non-surgical (deep cleaning) and surgical procedures. Often requires ongoing maintenance.

Treatment Options for Periodontitis.

For periodontitis, treatment is more complex and often involves a stepwise approach:

Non-Surgical Therapy (The First Line of Defense).
This is the initial treatment for most periodontitis cases and focuses on deep cleaning to remove bacterial deposits below the gumline.

Scaling and Root Planing: A deep-cleaning procedure performed under local anesthesia. It involves scraping away plaque and tartar from below the gum line and smoothing the tooth root to help gums reattach.

Adjunctive Medications: Dentists may place antibiotics like chlorhexidine chips or minocycline (Arestin®) directly into the infected gum pockets to help control bacterial growth.

Surgical Therapy (For Advanced or Unresponsive Cases).
Surgery may be necessary when non-surgical treatments are insufficient, especially for deep pockets (>7 mm) that don't heal adequately.

Flap Surgery (Pocket Reduction): The gums are lifted back to allow for thorough cleaning of the tooth roots and bone. The gums are then sutured to fit more snugly around the teeth, reducing the depth of the pockets where bacteria thrive.

Regenerative Procedures: For cases with significant bone loss, techniques like bone grafting, guided tissue regeneration, or the use of growth proteins can help regenerate lost bone and tissue.

Soft Tissue Grafts: Used to cover exposed tooth roots or to augment thin, receding gums.

Key Takeaway.

Think of gum disease on a spectrum. Gingivitis is like a minor cut that can be cleaned and healed with good care. Periodontitis is a deep, infected wound that requires professional cleaning and possibly surgery to repair the underlying structural damage. Both stages require professional evaluation for an accurate diagnosis and a personalized treatment plan.

I hope this helps clarify the different approaches to treating gum disease. If you are experiencing symptoms like bleeding or swollen gums, it's always best to consult a dentist for a proper diagnosis.

Q24 Is gum treatment painful?

For most standard gum treatments, discomfort is usually well-managed, and the procedure itself is often not painful. However, you may experience some soreness afterward.

Here is a breakdown of what to expect with common gum treatments:

During the Procedure.

For a deep cleaning procedure like scaling and root planing, your dentist will typically use a local anesthetic to numb the area, so the treatment itself causes little to no pain. Some clinics also use ultrasonic tools, which can be more comfortable than traditional scraping tools.

Factors like your age, anxiety level, and the specific type of procedure can influence your perception of pain. If you're feeling nervous, it's a good idea to let your dentist know so they can help make you more comfortable.

After the Procedure.

It's common to have some discomfort once the anesthetic wears off. Here's what you might experience:

For deep cleanings: You may have pain, tenderness, and tooth sensitivity for a day or two, which can last up to a week.

For early gum disease (gingivitis): Some people experience discomfort when plaque and tartar are removed during a professional cleaning, but tenderness and bleeding typically lessen within 1 to 2 weeks with good home care.

For surgical procedures: Pain typically peaks around 12 hours after surgery and can last for a few days, reducing as healing progresses.

Managing Post-Treatment Discomfort.

Your dentist can help you manage any pain after treatment.

Medication: Your dentist may prescribe or recommend over-the-counter anti-inflammatory pain relievers.

Home care: Rinsing with warm salt water can reduce gum swelling.

Laser technology: Some newer treatments, like using an Er:YAG laser combined with mechanical cleaning, have been shown to significantly reduce pain after the procedure.

To put it simply, while modern techniques and numbing agents ensure that the treatment itself is usually painless, some post-procedure tenderness is normal and can be managed effectively with your dentist's help and at-home care.

Q25 Can gum surgery cause tooth loss?

The short answer is no, gum surgery does not directly cause tooth loss. In fact, the goal of the surgery is to treat the underlying gum disease that, if left untreated, would be the real cause of tooth loss .

Here's a more detailed breakdown of the relationship between gum surgery and tooth loss:

The Real Cause: Untreated Periodontal Disease.

The primary reason teeth are lost in patients with gum disease is the disease itself, not the treatment. Periodontal disease destroys the bone and tissue that support your teeth. When this support is lost, teeth can become loose and eventually fall out .

Gum surgery is a treatment designed to stop this process. Studies show that while patients who had surgery initially appeared to lose more teeth than those who didn't, this was because they had more severe disease to begin with. When accounting for the initial severity, the type of therapy (surgical vs. non-surgical) was not a significant predictor of tooth loss .

Predictors of Tooth Loss After Surgery.

Research has identified that the risk of losing a tooth after surgery is primarily linked to the condition of the tooth *before* the operation. A 2024 study found that the most significant risk factors were deep periodontal pockets and significant bone loss prior to surgery .

What the Data Shows.

The long-term outlook after surgery is generally positive, especially when followed by proper care:

Overall Survival: In one study, the survival rate for teeth five years after a flap surgery was 87% .

Patient Compliance: The most important factor for long-term success is your commitment to maintenance. Patients who attended regular follow-up appointments after surgery had a significantly lower rate of tooth loss compared to those who didn't .

Tooth Type: Molars (back teeth) have been shown to be at a higher risk of being lost compared to front teeth or canines .

Normal Post-Surgery Changes vs. Tooth Loss.

It's also important to note that some changes after surgery are normal and are not signs of tooth loss:

Increased Sensitivity: Your teeth may become more sensitive to temperature and pressure as the gums heal .

Increased Mobility: You might notice your teeth feel slightly more mobile right after surgery. This is typically temporary and should resolve as healing progresses .

Gum Recession: Some minor shrinkage of the gum tissue is expected, which can make the teeth look longer or create larger gaps between them. This is a normal part of healing, not a sign that a tooth will be lost .

Key to Success: Your Role After Surgery.

The success of gum surgery depends heavily on what happens after the procedure. The best way to ensure your teeth survive long-term is to:

Maintain Excellent Oral Hygiene: Brush and floss diligently to keep the surgical site clean.

Attend All Follow-up Appointments: Regular professional cleanings and check-ups are crucial to prevent the disease from returning .

To learn more about how to care for your teeth after surgery or what to expect during the healing process, feel free to ask.

Q26 Can gum treatment make my teeth feel loose?

The short answer is no. Gum treatment is actually meant to stabilize your teeth, not make them loose. In fact, research shows that treatment often helps to tighten teeth that have become loose due to gum disease.

The feeling of looseness you might be concerned about is more likely related to the underlying disease itself, or a temporary sensation during healing.

Why Treatment Doesn't Cause Looseness.

Gum disease, or periodontitis, is the real culprit behind loose teeth. It's an infection that destroys the bone and tissue anchoring your teeth in place. The primary goal of professional treatment is to remove the bacteria causing this destruction, which helps your gums heal and reattach to your teeth.

A 2024 study on the effects of non-surgical periodontal therapy (a deep cleaning called scaling and root planing) found that it is very effective at reducing tooth mobility.

71.2% of teeth with slight mobility (Class 1) became completely stable (Class 0) within a year.

Even with moderate mobility (Class 2), 86.3% improved to a lower, more stable class.

Why You Might *Feel* More Movement.

There are a few reasons why you might be worried about your teeth feeling loose in connection with treatment:

The Treatment is for the Underlying Cause: If your teeth are already loose from gum disease, the treatment isn't creating the problem it's addressing it. The disease has already caused some loss of bone support, which is why your teeth may feel less firm than before.

Temporary Sensation After Deep Cleaning: During a deep cleaning, your dentist removes hardened tartar (calculus) from below the gumline. This tartar can sometimes act as a splint, holding teeth together. Once it's removed, your teeth may feel different or slightly more mobile for a short time as your gums begin to heal. This is a normal part of the process, and your gums will tighten around the teeth as they heal.

The Goal is Stabilization: Gum treatment aims to prevent the disease from progressing and causing further bone loss. While it can significantly reduce looseness, it cannot fully replace the bone that has already been lost. This is why long-term maintenance visits with your dentist are so important to prevent the disease from returning.

I hope this clarifies how gum treatment works. It's a procedure designed to save your teeth by creating a healthier foundation, and the evidence shows it is successful in doing so. If you have specific concerns about your own teeth, it's always best to discuss them with your dentist, who can assess your individual situation.

Q27 Does tartar removal cause more tartar buildup?

This is a very common myth, and the short, definitive answer is no.

Professional tartar removal (scaling) does not cause more tartar to grow back. In fact, it is the only way to prevent serious buildup and gum disease.

Here is why this myth exists, what actually happens, and why your teeth might *feel* like they are getting dirtier faster after a cleaning.

Why the myth exists (What you are actually feeling):

Rougher enamel: During scaling, dental instruments can leave microscopic scratches on the tooth enamel. For the first 24 to 48 hours, these tiny scratches can feel slightly rougher to your tongue, making it *feel* like plaque is sticking to them faster. (Dentists usually polish the teeth afterward to smooth these out).

"Spaces" between teeth: Tartar acts like cement, filling in the gaps between your teeth. When the hygienist removes it, you suddenly feel wide, open spaces with your tongue. These gaps trap food more easily, making it *feel* like your teeth are getting dirty quicker, even though they aren't.

Sensitivity: Clean teeth transmit hot and cold sensations directly to the nerve, whereas a thick layer of tartar acts as an insulator. This new sensitivity is often mistaken for "weakened" teeth that are now more prone to damage.

What actually happens after a cleaning:

Slower buildup: A clean, smooth tooth surface is actually harder for plaque to adhere to than a rough, calcified tartar surface. Think of it like glass versus sandpaper; dirt slides off glass but gets stuck in sandpaper.

Biological reset: Tartar is calcified (hardened) bacteria. When it is removed, the harmful bacteria count in your mouth drops dramatically. It takes weeks for that specific destructive biofilm to rebuild to dangerous levels.

So, why does it seem like it comes back so fast?

Your saliva contains calcium and phosphate. Within 24 to 48 hours of a cleaning, a microscopic, naturally occurring film called the *acquired pellicle* coats your teeth. Within 48 hours, if you miss brushing, this film can start to harden into new calculus (tartar).

It is not growing back faster than before; it is simply that you are now hyper-aware of your teeth and notice the new film immediately, whereas you had stopped noticing the old buildup.

The real danger of skipping cleanings:

If you skip tartar removal because you fear it will cause more buildup, the tartar will continue to grow uninterrupted. Over time, it will creep below your gumline, create deep "pockets" around your teeth, and cause bone loss. That is what actually causes teeth to fall out not the cleaning itself.

The takeaway:
Get your teeth cleaned every 6 months (or as your dentist recommends). To prevent new tartar from forming quickly, wait 30 minutes after eating to brush (to avoid brushing acid into your enamel), and focus on flossing, as plaque turns into tartar most quickly between the teeth where your toothbrush can't reach.

Q28 Are antibiotics enough to treat gum disease?

No, antibiotics alone are not enough to treat gum disease. They are most effective when used as a secondary treatment alongside a professional, deep-cleaning procedure called scaling and root planing .

Here is why a combined approach is essential and how the treatments work together.

The Core Problem: The Bacterial Shield.

Gum disease is caused by a sticky film of bacteria called plaque (biofilm) that forms on your teeth. These bacteria can build up below the gumline, creating a physical barrier. Antibiotics taken orally cannot effectively penetrate this dense biofilm, so relying on them alone will not resolve the infection .

The Primary Treatment: Mechanical Disruption.

The foundation of gum disease treatment is physically removing this bacterial plaque and hardened tartar (calculus). This is typically done through:

Scaling and Root Planing (SRP): Often called a "deep cleaning," this is the first-line treatment. It involves carefully cleaning the tooth surfaces and smoothing the roots below the gums to allow the tissue to heal and reattach to the tooth .

The Supporting Role: Antibiotics as an "Adjunct".

Antibiotics act as an adjunctive, or supporting, treatment to SRP . After the dentist has physically disrupted the biofilm, antibiotics can help eliminate the remaining bacteria that have invaded the gum tissue or are in areas the instruments couldn't reach .

Do Antibiotics Add a Meaningful Benefit?

Research shows that adding antibiotics to SRP can significantly improve outcomes, but the benefit depends on the specific drug and the severity of your case.

Most Effective Combination: The combination of amoxicillin and metronidazole has been shown to be statistically superior to SRP alone. A large study found it significantly reduced bleeding, pocket depth, and the risk of disease worsening. For instance, it reduced the odds of worsening tooth mobility by 63% and furcation involvement by 50% .

Greatest Benefit in Severe Cases: The benefits of this antibiotic combination are most pronounced in patients with severe (Stage III/IV) periodontitis. For milder cases (Stage I/II), the added benefit is modest .

Not All Antibiotics Are Equal: Not every antibiotic provides the same level of benefit. For example, one study found that adding azithromycin provided no additional clinical benefit over SRP alone in patients with severe chronic periodontitis . Evidence for drugs like doxycycline is also less conclusive .

Key Points to Remember.

Not a First-Line Treatment: Antibiotics are generally reserved for when SRP alone isn't sufficient, or for aggressive forms of the disease. Dentists are cautious about prescribing them due to concerns about antibiotic resistance .

High-Risk Patients: For patients with diabetes, the amoxicillin-metronidazole combination has shown sustained improvements .

Follow-up is Crucial: Even with the best treatment, long-term success depends on a strict daily oral hygiene routine and regular professional maintenance to prevent the disease from returning .

In short, think of treating gum disease like cleaning a dirty wound. You must first clean out the debris (SRP) before the medicine (antibiotics) can be truly effective on the remaining infection.

I hope this clarifies the role of antibiotics in treating gum disease. If you're dealing with this condition, discussing these options with a dentist or periodontist is the best next step. Would you like to know more about what a scaling and root planing procedure involves?

Q29 After gum disease treatment, will I ever need treatment again?

Yes, it is very possible that you will need treatment again. Gum disease is a chronic condition, meaning it can be managed and controlled, but not permanently cured. The long-term success of your initial treatment depends heavily on a lifelong commitment to maintenance care .

Research shows that with consistent maintenance, many people can go years without needing further active treatment. Here’s what the long-term picture looks like:

The Long-Term Picture: What the Research Shows.

Key Finding.	Details.
Risk of Recurrence.	Treated patients remain at high risk of disease recurrence . Without proper follow-up, disease can return.
Need for Retreatment.	In a study with a 10-year follow-up, roughly 64% of patients who had one additional treatment needed a second one .
Time to Retreatment.	On average, the time between initial treatment and the first additional treatment was about 16.3 years .
Impact of Maintenance.	More frequent maintenance visits are linked to lower recurrence. Patients on 3-month intervals had an 8% recurrence rate, compared to 12% at 6 months and 20% at annual intervals .

The most important factor in determining whether your disease returns is your commitment to Supportive Periodontal Therapy (SPT), often called periodontal maintenance .

Why Maintenance is Critical.

Think of your initial treatment as getting a severe infection under control. The maintenance phase is the ongoing care needed to keep it from flaring up again.

It's a Lifelong Commitment: Managing periodontitis requires a lifelong commitment to regular SPT and excellent home care . Skipping these appointments allows harmful bacteria to re-establish themselves, leading to disease recurrence .

It's Personal: Your dentist will create a personalized maintenance schedule for you. More frequent visits (e.g., every 3 months) are often recommended for patients with a history of more severe disease or other risk factors .

Factors That Increase Your Risk.

Several factors can increase your likelihood of needing future treatment. Research highlights these key risks:

Initial Disease Severity: If you were diagnosed with advanced periodontitis (Stage III or IV), you are more likely to need retreatment sooner than someone with a milder case .

Smoking: Active smokers have a much higher risk of recurrence and tooth loss compared to non-smokers .

Overall Health: Conditions like diabetes can affect your body's ability to fight infection and heal, influencing the long-term stability of your gums .

Treatment Type: Some research suggests that patients who initially undergo surgery for their gum disease may have a higher likelihood of needing further surgical interventions later .

While the prospect of needing treatment again is real, remember that you have significant control over the outcome. By adhering to your dentist's recommended maintenance schedule and maintaining excellent oral hygiene at home, you can dramatically reduce your risk of recurrence and keep your gums healthy for the long term.

Q30 Is surgery always necessary for gum treatment?

No, surgery is not always necessary for gum treatment. In fact, non-surgical therapy is considered the foundational first step for managing gum disease, and for many people, it is sufficient on its own.

Here is a breakdown of the standard treatment approach for gum disease, which usually starts with non-surgical options.

The "Gold Standard": Non-Surgical Therapy.

The initial and primary treatment for periodontitis is a professional deep-cleaning procedure called scaling and root planing (SRP). This non-surgical approach is highly effective for many cases, especially when gum pockets are moderately deep (4 6 mm).

The goal of SRP is to remove plaque and tartar from below the gum line and smooth the tooth roots, which helps the gums reattach to the teeth. Research shows that this foundational therapy is effective in stabilizing the clinical attachment level (CAL) of teeth and controlling inflammation. A recent study on patients with Stage III periodontitis found that both quadrant-wise SRP and full-mouth disinfection approaches led to significant clinical improvements over six months.

Dentists also have a growing number of tools to enhance non-surgical outcomes. These advanced options can be used alongside SRP and include:

Host Modulation Therapy: This uses medications to help regulate the body's inflammatory response to bacteria, preserving bone and tissue. Examples include probiotics, omega-3 supplements, and low-dose NSAIDs, which have shown anti-inflammatory effects.

Photodynamic Therapy (PDT): This treatment applies a light-activated photosensitizer to periodontal pockets to selectively kill bacteria. In one study, PDT used as an adjunct to SRP reduced pocket depth by an average of 1.9 mm and significantly decreased bleeding.

Full-Mouth Disinfection (FMD): This approach completes the scaling and root planing for the entire mouth within 24 hours, often combined with antiseptic rinses to prevent re-infection from untreated areas.

When Surgery Becomes Necessary.

Surgery is typically considered a secondary step for more advanced cases, particularly when deep pockets (≥ 6 mm) remain after non-surgical treatment.

The main reasons to consider surgery include:

Treating Deep Pockets: Access flap surgery is more effective than non-surgical methods alone at reducing pocket depth in severe cases, as it allows the dentist direct visual access to clean root surfaces thoroughly.

Enabling Regeneration: Specific surgical techniques can be used to regenerate lost bone and gum tissue, which is not possible with non-surgical methods alone.

Addressing Recession: Procedures like gum grafting are used to cover exposed tooth roots caused by receding gums, which can reduce sensitivity and improve aesthetics.

The Long-Term Perspective.

It's important to note that long-term studies (over 3-5 years) show that the difference in outcomes between surgical and non-surgical treatments often diminishes. Both approaches are effective at stabilizing attachment levels and preventing tooth loss, although surgery provides a greater immediate reduction in deep pockets.

Crucially, the decision to operate is usually made after a healing period following non-surgical therapy. The current standard of care is to first use SRP and then evaluate the results. If pockets remain deep and inflamed, surgery may be recommended as the "third step" of a treatment plan.

Key Takeaway.

Modern periodontal care follows a stepwise, patient-centered approach. Non-surgical therapy is the primary treatment that works for a vast majority of cases, and surgery is considered only when it's needed to address specific issues, like deep pockets or the need for tissue regeneration.
