# Supplementary material for: Comparative Evaluation of Large Language Models in Responding to Common Misconceptions Regarding Periodontal Disease and Oral Hygiene
Source: Diagnostics (Basel). 2026 Sep 28;16(19):3158. doi: 10.3390/diagnostics16193158 (PMC13664004; doi:10.3390/diagnostics16193158)
Supplement: Supplementary file 1 [file diagnostics-16-03158-s001.zip › Supplementary File S5 LLMs response stylistic elements removed.zip!/2. LLMs response/Gemini 3.1.pdf]

Q1 Do gum diseases only affect elderly people?

No, gum diseases do not only affect elderly people. While severe gum disease is more common in older adults, the early stages of the disease can-and frequently do-affect people of all ages, including children and teenagers.

Children and Teenagers.

The earliest stage of gum disease, known as gingivitis, is incredibly common in children and adolescents.

Hormonal Changes: During puberty, an increase in sex hormones causes increased blood circulation to the gums, making them more sensitive and susceptible to irritation and inflammation from plaque.

Orthodontics: Braces and retainers make proper brushing and flossing much more difficult, frequently leading to plaque accumulation and gingivitis.

Pediatric Dentist Diet and Hygiene: High sugar intake combined with inconsistent brushing habits makes young people highly prone to early-stage gum inflammation.

If caught early, childhood gingivitis is entirely reversible with professional cleanings and improved daily hygiene. However, localized aggressive periodontitis-a more severe form involving bone loss-can occasionally affect healthy teenagers and young adults.

Adults (Ages 30 and Older).

As we enter adulthood, the risk of developing more advanced, destructive forms of gum disease increases significantly. According to the Centers for Disease Control and Prevention Nearly 47% (almost half) of all adults aged 30 and older have some form of periodontal (gum) disease.

Risk factors like smoking, diabetes, and prolonged periods without professional dental care drive these numbers up. For instance, more than 60% of current smokers have periodontal disease.

It is more prevalent in men (over 50%) than in women (around 35%).

Older Adults (Ages 65 and Older).

It is true that the highest rates of severe gum disease are found in older populations, but this is usually the result of a cumulative process rather than an inevitable part of aging.

Almost 60% to 70% of adults aged 65 and older have some form of periodontal disease.

Gum disease is chronic and cumulative. If mild gingivitis in a young adult is left untreated, the continuous presence of plaque and tartar will steadily destroy the connective tissues and bone over decades, culminating in severe periodontitis and tooth loss in their senior

years.

Age itself doesn't cause gum disease; rather, older individuals have simply had more time for the chronic inflammation to do structural damage to their teeth and jawbone.

Q2 Is it normal if my gums bleed when I brush my teeth?

This text is for informational purposes only. Consult a specialist for medical advice or diagnoses.

The short answer is no, it's not normal.

While it is very common, healthy gums should not bleed from standard brushing and flossing. Just like your scalp doesn't bleed when you wash your hair, your gums shouldn't bleed when you clean your teeth. Bleeding is your body's alarm system signaling that your gum tissue is inflamed or irritated.

Why is it happening?

Most of the time, bleeding gums point to a few easily fixable culprits:

Gingivitis (Early Gum Disease): This is the most common cause. A sticky film of bacteria called plaque constantly forms on your teeth. If it isn't thoroughly swept away by brushing and flossing, it accumulates at the gumline and irritates the tissue, making it swollen, red, and prone to bleeding.

Brushing too hard: Using a medium or hard-bristled toothbrush, or scrubbing your teeth aggressively, can physically tear the delicate gum tissue.

A new flossing routine: If you haven't flossed in a while, your gums will likely bleed for the first few days when you start again. This is normal and should subside within a week as the inflammation goes down.

Hormones or Medications: Hormonal shifts (like during pregnancy) can increase blood flow to the gums and make them more sensitive. Certain medications, like blood thinners, also make bleeding more likely.

What you should do.

The instinct for most people is to avoid brushing the area that bleeds. Don't do this. Avoiding the area allows more plaque to build up, which only makes the inflammation and the bleeding worse.

Instead, take these steps:

Keep brushing, but go soft: Switch to a soft-bristled toothbrush if you aren't already using one. Brush gently in circular motions angled toward the gumline rather than scrubbing vigorously back and forth.

Floss daily: Brushing only cleans about 60% of your tooth's surface. If you skip flossing, you are leaving bacteria sitting directly between your teeth and gums.

See your dentist: If plaque sits on your teeth for too long, it hardens into tartar (calculus). Once it turns into tartar, no amount of brushing at home will remove it. A dental hygienist needs to clear it away so your gums can finally heal and reattach tightly to your teeth.

If your gums continue to bleed after a week or two of consistent, gentle oral hygiene, it's definitely time to book a checkup to ensure it hasn't progressed to a more advanced stage of gum disease.

Q3 Can I get gum disease even if I brush my teeth regularly?

The short answer is yes, absolutely. While brushing twice a day is a fantastic foundation, it is actually quite common to develop gum disease even if you are diligent with your toothbrush.

Gum disease (gingivitis in its early stage, and periodontitis if it advances) is caused by a buildup of plaque a sticky film of bacteria. Brushing alone doesn't tell the whole story.

Why Brushing Alone Isn't Always Enough.

The Toothbrush Blind Spot: A toothbrush only cleans the front, back, and chewing surfaces of your teeth. It completely misses the tight spaces *between* your teeth and just under the gumline. If plaque sits in those gaps undisturbed, it hardens into tartar (calculus) within 24 to 72 hours. Once tartar forms, you cannot brush it away; only a dental hygienist can scrape it off.

Technique Matters: If you brush vigorously back-and-forth instead of using gentle, circular motions angled at 45 degrees toward your gums, you might actually be receding your gums rather than cleaning them effectively.

Other Hidden Risk Factors: Your mouth doesn't exist in a vacuum. Several systemic and genetic factors can make your gums vulnerable, even if your hygiene is pristine:

> Genetics: Some people naturally produce saliva with different mineral compositions or have an immune response that is highly sensitive to even tiny amounts of bacteria.
>
> Hormonal Changes: Pregnancy, menstruation, and menopause cause hormonal fluctuations that increase blood flow to the gums, making them much more reactive to plaque.
>
> Medical Conditions Medications: Conditions like diabetes affect how your body handles inflammation. Furthermore, hundreds of common medications cause dry mouth (xerostomia). Without enough saliva to wash away bacteria and neutralize acids, plaque multiplies rapidly.

Early Warning Signs to Watch For.

If you notice any of these, your gums are likely dealing with early-stage inflammation, even if your teeth look white and clean:

Gums that bleed when you brush or floss (healthy gums *never* bleed from normal cleaning).

Gums that look swollen, bright red, or tender.

Persistent bad breath or a bad taste in your mouth that doesn't go away after brushing.

Gums that look like they are pulling away from your teeth, making your teeth look longer.

How to Close the Protection Gap.

To truly protect your gums, you need to disrupt bacteria where a toothbrush can't reach:

Clean Between the Teeth Daily: Standard floss, interdental brushes (tiny little trees for wide gaps), or a water flosser are non-negotiable for gum health.

Get Professional Cleanings: Visiting a dentist every 6 months ensures that any hardened tartar is removed before it can trigger chronic inflammation.

Q4 Can I have gum disease even if my gums don't hurt?

Yes, absolutely. It is incredibly common to have gum disease without feeling any pain at all. In fact, dentists often refer to gum disease (periodontal disease) as a "silent" condition because it frequently progresses unnoticed until it reaches an advanced stage.

Because the gums don't have the same type of sensitive nerve endings as the inside of your teeth, early inflammation doesn't usually trigger pain signals.

If you aren't feeling pain, you have to look for other warning signs.

The Silent Warning Signs.

Even if your gums don't hurt, they might be trying to tell you something is wrong. Look out for:

Bleeding: Spitting blood when you brush or floss is the most common early sign of gum disease. Healthy gums do not bleed from normal cleaning.

Color changes: Healthy gums are usually light pink (or coral, depending on your complexion). Red, purplish, or swollen gums indicate inflammation.

Receding gums: If your teeth look longer than they used to, your gums may be pulling away from the teeth.

Persistent bad breath: Bacteria trapped in pockets below the gumline produce toxins that cause chronic bad breath or a bad taste in your mouth.

Changes in your bite: If your teeth feel slightly loose or like they fit together differently when you bite down, this is a sign of advanced bone loss.

The Two Stages of Gum Disease.

The progression of the disease explains why you might not feel it happening:

Stage.	What's Happening.	Pain Level.	Reversibility.
Gingivitis (Early).	Plaque builds up, causing mild inflammation on the surface of the gums.	Usually painless.	Fully reversible with good daily brushing, flossing, and a professional cleaning.
Periodontitis (Advanced).	Infection spreads below the gumline, destroying the tissue and bone that hold your teeth in place.	Rarely painful, unless a severe abscess (pus pocket) forms.	Irreversible, but can be halted and managed by a dentist.

Why the Dentist is Crucial.

Because you can't rely on pain to tell you when something is wrong, regular dental checkups are essential. During an exam, your dentist or hygienist will use a tiny ruler called a periodontal probe to measure the depth of the "pockets" between your teeth and gums.

Healthy pockets are 1 to 3 millimeters deep. Pockets measuring 4 millimeters or deeper are a physical sign of gum disease that you cannot see or feel at home.

Q5 Can gum disease get better without treatment?

The short answer is no, gum disease will not simply go away on its own.However, whether you can turn it around yourself or if you need to see a dentist depends entirely on how far it has progressed.

Gum disease is divided into two distinct stages:

Gingivitis (The Early, Reversible Stage).

If your gums are just slightly red, puffy, or bleed a bit when you brush or floss, you are likely dealing with gingivitis.

Can it get better without a dentist? Yes, but not without action. It requires an intense upgrade to your daily routine. Since gingivitis is an inflammatory response to plaque buildup, you can reverse it within a couple of weeks by strictly brushing twice a day, flossing daily (the step most people skip), and using an antibacterial mouthwash.

The Catch: If that soft plaque has already hardened into tartar (calculus), no toothbrush or floss on earth can remove it.At that point, you need a dental hygienist to scrape it away, or the inflammation will just keep going.

Periodontitis (The Advanced, Irreversible Stage).

If gingivitis is ignored, the bacteria sneak deep beneath your gumline, creating "pockets" and starting to destroy the actual bone holding your teeth in place. Signs include receding gums, persistent bad breath, pus, or loose teeth.

Can it get better without treatment? Absolutely not. Once gum disease reaches this stage, it is a chronic infection. At-home brushing and flossing cannot reach deep enough into those pockets to clear out the bacteria.

What happens next: Left completely untreated, periodontitis will steadily progress, eventually leading to tooth loss.It also causes systemic inflammation, which researchers have linked to broader health issues like heart disease and diabetes.While a dentist can't "cure" periodontitis (lost bone doesn't grow back), they can perform deep cleanings (scaling and root planing) to stop the damage in its tracks and save your teeth.

The Takeaway: If your gums are bleeding, give your brushing and flossing a major upgrade for two weeks. If things don't improve, it's time to book a cleaning before a minor issue turns into permanent damage.

Q6 Are gum diseases rare?

Not at all in fact, gum diseases are incredibly common. They are actually among the most widespread chronic conditions in the world.

According to data from the Centers for Disease Control and Prevention (CDC), nearly 47% of adults aged 30 and older in the United States have some form of periodontal (gum) disease. For adults over the age of 65, that number jumps to over 70%.

Gum disease generally progresses through two main stages:

Gingivitis: This is the early, mild form. It's an inflammation of the gums caused by plaque buildup. Your gums might bleed when you brush or floss, but at this stage, the damage is completely reversible with good oral hygiene and a professional cleaning.

Periodontitis: If gingivitis is left untreated, it can advance to periodontitis. The inflammation moves deeper, causing the gums to pull away from the teeth and form "pockets" that become infected. Over time, the body's immune response breaks down the bone and connective tissue holding your teeth in place, which can eventually lead to loose teeth or tooth loss.

Because early gum disease rarely causes pain, millions of people have it without even realizing it. Regular dental checkups are usually the first line of defense to catch it before it causes permanent damage.

Q7 Is it normal to lose teeth because of gum disease as you get older?

This text is for informational purposes only. Consult a specialist for medical advice or diagnoses.

The short answer is no, it is not a normal or inevitable part of aging but it is remarkably common.

While aging itself does not cause teeth to fall out, the risk of developing advanced gum disease increases over time. According to the CDC, over 70% of adults aged 65 and older have some form of periodontal (gum) disease. When left untreated, it becomes the leading cause of adult tooth loss.

Why Gum Disease Leads to Tooth Loss.

Tooth loss does not happen overnight. It is the result of a slow, progressive inflammatory process that damages the very foundation holding your teeth in place.

As shown in the diagram above, the progression moves through distinct phases:

Plaque and Calculus Build-up: Bacteria constantly form a sticky film (plaque) on your teeth. If it is not brushed away, it hardens into calculus (tartar), which can only be removed by a dentist.

Deepening Pockets: The bacteria in tartar irritate the gums, causing them to pull away from the tooth. This creates "deepening pockets" where more destructive bacteria can hide and multiply.

Bone Loss: As your immune system tries to fight off the infection, the chronic inflammation begins to break down the underlying jawbone and connective tissues ("bone loss").

Mobility and Loss: Once too much bone anchor is destroyed, the teeth lose their stability, become loose, and eventually fall out or must be extracted.

Why the Risk Creeps Up As We Age.

Several age-related factors make older adults more vulnerable to this cycle:

Dry Mouth (Xerostomia): Hundreds of common medications (for blood pressure, cholesterol, depression) cause dry mouth. Saliva is your mouth’s natural defense mechanism; without it, bacteria multiply much faster.

Wear and Tear: Receding gums are common as we age, exposing the softer root surfaces of the teeth, which are more susceptible to decay and bacterial buildup.

Physical Limitations: Conditions like arthritis or the aftermath of a stroke can make thorough brushing and flossing much more difficult.

The Good News: Periodontal disease is highly preventable and, in its early stages (gingivitis), completely reversible with proper oral hygiene and regular professional cleanings.

Q8 If gum disease runs in my family, will I definitely get it too?

The short answer isno, you are not definitely going to get it.

While having a family history increases your risk, genetics are only one piece of the puzzle. Think of it this way: your genes might load the gun, but your lifestyle and daily habits are what pull the trigger.

The diagram below shows exactly what happens when gum disease progresses from healthy tissue into periodontitis (advanced gum disease).

As you can see in the comparison, gum disease is ultimately driven by the buildup of plaque and calculus (hardened plaque), which triggers inflammation. If your family is prone to gum disease, it usually means your immune system might react more aggressively to that plaque, making you more susceptible to inflammation, a deepening pocket around the tooth, and eventual bone loss.

What You Can Control.

Because gum disease is primarily caused by bacterial plaque, meticulous oral hygiene can heavily counteract a genetic predisposition. You can significantly lower your risk by managing the factors within your control:

Flossing daily: Brushing only cleans about 60% of the tooth's surface. Flossing is what cleans the hidden pockets where the bacteria thrive.

Routine dental cleanings: Once plaque hardens into calculus (tartar), you cannot brush it off at home; it must be removed by a dental professional.

Avoiding tobacco: Smoking or vaping weakens your gums' ability to fight infections and heal, multiplying your risk.

Managing systemic health: Conditions like diabetes or high stress levels can increase systemic inflammation, making your gums more vulnerable.

Knowing your family history is actually a massive advantage because it allows you to be proactive with preventive care before any real damage to the bone occurs.

Q9 Can stress make me more likely to get gum disease?

The short answer is yes, absolutely. Stress is a major, often overlooked risk factor for gum disease (periodontitis).

When you are dealing with chronic stress, your body goes into a constant "fight or flight" mode, which triggers a biological domino effect that directly targets your oral health.

How Stress Attacks Your Gums.

Stress disrupts your mouth's ecosystem in three main ways:

It Spikes Your Cortisol Levels.

When you're stressed, your body pumps out a hormone called cortisol. While helpful in short bursts, chronic high cortisol acts like a systemic dampener on your immune system. It makes it

much harder for your body to fight off the harmful bacteria hiding in plaque, allowing them to multiply and inflame your gums more aggressively.

It Triggers Unconscious Habits.

Stress heavily influences our daily behavior. When we're overwhelmed, we are much more likely to:

Clench or grind our teeth (bruxism), which puts massive physical stress on the bone and tissue supporting your teeth.

Skip brushing or flossing at night.

Lean on comfort foods that are high in sugar or simple carbohydrates, which feed oral bacteria.

It Causes Dry Mouth.

Stress and anxiety alter your nervous system's control over saliva production. A dry mouth lacks the natural enzymes and washing mechanism that saliva provides to neutralize acids and clear away food particles, giving bacteria a free pass to thrive.

The Warning Signs: If your stress levels have been high, keep a close eye out for gums that bleed when you brush, persistent bad breath, or gums that look swollen and bright red instead of a healthy, pale pink.

Managing stress through sleep, exercise, or mindfulness isn't just good for your mental health it's a critical part of keeping your teeth firmly in place.

Q10 Do gum diseases only affect the mouth and teeth?

Not at all. While gum disease starts in the mouth, its effects can ripple across your entire body.

Your mouth isn't an isolated ecosystem. When you have advanced gum disease (periodontitis), your gums become inflamed, bleed, and form deep pockets around the teeth. This essentially creates an open doorway for oral bacteria and inflammatory chemical messengers to enter your bloodstream and travel to other organs.

Medical and dental researchers call this the oral-systemic connection.

Here is how users are looking stroll through how a localized infection in your mouth can impact your broader health:

Heart Disease and Stroke: The same bacteria found in diseased gums have been discovered inside the fatty plaques that clog human arteries. The chronic inflammation triggered by gum disease can irritate blood vessels, increasing the risk of blood clots, heart attacks, and stroke.

Diabetes (A Two-Way Street): This is one of the strongest links. Severe gum disease makes it much harder for your body to utilize insulin, causing blood sugar levels to spike. Conversely, poorly controlled diabetes makes you much more susceptible to severe oral infections.

Respiratory Infections: You constantly inhale air that passes through your mouth. If your mouth is heavily populated with harmful bacteria, you can accidentally inhale those microbes into your lungs, potentially leading to conditions like aspiration pneumonia, especially in older adults.

Rheumatoid Arthritis: Both periodontitis and rheumatoid arthritis are chronic inflammatory diseases. Studies suggest that the specific bacteria associated with gum disease can trigger the production of certain proteins that cause the immune system to attack the joints.

Treating gum disease isn't just about saving your smile or preventing tooth loss it is a foundational step in lowering chronic inflammation throughout your entire body.

Q11 Will a hard-bristled toothbrush clean my teeth better?

The short answer is no and it might actually do some serious damage.

While it feels like stiff bristles would scrub away plaque better (like using a heavy-duty brush on dirty grout), your teeth and gums require a much gentler touch.

Here is why dental professionals almost universally recommend avoiding hard-bristled brushes:

Enamel Wear: Your enamel the protective outer layer of your teeth is incredibly strong, but it is not invincible. Over time, the friction from hard bristles combined with abrasive toothpaste can wear down this layer, revealing the yellow dentin underneath and causing tooth sensitivity.

Gum Recession: Hard bristles can irritate and literally push back your gum line. Once gums recede, they do not grow back, leaving the vulnerable roots of your teeth exposed to decay and sensitivity.

Plaque is Soft: Plaque is actually a soft, sticky film of bacteria. It doesn't take massive scrubbing power to remove it; it just takes thorough, precise coverage.

What to Look For Instead

Bristle Type.	Recommendation.	Why.
Soft / Extra-Soft.	Highly Recommended.	Flexible enough to bend and clean just under the gumline (where plaque hides) without damaging tissue.
Medium.	Use with Caution.	Okay for healthy gums if you use a very light hand, but still carries a risk of tissue wear.
Hard.	Avoid.	Best kept for cleaning bathroom tiles or car parts, not your mouth.

The Real Secret to Clean Teeth: It's all about technique, not force. Tilt your brush at a 45-degree angle toward your gum line and use gentle, circular motions rather than scrubbing back and forth like a saw.

Q12 Should I stop brushing my teeth for a few days if my gums are bleeding?

This text is for informational purposes only. Consult a specialist for medical advice or diagnoses.

No, you should not stop brushing your teeth.

It completely makes sense why you would want to usually, when a part of your body is bleeding, leaving it alone to heal is the right move. But with gums, it's the exact opposite.

Bleeding gums are most commonly a sign of early gum disease (gingivitis), which is caused by a buildup of plaque along the gumline. Plaque is full of bacteria that irritate the gums, making them inflamed, swollen, and prone to bleeding.

If you stop brushing, that plaque stays on your teeth and multiplies. This will make the inflammation worse and cause your gums to bleed even more.

Here is what you should do instead to help them heal:

Switch to a soft-bristled brush. Medium or hard bristles can tear at already tender gums. Soft bristles are all you need to effectively sweep away plaque.

Brush gently. You don't need to scrub hard to clean your teeth. Use light, circular motions at a 45-degree angle toward the gumline.

Keep flossing. Flossing removes the plaque between teeth that your brush can't reach. Your gums might bleed a bit more when you first start, but as the plaque is cleared away and the inflammation goes down, the bleeding should stop within a week or two.

Use a saltwater rinse. Swishing with warm saltwater (half a teaspoon of salt in a cup of warm water) can help soothe irritated gums and keep the area clean.

When to see a dentist: If your gums continue to bleed after a week or two of consistent, gentle brushing and flossing, or if the bleeding is severe or painful, schedule a visit with your dentist. They can check for more advanced gum disease or tartar buildup (hardened plaque) that can only be removed with professional tools.

Q13 Do I need to brush my teeth after every meal to keep my gums healthy?

The short answer is no, you do not need to brush your teeth after every single meal to keep your gums healthy. In fact, brushing too frequently or at the wrong time can sometimes do more harm than good.

For optimal gum health, the baseline recommendation from dental organizations (like the American Dental Association) is to brush thoroughly twice a day for two minutes each time, and to floss at least once a day.

Here is why brushing after every meal isn't necessary, and what you should do instead:

The "Acid Trap" (Why to avoid brushing immediately)

When you eat, especially if your meal includes acidic foods or drinks (like citrus fruits, tomatoes, coffee, or soda), the acid temporarily weakens your tooth enamel. If you brush right away, you are essentially scrubbing that softened enamel while it's vulnerable.

The Rule: If you want to brush after a meal, wait at least 30 minutes to give your saliva time to naturally neutralize the acid and remineralize your enamel.

What to do after meals instead.

To keep plaque from building up along your gumline throughout the day without over-brushing, use these methods:

Rinse with water: Swishing water around your mouth after eating helps dislodge food particles and washes away sugars and acids.

Chew sugar-free gum: This stimulates saliva production, which is your mouth's natural defense mechanism against plaque-causing bacteria.

Focus your energy on doing an excellent job during your morning and nighttime brushing sessions making sure to gently massage the gumline at a 45-degree angle rather than rushing to brush every time you eat.

Q14 Even if I brush my teeth regularly, do I still need to get my gums checked?

The short answer is yes, absolutely.

Brushing your teeth perfectly every day is a massive part of oral health, but it only cleans about 60% of your teeth's surfaces and it can't reach what's happening beneath the gumline.

Here is why regular gum checks (periodontal exams) are still essential, even for stellar brushers:

Plaque Has Hiding Spots.

No matter how great your technique is, plaque can still build up in the deep grooves of your teeth and just below the gumline. Over time, saliva minerals turn this soft plaque into tartar (calculus). Tartar is rock-hard, and no toothbrush or floss on earth can remove it only a dental professional using specialized ultrasonic or hand scalers can.

Gum Disease Can Be Invisible.

Early gum disease (gingivitis) often doesn't hurt. You might have slight inflammation or minor bleeding when you floss that you barely notice. Left unchecked, it can quietely progress into periodontitis, which damages the bone supporting your teeth. A dentist or hygienist uses a tiny instrument called a periodontal probe to measure the "pockets" between your gums and teeth.

Healthy pockets: 1 to 3 millimeters deep.

Trouble signs: 4 millimeters or deeper mean the gums are pulling away, leaving space for bacteria to colonize where your brush can't reach.

Your Gums Reflect Your Total Body Health.

Gum health isn't just about your mouth. Systemic factors like genetics, stress, hormonal shifts, and conditions like diabetes directly impact how your gums react to bacteria. A dental professional looks at the color, texture, and shape of your tissue to catch these shifts early.

The Takeaway: Think of brushing as daily maintenance (like washing your car) and a gum check as the mechanic looking under the hood. Skipping the checkups means missing the early warning signs before they turn into painful, expensive problems.

Q15 Is brushing my teeth once a day enough to keep my mouth healthy?

This text is for informational purposes only. Consult a specialist for medical advice or diagnoses.

The short answer is no. While brushing once a day is certainly better than not brushing at all, it falls significantly short of what your mouth needs to stay genuinely healthy and avoid long-term dental issues.

The universal recommendation from dental professionals (including the American Dental Association) is to brush twice a day for two minutes and clean between your teeth (floss) once a day.

Why Once a Day Falls Short.

The Plaque Clock: Plaque a sticky, colorless film of bacteria forms on your teeth constantly. Brushing only once gives these bacteria a massive 24-hour window to multiply undisturbed.

The Tartar Trap: If plaque sits on your teeth for too long, minerals in your saliva cause it to harden into tartar (calculus) in as little as 24 to 72 hours. Once tartar forms, your toothbrush can no longer remove it; only a dental professional can scrape it off. Tartar buildup is a primary driver of gum disease.

Extended Acid Attacks: Every time you eat (especially carbohydrates and sugars), bacteria feed on the residual food particles and produce acids that erode your tooth enamel. Brushing only once leaves your enamel exposed to these acids for the vast majority of your day, dramatically increasing your risk of cavities.

Gum Inflammation: Leaving plaque undisturbed near the gumline causes your gums to become inflamed and bleed easily (gingivitis). Over time, this can progress to severe gum disease (periodontitis), which can eventually lead to bone and tooth loss.

The Bottom Line: Think of brushing like doing the dishes. If you only wash your dishes once a day, the food sits, hardens, and requires heavy scrubbing to remove. Brushing twice a day disrupts the bacteria before it can harden into tartar or eat away at your enamel.

Q16 Can flossing cause gum recession?

Yes, flossing *can* cause gum recession but only if you are doing it too aggressively or using the wrong technique.

When done gently and correctly, flossing is actually one of the best ways to prevent gum recession because it removes the plaque and bacteria that cause gum disease (the leading cause of receding gums).

How Flossing Can Cause Damage.

Gum tissue is delicate. If you repeatedly apply too much physical force, you can traumatize the gums, causing them to pull back over time. This usually happens through:

"Snapping" the floss: Forcing the floss down hard between tight teeth so that it snaps forcefully into the gum pocket.

Sawing motions: Pulling the floss back and forth aggressively directly against the gums, rather than hugging the sides of the teeth.

Using too much pressure: Pressing the floss deeply into the gum line until it hurts or bleeds profusely.

The "Shrinking Gum" Illusion: If you haven't flossed in a while, your gums are likely swollen and inflamed from mild gingivitis. When you start a healthy flossing routine, that inflammation goes away, and the gums shrink back down to their normal, tight size. To many people, this sudden drop in the gum line looks like recession, but it is actually just your gums healing from chronic swelling.

The Correct Technique (The "C-Shape").

To clean below the gum line without damaging the tissue, dental professionals recommend the "C-shape" technique.

Curve the floss: Once the floss is gently worked between your teeth, wrap it into a "C" shape against the side of one tooth.

Slide gently: Gently slide the floss up and down the side of the tooth, pushing it slightly below the gum line until you feel mild resistance.

Avoid the V: Do not drag the floss aggressively across the V-shaped point of gum tissue between your teeth (the interdental papilla).

Switch sides: Curve the floss around the neighboring tooth in the same gap and repeat the up-and-down motion.

A More Common Culprit.

While aggressive flossing can cause damage, brushing your teeth too hard is a much more common cause of physical gum recession. If you are experiencing recession, ensure you are using a soft-bristled brush and applying very light pressure.

Q17 Do electric toothbrushes damage the gums more than regular toothbrushes?

The short answer is no in fact, it's usually the exact opposite. When used correctly, electric toothbrushes are actually *safer* and gentler on your gums than manual ones.

The misconception comes from the power. Because electric toothbrushes vibrate or rotate thousands of times per minute, people assume they must be tearing up the soft tissue. However, dental studies consistently show that electric toothbrushes reduce gum inflammation (gingivitis) and bleeding significantly better than manual brushing.

Here is why manual brushes often cause more damage, and how electric ones protect you:

The "Scrubbing" Problem.

When people use manual toothbrushes, they tend to use a sawing, back-and-forth motion and press down way too hard. This aggressive scrubbing can lead to gum recession (where the gums pull back, exposing the sensitive tooth roots) and enamel wear.

Electric toothbrushes remove the need for that aggressive scrubbing. You simply glide the brush head from tooth to tooth, and the motor does the micro-movements for you.

Built-In Safety Features.

Most modern electric toothbrushes are specifically engineered to prevent gum damage through a few key features:

Pressure Sensors: Many models will flash a light, buzz, or completely slow down the motor if you press too hard against your teeth or gums.

Sensitive/Gum Care Modes: These settings lower the speed of the vibrations to provide a gentler clean for sensitive areas.

Timers: They ensure you brush for the dentist-recommended 2 minutes, preventing over-brushing in one spot.

The Golden Rule for Electric Brushes: Let the brush do the work. Hold it lightly like a pencil, place the bristles at a 45-degree angle toward your gum line, and just hold it on each tooth for a few seconds before moving to the next.

As long as you choose soft bristles (never medium or hard) and let the motor do the moving, your gums will thank you for making the switch.

Q18 Would using mouthwash alone be enough without brushing my teeth?

This text is for informational purposes only. Consult a specialist for medical advice or diagnoses.

No, using mouthwash alone is not enough to keep your teeth and gums healthy. While it is a great addition to your routine, it cannot replace brushing.

Here is why you still need to reach for your toothbrush:

The Sticky Problem: Dental Plaque.

The main reason we brush our teeth is to remove plaque a sticky, invisible film of bacteria that constantly forms on your teeth. Plaque is a "biofilm," meaning it clings tightly to the microscopic grooves of your tooth enamel and gums.

Because plaque is so sticky, it requires physical friction to remove. Mouthwash is just a liquid it can't penetrate or wash away that stubborn biofilm on its own.

Think of it like washing dishes: Imagine trying to clean a baked-on lasagna pan by just rinsing it with soapy water. The water might smell nice and wash away loose crumbs, but the baked-on food won't budge until you scrub it with a sponge. Your teeth work the same way!

What Mouthwash Actually Does.

Think of mouthwash as the finishing touch, not the main event. Depending on the formula you use, a good rinse acts as a supporting player:

Reaches missed spots: It washes over areas your toothbrush and floss might have missed.

Delivers fluoride: Many rinses leave a coating of fluoride to help strengthen enamel and prevent cavities.

Reduces bacteria: Antibacterial mouthwashes help reduce the loose bacteria that cause gingivitis and bad breath.

If you don't physically brush the plaque away, it eventually hardens into tartar (calculus) a rough, porous substance that only a dentist or hygienist can scrape off with specialized tools.

Q19 After I quit smoking, my gums started bleeding. Is that normal?

This text is for informational purposes only. Consult a specialist for medical advice or diagnoses.

First, congratulations on quitting smoking that is a massive step for your health. To answer your question directly:yes, this is completely normal.

While it feels alarming, bleeding gums after quitting are actually a positive sign that your body is healing. Dentists often refer to this as the "unmasking" phase.

Why This Happens.

The sudden bleeding is a result of your body's circulation waking back up:

How smoking hid the problem: Nicotine is a vasoconstrictor (it shrinks your blood vessels).While you were smoking, this restricted the blood flow to your mouth. Because there wasn't enough blood flow, the natural warning signs of plaque buildup and gum irritation like redness, swelling, and bleeding were completely suppressed.

The rush of recovery: Within just days of your last cigarette, your blood vessels dilate back to their normal size. Blood rushes back into your gum tissues, bringing a healthy surge of oxygen and immune cells.

The immune response: Your newly awakened immune system finally "sees" the bacterial plaque that accumulated while you were smoking and starts fighting it. Because your gums are now flooded with healthy blood, this battle causes them to swell, feel tender, and bleed when you brush or floss.

Essentially, your gums aren't bleeding because quitting caused damage; they are bleeding because your body's natural alarm system is finally working again to warn you about the plaque that was already there.

What to Do Next.

Don't let it discourage you. It can be tempting to think quitting made things worse, but going back to smoking will only restrict the blood vessels again and let the underlying infection silently destroy your gum tissue.

Keep brushing and flossing. Be gentle with your technique, but don't stop your daily oral hygiene routine to avoid the bleeding.

Book a dental cleaning. This is the critical next step. While your immune system is fighting back, a toothbrush can't remove the hardened tartar deep beneath the gumline. A professional cleaning removes that bacterial backlog, giving your newly oxygenated gums the clean slate they need to heal and firm up.

If the bleeding is still happening consistently after 3 to 4 weeks, it's a clear signal of active gum disease that needs a dentist's help to fully clear up.

Q20 Can natural products effectively keep my gums healthy?

This text is for informational purposes only. Consult a specialist for medical advice or diagnoses.

Yes, natural products can be highly effective at keeping your gums healthy- and there is a surprisingly robust body of clinical research backing them up.

While plant-based remedies won't magically fix advanced gum disease on their own, many contain powerful antimicrobial, anti-inflammatory, and antioxidant compounds.When used as an addition to your normal hygiene habits, they are excellent at preventing and managing gingivitis (early gum inflammation).

The Most Evidence-Based Natural Options.

Clinical trials have identified several botanicals that perform exceptionally well in the mouth. Here are the most heavily researched natural products for periodontal health:

Natural Product.	Active Mechanism.	Proven Gum Benefits.
Aloe Vera.	Contains soothing polysaccharides and anti-inflammatory enzymes.	Reduces gingival bleeding and plaque accumulation.
Green Tea.	Rich in EGCG, a highly potent antioxidant catechin.	Stops bacteria from sticking to teeth and lowers mouth acidity.
Tea Tree Oil.	Acts as a broad-spectrum natural antimicrobial.	Kills periodontal bacteria and reduces pocket depth around teeth.
Pomegranate.	High in punicalin and plant flavonoids.	Blocks inflammatory enzymes and stimulates tissue healing.
Propolis.	An antimicrobial resin produced by bees.	Inhibits gum pathogens and speeds up the repair of mouth tissue.

Natural vs. Conventional Treatments.

In clinical dentistry, the "gold standard" for treating gum inflammation is a prescription antimicrobial mouthwash called chlorhexidine (CHX).While CHX is highly effective at killing bacteria, long-term use is notorious for causing unpleasant side effects, including brown stains on your teeth and an altered sense of taste.

This is where natural products shine. Multiple clinical studies have shown that mouthwashes and gels made from aloe vera and green tea perform comparably to chlorhexidine in reducing plaque and bleeding, but without the tooth discoloration or taste alteration. This makes them ideal for safe, long-term daily maintenance.

The Reality Check: Mechanics Over Chemistry.

It is important to understand the limits of any product natural or synthetic.

Dental plaque is a sticky biofilm. If left on your teeth, it hardens into calculus (tartar). No mouthwash, gel, or herb can chemically dissolve tartar once it has hardened.

Gum health relies on a mechanical process first and a chemical process second. You must physically scrub the biofilm away using a toothbrush and floss. Natural products act as excellent adjuncts they soothe the tissue, reduce the bacterial load, and make it harder for new plaque to form but they cannot replace the mechanical friction of brushing or professional dental cleanings.

Q21 Can getting a dental scaling damage my teeth?

The short answer is no.When performed correctly by a dental professional, dental scaling (professional teeth cleaning) is completely safe and does not damage your teeth. In fact, skipping regular cleanings is what actually leads to permanent tooth and gum damage.

It is very common to worry that the scraping sounds and vibrations are harming your teeth, but this is a widespread misunderstanding of what the procedure actually does.

Debunking the Common Myths.

Many fears about dental scaling come from how your mouth feels *after* the tartar is gone, not from actual damage.

Myth: It scrapes away your enamel.Fact: Tooth enamel is the hardest substance in the human body it is much harder than bone. Dental scalers (both manual and ultrasonic water tools) are designed specifically to break the bonds of calculus (hardened tartar) without scratching the enamel underneath.

Myth: It creates gaps between your teeth.Fact: Scaling doesn't create gaps; it reveals them. When you have gum disease, tartar builds up and physically forces your gums to pull away from your teeth, filling the space. When the dentist removes that solid wall of tartar, you suddenly feel the gaps that the tartar created.

Myth: It makes your teeth loose.Fact: Tartar buildup causes infection that destroys the bone supporting your teeth. In severe cases, a thick bridge of tartar can actually act like a splint, holding loose teeth together. When the tartar is removed, the teeth may feel loose, but the scaling didn't cause the looseness the underlying gum disease did.

What You *Will* Actually Feel.

While scaling won't damage your teeth, it does temporarily change the environment in your mouth. You might experience a few normal, short-lived side effects:

Temporary Sensitivity: Tartar acts like an insulating blanket over your teeth. Once it's gone, your teeth are suddenly exposed to hot and cold temperatures again.This sensitivity is normal and usually fades within 1 to 3 days.

Mild Bleeding or Soreness: If your gums were already inflamed (gingivitis), they will likely be tender and might bleed slightly during and right after the cleaning. This goes away quickly as your gums heal and tighten back up around the tooth.

A "Rough" or "Different" Texture: Your teeth might feel strangely sharp or rough to your tongue. This is just your tongue feeling the natural, clean contours of your teeth after being used to a smooth, slippery coating of plaque and tartar.

The real risk: Leaving tartar on your teeth holds disease-causing bacteria directly against your gums. This triggers a constant state of inflammation that eventually eats away at your jawbone, leading to irreversible tooth loss.

Q22 Can dental scaling cause gaps between my teeth?

The short answer is no the dental scaling procedure itself does not create new gaps between your teeth or push them apart.

However, it is incredibly common to *feel* or *see* gaps immediately after a cleaning.This happens because the scaling reveals spaces that were already there, but had been temporarily hidden by buildup.

Why You Notice Gaps After Scaling.

Here is exactly what happens in your mouth to create the illusion of new gaps:

The Tartar "Wall": Over time, soft plaque hardens into a tough, cement-like substance called calculus (tartar). This tartar naturally accumulates in the small crevices between your teeth and along the gumline, essentially spackling over the natural gaps like plaster.

Gum Recession: As tartar builds up, the bacteria inside it continuously irritates your gums. Your gums become inflamed and slowly pull away from the teeth to escape the bacteria (gum recession), creating a larger physical gap underneath the tartar.

The Reveal: When the dental hygienist scales your teeth, they use specialized tools to chip away all that hardened tartar. Suddenly, the "wall" filling the gaps is gone, and the actual, current state of your teeth and gums is exposed.

Reduced Swelling: Before the cleaning, your gums were likely puffy and swollen from the bacterial infection.A few days after scaling, your gums begin to heal and the swelling goes down.As the inflamed tissue tightens up and becomes healthy again, the spaces between your teeth might look even more pronounced.

Why Scaling Actually Protects Your Teeth.

It can be alarming to suddenly feel air whistling between your teeth or to notice small "black triangles" near your gums when you look in the mirror. But leaving the tartar in place to fill those gaps is incredibly dangerous for your oral health.

If left untreated, tartar buildup causes periodontal disease. This infection slowly eats away at the underlying jawbone holding your teeth in place. As the bone deteriorates, the gaps become permanently larger, and your teeth can become loose or eventually fall out.

By getting your teeth scaled, you are stopping this destructive process in its tracks. You aren't losing tooth structure; you are just losing the hardened bacteria that was destroying your gums. In many cases, once the tartar is gone and you maintain good hygiene, healthy gums can reattach slightly to the teeth, naturally minimizing some of those spaces over time.

Q23 Do all gum diseases require the same treatment?

No, gum diseases do not all require the same treatment. The approach depends entirely on how far the disease has progressed and the extent of the damage.

Gum disease generally falls into two main categories: gingivitis (the early, reversible stage) and periodontitis (the advanced, irreversible stage that damages bone). Because the tissue and bone involvement is vastly different at each stage, dentists use a stepped approach to treatment.

Here is how treatment plans vary depending on the severity of the infection:

Gingivitis (Early Stage).

At this stage, the gums are inflamed and may bleed easily, but the bone and connective tissue holding the teeth in place are intact. Because no permanent damage has occurred, the goal is simply to reverse the condition.

Professional Dental Cleaning: A standard cleaning by a hygienist removes plaque and tartar (hardened plaque) from above and just below the gumline.

Improved At-Home Care: Brushing twice a day, flossing daily, and using an antibacterial mouthwash are usually enough to clear up the inflammation within a few weeks once the tartar is removed.

Mild to Moderate Periodontitis.

If gingivitis is left untreated, the inflammation moves deeper, causing the gums to pull away from the teeth and form "pockets" that trap bacteria. The bone begins to break down.Treatment here is non-surgical but much more intensive than a standard cleaning.

Scaling and Root Planing (Deep Cleaning): This is the gold standard for periodontitis. Scaling scrapes away plaque and tartar from above and below the gumline. Root planing smooths out the rough spots on the tooth roots, making it harder for bacteria to stick and helping the gums reattach to the tooth.

Antibiotic Therapy: Dentists may prescribe oral antibiotics or place slow-releasing topical antibiotics (like Arestin or PerioChip) directly into the gum pockets after a deep cleaning to eliminate persistent, localized infection.

Laser Therapy: Sometimes used as an alternative to traditional deep cleaning, dental lasers can remove diseased tissue and kill bacteria under the gums with less discomfort and quicker healing times.

Advanced Periodontitis.

At this stage, the pockets are deep, and significant bone and tissue loss has occurred.Teeth may become loose or shift out of place. Because non-surgical treatments can't reach deep enough to clean the roots or repair lost structure, surgery is often required.

Pocket Reduction Surgery (Flap Surgery): A periodontist makes small incisions in the gums to temporarily fold them back, exposing the roots for a thorough cleaning. The gums are then sutured snugly back around the tooth to eliminate deep pockets where bacteria hide.

Bone Grafts: If the disease has destroyed the jawbone surrounding the tooth root, fragments of your own bone, synthetic bone, or donated bone are placed in the area. This provides a scaffold for your body to regenerate new bone and stabilize the tooth.

Soft Tissue Grafts: When gums recede and expose the tooth roots, tissue is taken from the roof of your mouth and attached to the affected site to cover the roots, reduce sensitivity, and stop further recession.

Tooth Extraction: If a tooth is too loose and the supporting bone is too severely compromised to be saved, the tooth must be removed to prevent the infection from spreading to neighboring teeth.

Q24 Is gum treatment painful?

The short answer is no, the procedure itself shouldn't be painful, but you can expect some soreness and sensitivity for a few days afterward.

Because the gums are sensitive, dentists and periodontists take steps to make sure you are comfortable during treatment. Here is a breakdown of what you can expect during and after the most common gum treatment, a deep cleaning (also known as scaling and root planing).

During the Procedure.

Local Anesthesia: Before starting, your dentist will numb the area using a local anesthetic (like the one used when getting a filling). You won't feel sharp pain, though you will feel pressure, scraping sensations, and vibrations from the tools.

Topical Numbing: Sometimes, for very mild cases, a strong numbing gel applied directly to the gums is enough, meaning no needles are required.

Segmented Treatment: Deep cleanings are often broken up into two or four visits (e.g., doing one half of your mouth at a time). This prevents your entire mouth from being numb at once and makes the process much more manageable.

After the Procedure (The Recovery).

Once the numbing wears off, it is completely normal to experience some discomfort. This is usually mild and temporary.

Soreness and Aching: Your gums will likely feel tender or bruised for 1 to 3 days.

Tooth Sensitivity: Your teeth might be highly sensitive to hot, cold, or sweet foods and drinks. This is because the treatment removes tartar that was covering the roots of your teeth. This sensitivity can last anywhere from a few days to a couple of weeks.

Minor Bleeding: You might notice a little pink when you brush or spit for the first day or two.

How to Manage the Discomfort.

Most people find that the after-effects are easily manageable at home without needing prescription medication:

Over-the-Counter Relief: Ibuprofen (Advil) or acetaminophen (Tylenol) are usually highly effective for the soreness.

Salt Water Rinses: Gently rinsing your mouth with warm salt water 2-3 times a day helps soothe the gums and keep the area clean.

Soft Diet: Stick to soft, lukewarm foods (like yogurt, mashed potatoes, or soup) for the first 48 hours to avoid irritating your gums.

Gentle Brushing: Continue to brush and floss, but be extra gentle around the treated areas. Using a toothpaste designed for sensitive teeth can also help.

When to call your dentist: If you experience severe, throbbing pain, excessive bleeding, or if the discomfort lasts longer than a few weeks, you should reach out to your dental office.

Q25 Can gum surgery cause tooth loss?

The short answer is no in fact, the primary goal of gum surgery (periodontal surgery) is the exact opposite:to save your teeth from falling out.

However, people often associate gum surgery with tooth loss because the two are linked by the same underlying problem: severe gum disease (periodontitis).

Why the Confusion Happens.

While the surgery itself isn't what causes tooth loss, there are a few reasons why a tooth might be lost around the time of surgery, or why it might feel like you are at risk:

The tooth was already beyond repair: Sometimes, a periodontist opens the gums during surgery and discovers that the bone loss is much more severe than the X-rays showed. If there isn't enough bone left to support the tooth, they may have to extract it during the procedure to prevent massive infection.

Temporary looseness: Immediately after flap surgery or bone grafting, your teeth might actually feel *looser* for a few weeks. This is normal as the gums and bone heal, reduce in swelling, and reattach. It can be scary, but the teeth usually tighten back up as the area heals.

Post-surgical complications: As with any surgery, severe infection or poor healing can compromise the surgical site. This is rare but is significantly more common in patients who smoke or fail to follow strict post-operative hygiene instructions.

Common Side Effects (Not Tooth Loss).

Gum surgery does have common side effects that change the appearance and feel of your mouth:

Gum Recession: As the swollen, diseased gum tissue heals and becomes healthy, it shrinks and tightens up. This often causes the gumline to recede, making the teeth look longer and exposing the tooth roots.

Temperature Sensitivity: Because the tooth roots are newly exposed after the gums recede, your teeth may become temporarily sensitive to hot and cold.

If a dentist is recommending gum surgery, it means they believe there is a good chance your teeth can be saved. Without the surgery, the progressive bone loss from untreated periodontitis will eventually cause the teeth to become loose and fall out on their own.

Q26 Can gum treatment make my teeth feel loose?

Yes, it is incredibly common for teeth to feel slightly wobbly or loose after gum treatments like scaling and root planing (deep cleaning).While this sensation can be unsettling, the treatment itself didn't damage your teeth it simply uncovered their true condition.

Why Your Teeth Feel Loose.

When you have periodontal (gum) disease, two things happen that artificially hold your teeth in place:

The "Tartar Cast": Over time, hardened plaque (tartar or calculus) builds up below the gumline. In advanced stages of gum disease, this heavy buildup acts almost like a concrete splint, cementing weakened teeth together.When your dentist removes this tartar, that false structural support disappears.

Inflammation: Infection causes your gums to become chronically swollen and inflamed, which physically props the teeth up.After a deep cleaning, the infection is cleared and the swelling subsides.As the gums shrink back to a healthy size, the teeth can temporarily feel less secure.

Before vs. After Treatment.

Feature.	Before Gum Treatment.	After Gum Treatment.
Tartar.	Heavy buildup acts as an artificial splint.	Removed, revealing the tooth's true mobility.
Gums.	Swollen and inflamed, propping teeth up.	Swelling drops; gums heal and shrink to a normal size.
Bone.	Actively deteriorating from bacterial infection.	Infection halts, allowing healthy gums to reattach to the tooth.

Will They Tighten Back Up?

In most cases, yes.As your gums heal over the weeks following your procedure, the tissue will begin to reattach tightly to the newly cleaned root surfaces.As this happens, your teeth should start to feel much more stable.

However, if the gum disease went untreated for a long time, the infection may have permanently destroyed some of the underlying jawbone. If bone loss is severe, the looseness might persist even after the gums heal.If this happens, your dentist or periodontist has ways to help stabilize your bite:

Splinting: Bonding a wobbly tooth to strong neighboring teeth so they can share the chewing load.

Bite Adjustment: Slightly reshaping the biting surface of the tooth so it doesn't take the brunt of the pressure when your mouth is closed.

Regenerative Therapy: Using bone grafts or special proteins to help your body rebuild the lost bone over time.

The Bottom Line: It is completely normal to feel anxious if your teeth feel loose right now, but your gum treatment is actively saving your smile. Leaving the tartar in place would have allowed the infection to continue eating away at your jawbone, eventually guaranteeing permanent tooth loss.

Q27 Does tartar removal cause more tartar buildup?

No, this is a common dental myth. In fact, professional tartar removal (scaling) does the exact opposite it makes it harder for new tartar to stick to your teeth.

Here is why so many people believe this myth, and what is actually happening in your mouth.

Why It *Feels* Like Tartar Comes Back Faster.

It is completely normal to feel like your teeth are suddenly magnets for buildup right after a cleaning, but it comes down to contrast and sensation:

The "Empty Space" Illusion: Over time, tartar fills in the natural microscopic gaps between your teeth and along your gumline. When a hygienist removes it, those spaces are suddenly open again. As new tartar begins to form, you feel it filling those spaces much more noticeably than before.

The Contrast Effect: After a cleaning, your teeth are perfectly smooth. Any new plaque or roughness is immediately obvious to your tongue. Before the cleaning, your teeth were already coated, so a little new buildup didn't feel like a big change.

Habits Haven't Changed: If the brushing and flossing routine that allowed tartar to build up in the first place doesn't improve after the cleaning, the tartar will simply return at its natural, rapid pace. It isn't returning faster it is just returning at its normal speed.

What Actually Happens During a Cleaning.

When a dentist or hygienist cleans your teeth, they do more than just scrape away the hard deposits.The procedure usually ends with polishing.

Polishing smooths out the surface of your tooth enamel. Because plaque (the sticky precursor to tartar) is just a bacterial film, it has a very hard time clinging to a perfectly smooth surface.

If you *don't* get your teeth cleaned, the existing rough tartar acts like a coral reef.Its porous surface gives new bacteria the perfect foundation to latch onto, multiply, and create even more tartar.

The Reality: Tartar Works Fast.

The reason tartar seems to "rush" back isn't because of the cleaning, but because of how fast the biological process happens in your mouth if you miss a spot while brushing.

Soft Plaque Forms.

0 24 Hours.

A soft, sticky film of bacteria forms on your teeth. At this stage, it can be completely removed with normal brushing and flossing.

Mineralization Begins.

24 72 Hours.

If plaque isn't brushed away, calcium and phosphate from your saliva begin to bind to it, starting the hardening process.

Fully Hardened Tartar.

10 14 Days.

The plaque has completely calcified into tartar (calculus). It is now bonded to the enamel and can only be removed with professional dental tools.

Q28 Are antibiotics enough to treat gum disease?

No, antibiotics alone are not enough to treat gum disease.While they can temporarily suppress bacterial growth and reduce inflammation, they cannot cure the infection or replace the need for professional dental treatment.

The Biofilm Shield.

Gum disease is driven by bacteria that live inside plaque and tartar (calculus). When plaque hardens into tartar beneath the gumline, it creates a highly protected ecosystem called a biofilm.

Antibiotics whether taken as an oral pill or applied as a gel cannot effectively penetrate this hardened, structural barrier. If the tartar isn't physically removed, the bacteria will survive inside those deposits and rapidly multiply again as soon as the course of antibiotics is finished.

The Gold Standard: Mechanical Cleaning.

Because medication can't break through the tartar, the foundation of all gum disease treatment is the physical removal of the bacteria and their habitat.

Scaling and Root Planing: Often called a "deep cleaning," this is a non-surgical procedure where a dentist or periodontist uses specialized instruments to scrape away the tartar from above and below the gumline.They then smooth the roots of the teeth so the inflamed gums can heal and reattach tightly to the tooth.

Ongoing Maintenance: Gum disease is a chronic condition.Long-term success relies on excellent daily brushing and flossing to prevent new biofilm from hardening, combined with routine professional cleanings.

When Are Antibiotics Actually Used?

Antibiotics are sometimes used as an adjunct (a helper) to a deep cleaning, but they are never a substitute for it. A dental professional might prescribe them in specific, severe scenarios:

Aggressive Periodontitis: The disease is progressing exceptionally fast and not responding to standard cleaning alone.

Stubborn Pockets: Severe cases where the pockets between the gums and teeth are too deep to clear out entirely with instruments, and bacteria keep recolonizing.

Systemic Symptoms: The infection has spread beyond the gums, causing fever or swollen lymph glands.

When antibiotics are necessary, dentists often prefer topical applications such as biodegradable antimicrobial chips or antibiotic microspheres placed directly into the infected gum pockets right after a deep cleaning.This delivers a high dose of medicine exactly where it's needed over several days, while minimizing full-body side effects and reducing the risk of antibiotic resistance.

Q29 After gum disease treatment, will I ever need treatment again?

The short answer is yes. While initial gum disease treatments like deep cleanings (scaling and root planing) or surgery are highly effective at stopping the active infection, advanced gum disease (periodontitis) is a chronic condition.

You don't usually "cure" it forever; rather, you manage it to keep it in remission and prevent further bone and tooth loss.

The New Normal: Periodontal Maintenance.

Once your initial treatment is finished and your gums have healed, you will likely be placed on a strict cleaning schedule known as periodontal maintenance.

This replaces the standard six-month dental cleaning you may be used to.

	Standard Dental Cleaning.	Periodontal Maintenance.
Who it's for.	People with healthy gums.	People with a history of gum disease.
Frequency.	Every 6 months.	Every 3 to 4 months.
Focus.	Cleaning teeth above the gumline.	Cleaning teeth both above and deeply below the gumline.
Monitoring.	General check for cavities and health.	Measuring "pocket depths" around teeth to track disease progression.

Why every 3 to 4 months?

The bacteria that cause gum disease multiply and repopulate the deep pockets under your gums every 90 to 120 days. Even with excellent brushing and flossing, your at-home tools simply cannot reach deep enough to clean out these pockets. Regular maintenance visits disrupt this bacteria before it can start destroying tissue and bone again.

Will you ever need the "major" treatments again?

If you stick to your periodontal maintenance schedule and maintain excellent daily oral hygiene, you may never need another major intervention (like scaling and root planing or flap surgery).

However, you might require retreatment if the disease flares up. A relapse is most common when:

Appointments are missed: Skipping maintenance visits allows the aggressive bacteria to re-establish themselves.

Home care slips: Inconsistent brushing, flossing, or using prescribed interdental brushes allows plaque to harden into tartar.

Tobacco use: Smoking drastically reduces blood flow to the gums, masking the signs of infection and severely impairing the healing process.

Health changes: Conditions that affect your immune system, particularly uncontrolled diabetes, make it much harder for your body to fight off gum infections.

Q30 Is surgery always necessary for gum treatment?

No, surgery is not always necessary to treat gum disease.In fact, dentists and periodontists almost always start with the least invasive options.

Whether you need surgery depends entirely on how advanced the disease is and how your gums respond to initial, non-surgical therapies.

The First Line of Defense: Non-Surgical Options.

For early-stage gum disease (gingivitis) and mild to moderate periodontitis, non-surgical treatments are often highly successful at halting the infection.

Scaling and Root Planing: Commonly known as a "deep cleaning," this is the gold standard for treating early gum disease.A dental professional removes plaque and hard tartar from below the gumline (scaling) and then smooths the tooth roots (planing). This prevents bacteria from easily reattaching and helps the gums heal tightly against the teeth.

Antibiotic Therapy: Often combined with a deep cleaning, your dentist might place topical antibiotics (like a slow-release gel or chip) directly into the gum pockets or prescribe oral antibiotics to control the bacterial infection.

Laser Therapy (LANAP): Some practices use specialized lasers to remove diseased tissue and kill bacteria inside the gum pockets.It is a highly effective, minimally invasive alternative to traditional scalpel surgery, usually resulting in less discomfort and a faster recovery.

When Surgery Becomes Necessary.

Dentists measure the health of your gums by checking "pocket depths" the space between your tooth and the surrounding gum tissue. Healthy pockets are 1 to 3 millimeters deep.

Surgery is generally recommended when non-surgical methods aren't enough to stop the infection. This usually happens in a few specific scenarios:

Pockets remain 6mm or deeper: At this depth, standard dental instruments simply cannot reach the bottom of the pocket to thoroughly clean out the bacteria and tartar without lifting the gum tissue surgically.

Significant bone loss: If the disease has started eating away at the jawbone, surgery (like bone grafting or guided tissue regeneration) is needed to rebuild the lost support structure so the teeth don't fall out.

Severe gum recession: If the gums have pulled back so far that the tooth roots are dangerously exposed, a periodontist may need to perform a gum graft to cover the roots, reduce sensitivity, and protect against decay.

The standard protocol: Most dentists will perform a deep cleaning first, then wait 6 to 8 weeks to re-evaluate. If your pockets shrink to a healthy depth and the bleeding stops, you've succeeded and move to standard maintenance. If deep pockets and inflammation persist, surgery becomes the necessary next step to save your teeth.
