# Supplementary material for: Comparative Evaluation of Large Language Models in Responding to Common Misconceptions Regarding Periodontal Disease and Oral Hygiene
Source: Diagnostics (Basel). 2026 Sep 28;16(19):3158. doi: 10.3390/diagnostics16193158 (PMC13664004; doi:10.3390/diagnostics16193158)
Supplement: Supplementary file 1 [file diagnostics-16-03158-s001.zip › Supplementary File S5 LLMs response stylistic elements removed.zip!/2. LLMs response/GPT-5.5.pdf]

Q1 Do gum diseases only affect elderly people?

No. Gum disease can affect people of almost any age, although it becomes more common as people get older.

There are two main stages:

Gingivitis: Early gum disease. It causes red, swollen, or bleeding gums and can occur in children, teenagers, and adults.

Periodontitis: A more advanced form that damages the tissues and bone supporting the teeth. It is more common in adults, especially if gingivitis goes untreated.

Risk factors include:

Poor oral hygiene .

Smoking or tobacco use .

Diabetes .

Hormonal changes (such as during pregnancy) .

Certain medications .

Genetic predisposition .

Dry mouth .

Stress and some medical conditions .

Signs of gum disease can include:

Bleeding when brushing or flossing .

Red, swollen, or tender gums .

Persistent bad breath .

Receding gums .

Loose teeth (in more advanced cases) .

While older adults have a higher overall risk because gum disease can progress over many years, it is not an "elderly-only" condition. Even teenagers and young adults can develop gum disease if risk factors are present.

If you're concerned about your own symptoms, I can help you figure out whether they sound more like gingivitis, periodontitis, or something else.

Q2 Is it normal if my gums bleed when I brush my teeth?

Occasional bleeding can happen if you brush too hard, start flossing after a long break, or irritate your gums. However, gums that bleed regularly when brushing are not usually considered normal and often indicate gum inflammation.

Common causes include:

Early gum disease (Gingivitis) .

More advanced gum disease (Periodontitis) .

Brushing too aggressively or using a hard-bristled toothbrush .

Poor oral hygiene leading to plaque buildup .

Hormonal changes (such as during pregnancy) .

Certain medications (especially blood thinners) .

Less commonly, nutritional deficiencies or medical conditions .

A few things you can do:

Use a soft-bristled toothbrush.

Brush gently in small circular motions.

Continue flossing daily if you recently started; mild bleeding often improves within 1 2 weeks as gum health improves.

Schedule a dental checkup and cleaning if the bleeding is recurrent.

You should seek dental evaluation sooner if:

The bleeding has been happening for more than about a week or two.

Your gums are swollen, red, tender, or receding.

You have loose teeth, persistent bad breath, or pus around the gums.

You experience unusual bleeding elsewhere (such as frequent nosebleeds or easy bruising).

How often does the bleeding happen, and is it only when brushing, or also when flossing or eating?

Q3 Can I get gum disease even if I brush my teeth regularly?

Yes. Brushing regularly lowers your risk of gum disease, but it doesn't eliminate it.

Gum disease develops when bacteria in dental plaque accumulate along and below the gumline. Even if you brush twice a day, several factors can still contribute:

Not cleaning between teeth: Brushing alone often misses plaque between teeth. Flossing or using interdental brushes helps remove it.

Plaque hardening into tartar: Once plaque hardens, it can't be removed by brushing and usually requires professional cleaning.

Brushing technique: You may be brushing regularly but not effectively reaching the gumline or all tooth surfaces.

Smoking or vaping: Tobacco use significantly increases gum disease risk.

Medical conditions: Conditions such as Diabetes can make gum disease more likely.

Hormonal changes: Pregnancy, puberty, and menopause can affect gum health.

Genetics: Some people are more susceptible despite good oral hygiene.

Dry mouth: Reduced saliva can allow bacteria to thrive.

Early gum disease (gingivitis) may cause:

Red, swollen, or tender gums .

Bleeding when brushing or flossing .

Persistent bad breath .

If it progresses to periodontitis, you may notice:

Receding gums .

Loose teeth .

Changes in your bite .

If you're brushing regularly but still notice bleeding, swelling, or bad breath, it's a good idea to see a dentist for an evaluation and cleaning. Those symptoms can be early signs that plaque or tartar is accumulating in places your toothbrush can't reach.

Are you experiencing any gum symptoms, or are you asking generally?

Q4 Can I have gum disease even if my gums don't hurt?

Yes. Gum disease can be present even when your gums don't hurt.

In fact, early gum disease called Gingivitis often causes little or no pain. Common signs may include:

Gums that bleed when brushing or flossing .

Red, swollen, or puffy gums .

Persistent bad breath .

Gums that seem to be pulling away from the teeth .

Increased sensitivity in some areas .

More advanced gum disease Periodontitis can also progress with surprisingly little discomfort until significant damage has occurred. Signs can include:

Receding gums .

Loose or shifting teeth .

Changes in your bite .

Pus between teeth and gums .

Ongoing bad breath or bad taste .

Pain is often a late symptom rather than an early one.

If you're noticing bleeding, gum recession, loose teeth, or other changes, it's a good idea to have a dentist examine your gums. They can measure the spaces around your teeth (gum pockets) and check for bone loss to determine whether gum disease is present.

What symptoms, if any, are you noticing? That can help narrow down how concerning the situation might be.

Q5 Can gum disease get better without treatment?

It depends on the stage of the gum disease.

Early gum disease (gingivitis) can often improve and even reverse if you remove the cause usually plaque buildup. Better brushing, flossing/interdental cleaning, and regular professional cleanings can allow the gums to heal. Symptoms such as redness, swelling, and bleeding may improve within days to weeks.

More advanced gum disease (periodontitis) generally does not get better on its own. Once the supporting tissues and bone around the teeth are damaged, that damage typically cannot be fully reversed without professional treatment. The disease may temporarily seem less noticeable, but it can continue progressing.

Signs that warrant a dental evaluation include:

Gums that bleed regularly .

Persistent bad breath .

Receding gums .

Loose teeth .

Pus around the gums .

Pain when chewing .

If you're noticing bleeding gums but haven't been diagnosed with periodontitis, improving oral hygiene right away may help significantly. If symptoms persist for more than a couple of weeks, it's a good idea to see a dentist or periodontist for an assessment.

What symptoms are you experiencing bleeding, swelling, gum recession, loose teeth, or something else?

Q6 Are gum diseases rare?

No, gum diseases are very common.

The two main forms are:

Gingivitis – the early stage, where gums become red, swollen, or bleed easily when brushing or flossing.

Periodontitis a more serious form that can damage the tissues and bone supporting the teeth, potentially leading to tooth loss.

Studies around the world consistently find that:

Mild gum inflammation (gingivitis) affects a large portion of adults at some point.

More severe periodontitis affects hundreds of millions of people globally and is one of the most common chronic diseases.

Risk factors include:

Inadequate oral hygiene .

Smoking or tobacco use .

Diabetes .

Certain medications .

Hormonal changes (such as during pregnancy) .

Genetics .

Increasing age .

Common warning signs are:

Bleeding gums .

Persistent bad breath .

Red, swollen, or tender gums .

Receding gums .

Loose teeth (in more advanced cases) .

If you're asking because of symptoms you're experiencing, I can help you understand what they might suggest and when it's worth seeing a dentist.

Q7 Is it normal to lose teeth because of gum disease as you get older?

No. While gum disease becomes more common with age, losing teeth is not considered a normal or inevitable part of aging.

The most common cause of tooth loss in older adults is advanced gum disease, called Periodontitis. In periodontitis, bacteria and inflammation gradually damage the gums, bone, and supporting tissues that hold teeth in place. If untreated, teeth can become loose and eventually fall out or require extraction.

Signs of gum disease can include:

Bleeding when brushing or flossing .

Red, swollen, or tender gums .

Receding gums (teeth look longer) .

Persistent bad breath .

Loose or shifting teeth .

Pain when chewing .

Many people keep their natural teeth throughout their lives with:

Regular brushing and flossing .

Professional dental cleanings .

Treatment of gum disease when it develops .

Avoiding tobacco use .

Managing conditions such as Type 2 Diabetes, which can worsen gum disease .

If you're noticing loose teeth, gum recession, bleeding gums, or tooth loss, it's worth seeing a dentist promptly. Early treatment can often stop gum disease from progressing and may help preserve teeth.

Are you asking because you've already lost a tooth, or are you noticing symptoms such as bleeding gums or loose teeth?

Q8 If gum disease runs in my family, will I definitely get it too?

No. A family history of gum disease increases your risk, but it does not mean you will definitely develop it.

Gum disease (also called Periodontal disease) is influenced by both genetics and environment. Some inherited traits can affect how your immune system responds to the bacteria that cause gum disease, making some people more susceptible than others. However, genetics is only one part of the picture.

Other important factors include:

Oral hygiene habits (brushing and flossing) .

Regular dental cleanings and checkups .

Smoking or tobacco use .

Diabetes and certain other health conditions .

Some medications that affect saliva or gum health .

Diet and overall health .

Even if several relatives have had gum disease, good preventive care can significantly reduce your chances of developing severe disease.

If gum disease runs in your family, dentists often recommend:

Consistent brushing with fluoride toothpaste twice daily .

Cleaning between teeth daily (floss, interdental brushes, etc.) .

Regular professional cleanings and periodontal evaluations .

Avoiding smoking .

Seeking dental care promptly if you notice bleeding gums, persistent bad breath, gum recession, or loose teeth .

If you'd like, I can also explain how much genetics contributes to gum disease risk and whether genetic testing is useful.

Q9 Can stress make me more likely to get gum disease?

Yes. Stress can increase your risk of gum disease, although it is usually one factor among several rather than the sole cause.

Here's how stress may contribute:

Weakened immune response: Chronic stress can make it harder for your immune system to control the bacteria that naturally live in the mouth.

Increased inflammation: Stress hormones such as cortisol can promote inflammation, which plays a major role in gum disease.

Changes in behavior: People under stress may be more likely to skip brushing or flossing, smoke, consume more sugary foods, grind their teeth, or delay dental visits.

Dry mouth: Stress and some medications used during stressful periods can reduce saliva flow. Saliva helps protect teeth and gums by washing away bacteria.

Early signs of gum disease can include:

Red, swollen, or tender gums .

Bleeding when brushing or flossing .

Persistent bad breath .

Receding gums .

Loose teeth (in more advanced cases) .

The good news is that reducing stress, maintaining good oral hygiene, and getting regular dental care can help lower your risk. If you've noticed bleeding gums, swelling, or other symptoms, it's worth scheduling an examination with a dentist, since gum disease is often easier to treat when caught early.

Are you experiencing gum symptoms now, or are you asking about stress in general?

Q10 Do gum diseases only affect the mouth and teeth?

No gum diseases don’t stay confined to the mouth.

The most common form, often referred to as Periodontal disease, starts in the gums and tissues supporting the teeth. If it progresses, it can lead to gum recession, bone loss, and tooth loss. But its effects can extend beyond the mouth.

Research has shown strong links between gum disease and several systemic health conditions. The inflammation and bacteria involved can enter the bloodstream and contribute to issues elsewhere in the body. Associations have been found with:

Heart and blood vessel disease (including increased risk of heart attack and stroke) .

Diabetes (gum disease can make blood sugar harder to control, and diabetes can worsen gum disease) .

Respiratory problems (bacteria may be inhaled into the lungs, especially in vulnerable people) .

Pregnancy complications (such as preterm birth and low birth weight) .

That said, gum disease is not usually described as a direct cause of these conditions. It's more accurate to say it can *increase risk* or *worsen existing health problems*, mainly through chronic inflammation and bacterial spread.

If you want, I can explain how to tell early gum disease from more advanced stages, since the early signs are often easy to miss.

Q11 Will a hard-bristled toothbrush clean my teeth better?

Not really in most cases, a hard-bristled toothbrush does not clean your teeth better, and it can actually do more harm than good.

Dentists generally recommend a soft-bristled toothbrush because it removes plaque effectively when used properly, while being gentle on your gums and enamel. The key to cleaning isn't bristle hardness it's technique + time + consistency.

Why hard bristles aren't usually better .

They can wear down enamel over time .

They may cause gum recession (gums pulling back from teeth) .

They can increase tooth sensitivity .

They don't remove significantly more plaque than soft brushes when brushing is done correctly .

What actually works best

Soft bristles gentle circular motions .

Brushing for 2 minutes, twice a day .

Cleaning along the gumline (where plaque builds up most) .

Using floss or interdental cleaners for between teeth .

There are a few rare cases where a dentist might suggest a firmer brush (for example, certain dentures or heavy staining), but for natural teeth, soft is the standard recommendation.

If you want, I can also tell you how to choose the best toothbrush for your specific situation (sensitive teeth, braces, whitening, etc.).

Q12 Should I stop brushing my teeth for a few days if my gums are bleeding?

No you should not stop brushing your teeth just because your gums are bleeding.

Bleeding gums are very commonly caused by inflammation (often gingivitis) from plaque buildup. If you stop brushing, plaque builds up even more, which usually makes the bleeding and inflammation worse.

What you should do instead:

Keep brushing twice a day, but be very gentle
Use a soft-bristled toothbrush and light pressure (don't "scrub").

Brush along the gumline, where plaque collects, but carefully.

Floss daily (you may see some bleeding at first, but this often improves within about a week if inflammation is the cause).

Consider an antiseptic mouthwash if your gums are irritated (not a replacement for brushing).

When bleeding is more concerning:

If it lasts more than 7 10 days despite good oral care .

If gums are very swollen, painful, or receding .

If you notice loose teeth or persistent bad breath .

In those cases, it’s a good idea to see a dentist, because it could be progressing gum disease or another issue that needs treatment.

If you want, tell me how long this has been happening and whether it’s all over or in one spot I can help you narrow down what’s most likely going on.

Q13 Do I need to brush my teeth after every meal to keep my gums healthy?

No you don’t need to brush after every meal to keep your gums healthy, and doing so too often can even be counterproductive.

For most people, what actually protects gum health is consistent daily care, not constant brushing:

Brush twice a day (morning and before bed) with fluoride toothpaste .

Clean between teeth once a day (floss or interdental brushes) .

Regular dental checkups and cleanings .

Brushing after meals *can* be helpful in some situations, but it depends on what you ate:

If you had sugary or sticky foods, rinsing your mouth or brushing later can help .

If you had acidic foods/drinks (citrus, soda, coffee, tomato-based sauces), it’s better to wait ~30 minutes before brushing, because your enamel is temporarily softened and brushing too soon can wear it down .

A lot of gum problems (like gingivitis) are more about plaque along the gumline and between teeth, which is why flossing/interdental cleaning matters as much as brushing sometimes even more.

If you want a simple rule:

Brush twice daily .

Floss once daily .

Rinse with water after meals if you want a quick clean feeling .

Brush after meals only occasionally, not as a strict routine .

If you want, I can tailor a gum-health routine based on whether you have bleeding gums, sensitivity, or orthodontic work.

Q14 Even if I brush my teeth regularly, do I still need to get my gums checked?

Yes.

Brushing regularly is great for preventing plaque buildup, but it doesn't guarantee that your gums are healthy underneath the surface. Gum problems often develop quietly and can go unnoticed until they become more serious.

Dentists check for things you can't easily see or feel at home, such as early signs of gingivitis (gum inflammation) or the more advanced periodontal disease, where the bone and tissues supporting your teeth can be affected. These conditions can progress even in people who brush well, especially if flossing is inconsistent or if there are other risk factors like genetics, smoking, stress, or certain medical conditions.

During a gum check, a dentist or hygienist measures the depth of the pockets around your teeth and looks for bleeding, recession, or hidden plaque/tartar under the gumline things brushing alone can't fully address.

For most people, a gum check is part of a routine dental visit every 6 12 months. If you've ever had gum bleeding, bad breath that doesn't go away, or sensitivity along the gumline, it's even more important not to skip those checkups.

If you want, I can tell you the early warning signs that your gums might already be developing problems at home.

Q15 Is brushing my teeth once a day enough to keep my mouth healthy?

For most people, brushing once a day is not enough to keep your mouth fully healthy.

Dental professionals generally recommend:

Brushing at least twice a day (morning and before bed) .

Using fluoride toothpaste .

Cleaning for about 2 minutes each time .

Brushing once daily can still remove some plaque, but it leaves a lot of time for bacteria to build up. Overnight is especially important while you sleep, saliva flow decreases, which makes it easier for plaque and acids to damage teeth and gums.

The American Dental Association recommends brushing twice daily because it helps:

Prevent cavities .

Reduce gum disease risk .

Control bad breath .

Limit plaque buildup .

Also, brushing alone isn't the whole picture. Even with twice-daily brushing, good oral health usually also includes:

Flossing at least once a day (or interdental cleaning) .

Limiting sugary snacks/drinks .

Regular dental checkups .

If you're currently only brushing once a day, moving to twice daily (especially adding a bedtime brush) is one of the most effective improvements you can make.

Q16 Can flossing cause gum recession?

Flossing itself does not normally cause gum recession. In fact, proper flossing is generally protective for your gums because it removes plaque that can lead to inflammation and periodontal disease.

That said, there are a few situations where flossing can *seem* linked to recession or may contribute to gum irritation:

If you floss too aggressively snapping the floss into the gums or forcing it down this can cause mechanical trauma, leading to soreness, bleeding, and in rare cases minor gum damage over time. But even then, it's usually not true long-term recession on its own.

More commonly, what people notice is that when they start flossing regularly, their gums bleed or feel tender. That's usually because there was already gum inflammation (gingivitis) present. As the inflammation improves, the gums can look like they've "changed," but that's healing rather than recession being caused by flossing.

True gum recession is more often caused by things like:

Periodontal disease .

Long-term aggressive tooth brushing .

Genetic susceptibility / thin gum tissue .

Teeth grinding or misalignment .

Smoking .

If flossing seems to be "cutting" your gums or making them worse, it usually means technique needs adjustment gentle C-shape wrapping around each tooth works best.

If you want, I can show you the correct flossing technique step-by-step so you can avoid irritation.

Q17 Do electric toothbrushes damage the gums more than regular toothbrushes?

No electric toothbrushes do not inherently damage gums more than regular (manual) toothbrushes. In fact, when used correctly, they often reduce gum inflammation and gingivitis more effectively than manual brushing.

The key difference isn't the toothbrush type it's how it's used.

Electric toothbrushes (like those from Oral-B or Philips / Philips Sonicare) are designed to:

Maintain a consistent, optimal brushing motion and pressure .

Help people brush long enough (often with built-in timers) .

Reduce the need for aggressive scrubbing .

When gum damage *can* happen.

Gums get irritated or recede mainly due to:

Too much pressure (very common cause) .

Using a hard-bristled brush .

Brushing incorrectly (scrubbing horizontally or forcing the brush) .

Existing gum disease .

Electric brushes can actually *increase risk of irritation only if misused*, for example:

Pressing too hard and letting the brush “dig” into the gums .

Not letting the brush do the work (scrubbing like a manual brush) .

Many modern electric toothbrushes include pressure sensors that stop or warn you when you’re pressing too hard, which helps prevent gum damage.

Manual vs electric real comparison.

Electric toothbrush: more effective plaque removal, easier for most people, lower risk of technique errors .

Manual toothbrush: safe when used correctly, but more dependent on user technique .

Bottom line.

Electric toothbrushes are not more damaging to gums they are often safer and more effective, especially for people who tend to brush too hard or inconsistently.

If you want, I can tell you the ideal brushing technique for either type so you avoid gum recession entirely.

Q18 Would using mouthwash alone be enough without brushing my teeth?

No mouthwash alone isn’t enough to replace brushing.

Mouthwash can help reduce bacteria, freshen breath, and sometimes deliver fluoride, but it doesn’t remove the sticky layer of plaque that builds up on your teeth. That plaque is the main cause of cavities and gum disease, and it has to be physically disrupted.

Brushing does that mechanical work. When you brush (ideally twice a day with fluoride toothpaste), you’re:

scraping off plaque from tooth surfaces .

cleaning along the gumline where disease often starts .

helping fluoride stay on the teeth to strengthen enamel .

Mouthwash is best thought of as a supplement, not a substitute. Even a good antibacterial or fluoride rinse won’t reach or remove plaque effectively on its own.

If brushing is difficult sometimes, mouthwash is better than doing nothing but relying on it long-term will significantly increase your risk of cavities and gum problems.

If you want, tell me your situation (e.g., braces, sensitive gums, busy schedule), and I can suggest a minimal routine that still works well.

Q19 After I quit smoking, my gums started bleeding. Is that normal?

Yes this can happen after quitting smoking, and it's fairly common, but it shouldn't be ignored if it continues.

When you smoke, nicotine causes blood vessels in your gums to constrict (narrow). That reduces blood flow and can actually mask gum inflammation and bleeding. So even if you had early gum disease, you might not have noticed it while smoking.

After you quit, a few things change:

Blood flow returns to normal, so inflamed gums are more likely to bleed.

Your immune system starts functioning better, which can "reveal" existing issues like gum inflammation.

If plaque has built up over time, you may now notice signs of Gingivitis, which is the earliest stage of gum disease.

Some people also experience temporary changes in saliva and mouth sensitivity during nicotine withdrawal, which can make gums feel more reactive.

So the bleeding itself isn't unusual right after quitting, but it usually means there's underlying gum inflammation, not that quitting caused damage.

When it's still "normal" vs when to check it.

It's worth seeing a dentist if:

Bleeding lasts more than ~1 2 weeks after quitting .

Your gums are swollen, tender, or receding .

You have persistent bad breath or loose teeth .

Those can suggest progression toward Periodontitis, which is a more advanced gum infection that needs treatment.

What you can do now.

Brush gently twice a day (soft toothbrush) .

Clean between teeth daily (floss or interdental brushes) .

Use a mild antiseptic mouthwash if tolerated .

Book a dental cleaning if you haven't had one recently .

If you want, tell me how long it's been since you quit and how severe the bleeding is I can help you narrow down whether it sounds like temporary adjustment or something that needs faster dental care.

Q20 Can natural products effectively keep my gums healthy?

Yes but with an important caveat: "natural" products can support gum health, but they don't replace the fundamentals of plaque control (brushing, flossing, and professional cleanings). Gum disease is mainly caused by bacterial plaque, so anything that doesn't reduce or disrupt that biofilm will have limited effect.

Some natural products do have *supportive* benefits:

Green tea contains compounds (catechins) that may reduce inflammation and bacterial growth in the gums. It's associated in some studies with lower rates of gum bleeding and periodontal disease progression.

Saltwater rinses are simple but genuinely useful. Warm saline can reduce swelling, soothe irritated gums, and help control surface bacteria after brushing.

Aloe vera gel (used in some mouth rinses or gels) may help reduce gum inflammation in mild gingivitis, though results are modest.

Oil pulling (usually coconut oil) has mixed evidence. It may slightly reduce bacteria and plaque, but it's not as effective as fluoride toothpaste and proper brushing.

Herbal extracts like neem or tea tree oil show antibacterial properties in lab studies, but real-world dental outcomes are less consistent, and essential oils can irritate tissues if misused or too concentrated.

What matters most is that none of these can reliably remove hardened plaque (tartar) or treat established periodontal disease. Once gum disease progresses, professional cleaning is necessary.

If your goal is genuinely healthy gums, the strongest "natural effective" combo looks like:

Brushing twice daily with fluoride toothpaste .

Daily flossing or interdental cleaning .

Regular dental checkups .

Optional: green tea, saltwater rinses, or sugar-free chewing gum as supportive habits .

If you want, tell me what you're currently using I can help you figure out whether your routine is actually protecting your gums or just "feeling" healthy.

Q21 Can getting a dental scaling damage my teeth?

In normal circumstances, getting a professional dental scaling does not damage your teeth. It is a routine preventive treatment designed to remove hardened plaque (tartar/calculus) and bacteria that your toothbrush can't reach.

What actually happens during scaling.

Dentists or hygienists use manual tools or ultrasonic devices to remove:

hardened plaque (dental calculus) .

bacteria along the gumline and between teeth .

This helps prevent or treat conditions like gingivitis and more advanced gum disease like periodontitis.

Why people think it "damages" teeth

After scaling, some people notice:

temporary tooth sensitivity (especially to cold) .

mild gum soreness or bleeding .

a feeling that teeth are “looser” (actually because swelling has gone down and tartar was supporting them) .

These effects are usually short-term and not signs of damage.

Can scaling actually harm teeth?

When done properly by a trained professional, scaling:

does not remove healthy enamel .

does not weaken teeth .

does not create gaps between teeth .

However, problems can occur if:

it’s done too aggressively or improperly (rare in licensed clinics) .

you already have severe gum disease and underlying bone loss (teeth may feel more sensitive afterward, but scaling is still necessary) .

Bottom line

Scaling is generally protective, not harmful. The benefits (preventing gum disease and tooth loss) far outweigh the temporary discomfort some people feel afterward.

If you want, I can explain what a “deep cleaning” (scaling and root planing) is and how it differs from regular scaling.

Q22 Can dental scaling cause gaps between my teeth?

Short answer: no dental scaling doesn’t create real gaps between teeth, but it can sometimes *make existing spaces more noticeable*.

Here’s what’s usually going on:

After a professional cleaning (scaling), plaque and tartar are removed from between and around the teeth. If there was buildup “filling in” tight spaces, removing it can reveal the true shape of the gaps that were already there. So it may *look like new spaces appeared*, but they didn’t actually form because of the cleaning.

A few other common reasons people notice gaps afterward:

Gum swelling goes down: If your gums were inflamed before cleaning, they may have been puffy and partially filling spaces. Once inflammation reduces, teeth can look slightly more separated.

Tartar was acting like a bridge: Hardened calculus can sit between teeth and mask small spaces. Removing it uncovers them.

Existing gum recession or bone loss: If there was underlying periodontal disease, scaling is part of treatment, and the real tooth support condition becomes visible afterward.

What scaling *doesn’t* do:

It does not push teeth apart.

It does not shrink enamel or physically move teeth.

If you're noticing a new or widening gap that's bothering you, it's worth a follow-up with your dentist or periodontist especially if there was gum disease involved. They can check whether it's just a visual change after cleaning or something that needs further treatment.

If you want, describe what you're seeing and I can help you figure out what's most likely happening.

Q23 Do all gum diseases require the same treatment?

No, gum diseases do not all require the same treatment. "Gum disease" is an umbrella term, and the treatment depends on which stage and type you have.

The main conditions are:

Gingivitis (early stage)
This is inflammation of the gums caused by plaque buildup. The key feature is that it's reversible.
Treatment is usually simple:

Professional cleaning (removal of plaque and tartar) .

Improved daily brushing and flossing .

Sometimes antiseptic mouth rinses .

If treated early, gums can return to normal.

Periodontitis (advanced stage).
This is when inflammation has progressed and starts damaging the bone and tissues that support teeth. It is not fully reversible, but it can be controlled.
Treatment may include:

Deep cleaning below the gum line (scaling and root planing) .

More frequent dental cleanings .

Antibiotics in some cases .

Surgical procedures if there are deep pockets or bone loss .

Other gum conditions.
There are also less common issues like gum abscesses or gum recession from grinding or brushing too hard, and these each have different treatments (infection control, gum grafting, bite adjustment, etc.).

Bottom line:
The treatment depends on severity and cause. Early gum disease often needs only improved hygiene and cleaning, while advanced disease may require deep cleaning or surgery.

If you want, describe your symptoms and I can help you figure out which category it might fit into.

Q24 Is gum treatment painful?

Most gum treatments are not very painful, because dentists use local anesthesia to numb the area. What you feel depends on the type of treatment and how severe the gum disease is.

Here’s a general idea:

Deep cleaning (scaling and root planing):
Usually causes mild discomfort or pressure during the procedure. Your gums may feel sore or sensitive for a few days afterward.

Laser gum treatment:
Often less painful than traditional surgery and may have faster healing.

Gum surgery or grafting:
More invasive, so you can expect soreness, swelling, and tenderness for several days, but pain medication helps manage it.

Common after-effects include:

mild bleeding .

swollen gums .

tooth sensitivity .

tenderness when eating .

Most people say the discomfort is manageable and much less severe than they expected.

If you’re nervous, tell your dentist or periodontist they can offer:

stronger numbing .

sedation options .

anxiety-reducing techniques .

Untreated gum disease can become much more painful over time, so treatment is usually worth it.

Q25 Can gum surgery cause tooth loss?

Yes gum surgery (periodontal surgery) can *rarely* be associated with tooth loss, but it’s important to understand the context: in most cases, the surgery is actually done to prevent tooth loss, not cause it.

When tooth loss can happen.

Tooth loss after gum surgery is uncommon, but it may occur if:

The tooth was already very loose due to advanced Periodontitis (severe gum disease). In these cases, the tooth might have been close to failing anyway.

Severe bone loss is present, and surgery exposes that instability.

Infection or poor healing develops after the procedure (rare with proper care).

The tooth becomes non-restorable during treatment planning (sometimes revealed only during surgery).

More commonly, what actually happens

Most gum surgeries such as flap surgery, gum grafting, or bone reshaping are designed to:

Remove infected tissue .

Reduce gum pockets .

Stabilize teeth .

Improve long-term tooth survival .

So if a tooth is lost afterward, it’s usually because the underlying disease was already very advanced, not because the surgery “caused” it directly.

Key takeaway.

Gum surgery is generally a tooth-saving treatment, but in severe gum disease cases, it may expose that a tooth cannot be saved.

If you want, tell me what kind of gum surgery you’re considering or had, and I can explain the specific risks more precisely.

Q26 Can gum treatment make my teeth feel loose?

Yes gum treatment *can* make teeth feel temporarily loose in some situations, and it doesn’t always mean something is going wrong.

The most common reason is that the treatment is removing hardened plaque (tartar) and inflammation around the gums. In conditions like Periodontitis, tartar buildup can actually act like a “support brace” around teeth. Once it’s cleaned away, the underlying reality shows up: the tooth may already have some bone or ligament loss. That can make teeth feel more mobile afterward, even though the treatment itself didn’t cause the damage.

Another common scenario is after deep cleaning (scaling and root planing). When swollen gums shrink back down as inflammation improves, the teeth can feel more exposed and slightly different in “fit” or stability. This sensation is usually temporary.

In some cases:

Mild looseness is expected and may improve as gums heal (weeks to a few months) .

Moderate looseness may reflect existing bone loss that treatment is trying to stabilize.

Increasing or painful mobility is *not* typical and should be checked promptly .

The key idea: gum treatment doesn’t usually *create* looseness it often reveals or temporarily highlights the underlying support level of the teeth.

If you tell me what treatment you had (cleaning, deep cleaning, surgery, etc.) and how long ago, I can give a more specific expectation for your situation.

Q27 Does tartar removal cause more tartar buildup?

No tartar removal (usually called *dental scaling*) does not cause more tartar to build up.

Tartar (calculus) forms when plaque hardens on teeth over time. Professional cleaning removes that hardened layer, but it doesn’t change how your body produces plaque or how quickly tartar forms afterward.

What *can* make it seem like tartar comes back faster after cleaning:

You become more aware of it after seeing “clean” teeth .

Plaque regrowth resumes immediately (this is normal for everyone) .

If oral hygiene doesn’t improve, tartar will re-form at the same rate as before .

One important nuance:

A properly done cleaning smooths the tooth surface, which actually makes it *harder* for plaque to stick initially.

In rare cases, if cleaning is done aggressively or with poor technique, teeth can feel slightly rough or sensitive temporarily but this still doesn’t biologically increase tartar formation.

Bottom line: tartar removal doesn’t “trigger” more tartar. Tartar comes back based on plaque control (brushing, flossing, diet, saliva), not because it was cleaned off.

If you want, I can explain how fast tartar typically reforms or how to slow it down the most effectively.

Q28 Are antibiotics enough to treat gum disease?

Not usually.

“Gum disease” (especially the more serious form, periodontitis) is mainly a biofilm plaque problem, meaning bacteria live in hardened deposits (tartar/calculus) below the gumline. In that setting, antibiotics alone are not enough because they can’t fully penetrate or remove the physical buildup causing the infection.

Here’s how it breaks down:

Mild gum disease (gingivitis).

This is early-stage inflammation of the gums.

It is usually reversible with proper brushing, flossing, and professional cleaning.

Antibiotics are rarely needed.

Moderate to severe gum disease (periodontitis).

This involves deeper infection, gum pocket formation, and sometimes bone loss.

Main treatment is mechanical cleaning, such as:

Scaling and root planing (deep cleaning under the gums) .

Ongoing periodontal maintenance .

Antibiotics (like Amoxicillin or Metronidazole) may be used as an add-on, but not as the primary treatment.

Why antibiotics alone don’t work well.

They don’t remove plaque or tartar.

Bacteria quickly recolonize if the source remains.

Gum pockets physically protect bacteria from medication.

Bottom line:
Antibiotics can help in certain cases, especially acute infections or as support therapy, but successful treatment of gum disease almost always requires professional dental cleaning improved daily oral hygiene.

If you want, I can explain what stage your symptoms might suggest or what a dentist typically does step-by-step.

Q29 After gum disease treatment, will I ever need treatment again?

Not necessarily but it depends on the type and severity of the gum disease and how well it's maintained afterward.

If you've had treatment for gum disease (often gingivitis or more advanced periodontitis), the goal is usually to stop the infection and control it long-term, not "cure it forever" in one step.

Here's the realistic picture:

Gum disease can come back.

Even after successful treatment, gum disease can recur if plaque and bacteria build up again. That's because the bacteria that cause it are always present in the mouth.

Many people need ongoing maintenance.

Most patients who've had periodontitis are placed on a recall/maintenance schedule (often every 3 6 months) for:

Professional deep cleaning around the gums .

Monitoring bone and pocket depth .

Early treatment if it starts returning .

Some people stay stable for years.

If you:

Brush and floss consistently .

Stop smoking .

Control diabetes (if relevant) .

Go to regular dental cleanings .

you may never need another intensive treatment like scaling and root planing or surgery again.

But "once and done" is uncommon for advanced disease.

With periodontitis, especially moderate to severe cases, it's usually a lifelong condition to manage, not permanently eliminated.

If you want, tell me what treatment you had (deep cleaning, surgery, antibiotics, etc.), and I can give a more specific expectation for your situation.

Q30 Is surgery always necessary for gum treatment?

No surgery is not always necessary for gum (periodontal) treatment.

Whether you need surgery depends mainly on how advanced the gum disease is and how well you respond to non-surgical care.

When surgery is NOT needed.

Most people are treated successfully without surgery, especially in early or moderate gum disease:

Gingivitis (early gum inflammation): usually reversible with good brushing, flossing, and professional cleaning .

Mild to moderate Periodontitis: often treated with:

Scaling and root planing (deep cleaning under the gums) .

Improved home oral hygiene .

Antibacterial mouth rinses or sometimes antibiotics .

These approaches can reduce infection and allow gums to heal or stabilize.

When surgery may be needed.

Surgery is usually considered only if non-surgical treatment is not enough, such as:

Deep periodontal pockets that don’t improve after deep cleaning .

Significant bone loss around teeth .

Persistent infection or gum inflammation .

Loose teeth that need stabilization or regenerative treatment .

Common procedures include flap surgery (to clean deep areas), bone grafts, or gum grafts.

Key idea.

Dentists usually start with the least invasive treatment first. Surgery is more of a backup option, not the default.

If you want, you can describe your symptoms (bleeding, bad breath, gum recession, etc.), and I can help you estimate how severe it might be and what treatments are typically used.
