# Supplementary material for: Comparative Evaluation of Large Language Models in Responding to Common Misconceptions Regarding Periodontal Disease and Oral Hygiene
Source: Diagnostics (Basel). 2026 Sep 28;16(19):3158. doi: 10.3390/diagnostics16193158 (PMC13664004; doi:10.3390/diagnostics16193158)
Supplement: Supplementary file 1 [file diagnostics-16-03158-s001.zip › Supplementary File S5 LLMs response stylistic elements removed.zip!/2. LLMs response/Monica AI.pdf]

Q1 Do gum diseases only affect elderly people?

No, gum diseases do not only affect elderly people. While the risk of developing gum disease, such as gingivitis and periodontitis, increases with age, they can affect individuals of all ages, including children and young adults. Factors that contribute to gum disease include:

Poor Oral Hygiene: Inadequate brushing and flossing can lead to plaque buildup.

Smoking and Tobacco Use: These habits significantly increase the risk of gum disease.

Genetics: Some people may be more genetically predisposed to gum issues.

Medical Conditions: Conditions like diabetes, heart disease, and certain autoimmune disorders can increase susceptibility.

Hormonal Changes: Changes during puberty, menstruation, pregnancy, or menopause can affect gum health.

It's essential for individuals of all ages to maintain good oral hygiene and have regular dental check-ups to prevent gum disease.

Q2 Is it normal if my gums bleed when I brush my teeth?

Bleeding gums when brushing your teeth can be a sign of several issues, and it's not considered normal. Here are some common reasons for this condition:

Possible Causes.

Gingivitis: This is an early stage of gum disease caused by plaque buildup. It often leads to swollen and bleeding gums.

Poor Oral Hygiene: Inadequate brushing or flossing can lead to plaque accumulation, resulting in gum irritation and bleeding.

Vitamin Deficiencies: Lack of certain vitamins, especially Vitamin C, can affect gum health.

Hormonal Changes: Changes due to menstruation, pregnancy, or menopause can make gums more sensitive and prone to bleeding.

Medical Conditions: Conditions like diabetes or blood disorders can also contribute to gum bleeding.

Medications: Some medications can have side effects that affect gum health.

Recommendations.

Improve Oral Hygiene: Ensure you are brushing twice a day and flossing daily.

Visit a Dentist: It's important to consult a dental professional for a proper diagnosis and treatment.

Stay Hydrated: Drink plenty of water to help maintain oral health.

Balanced Diet: Include foods rich in vitamins and minerals that support gum health.

If the bleeding persists, it's best to seek advice from a healthcare professional.

Q3 Can I get gum disease even if I brush my teeth regularly?

Yes, it is possible to develop gum disease even if you brush your teeth regularly. Here are some factors that can contribute to gum disease:

Factors Contributing to Gum Disease.

Improper Technique: If you are not brushing effectively (e.g., not reaching all areas of your mouth), plaque can build up.

Frequency of Brushing: Brushing twice a day is generally recommended, but if you miss brushing after meals or snacks, it can lead to plaque accumulation.

Flossing: Not flossing regularly can leave food particles and plaque between teeth, which can lead to gum disease.

Diet: A diet high in sugars and starches can contribute to plaque formation.

Tobacco Use: Smoking or using other tobacco products can increase the risk of gum disease.

Hormonal Changes: Changes due to pregnancy, menstruation, or menopause can make gums more sensitive and prone to disease.

Medical Conditions: Certain health conditions, such as diabetes, can increase the risk of gum disease.

Medications: Some medications can reduce saliva flow, which helps protect against gum disease.

Genetics: Some individuals may be more genetically predisposed to gum disease.

Prevention Tips.

Brush Properly: Use the correct technique and a fluoride toothpaste.

Floss Daily: Ensure to clean between your teeth.

Regular Dental Check-ups: Visit your dentist regularly for cleanings and check-ups.

Healthy Diet: Maintain a balanced diet and limit sugary snacks.

If you have concerns about gum disease, it's best to consult with a dental professional for personalized advice and treatment options.

Q4 Can I have gum disease even if my gums don't hurt?

Yes, it is possible to have gum disease even if your gums don't hurt. Many people with gum disease may not experience pain or noticeable symptoms in the early stages. Here are some key points to consider:

Signs of Gum Disease.

Bleeding Gums: Especially during brushing or flossing.

Swollen or Red Gums: Healthy gums should be pink and firm.

Bad Breath: Persistent bad breath can be a sign of gum issues.

Loose Teeth: Advanced gum disease can lead to tooth mobility.

Receding Gums: Gums may pull away from teeth, making them look longer.

Types of Gum Disease.

Gingivitis: The earliest stage, often reversible with good oral hygiene.

Periodontitis: More severe, can lead to tooth loss if untreated.

Importance of Dental Check-ups.

Regular dental check-ups are crucial for early detection of gum disease, even in the absence of pain. A dentist can identify issues that may not be apparent to you.

If you have concerns about your gum health, it's advisable to consult a dental professional for a thorough examination.

Q5 Can gum disease get better without treatment?

Gum disease, also known as periodontal disease, can vary in severity. Here's a breakdown of how it may improve or worsen without treatment:

Mild Gum Disease (Gingivitis).

Can Improve: Gingivitis is the earliest stage of gum disease and is often reversible with good oral hygiene practices, such as regular brushing, flossing, and professional cleanings.

Symptoms: Red, swollen gums that may bleed during brushing.

Moderate to Severe Gum Disease (Periodontitis).

Less Likely to Improve: In more advanced stages, such as periodontitis, the condition typically does not improve without professional treatment.

Consequences: Without treatment, periodontitis can lead to gum recession, tooth mobility, and eventual tooth loss.

Symptoms: Persistent bad breath, deeper pockets between teeth and gums, and loss of bone around teeth.

Conclusion.

While mild cases may improve with better oral care, moderate to severe gum disease usually requires professional treatment to halt its progression and restore gum health. If you suspect gum disease, it's important to consult a dental professional for an appropriate evaluation and treatment plan.

Q6 Are gum diseases rare?

Gum diseases, also known as periodontal diseases, are not rare. They are quite common and can affect a significant portion of the population. Here are some key points:

Prevalence.

Mild Forms: Gingivitis, the mildest form of gum disease, affects a large number of adults at some point in their lives.

Severe Forms: Periodontitis, a more severe form, affects around 10-15% of adults globally.

Risk Factors.

Several factors can increase the risk of developing gum disease, including:

Poor oral hygiene.

Tobacco use.

Diabetes.

Certain medications.

Hormonal changes (e.g., pregnancy, menopause).

Genetic predisposition.

Importance of Oral Health.

Maintaining good oral hygiene practices, such as regular brushing, flossing, and dental check-ups, can help prevent gum disease. Early detection and treatment are crucial for managing the condition effectively.

If you have specific concerns about gum disease, it's best to consult with a dental professional.

Q7 Is it normal to lose teeth because of gum disease as you get older?

Yes, it is common for older adults to experience tooth loss due to gum disease, also known as periodontal disease. Here are some key points to consider:

Understanding Gum Disease.

Stages: Gum disease typically progresses from gingivitis (inflammation of the gums) to periodontitis (more severe inflammation that can affect the bone supporting the teeth).

Causes: Factors such as poor oral hygiene, smoking, diabetes, and certain medications can increase the risk of developing gum disease.

Impact of Aging.

Increased Risk: As people age, they may have a higher likelihood of experiencing gum disease due to cumulative effects of plaque buildup and changes in oral health.

Bone Density: Aging can also lead to a decrease in bone density, which can affect the jawbone that supports teeth.

Prevention and Management.

Regular Dental Check-ups: Routine visits to the dentist for cleanings and check-ups can help detect gum disease early.

Good Oral Hygiene: Brushing twice a day, flossing, and using mouthwash can help maintain gum health.

Healthy Lifestyle: A balanced diet, quitting smoking, and managing health conditions like diabetes can also reduce the risk.

If you or someone you know is concerned about gum disease and tooth loss, it’s important to consult a dental professional for personalized advice and treatment options.

Q8 If gum disease runs in my family, will I definitely get it too?

While having a family history of gum disease can increase your risk, it does not guarantee that you will develop it. Several factors contribute to the development of gum disease, including:

Genetic Factors.

Family History: Genetics can play a role in your susceptibility to gum disease.

Immune Response: Some individuals may have a genetic predisposition that affects their immune response to bacteria in the mouth.

Lifestyle Factors.

Oral Hygiene: Good dental care practices can significantly reduce your risk.

Diet: A balanced diet can strengthen your immune system and oral health.

Smoking: Tobacco use is a major risk factor for gum disease.

Other Health Conditions.

Diabetes: Conditions like diabetes can increase your risk of gum disease.

Hormonal Changes: Hormonal fluctuations can also affect gum health.

Prevention Tips.

Regular Dental Check-ups: Regular visits to the dentist can help detect early signs of gum disease.

Proper Oral Hygiene: Brushing twice a day and flossing daily are essential for maintaining gum health.

In conclusion, while family history is a factor, maintaining good oral hygiene and a healthy lifestyle can help mitigate your risk. If you're concerned, consider discussing your family history with a dental professional for personalized advice.

Q9 Can stress make me more likely to get gum disease?

Yes, stress can increase your likelihood of developing gum disease. Here’s how stress affects oral health:

How Stress Contributes to Gum Disease.

Hormonal Changes: Stress triggers the release of hormones like cortisol, which can lead to inflammation in the body, including the gums.

Immune System Suppression: Chronic stress can weaken your immune system, making it harder for your body to fight off infections, including those that affect the gums.

Neglecting Oral Hygiene: When stressed, individuals may neglect their oral hygiene routines, leading to plaque buildup and increased risk of gum disease.

Unhealthy Habits: Stress can lead to unhealthy behaviors such as smoking, poor diet, or teeth grinding (bruxism), all of which can contribute to gum problems.

Inflammation: Stress can exacerbate inflammatory conditions, including periodontal disease, making existing gum issues worse.

Tips to Manage Stress for Better Oral Health.

Practice Relaxation Techniques: Engage in activities like meditation, yoga, or deep breathing exercises.

Maintain Oral Hygiene: Brush and floss regularly, even when feeling stressed.

Healthy Diet: Eat a balanced diet rich in vitamins and minerals that support gum health.

Regular Dental Check-ups: Visit your dentist regularly for cleanings and check-ups.

If you're concerned about gum disease or the effects of stress on your oral health, it's a good idea to consult with a dental professional.

Q10 Do gum diseases only affect the mouth and teeth?

Gum diseases, primarily known as periodontal diseases, primarily affect the mouth and teeth, but their impact can extend beyond oral health. Here are some key points to consider:

Local Effects.

Gums and Teeth: The primary area affected is the gums, which can become inflamed, bleed, or recede. This can lead to tooth mobility and loss if untreated.

Oral Health: Gum disease can result in bad breath (halitosis) and contribute to other dental problems.

Systemic Effects.

Heart Disease: Studies suggest a link between periodontal disease and heart conditions, potentially increasing the risk of heart attacks and strokes.

Diabetes: There is a bidirectional relationship; gum disease can worsen diabetes, and poorly controlled diabetes can exacerbate gum disease.

Respiratory Issues: Bacteria from gum disease can be aspirated into the lungs, potentially leading to respiratory infections.

Pregnancy Complications: Pregnant women with periodontal disease may face a higher risk of premature birth and low birth weight.

Conclusion.

While gum diseases primarily affect the mouth and teeth, their implications can significantly influence overall health. Maintaining good oral hygiene and regular dental check-ups is essential for preventing gum disease and its potential systemic effects.

Q11 Will a hard-bristled toothbrush clean my teeth better?

Using a hard-bristled toothbrush can be effective for cleaning teeth, but it also comes with potential drawbacks. Here are some points to consider:

Advantages of Hard-Bristled Toothbrushes.

Effective Plaque Removal: They can remove plaque and food particles more effectively due to their stiffness.

Stain Removal: Hard bristles may help in removing surface stains on teeth.

Disadvantages of Hard-Bristled Toothbrushes.

Gum Damage: They can cause irritation or damage to your gums, leading to receding gums or sensitivity.

Enamel Wear: Over time, hard bristles can wear down tooth enamel, increasing the risk of cavities and sensitivity.

Less Comfortable: Many people find hard-bristled brushes uncomfortable to use.

Recommendations.

Soft or Medium-Bristled Brushes: Dentists often recommend using a soft or medium-bristled toothbrush for most people, as they are gentle on gums and enamel while still effective at cleaning.

Technique: Regardless of bristle hardness, proper brushing technique is crucial. Use gentle, circular motions rather than aggressive scrubbing.

Conclusion.

While a hard-bristled toothbrush may clean your teeth well, it’s essential to weigh the benefits against the risks. A softer brush with proper technique is generally recommended for maintaining oral health. Always consult with your dentist for personalized advice.

Q12 Should I stop brushing my teeth for a few days if my gums are bleeding?

If your gums are bleeding, it's important to maintain your oral hygiene rather than stop brushing your teeth. Here are some steps you can take:

Recommendations:

Continue Brushing: Brush your teeth gently with a soft-bristled toothbrush. This helps to remove plaque that can contribute to gum disease.

Floss Carefully: Flossing is crucial, but be gentle to avoid further irritation.

Use Antiseptic Mouthwash: An antiseptic mouthwash can help reduce bacteria in your mouth.

Stay Hydrated: Drink plenty of water to help keep your mouth moist and wash away food particles.

Monitor Your Diet: Avoid sugary and acidic foods that can irritate your gums.

Consult a Dentist: If the bleeding persists for more than a few days, or if you have other symptoms, it's important to see a dentist for a professional evaluation.

When to Seek Help:

Persistent bleeding.

Swelling or pain in the gums.

Bad breath that doesn’t go away.

Taking care of your gums is essential for overall oral health, so don't neglect your brushing routine!

Q13 Do I need to brush my teeth after every meal to keep my gums healthy?

Brushing your teeth after every meal can be beneficial for maintaining gum health, but it's not always necessary. Here are some key points to consider:

Benefits of Brushing After Meals.

Removes Food Particles: Brushing helps eliminate food debris that can contribute to plaque buildup.

Prevents Plaque Formation: Regular brushing reduces the risk of plaque, which can lead to gum disease.

Freshens Breath: It helps keep your breath fresh by removing odor-causing bacteria.

Recommendations.

At Least Twice Daily: Most dental professionals recommend brushing at least twice a day once in the morning and once before bed.

Wait After Eating: If you've consumed acidic foods (like citrus), wait about 30 minutes before brushing to avoid damaging enamel.

Flossing: Don’t forget to floss daily, as it helps clean between teeth where a toothbrush can't reach.

Conclusion.

While brushing after every meal can be helpful, focus on maintaining a consistent oral hygiene routine. If you have specific concerns about your gum health, consider consulting with a dentist for personalized advice.

Q14 Even if I brush my teeth regularly, do I still need to get my gums checked?

Yes, even if you brush your teeth regularly, it's important to get your gums checked by a dentist. Here are a few reasons why:

Importance of Gum Checks.

Early Detection of Gum Disease:

Gum disease can develop without noticeable symptoms. Regular check-ups can help identify early signs.

Professional Cleaning:

Dentists can remove plaque and tartar that regular brushing might miss, helping to prevent gum disease.

Assessment of Gum Health:

Dentists can measure the depth of gum pockets and assess overall gum health, which is crucial for preventing issues.

Personalized Care:

A dentist can provide tailored advice based on your oral health and habits.

Overall Health Connection:

Gum health is linked to overall health. Conditions like diabetes and heart disease can be influenced by gum health.

Recommendations.

Schedule regular dental check-ups (typically every 6 months).

Maintain good oral hygiene practices at home, including brushing and flossing.

Taking these steps will help ensure your gums remain healthy!

Q15 Is brushing my teeth once a day enough to keep my mouth healthy?

Brushıng your teeth once a day ıs better than not brusıing at ali, but ıt may not be suttıcıent tor optımaı oral health. Here are some key points to consider:

Recommendations tor Oral Health.

Frequency of Brushi ng:

Twice a Day: The American Oental Association (ADA) recommends brushing your teeth at least twice a day-once in the morning and once betore bed.

Technique:

Use proper brushing techniques. ensuring you brush tor at least two minutes each time.

Flossing:

Floss daily to remove plaque and tood particles between teeth that a toothbrush can't reach.

Regular Dental Check-ups:

Visit your dentist regularty (typically every six months) tor professional cleanings and ched-ups.

Diet:

Maintain a balanced diet and limit sugary snacks and drinks, which can contribute to tooth decay.

Mouthwash:

Consider using an antibacterial mouthwash to help reduce plaque and gingivitis.

Conclusion

For the best oral health, aim to brush twice a day, floss daily, and maintain regular dental visits. This comprehensive approach will help keep your mouth healthy.

Q16 Can flossing cause gum recession?

Flossing is generally recommended as an important part of oral hygiene, but improper technique can contribute to gum recession. Here are some key points to consider:

Causes of Gum Recession Related to Flossing.

Aggressive Technique:

Flossing too aggressively can damage the gum tissue, leading to recession.

It’s important to be gentle and avoid snapping the floss against the gums.

Incorrect Flossing Method:

Using a sawing motion or not following the natural curve of the tooth can irritate the gums.

Proper technique involves sliding the floss gently between the teeth and curving it around the base of each tooth.

Frequency:

While regular flossing is beneficial, overdoing it or flossing too harshly can lead to irritation and potential recession.

Recommendations for Healthy Flossing.

Use Gentle Pressure: Be gentle when inserting and moving the floss.

Proper Technique: Follow a proper method wrap the floss around your fingers and use a C-shape around each tooth.

Consult a Dentist: If you're experiencing gum recession or discomfort, consult with a dental professional for personalized advice and technique correction.

In summary, while flossing is essential for gum health, improper techniques can contribute to gum recession. It's crucial to practice good flossing habits to avoid potential issues.

Q17 Do electric toothbrushes damage the gums more than regular toothbrushes?

Electric toothbrushes do not inherently damage gums more than regular toothbrushes; however, the risk of gum damage can depend on several factors:

Factors to Consider.

Technique:

Electric Toothbrush: If used correctly, electric toothbrushes can effectively clean teeth with less effort. However, applying too much pressure can lead to gum irritation.

Manual Toothbrush: The effectiveness depends heavily on the user's brushing technique. Aggressive brushing can also cause gum damage.

Brushing Pressure:

Many electric toothbrushes have pressure sensors that alert users if they are brushing too hard, which can help prevent gum damage.

Manual toothbrush users may not realize they are brushing too hard unless they are mindful.

Brush Head Design:

Electric toothbrushes often come with softer bristle options that can be gentler on gums.

Manual toothbrushes vary widely in bristle firmness, so choosing the right one is essential.

Frequency and Duration:

Over-brushing, regardless of the type of toothbrush, can lead to gum recession and damage. It’s important to brush for the recommended two minutes twice a day.

User Compliance:

Some studies suggest that people may brush longer and more thoroughly with electric toothbrushes, potentially improving oral health.

Conclusion.

Both electric and manual toothbrushes can be safe and effective when used properly. It's crucial to follow good brushing techniques, choose the right brush head, and not apply excessive pressure to minimize the risk of gum damage. If you have concerns about your gum health, consulting with a dental professional is advisable.

Q18 Would using mouthwash alone be enough without brushing my teeth?

Using mouthwash alone is not sufficient for maintaining optimal oral hygiene. Here are some key points to consider:

Why Mouthwash is Not Enough.

Plaque Removal:

Brushing your teeth helps remove plaque and food particles. Mouthwash can help kill bacteria but does not physically remove plaque.

Fluoride Benefits:

Many toothpaste brands contain fluoride, which helps strengthen tooth enamel and prevent cavities. Mouthwash may or may not contain fluoride, and it's often not as effective in delivering it to the teeth.

Gum Health:

Brushing helps maintain gum health by removing debris and preventing gum disease. Mouthwash alone cannot provide the same benefits.

Fresh Breath:

While mouthwash can temporarily freshen breath, it does not address the underlying causes of bad breath, such as plaque buildup or gum disease.

Recommended Oral Hygiene Routine.

Brush Teeth: At least twice a day for two minutes using fluoride toothpaste.

Floss Daily: To remove food particles and plaque from between the teeth.

Use Mouthwash: As an adjunct to brushing and flossing, especially one that contains antibacterial properties or fluoride.

In summary, while mouthwash can be a helpful addition to your oral care routine, it should not replace brushing and flossing.

Q19 After I quit smoking, my gums started bleeding. Is that normal?

It's not uncommon for gums to bleed after quitting smoking, as your body goes through various changes during the recovery process. Here are some points to consider:

Why It Happens:

Improved Blood Flow: Quitting smoking improves circulation and blood flow to the gums, which may lead to temporary bleeding as they heal.

Gum Health: Smoking can mask gum disease symptoms. Once you quit, underlying issues may become more apparent, leading to bleeding.

Oral Hygiene Changes: Changes in your oral hygiene routine or increased sensitivity after quitting can also contribute to bleeding gums.

What to Do:

Maintain Oral Hygiene: Brush and floss regularly to keep your gums healthy.

Stay Hydrated: Drink plenty of water to help maintain oral moisture.

Visit a Dentist: If the bleeding persists or worsens, it's important to consult a dentist to rule out any serious issues.

When to Seek Help:

If bleeding continues for more than a couple of weeks.

If you experience pain, swelling, or other unusual symptoms.

It's always best to consult with a healthcare professional for personalized advice.

Q20 Can natural products effectively keep my gums healthy?

Yes, natural products can be effective in maintaining gum health. Here are some options that may help:

Essential Oils.

Tea Tree Oil: Known for its antibacterial properties, it can help reduce plaque and gingivitis.

Peppermint Oil: Provides a refreshing taste and may help reduce bacteria in the mouth.

Herbal Remedies.

Aloe Vera: Has anti-inflammatory properties and can soothe gum irritation.

Green Tea: Contains antioxidants that may help reduce inflammation and support gum health.

Natural Mouthwashes.

Salt Water Rinse: A simple solution that can help reduce inflammation and promote healing.

Apple Cider Vinegar: Diluted with water, it may help kill bacteria and freshen breath.

Dietary Considerations.

Vitamin C: Found in citrus fruits, it helps strengthen gums and prevent gum disease.

Omega-3 Fatty Acids: Found in fish and flaxseeds, they have anti-inflammatory effects.

Oral Hygiene Practices.

Oil Pulling: Swishing coconut oil in your mouth may help reduce bacteria and improve gum health.

Proper Brushing and Flossing: Using natural toothpaste and regular flossing are essential for maintaining gum health.

Conclusion.

While natural products can support gum health, they should complement regular dental care practices. It's always best to consult with a dental professional for personalized advice and treatment options.

Q21 Can getting a dental scaling damage my teeth?

Dental scaling is a common procedure performed by dentists or dental hygienists to remove plaque and tartar from the surface of the teeth and below the gum line. Here are some key points regarding its safety and potential impact on your teeth:

Benefits of Dental Scaling.

Prevention of Gum Disease: Regular scaling helps prevent gingivitis and periodontitis by removing harmful bacteria.

Improved Oral Hygiene: It enhances overall oral hygiene and can lead to fresher breath.

Detection of Issues: Scaling can help identify other dental problems early.

Potential Risks.

Enamel Damage: If not performed correctly, scaling can potentially damage the enamel, but this is rare with professional care.

Sensitivity: Some patients experience temporary tooth sensitivity after scaling, but this usually resolves quickly.

Gum Irritation: There may be some irritation or bleeding of the gums, especially if there is existing gum disease.

Conclusion.

When performed by a qualified dental professional, dental scaling is generally safe and beneficial for your oral health. If you have concerns about your specific situation, it's best to discuss them with your dentist. Regular dental visits and cleanings are essential for maintaining healthy teeth and gums.

Q22 Can dental scaling cause gaps between my teeth?

Dental scaling is a common procedure performed to remove plaque and tartar from the teeth and gums. While it is generally safe and beneficial for oral health, some patients may notice changes in their teeth after the procedure. Here are a few points to consider regarding gaps between teeth after scaling:

Possible Causes of Gaps After Scaling.

Removal of Tartar: Scaling removes built-up tartar, which can sometimes create the appearance of gaps if the tartar was holding teeth in closer proximity.

Gum Recession: If scaling is performed on patients with gum disease, it may lead to gum recession. Receding gums can expose more of the tooth's root, potentially creating the appearance of gaps.

Tooth Movement: In some cases, teeth may shift slightly after scaling, especially if there has been significant tartar buildup. This movement can cause gaps.

Improved Oral Hygiene: After scaling, improved oral hygiene practices may lead to healthier gums, which can also change the positioning of teeth over time.

Recommendations.

Consult Your Dentist: If you notice significant gaps or changes in your teeth after scaling, it's best to consult your dentist. They can assess your dental health and provide guidance.

Maintain Oral Hygiene: Regular brushing, flossing, and dental check-ups can help maintain the health of your gums and teeth.

Consider Orthodontic Options: If gaps persist and are a concern, your dentist may recommend orthodontic treatment to address the spacing.

In summary, while dental scaling can lead to changes in the appearance of your teeth, it is important to discuss any concerns with your dental professional for personalized advice.

Q23 Do all gum diseases require the same treatment?

No, not all gum diseases require the same treatment. Gum diseases can vary in severity and type, and the treatment approaches differ accordingly. Here are the main types of gum diseases and their typical treatments:

Types of Gum Diseases and Treatments.

Gingivitis.

Description: Mild inflammation of the gums, often caused by plaque buildup.

Treatment:

Professional dental cleaning.

Improved oral hygiene practices (brushing and flossing).

Antimicrobial mouthwashes.

Periodontitis.

Description: More severe than gingivitis, it involves the inflammation of the supporting structures of the teeth and can lead to tooth loss.

Treatment:

Scaling and root planing (deep cleaning).

Antibiotics to control bacterial infection.

Possible surgical interventions (e.g., flap surgery, bone grafts) for advanced cases.

Aggressive Periodontitis.

Description: A rapid form of periodontitis that occurs in otherwise healthy individuals, often with genetic factors.

Treatment:

Intensive scaling and root planing.

Antibiotics and possibly surgical treatment.

Regular follow-up appointments to monitor the condition.

Chronic Periodontitis.

Description: A common form of periodontitis that progresses slowly and is characterized by gum recession and pocket formation.

Treatment:

Scaling and root planing.

Maintenance cleanings every 3-4 months.

Surgery if pockets remain deep despite treatment.

Necrotizing Periodontal Disease.

Description: A severe form of gum disease often associated with systemic conditions like HIV or malnutrition.

Treatment:

Immediate professional cleaning.

Antibiotics and pain management.

Addressing underlying health issues.

Conclusion.

The treatment for gum disease depends on the type and severity of the condition. It's essential to consult a dental professional for an accurate diagnosis and tailored treatment plan. Regular dental check-ups can help prevent and manage gum diseases effectively.

Q24 Is gum treatment painful?

Gum treatment can vary in terms of pain and discomfort depending on the type of treatment being performed. Here are some common types of gum treatments and their associated pain levels:

Scaling and Root Planing.

Description: A deep cleaning procedure that removes plaque and tartar from below the gum line.

Pain Level: Mild to moderate discomfort; local anesthesia may be used to minimize pain.

Gum Surgery.

Description: Surgical procedures to treat advanced gum disease or to reshape the gums.

Pain Level: Can be more painful; anesthesia is usually administered, and recovery may involve discomfort.

Gum Graft Surgery.

Description: Involves taking tissue from another part of the mouth to cover exposed roots.

Pain Level: Moderate pain during recovery; pain management strategies are typically provided.

Laser Gum Treatment.

Description: Uses laser technology to remove diseased tissue.

Pain Level: Generally less painful than traditional methods; patients often report minimal discomfort.

Antibiotic Treatment.

Description: Involves the application of antibiotics to treat infection.

Pain Level: Usually painless; may cause mild discomfort if there is existing inflammation.

Conclusion.

While some discomfort is common, many dental professionals take steps to minimize pain during gum treatments. It's important to discuss any concerns about pain with your dentist beforehand, as they can provide options for pain management.

Q25 Can gum surgery cause tooth loss?

Gum surgery, also known as periodontal surgery, is generally performed to treat gum disease and improve oral health. However, there are some considerations regarding tooth loss associated with this procedure:

Possible Causes of Tooth Loss After Gum Surgery.

Underlying Gum Disease: If the gum disease is severe, even after surgery, there may be a risk of tooth loss due to the damage already done to the supporting structures of the teeth.

Surgical Complications: Rarely, complications such as infection or improper healing can occur, potentially leading to tooth loss.

Tooth Mobility: In cases where teeth are already loose before surgery, the procedure may not stabilize them, leading to loss.

Bone Loss: If there is significant bone loss due to periodontal disease, the stability of the teeth can be compromised, increasing the risk of loss.

Preventive Measures.

Follow Post-Operative Care: Adhering to the dentist's post-operative instructions can help ensure proper healing and reduce risks.

Regular Check-Ups: Ongoing dental visits can help monitor gum health and catch any issues early.

Good Oral Hygiene: Maintaining excellent oral hygiene can prevent further gum disease and tooth loss.

Conclusion.

While gum surgery aims to preserve teeth and improve gum health, it is essential to understand that tooth loss can still occur, particularly if there are pre-existing conditions or complications. Always consult with your dental professional for personalized advice and treatment options.

Q26 Can gum treatment make my teeth feel loose?

Yes, gum treatment can sometimes make your teeth feel loose. Here are a few reasons why this might happen:

Gum Recession.

During gum treatment, the gums may be reshaped or repositioned, which can expose more of the tooth root. This exposure can create a sensation of looseness.

Inflammation Reduction.

After treatment, the reduction of inflammation in the gums can change how the teeth feel. As the gums heal, they may pull away slightly from the teeth, contributing to a feeling of looseness.

Bone Changes.

If the gum treatment involves scaling or procedures that affect the bone supporting the teeth, it can lead to temporary changes in tooth stability.

Healing Process.

The healing process itself may cause temporary changes in the way your teeth feel. As tissues heal, they may shift slightly, leading to a sensation of looseness.

Recommendations.

Consult Your Dentist: If you experience persistent looseness or discomfort, it's important to follow up with your dentist. They can assess your situation and determine if it's a normal part of the healing process or if further intervention is needed.

Always ensure you maintain good oral hygiene and follow your dentist's post-treatment care instructions to support healing.

Q27 Does tartar removal cause more tartar buildup?

Tartar removal does not directly cause more tartar buildup. However, there are a few considerations regarding this:

Understanding Tartar Buildup.

Tartar Formation: Tartar, or calculus, forms when plaque (a sticky film of bacteria) hardens on the teeth. If not removed through regular brushing and flossing, plaque can mineralize into tartar.

Removal Process: When a dentist or dental hygienist removes tartar, they eliminate the hardened plaque from the teeth.

Factors Influencing Tartar Buildup.

Oral Hygiene: After tartar removal, maintaining good oral hygiene is crucial. If you do not brush and floss regularly, plaque can quickly accumulate again, leading to more tartar.

Diet: A diet high in sugars and starches can contribute to plaque formation, which can lead to more tartar if not managed properly.

Saliva Composition: Some individuals may have saliva that promotes faster tartar formation due to its mineral content.

Conclusion.

While the act of removing tartar itself doesn't lead to increased buildup, neglecting oral hygiene afterward can result in quicker accumulation of plaque and tartar. Regular dental visits and good oral care practices are essential to manage tartar effectively.

Q28 Are antibiotics enough to treat gum disease?

Antibiotics can be a part of the treatment for gum disease, but they are generally not sufficient on their own. Here's a breakdown of their role and the overall treatment approach:

Role of Antibiotics in Gum Disease.

Bacterial Infection: Gum disease, particularly periodontitis, is often caused by bacterial infections. Antibiotics can help reduce the bacterial load.

Adjunctive Therapy: They are typically used as an adjunct to other treatments, such as scaling and root planing (deep cleaning).

Specific Cases: Antibiotics may be prescribed for severe cases or when there is a systemic infection.

Comprehensive Treatment for Gum Disease.

Professional Cleaning:

Scaling and Root Planing: This deep cleaning removes plaque and tartar from below the gum line.

Oral Hygiene:

Daily Care: Brushing twice a day and flossing daily are crucial for managing gum disease.

Regular Dental Visits:

Check-Ups: Regular visits to the dentist for cleanings and check-ups help monitor gum health.

Lifestyle Changes:

Smoking Cessation: Quitting smoking can significantly improve gum health.

Dietary Adjustments: A balanced diet supports overall health and can aid in recovery.

Surgical Options:

In advanced cases, surgical interventions may be necessary to restore gum health.

Conclusion.

While antibiotics can assist in treating gum disease, they should not be relied upon as the sole treatment. A comprehensive approach that includes professional dental care and good oral hygiene is essential for effective management and recovery. If you suspect gum disease, it's best to consult a dental professional for a tailored treatment plan.

Q29 After gum disease treatment, will I ever need treatment again?

After gum disease treatment, it is possible that you may need further treatment in the future. Here are some key points to consider:

Ongoing Maintenance.

Regular dental check-ups and cleanings are essential to maintain gum health.

Your dentist may recommend more frequent visits to monitor your condition.

Risk Factors.

Factors such as smoking, diabetes, or a genetic predisposition can increase the likelihood of gum disease recurrence.

Maintaining good oral hygiene at home is crucial.

Signs of Recurrence.

Be aware of symptoms such as swollen or bleeding gums, persistent bad breath, or loose teeth, which may indicate that gum disease is returning.

Personalized Care.

Each individual's situation is unique, so it's important to follow your dentist's specific recommendations regarding follow-up care.

In summary, while successful treatment can improve your gum health, ongoing maintenance and vigilance are necessary to prevent future issues.

Q30 Is surgery always necessary for gum treatment?

Surgery is not always necessary for gum treatment; it depends on the severity of the gum disease and the specific condition being treated. Here are some key points to consider:

Non-Surgical Treatments.

Scaling and Root Planing: This deep cleaning procedure removes plaque and tartar from below the gum line and smooths the root surfaces.

Antibiotics: Topical or systemic antibiotics may be prescribed to help control bacterial infections.

Improved Oral Hygiene: Regular brushing, flossing, and professional cleanings can help manage gum disease.

Laser Therapy: Some dental practices use lasers to treat gum disease without surgery.

Surgical Treatments.

Surgery may be considered when:

Advanced Periodontitis: If there is significant bone loss or pockets that cannot be cleaned through non-surgical means.

Gum Grafting: To restore gum tissue lost due to recession.

Flap Surgery: To access and clean the roots of teeth in deep pockets.

Conclusion.

It's essential to consult with a dental professional to determine the most appropriate treatment based on individual circumstances. Regular dental check-ups can help catch gum disease early, potentially avoiding the need for surgical intervention.
